# Supplementary material for: Catalytic 4-exo-dig carbocyclization for the construction of furan-fused cyclobutanones and synthetic applications
Source: Nat Commun. 2023 Oct 11;14:6378. doi: 10.1038/s41467-023-42032-9 (PMC10567718; doi:10.1038/s41467-023-42032-9)
Supplement: Supplementary file 1 — Supplementary Information [file 41467_2023_42032_MOESM1_ESM.pdf]

SUPPLEMENTARY INFORMATION

Catalytic *4-exo-dig* carbocyclization for the construction of furan-fused cyclobutanones and synthetic applications

Kemiao Hong[1,§], Yi Zhou[2,§], Haoxuan Yuan[1], Zhijing Zhang[1], Jingjing Huang[1], Shanliang Dong[1], Wenhao Hu[1], Zhi-Xiang Yu[2*] & Xinfang Xu[1*]

[1]Guangdong Key Laboratory of Chiral Molecule and Drug Discovery, School of Pharmaceutical Sciences, Sun Yat-sen University, Guangzhou, Guangdong 510006, China

[2]Beijing National Laboratory for Molecular Sciences (BNLMS), Key Laboratory of Bioorganic Chemistry and Molecular Engineering of Ministry of Education, College of Chemistry, Peking University, Beijing 100871, China.

[§]These authors contributed equally: Kemiao Hong, Yi Zhou

E-mail: yuzx@pku.edu.cn
xuxinfang@mail.sysu.edu.cn

Table of Contents

1. Supplementary Notes S2

2. Supplementary Methods S2

2.1. General procedure for the synthesis of diazo compounds 1 S3-S24

2.2. General procedure for the synthesis of cyclobutanones 2 S25-S41

2.3. General procedure for the gram scale reaction S42

2.4. General procedure for ring opening transformations of 2 S42-S74

2.5. Synthesis of fused and poly-substituted denzene derivatives S75-S81

2.6. Control experiments S81-S83

2.7. NMR Spectra of new compounds S84-S195

2.8. X-Ray crystal structure and data of 2g, 2af, 38, 59, and 71 S196-S200

2.9. General procedure for the *in vitro* anti-tumor activity study S201-S204

2.10. Computational studies S205-S212

3. Supplementary References S213-S215

Supplementary Notes

All reactions were carried out in oven-dried glassware. Solvents were purified by following the standard methods. Flash column chromatography was performed using silica gel (300-400 mesh). Analytical thin-layer chromatography was performed using glass plates pre-coated with 200-300 mesh silica gel impregnated with a fluorescent indicator (254 nm). ^{1}H NMR, ^{13}C NMR and ^{19}F NMR spectra were recorded in $CDCl_3$ on 400 MHz or 500 MHz spectrometer; chemical shifts were reported in ppm with the solvent signal as reference, and coupling constants (*J*) were given in Hertz. The peak information was described as: br = broad, s = singlet, d = doublet, t = triplet, q = quartet, m = multiplet, comp = composite. High-resolution mass spectra (HRMS) were recorded on a commercial apparatus (ESI Source).

Supplementary Methods

General procedure for the synthesis of diazo compounds 1.

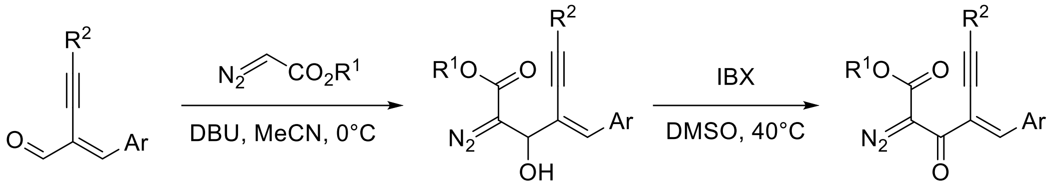

S1 **S2** **1**

The materials **S1** were prepared according to literature procedures[1].

Synthesis of S2: To a 50-mL oven-dried flask containing a magnetic stirring bar, ethyl diazoacetate (EDA, 1.37 g, 12.0 mmol), **S1** (10.0 mmol), MeCN (10.0 mL), and 1,8-diazabicyclo[5.4.0]undec-7-ene (DBU, 0.15 g, 1.0 mmol) were added at 0 ˚C in sequence. After the reaction mixture was stirred at 0 ˚C for 5.0 minutes, the reaction was quenched with saturated aqueous NH_4Cl (20.0 mL), then extracted with CH_2Cl_2 (3 × 20.0 mL). The combined organic phase was dried over Na_2SO_4 and the solvent was evaporated under vacuum after filtration. The obtained product **S2** was directly used for the next step without further purification.

Synthesis of 1: To a 50-mL oven-dried flask containing a magnetic stirring bar, the above obtained product **S2** in DMSO (20.0 mL), was added 2-iodoxybenzoic acid (IBX, 3.4 g, 12.0 mmol) slowly at 40 ˚C. The reaction mixture was stirred for 15.0 minutes under these conditions. Upon completion as indicated by TLC, the reaction was quenched with 20 mL water and extracted with ethyl acetate. The combined organic phase was dried over anhydrous Na_2SO_4 and the solvent was evaporated under vacuum after filtration. The crude material was purified by column chromatography on silica gel (eluent: petroleum ether/ethyl acetate = 10:1) to give the pure products **1** in good to high yields.

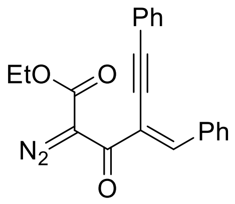

Ethyl (*E*)-4-benzylidene-2-diazo-3-oxo-6-phenylhex-5-ynoate (1a). Yellow oil. 70% yield. ^{1}H NMR (500 MHz, $CDCl_3$) (δ, ppm) 8.04 - 8.03 (m, 2H), 7.51 - 7.49 (m, 2H), 7.44 - 7.39 (comp, 4H), 7.37 - 7.36 (comp, 3H), 4.30 (q, *J* = 7.1 Hz, 2H), 1.29 (t, *J* = 7.1 Hz, 3H); ^{13}C NMR (125 MHz, $CDCl_3$) (δ, ppm) 182.8, 161.3, 143.3, 134.6, 131.5, 130.6, 130.3, 129.1, 128.61, 128.60, 122.6, 120.1, 100.1, 85.2, 75.0, 61.9, 14.3; HRMS (TOF MS ESI$^+$) calculated for $C_{21}H_{17}N_2O_3$ [M+H]$^+$: 345.1234, found 345.1233.

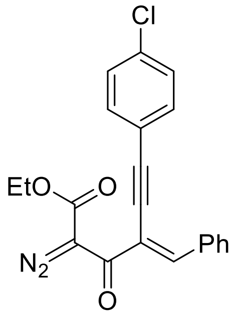

Ethyl (*E*)-4-benzylidene-6-(4-chlorophenyl)-2-diazo-3-oxohex-5-ynoate (1b). Yellow oil. 62% yield. ^{1}H NMR (400 MHz, $CDCl_3$) (δ, ppm) 8.00 - 7.98 (m, 2H), 7.43 - 7.38 (comp, 6H), 7.34 - 7.31 (m, 2H), 4.29 (q, *J* = 7.1 Hz, 2H), 1.28 (t, *J* = 7.1 Hz, 3H); ^{13}C NMR (100 MHz, $CDCl_3$) (δ, ppm) 182.6, 161.0, 143.6, 135.0, 134.4, 132.6, 130.6, 130.2, 128.9, 128.5, 121.0, 119.8, 98.5, 86.1, 75.0, 61.8, 14.3; HRMS (TOF MS ESI$^+$) calculated for $C_{21}H_{16}ClN_2O_3$ [M+H]$^+$: 379.0844, found 379.0847.

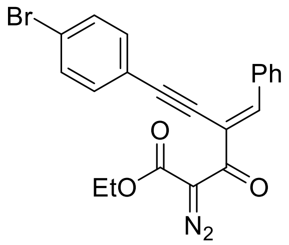

Ethyl (*E*)-4-benzylidene-6-(4-bromophenyl)-2-diazo-3-oxohex-5-ynoate (1c). Yellow oil. 66% yield. ^{1}H NMR (500 MHz, $CDCl_3$) (δ, ppm) 8.01 - 7.99 (m, 2H),

7.52 - 7.50 (m, 2H), 7.44 - 7.42 (comp, 4H), 7.36 - 7.34 (m, 2H), 4.31 (q, J = 7.1 Hz, 2H), 1.30 (t, J = 7.1 Hz, 3H); ^{13}C NMR (126 MHz, $CDCl_3$) (δ, ppm) 182.9, 161.3, 143.8, 134.6, 132.9, 132.0, 130.8, 130.4, 128.7, 123.5, 121.6, 120.0, 98.7, 86.4, 75.3, 62.1, 14.5; HRMS (TOF MS ESI$^+$) calculated for $C_{21}H_{16}BrN_2O_3$ [M+H]$^+$: 423.0339, found 423.0341.

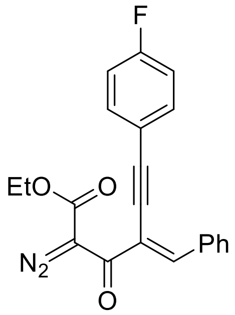

Ethyl (*E*)-4-benzylidene-2-diazo-6-(4-fluorophenyl)-3-oxohex-5-ynoate (1d). Yellow oil. 63% yield. ^{1}H NMR (400 MHz, $CDCl_3$) (δ, ppm) 8.02 - 7.99 (m, 2H), 7.49 - 7.36 (comp, 6H), 7.08 - 7.03 (m, 2H), 4.30 (q, J = 7.1 Hz, 2H), 1.29 (t, J = 7.1 Hz, 3H); ^{13}C NMR (100 MHz, $CDCl_3$) (δ, ppm) 182.7, 162.9 (d, J = 251.1 Hz), 161.1, 143.3, 134.5, 133.4 (d, J = 8.5 Hz), 130.5, 130.2, 128.5, 119.9, 118.7 (d, J = 3.5 Hz), 115.9 (d, J = 22.2 Hz), 98.7, 84.9, 75.0, 61.8, 14.2; ^{19}F NMR (376 MHz, $CDCl_3$) (δ, ppm) -109.31; HRMS (TOF MS ESI$^+$) calculated for $C_{21}H_{16}FN_2O_3$ [M+H]$^+$: 363.1139, found 363.1139.

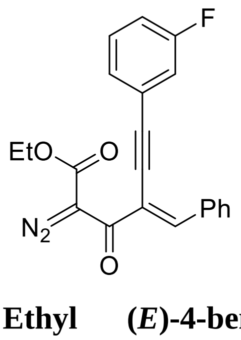

Ethyl (*E*)-4-benzylidene-2-diazo-6-(3-fluorophenyl)-3-oxohex-5-ynoate (1e). Yellow oil. 69% yield. ^{1}H NMR (400 MHz, $CDCl_3$) (δ, ppm) 8.02 - 7.99 (m, 2H), 7.45 - 7.42 (comp, 4H), 7.35 - 7.31 (m, 1H), 7.28 - 7.26 (m, 1H), 7.20 - 7.16 (m, 1H), 7.10 - 7.06 (m, 1H), 4.31 (q, J = 7.1 Hz, 2H), 1.30 (t, J = 7.1 Hz, 3H).; ^{13}C NMR (100

MHz, $CDCl_3$) (δ, ppm) 182.9, 162.5 (d, *J* = 247.1 Hz), 161.2, 144.1, 134.5, 130.8, 130.4, 130.3 (d, *J* = 8.7 Hz), 128.7, 127.5 (d, *J* = 3.0 Hz), 124.5 (d, *J* = 9.5 Hz), 119.9, 118.3 (d, *J* = 22.9 Hz), 116.5 (d, *J* = 21.3 Hz), 98.4, 86.1, 75.3, 62.0, 14.4; ^{19}F NMR (376 MHz, $CDCl_3$) (δ, ppm) -112.31; HRMS (TOF MS ESI$^+$) calculated for $C_{21}H_{16}FN_2O_3$ [M+H]$^+$: 363.1139, found 363.1138.

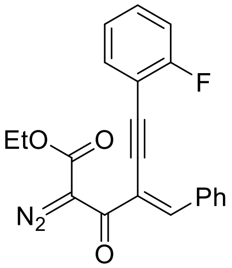

Ethyl (*E*)-4-benzylidene-2-diazo-6-(2-fluorophenyl)-3-oxohex-5-ynoate (1f). Yellow oil. 57% yield. ^{1}H NMR (400 MHz, $CDCl_3$) (δ, ppm) 8.10 - 8.08 (m, 2H), 7.49 - 7.42 (comp, 5H), 7.38 - 7.32 (m, 1H), 7.16 - 7.10 (m, 2H), 4.31 (q, *J* = 7.1 Hz, 2H), 1.30 (t, *J* = 7.1 Hz, 3H); ^{13}C NMR (100 MHz, $CDCl_3$) (δ, ppm) 182.9, 162.8 (d, *J* = 252.6 Hz), 161.3, 143.9, 134.4, 133.2, 130.9, 130.8, 130.5, 128.7, 124.3 (d, *J* = 3.7 Hz), 119.9, 115.8 (d, *J* = 20.6 Hz), 111.4 (d, *J* = 15.6 Hz), 93.8, 90.1, 75.3, 62.0, 14.3; ^{19}F NMR (376 MHz, $CDCl_3$) (δ, ppm) -109.19; HRMS (TOF MS ESI$^+$) calculated for $C_{21}H_{16}FN_2O_3$ [M+H]$^+$: 363.1139, found 363.1138.

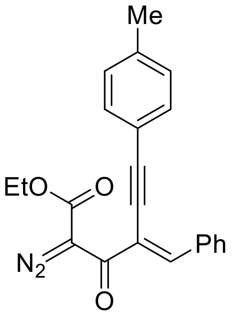

Ethyl (*E*)-4-benzylidene-2-diazo-3-oxo-6-(p-tolyl)hex-5-ynoate (1g). Yellow oil. 74% yield. ^{1}H NMR (400 MHz, $CDCl_3$) (δ, ppm) 8.06 - 8.04 (m, 2H), 7.44 - 7.40 (comp, 6H), 7.18 (d, *J* = 7.9 Hz, 2H), 4.31 (q, *J* = 7.1 Hz, 2H), 2.37 (s, 3H), 1.30 (t, *J* = 7.1 Hz, 3H); ^{13}C NMR (100 MHz, $CDCl_3$) (δ, ppm) 182.7, 161.3, 142.9, 139.4,

134.6, 131.3, 130.5, 130.2, 129.3, 128.5, 120.2, 119.5, 100.5, 84.6, 74.9, 61.9, 21.6, 14.3.; HRMS (TOF MS ESI$^+$) calculated for $C_{22}H_{19}N_2O_3$[M+H]$^+$: 359.1390, found 359.1390.

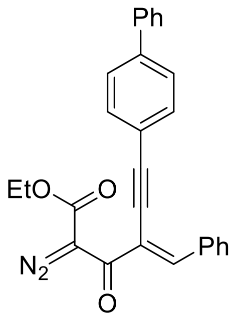

Ethyl (*E*)-6-([1,1'-biphenyl]-4-yl)-4-benzylidene-2-diazo-3-oxohex-5-ynoate (1h). Yellow oil. 73% yield. ^{1}H NMR (500 MHz, $CDCl_3$) (δ, ppm) 8.05 (d, *J* = 7.3 Hz, 2H), 7.62 - 7.56 (comp, 6H), 7.47 - 7.41 (comp, 6H), 7.37 (t, *J* = 7.3 Hz, 1H), 4.32 (q, *J* = 7.1 Hz, 2H), 1.31 (t, *J* = 7.1 Hz, 3H); ^{13}C NMR (126 MHz, $CDCl_3$) (δ, ppm) 182.9, 161.4, 143.4, 141.9, 140.2, 134.7, 132.0, 130.7, 130.4, 129.0, 128.7, 128.0, 127.3, 127.1, 121.5, 120.2, 100.2, 85.9, 75.1, 62.0, 14.4. HRMS (TOF MS ESI$^+$) calculated for $C_{27}H_{21}N_2O_3$ [M+H]$^+$: 421.1547, found 421.1545.

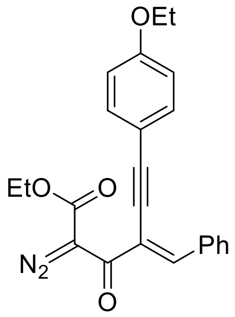

Ethyl (*E*)-4-benzylidene-2-diazo-6-(4-ethoxyphenyl)-3-oxohex-5-ynoate (1i). Yellow oil. 78% yield. ^{1}H NMR (500 MHz, $CDCl_3$) (δ, ppm) 8.04 - 8.02 (m, 2H), 7.44 - 7.38 (comp, 6H), 6.89 - 6.87 (m, 2H), 4.30 (q, *J* = 7.1 Hz, 2H), 4.04 (q, *J* = 7.0 Hz, 2H), 1.41 (t, *J* = 7.0 Hz, 3H), 1.29 (t, *J* = 7.1 Hz, 3H); ^{13}C NMR (125 MHz, $CDCl_3$) (δ, ppm) 182.8, 161.4, 159.7, 142.4, 134.7, 133.0, 130.4, 130.3, 128.6, 120.3, 114.8, 114.4, 100.7, 84.0, 74.8, 63.6, 61.9, 14.8, 14.3; HRMS (TOF MS ESI$^+$) calculated for $C_{23}H_{21}N_2O_4$ [M+H]$^+$: 389.1496, found 389.1499.

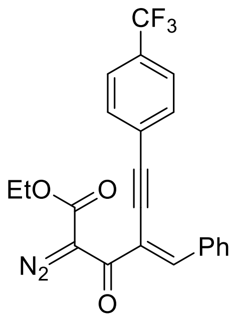

Ethyl (*E*)-4-benzylidene-2-diazo-3-oxo-6-(4-(trifluoromethyl)phenyl)hex-5-ynoate (1j). Yellow oil. 60% yield. ^{1}H NMR (400 MHz, $CDCl_3$) (δ, ppm) 8.02 - 7.99 (m, 2H), 7.63 - 7.58 (comp, 4H), 7.48 (s, 1H), 7.43 - 7.41 (comp, 3H), 4.30 (q, *J* = 7.1 Hz, 2H), 1.29 (t, *J* = 7.1 Hz, 3H); ^{13}C NMR (100 MHz, $CDCl_3$) (δ, ppm) 182.6, 160.9, 144.4, 134.3, 131.6, 130.8, 130.3, 128.6, 126.3, 125.4 (q, *J* = 3.6 Hz), 123.8 (q, *J* = 272.2 Hz), 119.5, 97.8, 87.4, 75.2, 61.8, 14.2; ^{19}F NMR (376 MHz, $CDCl_3$) (δ, ppm) -62.85; HRMS (TOF MS ESI$^+$) calculated for $C_{22}H_{16}F_3N_2O_3$ $[M+H]^+$: 413.1108, found 413.1104.

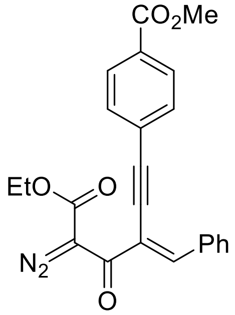

Methyl (*E*)-4-(3-benzylidene-5-diazo-6-ethoxy-4,6-dioxohex-1-yn-1-yl)benzoate (1k). Yellow oil. 67% yield. ^{1}H NMR (500 MHz, $CDCl_3$) (δ, ppm) 8.05 - 8.01 (comp, 4H), 7.55 (d, *J* = 8.0 Hz, 2H), 7.47 (s, 1H), 7.44 - 7.43 (comp, 3H), 4.31 (q, *J* = 7.1 Hz, 2H), 3.93 (s, 3H), 1.30 (t, *J* = 7.1 Hz, 3H); ^{13}C NMR (126 MHz, $CDCl_3$) (δ, ppm) 182.8, 166.4, 161.1, 144.3, 134.4, 131.4, 130.9, 130.4, 130.2, 129.7, 128.7, 127.2, 119.8, 98.7, 88.0, 75.3, 62.0, 52.4, 14.4; HRMS (TOF MS ESI$^+$) calculated for $C_{23}H_{19}N_2O_5$ $[M+H]^+$: 403.1288, found 403.1286.

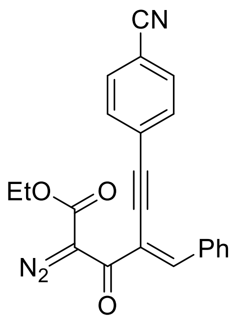

Ethyl (*E*)-4-benzylidene-6-(4-cyanophenyl)-2-diazo-3-oxohex-5-ynoate (1l). Yellow oil. 60% yield. ^{1}H NMR (500 MHz, $CDCl_3$) (δ, ppm) 7.98 (d, *J* = 3.7 Hz, 2H), 7.66 (d, *J* = 8.0 Hz, 2H), 7.57 (d, *J* = 7.8 Hz, 2H), 7.48 (s, 1H), 7.45 - 7.44 (comp, 3H), 4.31 (q, *J* = 7.0 Hz, 2H), 1.30 (t, *J* = 7.1 Hz, 3H); ^{13}C NMR (126 MHz, $CDCl_3$) (δ, ppm) 182.9, 161.0, 145.0, 134.3, 132.3, 132.0, 131.1, 130.4, 128.8, 127.5, 119.5, 118.4, 112.3, 97.4, 89.4, 75.6, 62.1, 14.4; HRMS (TOF MS ESI$^+$) calculated for $C_{22}H_{16}N_3O_3$ [M+H]$^+$: 370.1186, found 370.1184.

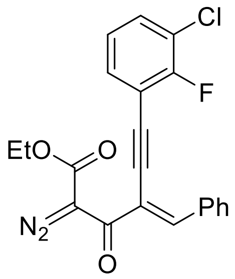

Ethyl (*E*)-4-benzylidene-6-(3-chloro-2-fluorophenyl)-2-diazo-3-oxohex-5-ynoate (1m). Yellow oil. 56% yield. ^{1}H NMR (400 MHz, $CDCl_3$) (δ, ppm) 8.07 - 8.05 (m, 2H), 7.48 - 7.41 (comp, 4H), 7.40 - 7.34 (m, 2H), 7.10 - 7.06 (m, 1H), 4.31 (q, *J* = 7.1 Hz, 2H), 1.30 (t, *J* = 7.1 Hz, 3H); ^{13}C NMR (100 MHz, $CDCl_3$) (δ, ppm) 182.8, 161.1, 158.3 (d, *J* = 254.3 Hz), 144.6, 134.2, 131.3, 131.0, 130.4, 128.7, 124.7 (d, *J* = 4.8 Hz), 121.7 (d, *J* = 17.2 Hz), 119.5, 113.0 (d, *J* = 15.6 Hz), 92.3, 91.1 (d, *J* = 3.6 Hz), 75.4, 62.0, 14.3; ^{19}F NMR (376 MHz, $CDCl_3$) (δ, ppm) -111.30; HRMS (TOF MS ESI$^+$) calculated for $C_{21}H_{15}ClFN_2O_3$ [M+H]$^+$: 397.0750, found 397.0753.

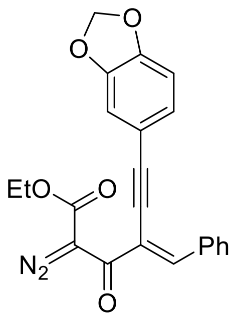

Ethyl (*E*)-6-(benzo[*d*][1,3]dioxol-5-yl)-4-benzylidene-2-diazo-3-oxohex-5-ynoate (1n). Yellow oil. 63% yield. ^{1}H NMR (500 MHz, $CDCl_3$) (δ, ppm) 8.01 (d, *J* = 7.5 Hz, 2H), 7.42 - 7.41 (comp, 4H), 7.03 (d, *J* = 8.0 Hz, 1H), 6.93 (s, 1H), 6.81 (d, *J* = 8.0 Hz, 1H), 6.00 (s, 2H), 4.31 (q, *J* = 7.0 Hz, 2H), 1.31 (t, *J* = 7.1 Hz, 3H); ^{13}C NMR (126 MHz, $CDCl_3$) (δ, ppm) 182.9, 161.4, 148.7, 147.8, 142.9, 134.7, 130.6, 130.3, 128.7, 126.5, 120.2, 115.9, 111.3, 108.8, 101.6, 100.3, 83.8, 75.0, 62.0, 14.4; HRMS (TOF MS ESI$^+$) calculated for $C_{22}H_{17}N_2O_5$ $[M+H]^+$: 389.1132, found 389.1137.

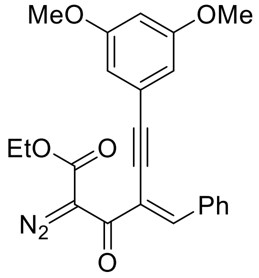

Ethyl (*E*)-4-benzylidene-2-diazo-6-(3,5-dimethoxyphenyl)-3-oxohex-5-ynoate (1o). Yellow oil. 70% yield. ^{1}H NMR (500 MHz, $CDCl_3$) (δ, ppm) 8.03 (d, *J* = 7.6 Hz, 2H), 7.44 - 7.41 (comp, 4H), 6.65 (d, *J* = 1.8 Hz, 2H), 6.50 (s, 1H), 4.32 (q, *J* = 7.1 Hz, 2H), 3.80 (s, 6H), 1.31 (t, *J* = 7.1 Hz, 3H); ^{13}C NMR (126 MHz, $CDCl_3$) (δ, ppm) 182.8, 161.3, 160.7, 143.6, 134.6, 130.7, 130.4, 128.7, 123.9, 120.0, 109.4, 102.3, 100.1, 84.7, 75.2, 62.0, 55.6, 14.4; HRMS (TOF MS ESI$^+$) calculated for $C_{23}H_{21}N_2O_5$ $[M+H]^+$: 405.1445, found 405.1449.

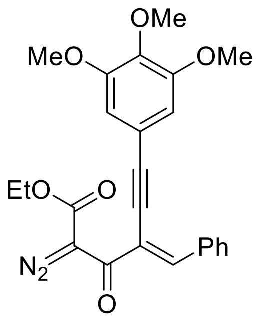

Ethyl (*E*)-4-benzylidene-2-diazo-3-oxo-6-(3,4,5-trimethoxyphenyl)hex-5-ynoate (1p). Yellow oil. 62% yield. ^{1}H NMR (500 MHz, $CDCl_3$) (δ, ppm) 8.04 - 8.02 (m, 2H), 7.47 - 7.42 (comp, 4H), 6.73 (s, 2H), 4.32 (q, J = 7.1 Hz, 2H), 3.88 (s, 9H), 1.31 (t, J = 7.1 Hz, 3H); ^{13}C NMR (126 MHz, $CDCl_3$) (δ, ppm) 182.9, 161.3, 153.3, 143.3, 139.6, 134.7, 130.6, 130.4, 128.6, 120.1, 117.6, 108.8, 100.1, 84.3, 75.2, 62.0, 61.1, 56.3, 14.4; HRMS (TOF MS ESI^{+}) calculated for $C_{24}H_{23}N_2O_6$ $[M+H]^+$: 435.1551, found 435.1552.

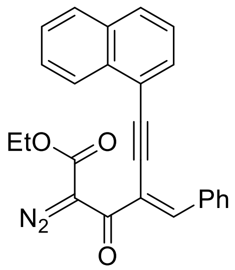

Ethyl (*E*)-4-benzylidene-2-diazo-6-(naphthalen-1-yl)-3-oxohex-5-ynoate (1q). Yellow oil. 72% yield. ^{1}H NMR (400 MHz, $CDCl_3$) (δ, ppm) 8.34 - 8.32 (m, 1H), 8.12 - 8.09 (m, 2H), 7.87 - 7.85 (m, 2H), 7.73 - 7.71 (m, 1H), 7.57 - 7.51 (m, 2H), 7.48 - 7.46 (m, 2H), 7.43 - 7.38 (comp, 3H), 4.29 (q, J = 7.1 Hz, 2H), 1.26 (t, J = 7.1 Hz, 3H); ^{13}C NMR (100 MHz, $CDCl_3$) (δ, ppm) 183.4, 161.2, 143.1, 134.7, 133.3, 133.2, 130.9, 130.6, 130.3, 129.6, 128.7, 128.5, 127.2, 126.7, 126.1, 125.4, 120.7, 120.4, 98.4, 89.8, 75.3, 62.0, 14.4; HRMS (TOF MS ESI^{+}) calculated for $C_{25}H_{19}N_2O_3$ $[M+H]^+$: 395.1390, found 395.1390.

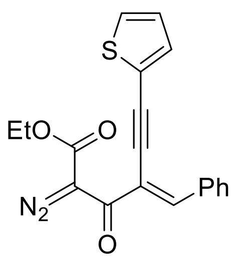

Ethyl (*E*)-4-benzylidene-2-diazo-3-oxo-6-(thiophen-2-yl)hex-5-ynoate (1r). Yellow oil. 71% yield. ^{1}H NMR (500 MHz, $CDCl_3$) (δ, ppm) 7.99 - 7.98 (m, 2H), 7.42 - 7.39 (comp, 4H), 7.35 - 7.34 (m, 1H), 7.28 (dd, *J* = 3.6, 0.5 Hz, 1H), 7.02 (dd, *J* = 5.0, 3.7 Hz, 1H), 4.31 (q, *J* = 7.1 Hz, 2H), 1.30 (t, *J* = 7.1 Hz, 3H); ^{13}C NMR (125 MHz, $CDCl_3$) (δ, ppm) 182.5, 161.1, 143.3, 134.4, 132.5, 130.7, 130.3, 128.61, 128.58, 127.4, 122.4, 119.7, 93.4, 88.9, 75.2, 61.9, 14.3; HRMS (TOF MS ESI^+) calculated for $C_{19}H_{15}N_2O_3S$ $[M+H]^+$: 351.0798, found 351.0795.

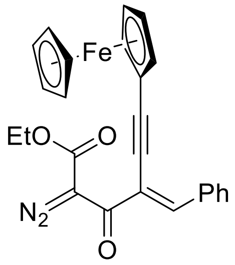

Ethyl (*E*)-4-ferrocene-2-diazo-3-oxo-6-phenylhex-5-ynoate (1s). Red oil. 52% yield. ^{1}H NMR (500 MHz, $CDCl_3$) (δ, ppm) 8.06 (d, *J* = 7.5 Hz, 2H), 7.45 - 7.40 (comp, 4H), 4.52 (s, 2H), 4.35 - 4.31 (comp, 4H), 4.26 (s, 5H), 1.32 (t, *J* = 7.1 Hz, 3H); ^{13}C NMR (126 MHz, $CDCl_3$) (δ, ppm) 182.7, 161.4, 142.1, 134.9, 130.5, 130.2, 128.5, 120.6, 101.0, 81.5, 74.6, 71.5, 70.1, 69.5, 64.3, 61.9, 14.4; HRMS (TOF MS ESI^+) calculated for $C_{25}H_{19}FeN_2O_3$ $[M+H]^+$: 451.0740, found 451.0743.

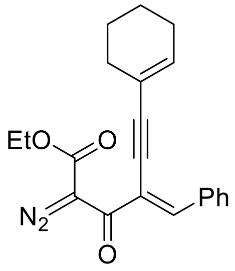

Ethyl (*E*)-4-benzylidene-6-(cyclohex-1-en-1-yl)-2-diazo-3-oxohex-5-ynoate (1t). Yellow oil. 57% yield. ^{1}H NMR (500 MHz, $CDCl_3$) (δ, ppm) 7.99 - 7.97 (m, 2H), 7.40 - 7.38 (comp, 3H), 7.34 (s, 1H), 6.23 (s, 1H), 4.32 (q, *J* = 7.0 Hz, 2H), 2.28 - 2.23 (m, 2H), 2.17 - 2.13 (m, 2H), 1.71 - 1.69 (m, 2H), 1.64 - 1.62 (m, 2H), 1.32 (t, *J* = 7.1 Hz, 3H); ^{13}C NMR (126 MHz, $CDCl_3$) (δ, ppm) 182.9, 161.5, 142.2, 137.0, 134.8, 130.4, 130.3, 128.5, 120.7, 120.5, 102.8, 82.8, 74.7, 61.9, 28.6, 26.0, 22.3, 21.5, 14.4; HRMS (TOF MS ESI$^+$) calculated for $C_{21}H_{21}N_2O_3$ $[M+H]^+$: 349.1547, found 349.1553.

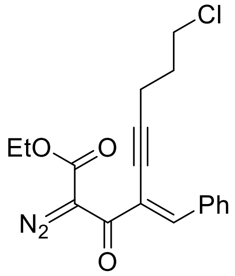

Ethyl (*E*)-4-benzylidene-9-chloro-2-diazo-3-oxonon-5-ynoate (1u). Yellow oil. 62% yield. ^{1}H NMR (400 MHz, $CDCl_3$) (δ, ppm) 7.95 - 7.92 (m, 2H), 7.41 - 7.37 (m, 3H), 7.29 (s, 1H), 4.30 (q, *J* = 7.1 Hz, 2H), 3.68 (t, *J* = 6.3 Hz, 2H), 2.70 (t, *J* = 6.8 Hz, 2H), 2.05 (p, *J* = 6.6 Hz, 2H), 1.31 (t, *J* = 7.1 Hz, 3H); ^{13}C NMR (100 MHz, $CDCl_3$) (δ, ppm) 183.5, 161.2, 142.8, 134.5, 130.4, 130.0, 128.6, 120.4, 99.8, 77.3, 74.8, 61.9, 43.6, 30.9, 17.5, 14.4; HRMS (TOF MS ESI$^+$) calculated for $C_{18}H_{18}ClN_2O_3$ $[M+H]^+$: 345.1000, found 345.1002.

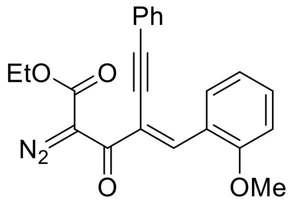

Ethyl (*E*)-2-diazo-4-(2-methoxybenzylidene)-3-oxo-6-phenylhex-5-ynoate (1v). Yellow oil. 73% yield. ^{1}H NMR (500 MHz, $CDCl_3$) (δ, ppm) 8.55 - 8.53 (m, 1H), 7.92 (s, 1H), 7.48 - 7.46 (m, 2H), 7.40 - 7.34 (comp, 4H), 7.00 (t, *J* = 7.6 Hz, 1H), 6.91 (d, *J* = 8.3 Hz, 1H), 4.31 (q, *J* = 7.1 Hz, 2H), 3.86 (s, 3H), 1.30 (t, *J* = 7.1 Hz, 3H); ^{13}C NMR (125 MHz, $CDCl_3$) (δ, ppm) 183.0, 161.6, 158.6, 138.1, 132.2, 131.5, 129.3, 128.9, 128.6, 123.7, 122.9, 120.3, 119.5, 110.8, 99.6, 85.5, 74.9, 61.9, 55.7, 14.4; HRMS (TOF MS ESI$^+$) calculated for $C_{22}H_{19}N_2O_4$ [M+H]$^+$: 375.1339, found 375.1340.

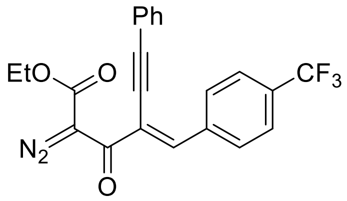

Ethyl (*E*)-2-diazo-3-oxo-6-phenyl-4-(4-(trifluoromethyl)benzylidene)hex-5-ynoate (1w). Yellow oil. 75% yield. ^{1}H NMR (400 MHz, $CDCl_3$) (δ, ppm) 8.12 (d, *J* = 8.2 Hz, 2H), 7.66 (d, *J* = 8.3 Hz, 2H), 7.52 - 7.46 (m, 2H), 7.39 (dd, *J* = 5.9, 3.4 Hz, 4H), 4.31 (q, *J* = 7.1 Hz, 2H), 1.30 (t, *J* = 7.1 Hz, 3H); ^{13}C NMR (125 MHz, $CDCl_3$) (δ, ppm) 182.8, 161.0, 140.5, 137.9, 131.6, 131.5 (q, *J* = 32.6 Hz), 130.2, 129.4, 128.7, 125.5 (q, *J* = 3.7 Hz), 123.9 (q, *J* = 272.3 Hz), 122.7, 122.2, 100.9, 84.6, 75.5, 62.0, 14.3; ^{19}F NMR (471 MHz, $CDCl_3$) (δ, ppm) -62.83; HRMS (TOF MS ESI$^+$) calculated for $C_{22}H_{16}F_3N_2O_3$ [M+H]$^+$: 413.1108, found 413.1108.

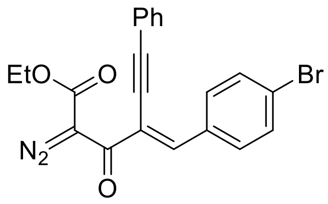

Ethyl (*E*)-4-(4-bromobenzylidene)-2-diazo-3-oxo-6-phenylhex-5-ynoate (1x). Yellow oil. 70% yield. ^{1}H NMR (400 MHz, $CDCl_3$) (δ, ppm) 7.91 - 7.89 (m, 2H), 7.57 - 7.55 (m, 2H), 7.50 - 7.47 (m, 2H), 7.39 - 7.38 (m, 2H), 7.34 (s, 1H), 4.31 (q, *J* = 7.1 Hz, 2H), 1.30 (t, *J* = 7.1 Hz, 3H); ^{13}C NMR (100 MHz, $CDCl_3$) (δ, ppm) 182.9, 161.3, 141.7, 133.6, 132.0, 131.7, 131.6, 129.3, 128.7, 124.9, 122.5, 120.9, 100.9, 84.9, 75.3, 62.1, 14.4; HRMS (TOF MS ESI$^+$) calculated for $C_{21}H_{16}BrN_2O_3$ $[M+H]^+$: 423.0339, found 423.0341.

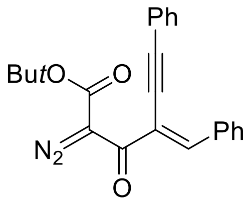

***tert*-Butyl (*E*)-4-benzylidene-2-diazo-3-oxo-6-phenylhex-5-ynoate (1y)** Yellow oil. 60% yield. ^{1}H NMR (500 MHz, $CDCl_3$) (δ, ppm) 8.04 - 8.02 (m, 2H), 7.51 - 7.49 (m, 2H), 7.42 - 7.35 (comp, 7H), 1.49 (s, 9H); ^{13}C NMR (125 MHz, $CDCl_3$) (δ, ppm) 183.5, 160.2, 142.6, 134.6, 131.5, 130.4, 130.2, 129.0, 128.54, 128.53, 122.7, 120.7, 99.9, 85.4, 83.4, 75.8, 28.2; HRMS (TOF MS ESI$^+$) calculated for $C_{23}H_{21}N_2O_3$ $[M+H]^+$: 373.1547, found 373.1541.

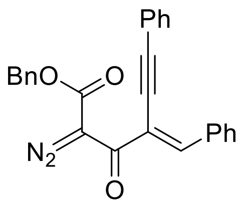

Benzyl (*E*)-4-benzylidene-2-diazo-3-oxo-6-phenylhex-5-ynoate (1z). Yellow oil. 69% yield. ^{1}H NMR (500 MHz, $CDCl_3$) (δ, ppm) 8.01 - 7.99 (m, 2H), 7.45 - 7.40 (comp, 6H), 7.38 - 7.33 (comp, 5H), 7.29 - 7.24 (comp, 3H), 5.28 (s, 2H); ^{13}C NMR

(125 MHz, $CDCl_3$) (δ, ppm) 182.9, 161.3, 143.4, 135.2, 134.6, 131.6, 130.7, 130.4, 129.1, 128.7, 128.67, 128.65, 128.6, 128.4, 122.6, 120.3, 100.3, 85.2, 75.3, 67.5; HRMS (TOF MS ESI^+) calculated for $C_{26}H_{19}N_2O_3$ $[M+H]^+$: 407.1390, found 407.1392.

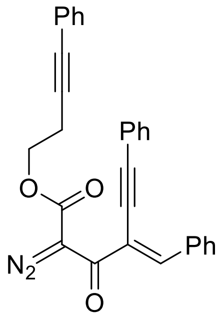

4-Phenylbut-3-yn-1-yl (*E*)-4-benzylidene-2-diazo-3-oxo-6-phenylhex-5-ynoate (1aa). Yellow oil. 41% yield. ^1H NMR (400 MHz, $CDCl_3$) (δ, ppm) 8.04 - 8.02 (m, 2H), 7.51 - 7.48 (m, 2H), 7.45 (s, 1H), 7.43 - 7.40 (comp, 3H), 7.37 - 7.34 (comp, 5H), 7.27 - 7.24 (comp, 3H), 4.43 (t, J = 6.9 Hz, 2H), 2.79 (t, J = 6.9 Hz, 2H); ^{13}C NMR (101 MHz, $CDCl_3$) (δ, ppm) 182.7, 161.0, 143.7, 141.9, 134.6, 131.7, 131.5, 130.7, 130.4, 129.2, 128.7, 128.3, 128.1, 123.2, 122.6, 119.9, 100.3, 85.1, 84.9, 82.5, 75.0, 63.5, 20.1; HRMS (TOF MS ESI^+) calculated for $C_{29}H_{21}N_2O_3$ $[M+H]^+$: 445.1547, found 445.1544.

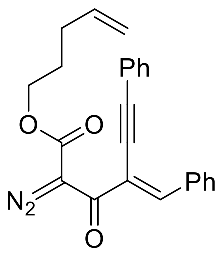

Pent-4-en-1-yl (*E*)-4-benzylidene-2-diazo-3-oxo-6-phenylhex-5-ynoate (1ab). Yellow oil. 49% yield. ^1H NMR (500 MHz, $CDCl_3$) (δ, ppm) 8.05 - 8.03 (m, 2H), 7.51 - 7.49 (m, 2H), 7.43 - 7.42 (comp, 4H), 7.40 - 7.38 (comp, 3H), 5.76 - 5.68 (m, 1H), 4.98 - 4.93 (m, 2H), 4.26 (t, J = 6.5 Hz, 2H), 2.12 - 2.08 (m, 2H), 1.79 - 1.74 (m,

2H); ^{13}C NMR (126 MHz, $CDCl_3$) (δ, ppm) 182.9, 161.5, 143.3, 137.2, 134.6, 131.6, 130.7, 130.4, 129.2, 128.69, 128.67, 122.7, 120.2, 115.5, 100.2, 85.2, 75.2, 65.4, 30.0, 27.9; HRMS (TOF MS ESI$^+$) calculated for $C_{24}H_{21}N_2O_3$ [M+H]$^+$: 385.1547, found 385.1545.

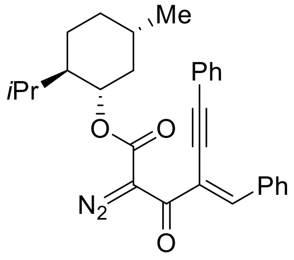

(1*R*,2*S*,5*R*)-2-Isopropyl-5-methylcyclohexyl 4-((*E*)-benzylidene)-2-diazo-3-oxo-6-phenylhex-5-ynoate (1ac). Yellow oil. 50% yield. ^{1}H NMR (400 MHz, $CDCl_3$) (δ, ppm) 8.04 - 8.02 (m, 2H), 7.51 - 7.49 (m, 2H), 7.43 - 7.35 (comp, 7H), 4.85 - 4.79 (m, 1H), 2.08 - 2.05 (m, 1H), 1.89 - 1.85 (m, 1H), 1.66 - 1.62 (m, 2H), 1.46 - 1.34 (comp, 3H), 1.08 - 0.95 (comp, 3H), 0.83 - 0.80 (comp, 6H), 0.73 (d, *J* = 7.0 Hz, 3H); ^{13}C NMR (100 MHz, $CDCl_3$) (δ, ppm) 183.4, 160.7, 142.7, 134.7, 131.6, 130.5, 130.3, 129.0, 128.6, 128.5, 122.7, 120.5, 100.0, 85.3, 76.3, 75.3, 47.0, 41.1, 34.1, 31.4, 26.5, 23.5, 22.0, 20.7, 16.5; HRMS (TOF MS ESI$^+$) calculated for $C_{29}H_{31}N_2O_3$ [M+H]$^+$: 455.2329, found 455.2328.

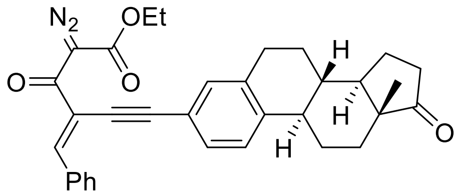

Ethyl 4-((*E*)-benzylidene)-2-diazo-6-((8*S*,9*R*,13*R*,14*R*)-13-methyl-17-oxo-7,8,9,11, 12,13,14,15,16,17-decahydro-6*H*-cyclopenta[*a*]phenanthren-3-yl)-3-oxohex-5-ynoate (1ad). Yellow oil. 66% yield. ^{1}H NMR (400 MHz, $CDCl_3$) (δ, ppm) 8.04 - 8.02 (m, 2H), 7.43 - 7.38 (comp, 4H), 7.29 (s, 2H), 7.24 (s, 1H), 4.31 (q, *J* = 7.1 Hz, 2H), 2.91 (dd, *J* = 8.7, 4.0 Hz, 2H), 2.50 (dd, *J* = 18.6, 8.6 Hz, 2H), 2.34 - 2.28 (m,

1H), 2.15 (dd, J = 18.4, 9.3 Hz, 2H), 2.06 - 2.02 (m, 2H), 1.99 - 1.96 (m, 1H), 1.63 - 1.59 (m, 2H), 1.53 - 1.48 (m, 3H), 1.31 (t, J = 7.1 Hz, 3H), 0.91 (s, 3H); ^{13}C NMR (100 MHz, $CDCl_3$) (δ, ppm) 220.6, 182.7, 161.4, 143.0, 141.3, 137.0, 134.6, 131.9, 130.5, 130.3, 128.8, 128.6, 125.7, 120.2, 119.9, 100.6, 84.6, 74.9, 61.9, 50.5, 47.9, 44.5, 37.9, 35.8, 31.6, 29.2, 26.3, 25.6, 21.6, 14.4, 13.9; HRMS (TOF MS ESI$^+$) calculated for $C_{33}H_{32}N_2O_4Na$ [M+Na]$^+$: 543.2254, found 543.2259.

Synthesis of pyridotriazole 1ae.

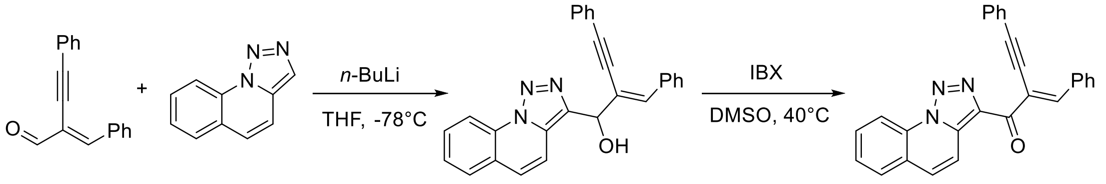

Synthesis of S4: To a stirred solution of compound **S3** (1.0 mmol, 169 mg) in THF was added dropwise *n*-BuLi (0.44 mL, 2.5 M solution in hexane, 1.1 mmol) at -78 °C under argon. After addition, the solution was allowed to stir for 1.0 h at the same temperature. Then, the solution of **S1a** (278 mg, 1.2 mmol) in THF (5.0 mL) was added dropwise and the solution was allowed to warm to room temperature then stirred for 1.0 h. The reaction was quenched with saturated aqueous NH_4Cl (20.0 mL), then extracted with DCM (3 × 20.0 mL). The combined organic phase was dried over Na_2SO_4 and the solvent was evaporated under vacuum after filtration. The crude material was purified by column chromatography on silica gel (eluent: petroleum ether/ethyl acetate = 5:1) to give the pure **S4** (180 mg, 45 % yield).

Synthesis of 1ae: To a 50-mL oven-dried flask containing a magnetic stirring bar, the above obtained crude product **S4** (0.45 mmol) in DMSO (3.0 mL), was added 2-iodoxybenzoic acid (IBX, 150 mg, 0.54 mmol) slowly at 40 ˚C. The reaction mixture was stirred for 15.0 minutes under these conditions. Upon completion as indicated by TLC, the reaction was quenched with 20 mL water and extracted with ethyl acetate, and dried over anhydrous Na_2SO_4. The crude material was purified by column chromatography on silica gel (eluent: petroleum ether / ethyl acetate = 10:1) to give

115 mg pure product **1ae** in 80 % yield; White solid; ^{1}H NMR (500 MHz, $CDCl_3$) (δ, ppm) 8.89 (d, *J* = 8.4 Hz, 1H), 8 .60 (s, 1H), 8.35 (d, *J* = 9.2 Hz, 1H), 8.24 (d, *J* = 7.3 Hz, 2H), 7.96 (d, *J* = 8.0 Hz, 1H), 7.88 - 7.85 (m, 2H), 7.70 (t, *J* = 7.5 Hz, 1H), 7.62 - 7.60 (m, 2H), 7.50 - 7.45 (comp, 3H), 7.38 - 7.37 (comp, 3H); ^{13}C NMR (126 MHz, $CDCl_3$) (δ, ppm) 184.8, 147.7, 138.3, 135.3, 134.7, 131.9, 131.6, 131.2, 131.1, 131.0, 130.8, 128.84, 128.78, 128.7, 128.5, 128.0, 124.6, 123.4, 121.6, 116.8, 116.7, 99.1, 86.4; HRMS (TOF MS ESI$^+$) calculated for $C_{27}H_{17}N_3ONa$ $[M+Na]^+$: 422.1264, found 422.1263.

Synthesis of diazo compound 1af.

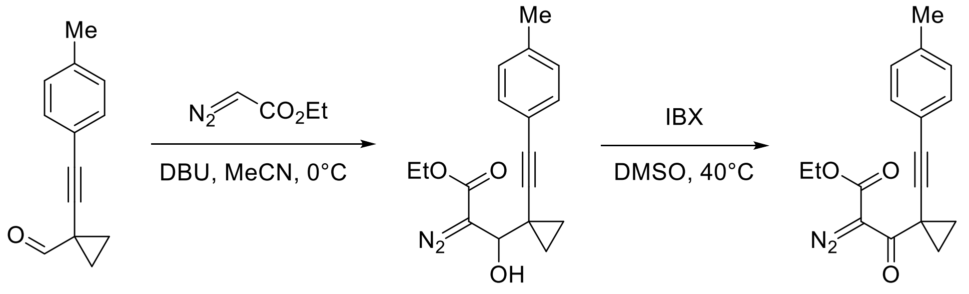

Synthesis of S6: To a 50-mL oven-dried flask containing a magnetic stirring bar, ethyl diazoacetate (EDA, 1.37 g, 12.0 mmol), **S5** (10.0 mmol), MeCN (10.0 mL), and 1,8-diazabicyclo[5.4.0]undec-7-ene (DBU, 0.15 g, 1.0 mmol) were added at 0 ˚C in sequence. After the reaction mixture was stirred at 0 ˚C for 5.0 minutes, the reaction was quenched with saturated aqueous NH_4Cl (20.0 mL), then extracted with CH_2Cl_2 (3 × 20.0 mL). The combined organic phase was dried over Na_2SO_4 and the solvent was evaporated under vacuum after filtration. The obtained product **S6** was directly used for the next step without further purification.

Synthesis of 1af: To a 50-mL oven-dried flask containing a magnetic stirring bar, the above obtained crude product **S6** in DMSO (20.0 mL), was added 2-iodoxybenzoic acid (IBX, 3.4 g, 12.0 mmol) slowly at 40 ˚C. The reaction mixture was stirred for 15.0 minutes under these conditions. Upon completion as indicated by TLC, the reaction was quenched with 20 mL water and extracted with ethyl acetate, and dried

over anhydrous Na_2SO_4. The crude material was purified by column chromatography on silica gel (eluent: petroleum ether/ ethyl acetate = 10:1) to give 1.15 g pure diazoacetate **1af** in 56% yield; Yellow oil; ^{1}H NMR (500 MHz, $CDCl_3$) (δ, ppm) 7.25 (d, *J* = 7.9 Hz, 2H), 7.05 (d, *J* = 7.8 Hz, 2H), 4.27 (q, *J* = 7.1 Hz, 2H), 2.29 (s, 3H), 1.63 - 1.62 (m, 2H), 1.34 - 1.33 (m, 2H), 1.29 (t, *J* = 7.1 Hz, 3H), 0.86 - 0.84 (m, 2H); ^{13}C NMR (126 MHz, $CDCl_3$) (δ, ppm) 185.1, 161.0, 138.2, 131.4, 128.9, 119.5, 87.9, 81.2, 74.2, 61.5, 22.9, 21.2, 19.0, 14.1; HRMS (TOF MS ESI$^+$) calculated for $C_{17}H_{16}N_2O_3Na$ $[M+Na]^+$: 319.1053, found 319.1059.

Synthesis of diazo compound 1ag.

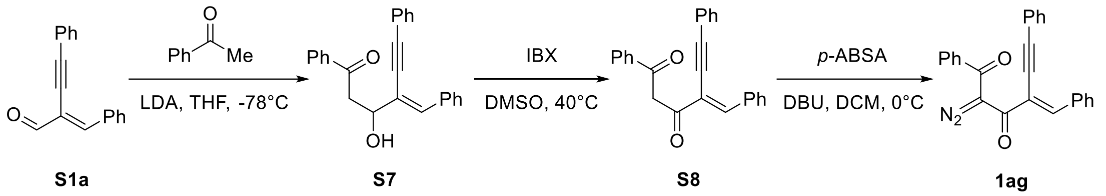

Synthesis of S7: To a stirred solution of disopropyl amine (DIPA, 1.52 g, 15.0 mmol) in THF (20.0 mL) at -78 C was added *n*-BuLi (6.0 mL, 2.5 M solution in hexane, 15.0 mmol) and stirred for 30 minutes at same temperature, The solution of acetophenone (1.80 g, 15.0 mmol) in THF (5.0 mL) was added dropwise to the generated LDA solution at -78 C and the reaction allowed to stir for 30 minutes. Then, the solution of **S1a** (2.32 g, 10.0 mmol) in THF (5.0 mL) was added and the mixture stirred for 30 minutes. After completion of reaction, which was monitored by TLC, it was then quenched with saturated aqueous NH_4Cl (20.0 mL), and extracted with EtOAc (2 × 20.0 mL). The combined organic phase was dried over Na_2SO_4 and the solvent was evaporated under vacuum after filtration. The crude material was purified by column chromatography on silica gel (eluent: petroleum ether/ ethyl acetate = 5:1) to give the pure **S7** (1.80 g, 51% yield).

Synthesis of S8: To a 50-mL oven-dried flask containing a magnetic stirring bar, the above obtained product **S7** (5.0 mmol) in DMSO (10.0 mL), was added 2-iodoxybenzoic acid (IBX, 1.70 g, 6.0 mmol) slowly at 40 ˚C. The reaction mixture

was stirred for 15.0 minutes under these conditions. Upon completion as indicated by TLC, the reaction was quenched with 20 mL water and extracted with ethyl acetate, and dried over anhydrous Na_2SO_4. The obtained product **S8** was directly used for the next step without further purification.

Synthesis of 1ag: To a 50-mL oven-dried flask containing a magnetic stirring bar, 4-acetamidobenzenesulfonyl azide (*p*-ABSA, 1.80 g, 7.5 mmol), crude product **S8** (5.0 mmol), DCM (15.0 mL), and 1,8-diazabicyclo[5.4.0]undec-7-ene (DBU, 1.14 g, 7.5 mmol) were added at 0 ˚C in sequence. After the reaction mixture was stirred at 0 ˚C for 15.0 minutes, the reaction was quenched with saturated aqueous NH_4Cl (20.0 mL), then extracted with DCM (3 × 20.0 mL). The combined organic phase was dried over Na_2SO_4 and the solvent was evaporated under vacuum after filtration. The crude material was purified by column chromatography on silica gel (eluent: petroleum ether/ethyl acetate = 15:1) to give 0.56 g pure diazo compound **1ag** in 15% yield; Yellow oil; ^{1}H NMR (400 MHz, $CDCl_3$) (δ, ppm) 7.92 - 7.90 (m, 2H), 7.68 - 7.66 (m, 2H), 7.58 (s, 1H), 7.46 - 7.38 (comp, 4H), 7.36 - 7.34 (comp, 3H), 7.32 - 7.30 (comp, 4H); ^{13}C NMR (101 MHz, $CDCl_3$) (δ, ppm) 186.6, 182.4, 144.4, 137.3, 134.2, 132.6, 131.3, 130.8, 130.3, 129.1, 128.5, 128.3, 128.2, 122.1, 119.7, 100.8, 85.5, 83.1; HRMS (TOF MS ESI$^+$) calculated for $C_{25}H_{16}N_2O_2Na$ [M+Na]$^+$: 399.1104, found 399.1108.

Synthesis of diazo compound 1ah.

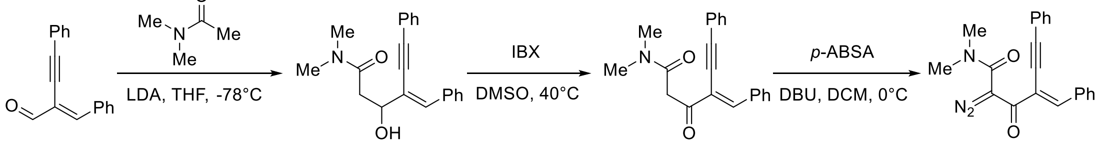

Synthesis of S9: To a stirred solution of disopropyl amine (DIPA, 1.52 g, 15.0 mmol) in THF (20.0 mL) at -78 C was added *n*-BuLi (6.0 mL, 2.5 M solution in hexane, 15.0 mmol) and stirred for 30 minutes at same temperature, The solution of DMA (1.31 g, 15.0 mmol) in THF (5.0 mL) was added dropwise to the generated LDA solution at -

78 C and the reaction allowed to stir for 30 minutes. Then, the solution of **S1a** (2.32 g, 10.0 mmol) in THF (5.0 mL) was added and the mixture stirred for 30 minutes. After completion of reaction, which was monitored by TLC, it was then quenched with saturated aqueous NH_4Cl (20.0 mL), then extracted with EtOAc (2 × 20.0 mL). The combined organic phase was dried over Na_2SO_4 and the solvent was evaporated under vacuum after filtration. The crude material was purified by column chromatography on silica gel (eluent: petroleum ether/ ethyl acetate = 5:1) to give the pure **S6** (1.34 g, 42% yield).

Synthesis of S10: To a 50-mL oven-dried flask containing a magnetic stirring bar, the above obtained product **S9** (4.0 mmol) in DMSO (10.0 mL), was added 2-iodoxybenzoic acid (IBX, 1.34 g, 4.8 mmol) slowly at 40 ˚C. The reaction mixture was stirred for 15.0 minutes under these conditions. Upon completion as indicated by TLC, the reaction was quenched with 20 mL water and extracted with ethyl acetate, and dried over anhydrous Na_2SO_4. The obtained product **S10** was directly used for the next step without further purification.

Synthesis of 1ah: To a 50-mL oven-dried flask containing a magnetic stirring bar, 4-acetamidobenzenesulfonyl azide (*p*-ABSA, 1.44 g, 6.0 mmol), crude product **S7** (5.0 mmol), DCM (15.0 mL), and 1,8-diazabicyclo[5.4.0]undec-7-ene (DBU, 0.91 g, 6.0 mmol) were added at 0 ˚C in sequence. After the reaction mixture was stirred at 0 ˚C for 15.0 minutes, the reaction was quenched with saturated aqueous NH_4Cl (20.0 mL), then extracted with DCM (3 × 20.0 mL). The combined organic phase was dried over Na_2SO_4 and the solvent was evaporated under vacuum after filtration. The crude material was purified by column chromatography on silica gel (eluent: petroleum ether/ethyl acetate = 5:1) to give 0.16 g pure diazo compound **1ah** in 12% yield; Yellow oil; ^{1}H NMR (500 MHz, $CDCl_3$) (δ, ppm) 8.06 - 8.04 (m, 2H), 7.64 (s, 1H), 7.54 - 7.52 (m, 2H), 7.46 - 7.43 (comp, 3H), 7.40 - 7.38 (comp, 3H), 3.01 (s, 6H); ^{13}C NMR (126 MHz, $CDCl_3$) (δ, ppm) 182.1, 161.7, 144.2, 134.5, 131.6, 130.9, 130.6, 129.3, 128.73, 128.72, 122.4, 119.2, 100.9, 85.0, 74.9, 38.9; HRMS (TOF MS ESI$^+$) calculated for $C_{21}H_{17}N_3O_2Na$ $[M+Na]^+$: 366.1213, found 366.1214.

Synthesis of diazo compounds 1ai and 1aj.

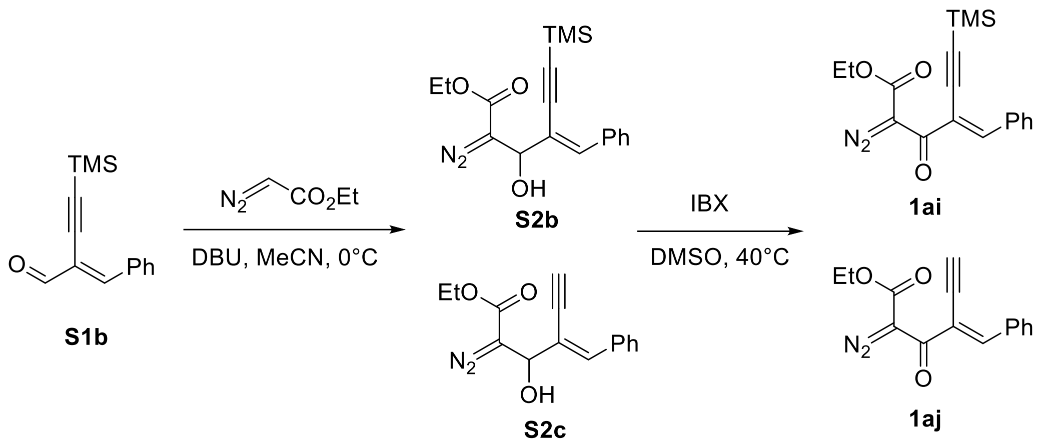

Synthesis of S2b and S2c: To a 50-mL oven-dried flask containing a magnetic stirring bar, ethyl diazoacetate (EDA, 1.37 g, 12.0 mmol), **S1b** (10.0 mmol), MeCN (10.0 mL), and 1,8-diazabicyclo[5.4.0]undec-7-ene (DBU, 0.15 g, 1.0 mmol) were added at 0 ˚C in sequence. After the reaction mixture was stirred at 0 ˚C for 5.0 minutes, the reaction was quenched with saturated aqueous NH_4Cl (20.0 mL), then extracted with CH_2Cl_2 (3 × 20.0 mL). The combined organic phase was dried over Na_2SO_4 and the solvent was evaporated under vacuum after filtration. The obtained products as a mixture of **S2** and **S3** was directly used for the next step without further purification.

Synthesis of 1ai and 1aj: To a 50-mL oven-dried flask containing a magnetic stirring bar, the above obtained mixture of **S2** and **S3** in DMSO (20.0 mL), was added 2-iodoxybenzoic acid (IBX, 3.4 g, 12.0 mmol) slowly at 40 ˚C. The reaction mixture was stirred for 15.0 minutes under these conditions. Upon completion as indicated by TLC, the reaction was quenched with 20 mL water and extracted with ethyl acetate, and dried over anhydrous Na_2SO_4. The crude material was purified by column chromatography on silica gel (eluent: petroleum ether/ ethyl acetate = 10:1) to give the pure diazoacetates **1ai** and **1aj** in combined high yields.

1ai, 1.36 g, 40% yield; Yellow oil; ^{1}H NMR (400 MHz, $CDCl_3$) (δ, ppm) 7.90 - 7.88 (m, 2H), 7.28 - 7.24 (comp, 4H), 4.18 (q, J = 7.1 Hz, 2H), 1.19 (t, J = 7.1 Hz, 3H), 0.15 (s, 9H); ^{13}C NMR (101 MHz, $CDCl_3$) (δ, ppm) 182.1, 161.2, 144.7, 134.3, 130.7, 130.4, 128.4, 120.1, 107.6, 99.96, 74.8, 61.8, 14.3, -0.5; HRMS (TOF MS ESI$^+$)

calculated for $C_{18}H_{20}N_2O_3SiNa$ $[M+Na]^+$: 363.1135, found 363.1139.

1aj, 1.13 g, 42% yield; Yellow oil; ^{1}H NMR (500 MHz, $CDCl_3$) (δ, ppm) 8.00 - 7.98 (m, 2H), 7.41 - 7.40 (comp, 4H), 4.31 (q, *J* = 7.1 Hz, 2H), 3.67 (d, *J* = 0.5 Hz, 1H), 1.31 (t, *J* = 7.1 Hz, 3H); ^{13}C NMR (126 MHz, $CDCl_3$) (δ, ppm) 183.1, 161.1, 145.3, 134.0, 130.9, 130.3, 128.6, 119.3, 88.5, 79.0, 75.3, 61.9, 14.4; HRMS (TOF MS ESI$^+$) calculated for $C_{15}H_{12}N_2O_3Na$ $[M+Na]^+$: 291.0740, found 291.0738.

General procedure for the synthesis of furan-fused cyclobutanones 2

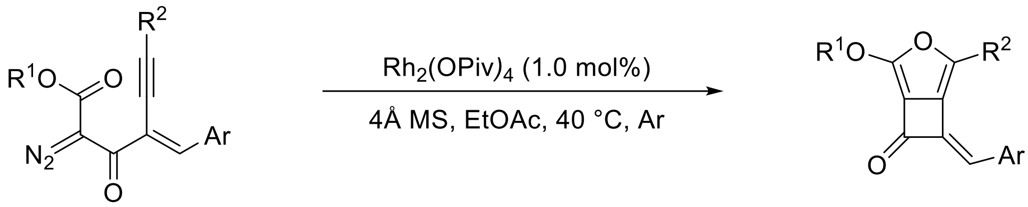

To a 10-mL oven-dried vial containing a magnetic stirring bar, $Rh_2(OPiv)_4$ (1.2 mg, 1.0 mol%), and 4Å MS (100 mg) in EtOAc (1.0 mL), was added as a solution of diazo compounds **1** (0.2 mmol) in the EtOAc (1.0 mL) slowly *via* a syringe at 40 °C under argon atmosphere. After addition, the reaction mixture was stirred for additional 1.0 h under these conditions. Until consumption of the material (monitored by TLC), the reaction mixture was purified by column chromatography on silica gel without any additional treatment (Hexanes : EtOAc = 15:1 to 5:1) or recrystallized from MeOH to give the pure products **2** in good to high yields.

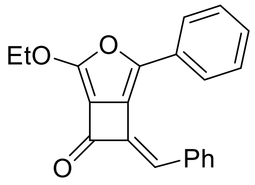

(*E*)-7-Benzylidene-4-ethoxy-2-phenyl-3-oxabicyclo[3.2.0]hepta-1,4-dien-6-one (2a). Yellow solid, m.p.: 131 - 132 °C; 53.2 mg, 84% yield; ^{1}H NMR (400 MHz, $CDCl_3$) (δ, ppm) 7.33 - 7.31 (comp, 3H), 7.29 - 7.22 (comp, 7H), 7.08 (s, 1H), 4.45 (q, *J* = 7.1 Hz, 2H), 1.54 (t, *J* = 7.1 Hz, 3H); ^{13}C NMR (100 MHz, $CDCl_3$) (δ, ppm) 174.6, 149.8, 138.0, 136.0, 133.9, 130.0, 129.6, 129.2, 129.0, 128.6, 128.2, 127.9, 127.6, 121.9, 116.9, 70.4, 14.5; HRMS (TOF MS ESI$^+$) calculated for $C_{21}H_{17}O_3$ [M+H]$^+$: 317.1172, found 317.1172.

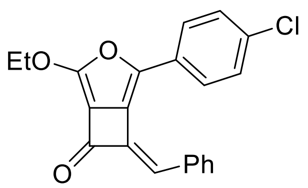

(*E*)-7-Benzylidene-2-(4-chlorophenyl)-4-ethoxy-3-oxabicyclo[3.2.0]hepta-1,4-dien-6-one (2b). Yellow solid, m.p.: 128 - 129 °C; 58.2 mg, 83% yield; ^{1}H NMR (400 MHz, $CDCl_3$) (δ, ppm) 7.37 - 7.34 (m, 1H), 7.29 - 7.24 (comp, 4H), 7.21 - 7.18 (m, 2H), 7.12 - 7.09 (m, 2H), 7.07 (s, 1H), 4.43 (q, *J* = 7.1 Hz, 2H), 1.52 (t, *J* = 7.1 Hz, 3H); ^{13}C NMR (125 MHz, $CDCl_3$) (δ, ppm) 174.2, 149.8, 137.8, 134.6, 133.8, 133.6, 130.1, 129.9, 129.4, 128.7, 128.5, 128.4, 127.4, 122.2, 117.0, 70.5, 14.4; HRMS (TOF MS ESI$^+$) calculated for $C_{21}H_{16}ClO_3$ $[M+H]^+$: 351.0782, found 351.0781.

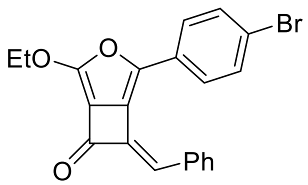

(*E*)-7-Benzylidene-2-(4-bromophenyl)-4-ethoxy-3-oxabicyclo[3.2.0]hepta-1,4-dien-6-one (2c). Yellow solid, m.p.: 139 - 140 °C; 63.2 mg, 80% yield; ^{1}H NMR (400 MHz, $CDCl_3$) (δ, ppm) 7.38 - 7.33 (comp, 3H), 7.29 - 7.24 (comp, 4H), 7.08 (s, 1H), 7.05 - 7.03 (m, 2H), 4.43 (q, *J* = 7.1 Hz, 2H), 1.52 (t, *J* = 7.1 Hz, 3H); ^{13}C NMR (125 MHz, $CDCl_3$) (δ, ppm) 174.2, 149.9, 137.8, 134.6, 133.8, 131.3, 130.2, 129.9, 129.4, 128.71, 128.66, 127.8, 122.3, 121.7, 117.0, 70.5, 14.5; HRMS (TOF MS ESI$^+$) calculated for $C_{21}H_{16}BrO_3$ $[M+H]^+$: 395.0277, found 395.0278.

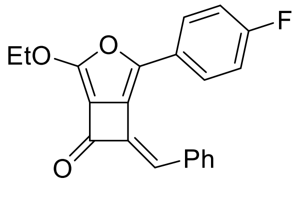

(*E*)-7-Benzylidene-4-ethoxy-2-(4-fluorophenyl)-3-oxabicyclo[3.2.0]hepta-1,4-dien-6-one (2d). Yellow solid, m.p.: 128 - 129 °C; 54.8 mg, 82% yield; ^{1}H NMR (400 MHz, $CDCl_3$) (δ, ppm) 7.36 - 7.32 (m, 1H), 7.29 - 7.21 (comp, 4H), 7.19 - 7.16 (m, 2H), 7.06 (s, 1H), 6.96 - 6.92 (m, 2H), 4.43 (q, *J* = 7.1 Hz, 2H), 1.52 (t, *J* = 7.1 Hz,

3H); ^{13}C NMR (125 MHz, $CDCl_3$) (δ, ppm) 174.3, 162.4 (d, *J* = 248.6 Hz), 149.7, 137.9, 134.8, 133.8, 129.8, 129.4 (d, *J* = 8.2 Hz), 129.3, 129.2, 128.6, 125.3 (d, *J* = 3.2 Hz), 121.9, 116.8, 115.2 (d, *J* = 21.9 Hz), 70.4, 14.4; ^{19}F NMR (376 MHz, $CDCl_3$) (δ, ppm) -112.90. HRMS (TOF MS ESI^+) calculated for $C_{21}H_{16}FO_3$ $[M+H]^+$: 335.1078, found 335.1076.

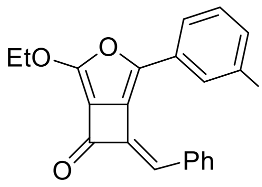

(*E*)-7-Benzylidene-4-ethoxy-2-(3-fluorophenyl)-3-oxabicyclo[3.2.0]hepta-1,4-dien-6-one (2e). Yellow oil. 54.2 mg, 81% yield; ^{1}H NMR (500 MHz, $CDCl_3$) (δ, ppm) 7.38 - 7.36 (m, 1H), 7.34 - 7.32 (m, 2H), 7.29 - 7.26 (m, 2H), 7.22 - 7.17 (m, 1H), 7.12 (s, 1H), 6.98 - 6.93 (comp, 3H), 4.46 (q, *J* = 7.1 Hz, 2H), 1.54 (t, *J* = 7.1 Hz, 3H); ^{13}C NMR (125 MHz, $CDCl_3$) (δ, ppm) 174.3, 162.6 (d, *J* = 246.0 Hz), 150.0, 137.9, 134.5 (d, *J* = 3.0 Hz), 133.8, 130.9 (d, *J* = 8.7 Hz), 130.6, 129.9, 129.8 (d, *J* = 8.5 Hz), 129.5, 128.7, 123.2 (d, *J* = 2.8 Hz), 122.6, 117.1, 114.6 (d, *J* = 21.3 Hz), 114.0 (d, *J* = 23.7 Hz), 70.6, 14.5; ^{19}F NMR (471 MHz, $CDCl_3$) (δ, ppm) -112.98; HRMS (TOF MS ESI^+) calculated for $C_{21}H_{16}FO_3$ $[M+H]^+$: 335.1078, found 335.1079.

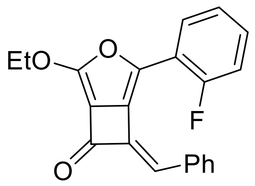

(*E*)-7-Benzylidene-4-ethoxy-2-(2-fluorophenyl)-3-oxabicyclo[3.2.0]hepta-1,4-dien-6-one (2f). Yellow oil. 47.5 mg, 71% yield; ^{1}H NMR (400 MHz, $CDCl_3$) (δ, ppm) 7.37 - 7.31 (m, 1H), 7.30 - 7.23 (comp, 4H), 7.12 - 7.08 (comp, 3H), 7.04 - 6.99 (m, 2H), 4.45 (q, *J* = 7.1 Hz, 2H), 1.53 (t, *J* = 7.1 Hz, 3H); ^{13}C NMR (100 MHz, $CDCl_3$) (δ, ppm) 174.2, 159.3 (d, *J* = 251.7 Hz), 150.3, 137.5, 133.9, 132.9 (d, *J* = 1.5 Hz), 131.0 (d, *J* = 1.9 Hz), 130.4 (d, *J* = 8.0 Hz), 129.7, 129.4, 129.2, 128.4, 123.9 (d, *J* = 3.7 Hz), 122.2, 117.8 (d, *J* = 13.3 Hz), 116.8, 116.0 (d, *J* = 21.1 Hz), 70.5, 14.5;

^{19}F NMR (376 MHz, $CDCl_3$) (δ, ppm) -111.67; HRMS (TOF MS ESI$^+$) calculated for $C_{21}H_{15}FO_3Na$ [M+Na]$^+$: 357.0897, found 357.0892.

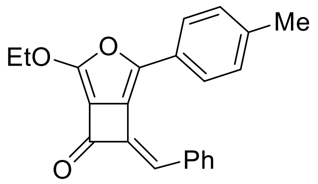

(*E*)-7-Benzylidene-4-ethoxy-2-(*p*-tolyl)-3-oxabicyclo[3.2.0]hepta-1,4-dien-6-one (2g). Yellow solid, m.p.: 109 - 110 °C; 59.5 mg, 90% yield; ^{1}H NMR (400 MHz, $CDCl_3$) (δ, ppm) 7.35 - 7.32 (m, 3H), 7.26 - 7.23 (m, 2H), 7.15 (d, *J* = 8.0 Hz, 2H), 7.07 (d, *J* = 10.3 Hz, 3H), 4.44 (q, *J* = 7.1 Hz, 2H), 2.37 (s, 3H), 1.53 (t, *J* = 7.1 Hz, 3H); ^{13}C NMR (100 MHz, $CDCl_3$) (δ, ppm) 174.7, 149.5, 138.0, 137.9, 136.3, 133.9, 130.0, 129.2, 128.9, 128.8, 128.6, 127.6, 126.4, 121.6, 116.7, 70.3, 21.5, 14.5; HRMS (TOF MS ESI$^+$) calculated for $C_{22}H_{19}O_3$ [M+H]$^+$: 331.1329, found 331.1325.

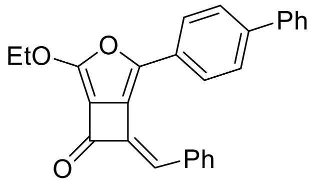

(*E*)-2-([1,1'-Biphenyl]-4-yl)-7-benzylidene-4-ethoxy-3-oxabicyclo[3.2.0]hepta-1,4-dien-6-one (2h). Yellow solid, m.p.: 177 - 178 °C; 67.5 mg, 86% yield; ^{1}H NMR (500 MHz, $CDCl_3$) (δ, ppm) 7.48 - 7.47 (m, 2H), 7.36 - 7.33 (comp, 4H), 7.24 - 7.21 (comp, 4H), 7.16 - 7.14 (comp, 4H), 6.97 (s, 1H), 4.32 (q, *J* = 6.1 Hz, 2H), 1.41 (t, *J* = 6.1 Hz, 3H); ^{13}C NMR (126 MHz, $CDCl_3$) (δ, ppm) 174.5, 149.7, 140.4, 140.3, 137.9, 135.6, 133.8, 130.0, 129.8, 129.2, 128.9, 128.6, 127.9, 127.8, 127.6, 127.0, 126.7, 121.9, 116.9, 70.4, 14.4; HRMS (TOF MS ESI$^+$) calculated for $C_{27}H_{21}O_3$ [M+H]$^+$: 393.1485, found 393.1488.

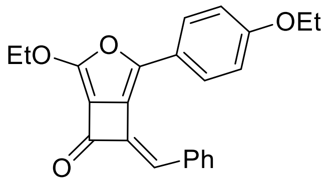

(*E*)-7-Benzylidene-4-ethoxy-2-(4-ethoxyphenyl)-3-oxabicyclo[3.2.0]hepta-1,4-dien-6-one (2i). Yellow solid, m.p.: 145 - 146 °C; 66.3 mg, 92% yield; ^{1}H NMR (400 MHz, $CDCl_3$) (δ, ppm) 7.34 - 7.30 (comp, 3H), 7.25 - 7.21 (m, 2H), 7.19 - 7.15 (m, 2H), 7.03 (s, 1H), 6.81 - 6.78 (m, 2H), 4.43 (q, *J* = 7.1 Hz, 2H), 4.05 (q, *J* = 7.0 Hz, 2H), 1.52 (t, *J* = 7.1 Hz, 3H), 1.43 (t, *J* = 7.0 Hz, 3H); ^{13}C NMR (100 MHz, $CDCl_3$) (δ, ppm) 174.8, 158.9, 149.3, 138.0, 136.2, 134.0, 129.9, 129.3, 129.1, 128.6, 128.0, 121.9, 121.2, 116.5, 114.1, 70.2, 63.7, 14.9, 14.5; HRMS (TOF MS ESI$^+$) calculated for $C_{23}H_{21}O_4$ $[M+H]^+$: 361.1434, found 361.1432.

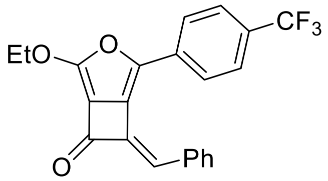

(*E*)-7-Benzylidene-4-ethoxy-2-(4-(trifluoromethyl)phenyl)-3-oxabicyclo[3.2.0]hepta-1,4-dien-6-one (2j). Yellow solid, m.p.: 146 - 147 °C; 56.1 mg, 73% yield; ^{1}H NMR (400 MHz, $CDCl_3$) (δ, ppm) 7.47 (d, *J* = 8.2 Hz, 2H), 7.39 (t, *J* = 6.9 Hz, 1H), 7.32 - 7.26 (comp, 6H), 7.14 (s, 1H), 4.47 (q, *J* = 7.1 Hz, 2H), 1.55 (t, *J* = 7.1 Hz, 3H); ^{13}C NMR (100 MHz, $CDCl_3$) (δ, ppm) 174.0, 150.4, 137.9, 134.2, 133.8, 132.1, 131.8, 130.0, 129.6, 129.3 (q, *J* = 32.6 Hz), 128.7, 127.1, 125.2 (q, *J* = 3.8 Hz), 124.2 (q, *J* = 271.9 Hz), 122.9, 117.5, 70.75, 14.48; ^{19}F NMR (376 MHz, $CDCl_3$) (δ, ppm) -62.47; HRMS (TOF MS ESI$^+$) calculated for $C_{22}H_{16}F_3O_3$ $[M+H]^+$: 385.1046, found 385.1044.

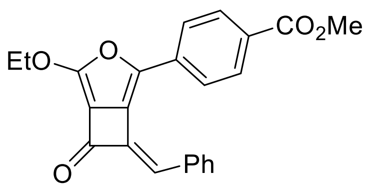

Methyl (*E*)-4-(7-benzylidene-4-ethoxy-6-oxo-3-oxabicyclo[3.2.0]hepta-1,4-dien-2 -yl)benzoate (2k). Yellow solid, m.p.: 114 - 115 °C; 58.4 mg, 78% yield; ^{1}H NMR (500 MHz, $CDCl_3$) (δ, ppm) 7.79 (d, *J* = 7.4 Hz, 2H), 7.30 - 7.29 (m, 1H), 7.23 - 7.18 (comp, 4H), 7.14 (d, *J* = 7.4 Hz, 2H), 7.03 (s, 1H), 4.39 - 4.35 (m, 2H), 3.83 (s, 3H), 1.45 (t, *J* = 7.0 Hz, 3H); ^{13}C NMR (126 MHz, $CDCl_3$) (δ, ppm) 174.0, 166.8, 150.3, 137.8, 134.7, 133.6, 132.8, 132.0, 129.9, 129.6, 129.5, 128.7, 126.7, 122.8, 117.5, 70.7, 52.3, 14.4; HRMS (TOF MS ESI^+) calculated for $C_{23}H_{19}O_5$ $[M+H]^+$: 375.1227, found 375.1229.

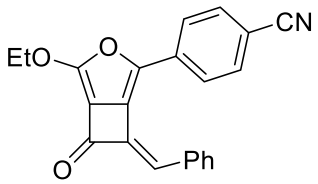

(*E*)-4-(7-Benzylidene-4-ethoxy-6-oxo-3-oxabicyclo[3.2.0]hepta-1,4-dien-2-yl) benzonitrile (2l). Yellow solid, m.p.: 175 - 176 °C; 51.9 mg, 76% yield; ^{1}H NMR (500 MHz, $CDCl_3$) (δ, ppm) 7.40 (d, *J* = 8.3 Hz, 2H), 7.35 - 7.32 (m, 1H), 7.23 - 7.22 (comp, 4H), 7.14 (d, *J* = 8.3 Hz, 2H), 7.09 (s, 1H), 4.40 (q, *J* = 7.0 Hz, 2H), 1.47 (t, *J* = 7.1 Hz, 3H); ^{13}C NMR (126 MHz, $CDCl_3$) (δ, ppm) 173.6, 150.7, 137.8, 133.7, 133.6, 133.1, 132.7, 132.0, 129.9, 129.7, 128.8, 126.9, 123.4, 118.9, 117.8, 110.4, 70.9, 14.5; HRMS (TOF MS ESI^+) calculated for $C_{22}H_{16}NO_3$ $[M+H]^+$: 342.1125, found 342.1126.

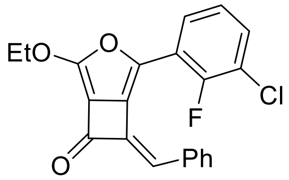

(*E*)-7-Benzylidene-2-(3-chloro-2-fluorophenyl)-4-ethoxy-3-oxabicyclo[3.2.0]hepta -1,4-dien-6-one (2m). Yellow oil. 44.3 mg, 60% yield; ^{1}H NMR (500 MHz, $CDCl_3$)

(δ, ppm) 7.39 (t, *J* = 7.1 Hz, 1H), 7.31 - 7.26 (comp, 3H), 7.20 - 7.13 (comp, 3H), 7.05 - 7.02 (m, 2H), 4.47 (q, *J* = 6.5 Hz, 2H), 1.54 (t, *J* = 6.5 Hz, 3H); ^{13}C NMR (126 MHz, $CDCl_3$) (δ, ppm) 174.0, 154.7 (d, *J* = 254.1 Hz), 150.5, 137.5, 133.9, 130.9, 130.5, 129.5, 129.3, 128.9, 128.8, 128.5, 124.3 (d, *J* = 4.8 Hz), 122.9, 122.0 (d, *J* = 17.5 Hz), 119.4 (d, *J* = 13.2 Hz), 117.0, 70.7, 14.5; ^{19}F NMR (471 MHz, $CDCl_3$) (δ, ppm) -113.27; HRMS (TOF MS ESI$^+$) calculated for $C_{21}H_{15}ClFO_3$ $[M+H]^+$: 369.0688, found 369.0686.

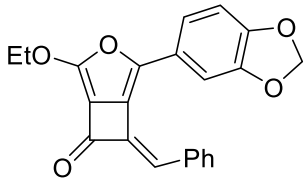

(*E*)-2-(Benzo[*d*][1,3]dioxol-5-yl)-7-benzylidene-4-ethoxy-3-oxabicyclo[3.2.0]hepta-1,4-dien-6-one (2n). Yellow solid, m.p.: 129 - 130 °C; 62.7 mg, 87% yield; ^{1}H NMR (500 MHz, $CDCl_3$) (δ, ppm) 7.26 - 7.20 (comp, 3H), 7.18 - 7.15 (m, 2H), 6.95 (s, 1H), 6.71 (s, 1H), 6.57 (d, *J* = 8.0 Hz, 1H), 6.51 (d, *J* = 8.1 Hz, 1H), 5.88 (s, 2H), 4.33 (q, *J* = 7.0 Hz, 2H), 1.43 (t, *J* = 7.1 Hz, 3H); ^{13}C NMR (126 MHz, $CDCl_3$) (δ, ppm) 174.6, 149.2, 147.6, 147.5, 137.9, 135.7, 133.8, 129.9, 129.2, 128.5, 128.3, 123.2, 122.6, 121.5, 116.5, 108.0, 107.6, 101.4, 70.3, 14.4; HRMS (TOF MS ESI$^+$) calculated for $C_{22}H_{17}O_5$ $[M+H]^+$: 361.1071, found 361.1074.

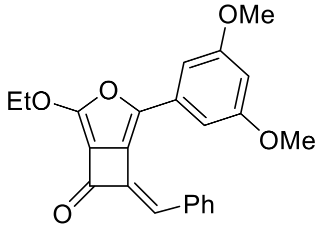

(*E*)-7-Benzylidene-2-(3,5-dimethoxyphenyl)-4-ethoxy-3-oxabicyclo[3.2.0]hepta-1,4-dien-6-one (2o). Yellow solid, m.p.: 146 - 147 °C; 67.8 mg, 90% yield; ^{1}H NMR (500 MHz, $CDCl_3$) (δ, ppm) 7.25 - 7.23 (comp, 3H), 7.17 - 7.14 (m, 2H), 6.98 (s, 1H), 6.32 - 6.30 (comp, 3H), 4.36 (q, *J* = 7.0 Hz, 2H), 3.45 (s, 6H), 1.45 (t, *J* = 7.1 Hz, 3H); ^{13}C NMR (126 MHz, $CDCl_3$) (δ, ppm) 174.4, 160.4, 149.6, 137.7, 135.8, 133.7, 130.6,

130.0, 129.9, 129.3, 128.6, 121.9, 116.7, 105.5, 101.3, 70.4, 55.2, 14.5; HRMS (TOF MS ESI$^+$) calculated for $C_{23}H_{21}O_5$ [M+H]$^+$: 377.1384, found 377.1387.

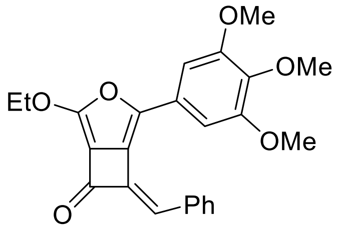

(*E*)-7-Benzylidene-4-ethoxy-2-(3,4,5-trimethoxyphenyl)-3-oxabicyclo[3.2.0]hepta-1,4-dien-6-one (2p). Yellow oil. 74.8 mg, 92% yield; ^{1}H NMR (500 MHz, $CDCl_3$) (δ, ppm) 7.33 - 7.29 (comp, 3H), 7.24 - 7.21 (m, 2H), 7.06 (s, 1H), 6.44 (s, 2H), 4.45 (q, *J* = 7.1 Hz, 2H), 3.87 (s, 3H), 3.52 (s, 6H), 1.54 (t, *J* = 7.1 Hz, 3H); ^{13}C NMR (126 MHz, $CDCl_3$) (δ, ppm) 174.3, 152.8, 149.5, 137.9, 137.7, 135.8, 133.8, 130.0, 129.3, 129.2, 128.6, 124.5, 121.6, 116.6, 105.0, 70.4, 61.0, 55.7, 14.4; HRMS (TOF MS ESI$^+$) calculated for $C_{24}H_{23}O_6$ [M+H]$^+$: 407.1489, found 407.1485.

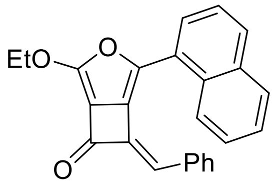

(*E*)-7-Benzylidene-4-ethoxy-2-(naphthalen-1-yl)-3-oxabicyclo[3.2.0]hepta-1,4-dien-6-one (2q). Yellow solid, m.p.: 122 - 123 °C; 58.6 mg, 80% yield; ^{1}H NMR (500 MHz, $CDCl_3$) (δ, ppm) 8.13 (d, *J* = 8.5 Hz, 1H), 7.91 (d, *J* = 7.8 Hz, 1H), 7.86 (d, *J* = 8.1 Hz, 1H), 7.45 - 7.40 (comp, 3H), 7.28 - 7.26 (m, 2H), 7.10 (d, *J* = 7.6 Hz, 2H), 7.01 (s, 1H), 6.74 (t, *J* = 7.6 Hz, 2H), 4.51 (q, *J* = 7.0 Hz, 2H), 1.57 (t, *J* = 7.0 Hz, 3H); ^{13}C NMR (125 MHz, $CDCl_3$) (δ, ppm) 174.5, 150.2, 137.7, 134.6, 133.5, 133.3, 132.5, 132.0, 129.58, 129.56, 129.4, 129.1, 128.1, 128.0, 126.5, 126.4, 126.3, 125.9, 124.8, 122.0, 116.6, 70.4, 14.5; HRMS (TOF MS ESI$^+$) calculated for $C_{25}H_{19}O_3$ [M+H]$^+$: 367.1329, found 367.1330.

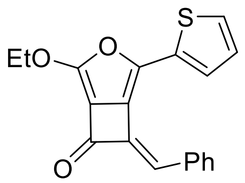

(*E*)-7-Benzylidene-4-ethoxy-2-(thiophen-2-yl)-3-oxabicyclo[3.2.0]hepta-1,4-dien-6-one (2r). Yellow solid, m.p.: 124 - 125 °C; 47.7 mg, 74% yield; ^{1}H NMR (400 MHz, $CDCl_3$) (δ, ppm) 7.38 - 7.32 (comp, 4H), 7.28 - 7.25 (m, 2H), 7.04 (s, 1H), 6.99 (dd, *J* = 5.0, 3.7 Hz, 1H), 6.79 (dd, *J* = 3.6, 1.1 Hz, 1H), 4.44 (q, *J* = 7.1 Hz, 2H), 1.53 (t, *J* = 7.1 Hz, 3H); ^{13}C NMR (100 MHz, $CDCl_3$) (δ, ppm) 174.3, 149.4, 137.4, 133.9, 130.9, 130.5, 130.0, 129.9, 129.4, 128.9, 128.7, 127.1, 126.6, 122.1, 116.7, 70.5, 14.5; HRMS (TOF MS ESI$^+$) calculated for $C_{19}H_{15}O_3S$ $[M+H]^+$: 323.0736, found 323.0738.

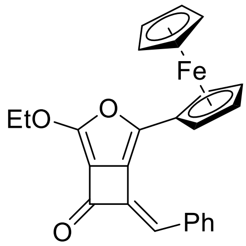

(*E*)-7-Ferrocene-4-ethoxy-2-phenyl-3-oxabicyclo[3.2.0]hepta-1,4-dien-6-one (2s). Red solid, m.p.: 139 - 140 °C; 65.3 mg, 77% yield; ^{1}H NMR (500 MHz, $CDCl_3$) (δ, ppm) 7.43 (d, *J* = 7.3 Hz, 2H), 7.34 - 7.26 (comp, 3H), 6.97 (s, 1H), 4.46 - 4.42 (comp, 4H), 4.27 (d, *J* = 1.3 Hz, 2H), 4.16 (s, 5H), 1.55 (t, *J* = 7.1 Hz, 3H); ^{13}C NMR (126 MHz, $CDCl_3$) (δ, ppm) 174.9, 148.6, 137.9, 135.7, 134.1, 129.8, 129.2, 128.8, 128.5, 120.8, 116.1, 75.0, 70.2, 69.5, 68.8, 68.8, 14.5; HRMS (TOF MS ESI$^+$) calculated for $C_{25}H_{21}FeO_3$ $[M+H]^+$: 425.0835, found 425.0838.

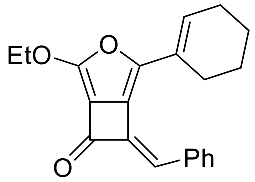

(*E*)-7-Benzylidene-2-(cyclohex-1-en-1-yl)-4-ethoxy-3-oxabicyclo[3.2.0]hepta-1,4-dien-6-one (2t). Yellow oil. 54.5 mg, 85% yield; ^{1}H NMR (500 MHz, $CDCl_3$) (δ, ppm) 7.24 - 7.23 (comp, 5H), 6.90 (s, 1H), 5.25 (s, 1H), 4.32 - 4.27 (m, 2H), 2.27 -

2.26 (m, 2H), 1.96 - 1.95 (m, 2H), 1.63 - 1.58 (comp, 4H), 1.42 - 1.39 (m, 3H); ^{13}C NMR (126 MHz, $CDCl_3$) (δ, ppm) 174.9, 148.5, 139.1, 138.2, 134.0, 132.1, 129.7, 128.8, 128.20, 128.16, 127.0, 120.7, 115.8, 70.0, 26.3, 25.5, 22.4, 22.1, 14.4; HRMS (TOF MS ESI$^+$) calculated for $C_{21}H_{21}O_3$ [M+H]$^+$: 321.1485, found 321.1488.

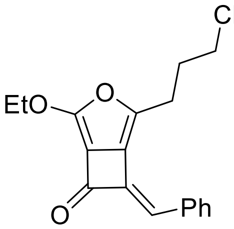

(*E*)-7-Benzylidene-2-(3-chloropropyl)-4-ethoxy-3-oxabicyclo[3.2.0]hepta-1,4-dien-6-one (2u). Yellow oil. 40.6 mg, 64% yield; ^{1}H NMR (500 MHz, $CDCl_3$) (δ, ppm) 7.56 (d, *J* = 7.3 Hz, 2H), 7.43 (t, *J* = 7.5 Hz, 2H), 7.36 (t, *J* = 7.3 Hz, 1H), 6.90 (s, 1H), 4.37 (q, *J* = 7.1 Hz, 2H), 3.58 (t, *J* = 6.2 Hz, 2H), 2.95 (t, *J* = 7.3 Hz, 2H), 2.17 - 2.11 (m, 2H), 1.49 (t, *J* = 7.1 Hz, 3H); ^{13}C NMR (125 MHz, $CDCl_3$) (δ, ppm) 174.4, 149.3, 138.1, 135.4, 134.6, 129.4, 129.2, 129.0, 128.7, 121.0, 115.3, 70.2, 44.1, 31.9, 25.2, 14.5; HRMS (TOF MS ESI$^+$) calculated for $C_{18}H_{18}ClO_3$ [M+H]$^+$: 317.0939, found 317.0936.

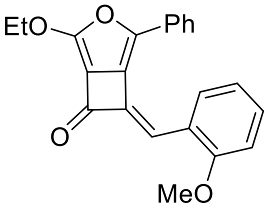

(*E*)-4-Ethoxy-7-(2-methoxybenzylidene)-2-phenyl-3-oxabicyclo[3.2.0]hepta-1,4-dien-6-one (2v). Yellow solid, mp = 141 - 142 ^{o}C. 58.2 mg, 84% yield; ^{1}H NMR (500 MHz, $CDCl_3$) (δ, ppm) 7.36 (s, 1H), 7.34 - 7.31 (m, 1H), 7.25 - 7.21 (comp, 5H), 7.17 - 7.15 (m, 1H), 6.89 (d, *J* = 8.3 Hz, 1H), 6.71 (t, *J* = 7.4 Hz, 1H), 4.45 (q, *J* = 7.1 Hz, 2H), 3.79 (s, 3H), 1.53 (t, *J* = 7.1 Hz, 3H); ^{13}C NMR (125 MHz, $CDCl_3$) (δ, ppm) 174.9, 158.3, 149.6, 138.1, 135.8, 130.9, 130.7, 130.0, 129.2, 128.2, 127.5, 127.1, 123.0, 120.4, 117.8, 116.7, 110.4, 70.3, 55.5, 14.5; HRMS (TOF MS ESI$^+$) calculated for $C_{22}H_{19}O_4$ [M+H]$^+$: 347.1278, found 347.1273.

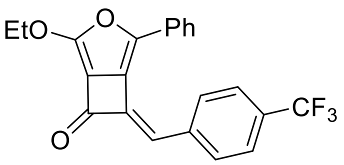

(*E*)-4-Ethoxy-2-phenyl-7-(4-(trifluoromethyl)benzylidene)-3-oxabicyclo[3.2.0] hepta-1,4-dien-6-one (2w). Yellow solid, mp = 147 - 148 °C. 63.8 mg, 83% yield; ^{1}H NMR (400 MHz, $CDCl_3$) (δ, ppm) 7.47 (d, *J* = 8.2 Hz, 2H), 7.38 (d, *J* = 8.2 Hz, 2H), 7.34 - 7.30 (m, 1H), 7.27 - 7.23 (m, 2H), 7.15 - 7.13 (m, 2H), 7.07 (s, 1H), 4.46 (q, *J* = 7.1 Hz, 2H), 1.54 (t, *J* = 7.1 Hz, 3H); ^{13}C NMR (125 MHz, $CDCl_3$) (δ, ppm) 173.8, 150.0, 140.2, 137.4, 136.2, 130.7 (q, *J* = 32.7 Hz), 130.0, 129.1, 128.8, 128.4, 128.2, 127.6, 125.6 (q, *J* = 3.7 Hz), 124.1 (q, *J* = 272.1 Hz), 119.9, 117.2, 70.6, 14.5; ^{19}F NMR (376 MHz, $CDCl_3$) (δ, ppm) -62.66; HRMS (TOF MS ESI$^+$) calculated for $C_{22}H_{16}F_3O_3$ [M+H]$^+$: 385.1046, found 385.1043.

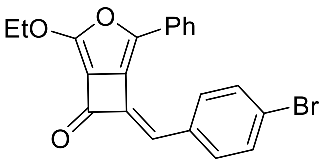

(*E*)-7-(4-Bromobenzylidene)-4-ethoxy-2-phenyl-3-oxabicyclo[3.2.0]hepta-1,4-dien -6-one (2x). Yellow solid, mp = 180 - 181 °C. 67.2 mg, 85% yield; ^{1}H NMR (400 MHz, $CDCl_3$) (δ, ppm) 7.35 - 7.28 (comp, 5H), 7.22 - 7.20 (m, 2H), 7.15 (d, *J* = 8.3 Hz, 2H), 6.97 (s, 1H), 4.44 (q, *J* = 7.1 Hz, 2H), 1.53 (t, *J* = 7.1 Hz, 3H); ^{13}C NMR (125 MHz, $CDCl_3$) (δ, ppm) 174.1, 149.8, 138.5, 136.0, 132.7, 131.8, 131.4, 129.3, 128.9, 128.2, 127.7, 123.4, 120.4, 116.9, 70.5, 14.5; HRMS (TOF MS ESI$^+$) calculated for $C_{21}H_{16}BrO_3$ [M+H]$^+$: 395.0277, found 395.0280.

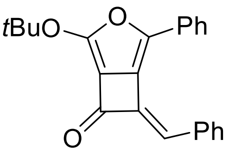

(*E*)-7-Benzylidene-4-(*tert*-butoxy)-2-phenyl-3-oxabicyclo[3.2.0]hepta-1,4-dien-6-one (2y). Yellow solid, mp = 150 - 151 °C. 54.4 mg, 79% yield; ^{1}H NMR (500 MHz, $CDCl_3$) (δ, ppm) 7.34 - 7.32 (comp, 3H), 7.26 - 7.23 (comp, 7H), 7.09 (s, 1H), 1.65 (s,

9H); ^{13}C NMR (125 MHz, $CDCl_3$) (δ, ppm) 175.6, 146.8, 137.9, 135.3, 134.0, 130.0, 129.9, 129.13, 129.05, 128.6, 128.1, 127.8, 127.7, 121.4, 117.2, 85.5, 27.5; HRMS (TOF MS ESI$^+$) calculated for $C_{23}H_{21}O_3$ [M+H]$^+$: 345.1485, found 345.1487.

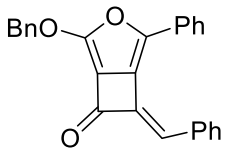

(*E*)-7-Benzylidene-4-(benzyloxy)-2-phenyl-3-oxabicyclo[3.2.0]hepta-1,4-dien-6-one (2z). Yellow oil. 65.8 mg, 87% yield; ^{1}H NMR (500 MHz, $CDCl_3$) (δ, ppm) 7.56 - 7.55 (m, 2H), 7.43 - 7.38 (comp, 3H), 7.34 - 7.30 (comp, 3H), 7.28 - 7.22 (comp, 7H), 7.09 (s, 1H), 5.37 (s, 2H); ^{13}C NMR (125 MHz, $CDCl_3$) (δ, ppm) 174.6, 149.3, 137.9, 136.3, 134.0, 133.8, 130.0, 129.7, 129.4, 129.3, 128.93, 128.90, 128.8, 128.6, 128.2, 127.9, 127.7, 122.1, 117.3, 75.8; HRMS (TOF MS ESI$^+$) calculated for $C_{26}H_{19}O_3$ [M+H]$^+$: 379.1329, found 379.1325.

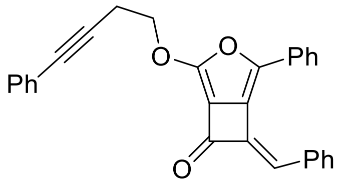

(*E*)-7-Benzylidene-2-phenyl-4-((4-phenylbut-3-yn-1-yl)oxy)-3-oxabicyclo[3.2.0] hepta-1,4-dien-6-one (2aa). Yellow solid, mp = 155 - 156 °C. 69.1 mg, 83% yield; ^{1}H NMR (400 MHz, $CDCl_3$) (δ, ppm) 7.43 - 7.41 (m, 2H), 7.37 - 7.30 (comp, 4H), 7.29 - 7.26 (comp, 7H), 7.25 - 7.23 (m, 2H), 7.10 (s, 1H), 4.56 (t, J = 6.6 Hz, 2H), 3.03 (t, J = 6.6 Hz, 2H); ^{13}C NMR (101 MHz, $CDCl_3$) (δ, ppm) 174.4, 149.0, 137.8, 136.3, 133.8, 131.9, 130.0, 129.6, 129.3, 128.9, 128.6, 128.4, 128.2, 128.0, 127.7, 123.2, 122.2, 117.2, 84.3, 82.9, 71.8, 20.4; HRMS (TOF MS ESI$^+$) calculated for $C_{29}H_{21}O_3$ [M+H]$^+$: 417.1485, found 417.1488.

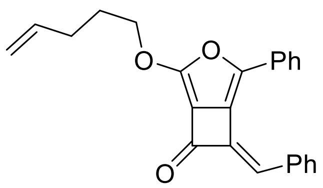

(*E*)-7-Benzylidene-4-(pent-4-en-1-yloxy)-2-phenyl-3-oxabicyclo[3.2.0]hepta-1,4-dien-6-one (2ab). Yellow solid, mp = 100 - 101 °C. 57.7 mg, 81% yield; ^{1}H NMR (400 MHz, $CDCl_3$) (δ, ppm) 7.28 - 7.24 (comp, 3H), 7.22 - 7.16 (comp, 7H), 7.01 (s, 1H), 5.82 - 5.72 (m, 1H), 5.05 - 4.96 (m, 2H), 4.33 (t, *J* = 6.3 Hz, 2H), 2.22 - 2.17 (m, 2H), 1.94 - 1.88 (m, 2H); ^{13}C NMR (126 MHz, $CDCl_3$) (δ, ppm) 174.6, 149.8, 137.9, 137.0, 136.0, 133.8, 130.0, 129.6, 129.3, 129.0, 128.6, 128.2, 127.9, 127.7, 121.9, 116.9, 116.0, 73.7, 29.8, 28.0; HRMS (TOF MS ESI$^+$) calculated for $C_{24}H_{21}O_3$ $[M+H]^+$: 357.1485, found 357.1486.

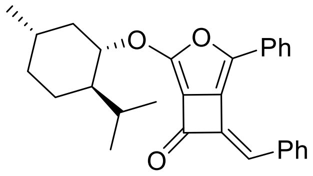

7-((*E*)-Benzylidene)-4-(((1*R*,2*S*,5*R*)-2-isopropyl-5-methylcyclohexyl)oxy)-2-phenyl-3-oxabicyclo[3.2.0]hepta-1,4-dien-6-one (2ac). Yellow oil. 64.0 mg, 75% yield; ^{1}H NMR (500 MHz, $CDCl_3$) (δ, ppm) 7.35 - 7.33 (comp, 3H), 7.28 - 7.23 (comp, 7H), 7.08 (s, 1H), 4.38 - 4.33 (m, 1H), 2.34 - 2.31 (m, 1H), 2.15 - 2.09 (m, 1H), 1.78 - 1.74 (m, 2H), 1.70 - 1.62 (m, 2H), 1.28 - 1.15 (comp, 3H), 0.98 (d, *J* = 6.6 Hz, 3H), 0.95 (d, *J* = 7.0 Hz, 3H), 0.84 (d, *J* = 6.9 Hz, 3H); ^{13}C NMR (125 MHz, $CDCl_3$) (δ, ppm) 174.4, 149.8, 138.2, 135.6, 134.0, 130.0, 129.6, 129.2, 129.1, 128.6, 128.2, 127.8, 127.6, 121.6, 116.3, 86.3, 47.5, 40.4, 34.1, 31.6, 26.2, 23.5, 22.2, 20.8, 16.5; HRMS (TOF MS ESI$^+$) calculated for $C_{29}H_{31}O_3$ $[M+H]^+$: 427.2268, found 427.2267.

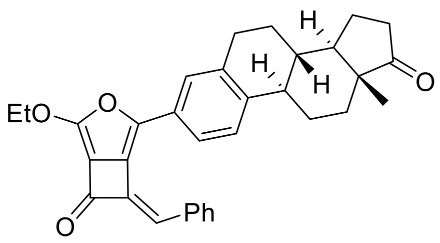

7-((*E*)-Benzylidene)-4-ethoxy-2-((8*S*,9*R*,13*R*,14*R*)-13-methyl-17-oxo-7,8,9,11,12,13,14,15,16,17-decahydro-6*H*-cyclopenta[*a*]phenanthren-3-yl)-3-oxabicyclo[3.2.0]hepta-1,4-dien-6-one (2ad). Yellow oil, 78.8 mg, 80% yield; ^{1}H NMR (500 MHz, $CDCl_3$) (δ, ppm) 7.36 - 7.34 (comp, 3H), 7.28 - 7.26 (m, 2H), 7.21 (d, *J* = 8.1 Hz, 1H), 7.10 (d, *J* = 8.1 Hz, 1H), 7.07 (s, 1H), 6.92 (s, 1H), 4.44 (q, *J* = 7.1 Hz, 2H), 2.72 - 2.63 (m, 2H), 2.54 - 2.49 (m, 1H), 2.44 - 2.41 (m, 1H), 2.34 - 2.30 (t, *J* = 9.1 Hz, 1H), 2.14 (dd, *J* = 18.7, 9.1 Hz, 1H), 2.08 - 2.04 (m, 1H), 2.00 - 1.98 (m, 2H), 1.64 - 1.60 (m, 2H), 1.55 - 1.52 (comp, 6H), 1.44 - 1.41 (m, 1H), 0.94 (s, 3H); ^{13}C NMR (125 MHz, $CDCl_3$) (δ, ppm) 220.8, 174.6, 149.5, 139.7, 138.0, 136.4, 136.1, 133.9, 130.0, 129.1, 128.9, 128.6, 128.3, 126.6, 125.2, 124.8, 121.6, 116.7, 70.3, 50.6, 48.1, 44.6, 38.2, 35.9, 31.7, 29.2, 26.5, 25.8, 21.7, 14.5, 14.0; HRMS (TOF MS ESI$^+$) calculated for $C_{33}H_{32}O_4Na$ $[M+Na]^+$: 515.2193, found 515.2192.

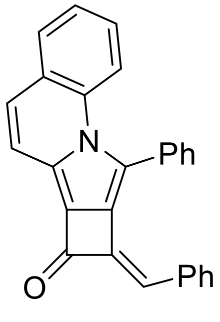

(*E*)-9-Benzylidene-8-phenylcyclobuta[3,4]pyrrolo[2,1-a]isoquinolin-10(9*H*)-one (2ae). Yellow oil, 31.2 mg, 42% yield; ^{1}H NMR (500 MHz, DMSO) (δ, ppm) 7.99 (d, *J* = 7.7 Hz, 1H), 7.76 - 7.71 (comp, 3H), 7.65 - 7.64 (comp, 4H), 7.48 - 7.45 (m, 1H), 7.33 - 7.31 (m, 2H), 7.13 (t, *J* = 7.4 Hz, 1H), 7.09 (d, *J* = 7.5 Hz, 2H), 6.88 (t, *J* = 7.6 Hz, 2H), 6.73 (s, 1H); ^{13}C NMR (126 MHz, DMSO) (δ, ppm) 176.3, 142.2, 141.2, 136.6, 134.4, 133.6, 133.4, 131.7, 129.9, 129.7, 129.2, 128.7, 128.11, 128.08, 126.5,

125.8, 125.5, 123.6, 121.7, 117.5, 117.0, 115.8; HRMS (TOF MS ESI$^+$) calculated for $C_{27}H_{18}NO$ [M+H]$^+$: 372.1383, found 372.1389.

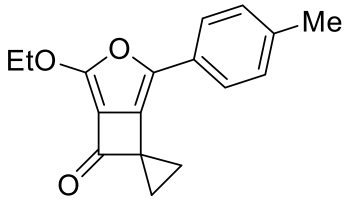

2-Ethoxy-4-(*p*-tolyl)-3-oxaspiro[bicyclo[3.2.0]heptane-6,1'-cyclopropane]-1,4-dien-7-one (2af). White solid, 48.8 mg, 91% yield; ^{1}H NMR (400 MHz, $CDCl_3$) (δ, ppm) 7.08 (dd, *J* = 19.7, 8.1 Hz, 4H), 4.35 (q, *J* = 7.1 Hz, 2H), 2.31 (s, 3H), 1.51 - 1.42 (comp, 7H); ^{13}C NMR (126 MHz, $CDCl_3$) (δ, ppm) 183.3, 148.7, 136.2, 133.8, 129.5, 128.1, 127.0, 122.6, 111.1, 70.0, 40.6, 21.2, 14.4, 12.5; HRMS (TOF MS ESI$^+$) calculated for $C_{17}H_{16}O_3Na$ [M+Na]$^+$: 291.0992, found 291.0995.

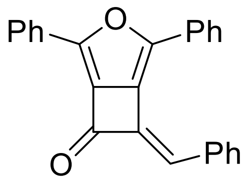

(*E*)-7-Benzylidene-2,4-diphenyl-3-oxabicyclo[3.2.0]hepta-1,4-dien-6-one (2ag). Yellow solid, m.p.: 115 - 116 °C; 50.1 mg, 72% yield; ^{1}H NMR (500 MHz, DMSO) (δ, ppm) 8.07 (d, *J* = 7.5 Hz, 2H), 7.82 - 7.79 (comp, 4H), 7.57 - 7.53 (comp, 4H), 7.48 - 7.45 (comp, 3H), 7.43 - 7.39 (m, 2H), 7.08 (s, 1H); ^{13}C NMR (126 MHz, DMSO) (δ, ppm) 173.9, 143.5, 142.2, 139.6, 138.1, 134.5, 133.9, 130.2, 129.8, 129.5, 129.4, 129.3, 129.2, 128.63, 128.55, 128.5, 127.1, 125.6, 124.6; HRMS (TOF MS ESI$^+$) calculated for $C_{25}H_{16}O_2Na$ [M+Na]$^+$: 371.1043, found 371.1043.

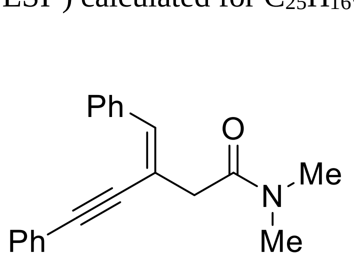

(*Z*)-3-Benzylidene-*N*,*N*-dimethyl-5-phenylpent-4-ynamide (2ah). Colorless oil; 19.1 mg, 33% yield; ^{1}H NMR (400 MHz, $CDCl_3$) (δ, ppm) 7.88 (d, *J* = 7.4 Hz, 2H),

7.47 - 7.46 (m, 2H), 7.38 - 7.35 (comp, 5H), 7.29 (d, *J* = 7.1 Hz, 1H), 6.72 (s, 1H), 3.50 (s, 2H), 3.15 (s, 3H), 3.02 (s, 3H); ^{13}C NMR (101 MHz, $CDCl_3$) (δ, ppm) 170.2, 136.9, 136.9, 136.4, 131.7, 128.8, 128.7, 128.6, 128.3, 123.3, 115.2, 96.1, 89.2, 43.6, 38.2, 35.9; HRMS (TOF MS ESI$^+$) calculated for $C_{20}H_{19}NONa$ [M+Na]$^+$: 312.1359, found 312.1363.

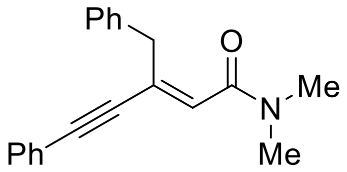

(*E*)-3-Benzyl-*N,N*-dimethyl-5-phenylpent-2-en-4-ynamide (2ah'). Colorless oil; 23.6 mg, 41% yield; ^{1}H NMR (400 MHz, $CDCl_3$) (δ, ppm) 7.39 (d, *J* = 7.3 Hz, 2H), 7.32 - 7.20 (commp, 8H), 6.47 (s, 1H), 3.91 (s, 2H), 3.04 (d, *J* = 7.8 Hz, 6H); ^{13}C NMR (101 MHz, $CDCl_3$) (δ, ppm) 166.8, 138.8, 133.4, 131.8, 129.5, 128.8, 128.43, 128.39, 126.91, 126.88, 126.5, 122.8, 93.0, 90.2, 38.7, 37.9, 35.1; HRMS (TOF MS ESI$^+$) calculated for $C_{20}H_{19}NONa$ [M+Na]$^+$: 312.1359, found 312.1357.

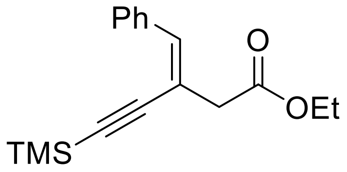

Ethyl (*Z*)-3-benzylidene-5-(trimethylsilyl)pent-4-ynoate (2ai). Colorless oil; 19.4 mg, 34% yield; ^{1}H NMR (500 MHz, $CDCl_3$) (δ, ppm) 7.87 (d, *J* = 7.7 Hz, 2H), 7.34 - 7.31 (m, 2H), 7.29 - 7.25 (m, 1H), 6.65 (s, 1H), 4.19 (q, *J* = 7.1 Hz, 2H), 3.29 (s, 2H), 1.28 (t, *J* = 7.1 Hz, 3H), 0.23 (s, 9H); ^{13}C NMR (126 MHz, $CDCl_3$) (δ, ppm) 170.6, 138.6, 136.0, 128.9, 128.6, 128.2, 114.4, 104.1, 102.4, 61.1, 44.4, 14.4, -0.2; HRMS (TOF MS ESI$^+$) calculated for $C_{17}H_{22}O_2SiNa$ [M+Na]$^+$: 309.1281, found 309.1286.

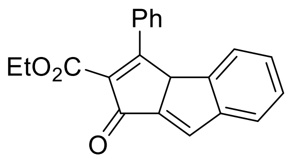

Ethyl 1-oxo-3-phenyl-1,3a-dihydrocyclopenta[*a*]indene-2-carboxylate (3a). White oil; ^{1}H NMR (500 MHz, $CDCl_3$) (δ, ppm) 8.06 - 8.04 (m, 1H), 8.00 (s, 1H), 7.96 (d, J = 8.3 Hz, 1H), 7.62 - 7.55 (m, 2H), 7.53 - 7.47 (comp, 4H), 7.26 (s, 1H), 5.16 (s, 1H), 4.15 - 4.03 (m, 2H), 1.12 (t, J = 7.1 Hz, 3H); ^{13}C NMR (125 MHz, $CDCl_3$) (δ, ppm) 182.9, 167.6, 145.8, 140.6, 136.8, 135.9, 135.6, 135.3, 131.4, 129.9, 129.4, 128.8, 128.3, 126.74, 126.68, 121.2, 69.3, 61.6, 14.2; HRMS (TOF MS ESI^+) calculated for $C_{21}H_{16}O_3Na$ $[M+Na]^+$: 339.0992, found 339.0996.

General procedure of the gram scale reaction

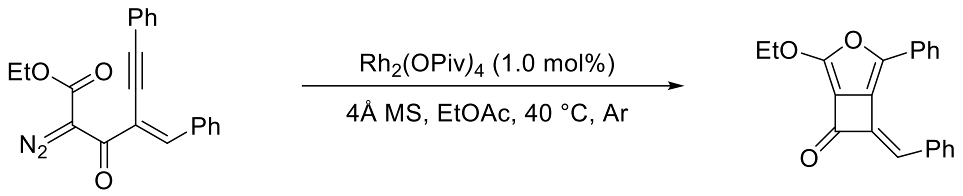

1a, 5.0 mmol, 1.72 g

2a, 80% yield, 1.26 g

To a 50-mL oven-dried vial containing a magnetic stirring bar, $Rh_2(OPiv)_4$ (7.6 mg, 0.25 mol%), and 4Å MS (2.0 g) in EtOAc (10 mL), was added as a solution of diazo compound **1a** (1.72 g, 5.0 mmol) in EtOAc (5.0 mL) *via* a syringe under argon atmosphere at 40 ˚C. After addition, the reaction mixture was stirred for additional 2.0 h. Then most of the solvent was evaporated in vacuo, the residue was recrystallized from MeOH to give 1.27 g of pure **2a** in 80% yield.

General procedure for ring opening transformations of 2

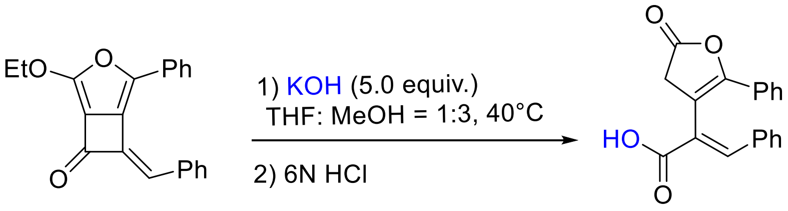

2a

4, 81% yield

<u>Synthesis of 4:</u> To a 10-mL oven-dried vial containing a magnetic stirring bar, and **2a** (63.2 mg, 0.2 mmol) in THF/MeOH = 1:3 (2.0 mL), was added KOH (56.0 mg, 1.0 mmol, 5.0 equiv.) at room temperature. Then the reaction mixture was stirred for additional 12 h under these conditions until consumption of the material (monitored by TLC). The reaction mixture was acidified with 2.0 mL of 6 N HCl, and extracted with Et_2O (3 × 10 mL). The combined extract was washed with brine (10 mL), dried with Na_2SO_4, and then concentrated under reduced pressure after filtration. The crude product was purified by flash chromatography on silica gel (Hexanes : EtOAc = 1:1 to 1:2) to give 49.6 mg pure product **4** in 81% yield. Colorless oil. ^{1}H NMR (400 MHz, $CDCl_3$) (δ, ppm) 7.96 (s, 1H), 7.56 - 7.54 (m, 2H), 7.46 (d, J = 7.5 Hz, 2H), 7.35 - 7.30 (comp, 6H), 3.53 (s, 2H); ^{13}C NMR (100 MHz, $CDCl_3$) (δ, ppm) 174.5, 171.3,

150.3, 145.8, 133.9, 130.8, 129.9, 129.8, 129.2, 128.8, 128.7, 126.4, 122.9, 107.3, 37.6; HRMS (TOF MS ESI$^+$) calculated for $C_{19}H_{14}O_4Na$ [M+Na]$^+$: 329.0784, found 329.0782.

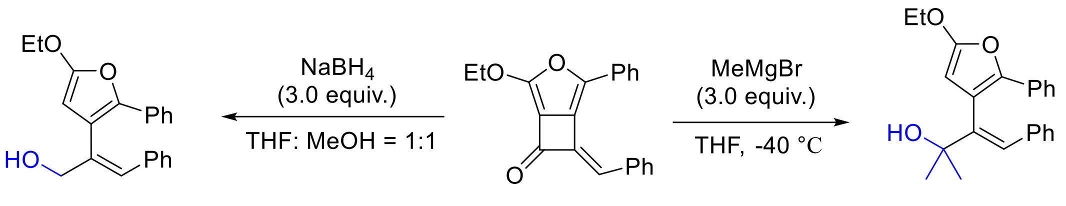

5a, 94% yield **2a** **5b**, 90% yield

Synthesis of 5a: To a 10-mL oven-dried vial containing a magnetic stirring bar, and **2a** (63.2 mg, 0.2 mmol) in THF/MeOH = 1 : 1 (2.0 mL), was added $NaBH_4$ (22.7 mg, 0.6 mmol, 3.0 equiv.) at 0 °C under stirring. The reaction mixture was stirred for additional 30 minutes under these conditions until consumption of the material (monitored by TLC). The reaction mixture was quenched with saturated aqueous NH_4Cl solution (10 mL), and extracted with Et_2O (2 × 10 mL). The combined extract was washed with brine (10 mL), dried with anhydrous Na_2SO_4, and then concentrated under reduced pressure after filtration. The crude product was purified by flash chromatography on silica gel (Hexanes : EtOAc = 10:1 to 5:1) to give 60.2 mg pure product **5a** in 94% yield. Colorless oil. ^{1}H NMR (400 MHz, $CDCl_3$) (δ, ppm) 7.63 - 7.61 (m, 2H), 7.37 - 7.35 (m, 2H), 7.31 - 7.27 (m, 2H), 7.24 - 7.20 (m, 2H), 7.18 - 7.15 (m, 2H), 6.84 (s, 1H), 5.10 (s, 1H), 4.37 (d, *J* = 1.3 Hz, 2H), 4.12 (q, *J* = 7.1 Hz, 2H), 1.95 (bs, 1H), 1.45 (t, *J* = 7.1 Hz, 3H); ^{13}C NMR (125 MHz, $CDCl_3$) (δ, ppm) 160.3, 138.8, 136.3, 134.2, 131.0, 128.8, 128.6, 128.3, 128.2, 127.3, 126.4, 124.0, 119.4, 84.5, 67.1, 66.9, 14.6; HRMS (TOF MS ESI$^+$) calculated for $C_{21}H_{20}O_3Na$ [M+Na]$^+$: 343.1305, found 343.1303.

Synthesis of 5b: To a 10-mL oven-dried vial containing a magnetic stirring bar, and **2a** (63.2 mg, 0.2 mmol) in anhydrous THF (2.0 mL), was added methyl magnesium bromide (3.0 M in 2-methyl tetrahydrofuran, 0.2 mL, 0.6 mmol, 3.0 equiv.) drop wise under argon atmosphere at -40 °C under stirring. After addition, the reaction mixture was stirred under these conditions for 2.0 h until consumption of the material

(monitored by TLC). The reaction mixture was quenched with saturated aqueous NH_4Cl solution (10 mL), and extracted with Et_2O (3 × 10 mL). The combined organic extract was washed with brine, dried with anhydrous Na_2SO_4, and then concentrated under reduced pressure after filtration. The crude product was purified by flash chromatography on silica gel (Hexanes : EtOAc = 10:1 to 4:1) to afford 62.7 mg pure product **5b** in 90% yield. Colorless oil. ^1H NMR (400 MHz, $CDCl_3$) (δ, ppm) 7.57 - 7.55 (m, 2H), 7.27 - 7.25 (m, 2H), 7.21 - 7.18 (m, 2H), 7.15 - 7.08 (comp, 4H), 6.98 (s, 1H), 5.14 (s, 1H), 4.15 (q, *J* = 7.1 Hz, 2H), 1.80 (bs, 1H), 1.47 - 1.43 (comp, 6H), 1.36 (s, 3H); ^{13}C NMR (100 MHz, $CDCl_3$) (δ, ppm) 160.1, 141.2, 139.0, 136.7, 131.4, 128.9, 128.4, 128.3, 127.7, 127.3, 126.4, 124.4, 119.1, 86.0, 74.7, 67.0, 30.2, 29.4, 14.7; HRMS (TOF MS ESI$^+$) calculated for $C_{23}H_{24}O_3Na$ [M+Na]$^+$: 371.1618, found 371.1620.

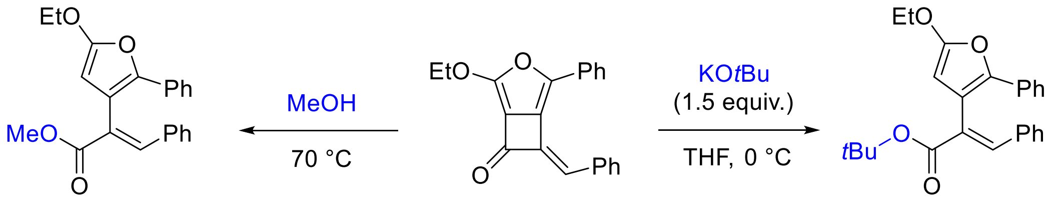

6a, 82% yield **2a** **6b**, 84% yield

Synthesis of 6a: A solution of **2a** (63.2 mg, 0.2 mmol) in MeOH (2.0 mL) was stirred at 70 ˚C for 2.0 h. Upon completion (monitored by TLC), the mixture was cooled to room temperature and the solvent was removed under reduced pressure. The crude product was purified by flash chromatography on silica gel (Hexanes : EtOAc = 20:1 to 5:1) to give 57.1 mg pure product **6a** in 82% yield. Yellow oil. ^1H NMR (400 MHz, $CDCl_3$) (δ, ppm) 7.89 (s, 1H), 7.47 (d, *J* = 8.0 Hz, 2H), 7.42 - 7.40 (m, 2H), 7.25 - 7.22 (comp, 5H), 7.11 (t, *J* = 7.4 Hz, 1H), 5.06 (s, 1H), 4.11 (q, *J* = 7.0 Hz, 2H), 3.68 (s, 3H), 1.43 (t, *J* = 7.1 Hz, 3H); ^{13}C NMR (100 MHz, $CDCl_3$) (δ, ppm) 168.1, 160.1, 142.6, 140.1, 134.6, 131.2, 130.6, 129.7, 128.5, 126.5, 125.0, 124.1, 116.6, 84.7, 67.0, 52.6, 14.7. HRMS (TOF MS ESI$^+$) calculated for $C_{22}H_{20}O_4Na$ [M+Na]$^+$: 371.1254, found 371.1255.

Synthesis of 6b: To a 10-mL oven-dried vial containing a magnetic stirring bar, and

*t*BuOK (33.7 mg, 0.3 mmol, 1.5 equiv.) in THF (1.0 mL), was added as a solution of **2a** (63.2 mg, 0.2 mmol) in THF (1.0 mL) *via* a syringe under argon atmosphere at 0 ˚C. The reaction mixture was stirred for additional 1.0 h under these conditions until consumption of the material (monitored by TLC). The reaction mixture was quenched with saturated aqueous NH_4Cl solution (10 mL), and extracted with Et_2O (3 × 10 mL). The combined organic extract was washed with brine (10 mL), dried with anhydrous Na_2SO_4, and then concentrated under reduced pressure after filtration. The crude product was purified by flash chromatography on silica gel (Hexanes : EtOAc = 20:1 to 5:1) to give 65.6 mg pure product **6b** in 84% yield. Yellow oil. ^{1}H NMR (400 MHz, $CDCl_3$) (δ, ppm) 7.78 (s, 1H), 7.50 (d, *J* = 8.0 Hz, 2H), 7.43 - 7.41 (m, 2H), 7.28 - 7.22 (comp, 5H), 7.12 (t, *J* = 7.4 Hz, 1H), 5.00 (s, 1H), 4.08 (q, *J* = 7.0 Hz, 2H), 1.41 (t, *J* = 7.0 Hz, 3H), 1.28 (s, 9H); ^{13}C NMR (100 MHz, $CDCl_3$) (δ, ppm) 166.4, 159.8, 141.0, 140.5, 134.9, 131.6, 130.5, 129.3, 128.5, 128.4, 127.1, 126.4, 124.4, 117.2, 85.0, 81.1, 67.0, 27.9, 14.6; HRMS (TOF MS ESI$^+$) calculated for $C_{25}H_{26}O_4Na$ $[M+Na]^+$: 413.1723, found 413.1724.

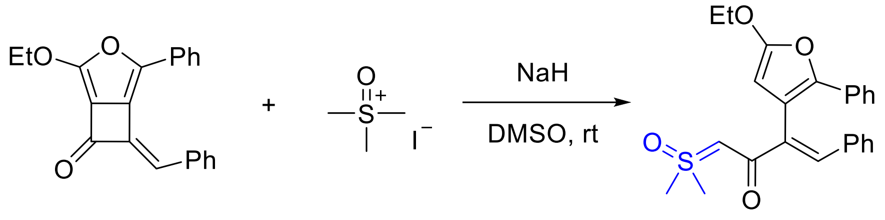

2a (1.5 equiv.) **7**, 50% yield

Synthesis of 7: To a stirred solution of trimethylsulfoxonium iodide (33.1 mg, 0.15 mmol, 1.5 equiv.) in anhydrous DMSO (1.0 mL) was added NaH (60% in mineral oil, 6.0 mg, 0.15 mmol) at room temperature. After addition, the solution was allowed to stir at the same temperature for 30 min. Then, the solution of furan-fused cyclobutanone **2a** (31.6 mg, 0.1 mmol) in DMSO (1.0 mL) was added. After addition, the reaction mixture was stirred for additional 3.0 h under these conditions. Until consumption of the material (monitored by TLC), the reaction was quenched with 5.0 mL water and extracted with ethyl acetate, and dried over anhydrous Na_2SO_4. The crude material was purified by column chromatography on silica gel (eluent:

petroleum ether / ethyl acetate = 2:1) to give 20.4 mg pure product **7** in 50 % yield; Colorless oil; ^{1}H NMR (500 MHz, $CDCl_3$) (δ, ppm) 7.70 (s, 1H), 7.55 (d, *J* = 7.6 Hz, 2H), 7.40 - 7.39 (m, 2H), 7.26 - 7.20 (comp, 5H), 7.10 (t, *J* = 7.4 Hz, 1H), 5.07 (s, 1H), 4.55 (s, 1H), 4.12 (q, *J* = 7.0 Hz, 2H), 3.31 (s, 6H), 1.44 (t, *J* = 7.0 Hz, 3H); ^{13}C NMR (126 MHz, $CDCl_3$) (δ, ppm) 180.7, 160.2, 139.8, 135.7, 134.8, 132.5, 131.3, 130.4, 128.6, 128.5, 128.4, 126.3, 124.1, 119.3, 85.1, 71.9, 67.0, 42.0, 14.7; HRMS (TOF MS ESI$^+$) calculated for $C_{24}H_{24}O_4SNa$ $[M+Na]^+$: 431.1288, found 431.1285.

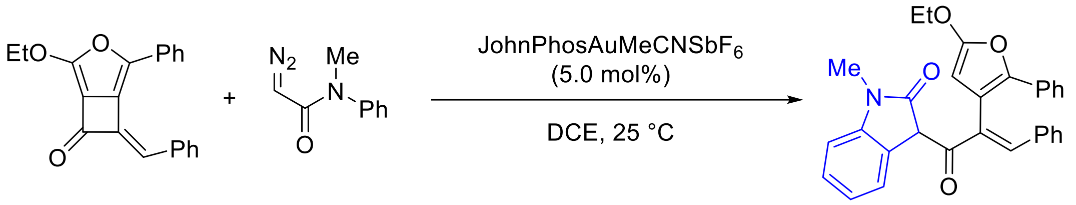

2a (1.5 equiv.) **8**, 56% yield

<u>Synthesis of 8</u>: To a 10-mL oven-dried vial containing a magnetic stirring bar, furan-fused cyclobutanone **2a** (31.6 mg, 0.1 mmol), and diazo compound (26.3 mg, 0.15 mmol, 1.5 equiv.) in DCE (1.0 mL), was added as a solution of JohnPhosAu(MeCN)SbF_6 (3.9 mg, 5.0 mol%) in the DCE (1.0 mL) slowly *via* a syringe at 25 °C under argon atmosphere. After addition, the reaction mixture was stirred for additional 2.0 h under these conditions. Until consumption of the material (monitored by TLC), the reaction mixture was purified by column chromatography on silica gel without any additional treatment (Hexanes : EtOAc = 10:1 to 3:1) to give the 25.9 mg pure product **8** in 56% yield; Colorless oil; ^{1}H NMR (500 MHz, $CDCl_3$) (δ, ppm) 8.13 (s, 1H), 7.58 (d, *J* = 8.2 Hz, 2H), 7.55 - 7.50 (comp, 3H), 7.35 - 7.26 (comp, 5H), 7.17 - 7.13 (comp, 3H), 7.08 - 7.05 (m, 1H), 6.27 (s, 1H), 5.17 (s, 1H), 4.15 (q, *J* = 7.0 Hz, 2H), 3.21 (s, 3H), 1.46 (t, *J* = 7.0 Hz, 3H); ^{13}C NMR (126 MHz, $CDCl_3$) (δ, ppm) 163.9, 160.4, 145.5, 143.1, 140.4, 134.1, 132.5, 131.1, 131.0, 130.5, 128.9, 128.8, 126.9, 126.3, 124.2, 123.5, 121.2, 120.6, 120.0, 116.1, 108.8, 87.6, 84.6, 67.2, 28.0, 14.7; HRMS (TOF MS ESI$^+$) calculated for $C_{30}H_{25}NO_4Na$ $[M+Na]^+$: 486.1676, found 486.1678.

General procedure for the synthesis of amide derivatives 9 - 49 and 53 - 58

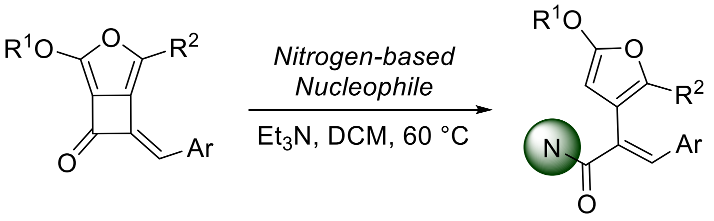

To a 10-mL oven-dried vial containing a magnetic stirring bar, cyclobutanones **2** (0.1 mmol), amine or hydrochloride of amino acid ester (0.15 mmol, 1.5 equiv.), Et_3N (0.2 mmol, 2.0 equiv.), and DCM (2.0 mL) were added sequentially. The vial was capped, and stirring for 48 h in an oil bath at 60 °C. After completion of the reaction (monitored by TLC), the solvent was evaporated under reduced pressure, and the residue was purified by flash chromatography on silica gel (Hexanes : EtOAc = 10:1 to 1:1) to afford a ring opening amide products in good to excellent yields.

General procedure for the synthesis of esters 50-52

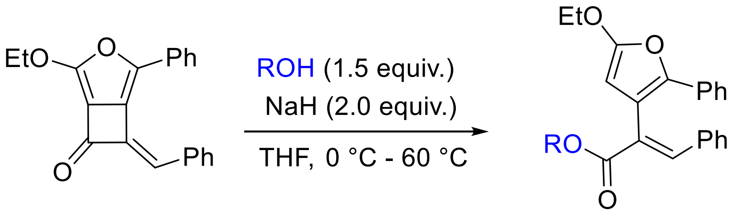

To a 10-mL oven-dried vial containing a magnetic stirring bar, and sodium hydride (60% dispersion in mineral oil, 0.2 mmol, 2.0 equiv.) in dry THF (2.0 mL), was added alcohol (0.15 mmol, 1.5 equiv.) dropwise under stirring at 0 ºC under a nitrogen atmosphere. After the reaction mixture turned to clear, a solution of cyclobutanone **2** (0.1 mmol) in THF (1.0 mL) was added dropwise at room temperature, then the reaction was stirred at 60 ºC for 0.5 h. Saturated aqueous NH_4Cl (10 mL) was added to quench the reaction, the organic phase was separated, and the aqueous layer was extracted with EtOAc (3 × 5.0 mL). The combined organic layer was washed with brine, dried over anhydrous $MgSO_4$, and concentrated in vacuo after filtration. The residue was purified by chromatography on silica gel (Hexanes : EtOAc = 30:1) to afford the ester products in high to excellent yields.

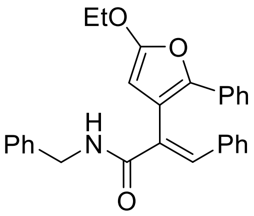

(*E*)-*N*-Benzyl-2-(5-ethoxy-2-phenylfuran-3-yl)-3-phenylacrylamide (9). Yellow oil. 30.1 mg, 71% yield; ^{1}H NMR (500 MHz, $CDCl_3$) (δ, ppm) 8.05 (s, 1H), 7.56 (d, J = 7.7 Hz, 2H), 7.40 (s, 2H), 7.29 (t, J = 7.5 Hz, 2H), 7.24 (s, 3H), 7.21 - 7.18 (m, 1H), 7.16 - 7.12 (comp, 3H), 6.90 (d, J = 7.0 Hz, 2H), 6.36 (bs, 1H), 5.05 (s, 1H), 4.43 (d, J = 5.3 Hz, 2H), 4.09 (q, J = 6.9 Hz, 2H), 1.41 (t, J = 7.0 Hz, 3H); ^{13}C NMR (125 MHz, $CDCl_3$) (δ, ppm) 165.8, 160.7, 140.1, 139.8, 138.1, 134.9, 130.4, 130.2, 129.3, 128.9, 128.6, 128.5, 127.3, 127.2, 127.1, 126.3, 124.1, 116.7, 84.5, 67.2, 44.0, 14.6; HRMS (TOF MS ESI$^+$) calculated for $C_{28}H_{25}NO_3Na$ $[M+Na]^+$: 446.1727, found 446.1725.

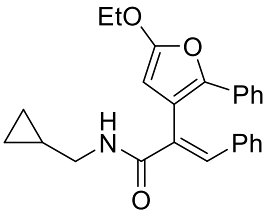

(*E*)-*N*-(Cyclopropylmethyl)-2-(5-ethoxy-2-phenylfuran-3-yl)-3-phenylacrylamide (10). Yellow oil. 36.0 mg, 93% yield; ^{1}H NMR (500 MHz, $CDCl_3$) (δ, ppm) 7.98 (s, 1H), 7.57 - 7.55 (m, 2H), 7.39 - 7.37 (m, 2H), 7.29 - 7.26 (m, 2H), 7.23 - 7.22 (comp, 3H), 7.17 - 7.14 (m, 1H), 6.16 (t, J = 5.1 Hz, 1H), 5.06 (s, 1H), 4.13 (q, J = 7.0 Hz, 2H), 3.10 - 3.07 (m, 2H), 1.45 (t, J = 7.0 Hz, 3H), 0.72 - 0.66 (m, 1H), 0.28 - 0.24 (m, 2H), -0.03 - -0.06 (m, 2H); ^{13}C NMR (125 MHz, $CDCl_3$) (δ, ppm) 165.6, 160.7, 140.1, 139.2, 135.1, 130.4, 130.3, 129.1, 128.8, 128.5, 127.1, 126.6, 124.1, 117.0, 84.7, 67.2, 44.8, 14.6, 10.6, 3.2; HRMS (TOF MS ESI$^+$) calculated for $C_{25}H_{25}NO_3Na$ $[M+Na]^+$: 410.1727, found 410.1728.

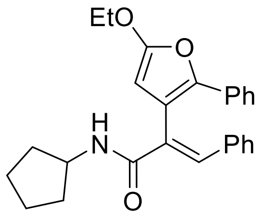

(*E*)-*N*-Cyclopentyl-2-(5-ethoxy-2-phenylfuran-3-yl)-3-phenylacrylamide (11). Yellow oil. 32.2 mg, 80% yield; ^{1}H NMR (500 MHz, $CDCl_3$) (δ, ppm) 7.97 (s, 1H), 7.54 (d, *J* = 7.9 Hz, 2H), 7.38 - 7.37 (m, 2H), 7.30 - 7.26 (m, 2H), 7.23 - 7.22 (comp, 3H), 7.17 (t, *J* = 7.4 Hz, 1H), 5.97 (d, *J* = 7.4 Hz, 1H), 5.02 (s, 1H), 4.23 - 4.16 (m, 1H), 4.11 (q, *J* = 7.0 Hz, 2H), 1.79 - 1.76 (m, 2H), 1.44 (t, *J* = 7.0 Hz, 5H), 1.36 - 1.34 (m, 2H), 1.09 - 1.07 (m, 2H); ^{13}C NMR (125 MHz, $CDCl_3$) (δ, ppm) 165.0, 160.6, 140.1, 139.0, 135.1, 130.3, 130.2, 129.0, 128.8, 128.4, 127.1, 126.7, 124.1, 117.1, 84.6, 67.2, 51.7, 32.9, 23.6, 14.6; HRMS (TOF MS ESI$^+$) calculated for $C_{26}H_{27}NO_3Na$ [M+Na]$^+$: 424.1883, found 424.1881.

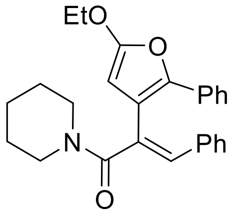

(*E*)-2-(5-Ethoxy-2-phenylfuran-3-yl)-3-phenyl-1-(piperidin-1-yl)prop-2-en-1-one (12). Yellow oil. 37.0 mg, 92% yield; ^{1}H NMR (400 MHz, $CDCl_3$) (δ, ppm) 7.56 - 7.54 (m, 2H), 7.37 - 7.34 (m, 2H), 7.28 - 7.24 (m, 2H), 7.22 - 7.17 (comp, 3H), 7.16 - 7.12 (m, 1H), 6.99 (s, 1H), 5.07 (s, 1H), 4.05 (q, *J* = 7.1 Hz, 2H), 3.31 (s, 4H), 1.50 - 1.44 (m, 2H), 1.39 (t, *J* = 7.1 Hz, 3H), 1.27 - 1.20 (comp, 4H); ^{13}C NMR (100 MHz, $CDCl_3$) (δ, ppm) 169.5, 159.9, 140.6, 135.7, 135.6, 130.6, 130.3, 129.5, 128.3, 128.20, 128.15, 126.9, 125.4, 117.6, 84.3, 67.0, 48.2, 43.6, 25.5, 24.5, 14.5; HRMS (TOF MS ESI$^+$) calculated for $C_{26}H_{28}NO_3$ [M+H]$^+$: 402.2064, found 402.2061.

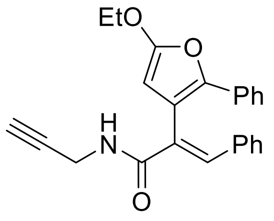

(E)-2-(5-Ethoxy-2-phenylfuran-3-yl)-3-phenyl-N-(prop-2-yn-1-yl)acrylamide (13). Yellow oil; 32.7 mg, 88% yield; ^{1}H NMR (500 MHz, $CDCl_3$) (δ, ppm) 8.00 (s, 1H), 7.54 (d, J = 7.5 Hz, 2H), 7.38 - 7.36 (m, 2H), 7.28 - 7.22 (comp, 5H), 7.15 (t, J = 7.4 Hz, 1H), 6.24 (t, J = 5.1 Hz, 1H), 5.08 (s, 1H), 4.13 (q, J = 7.0 Hz, 2H), 4.03 (dd, J = 5.4, 2.4 Hz, 2H), 2.09 (t, J = 2.5 Hz, 1H), 1.45 (t, J = 7.0 Hz, 3H); ^{13}C NMR (125 MHz, $CDCl_3$) (δ, ppm) 165.6, 160.7, 140.11, 140.06, 134.7, 130.4, 130.1, 129.4, 128.8, 128.5, 127.1, 125.7, 124.0, 116.2, 84.4, 79.4, 71.4, 67.2, 29.8, 14.6; HRMS (TOF MS ESI$^+$) calculated for $C_{24}H_{21}NO_3Na$ $[M+Na]^+$: 394.1414, found 394.1416.

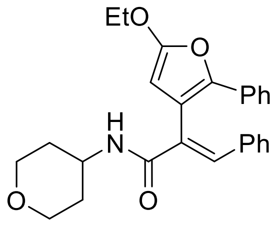

(E)-2-(5-Ethoxy-2-phenylfuran-3-yl)-3-phenyl-N-(tetrahydro-2H-pyran-4-yl) acrylamide (14). Yellow oil. 36.4 mg, 87% yield; ^{1}H NMR (500 MHz, $CDCl_3$) (δ, ppm) 7.96 (s, 1H), 7.54 (d, J = 7.6 Hz, 2H), 7.40 - 7.38 (m, 2H), 7.30 - 7.27 (m, 2H), 7.24 - 7.23 (comp, 3H), 7.17 (t, J = 7.4 Hz, 1H), 5.94 (d, J = 7.9 Hz, 1H), 5.02 (s, 1H), 4.12 (q, J = 7.0 Hz, 2H), 4.02 - 3.95 (m, 1H), 3.74 - 3.72 (m, 2H), 3.40 - 3.35 (m, 2H), 1.62 - 1.60 (m, 2H), 1.44 (t, J = 7.0 Hz, 3H), 1.15 - 1.11 (m, 2H); ^{13}C NMR (125 MHz, $CDCl_3$) (δ, ppm) 164.8, 160.7, 140.2, 139.5, 135.0, 130.4, 130.2, 129.2, 128.9, 128.5, 127.2, 126.5, 124.2, 116.9, 84.6, 67.2, 66.6, 46.0, 32.6, 14.6; HRMS (TOF MS ESI$^+$) calculated for $C_{26}H_{27}NO_4Na$ $[M+Na]^+$: 440.1832, found 440.1831.

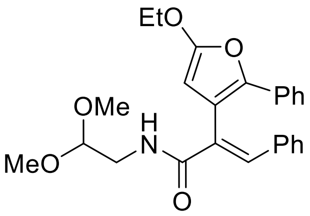

(*E*)-*N*-(2,2-Dimethoxyethyl)-2-(5-ethoxy-2-phenylfuran-3-yl)-3-phenylacrylamide (15). Yellow oil. 40.9 mg, 97% yield; ^{1}H NMR (500 MHz, $CDCl_3$) (δ, ppm) 7.99 (s, 1H), 7.56 (d, *J* = 7.7 Hz, 2H), 7.40 - 7.38 (m, 2H), 7.29 - 7.26 (m, 2H), 7.23 - 7.22 (comp, 3H), 7.14 (t, *J* = 7.4 Hz, 1H), 6.27 (t, *J* = 5.8 Hz, 1H), 5.05 (s, 1H), 4.12 (q, *J* = 7.0 Hz, 2H), 4.08 (t, *J* = 5.6 Hz, 1H), 3.38 - 3.36 (m, 2H), 3.14 (s, 6H), 1.45 (t, *J* = 7.0 Hz, 3H); ^{13}C NMR (125 MHz, $CDCl_3$) (δ, ppm) 165.8, 160.7, 140.0, 139.5, 134.9, 130.4, 130.3, 129.2, 128.8, 128.5, 126.9, 126.4, 124.0, 116.7, 102.9, 84.5, 67.2, 54.6, 41.8, 14.6; HRMS (TOF MS ESI$^+$) calculated for $C_{25}H_{27}NO_5Na$ $[M+Na]^+$: 444.1781, found 444.1779.

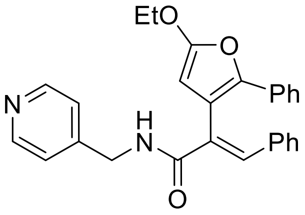

(*E*)-2-(5-Ethoxy-2-phenylfuran-3-yl)-3-phenyl-*N*-(pyridin-4-ylmethyl)acrylamide (16). Yellow oil. 30.6 mg, 72% yield; ^{1}H NMR (500 MHz, $CDCl_3$) (δ, ppm) 8.32 (d, *J* = 3.8 Hz, 2H), 8.04 (s, 1H), 7.57 - 7.56 (m, 2H), 7.44 - 7.42 (m, 2H), 7.32 - 7.29 (m, 2H), 7.27 - 7.23 (comp, 4H), 6.80 (d, *J* = 5.1 Hz, 2H), 6.53 (t, *J* = 6.1 Hz, 1H), 5.08 (s, 1H), 4.43 (s, 2H), 4.11 (q, *J* = 7.1 Hz, 2H), 1.43 (t, *J* = 7.0 Hz, 3H); ^{13}C NMR (125 MHz, $CDCl_3$) (δ, ppm) 166.1, 160.9, 149.5, 148.1, 140.4, 140.1, 134.7, 130.5, 130.2, 129.5, 129.1, 128.6, 127.4, 125.8, 124.2, 122.1, 116.7, 84.4, 67.2, 42.7, 14.6; HRMS (TOF MS ESI$^+$) calculated for $C_{27}H_{25}N_2O_3$ $[M+H]^+$: 425.1860, found 425.1858.

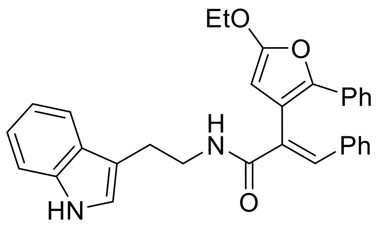

(*E*)-*N*-(2-(1*H*-Indol-3-yl)ethyl)-2-(5-ethoxy-2-phenylfuran-3-yl)-3-phenylacryl amide (17). Yellow oil. 35.2 mg, 74% yield; ^{1}H NMR (400 MHz, $CDCl_3$) (δ, ppm) 8.00 (s, 1H), 7.83 (s, 1H), 7.52 - 7.48 (comp, 3H), 7.36 - 7.35 (m, 2H), 7.31 - 7.25 (comp, 4H), 7.22 - 7.20 (m, 2H), 7.16 (t, *J* = 7.4 Hz, 2H), 7.07 (t, *J* = 7.4 Hz, 1H), 6.50 (s, 1H), 6.13 (t, *J* = 5.3 Hz, 1H), 4.86 (s, 1H), 3.97 (q, *J* = 7.0 Hz, 2H), 3.56 (dd, *J* = 12.4, 6.3 Hz, 2H), 2.79 (t, *J* = 6.5 Hz, 2H), 1.40 (t, *J* = 7.0 Hz, 3H); ^{13}C NMR (100 MHz, $CDCl_3$) (δ, ppm) 165.7, 160.6, 139.7, 139.2, 136.4, 135.0, 130.40, 130.35, 129.2, 128.8, 128.5, 127.2, 127.0, 126.6, 123.9, 122.3, 122.1, 119.5, 118.7, 116.8, 112.7, 111.2, 84.3, 67.1, 40.2, 25.0, 14.6; HRMS (TOF MS ESI$^+$) calculated for $C_{31}H_{28}N_2O_3Na$ $[M+Na]^+$: 499.1992, found 499.1991.

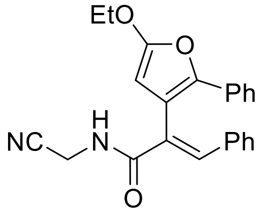

(*E*)-*N*-(Cyanomethyl)-2-(5-ethoxy-2-phenylfuran-3-yl)-3-phenylacrylamide (18). Yellow oil. 30.5 mg, 82% yield; ^{1}H NMR (500 MHz, $CDCl_3$) (δ, ppm) 8.02 (s, 1H), 7.52 - 7.51 (m, 2H), 7.40 - 7.38 (m, 2H), 7.29 - 7.24 (comp, 5H), 7.18 - 7.15 (m, 1H), 6.43 (t, *J* = 5.7 Hz, 1H), 5.08 (s, 1H), 4.15 - 4.09 (comp, 4H), 1.44 (t, *J* = 7.0 Hz, 3H); ^{13}C NMR (125 MHz, $CDCl_3$) (δ, ppm) 166.1, 160.9, 141.4, 140.1, 134.3, 130.6, 129.9, 129.8, 129.0, 128.6, 127.3, 124.7, 123.9, 115.83, 115.77, 84.3, 67.3, 28.2, 14.6; HRMS (TOF MS ESI$^+$) calculated for $C_{23}H_{20}N_2O_3Na$ $[M+Na]^+$: 395.1366 found 395.1369.

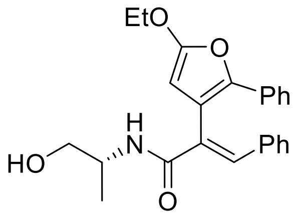

(*R*,*E*)-2-(5-Ethoxy-2-phenylfuran-3-yl)-*N*-(1-hydroxypropan-2-yl)-3-phenylacryl amide (19). Yellow oil. 34.5 mg, 88% yield; ^{1}H NMR (500 MHz, $CDCl_3$) (δ, ppm) 7.96 (s, 1H), 7.55 - 7.54 (m, 2H), 7.40 - 7.38 (m, 2H), 7.32 - 7.29 (m, 2H), 7.25 - 7.24 (comp, 3H), 7.19 (t, *J* = 7.4 Hz, 1H), 6.12 (d, *J* = 7.4 Hz, 1H), 5.03 (s, 1H), 4.11 (q, *J* = 7.0 Hz, 2H), 4.03 - 3.99 (m, 1H), 3.42 (dd, *J* = 11.0, 3.2 Hz, 1H), 3.31 (dd, *J* = 11.0, 5.9 Hz, 1H), 2.54 (bs, 1H), 1.44 (t, *J* = 7.0 Hz, 3H), 0.88 (d, *J* = 6.7 Hz, 3H); ^{13}C NMR (125 MHz, $CDCl_3$) (δ, ppm) 166.2, 160.7, 140.2, 139.8, 134.9, 130.4, 130.3, 129.2, 128.9, 128.5, 127.3, 126.3, 124.2, 117.0, 84.6, 67.2, 67.1, 48.4, 16.7, 14.6; HRMS (TOF MS ESI$^+$) calculated for $C_{24}H_{25}NO_4Na$ $[M+Na]^+$: 414.1676, found 414.1675.

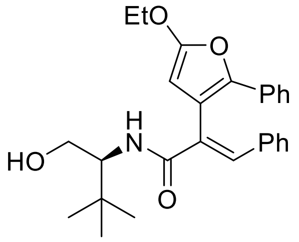

(*S*,*E*)-2-(5-Ethoxy-2-phenylfuran-3-yl)-*N*-(1-hydroxy-3,3-dimethylbutan-2-yl)-3-phenylacrylamide (20). Yellow oil. 36.9 mg, 85% yield; ^{1}H NMR (500 MHz, $CDCl_3$) (δ, ppm) 8.01 (s, 1H), 7.58 - 7.56 (m, 2H), 7.42 - 7.40 (m, 2H), 7.31 - 7.27 (m, 2H), 7.25 - 7.24 (comp, 3H), 7.17 (t, *J* = 7.4 Hz, 1H), 6.26 (d, *J* = 8.5 Hz, 1H), 5.10 (s, 1H), 4.13 (q, *J* = 7.0 Hz, 2H), 3.79 - 3.75 (m, 1H), 3.68 - 3.67 (m, 1H), 3.36 - 3.26 (m, 1H), 2.40 (bs, 1H), 1.44 (t, *J* = 7.0 Hz, 3H), 0.69 (s, 9H); ^{13}C NMR (100 MHz, $CDCl_3$) (δ, ppm) 167.0, 160.9, 140.2, 140.0, 134.8, 130.4, 130.2, 129.3, 129.0, 128.6, 127.3, 126.3, 124.1, 117.0, 84.5, 67.3, 63.7, 60.7, 33.4, 26.7, 14.6; HRMS (TOF MS ESI$^+$) calculated for $C_{27}H_{31}NO_4Na$ $[M+Na]^+$: 456.2145, found 456.2145.

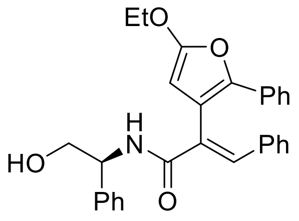

(*S*,*E*)-2-(5-Ethoxy-2-phenylfuran-3-yl)-*N*-(2-hydroxy-1-phenylethyl)-3-phenylacrylamide (21). Yellow oil. 39.0 mg, 86% yield, $[\alpha]_D^{20}$ = -54.00 (c = 0.05, EtOAc); ^{1}H NMR (500 MHz, $CDCl_3$) (δ, ppm) 7.99 (s, 1H), 7.57 (d, *J* = 7.7 Hz, 2H), 7.41 - 7.40 (m, 2H), 7.30 (t, *J* = 7.4 Hz, 2H), 7.25 - 7.19 (comp, 4H), 7.17 - 7.16 (comp, 3H), 6.91 - 6.90 (m, 2H), 6.70 (d, *J* = 7.0 Hz, 1H), 5.05 - 5.02 (m, 2H), 4.11 - 4.07 (m, 2H), 3.67 - 3.62 (m, 2H), 2.42 (bs, 1H), 1.41 (t, *J* = 6.9 Hz, 3H); ^{13}C NMR (125 MHz, $CDCl_3$) (δ, ppm) 166.1, 160.8, 140.2, 140.1, 138.8, 134.8, 130.5, 130.3, 129.3, 129.0, 128.8, 128.5, 127.7, 127.3, 126.5, 126.1, 124.3, 116.8, 84.5, 67.2, 66.4, 56.3, 14.5; HRMS (TOF MS ESI$^+$) calculated for $C_{29}H_{28}NO_4$ $[M+H]^+$: 454.2013, found 454.2011.

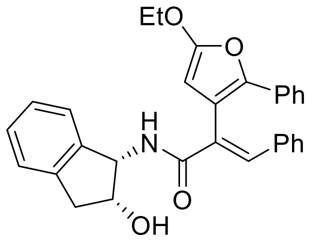

(*E*)-2-(5-Ethoxy-2-phenylfuran-3-yl)-*N*-((1*S*,2*R*)-2-hydroxy-2,3-dihydro-1*H*-inden-1-yl)-3-phenylacrylamide (22). Yellow oil. 40.0 mg, 86% yield, $[\alpha]_D^{20}$ = -102.00 (c = 0.05, EtOAc); ^{1}H NMR (500 MHz, $CDCl_3$) (δ, ppm) 8.07 (s, 1H), 7.60 - 7.58 (m, 2H), 7.45 - 7.43 (m, 2H), 7.36 - 7.33 (m, 2H), 7.27 - 7.22 (comp, 5H), 7.15 (d, *J* = 4.1 Hz, 2H), 7.02 (s, 1H), 6.61 (d, *J* = 8.4 Hz, 1H), 5.41 (dd, *J* = 8.4, 4.9 Hz, 1H), 5.04 (s, 1H), 4.42 (bs, 1H), 4.05 (q, *J* = 7.1 Hz, 2H), 3.05 (dd, *J* = 16.5, 5.1 Hz, 1H), 2.80 (d, *J* = 16.6 Hz, 1H), 1.38 (t, *J* = 7.1 Hz, 3H); ^{13}C NMR (125 MHz, $CDCl_3$) (δ, ppm) 166.3, 160.7, 140.3, 140.1, 140.0, 134.9, 130.52, 130.48, 129.3, 129.0, 128.5, 128.2, 127.24, 127.18, 126.3, 125.2, 124.21, 124.19, 117.0, 84.6, 73.8, 67.2, 58.3, 39.5, 14.5; HRMS (TOF MS ESI$^+$) calculated for $C_{30}H_{27}NO_4Na$ $[M+Na]^+$: 488.1832,

found 488.1833.

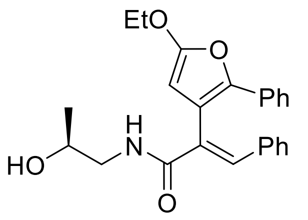

(*S*,*E*)-2-(5-Ethoxy-2-phenylfuran-3-yl)-*N*-(2-hydroxypropyl)-3-phenylacrylamide (23). Yellow oil. 32.1 mg, 82% yield, $[\alpha]_D^{20}$ = -14.00 (c = 0.05, EtOAc); ^{1}H NMR (500 MHz, $CDCl_3$) (δ, ppm) 7.99 (s, 1H), 7.57 - 7.56 (m, 2H), 7.40 - 7.39 (m, 2H), 7.32 - 7.28 (m, 2H), 7.25 - 7.23 (comp, 3H), 7.19 - 7.17 (m, 1H), 6.47 (t, *J* = 5.7 Hz, 1H), 5.05 (s, 1H), 4.12 (q, *J* = 7.1 Hz, 2H), 3.70 - 3.64 (m, 1H), 3.41 - 3.37 (m, 1H), 3.06 - 3.01 (m, 1H), 2.35 (bs, 1H), 1.44 (t, *J* = 7.0 Hz, 3H), 0.97 (d, *J* = 6.3 Hz, 3H); ^{13}C NMR (125 MHz, $CDCl_3$) (δ, ppm) 166.7, 160.8, 140.0, 139.8, 134.8, 130.4, 130.3, 129.3, 128.9, 128.5, 127.2, 126.2, 124.1, 116.9, 84.6, 67.5, 67.2, 47.7, 20.6, 14.6; HRMS (TOF MS ESI$^+$) calculated for $C_{24}H_{25}NO_4Na$ $[M+Na]^+$: 414.1676, found 414.1678.

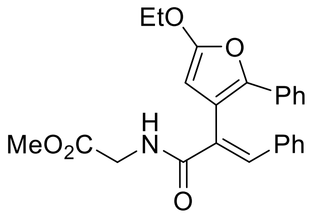

Methyl (*E*)-(2-(5-ethoxy-2-phenylfuran-3-yl)-3-phenylacryloyl)glycinate (24). Yellow oil. 33.7 mg, 83% yield; ^{1}H NMR (400 MHz, $CDCl_3$) (δ, ppm) 7.99 (s, 1H), 7.55 (d, *J* = 7.7 Hz, 2H), 7.39 - 7.37 (m, 2H), 7.28 - 7.22 (comp, 5H), 7.14 (t, *J* = 7.4 Hz, 1H), 6.53 (t, *J* = 5.2 Hz, 1H), 5.13 (s, 1H), 4.15 (q, *J* = 7.0 Hz, 2H), 4.04 (d, *J* = 5.4 Hz, 2H), 3.64 (s, 3H), 1.46 (t, *J* = 7.0 Hz, 3H); ^{13}C NMR (100 MHz, $CDCl_3$) (δ, ppm) 170.1, 166.3, 160.8, 140.1, 140.1, 134.7, 130.4, 130.2, 129.4, 128.8, 128.5, 127.0, 125.8, 124.1, 116.2, 84.4, 67.2, 52.4, 42.0, 14.6; HRMS (TOF MS ESI$^+$) calculated for $C_{24}H_{23}NO_5Na$ $[M+Na]^+$: 428.1468, found 428.1470.

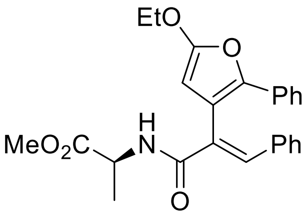

Methyl (*E*)-(2-(5-ethoxy-2-phenylfuran-3-yl)-3-phenylacryloyl)-*L*-alaninate (25). Yellow oil. 36.9 mg, 88% yield; ^{1}H NMR (500 MHz, $CDCl_3$) (δ, ppm) 7.96 (s, 1H), 7.54 (d, *J* = 7.7 Hz, 2H), 7.39 - 7.36 (m, 2H), 7.28 - 7.25 (m, 2H), 7.23 - 7.22 (comp, 3H), 7.15 (t, *J* = 7.4 Hz, 1H), 6.53 (d, *J* = 7.5 Hz, 1H), 5.09 (s, 1H), 4.63 - 4.58 (m, 1H), 4.13 (q, *J* = 7.1 Hz, 2H), 3.61 (s, 3H), 1.45 (t, *J* = 7.0 Hz, 3H), 1.16 (d, *J* = 7.1 Hz, 3H); ^{13}C NMR (125 MHz, $CDCl_3$) (δ, ppm) 173.1, 165.3, 160.7, 140.3, 139.8, 134.8, 130.4, 130.3, 129.3, 128.7, 128.5, 127.0, 126.1, 124.2, 116.5, 84.5, 67.2, 52.4, 48.6, 18.1, 14.6; HRMS (TOF MS ESI$^+$) calculated for $C_{25}H_{26}NO_5$ $[M+H]^+$: 420.1805, found 420.1808.

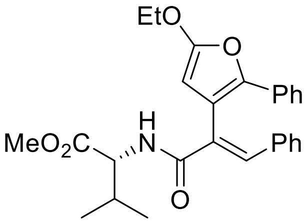

Methyl (*E*)-(2-(5-ethoxy-2-phenylfuran-3-yl)-3-phenylacryloyl)-*D*-valinate (26). Yellow oil. 38.0 mg, 85% yield, $[\alpha]_D^{20}$ = 52.00 (c = 0.05, EtOAc); ^{1}H NMR (500 MHz, $CDCl_3$) (δ, ppm) 7.98 (s, 1H), 7.57 - 7.55 (m, 2H), 7.40 - 7.38 (m, 2H), 7.28 - 7.23 (comp, 5H), 7.16 – 7.13 (m, 1H), 6.50 (d, *J* = 8.7 Hz, 1H), 5.11 (s, 1H), 4.56 (dd, *J* = 8.8, 4.9 Hz, 1H), 4.17 - 4.11 (m, 2H), 3.59 (s, 3H), 2.04 - 1.97 (m, 1H), 1.45 (t, *J* = 7.1 Hz, 3H), 0.68 (d, *J* = 6.7 Hz, 3H), 0.58 (d, *J* = 6.7 Hz, 3H); ^{13}C NMR (125 MHz, $CDCl_3$) (δ, ppm) 172.1, 165.6, 160.8, 140.3, 139.9, 134.8, 130.4, 130.3, 129.3, 128.8, 128.5, 127.1, 126.2, 124.2, 116.5, 84.4, 52.1, 31.3, 18.8, 17.4, 14.6; HRMS (TOF MS ESI$^+$) calculated for $C_{27}H_{29}NO_5Na$ $[M+Na]^+$: 470.1938, found 470.1936.

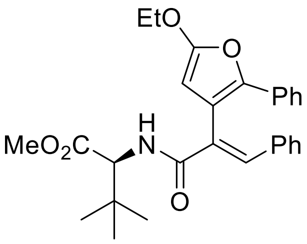

Methyl (*S*,*E*)-2-(2-(5-ethoxy-2-phenylfuran-3-yl)-3-phenylacrylamido)-3,3-dimethylbutanoate (27). Yellow oil. 39.7 mg, 86% yield; ^{1}H NMR (500 MHz, $CDCl_3$) (δ, ppm) 7.98 (s, 1H), 7.57 - 7.56 (m, 2H), 7.41 - 7.38 (m, 2H), 7.26 - 7.23 (comp, 5H), 7.16 - 7.13 (m, 1H), 6.56 (d, *J* = 9.2 Hz, 1H), 5.10 (s, 1H), 4.45 (d, *J* = 9.3 Hz, 1H), 4.17 - 4.11 (m, 2H), 3.56 (s, 3H), 1.45 (t, *J* = 7.0 Hz, 3H), 0.71 (s, 9H); ^{13}C NMR (125 MHz, $CDCl_3$) (δ, ppm) 171.6, 165.5, 160.8, 140.3, 139.9, 134.8, 130.4, 130.3, 129.3, 128.8, 128.5, 127.1, 126.3, 124.2, 116.5, 84.4, 67.3, 60.7, 51.7, 34.7, 26.3, 14.5; HRMS (TOF MS ESI$^+$) calculated for $C_{28}H_{31}NO_5Na$ $[M+Na]^+$: 484.2094, found 484.2094.

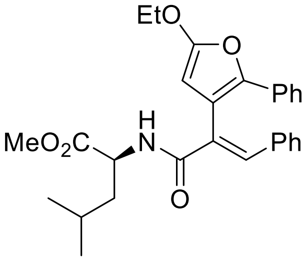

Methyl (*E*)-(2-(5-ethoxy-2-phenylfuran-3-yl)-3-phenylacryloyl)-*L*-leucinate (28). Yellow oil. 41.1 mg, 89% yield; ^{1}H NMR (500 MHz, $CDCl_3$) (δ, ppm) 7.98 (s, 1H), 7.55 (d, *J* = 7.6 Hz, 2H), 7.40 - 7.38 (m, 2H), 7.28 - 7.23 (comp, 5H), 7.17 - 7.14 (t, *J* = 7.4 Hz, 1H), 6.34 - 6.32 (m, 1H), 5.09 (s, 1H), 4.66 - 4.62 (m, 1H), 4.16 - 4.10 (m, 2H), 3.60 (s, 3H), 1.45 (t, *J* = 7.0 Hz, 3H), 1.34 - 1.27 (m, 2H), 1.14 - 1.11 (m, 1H), 0.73 (d, *J* = 6.4 Hz, 3H), 0.70 (d, *J* = 6.4 Hz, 3H); ^{13}C NMR (125 MHz, $CDCl_3$) (δ, ppm) 173.2, 165.6, 160.7, 140.2, 140.0, 134.8, 130.4, 130.3, 129.3, 128.8, 128.5, 127.0, 126.0, 124.1, 116.5, 84.5, 67.2, 52.2, 51.2, 41.2, 24.6, 22.9, 21.7, 14.6; HRMS (TOF MS ESI$^+$) calculated for $C_{28}H_{31}NO_5Na$ $[M+Na]^+$: 484.2094, found 484.2097.

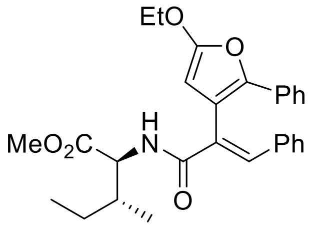

Methyl ((*E*)-2-(5-ethoxy-2-phenylfuran-3-yl)-3-phenylacryloyl)-*L*-alloisoleucinate (29). Yellow oil. 37.4 mg, 81% yield; ^{1}H NMR (400 MHz, $CDCl_3$) (δ, ppm) 7.97 (s, 1H), 7.55 (d, *J* = 7.7 Hz, 2H), 7.41 - 7.38 (m, 2H), 7.28 - 7.23 (comp, 5H), 7.15 (t, *J* = 7.4 Hz, 1H), 6.50 (d, *J* = 8.6 Hz, 1H), 5.09 (s, 1H), 4.59 (dd, *J* = 8.6, 5.1 Hz, 1H), 4.17 - 4.11 (m, 2H), 3.59 (s, 3H), 1.71 - 1.66 (m, 2H), 1.45 (t, *J* = 7.0 Hz, 3H), 1.18 - 1.11 (m, 1H), 0.73 - 0.70 (m, 3H), 0.62 (d, *J* = 6.8 Hz, 3H); ^{13}C NMR (100 MHz, $CDCl_3$) (δ, ppm) 172.2, 165.5, 160.8, 140.3, 139.9, 134.9, 130.4, 130.3, 129.3, 128.8, 128.5, 127.1, 126.3, 124.2, 116.6, 84.5, 67.3, 57.1, 52.1, 37.9, 24.9, 15.3, 14.6, 11.4; HRMS (TOF MS ESI$^+$) calculated for $C_{28}H_{31}NO_5Na$ $[M+Na]^+$: 484.2094, found 484.2092.

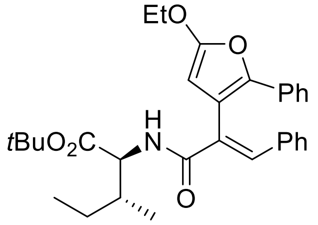

***tert*-Butyl ((*E*)-2-(5-ethoxy-2-phenylfuran-3-yl)-3-phenylacryloyl)-*L*-alloisoleucinate (30).** Yellow oil. 42.8 mg, 85% yield; ^{1}H NMR (500 MHz, $CDCl_3$) (δ, ppm) 7.97 (s, 1H), 7.56 - 7.55 (m, 2H), 7.41 - 7.39 (m, 2H), 7.27 - 7.22 (comp, 5H), 7.15 - 7.12 (m, 1H), 6.56 (d, *J* = 8.6 Hz, 1H), 5.10 (s, 1H), 4.50 (dd, *J* = 8.6, 4.6 Hz, 1H), 4.17 - 4.09 (m, 2H), 1.69 - 1.65 (m, 1H), 1.44 (t, *J* = 7.1 Hz, 3H), 1.36 (s, 9H), 1.23 - 1.20 (m, 1H), 0.83 - 0.80 (m, 1H), 0.75 (t, *J* = 6.8 Hz, 3H), 0.59 (d, *J* = 6.3 Hz, 3H); ^{13}C NMR (125 MHz, $CDCl_3$) (δ, ppm) 170.4, 165.2, 160.7, 140.2, 139.5, 134.9, 130.4, 130.3, 129.2, 128.7, 128.5, 127.0, 126.5, 124.2, 116.6, 84.5, 81.8, 67.2, 57.4, 38.4, 28.0, 25.0, 15.1, 14.5, 11.6; HRMS (TOF MS ESI$^+$) calculated for $C_{31}H_{37}NO_5Na$ $[M+Na]^+$: 526.2564, found 526.2569.

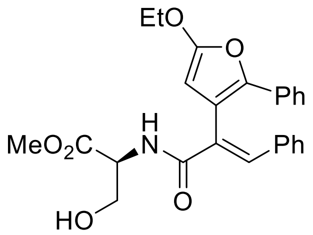

Methyl (*E*)-(2-(5-ethoxy-2-phenylfuran-3-yl)-3-phenylacryloyl)-*L*-serinate (31). Yellow oil. 40.5 mg, 93% yield, $[\alpha]_D^{20}$ = -8.00 (c = 0.05, EtOAc); ^{1}H NMR (400 MHz, $CDCl_3$) (δ, ppm) 7.96 (s, 1H), 7.57 (d, *J* = 7.6 Hz, 2H), 7.42 - 7.40 (m, 2H), 7.33 - 7.29 (m, 2H), 7.26 - 7.25 (comp, 3H), 7.19 (t, *J* = 7.4 Hz, 1H), 6.89 (d, *J* = 7.2 Hz, 1H), 5.08 (s, 1H), 4.65 - 4.61 (m, 1H), 4.12 (q, *J* = 7.0 Hz, 2H), 3.77 - 3.73 (m, 2H), 3.67 - 3.64 (comp, 4H), 1.44 (t, *J* = 7.0 Hz, 3H); ^{13}C NMR (100 MHz, $CDCl_3$) (δ, ppm) 170.6, 166.0, 160.8, 140.2, 134.7, 130.5, 129.4, 128.9, 128.5, 127.2, 125.9, 124.3, 116.6, 84.5, 67.2, 63.4, 55.3, 52.8, 14.6; HRMS (TOF MS ESI$^+$) calculated for $C_{25}H_{25}NO_6Na$ $[M+Na]^+$: 458.1574, found 458.1574.

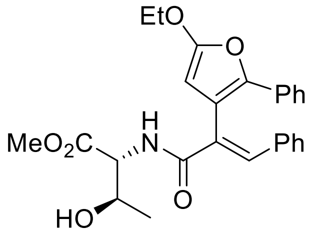

Methyl ((*E*)-2-(5-ethoxy-2-phenylfuran-3-yl)-3-phenylacryloyl)-*D*-allothreoninate (32). Yellow oil. 41.8 mg, 93% yield, $[\alpha]_D^{20}$ = 78.00 (c = 0.05, EtOAc); ^{1}H NMR (500 MHz, $CDCl_3$) (δ, ppm) 7.98 (s, 1H), 7.56 (d, *J* = 7.6 Hz, 2H), 7.42 - 7.40 (m, 2H), 7.29 - 7.24 (comp, 5H), 7.17 - 7.14 (m, 1H), 6.73 (d, *J* = 8.7 Hz, 1H), 5.11 (s, 1H), 4.60 - 4.58 (m, 1H), 4.18 - 4.10 (comp, 3H), 3.61 (s, 3H), 2.02 (bs, 1H), 1.44 (t, *J* = 7.0 Hz, 3H), 0.88 (d, *J* = 4.1 Hz, 3H); ^{13}C NMR (125 MHz, $CDCl_3$) (δ, ppm) 171.2, 166.3, 160.8, 140.2, 134.7, 130.44, 130.39, 129.4, 128.8, 128.5, 127.1, 126.0, 124.3, 116.4, 84.4, 68.0, 67.2, 57.8, 52.5, 19.7, 14.5; HRMS (TOF MS ESI$^+$) calculated for $C_{26}H_{27}NO_6Na$ $[M+Na]^+$: 472.1731, found 472.1730.

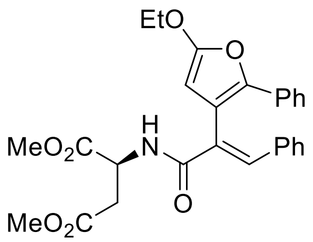

Dimethyl (*E*)-(2-(5-ethoxy-2-phenylfuran-3-yl)-3-phenylacryloyl)-*L*-aspartate (33). Yellow oil. 36.8 mg, 77% yield; ^{1}H NMR (500 MHz, $CDCl_3$) (δ, ppm) 7.96 (s, 1H), 7.52 (d, *J* = 7.8 Hz, 2H), 7.40 - 7.39 (m, 2H), 7.27 - 7.23 (comp, 5H), 7.16 - 7.13 (m, 1H), 7.03 (d, *J* = 8.3 Hz, 1H), 5.09 (s, 1H), 4.90 - 4.87 (m, 1H), 4.17 - 4.11 (m, 2H), 3.60 (s, 3H), 3.44 (s, 3H), 2.86 (dd, *J* = 17.1, 4.3 Hz, 1H), 2.52 - 2.49 (m, 1H), 1.45 (t, *J* = 7.0 Hz, 3H); ^{13}C NMR (125 MHz, $CDCl_3$) (δ, ppm) 171.1, 171.0, 165.6, 160.7, 140.3, 140.0, 134.8, 130.44, 130.40, 129.3, 128.6, 128.5, 126.9, 126.1, 124.1, 116.3, 67.2, 52.7, 51.8, 48.9, 35.9, 14.5; HRMS (TOF MS ESI$^+$) calculated for $C_{27}H_{27}NO_7Na$ $[M+Na]^+$: 500.1680, found 500.1681.

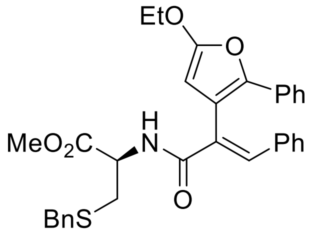

Methyl (*E*)-*S*-benzyl-*N*-(2-(5-ethoxy-2-phenylfuran-3-yl)-3-phenylacryloyl)-*L*-cysteinate (34). Yellow oil. 40.1 mg, 74% yield, $[\alpha]_D^{20}$ = -38.00 (c = 0.05, EtOAc); ^{1}H NMR (500 MHz, $CDCl_3$) (δ, ppm) 7.98 (s, 1H), 7.56 - 7.55 (m, 2H), 7.41 - 7.39 (m, 2H), 7.28 - 7.22 (comp, 10H), 7.16 (d, *J* = 7.1 Hz, 2H), 7.11 (t, *J* = 7.4 Hz, 1H), 6.83 (d, *J* = 7.6 Hz, 1H), 5.13 (s, 1H), 4.81 - 4.77 (m, 1H), 4.15 - 4.09 (m, 2H), 3.59 (s, 3H), 3.47 (q, *J* = 13.2 Hz, 2H), 2.69 (d, *J* = 5.4 Hz, 2H), 1.44 (t, *J* = 7.0 Hz, 3H); ^{13}C NMR (125 MHz, $CDCl_3$) (δ, ppm) 171.1, 165.8, 160.8, 140.3, 140.1, 137.6, 134.8, 130.4, 130.3, 129.4, 129.0, 128.7, 128.6, 128.5, 127.3, 127.0, 126.0, 124.2, 116.2, 84.5, 67.2, 52.6, 52.3, 36.4, 33.2, 14.6; HRMS (TOF MS ESI$^+$) calculated for $C_{32}H_{31}NO_5Na$ $[M+Na]^+$: 564.1815, found 564.1816.

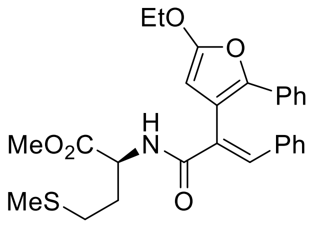

Methyl (*E*)-(2-(5-ethoxy-2-phenylfuran-3-yl)-3-phenylacryloyl)-*L*-methioninate (35). Yellow oil. 40.7 mg, 85% yield; ^{1}H NMR (400 MHz, $CDCl_3$) (δ, ppm) 7.97 (s, 1H), 7.56 (d, *J* = 8.0 Hz, 2H), 7.41 - 7.39 (m, 2H), 7.30 - 7.24 (m, 5H), 7.17 (t, *J* = 7.3 Hz, 1H), 6.72 (d, *J* = 7.9 Hz, 1H), 5.10 (s, 1H), 4.75 - 4.70 (m, 1H), 4.14 (q, *J* = 7.0 Hz, 2H), 3.62 (s, 3H), 2.11 - 2.09 (m, 2H), 2.04 - 1.98 (m, 1H), 1.90 (s, 3H), 1.81 - 1.76 (m, 1H), 1.45 (t, *J* = 7.0 Hz, 3H); ^{13}C NMR (100 MHz, $CDCl_3$) (δ, ppm) 172.1, 165.7, 160.8, 140.2, 140.0, 134.7, 130.5, 130.3, 129.4, 128.8, 128.5, 127.1, 125.9, 124.1, 116.5, 84.4, 67.2, 52.5, 52.1, 31.2, 29.6, 15.3, 14.6; HRMS (TOF MS ESI$^+$) calculated for $C_{27}H_{29}NO_5SNa$ [M+Na]$^+$: 502.1659, found 502.1657.

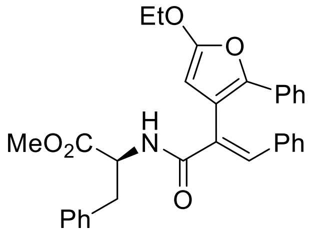

Methyl (*E*)-(2-(5-ethoxy-2-phenylfuran-3-yl)-3-phenylacryloyl)-*L*-phenyl alaninate (36). Yellow oil. 37.7 mg, 76% yield, $[\alpha]_D^{20}$ = -26.00 (c = 0.05, EtOAc); ^{1}H NMR (400 MHz, $CDCl_3$) (δ, ppm) 7.95 (s, 1H), 7.52 (d, *J* = 7.8 Hz, 2H), 7.38 - 7.36 (m, 2H), 7.27 - 7.21 (comp, 5H), 7.16 - 7.09 (comp, 4H), 6.84 (d, *J* = 6.9 Hz, 2H), 6.42 (d, *J* = 7.9 Hz, 1H), 4.99 (s, 1H), 4.90 - 4.85 (m, 1H), 4.07 (q, *J* = 7.0 Hz, 2H), 3.59 (s, 3H), 2.97 (t, *J* = 6.6 Hz, 2H), 1.44 (t, *J* = 7.0 Hz, 3H); ^{13}C NMR (100 MHz, $CDCl_3$) (δ, ppm) 171.7, 165.6, 160.7, 139.9, 135.6, 134.7, 130.4, 130.3, 129.4, 129.2, 128.7, 128.6, 128.5, 127.1, 127.0, 125.9, 124.0, 116.1, 84.2, 67.1, 53.5, 52.3, 37.7, 14.6; HRMS (TOF MS ESI$^+$) calculated for $C_{31}H_{29}NO_5Na$ [M+Na]$^+$: 518.1938, found 518.1937.

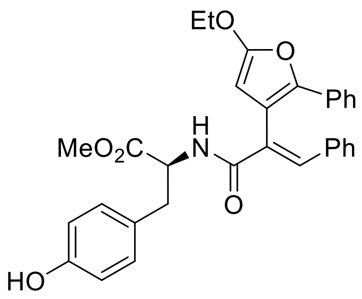

Methyl (*E*)-(2-(5-ethoxy-2-phenylfuran-3-yl)-3-phenylacryloyl)-*L*-tyrosinate (37). Yellow oil. 44.5 mg, 87% yield, $[\alpha]_D^{20}$ = -20.00 (c = 0.05, EtOAc); ^{1}H NMR (500 MHz, $CDCl_3$) (δ, ppm) 7.94 (s, 1H), 7.52 (d, *J* = 7.7 Hz, 2H), 7.36 - 7.34 (m, 2H), 7.25 - 7.19 (comp, 5H), 7.15 - 7.12 (m, 1H), 6.68 (d, *J* = 7.9 Hz, 2H), 6.59 (d, *J* = 8.2 Hz, 2H), 6.49 (d, *J* = 8.0 Hz, 1H), 4.99 (s, 1H), 4.85 - 4.81 (m, 1H), 4.07 (q, *J* = 7.0 Hz, 2H), 3.58 (s, 3H), 2.92 - 2.84 (m, 2H), 1.43 (t, *J* = 7.0 Hz, 3H); ^{13}C NMR (125 MHz, $CDCl_3$) (δ, ppm) 171.9, 166.0, 160.8, 155.5, 140.2, 140.0, 134.6, 130.4, 130.2, 129.5, 128.8, 128.5, 127.0, 126.8, 125.8, 124.0, 115.9, 115.7, 84.2, 67.2, 53.8, 52.4, 37.0, 14.5; HRMS (TOF MS ESI$^+$) calculated for $C_{31}H_{29}NO_6Na$ $[M+Na]^+$: 534.1887, found 534.1886.

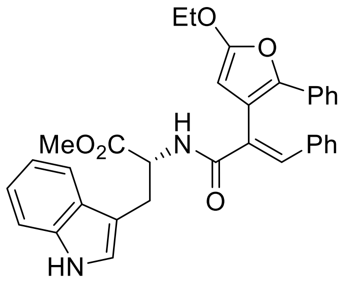

Methyl (*E*)-(2-(5-ethoxy-2-phenylfuran-3-yl)-3-phenylacryloyl)-*D*-tryptophanate (38). Yellow solid, mp = 136 - 137 °C.. 43.8 mg, 82% yield; ^{1}H NMR (400 MHz, $CDCl_3$) (δ, ppm) 7.97 (s, 1H), 7.84 (bs, 1H), 7.49 (d, *J* = 7.8 Hz, 2H), 7.39 (d, *J* = 7.9 Hz, 1H), 7.36 - 7.34 (m, 2H), 7.27 - 7.20 (comp, 6H), 7.13 (t, *J* = 7.8 Hz, 2H), 7.03 (t, *J* = 7.5 Hz, 1H), 6.53 (d, *J* = 7.6 Hz, 1H), 6.48 (bs, 1H) 4.91 - 4.85 (m, 2H), 3.93 (q, *J* = 7.0 Hz, 2H), 3.55 (s, 3H), 3.24 - 3.13 (m, 2H), 1.39 (t, *J* = 7.0 Hz, 3H); ^{13}C NMR (100 MHz, $CDCl_3$) (δ, ppm) 172.2, 165.8, 160.7, 139.9, 139.8, 136.1, 134.8, 130.4, 129.3, 128.7, 128.5, 127.4, 126.9, 126.1, 124.1, 123.0, 122.2, 119.7, 118.5, 116.3,

111.2, 109.7, 84.3, 67.1, 53.3, 52.4, 27.4, 14.5; HRMS (TOF MS ESI$^+$) calculated for $C_{33}H_{30}N_2O_5Na$ [M+Na]$^+$: 557.2047, found 557.2045.

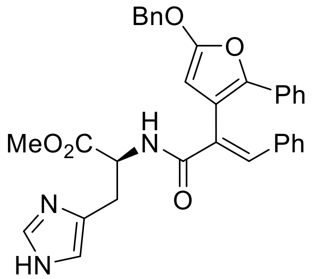

Methyl (*E*)-(2-(5-(benzyloxy)-2-phenylfuran-3-yl)-3-phenylacryloyl)-*L*-histidinate (39). Yellow oil. 45.9 mg, 84% yield; ^{1}H NMR (400 MHz, $CDCl_3$) (δ, ppm) 7.95 (s, 1H), 7.53 (d, *J* = 8.0 Hz, 2H), 7.44 (d, *J* = 7.4 Hz, 2H), 7.41 - 7.35 (comp, 5H), 7.27 - 7.26 (comp, 4H), 7.24 - 7.20 (comp, 3H), 7.14 (t, *J* = 7.4 Hz, 1H), 6.45 (bs, 1H), 5.18 (s, 1H), 5.13 (s, 2H), 4.81 - 4.77 (m, 1H), 3.58 (s, 3H), 3.03 - 2.88 (m, 3H); ^{13}C NMR (100 MHz, $CDCl_3$) (δ, ppm) 171.8, 166.0, 160.4, 140.4, 139.9, 135.3, 134.8, 130.6, 130.4, 129.4, 128.84, 128.80, 128.77, 128.6, 128.1, 126.9, 126.1, 124.2, 116.5, 86.0, 73.3, 52.9, 52.5, 29.1; HRMS (TOF MS ESI$^+$) calculated for $C_{33}H_{30}N_3O_5$ [M+H]$^+$: 548.2180, found 548.2178.

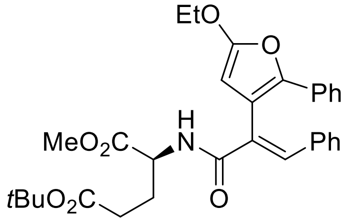

5-(*tert*-Butyl) 1-methyl (*E*)-(2-(5-ethoxy-2-phenylfuran-3-yl)-3-phenylacryloyl)-*L*-glutamate (40). Yellow oil. 39.0 mg, 73% yield, $[\alpha]_D^{20}$ = -48.00 (c = 0.05, EtOAc); ^{1}H NMR (500 MHz, $CDCl_3$) (δ, ppm) 7.96 (s, 1H), 7.55 (d, *J* = 8.0 Hz, 2H), 7.39 (s, 2H), 7.28 - 7.24 (comp, 5H), 7.14 (t, *J* = 7.2 Hz, 1H), 6.67 (d, *J* = 7.7 Hz, 1H), 5.09 (s, 1H), 4.64 - 4.60 (m, 1H), 4.14 (q, *J* = 7.0 Hz, 2H), 3.60 (s, 3H), 1.99 - 1.96 (m, 2H), 1.73 - 1.71 (m, 2H), 1.45 (t, *J* = 6.8 Hz, 3H), 1.40 (s, 9H); ^{13}C NMR (125 MHz, $CDCl_3$) (δ, ppm) 172.1, 172.0, 165.7, 160.8, 140.3, 139.9, 134.8, 130.4, 130.3, 129.3, 128.8, 128.5, 127.1, 126.1, 124.2, 116.4, 84.5, 80.6, 67.2, 52.4, 52.2, 31.1, 28.1, 27.4,

14.6; HRMS (TOF MS ESI$^+$) calculated for $C_{31}H_{35}NO_7Na$ [M+Na]$^+$: 556.2306, found 556.2309.

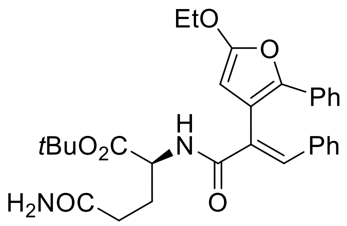

***tert*-Butyl (*E*)-(2-(5-ethoxy-2-phenylfuran-3-yl)-3-phenylacryloyl)-*L*-glutaminate (41).** Yellow oil. 35.3 mg, 68% yield; ^{1}H NMR (400 MHz, $CDCl_3$) (δ, ppm) 7.94 (s, 1H), 7.56 (d, *J* = 7.7 Hz, 2H), 7.42 - 7.40 (m, 2H), 7.30 - 7.25 (comp, 5H), 7.16 (t, *J* = 7.4 Hz, 1H), 6.66 (d, *J* = 7.8 Hz, 1H), 6.09 (bs, 1H), 5.22 (bs, 1H), 5.10 (s, 1H), 4.52 - 4.47 (m, 1H), 4.15 (q, *J* = 7.0 Hz, 2H), 2.08 - 2.01 (m, 2H), 1.94 - 1,84 (m, 2H), 1.46 (t, *J* = 7.0 Hz, 3H), 1.36 (s, 9H); ^{13}C NMR (125 MHz, $CDCl_3$) (δ, ppm) 174.5, 170.5, 166.2, 160.9, 140.1, 140.0, 134.7, 130.6, 130.5, 129.4, 128.9, 128.6, 127.0, 126.1, 124.2, 116.6, 84.5, 82.6, 67.2, 52.8, 31.6, 29.1, 28.0, 14.6; HRMS (TOF MS ESI$^+$) calculated for $C_{30}H_{34}N_2O_6Na$ [M+Na]$^+$: 541.2309, found 541.2308.

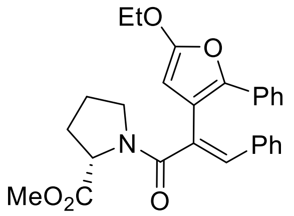

Methyl (*E*)-(2-(5-ethoxy-2-phenylfuran-3-yl)-3-phenylacryloyl)-*L*-prolinate (42). Yellow oil. 32.1 mg, 72% yield; ^{1}H NMR (500 MHz, $CDCl_3$) (δ, ppm) 7.54 (d, *J* = 7.6 Hz, 2H), 7.35 - 7.34 (m, 2H), 7.29 (s, 1H), 7.26 (s, 1H), 7.25 - 7.23 (m, 2H), 7.19 - 7.17 (m, 2H), 7.15 - 7.12 (m, 1H), 5.11 (s, 1H), 4.33 - 4.31 (m, 1H), 4.10 - 4.06 (m, 2H), 3.65 (s, 3H), 3.43 - 3.39 (m, 1H), 3.29 - 3.24 (m, 1H), 1.97 - 1.94 (m, 1H), 1.84 - 1.81 (m, 1H), 1.78 - 1.73 (m, 2H), 1.41 (t, *J* = 7.1 Hz, 3H); ^{13}C NMR (125 MHz, $CDCl_3$) (δ, ppm) 172.9, 168.7, 160.1, 140.5, 138.0, 135.5, 130.6, 129.8, 129.5, 128.6, 128.4, 128.2, 126.9, 124.8, 117.0, 84.8, 67.1, 60.0, 52.2, 48.5, 28.9, 25.5, 14.6; HRMS

(TOF MS ESI$^+$) calculated for $C_{27}H_{27}NO_5Na$ [M+Na]$^+$: 468.1781, found 468.1776.

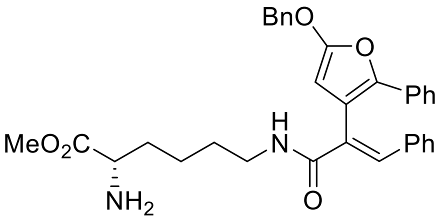

Methyl (*E*)-*N*^{6}-(2-(5-(benzyloxy)-2-phenylfuran-3-yl)-3-phenylacryloyl)-*L*-lysinate (43). Yellow oil. 40.9 mg, 76% yield; ^{1}H NMR (500 MHz, $CDCl_3$) (δ, ppm) 7.96 (s, 1H), 7.54 (d, *J* = 7.8 Hz, 2H), 7.44 - 7.41 (m, 2H), 7.39 - 7.36 (m, 2H), 7.34 - 7.33 (m, 2H), 7.29 - 7.26 (m, 2H), 7.22 - 7.15 (comp, 5H), 6.13 - 6.11 (m, 1H), 5.13 - 5.12 (comp, 3H), 3.70 (s, 3H), 3.40 - 3.37 (m, 1H), 3.23 - 3.19 (m, 2H), 2.53 (bs, 2H), 1.65 - 1.60 (m, 1H), 1.49 - 1.43 (m, 1H), 1.41 - 1.37 (m, 1H), 1.30 - 1.27 (m, 2H), 1.17 - 1.12 (m, 2H); ^{13}C NMR (125 MHz, $CDCl_3$) (δ, ppm) 175.6, 165.7, 160.3, 140.3, 139.4, 135.1, 134.9, 130.3, 130.2, 129.1, 128.9, 128.84, 128.82, 128.5, 128.1, 127.2, 126.4, 124.1, 116.8, 86.0, 73.2, 54.1, 52.2, 39.8, 33.8, 29.1, 22.8; HRMS (TOF MS ESI$^+$) calculated for $C_{33}H_{35}N_2O_5$ [M+H]$^+$: 539.2540, found 539.2540.

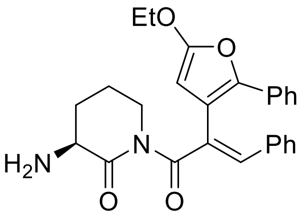

(*S*,*E*)-3-Amino-1-(2-(5-ethoxy-2-phenylfuran-3-yl)-3-phenylacryloyl)piperidin-2-one (44). Yellow oil. 27.9 mg, 65% yield; ^{1}H NMR (400 MHz, $CDCl_3$) (δ, ppm) 7.93 (s, 1H), 7.53 (d, *J* = 8.4 Hz, 2H), 7.39 - 7.37 (m, 2H), 7.28 - 7.26 (m, 2H), 7.23 - 7.22 (comp, 3H), 7.14 (t, *J* = 7.4 Hz, 1H), 6.81 (d, *J* = 5.8 Hz, 1H), 5.94 (bs, 1H), 5.11 (s, 1H), 4.32 - 4.27 (m, 1H), 4.12 (q, *J* = 7.0 Hz, 2H), 3.25 - 3.18 (m, 2H), 2.33 - 2.28 (m, 1H), 1.86 - 1.76 (m, 3H), 1.44 (t, *J* = 7.0 Hz, 3H); ^{13}C NMR (100 MHz, $CDCl_3$) (δ, ppm) 171.4, 166.1, 160.7, 140.2, 139.4, 135.0, 130.5, 130.4, 129.2, 128.7, 128.5, 126.9, 126.5, 124.2, 116.6, 84.6, 67.2, 51.0, 41.9, 26.9, 21.1, 14.6; HRMS (TOF MS

ESI$^+$) calculated for $C_{26}H_{26}N_2O_4Na$ [M+Na]$^+$: 453.1785, found 453.1787.

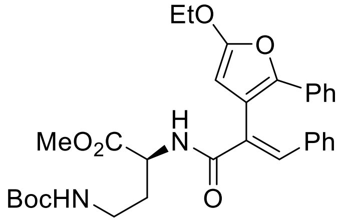

Methyl (*S,E*)-4-((*tert*-butoxycarbonyl)amino)-2-(2-(5-ethoxy-2-phenylfuran-3-yl)-3-phenylacrylamido)butanoate (45). Yellow oil. 42.2 mg, 77% yield; ^{1}H NMR (500 MHz, $CDCl_3$) (δ, ppm) 7.95 (s, 1H), 7.54 (d, *J* = 7.8 Hz, 2H), 7.42 - 7.40 (m, 2H), 7.29 - 7.25 (comp, 5H), 7.17 - 7.14 (m, 1H), 6.74 (d, *J* = 7.7 Hz, 1H), 5.10 (s, 2H), 4.70 - 4.65 (m, 1H), 4.14 (q, *J* = 7.0 Hz, 2H), 3.59 (s, 3H), 3.12 - 3.09 (m, 1H), 2.60 - 2.50 (m, 1H), 1.95 - 1.89 (m, 2H), 1.47 - 1.43 (comp, 12H); ^{13}C NMR (126 MHz, $CDCl_3$) (δ, ppm) 172.6, 166.2, 160.9, 156.0, 140.2, 140.1, 134.7, 130.5, 130.4, 129.4, 128.8, 128.5, 127.1, 126.0, 124.2, 116.3, 84.3, 79.3, 67.1, 52.6, 50.1, 36.2, 33.2, 28.5, 14.6; HRMS (TOF MS ESI$^+$) calculated for $C_{31}H_{36}N_2O_7Na$ [M+Na]$^+$: 571.2415, found 571.2418.

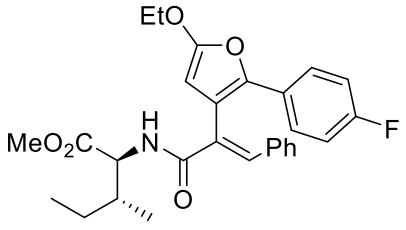

Methyl ((*E*)-2-(5-ethoxy-2-(4-fluorophenyl)furan-3-yl)-3-phenylacryloyl)-*L*-alloisoleucinate (46). Yellow oil. 39.8 mg, 83% yield; ^{1}H NMR (500 MHz, $CDCl_3$) (δ, ppm) 7.97 (s, 1H), 7.53 - 7.50 (m, 2H), 7.39 - 7.37 (m, 2H), 7.25 - 7.23 (comp, 3H), 6.98 - 6.94 (m, 2H), 6.50 (d, *J* = 8.6 Hz, 1H), 5.11 (s, 1H), 4.60 (dd, *J* = 8.6, 5.1 Hz, 1H), 4.17 - 4.11 (m, 2H), 3.62 (s, 3H), 1.76 - 1.70 (m, 1H), 1.45 (t, *J* = 7.0 Hz, 3H), 1.22 - 1.14 (m, 1H), 0.86 - 0.80 (m, 1H), 0.75 (t, *J* = 7.2 Hz, 3H), 0.66 (d, *J* = 6.8 Hz, 3H); ^{13}C NMR (125 MHz, $CDCl_3$) (δ, ppm) 172.1, 165.4, 161.8 (d, *J* = 247.5 Hz), 160.8, 140.1, 139.4, 134.7, 130.3, 129.4, 128.5, 126.6 (d, *J* = 3.2 Hz), 126.0 (d, *J* =

8.0 Hz), 125.9, 116.0, 115.8 (d, *J* = 21.9 Hz), 84.3, 67.2, 57.0, 52.0, 37.9, 24.9, 15.3, 14.5, 11.4; ^{19}F NMR (376 MHz, $CDCl_3$) (δ, ppm) -114.31; HRMS (TOF MS ESI$^+$) calculated for $C_{28}H_{30}FNO_5Na$ [M+Na]$^+$: 502.2000, found 502.2003.

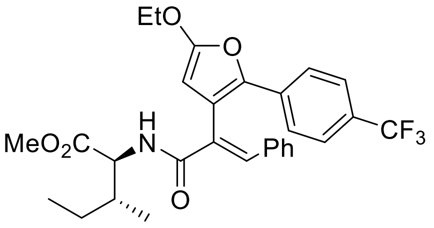

Methyl ((*E*)-2-(5-ethoxy-2-(4-(trifluoromethyl)phenyl)furan-3-yl)-3-phenyl acryloyl)-*L*-alloisoleucinate (47). Yellow oil. 43.4 mg, 82% yield; ^{1}H NMR (500 MHz, $CDCl_3$) (δ, ppm) 7.97 (s, 1H), 7.53 - 7.52 (m, 2H), 7.49 - 7.45 (comp, 4H), 7.29 - 7.26 (m, 2H), 7.18 - 7.15 (m, 1H), 6.56 (d, *J* = 8.6 Hz, 1H), 5.08 (s, 1H), 4.60 (dd, *J* = 8.6, 5.0 Hz, 1H), 4.18 - 4.12 (m, 2H), 3.61 (s, 3H), 1.76 - 1.69 (m, 1H), 1.46 (t, *J* = 7.1 Hz, 3H), 1.19 - 1.14 (m, 1H), 0.83 - 0.77 (m, 1H), 0.73 (t, *J* = 7.1 Hz, 3H), 0.64 (d, *J* = 6.7 Hz, 3H); ^{13}C NMR (125 MHz, $CDCl_3$) (δ, ppm) 172.0, 165.0, 161.0, 140.5, 138.37, 138.36, 138.0, 130.6 (q, *J* = 32.5 Hz), 130.3, 130.0, 128.9, 127.3, 125.4 (q, *J* = 3.6 Hz), 124.2, 124.0 (q, *J* = 272.1 Hz), 115.7, 84.1, 67.3, 57.1, 52.1, 37.9, 24.9, 15.3, 14.5, 11.4; ^{19}F NMR (376 MHz, $CDCl_3$) (δ, ppm) -62.90; HRMS (TOF MS ESI$^+$) calculated for $C_{29}H_{30}F_3NO_5Na$ [M+Na]$^+$: 552.1968, found 552.1970.

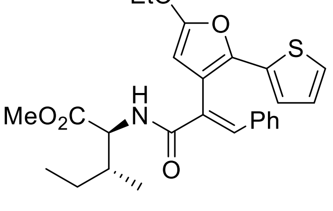

Methyl ((*E*)-2-(5-ethoxy-2-(thiophen-2-yl)furan-3-yl)-3-phenylacryloyl)-*L*-alloisoleucinate (48). Yellow oil. 33.2 mg, 71% yield; ^{1}H NMR (500 MHz, $CDCl_3$) (δ, ppm) 7.99 (s, 1H), 7.38 - 7.36 (m, 2H), 7.25 - 7.23 (comp, 3H), 7.11 - 7.09 (m, 2H), 6.92 - 6.90 (m, 1H), 6.47 (d, *J* = 8.6 Hz, 1H), 5.14 (s, 1H), 4.62 (dd, *J* = 8.6, 5.1 Hz, 1H), 4.18 - 4.12 (m, 2H), 3.63 (s, 3H), 1.80 - 1.74 (m, 1H), 1.46 (t, *J* = 7.1 Hz, 3H),

1.23 - 1.20 (m, 1H), 0.95 - 0.91 (m, 1H), 0.78 (t, *J* = 7.3 Hz, 3H), 0.70 (d, *J* = 6.9 Hz, 3H); ^{13}C NMR (125 MHz, $CDCl_3$) (δ, ppm) 172.3, 165.5, 160.5, 141.0, 137.2, 134.9, 132.0, 130.4, 129.4, 128.6, 127.6, 125.3, 124.4, 122.9, 115.4, 84.1, 67.4, 57.1, 52.1, 38.0, 25.1, 15.4, 14.6, 11.5; HRMS (TOF MS ESI$^+$) calculated for $C_{26}H_{29}NO_5SNa$ [M+Na]$^+$: 490.1659, found 490.1660.

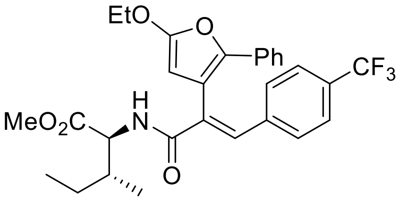

Methyl ((*E*)-2-(5-ethoxy-2-phenylfuran-3-yl)-3-(4-(trifluoromethyl)phenyl) acryloyl)-*L*-alloisoleucinate (49). Yellow oil. 45.0 mg, 85% yield; ^{1}H NMR (500 MHz, $CDCl_3$) (δ, ppm) 8.00 (s, 1H), 7.66 - 7.64 (m, 2H), 7.52 - 7.50 (m, 2H), 7.38 - 7.36 (m, 2H), 7.25 - 7.23 (comp, 3H), 6.44 (d, *J* = 8.6 Hz, 1H), 5.16 (s, 1H), 4.60 (dd, *J* = 8.6, 5.1 Hz, 1H), 4.20 - 4.14 (m, 2H), 3.61 (s, 3H), 1.72 - 1.68 (m, 1H), 1.47 (t, *J* = 7.1 Hz, 3H), 1.19 - 1.15 (m, 1H), 0.81 - 0.78 (m, 1H), 0.73 (t, *J* = 6.9 Hz, 3H), 0.64 (d, *J* = 6.5 Hz, 3H); ^{13}C NMR (125 MHz, $CDCl_3$) (δ, ppm) 172.2, 165.2, 161.5, 140.4, 138.7, 134.6, 133.4, 130.4, 129.6, 128.6, 128.5 (q, *J* = 32.6 Hz), 125.8 (q, *J* = 3.7 Hz), 125.7, 124.2 (q, *J* = 271.7 Hz), 124.0, 119.1, 84.9, 67.4, 57.0, 52.1, 38.0, 25.0, 15.3, 14.5, 11.3; ^{19}F NMR (376 MHz, $CDCl_3$) (δ, ppm) -62.67. HRMS (TOF MS ESI$^+$) calculated for $C_{29}H_{30}F_3NO_5Na$ [M+Na]$^+$: 552.1968, found 552.1964.

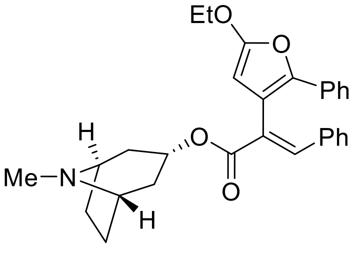

(1*R*,3*r*,5*S*)-8-Methyl-8-azabicyclo[3.2.1]octan-3-yl (*E*)-2-(5-ethoxy-2-phenylfuran -3-yl)-3-phenylacrylate (50). Yellow oil. 38.9 mg, 85% yield; ^{1}H NMR (500 MHz, $CDCl_3$) (δ, ppm) 7.98 (s, 1H), 7.50 - 7.47 (comp, 4H), 7.31 - 7.26 (comp, 5H), 7.15 (t, *J* = 7.4 Hz, 1H), 5.14 (t, *J* = 4.0 Hz, 1H), 5.06 (s, 1H), 4.12 (q, *J* = 7.0 Hz, 2H), 3.55

(s, 2H), 2.83 (s, 2H), 2.63 (s, 3H), 1.89 (s, 2H), 1.76 (d, *J* = 6.4 Hz, 2H), 1.62 (d, *J* = 13.5 Hz, 2H), 1.44 (t, *J* = 7.0 Hz, 3H); ^{13}C NMR (125 MHz, $CDCl_3$) (δ, ppm) 166.2, 160.3, 144.1, 139.8, 134.1, 131.0, 130.8, 130.2, 128.9, 128.7, 126.9, 124.1, 123.6, 116.9, 84.6, 67.3, 65.2, 61.9, 38.8, 34.3, 29.8, 24.0, 14.6; HRMS (TOF MS ESI$^+$) calculated for $C_{29}H_{32}NO_4$ [M+H]$^+$: 458.2326, found 458.2327.

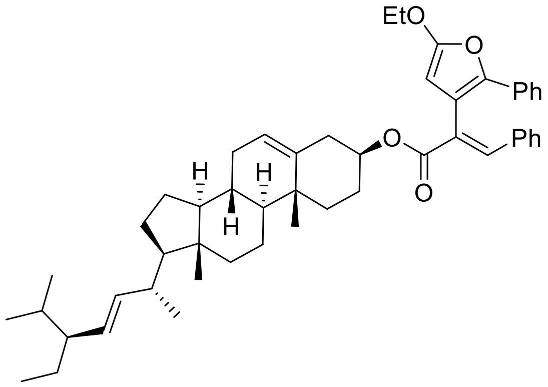

(3*S*,8*S*,9*S*,10*R*,13*R*,14*S*,17*R*)-17-((2*R*,5*S*,*E*)-5-Ethyl-6-methylhept-3-en-2-yl)-10,13-dimethyl-2,3,4,7,8,9,10,11,12,13,14,15,16,17-tetradecahydro-1*H*-cyclopenta[*a*]phenanthren-3-yl (*E*)-2-(5-ethoxy-2-phenylfuran-3-yl)-3-phenylacrylate (51). Colorless oil. 65.6 mg, 90% yield; ^{1}H NMR (500 MHz, $CDCl_3$) (δ, ppm) 7.85 (s, 1H), 7.49 - 7.47 (m, 2H), 7.44 - 7.42 (m, 2H), 7.25 - 7.23 (comp, 5H), 7.14 - 7.11 (m, 1H), 5.27 (d, *J* = 4.8 Hz, 1H), 5.15 (dd, *J* = 15.1, 8.6 Hz, 1H), 5.03 - 4.99 (m, 2H), 4.60 - 4.54 (m, 1H), 4.08 (q, *J* = 7.0 Hz, 2H), 2.07 - 1.92 (comp, 5H), 1.78 - 1.74 (m, 1H), 1.72 - 1.64 (m, 2H), 1.60 (s, 1H), 1.55 - 1.51 (comp, 3H), 1.47 - 1.45 (m, 1H), 1.43 - 1.37 (comp, 6H), 1.35 - 1.33 (m, 1H), 1.29 - 1.24 (m, 2H), 1.19 - 1.13 (comp, 3H), 1.08 - 1.01 (comp, 6H), 0.92 (s, 3H), 0.84 (d, *J* = 6.4 Hz, 3H), 0.82 (s, 1H), 0.80 - 0.79 (comp, 5H), 0.68 (s, 3H); ^{13}C NMR (125 MHz, $CDCl_3$) (δ, ppm) 166.8, 159.9, 141.8, 140.6, 139.9, 138.5, 134.8, 131.5, 130.6, 129.5, 129.4, 128.5, 128.4, 126.5, 125.8, 124.4, 122.5, 116.8, 84.9, 75.1, 67.0, 56.9, 56.1, 51.4, 50.1, 42.3, 40.6, 39.8, 37.8, 37.1, 36.7, 32.01, 31.97, 29.0, 27.5, 25.5, 24.5, 21.4, 21.2, 21.1, 19.4, 19.1, 14.6, 12.4, 12.2; HRMS (TOF MS ESI$^+$) calculated for $C_{50}H_{65}O_4$ [M+H]$^+$: 729.4877, found 729.4878.

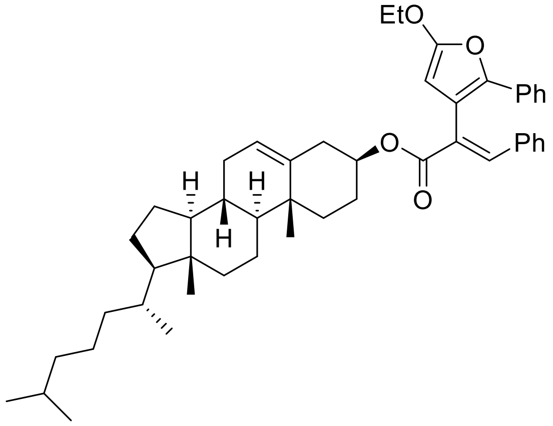

(3*S*,8*S*,9*S*,10*R*,13*R*,14*S*,17*R*)-10,13-Dimethyl-17-((*R*)-6-methylheptan-2-yl)-2,3,4,7,8,9,10,11,12,13,14,15,16,17-tetradecahydro-1*H*-cyclopenta[*a*]phenanthren-3-yl (*E*)-2-(5-ethoxy-2-phenylfuran-3-yl)-3-phenylacrylate (52). Colorless oil. 61.8 mg, 88% yield; ^{1}H NMR (500 MHz, $CDCl_3$) (δ, ppm) 7.85 (s, 1H), 7.48 - 7.47 (m, 2H), 7.44 - 7.42 (m, 2H), 7.25 - 7.23 (comp, 5H), 7.14 - 7.11 (m, 1H), 5.27 (d, *J* = 4.8 Hz, 1H), 5.01 (s, 1H), 4.60 - 4.54 (m, 1H), 4.08 (t, *J* = 7.1 Hz, 2H), 2.07 - 2.04 (m, 2H), 2.00 - 1.91 (m, 2H), 1.85 - 1.79 (m, 1H), 1.78 - 1.74 (m, 1H), 1.66 - 1.63 (m, 1H), 1.60 (s, 1H), 1.57 - 1.49 (comp, 3H), 1.46 - 1.44 (m, 1H), 1.43 - 1.40 (comp, 4H), 1.37 - 1.32 (comp, 4H), 1.26 - 1.24 (m, 2H), 1.17 - 1.11 (comp, 4H), 1.09 - 1.05 (comp, 3H), 1.03 - 0.95 (comp, 3H), 0.92 - 0.90 (comp, 6H), 0.86 (comp, 6H), 0.66 (s, 3H); ^{13}C NMR (125 MHz, $CDCl_3$) (δ, ppm) 166.8, 159.9, 141.8, 140.6, 139.9, 134.8, 131.5, 130.6, 129.5, 128.5, 128.4, 126.5, 125.9, 124.4, 122.6, 116.8, 84.9, 75.1, 67.0, 56.8, 56.3, 50.1, 42.4, 39.9, 39.7, 37.8, 37.1, 36.7, 36.3, 35.9, 32.03, 31.98, 28.4, 28.2, 27.5, 24.4, 24.0, 23.0, 22.7, 21.1, 19.4, 18.9, 14.7, 12.0; HRMS (TOF MS ESI$^+$) calculated for $C_{48}H_{63}O_4$ $[M+H]^+$: 703.4721, found 703.4723.

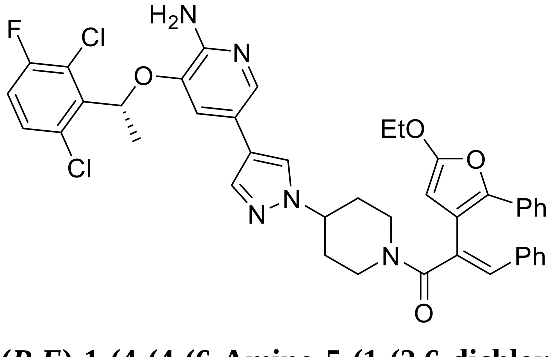

(*R*,*E*)-1-(4-(4-(6-Amino-5-(1-(2,6-dichloro-3-fluorophenyl)ethoxy)pyridin-3-yl)-

1*H*-pyrazol-1-yl)piperidin-1-yl)-2-(5-ethoxy-2-phenylfuran-3-yl)-3-phenylprop-2-en-1-one (53). Yellow oil. 52.0 mg, 68% yield; ^{1}H NMR (400 MHz, $CDCl_3$) (δ, ppm) 7.74 (d, *J* = 1.5 Hz, 1H), 7.56 (d, *J* = 7.5 Hz, 2H), 7.51 (s, 1H), 7.40 - 7.38 (m, 2H), 7.33 - 7.29 (comp, 4H), 7.25 - 7.23 (m, 2H), 7.21 - 7.18 (m, 1H), 7.14 (s, 1H), 7.05 (t, *J* = 8.4 Hz, 1H), 6.87 (d, *J* = 1.3 Hz, 1H), 6.08 (q, *J* = 6.7 Hz, 1H), 5.03 (s, 1H), 4.94 (bs, 2H), 4.33 (s, 1H), 4.19 - 4.12 (m, 1H), 4.04 (q, *J* = 7.0 Hz, 2H), 3.95 - 3.91 (m, 1H), 2.77 (s, 2H), 2.28 - 2.20 (comp, 3H), 1.91 - 1.86 (comp, 5H), 1.39 (t, *J* = 7.0 Hz, 3H); ^{13}C NMR (100 MHz, $CDCl_3$) (δ, ppm) 169.7, 160.2, 149.2, 140.9, 140.1, 137.4, 137.0, 135.9, 135.5, 135.2, 130.6, 129.8, 129.7, 128.7, 128.5, 128.3, 127.4, 125.5, 122.5, 120.1, 119.1, 117.6, 117.0, 116.8, 115.2, 84.1, 72.7, 67.1, 59.1, 31.9, 29.8, 19.0, 14.5; ^{19}F NMR (376 MHz, $CDCl_3$) (δ, ppm) -111.96; HRMS (TOF MS ESI^{+}) calculated for $C_{42}H_{39}Cl_2FN_5O_4$ $[M+H]^{+}$: 766.2358, found 766.2354.

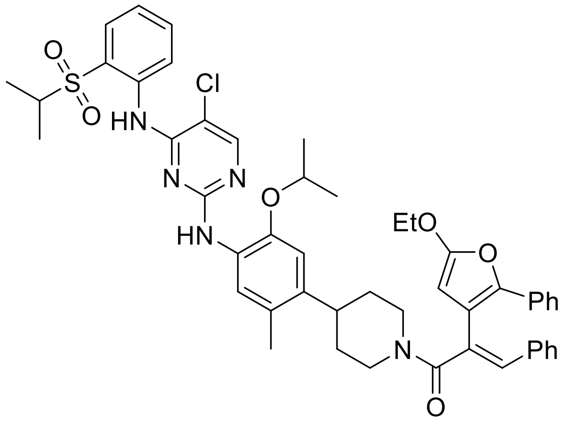

(*E*)-1-(4-(4-((5-Chloro-4-((2-(isopropylsulfonyl)phenyl)amino)pyrimidin-2-yl) amino)-5-isopropoxy-2-methylphenyl)piperidin-1-yl)-2-(5-ethoxy-2-phenylfuran-3-yl)-3-phenylprop-2-en-1-one (54). Yellow oil. 82.1 mg, 94% yield; ^{1}H NMR (400 MHz, $CDCl_3$) (δ, ppm) 9.50 (s, 1H), 8.56 (d, *J* = 8.3 Hz, 1H), 8.15 (s, 1H), 7.98 (s, 1H), 7.93 - 7.91 (m, 1H), 7.61 - 7.59 (comp, 3H), 7.55 (s, 1H), 7.41 - 7.39 (m, 2H), 7.32 - 7.28 (m, 2H), 7.26 - 7.23 (comp, 4H), 7.20 - 7.16 (m, 1H), 7.13 (s, 1H), 6.52 (s, 1H), 5.06 (s, 1H), 4.58 - 4.46 (m, 2H), 4.07 - 3.96 (m, 3H), 3.29 - 3.22 (m, 1H), 2.87 (s, 1H), 2.72 (t, *J* = 12.0 Hz, 1H), 2.53 (s, 1H), 2.10 (s, 3H), 1.89 (s, 1H), 1.42 - 1.37 (comp, 9H), 1.31 (d, *J* = 6.8 Hz, 6H), 1.26 (s, 1H), 1.18 - 1.08 (m, 2H); ^{13}C NMR

(100 MHz, $CDCl_3$) (δ, ppm) 169.7, 160.0, 157.5, 155.4, 144.7, 140.7, 138.5, 136.80, 136.79, 135.5, 134.7, 131.3, 130.6, 130.1, 129.7, 128.5, 128.3, 128.2, 128.0, 127.1, 127.0, 125.3, 125.0, 123.7, 123.2, 120.8, 117.8, 111.1, 105.9, 84.4, 71.9, 67.0, 55.5, 48.0, 43.7, 38.4, 32.6, 32.0, 22.4, 19.0, 15.4, 14.5; HRMS (TOF MS ESI^+) calculated for $C_{49}H_{52}ClN_5O_6SNa$ $[M+Na]^+$: 896.3219, found 896.3221.

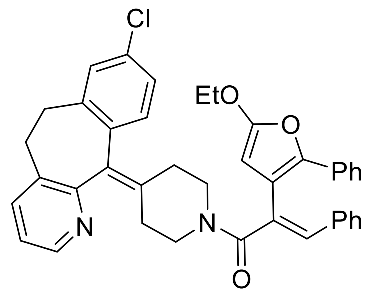

(*E*)-1-(4-(8-Chloro-5,6-dihydro-11*H*-benzo[5,6]cyclohepta[1,2-*b*]pyridin-11-ylidene)piperidin-1-yl)-2-(5-ethoxy-2-phenylfuran-3-yl)-3-phenylprop-2-en-1-one (55). Yellow oil. 59.5 mg, 95% yield; ^1H NMR (400 MHz, $CDCl_3$) (δ, ppm) 8.35 (d, *J* = 4.5 Hz, 1H), 7.54 (d, *J* = 7.8 Hz, 2H), 7.40 (d, *J* = 7.6 Hz, 1H), 7.35 - 7.33 (m, 2H), 7.25 (t, *J* = 7.4 Hz, 2H), 7.21 - 7.18 (comp, 3H), 7.15 - 7.08 (comp, 4H), 7.06 - 7.02 (m, 2H), 5.03 (s, 1H), 4.03 (q, *J* = 7.0 Hz, 2H), 3.78 - 3.62 (m, 2H), 3.35 - 3.24 (m, 2H), 3.01 (s, 2H), 2.84 - 2.72 (m, 2H), 2.21 - 2.18 (m, 1H), 2.07 - 2.06 (m, 3H), 1.38 (t, *J* = 7.0 Hz, 3H); ^{13}C NMR (100 MHz, $CDCl_3$) (δ, ppm) 169.7, 160.0, 157.0, 146.7, 140.7, 139.5, 137.6, 137.5, 137.2, 136.4, 135.5, 134.3, 133.4, 133.0, 130.6, 130.5, 129.9, 129.6, 129.1, 128.4, 128.3, 128.1, 127.1, 126.2, 125.4, 122.4, 117.5, 84.1, 67.0, 47.8, 43.8, 31.7, 31.5, 30.5, 30.4, 14.5; HRMS (TOF MS ESI^+) calculated for $C_{40}H_{36}ClN_2O_3$ $[M+H]^+$: 627.2409, found 627.2401.

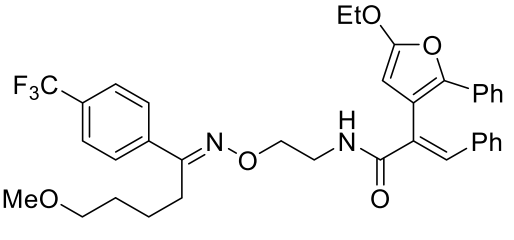

(*E*)-2-(5-Ethoxy-2-phenylfuran-3-yl)-*N*-(2-((((*E*)-5-methoxy-1-(4-(trifluoromethyl)

phenyl)pentylidene)amino)oxy)ethyl)-3-phenylacrylamide (56). Yellow oil; 57.1 mg, 90% yield; ^{1}H NMR (500 MHz, $CDCl_3$) (δ, ppm) 7.98 (s, 1H), 7.64 - 7.58 (comp, 4H), 7.49 (d, *J* = 7.8 Hz, 2H), 7.35 - 7.34 (m, 2H), 7.24 - 7.21 (comp, 5H), 7.10 (t, *J* = 7.3 Hz, 1H), 6.46 (t, *J* = 5.2 Hz, 1H), 5.02 (s, 1H), 4.09 (t, *J* = 4.9 Hz, 2H), 4.03 (q, *J* = 7.0 Hz, 2H), 3.62 (dd, *J* = 10.4, 5.1 Hz, 2H), 3.32 (t, *J* = 6.1 Hz, 2H), 3.27 (s, 3H), 2.57 - 2.54 (m, 2H), 1.56 - 1.51 (m, 2H), 1.49 - 1.44 (m, 2H), 1.40 (t, *J* = 7.0 Hz, 3H); ^{13}C NMR (125 MHz, $CDCl_3$) (δ, ppm) 165.9, 160.6, 157.8, 140.0, 139.4, 138.9, 134.9, 130.9 (q, *J* = 32.4 Hz), 130.3, 130.1, 129.2, 128.7, 128.4, 127.0, 126.5, 126.4, 125.5 (q, *J* = 3.6 Hz), 124.1 (d, *J* = 272.0 Hz), 123.9, 116.6, 84.4, 72.6, 72.2, 67.1, 58.6, 39.9, 29.5, 26.0, 23.1, 14.5; ^{19}F NMR (470 MHz, $CDCl_3$) (δ, ppm) -62.69; HRMS (TOF MS ESI$^+$) calculated for $C_{36}H_{37}F_3N_2O_5Na$ $[M+Na]^+$: 657.2547, found 657.2549.

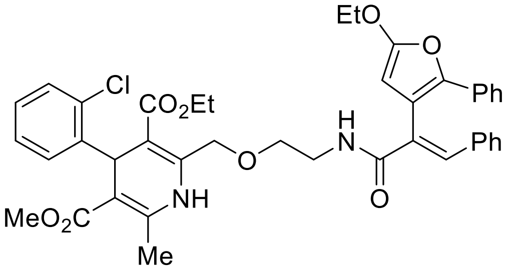

3-Ethyl 5-methyl (*E*)-4-(2-chlorophenyl)-2-((2-(2-(5-ethoxy-2-phenylfuran-3-yl)-3-phenylacrylamido)ethoxy)methyl)-6-methyl-1,4-dihydropyridine-3,5-dicarboxylate (57). Yellow oil; 66.6 mg, 92% yield; ^{1}H NMR (400 MHz, $CDCl_3$) (δ, ppm) 8.01 (s, 1H), 7.53 - 7.51 (m, 2H), 7.40 - 7.38 (comp, 3H), 7.31 (s, 1H), 7.26 - 7.21 (comp, 7H), 7.14 - 7.09 (m, 2H), 7.04 - 7.00 (m, 1H), 6.39 (t, *J* = 5.9 Hz, 1H), 5.43 (s, 1H), 5.06 (s, 1H), 4.41 (q, *J* = 15.7 Hz, 2H), 4.14 - 4.01 (comp, 4H), 3.62 (s, 4H), 3.44 - 3.40 (m, 2H), 3.27 - 3.24 (m, 1H), 2.43 (s, 3H), 1.43 (t, *J* = 7.1 Hz, 3H), 1.19 (t, *J* = 7.1 Hz, 3H); ^{13}C NMR (100 MHz, $CDCl_3$) (δ, ppm) 168.2, 167.2, 166.4, 160.8, 146.1, 145.4, 144.7, 140.1, 139.8, 134.7, 132.3, 131.5, 130.4, 130.1, 129.4, 129.2, 128.8, 128.6, 127.4, 127.2, 127.0, 126.2, 123.9, 116.8, 103.8, 101.2, 84.4, 70.7, 68.1, 67.2, 59.8, 50.8, 39.7, 37.0, 19.3, 14.6, 14.4; HRMS (TOF MS ESI$^+$) calculated for $C_{41}H_{41}ClN_2O_8Na$ $[M+Na]^+$: 747.2444, found 747.2447.

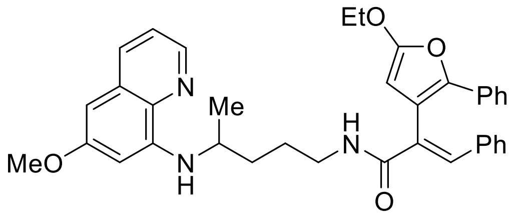

(*E*)-2-(5-Ethoxy-2-phenylfuran-3-yl)-*N*-(4-((6-methoxyquinolin-8-yl)amino) pentyl)-3-phenylacrylamide (58). Yellow oil; 52.3 mg, 91% yield; ^{1}H NMR (500 MHz, $CDCl_3$) (δ, ppm) 8.53 - 8.52 (m, 1H), 7.99 (s, 1H), 7.91 - 7.89 (m, 1H), 7.54 (d, *J* = 7.6 Hz, 2H), 7.38 (dd, *J* = 6.4, 2.8 Hz, 2H), 7.29 (dd, *J* = 8.2, 4.2 Hz, 1H), 7.25 - 7.21 (comp, 5H), 7.10 (t, *J* = 7.4 Hz, 1H), 6.31 (d, *J* = 2.3 Hz, 1H), 6.20 (d, *J* = 2.3 Hz, 1H), 6.12 (t, *J* = 5.8 Hz, 1H), 5.86 (bs, 1H), 5.01 (s, 1H), 4.05 (q, *J* = 7.0 Hz, 2H), 3.85 (s, 3H), 3.43 (s, 1H), 3.26 (s, 2H), 1.47 - 1.33 (comp, 7H), 1.14 (d, *J* = 6.3 Hz, 3H); ^{13}C NMR (125 MHz, $CDCl_3$) (δ, ppm) 165.6, 160.7, 159.5, 145.0, 144.3, 139.9, 139.2, 135.4, 135.0, 134.8, 130.3, 130.2, 130.0, 129.1, 128.9, 128.4, 127.1, 126.5, 124.0, 121.9, 116.9, 96.7, 91.6, 84.4, 67.0, 55.3, 47.8, 40.0, 33.7, 26.2, 20.4, 14.5; HRMS (TOF MS ESI^{+}) calculated for $C_{36}H_{37}N_3O_4Na$ $[M+Na]^{+}$: 598.2676, found 598.2679.

Synthesis of fused and poly-substituted benzene derivatives

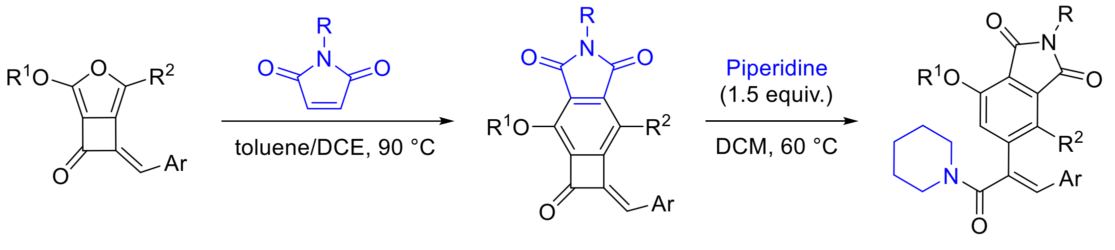

To a 10-mL oven-dried vial containing a magnetic stirring bar, **2** (0.2 mmol), maleimide (0.3 mmol, 1.5 equiv.), and mixed solvent toluene/DCE (1.0/0.1 mL) were added in sequence, and the reaction mixture was stirred for 12 h at 90 °C. Upon completion, the mixture was cooled to room temperature, and the solvent was removed under reduced pressure. The residue was purified by silica gel chromatography (Hexanes : EtOAc = 10:1 to 5:1) to afford compounds **59** – **62** in synthetically useful yields.

To a 10-mL oven-dried vial containing a magnetic stirring bar, the above obtained benzene fused cyclobutanone derivatives (0.1 mmol), piperidine (0.15 mmol, 1.5 equiv.), and DCM (2.0 mL) were added sequentially. The vial was capped, and stirring for 24 h in an oil bath at 60 °C. After completion of the reaction (monitored by TLC), the solvent was evaporated under reduced pressure, and the residue was purified by flash chromatography on silica gel (Hexanes : EtOAc = 10:1 to 1:1) to afford the ring opening products **63** – **69** in moderate to high yields.

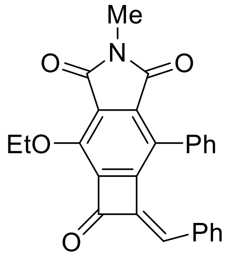

(*E*)-6-Benzylidene-4-ethoxy-2-methyl-7-phenyl-1H-cyclobuta[*f*]isoindole-1,3,5(2*H*,6*H*)-trione (59). Yellow solid, mp = 223 - 224 °C. 27.0 mg, 66% yield; ^{1}H NMR (400 MHz, $CDCl_3$) (δ, ppm) 7.19 - 7.11 (comp, 4H), 7.06 (t, *J* = 7.3 Hz, 1H), 7.00 (t, *J* = 7.6 Hz, 2H), 6.79 (t, *J* = 7.6 Hz, 2H), 6.69 (d, *J* = 7.7 Hz, 2H), 4.74 (q, *J* =

7.0 Hz, 2H), 3.09 (s, 3H), 1.59 (t, *J* = 7.0 Hz, 3H); ^{13}C NMR (125 MHz, DMSO-d$_6$) (δ, ppm) 181.2, 165.9, 164.7, 163.0, 148.2, 144.8, 142.7, 137.5, 132.9, 132.8, 128.3, 128.2, 128.1, 127.2, 126.9, 126.1, 122.5, 119.2, 69.9, 23.8, 14.8; HRMS (TOF MS ESI$^+$) calculated for $C_{26}H_{19}NO_4Na$ [M+Na]$^+$: 432.1206, found 432.1205.

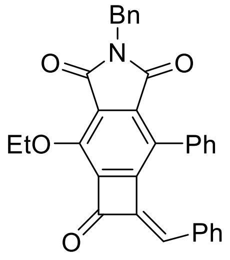

(*E*)-2-Benzyl-6-benzylidene-4-ethoxy-7-phenyl-1*H*-cyclobuta[*f*]isoindole-1,3,5(2*H*,6*H*)-trione (60). Yellow solid, mp = 208 - 209 ºC. 31.6 mg, 65% yield; ^{1}H NMR (400 MHz, CDCl$_3$) (δ, ppm) 7.40 (d, *J* = 6.8 Hz, 2H), 7.29 - 7.22 (comp, 3H), 7.18 - 7.10 (comp, 4H), 7.05 (t, *J* = 7.5 Hz, 1H), 6.99 (t, *J* = 7.7 Hz, 2H), 6.78 (t, *J* = 7.7 Hz, 2H), 6.67 (d, *J* = 7.5 Hz, 2H), 4.75 - 4.70 (comp, 4H), 1.57 (t, *J* = 6.9 Hz, 3H); ^{13}C NMR (100 MHz, CDCl$_3$) (δ, ppm) 181.8, 166.2, 165.3, 164.4, 149.4, 145.5, 143.2, 137.3, 136.3, 133.4, 132.7, 129.9, 129.1, 128.9, 128.8, 128.5, 128.2, 128.0, 127.5, 127.4, 127.3, 123.8, 119.4, 71.0, 41.9, 15.1; HRMS (TOF MS ESI$^+$) calculated for $C_{32}H_{23}NO_4Na$ [M+Na]$^+$: 508.1519, found 508.1516.

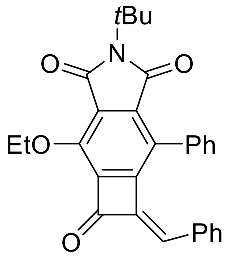

(*E*)-6-Benzylidene-2-(*tert*-butyl)-4-ethoxy-7-phenyl-1*H*-cyclobuta[*f*]isoindole-1,3,5(2*H*,6*H*)-trione (61). Yellow solid, mp = 209 - 210 ºC. 27.5 mg, 61% yield; ^{1}H NMR (500 MHz, CDCl$_3$) (δ, ppm) 7.17 - 7.14 (m, 1H), 7.12 - 7.11 (comp, 3H), 7.05 (t, *J* = 7.4 Hz, 1H), 7.01 - 6.98 (m, 2H), 6.78 (t, *J* = 7.7 Hz, 2H), 6.66 (d, *J* = 7.5 Hz, 2H), 4.72 (q, *J* = 7.0 Hz, 2H), 1.63 (s, 9H), 1.58 (t, *J* = 7.0 Hz, 3H); ^{13}C NMR (125 MHz,

$CDCl_3$) (δ, ppm) 182.1, 167.5, 166.9, 163.7, 149.1, 145.0, 143.3, 137.3, 133.5, 133.1, 129.9, 128.7, 128.4, 128.2, 127.5, 127.4, 126.6, 123.5, 119.5, 71.0, 58.5, 29.2, 15.1; HRMS (TOF MS ESI$^+$) calculated for $C_{29}H_{25}NO_4Na$ $[M+Na]^+$: 474.1676, found 474.1680.

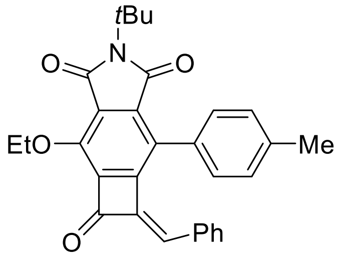

(*E*)-6-Benzylidene-2-(*tert*-butyl)-4-ethoxy-7-(*p*-tolyl)-1*H*-cyclobuta[*f*]isoindole-1,3,5(2*H*,6*H*)-trione (62). Yellow solid, mp = 205 - 206 °C. 29.3 mg, 63% yield; ^{1}H NMR (500 MHz, $CDCl_3$) (δ, ppm) 7.11 (s, 1H), 7.05 (t, *J* = 7.4 Hz, 1H), 6.99 (d, *J* = 8.0 Hz, 2H), 6.81 - 6.77 (comp, 4H), 6.67 (d, *J* = 7.5 Hz, 2H), 4.71 (q, *J* = 7.0 Hz, 2H), 2.26 (s, 3H), 1.63 (s, 9H), 1.58 (t, *J* = 7.0 Hz, 3H); ^{13}C NMR (125 MHz, $CDCl_3$) (δ, ppm) 182.1, 167.6, 167.0, 164.0, 149.0, 145.0, 143.6, 138.6, 137.1, 133.7, 130.0, 129.7, 128.21, 128.19, 128.0, 127.4, 126.8, 123.2, 119.5, 70.9, 58.5, 29.2, 21.5, 15.1; HRMS (TOF MS ESI$^+$) calculated for $C_{30}H_{27}NO_4Na$ $[M+Na]^+$: 488.1832, found 488.1831.

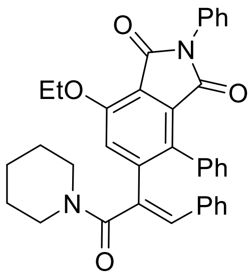

(*E*)-7-Ethoxy-5-(3-oxo-1-phenyl-3-(piperidin-1-yl)prop-1-en-2-yl)-2,4-diphenylisoindoline-1,3-dione (63). Colorless oil. 37.3 mg, 67% yield; ^{1}H NMR (400 MHz, $CDCl_3$) (δ, ppm) 7.62 (s, 1H), 7.41 - 7.34 (comp, 9H), 7.31 - 7.26 (comp, 4H), 7.17 - 7.15 (m, 2H), 6.61 (s, 1H), 4.38 (q, *J* = 6.9 Hz, 2H), 3.50 - 3.22 (m, 2H), 2.58 - 2.52 (m, 2H), 1.68 - 1.62 (m, 2H), 1.56 (t, *J* = 6.9 Hz, 3H), 1.35 - 1.26 (comp,

4H); ^{13}C NMR (100 MHz, $CDCl_3$) (δ, ppm) 167.2, 166.3, 165.2, 155.6, 147.3, 135.6, 135.4, 135.1, 134.0, 131.83, 131.75, 131.1, 130.3, 128.83, 128.76, 128.6, 128.5, 128.1, 128.0, 127.8, 126.8, 120.7, 116.6, 65.4, 46.8, 42.2, 25.0, 24.6, 24.2, 14.8; HRMS (TOF MS ESI^+) calculated for $C_{36}H_{32}N_2O_4Na$ $[M+Na]^+$: 579.2254, found 579.2253.

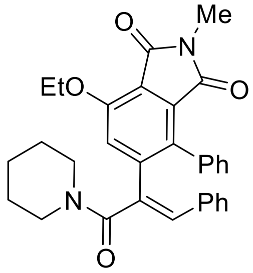

(*E*)-7-Ethoxy-2-methyl-5-(3-oxo-1-phenyl-3-(piperidin-1-yl)prop-1-en-2-yl)-4-phenylisoindoline-1,3-dione (64). Colorless oil. 29.7 mg, 60% yield; ^1H NMR (500 MHz, $CDCl_3$) (δ, ppm) 7.53 (s, 1H), 7.41 - 7.33 (comp, 5H), 7.28 - 7.23 (comp, 3H), 7.16 - 7.15 (m, 2H), 6.58 (s, 1H), 4.35 (q, *J* = 7.0 Hz, 2H), 3.62 - 3.50 (m, 1H), 3.21 - 3.02 (comp, 4H), 2.71 - 2.42 (m, 2H), 1.75 - 1.67 (m, 1H), 1.55 (t, *J* = 7.0 Hz, 3H), 1.40 - 1.21 (comp, 5H); ^{13}C NMR (125 MHz, $CDCl_3$) (δ, ppm) 167.6, 167.2, 166.5, 155.0, 146.7, 135.7, 135.5, 134.9, 134.0, 131.7, 131.3, 130.3, 128.7, 128.6, 128.5, 128.1, 127.9, 120.3, 117.0, 65.3, 46.7, 42.1, 24.9, 24.6, 24.2, 23.7, 14.8; HRMS (TOF MS ESI^+) calculated for $C_{31}H_{30}N_2O_4Na$ $[M+Na]^+$: 517.2098, found 517.2098.

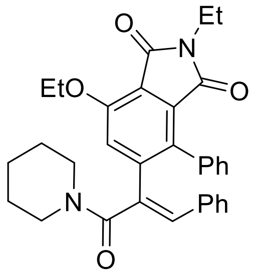

(*E*)-7-Ethoxy-2-ethyl-5-(3-oxo-1-phenyl-3-(piperidin-1-yl)prop-1-en-2-yl)-4-phenylisoindoline-1,3-dione (65). Colorless oil. 25.9 mg, 51% yield; ^1H NMR (400 MHz, $CDCl_3$) (δ, ppm) 7.53 (s, 1H), 7.43 - 7.33 (comp, 5H), 7.26 - 7.25 (comp, 3H), 7.16 - 7.15 (m, 2H), 6.58 (s, 1H), 4.34 (q, *J* = 7.0 Hz, 2H), 3.59 (q, *J* = 7.1 Hz, 2H), 3.42 - 3.11 (m, 2H), 2.71 - 2.41 (m, 2H), 1.71 - 1.63 (m, 2H), 1.55 (t, *J* = 7.0 Hz, 3H),

1.33 - 1.29 (comp, 4H), 1.17 (t, *J* = 7.2 Hz, 3H); ^{13}C NMR (100 MHz, $CDCl_3$) (δ, ppm) 167.3, 166.4, 155.1, 146.7, 135.7, 135.5, 135.0, 134.1, 131.7, 131.3, 130.4, 128.7, 128.6, 128.5, 128.1, 128.0, 120.3, 117.1, 65.3, 46.8, 42.2, 32.8, 25.0, 24.6, 24.2, 14.8, 13.9; HRMS (TOF MS ESI$^+$) calculated for $C_{32}H_{32}N_2O_4Na$ [M+Na]$^+$: 531.2254, found 531.2253.

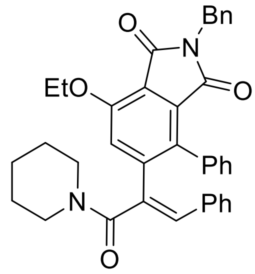

(*E*)-2-Benzyl-7-ethoxy-5-(3-oxo-1-phenyl-3-(piperidin-1-yl)prop-1-en-2-yl)-4-phenylisoindoline-1,3-dione (66). Colorless oil. 31.9 mg, 56% yield; ^{1}H NMR (500 MHz, $CDCl_3$) (δ, ppm) 7.53 (s, 1H), 7.41 - 7.34 (comp, 6H), 7.27 - 7.20 (comp, 7H), 7.15 - 7.13 (m, 2H), 6.55 (s, 1H), 4.69 (s, 2H), 4.33 (q, *J* = 7.0 Hz, 2H), 3.63 - 3.10 (m, 2H), 2.71 - 2.37 (m, 2H), 1.77 - 1.62 (m, 2H), 1.54 (t, *J* = 7.0 Hz, 3H), 1.33 - 1.30 (m, 2H), 1.27 - 1.24 (m, 2H); ^{13}C NMR (125 MHz, $CDCl_3$) (δ, ppm) 167.2, 167.0, 166.2, 155.2, 146.9, 136.7, 135.6, 135.4, 135.0, 134.0, 131.5, 131.4, 130.4, 128.9, 128.7, 128.62, 128.56, 128.5, 128.1, 128.0, 127.7, 120.3, 116.9, 65.2, 46.7, 42.2, 41.5, 25.0, 24.6, 24.2, 14.8; HRMS (TOF MS ESI$^+$) calculated for $C_{37}H_{34}N_2O_4Na$ [M+Na]$^+$: 593.2411, found 593.2412.

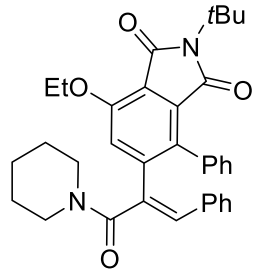

(*E*)-2-(*tert*-Butyl)-7-ethoxy-5-(3-oxo-1-phenyl-3-(piperidin-1-yl)prop-1-en-2-yl)-4-phenylisoindoline-1,3-dione (67). Colorless oil. 25.8 mg, 48% yield; ^{1}H NMR (500 MHz, $CDCl_3$) (δ, ppm) 7.51 (s, 1H), 7.42 - 7.30 (comp, 5H), 7.26 - 7.23 (comp, 3H),

7.14 - 7.13 (m, 2H), 6.53 (s, 1H), 4.32 (q, J = 7.0 Hz, 2H), 3.57 - 3.08 (m, 2H), 2.70 - 2.30 (m, 2H), 1.68 - 1.62 (m, 2H), 1.59 (s, 9H), 1.55 (t, J = 7.0 Hz, 3H), 1.37 - 1.33 (m, 2H), 1.27 - 1.25 (m, 2H); ^{13}C NMR (100 MHz, $CDCl_3$) (δ, ppm) 168.4, 168.0, 167.3, 154.8, 146.5, 136.1, 135.6, 134.9, 134.1, 131.5, 130.6, 130.4, 128.63, 128.55, 128.5, 128.0, 127.9, 119.7, 117.1, 65.1, 57.8, 46.7, 42.2, 29.3, 25.0, 24.7, 24.2, 14.8; HRMS (TOF MS ESI$^+$) calculated for $C_{34}H_{36}N_2O_4Na$ [M+Na]$^+$: 559.2567, found 559.2565.

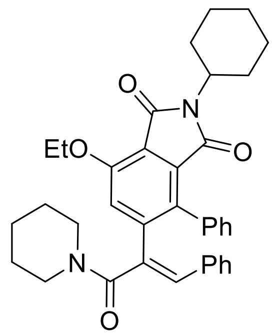

(*E*)-2-Cyclohexyl-7-ethoxy-5-(3-oxo-1-phenyl-3-(piperidin-1-yl)prop-1-en-2-yl)-4-phenylisoindoline-1,3-dione (68). Colorless oil. 36.6 mg, 65% yield; ^{1}H NMR (500 MHz, $CDCl_3$) (δ, ppm) 7.52 (s, 1H), 7.41 - 7.35 (comp, 4H), 7.28 - 7.24 (comp, 4H), 7.16 - 7.14 (m, 2H), 6.57 (s, 1H), 4.32 (q, J = 7.0 Hz, 2H), 4.00 - 3.94 (m, 1H), 3.61 - 3.14 (m, 2H), 2.70 - 2.34 (m, 2H), 2.17 - 2.10 (m, 2H), 1.79 - 1.77 (m, 3H), 1.64 - 1.59 (comp, 4H), 1.55 (t, J = 7.0 Hz, 3H), 1.38 - 1.33 (m, 2H), 1.24 - 1.15 (comp, 5H); ^{13}C NMR (125 MHz, $CDCl_3$) (δ, ppm) 167.4, 167.3, 166.7, 155.1, 146.6, 135.8, 135.5, 134.9, 134.1, 131.4, 131.1, 130.4, 128.7, 128.6, 128.5, 128.0, 127.9, 120.1, 116.9, 65.2, 50.9, 46.7, 42.2, 29.9, 26.2, 25.3, 24.9, 24.6, 24.2, 14.8; HRMS (TOF MS ESI$^+$) calculated for $C_{36}H_{38}N_2O_4Na$ [M+Na]$^+$: 585.2724, found 585.2722.

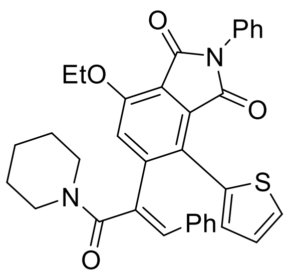

(*E*)-7-Ethoxy-5-(3-oxo-1-phenyl-3-(piperidin-1-yl)prop-1-en-2-yl)-2-phenyl-4-(thiophen-2-yl)isoindoline-1,3-dione (69). Yellow oil. 32.1 mg, 57% yield; ^{1}H NMR (500 MHz, $CDCl_3$) (δ, ppm) 7.68 (s, 1H), 7.42 - 7.39 (comp, 3H), 7.37 - 7.36 (m, 2H), 7.33 - 7.29 (comp, 3H), 7.26 - 7.24 (comp, 3H), 7.09 - 7.06 (m, 2H), 6.78 (s, 1H), 4.38 (q, J = 7.0 Hz, 2H), 3.57 - 3.24 (m, 2H), 2.76 - 2.66 (m, 2H), 1.76 - 1.63 (m, 2H), 1.56 (t, J = 7.0 Hz, 3H), 1.41 - 1.35 (m, 2H), 1.33 - 1.29 (m, 2H); ^{13}C NMR (125 MHz, $CDCl_3$) (δ, ppm) 167.1, 165.9, 165.0, 156.2, 148.7, 135.3, 135.3, 135.1, 133.6, 132.7, 131.8, 129.8, 129.5, 128.89, 128.86, 128.7, 127.9, 127.1, 126.9, 126.8, 123.5, 120.6, 116.8, 65.5, 46.8, 42.4, 25.1, 24.7, 24.2, 14.7; HRMS (TOF MS ESI$^+$) calculated for $C_{34}H_{30}N_2O_4SNa$ [M+Na]$^+$: 585.1818, found 585.1816.

Control experiments

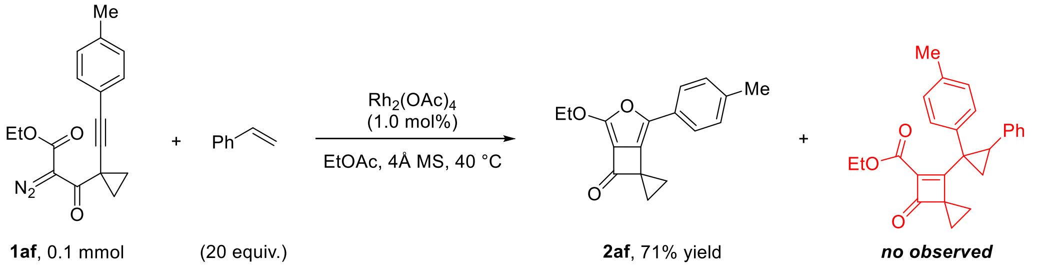

To a 10-mL oven-dried vial containing a magnetic stirring bar, styrene (208 mg, 2.0 mmol, 20 equiv.), $Rh_2(OAc)_4$ (0.5 mg, 1.0 mol%), and 4Å MS (100 mg) in ethyl acetate (1.0 mL), was added as a solution of diazo compound **1af** (29.6 mg, 0.1 mmol) in ethyl acetate (1.0 mL) *via* a syringe under argon atmosphere at 40 ˚C. After addition, the reaction mixture was stirred for additional 4.0 h under these conditions until consumption of the material (monitored by TLC). Then, the crude reaction mixture was subjected to the mass spectrometry (MS) analysis, and no formation of

the cyclopropanation product was observed. The crude product was purified by flash chromatography on silica gel (Hexanes : EtOAc = 40:1 to 15:1) to give 19.0 mg pure product **2af** in 71% yield.

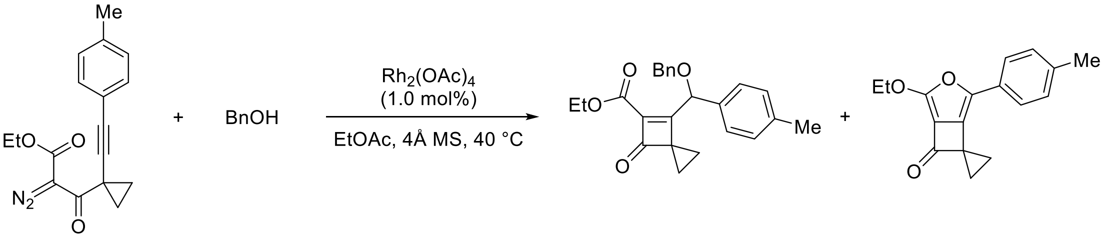

1af, 0.1 mmol (1.5 equiv.) **70,** 31% yield **2af**, 40% yield

To a 10-mL oven-dried vial containing a magnetic stirring bar, BnOH (16.2 mg, 0.15 mmol, 1.5 equiv.), $Rh_2(OAc)_4$ (0.5 mg, 1.0 mol%), and 4Å MS (100 mg) in ethyl acetate (1.0 mL), was added as a solution of diazo compound **1af** (29.6 mg, 0.1 mmol) in ethyl acetate (1.0 mL) *via* a syringe under argon atmosphere at 40 ˚C. After addition, the reaction mixture was stirred for additional 4.0 h under these conditions until consumption of the material (monitored by TLC). The crude product was purified by flash chromatography on silica gel (Hexanes : EtOAc = 40:1 to 15:1) to give 11.7 mg pure product **70** in 31% yield combined with **2af** in 40% yield. Colorless oil. ^{1}H NMR (400 MHz, $CDCl_3$) (δ, ppm) 7.34 - 7.27 (comp, 3H), 7.26 - 7.22 (m, 2H), 7.20 - 7.18 (comp, 4H), 4.65 (s, 1H), 4.56 (q, *J* = 11.6 Hz, 2H), 4.20 (q, *J* = 7.1 Hz, 2H), 2.38 (s, 3H), 1.55 - 1.50 (m, 1H), 1.45 - 1.40 (m, 1H), 1.28 - 1.23 (comp, 4H), 1.08 - 1.03 (m, 1H); ^{13}C NMR (101 MHz, $CDCl_3$) (δ, ppm) 204.5, 166.9, 148.0, 139.2, 137.7, 130.7, 129.3, 128.6, 128.4, 127.97, 127.95, 114.4, 71.2, 67.5, 61.5, 44.4, 21.5, 20.7, 19.2, 14.3; HRMS (TOF MS ESI$^+$) calculated for $C_{24}H_{25}O_4$ $[M+H]^+$: 377.1747, found 377.1745.

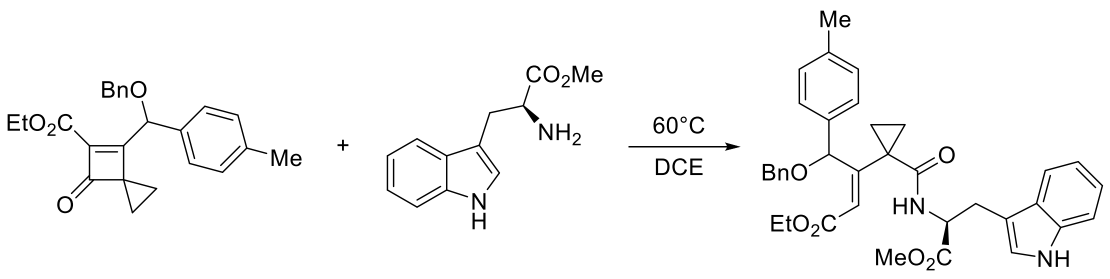

70

71, 70% yield
(CCDC: 2265899)

Synthesis of 71: To a 10-mL oven-dried vial containing a magnetic stirring bar, methyl *L*-tryptophanate (32.7 mg, 0.15 mmol) in DCE (1.0 mL), was added as a solution of cyclobutanone **70** (37.6 mg, 0.1 mmol) in DCE (1.0 mL) *via* a syringe at 60 ˚C. After addition, the reaction mixture was stirred for additional 3.0 h under these conditions until consumption of the material (monitored by TLC). The crude product was purified by flash chromatography on silica gel (Hexanes : EtOAc = 4:1 to 3:1) to give 41.6 mg pure product **71** in 70% yield. ^{1}H NMR (400 MHz, DMSO) (δ, ppm) 10.89 (s, 1H), 8.01 (d, *J* = 7.3 Hz, 1H), 7.54 (d, *J* = 7.6 Hz, 1H), 7.37 - 7.33 (comp, 3H), 7.31 - 7.26 (comp, 4H), 7.20 (s, 1H), 7.11 - 7.07 (m, 2H), 7.02 - 6.93 (comp, 4H), 4.67 - 4.61 (m, 1H), 4.34 (s, 2H), 3.96 - 3.82 (m, 2H), 3.69 (s, 3H), 3.31 - 3.27 (m, 1H), 3.18 - 3.12 (m, 1H), 3.04 - 3.00 (m, 1H), 2.28 (s, 3H), 1.28 - 1.24 (m, 1H), 1.05 (t, *J* = 7.1 Hz, 3H), 0.90 - 0.89 (m, 1H), 0.80 - 0.78 (m, 1H), 0.46 - 0.27 (m, 1H); ^{13}C NMR (101 MHz, DMSO) (δ, ppm) 173.6, 173.0, 172.3, 157.3, 138.0, 137.2, 136.3, 130.4, 128.7, 128.3, 128.2, 127.5, 126.98, 126.96, 123.6, 121.0, 118.4, 118.0, 111.7, 111.5, 109.4, 70.0, 60.5, 53.6, 52.0, 51.9, 37.2, 28.1, 26.5, 20.8, 18.0, 13.7; HRMS (TOF MS ESI^{+}) calculated for $C_{36}H_{39}N_{2}O_{6}$ [M+H]$^{+}$: 595.2803, found 595.2806.

NMR Spectra of new compounds

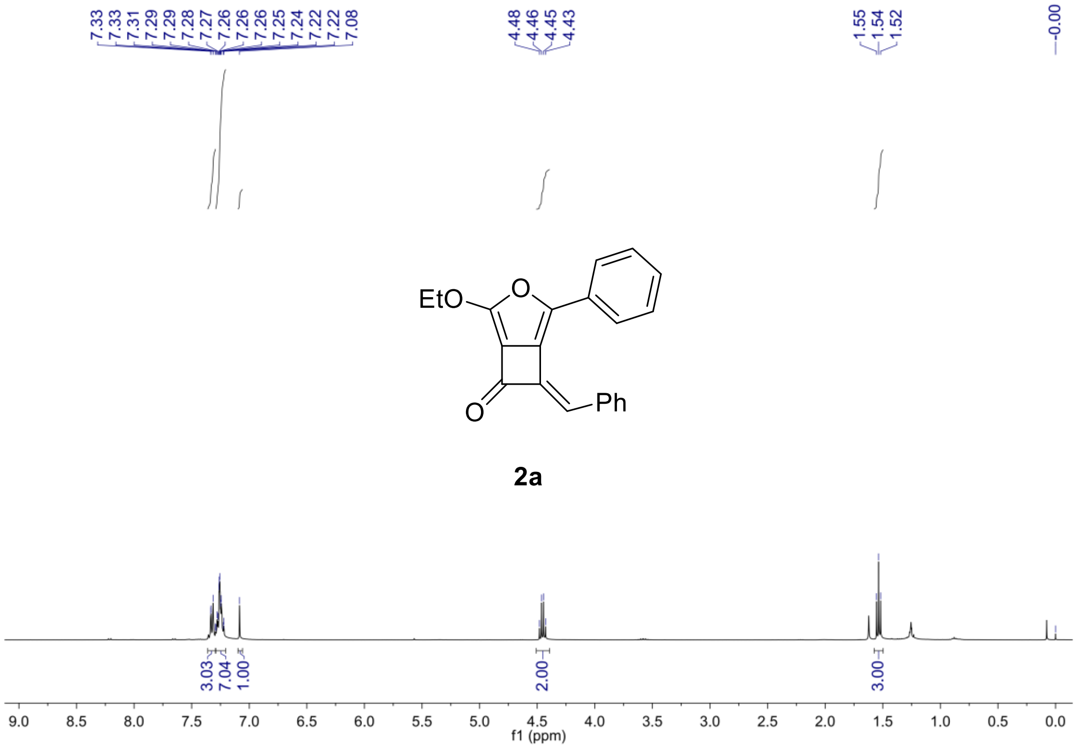

Supplementary Fig. 1 | ^{1}H NMR of compound 2a (400 MHz, at 25 °C, in $CDCl_3$).

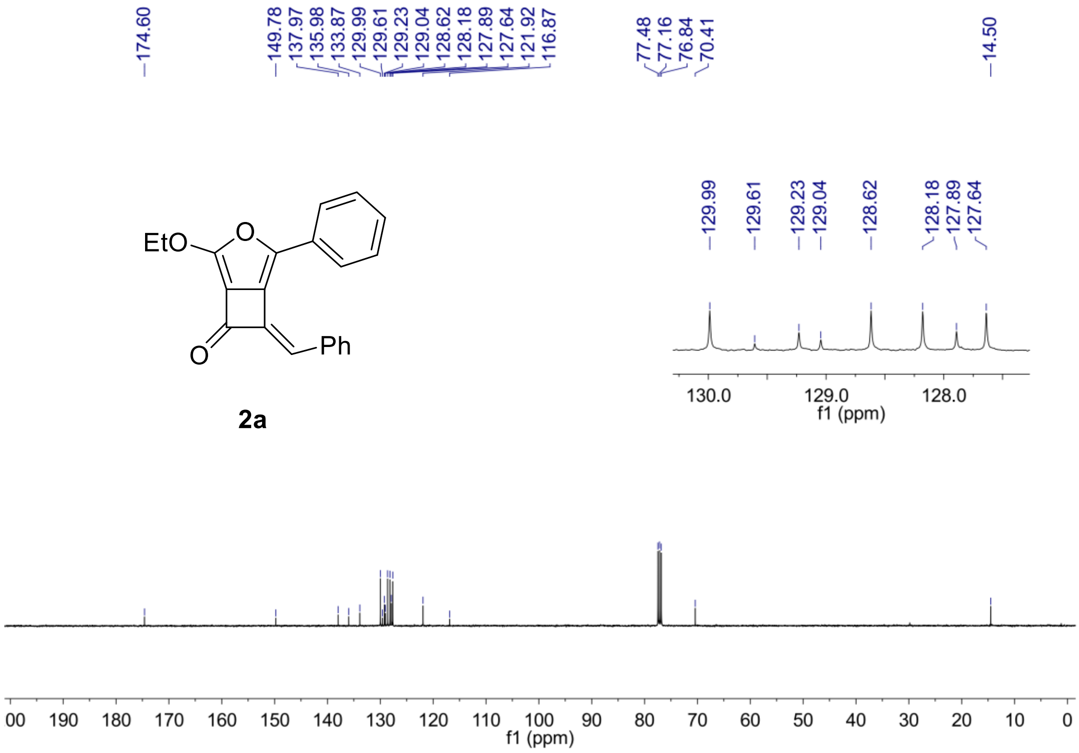

Supplementary Fig. 2 | ^{13}C NMR of compound 2a (100 MHz, at 25 °C, in $CDCl_3$).

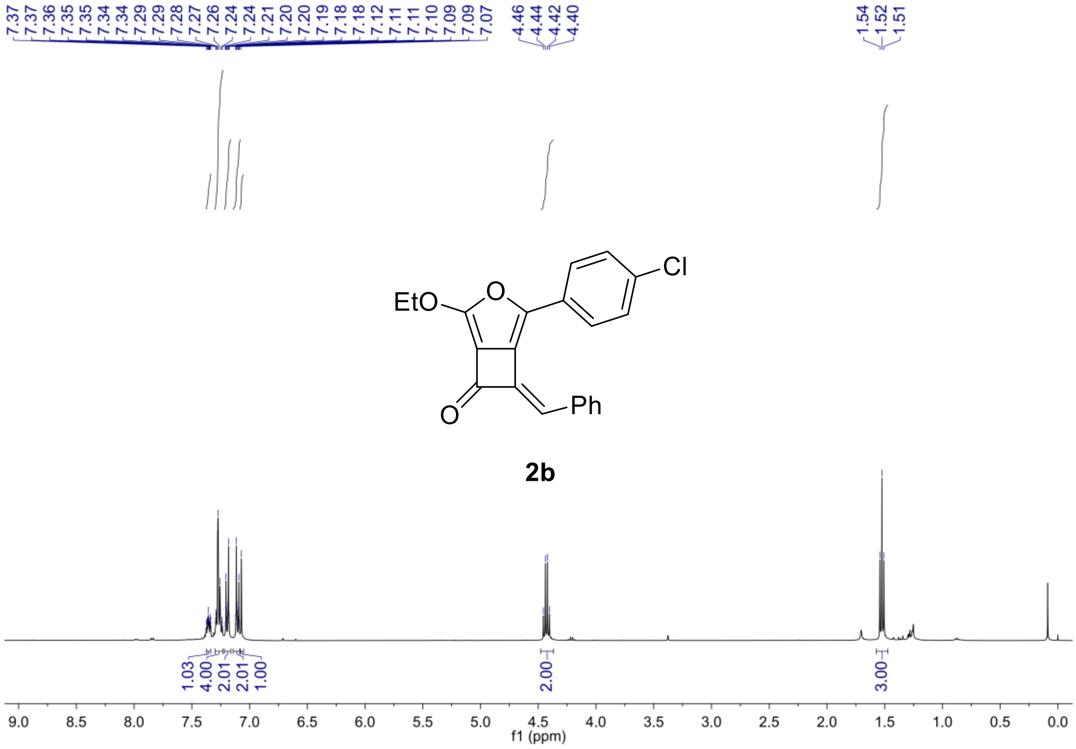

Supplementary Fig. 3 | ^{1}H NMR of compound 2b (400 MHz, at 25 °C, in $CDCl_3$).

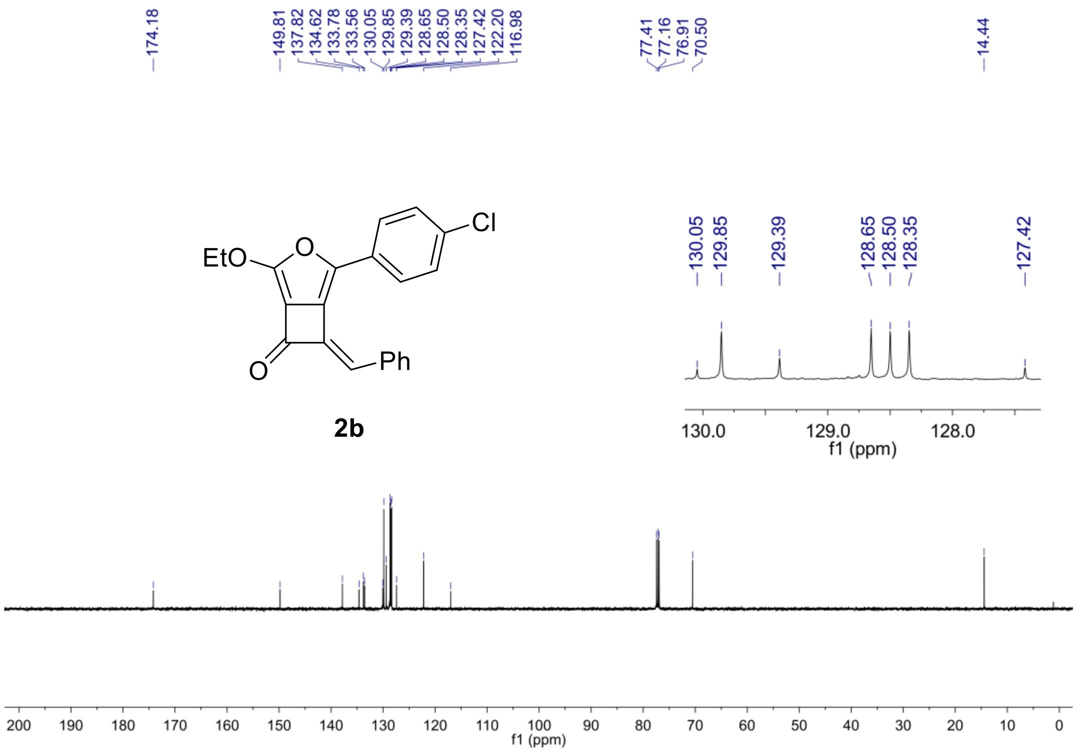

Supplementary Fig. 4 | ^{13}C NMR of compound 2b (125 MHz, at 25 °C, in $CDCl_3$).

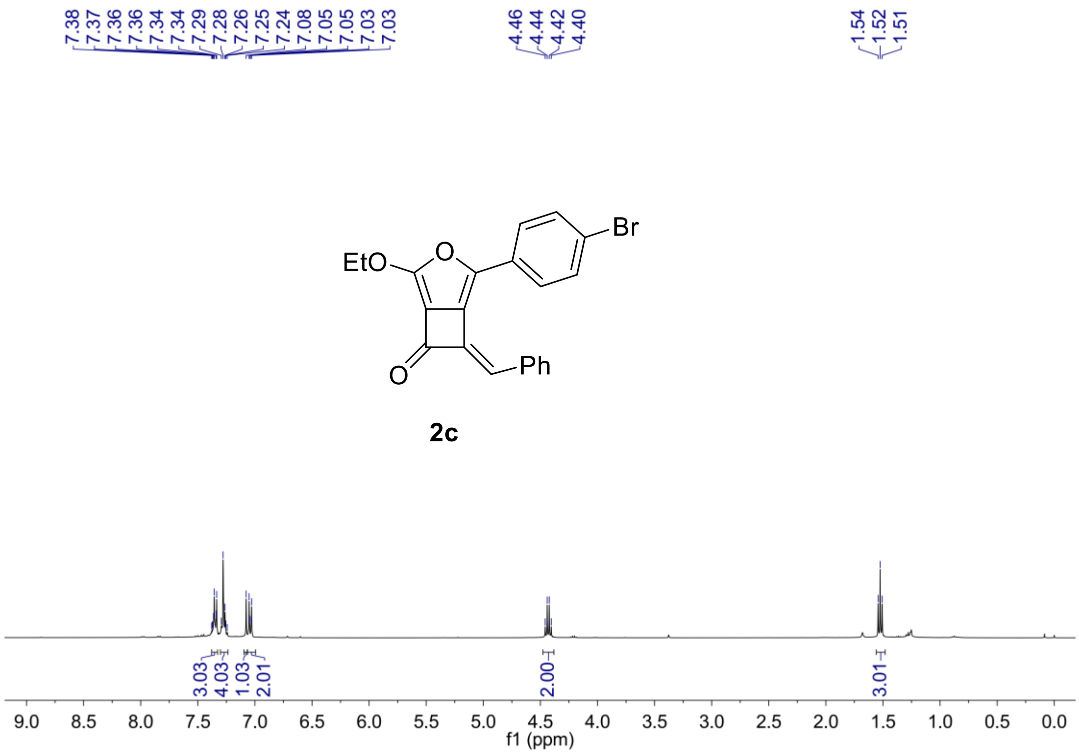

Supplementary Fig. 5 | ^{1}H NMR of compound 2c (400 MHz, at 25 °C, in $CDCl_3$).

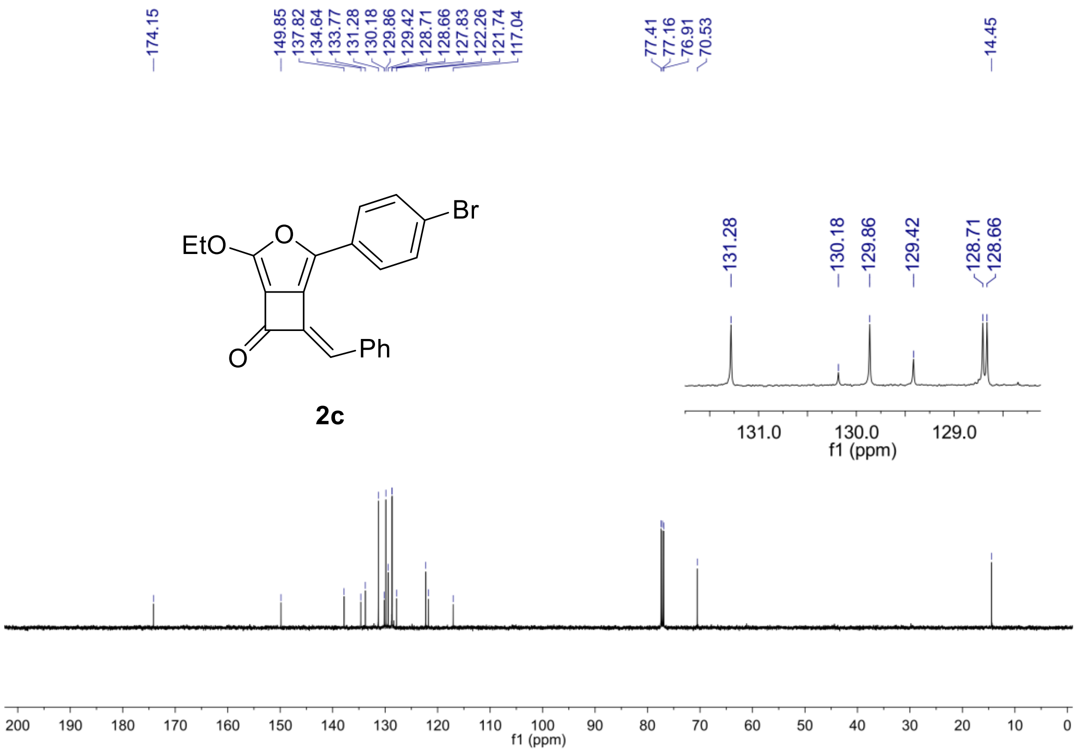

Supplementary Fig. 6 | ^{13}C NMR of compound 2c (125 MHz, at 25 °C, in $CDCl_3$).

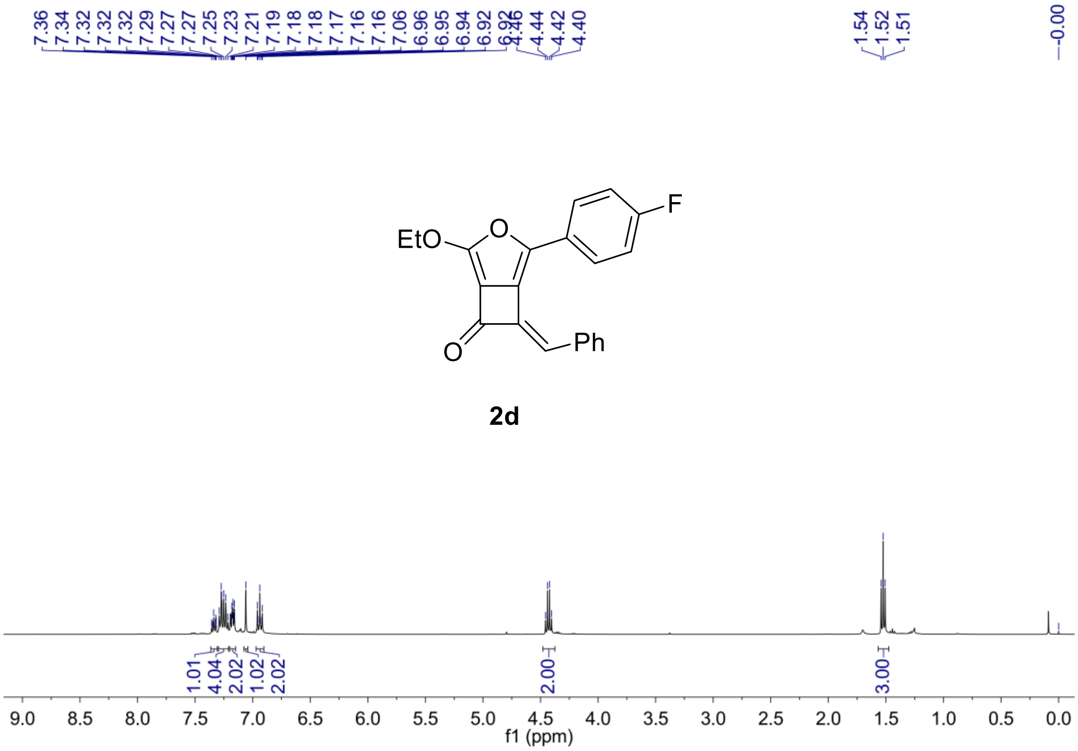

Supplementary Fig. 7 | ^{1}H NMR of compound 2d (400 MHz, at 25 ^{o}C, in $CDCl_3$).

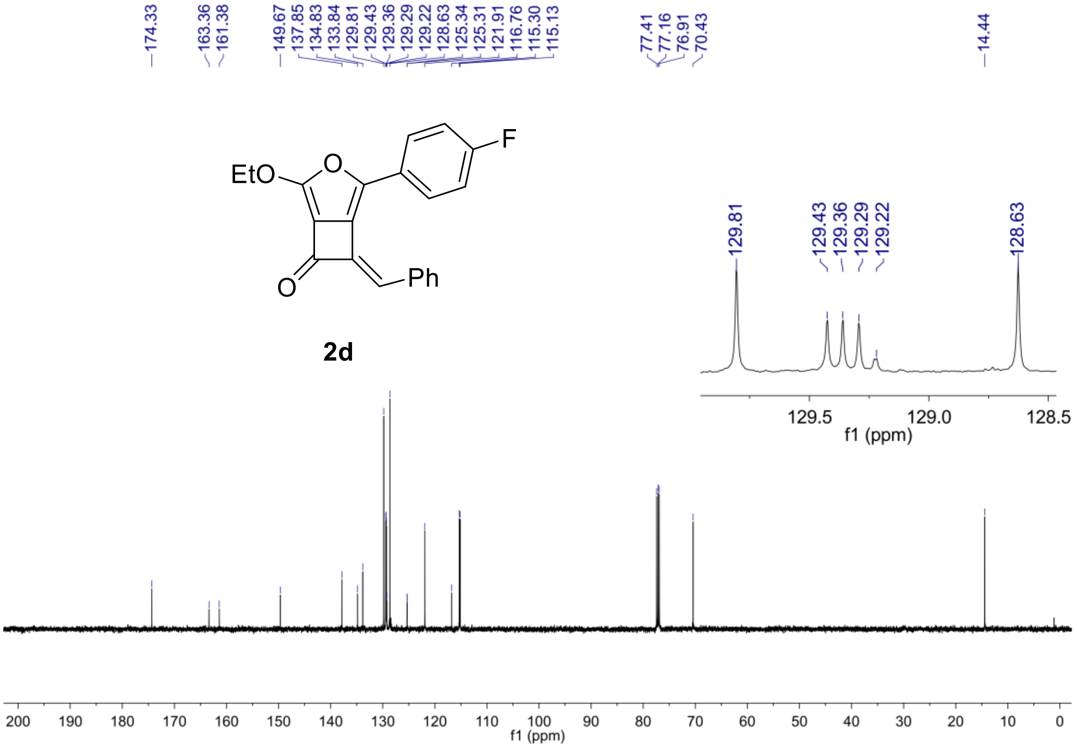

Supplementary Fig. 8 | ^{13}C NMR of compound 2d (125 MHz, at 25 ^{o}C, in $CDCl_3$).

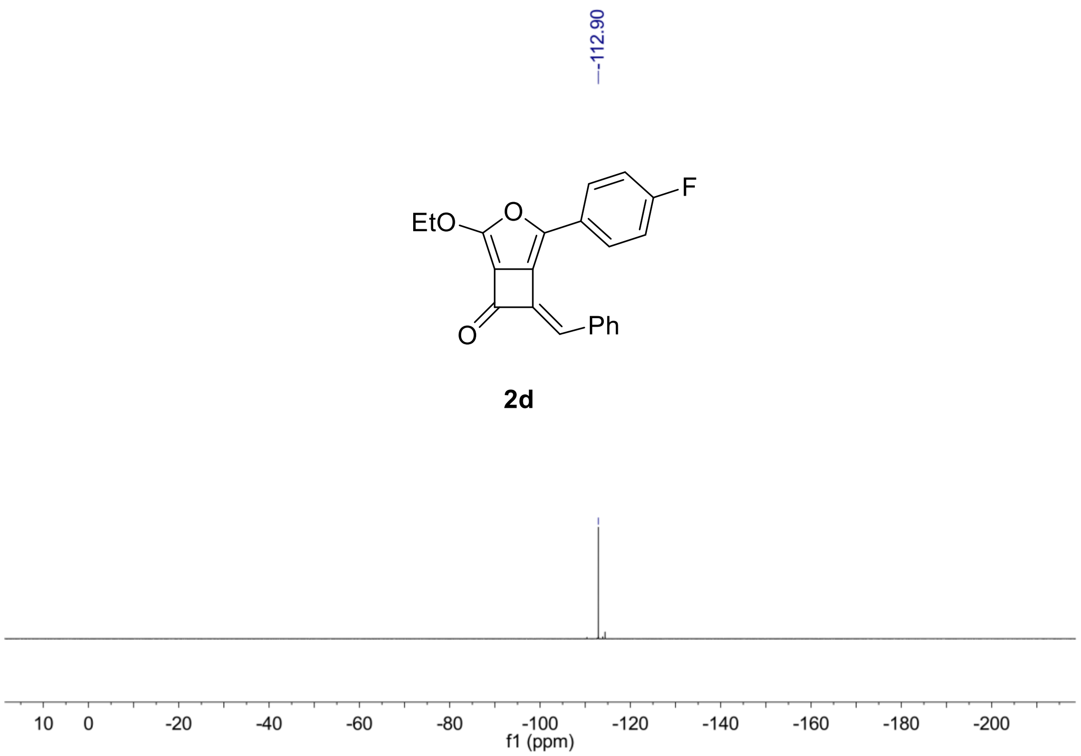

Supplementary Fig. 9 | ^{19}F NMR of compound 2d (376 MHz, at 25 °C, in $CDCl_3$).

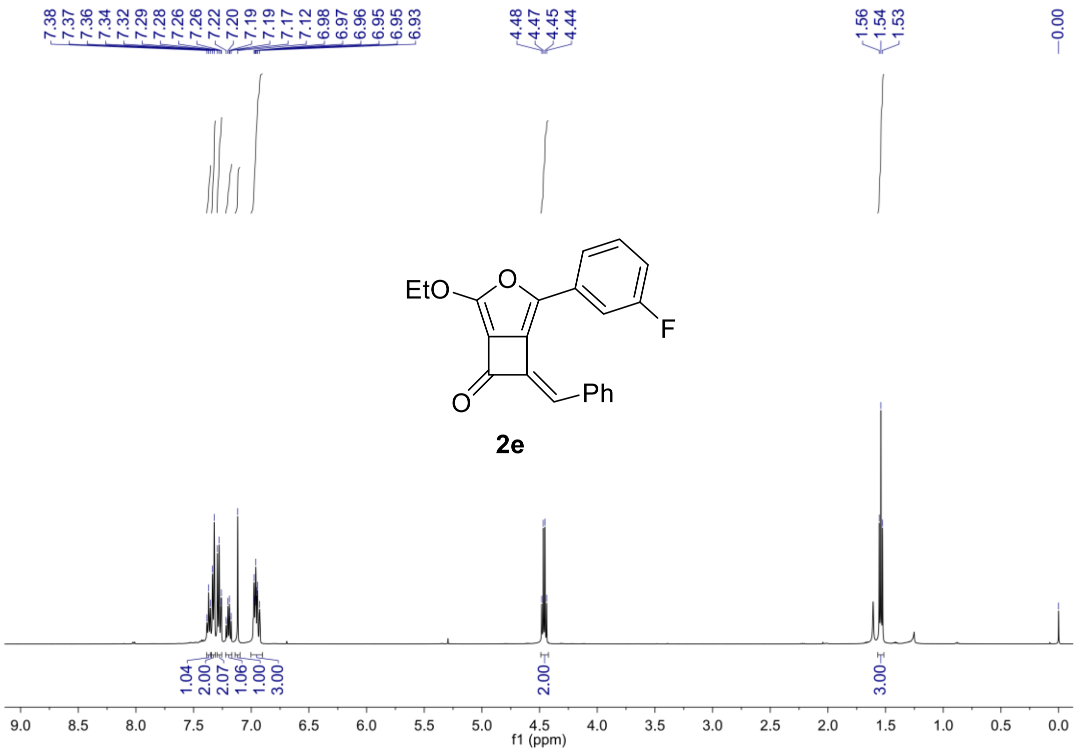

Supplementary Fig. 10 | ^{1}H NMR of compound 2e (500 MHz, at 25 °C, in $CDCl_3$).

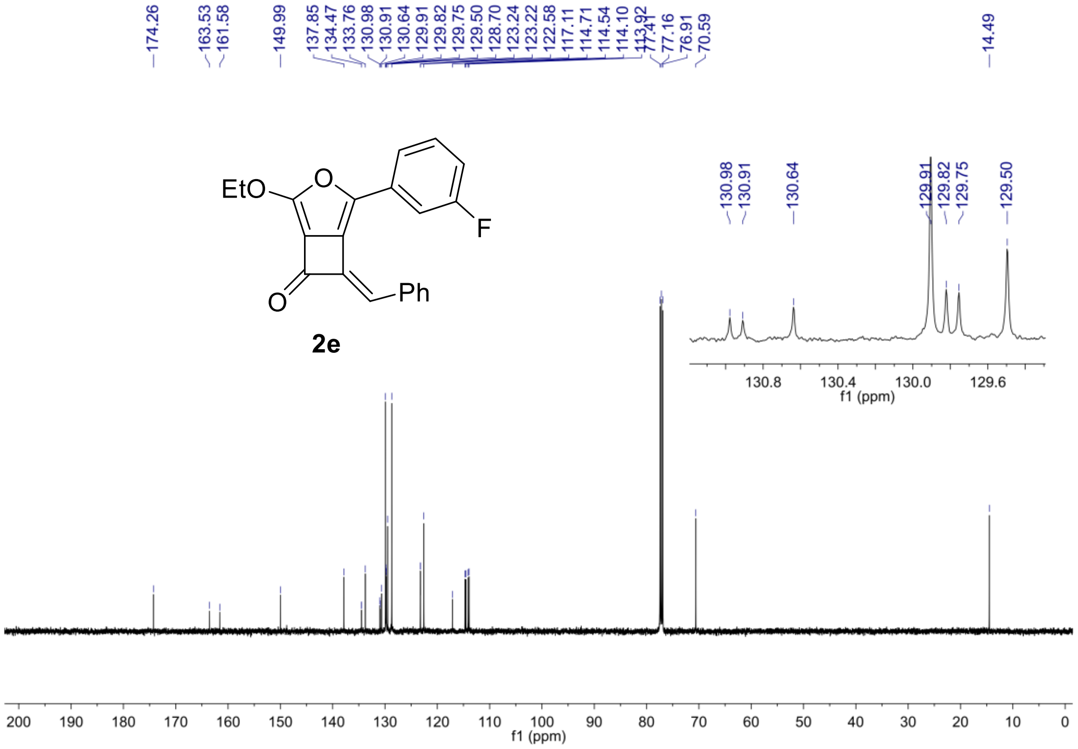

Supplementary Fig. 11 | ^{13}C NMR of compound 2e (125 MHz, at 25 ºC, in $CDCl_3$).

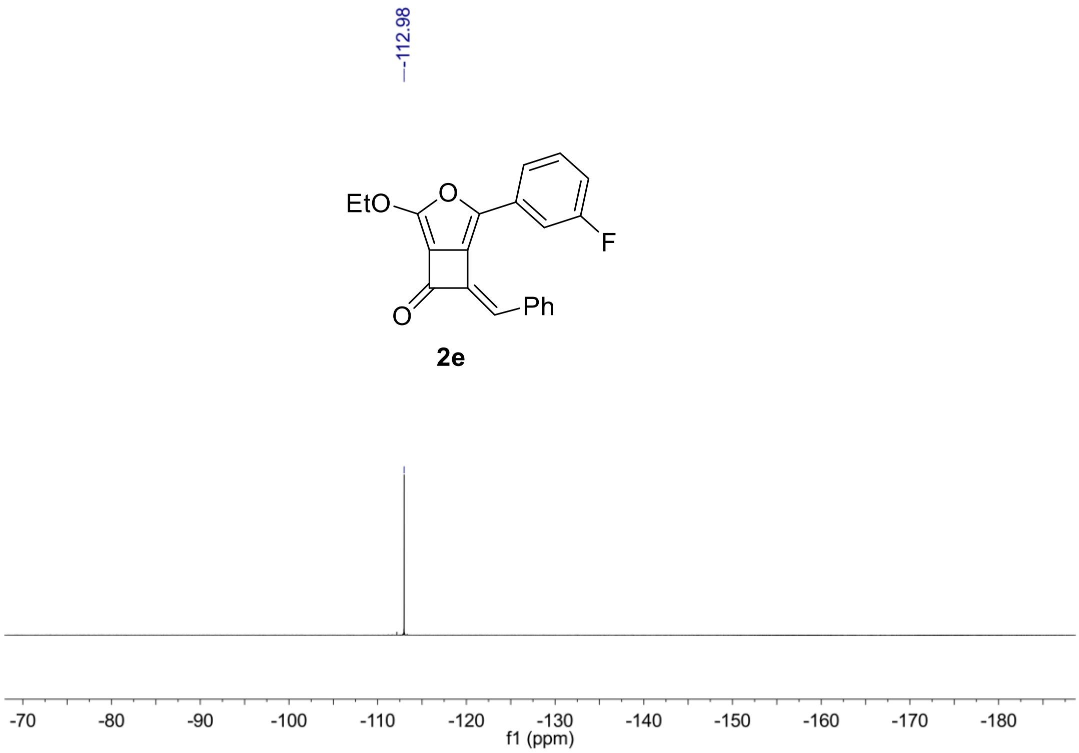

Supplementary Fig. 12 | ^{19}F NMR of compound 2e (471 MHz, at 25 ºC, in $CDCl_3$).

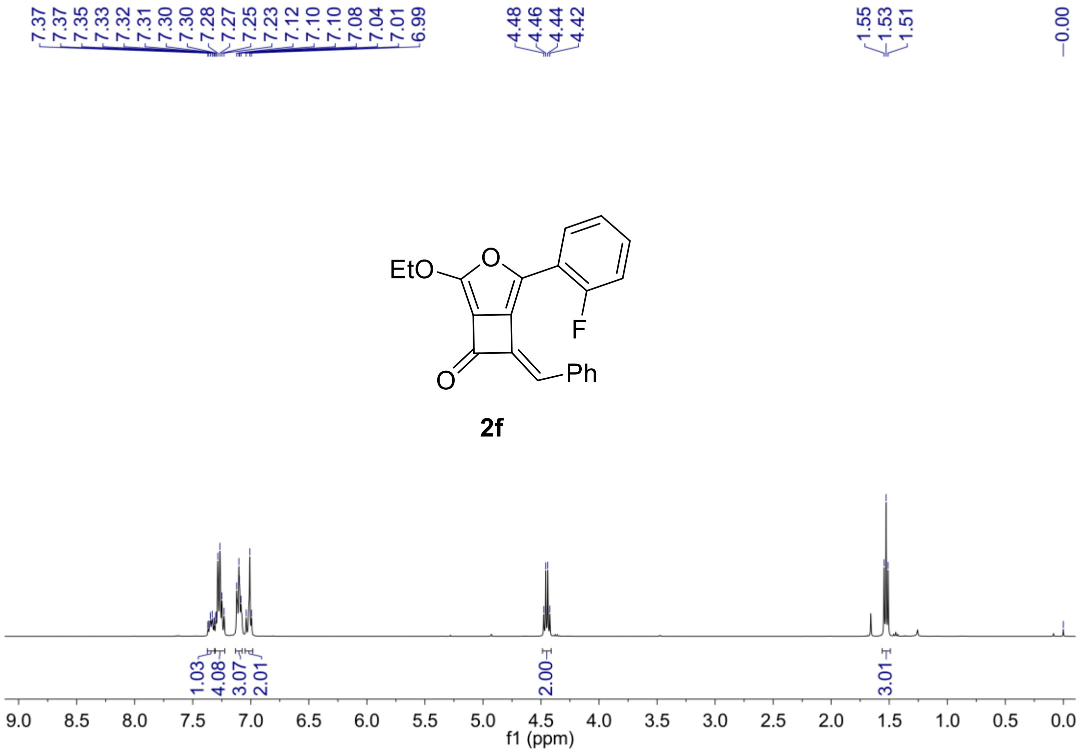

Supplementary Fig. 13 | ^{1}H NMR of compound 2f (400 MHz, at 25 ºC, in $CDCl_3$).

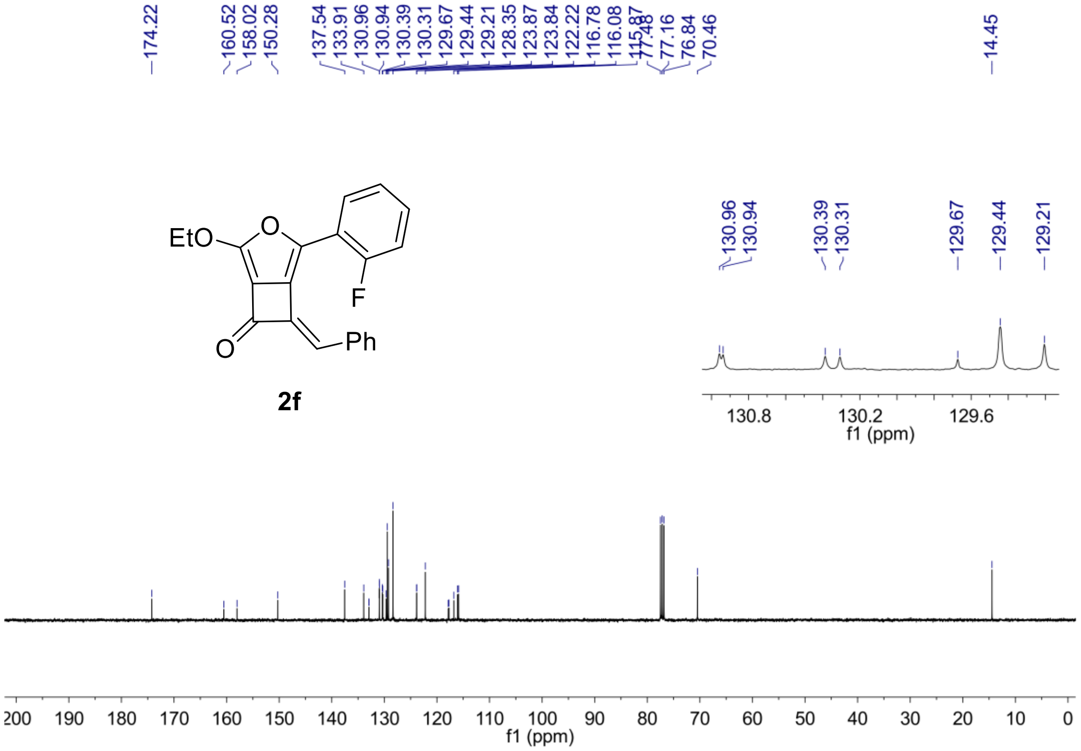

Supplementary Fig. 14 | ^{13}C NMR of compound 2f (100 MHz, at 25 ºC, in $CDCl_3$).

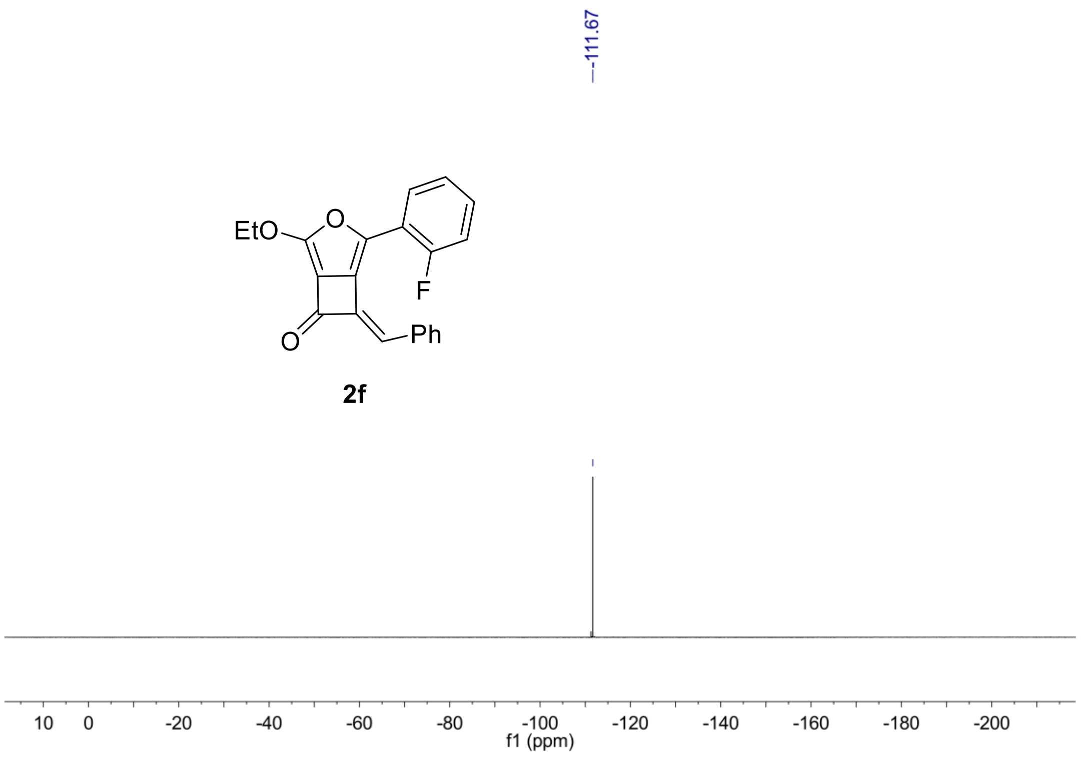

Supplementary Fig. 15 | ^{19}F NMR of compound 2f (376 MHz, at 25 ^{o}C, in $CDCl_3$).

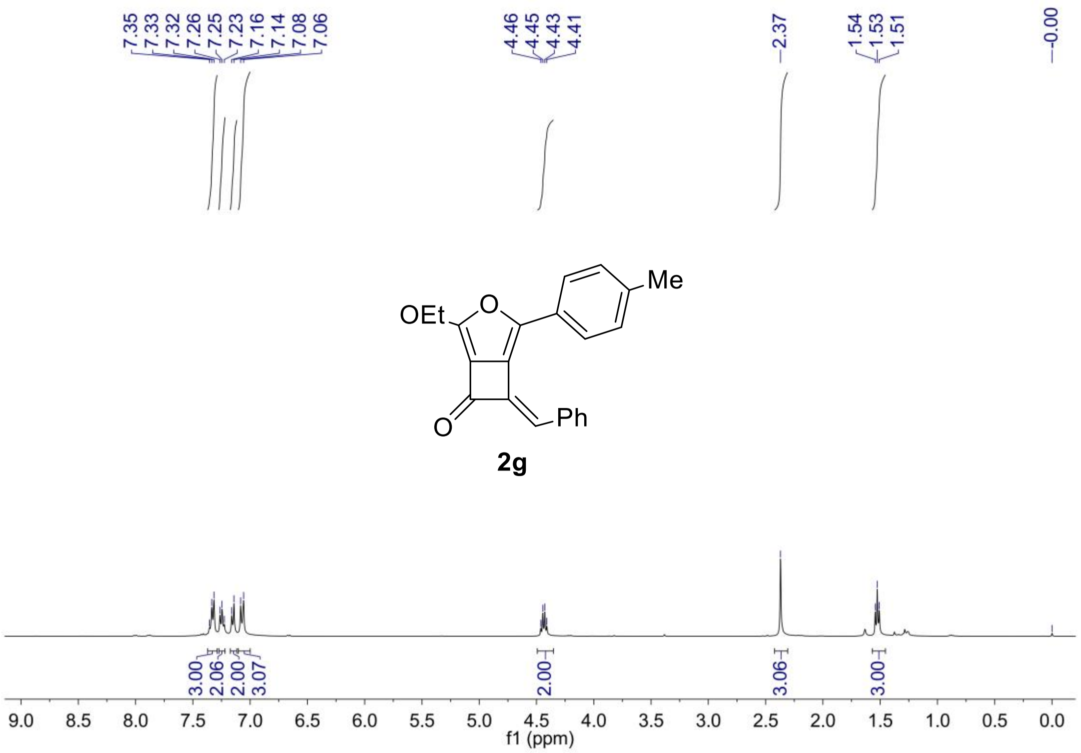

Supplementary Fig. 16 | ^{1}H NMR of compound 2g (400 MHz, at 25 ^{o}C, in $CDCl_3$).

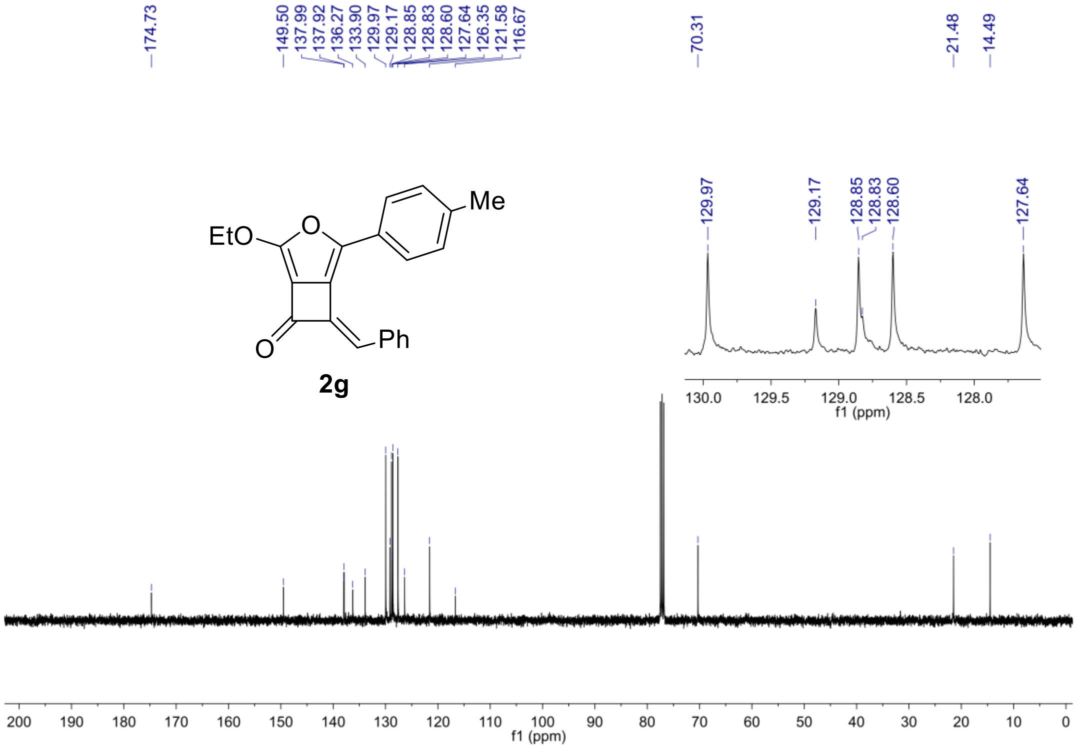

Supplementary Fig. 17 | ^{13}C NMR of compound 2g (100 MHz, at 25 °C, in $CDCl_3$).

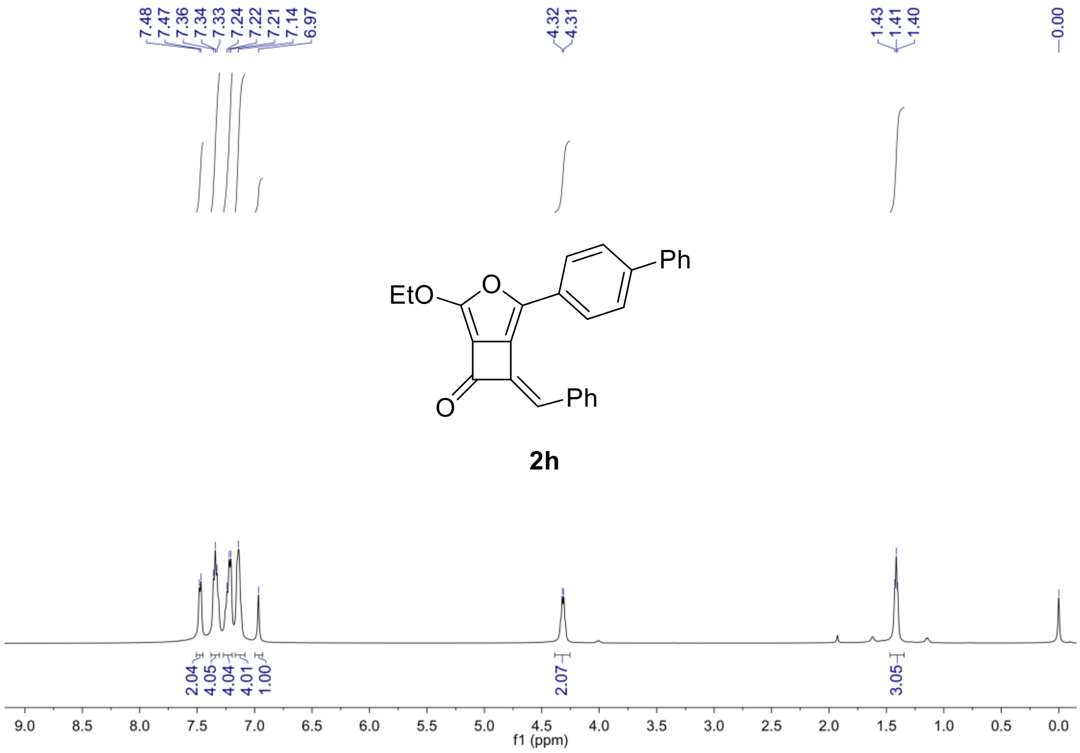

Supplementary Fig. 18 | ^{1}H NMR of compound 2h (500 MHz, at 25 °C, in $CDCl_3$).

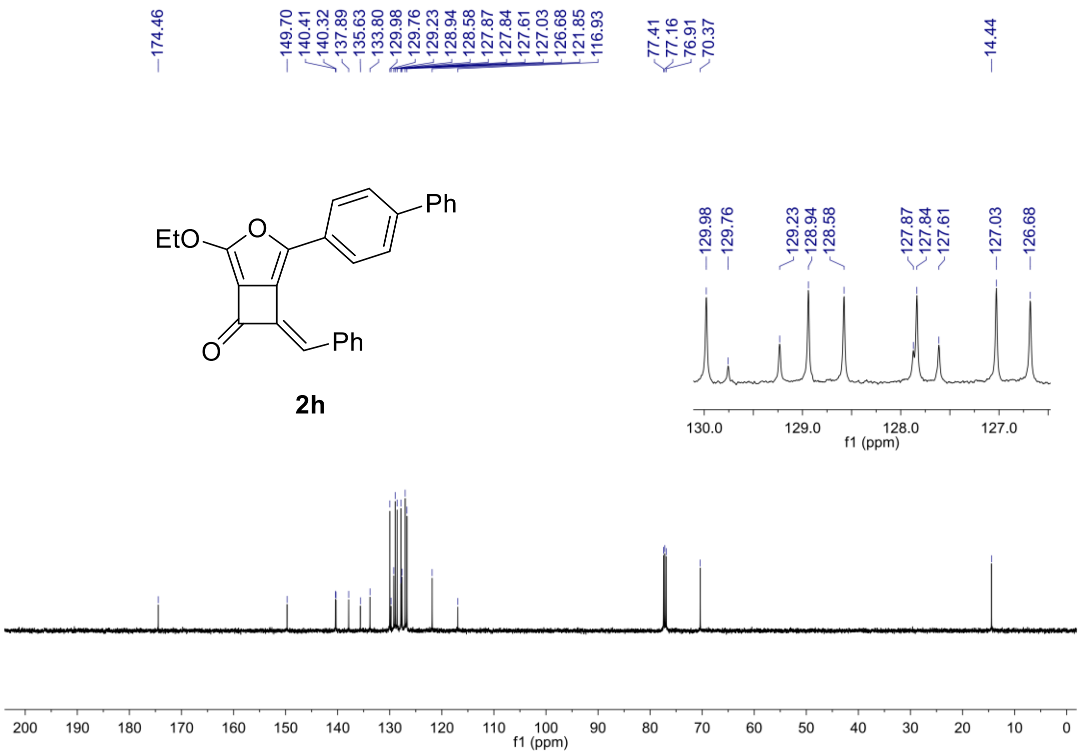

Supplementary Fig. 19 | ^{13}C NMR of compound 2h (125 MHz, at 25 °C, in $CDCl_3$).

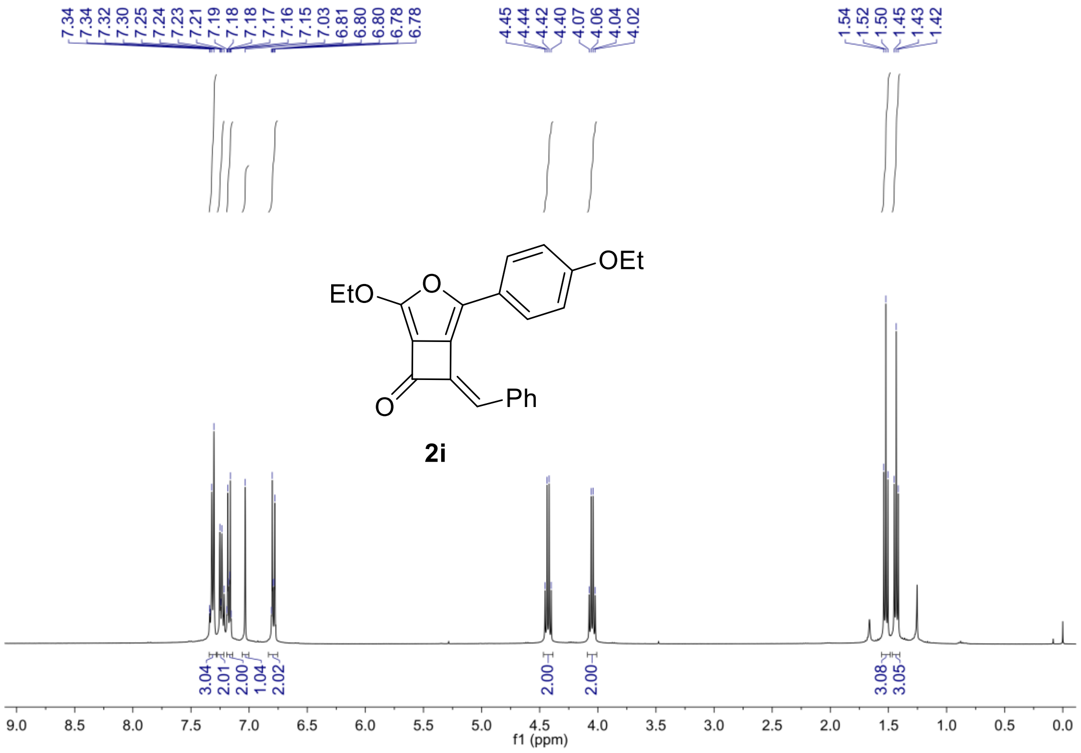

Supplementary Fig. 20 | ^{1}H NMR of compound 2i (400 MHz, at 25 °C, in $CDCl_3$).

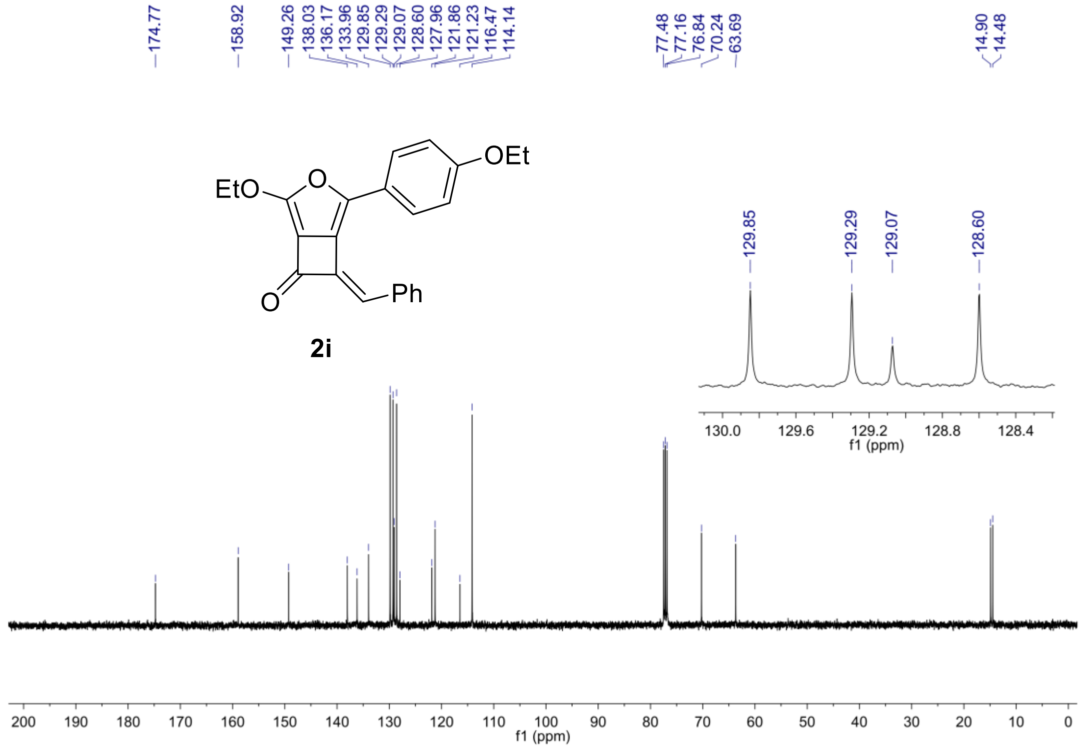

Supplementary Fig. 21 | ^{13}C NMR of compound 2i (100 MHz, at 25 °C, in $CDCl_3$).

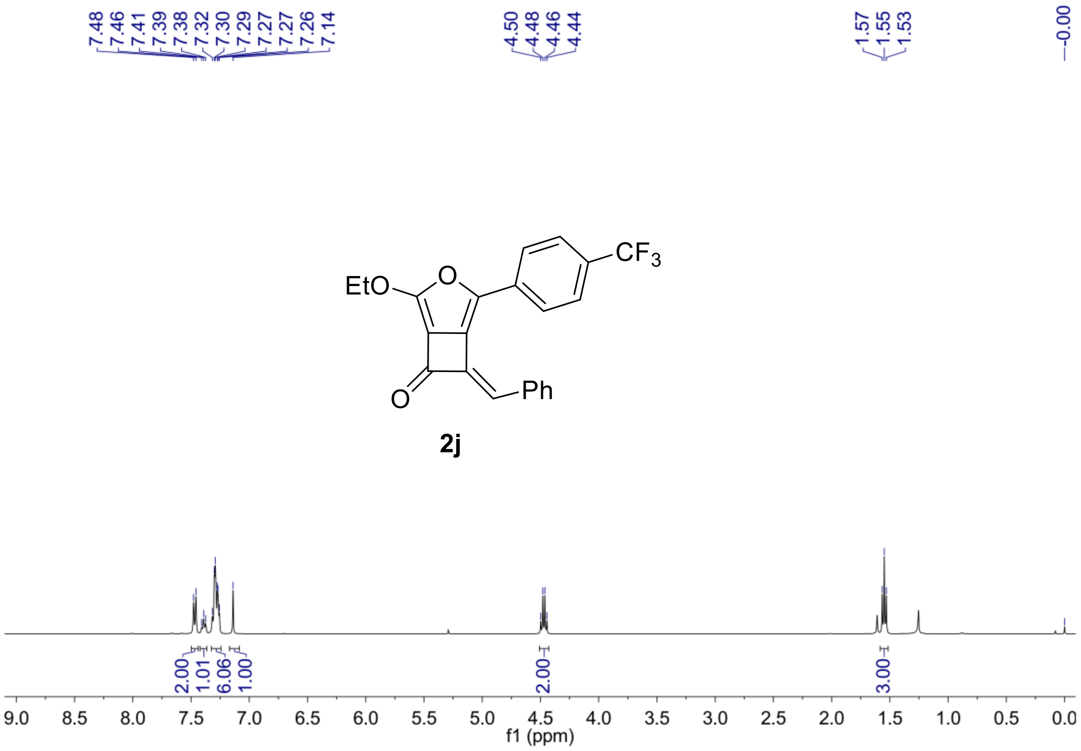

Supplementary Fig. 22 | ^{1}H NMR of compound 2j (400 MHz, at 25 °C, in $CDCl_3$).

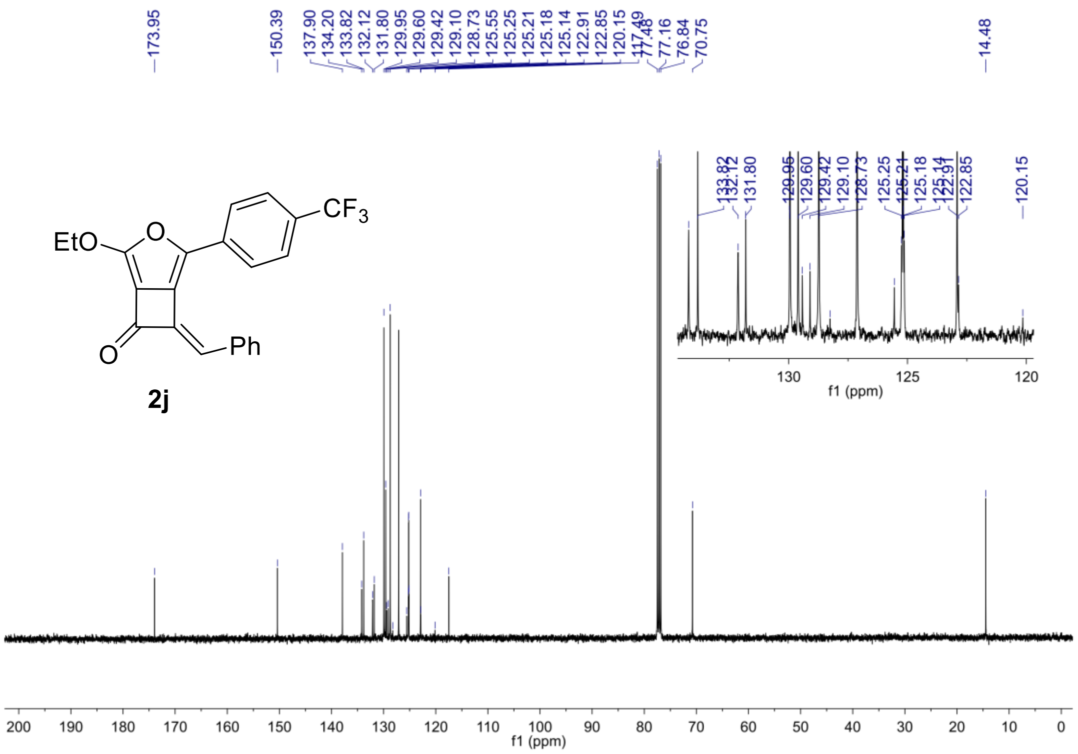

Supplementary Fig. 23 | ^{13}C NMR of compound 2j (100 MHz, at 25 ^{o}C, in $CDCl_3$).

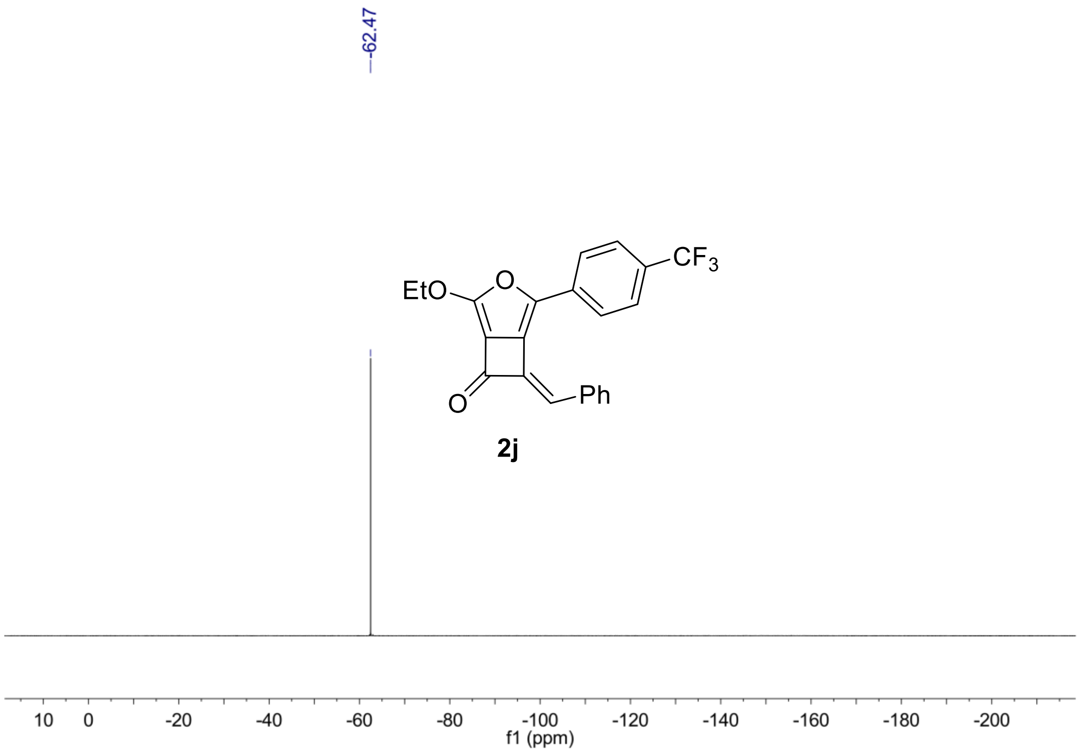

Supplementary Fig. 24 | ^{19}F NMR of compound 2j (376 MHz, at 25 ^{o}C, in $CDCl_3$).

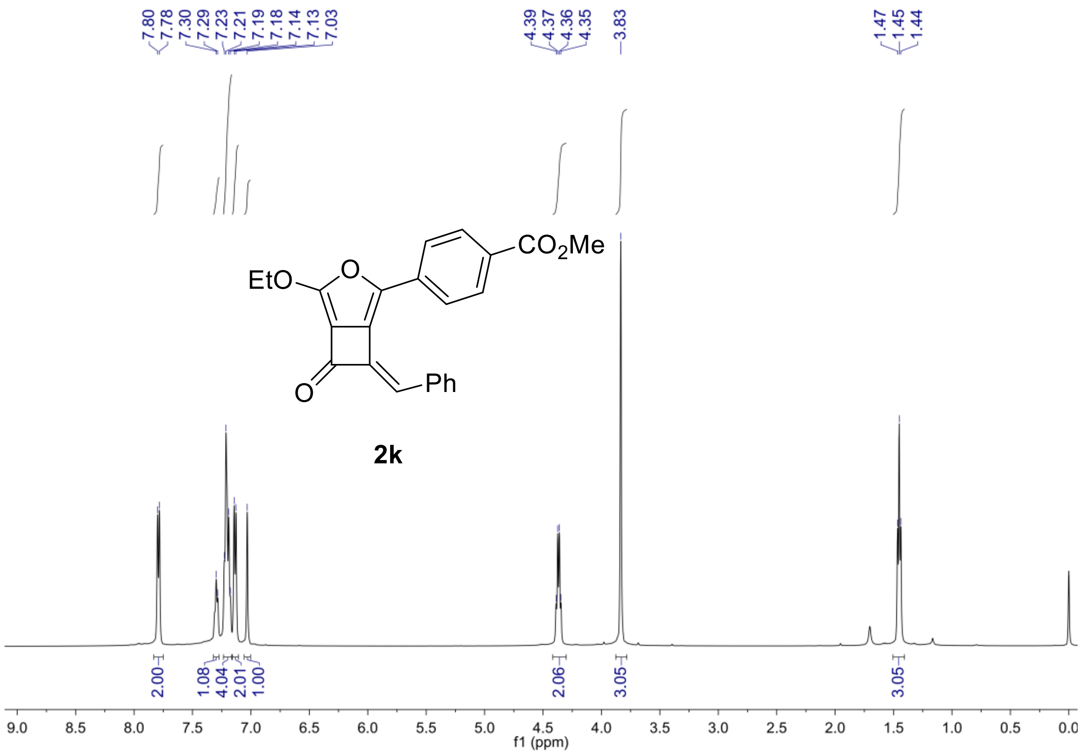

Supplementary Fig. 25 | ^{1}H NMR of compound 2k (500 MHz, at 25 °C, in $CDCl_3$).

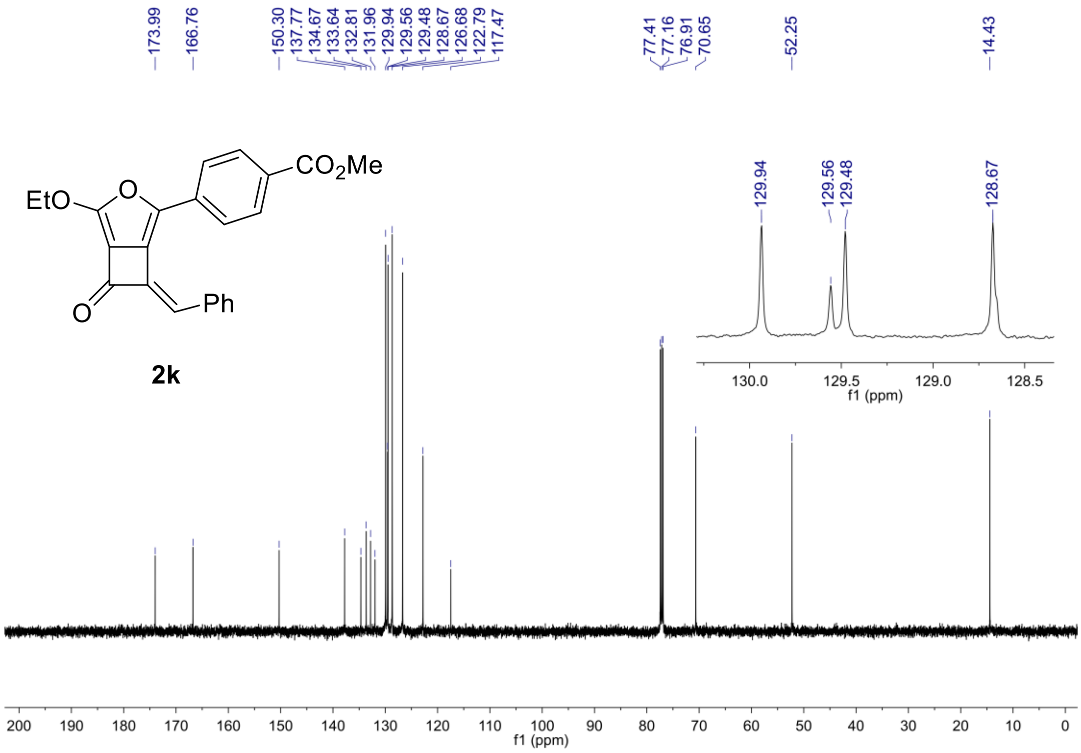

Supplementary Fig. 26 | ^{13}C NMR of compound 2k (125 MHz, at 25 °C, in $CDCl_3$).

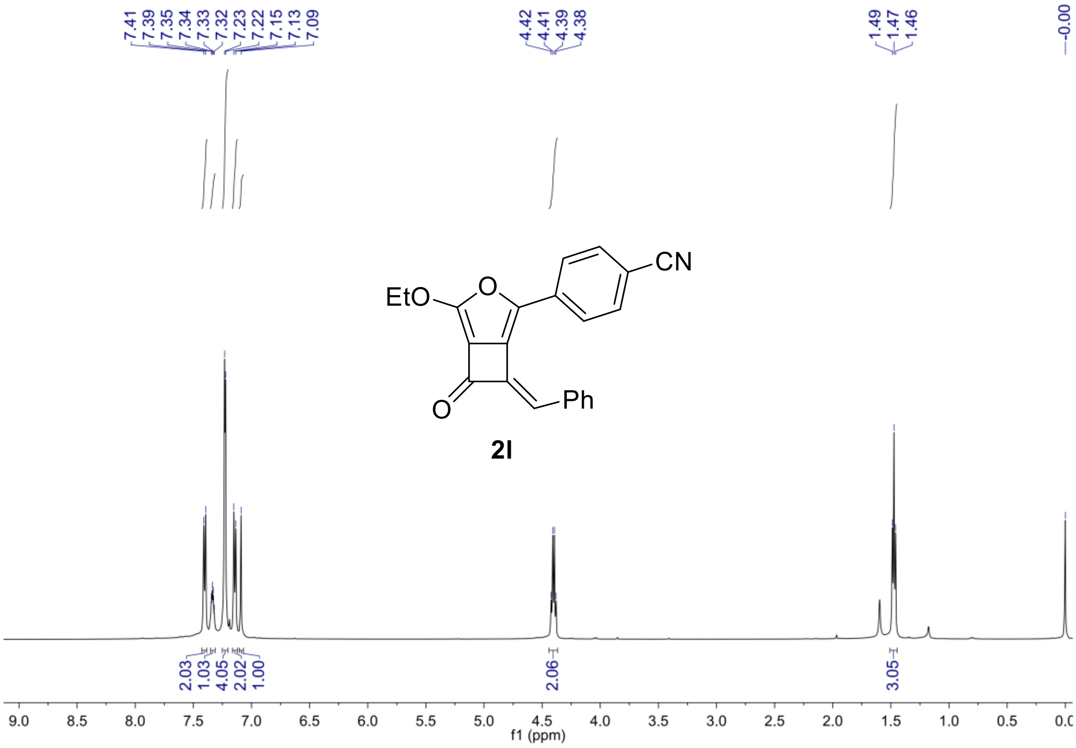

Supplementary Fig. 27 | ^{1}H NMR of compound 2l (500 MHz, at 25 ^{o}C, in $CDCl_3$).

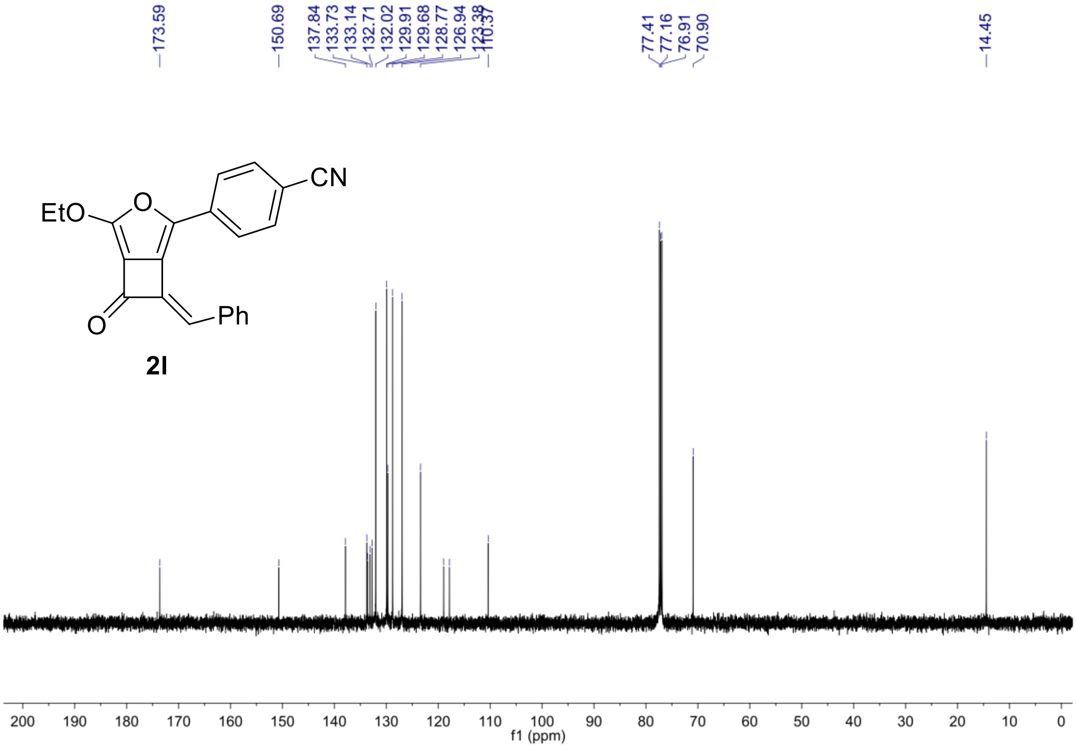

Supplementary Fig. 28 | ^{13}C NMR of compound 2l (125 MHz, at 25 ^{o}C, in $CDCl_3$).

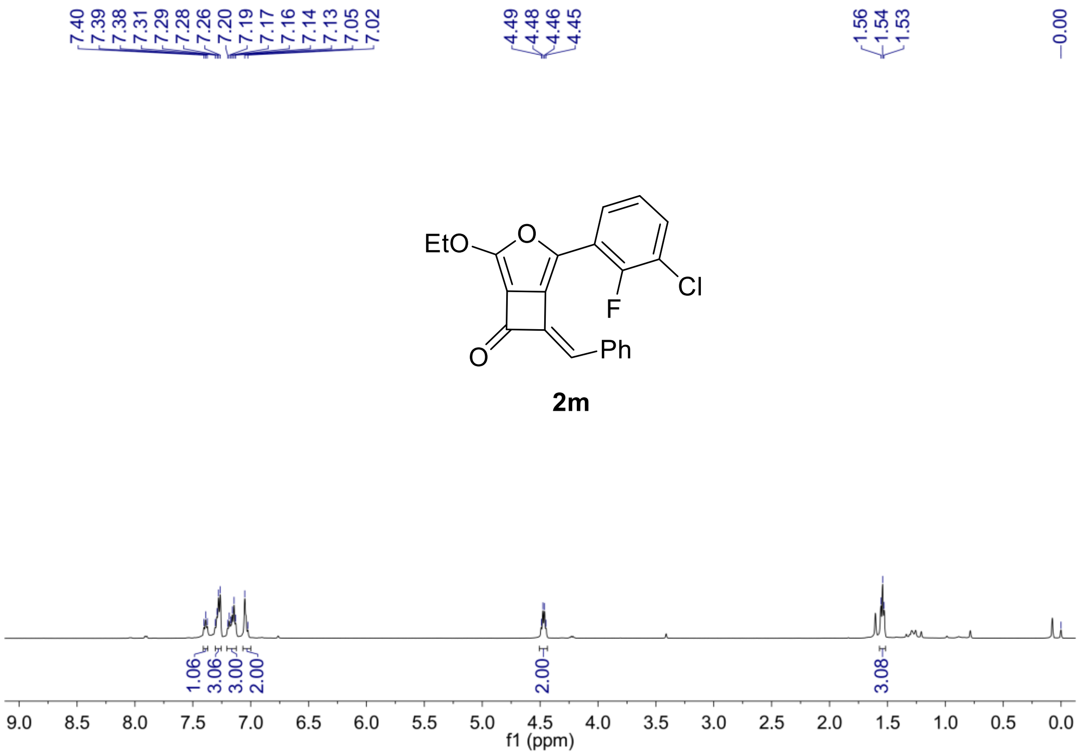

Supplementary Fig. 29 | ^{1}H NMR of compound 2m (500 MHz, at 25 °C, in $CDCl_3$).

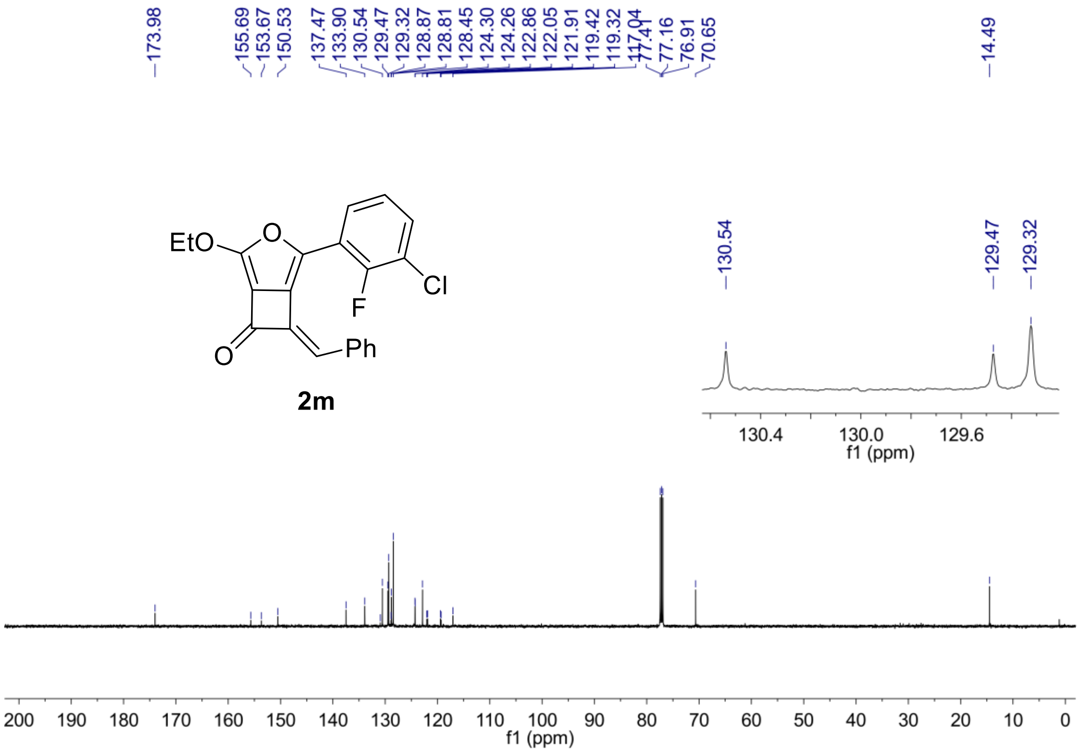

Supplementary Fig. 30 | ^{13}C NMR of compound 2m (125 MHz, at 25 °C, in $CDCl_3$).

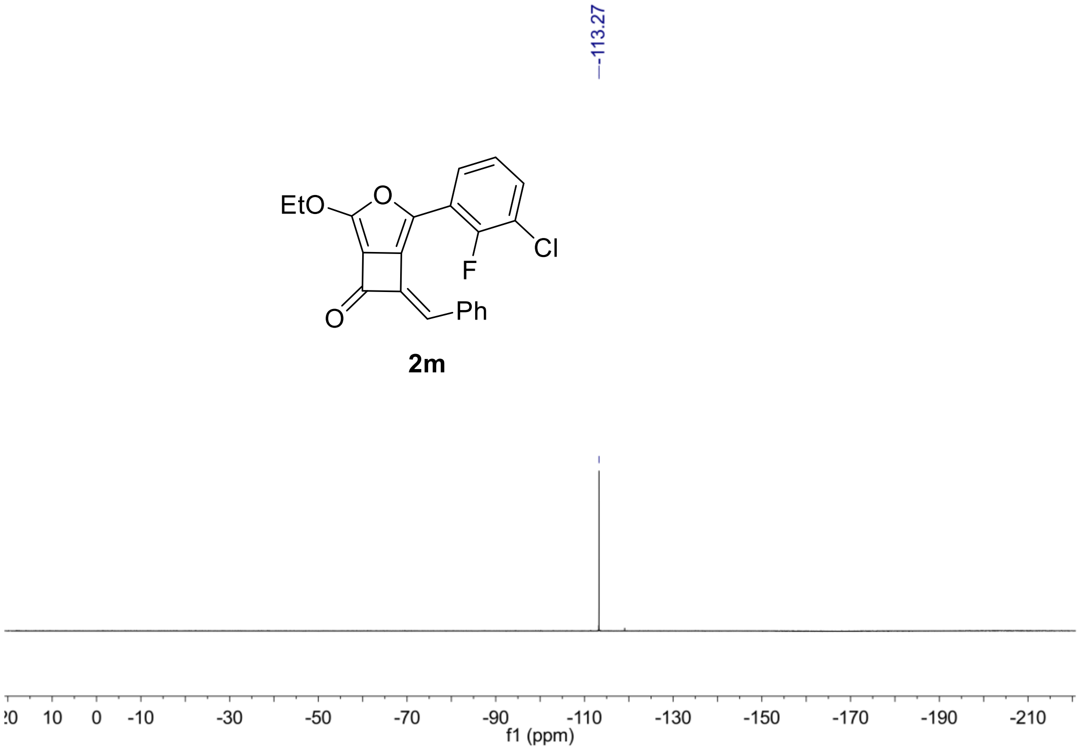

Supplementary Fig. 31 | ^{19}F NMR of compound 2m (471 MHz, at 25 °C, in $CDCl_3$).

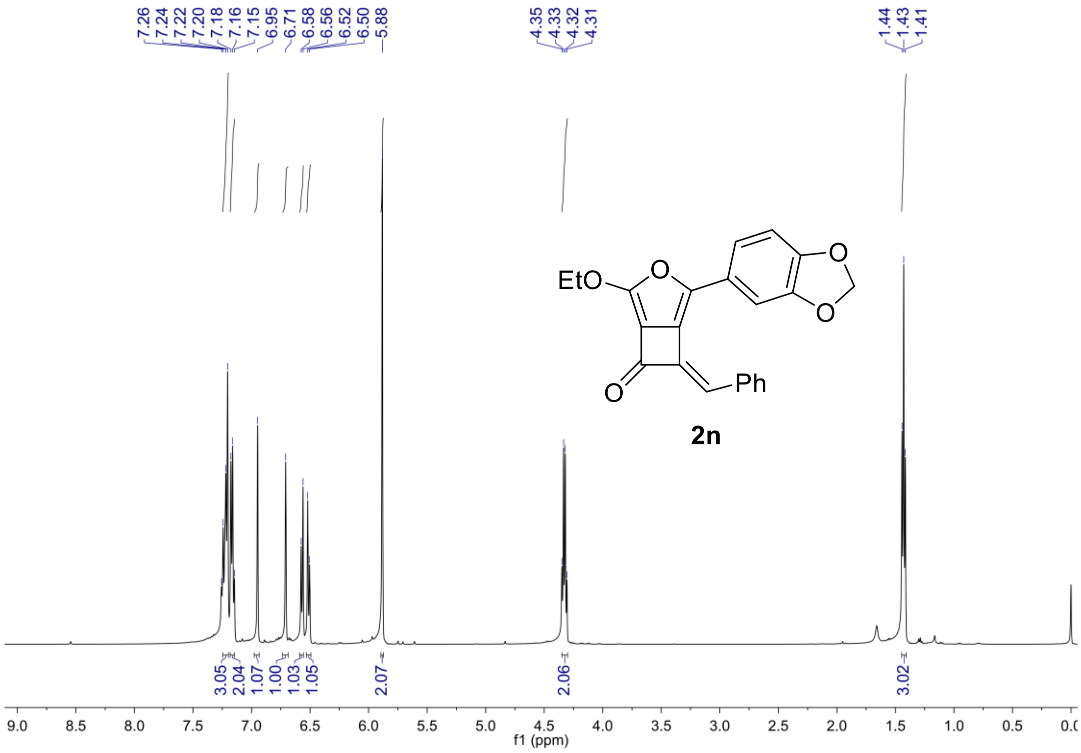

Supplementary Fig. 32 | ^{1}H NMR of compound 2n (500 MHz, at 25 °C, in $CDCl_3$).

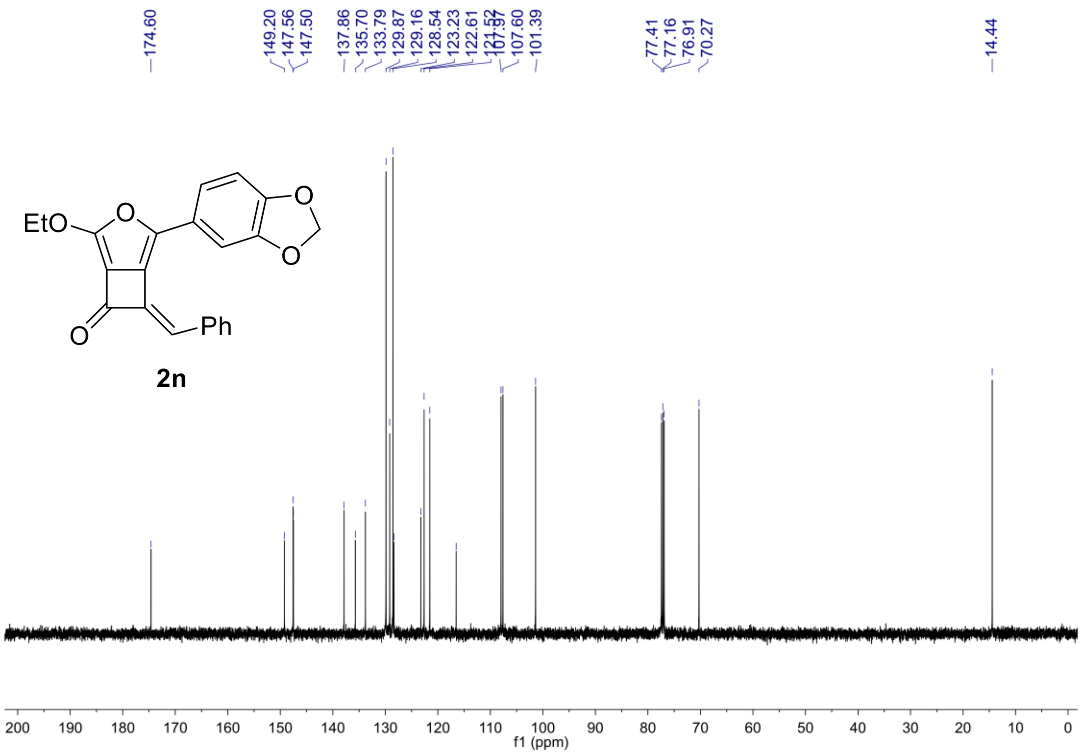

Supplementary Fig. 33 | ^{13}C NMR of compound 2n (125 MHz, at 25 °C, in $CDCl_3$).

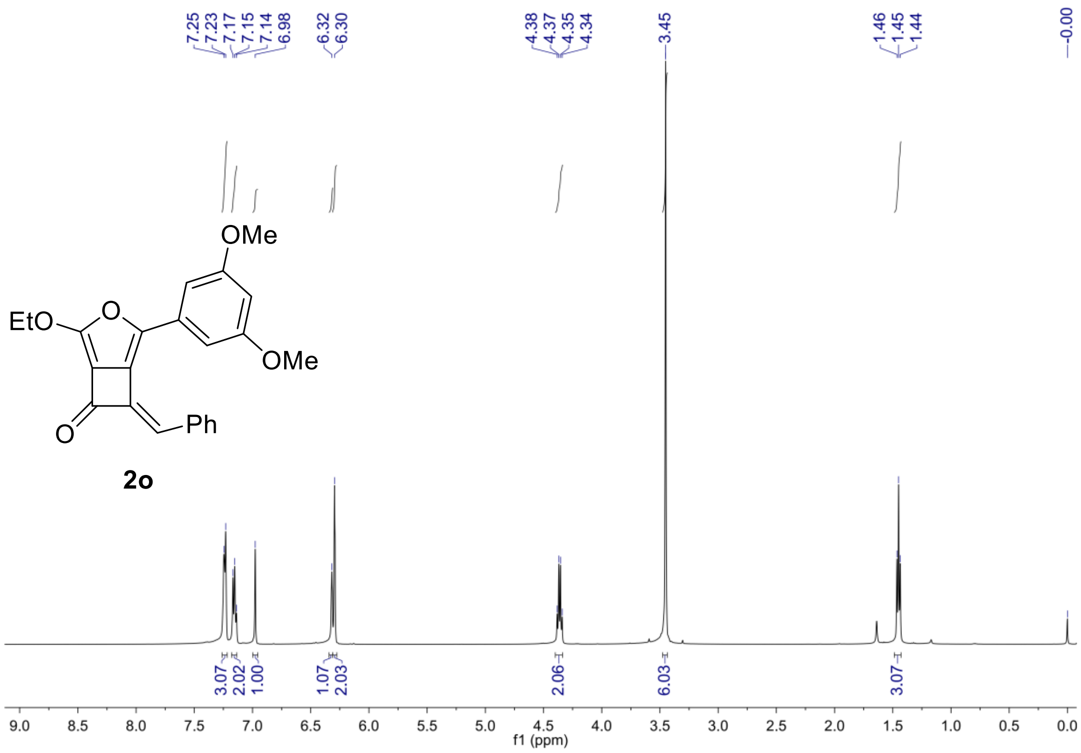

Supplementary Fig. 34 | ^{1}H NMR of compound 2o (500 MHz, at 25 °C, in $CDCl_3$).

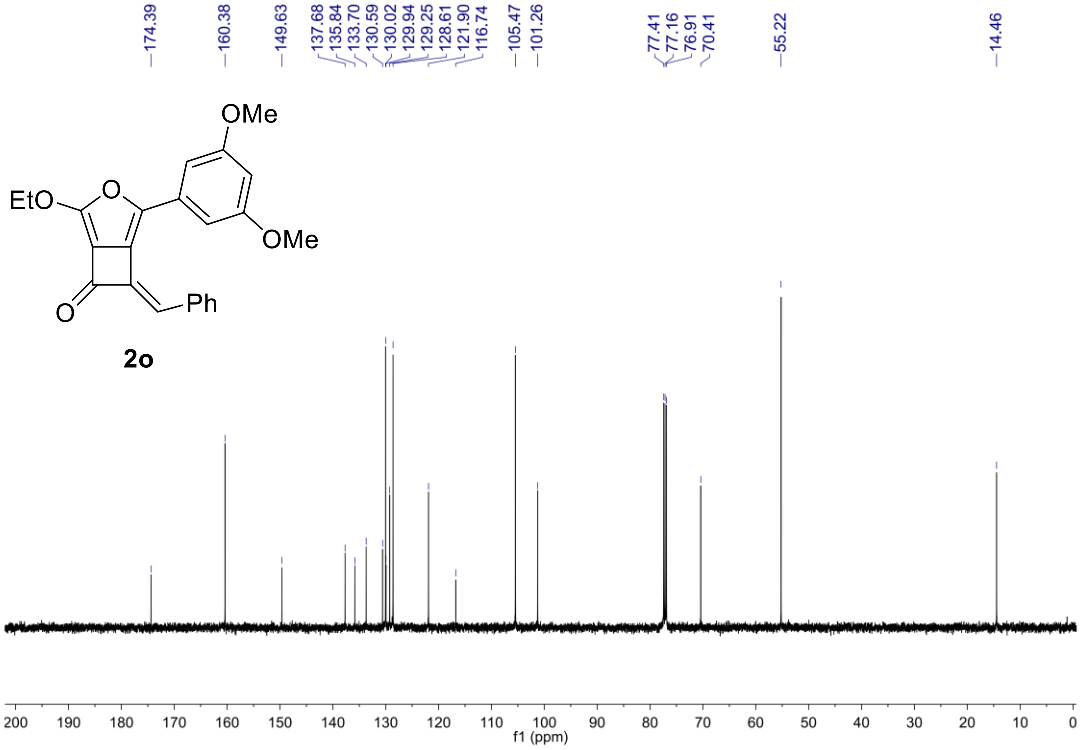

Supplementary Fig. 35 | ^{13}C NMR of compound 2o (125 MHz, at 25 °C, in $CDCl_3$).

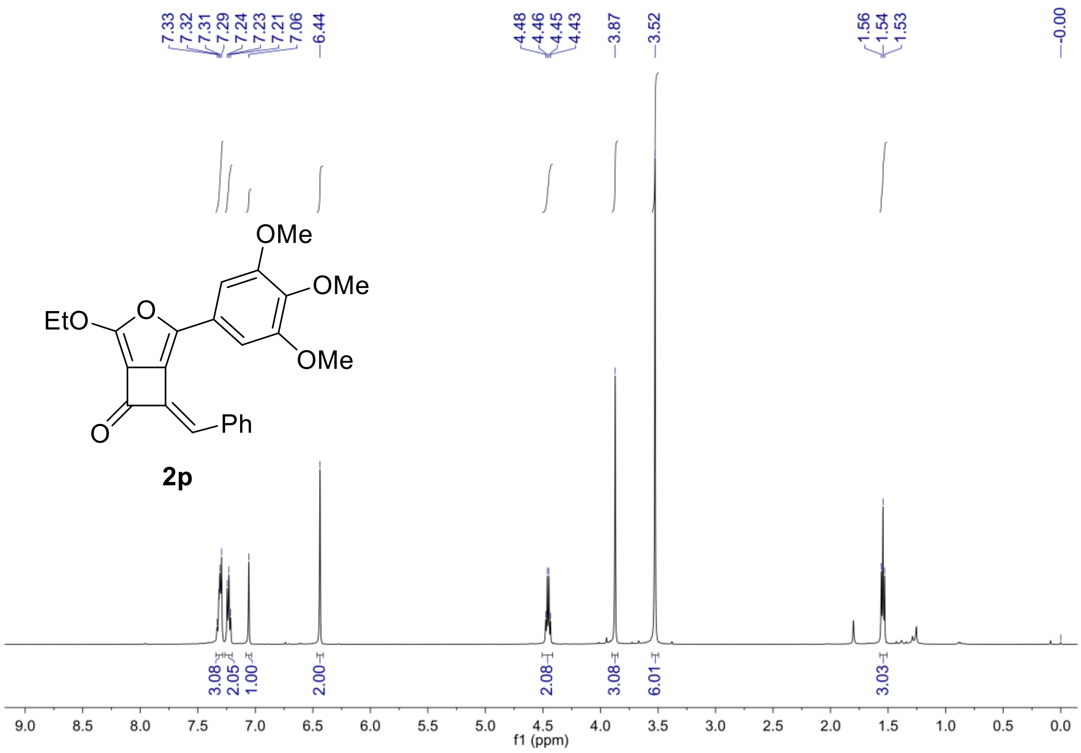

Supplementary Fig. 36 | ^{1}H NMR of compound 2p (500 MHz, at 25 °C, in $CDCl_3$).

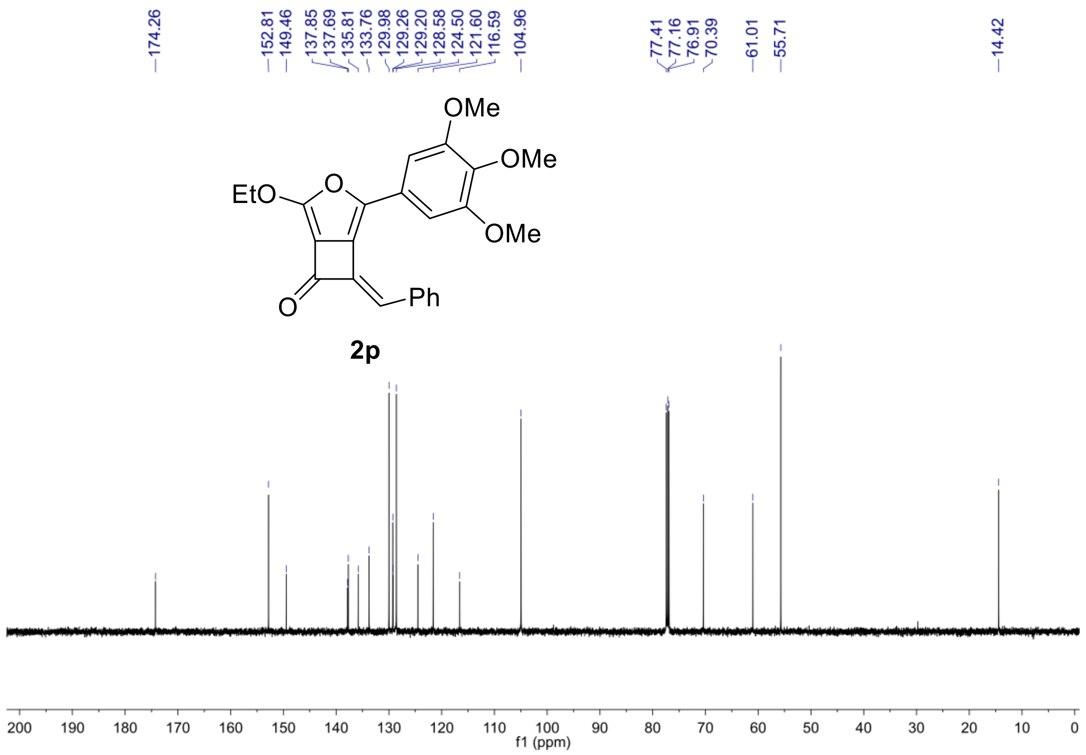

Supplementary Fig. 37 | ^{13}C NMR of compound 2p (125 MHz, at 25 ^{o}C, in $CDCl_3$).

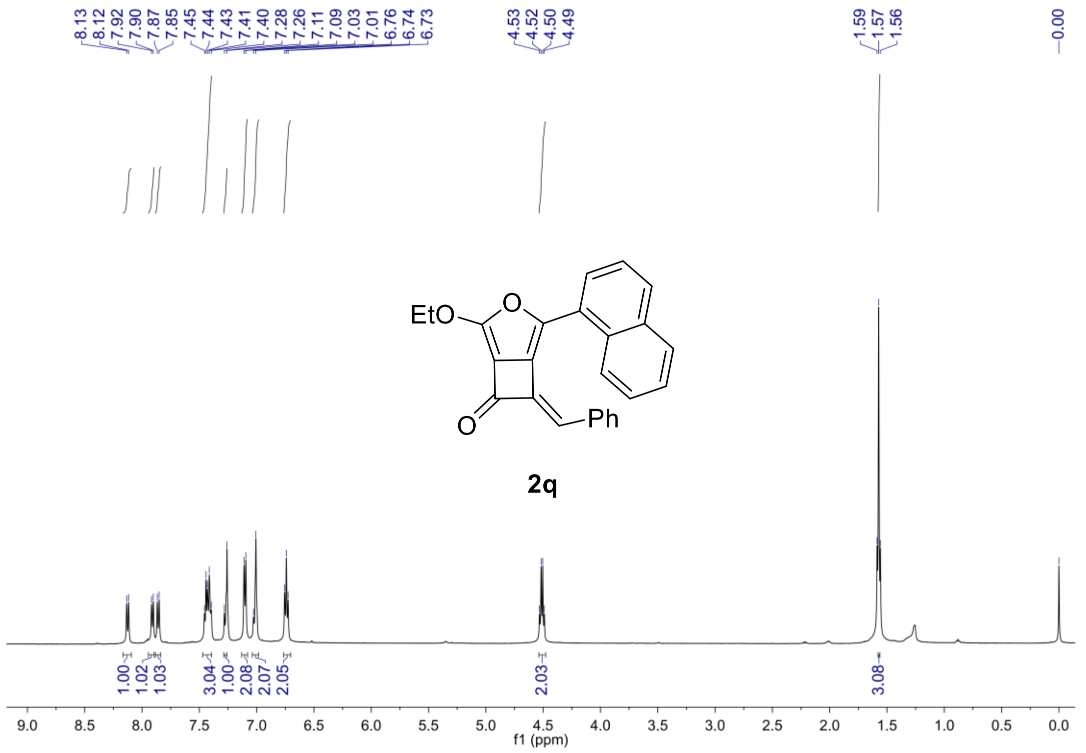

Supplementary Fig. 38 | ^{1}H NMR of compound 2q (500 MHz, at 25 ^{o}C, in $CDCl_3$).

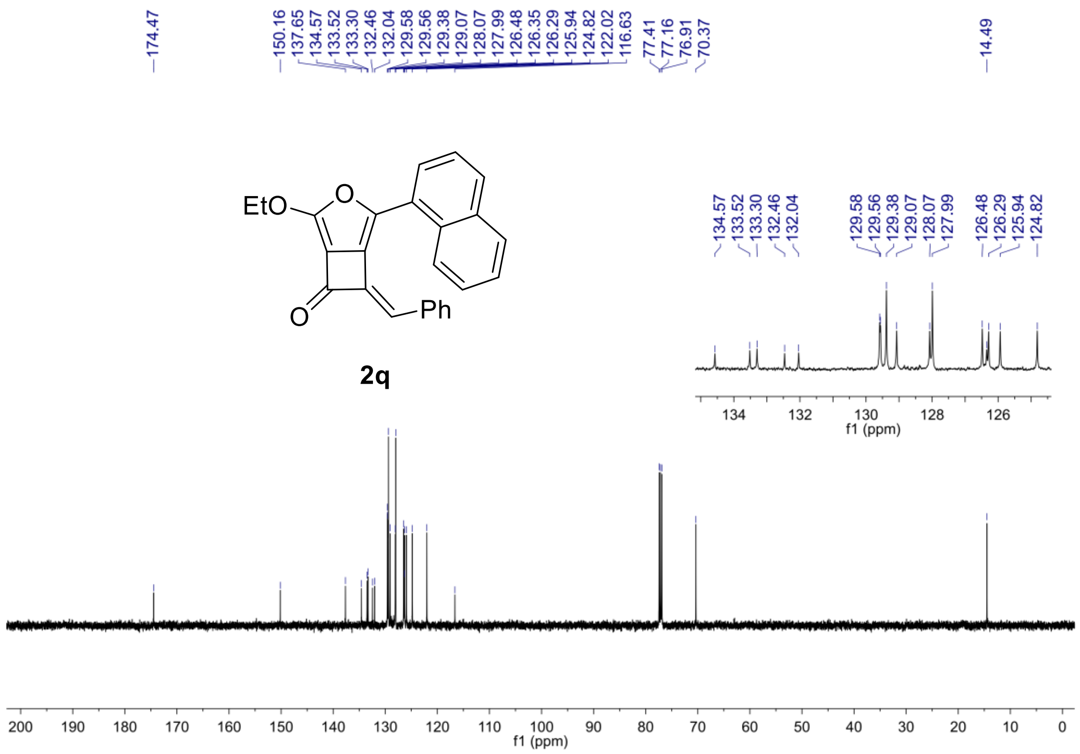

Supplementary Fig. 39 | ^{13}C NMR of compound 2q (125 MHz, at 25 °C, in $CDCl_3$).

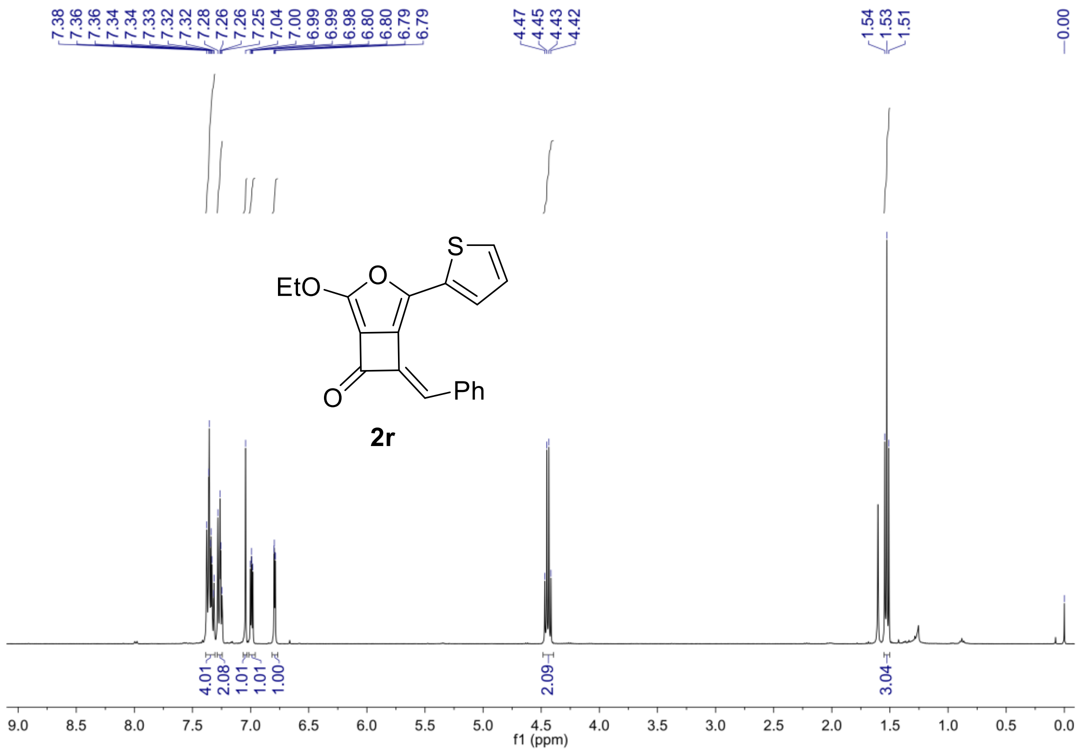

Supplementary Fig. 40 | ^{1}H NMR of compound 2r (400 MHz, at 25 °C, in $CDCl_3$).

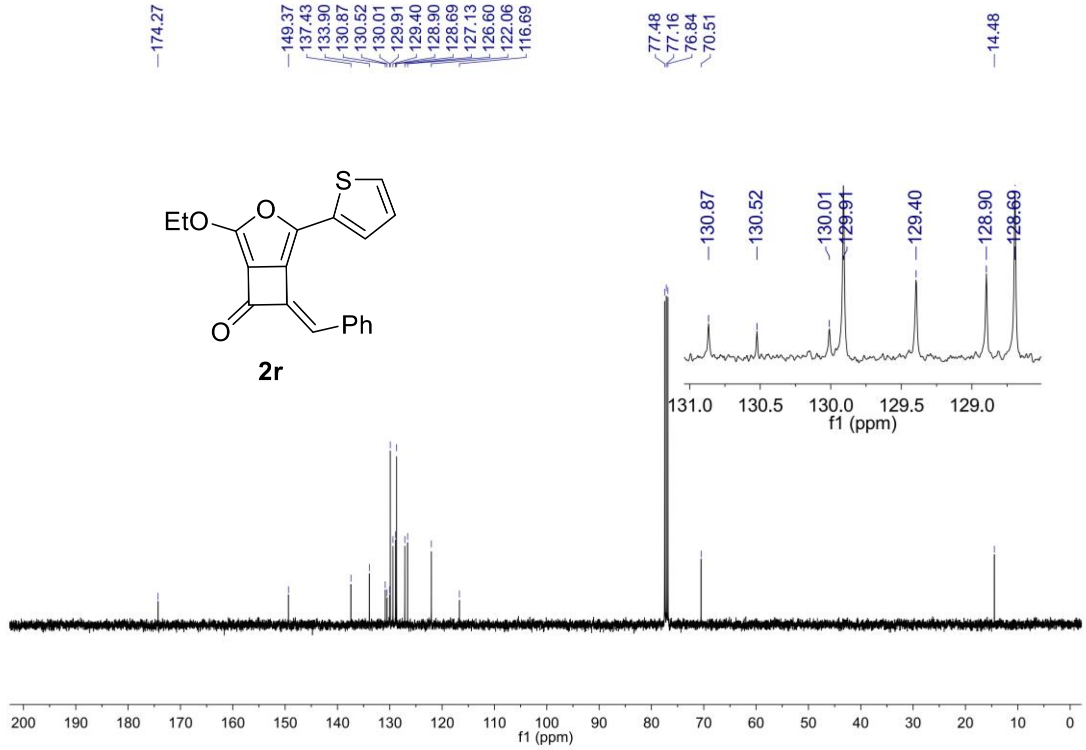

Supplementary Fig. 41 | ^{13}C NMR of compound 2r (100 MHz, at 25 ^{o}C, in $CDCl_3$).

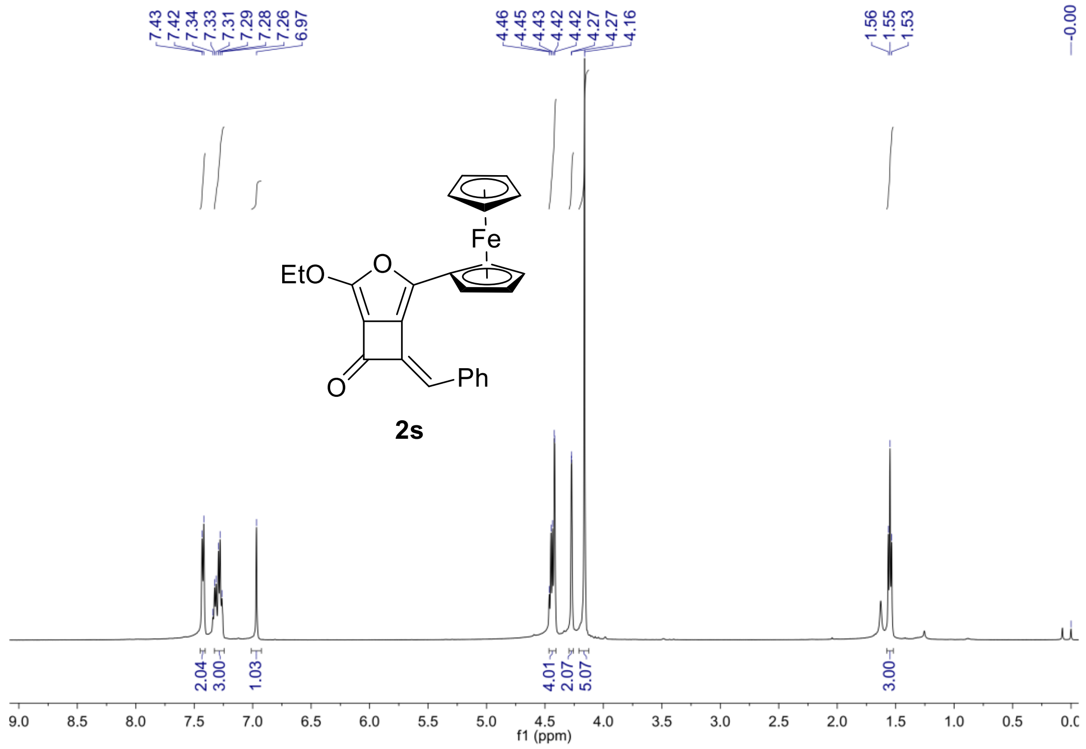

Supplementary Fig. 42 | ^{1}H NMR of compound 2s (500 MHz, at 25 ^{o}C, in $CDCl_3$).

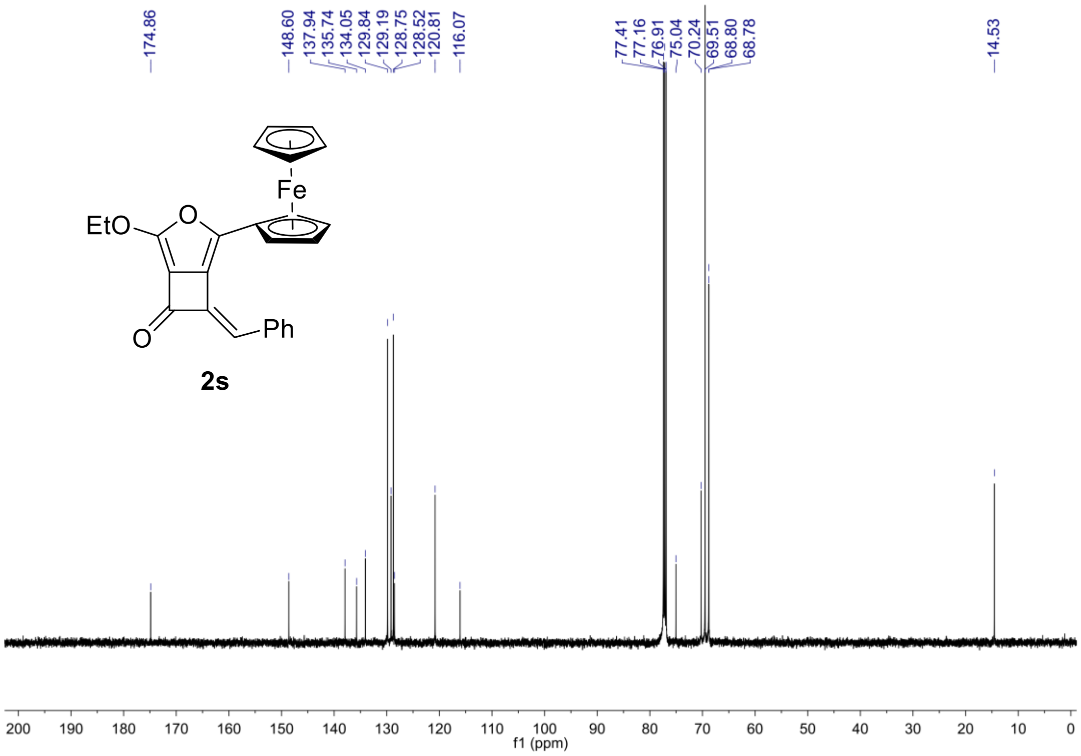

Supplementary Fig. 43 | ^{13}C NMR of compound 2s (125 MHz, at 25 ºC, in $CDCl_3$).

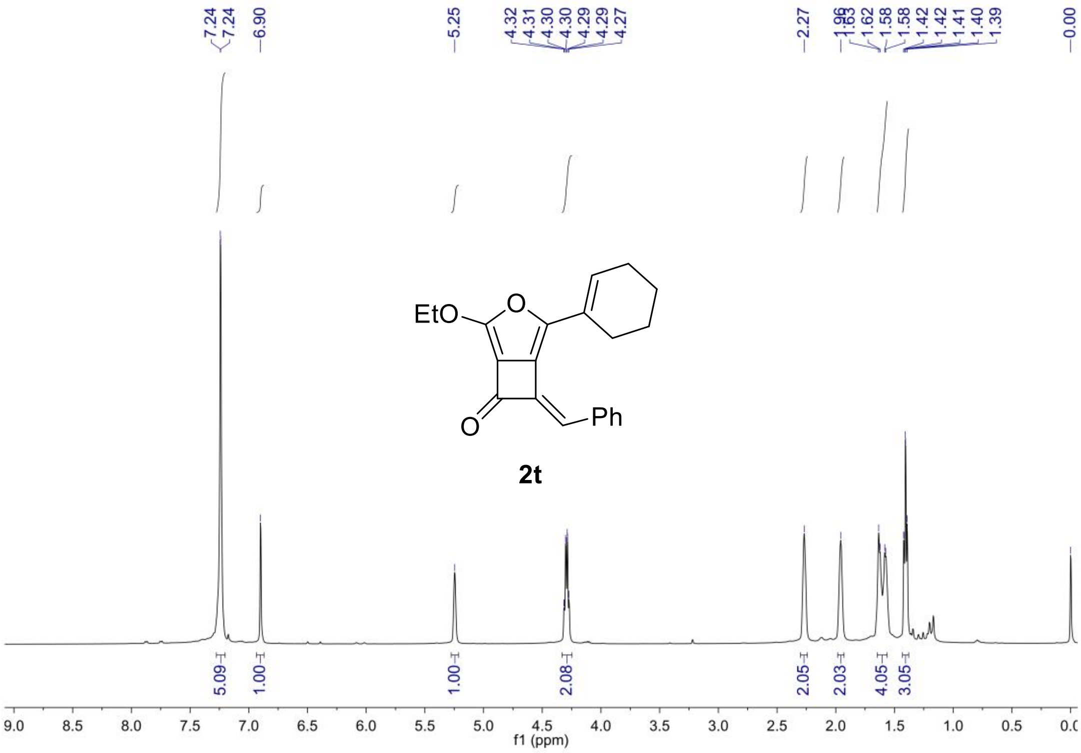

Supplementary Fig. 44 | ^{1}H NMR of compound 2t (500 MHz, at 25 ºC, in $CDCl_3$).

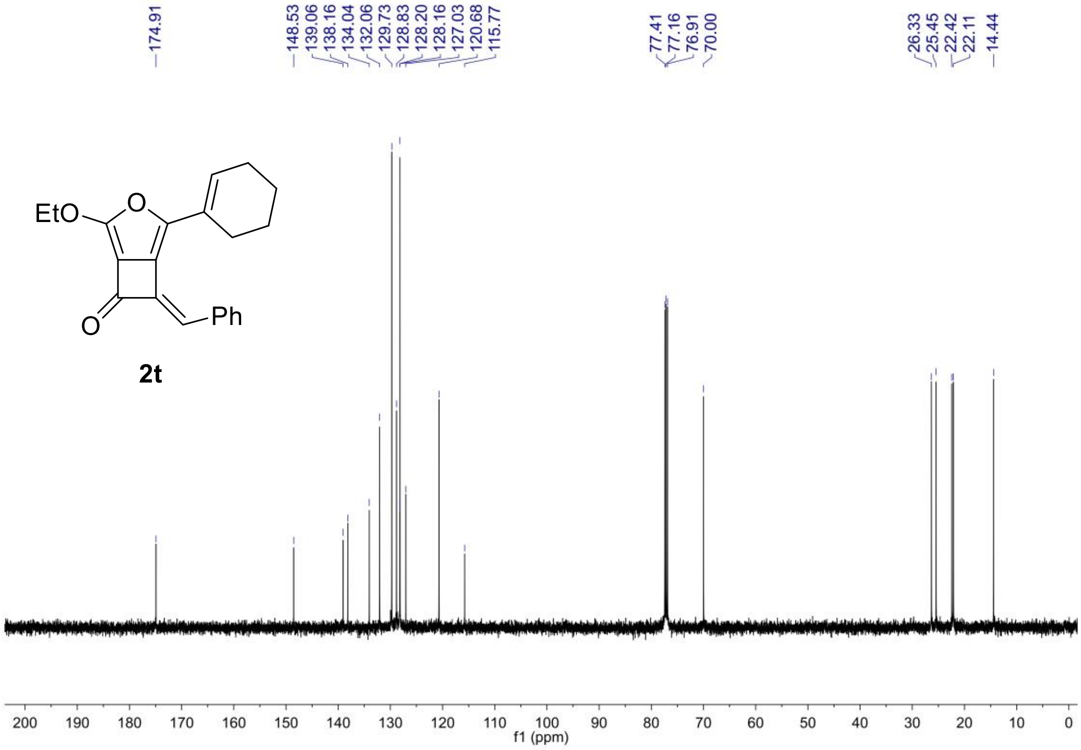

Supplementary Fig. 45 | ^{13}C NMR of compound 2t (125 MHz, at 25 ºC, in $CDCl_3$).

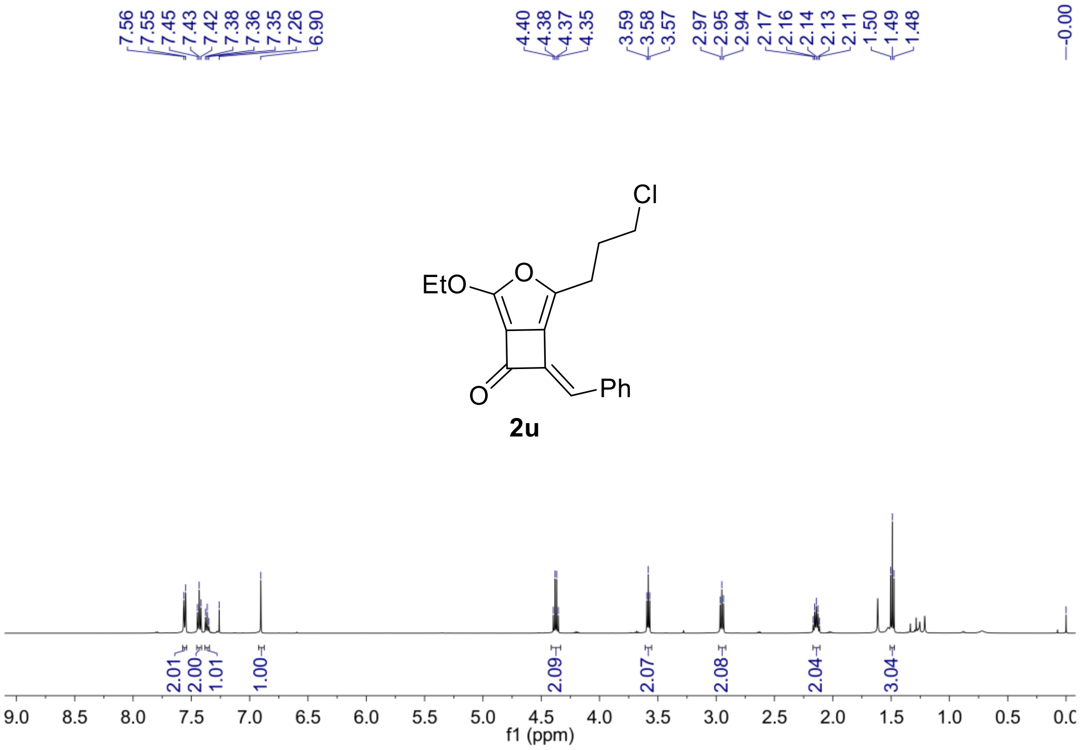

Supplementary Fig. 46 | ^{1}H NMR of compound 2u (500 MHz, at 25 ºC, in $CDCl_3$).

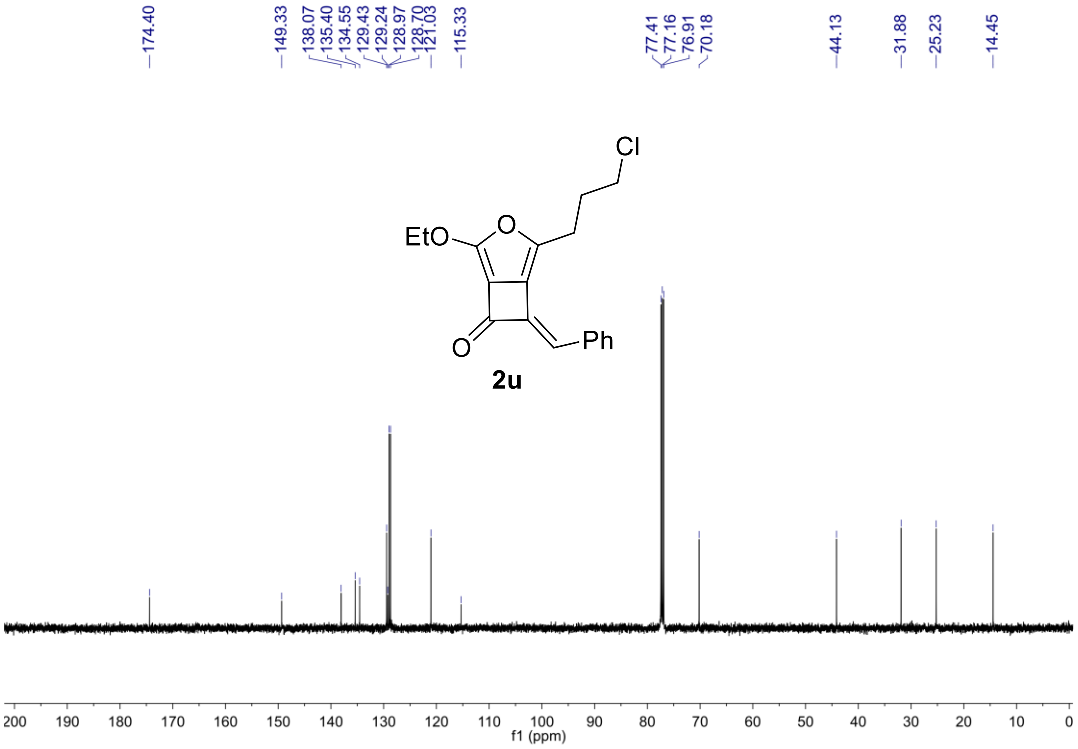

Supplementary Fig. 47 | ^{13}C NMR of compound 2u (125 MHz, at 25 °C, in $CDCl_3$).

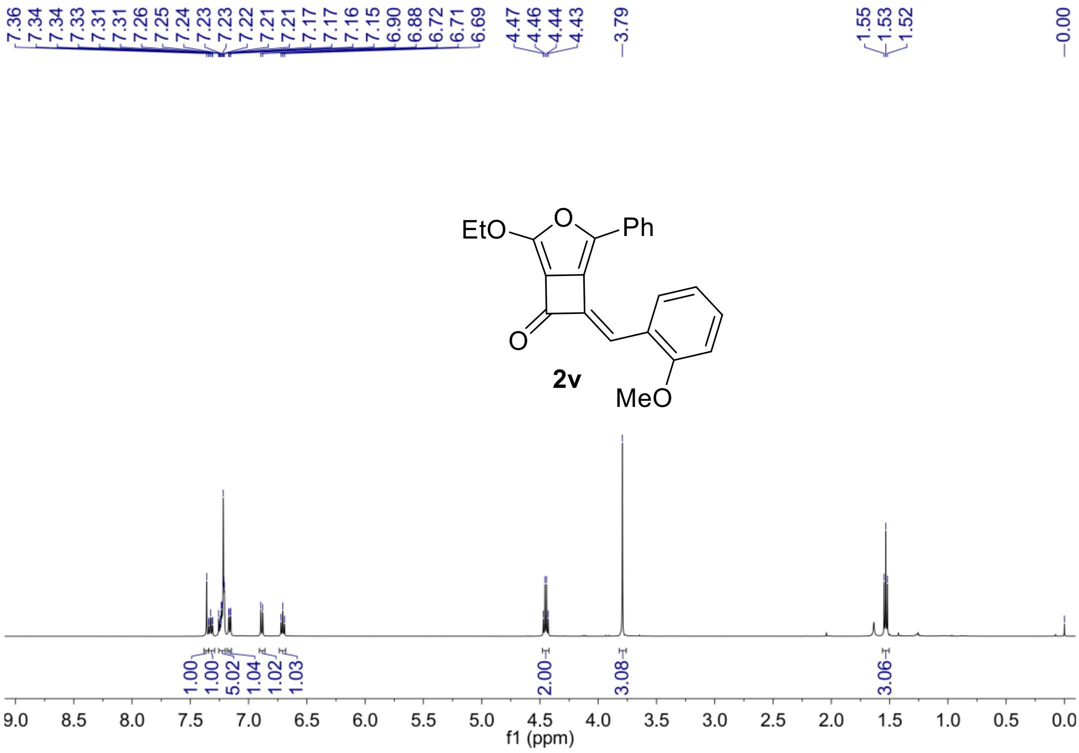

Supplementary Fig. 48 | ^{1}H NMR of compound 2v (500 MHz, at 25 °C, in $CDCl_3$).

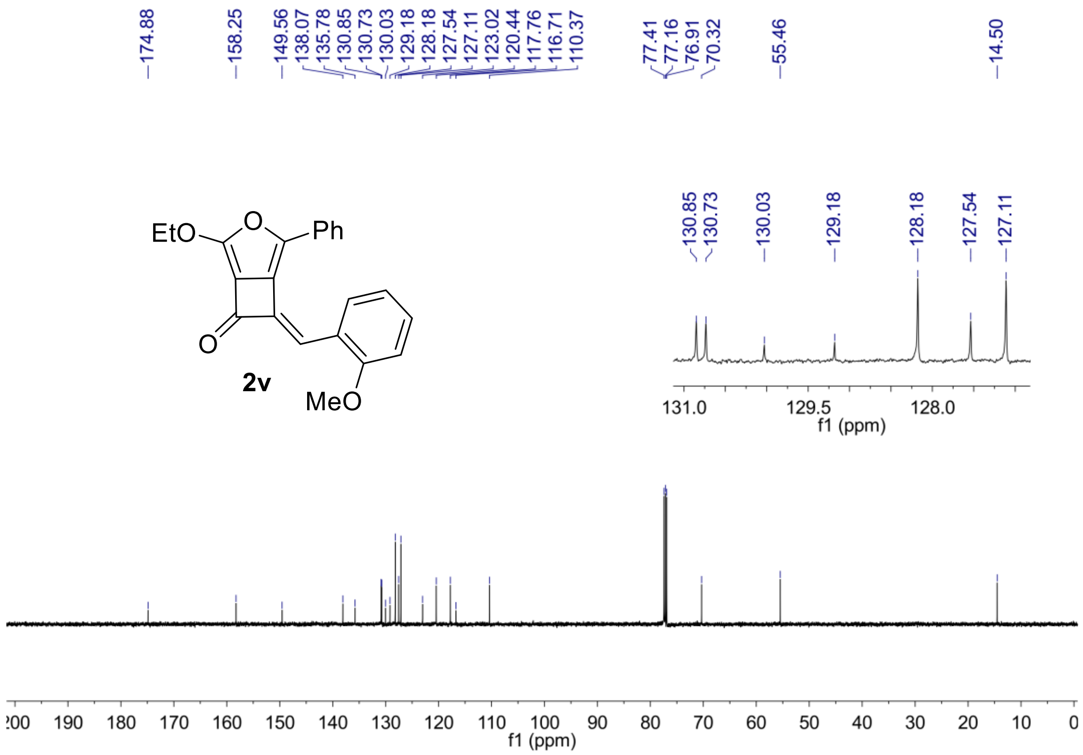

Supplementary Fig. 49 | ^{13}C NMR of compound 2v (125 MHz, at 25 ^{o}C, in $CDCl_3$).

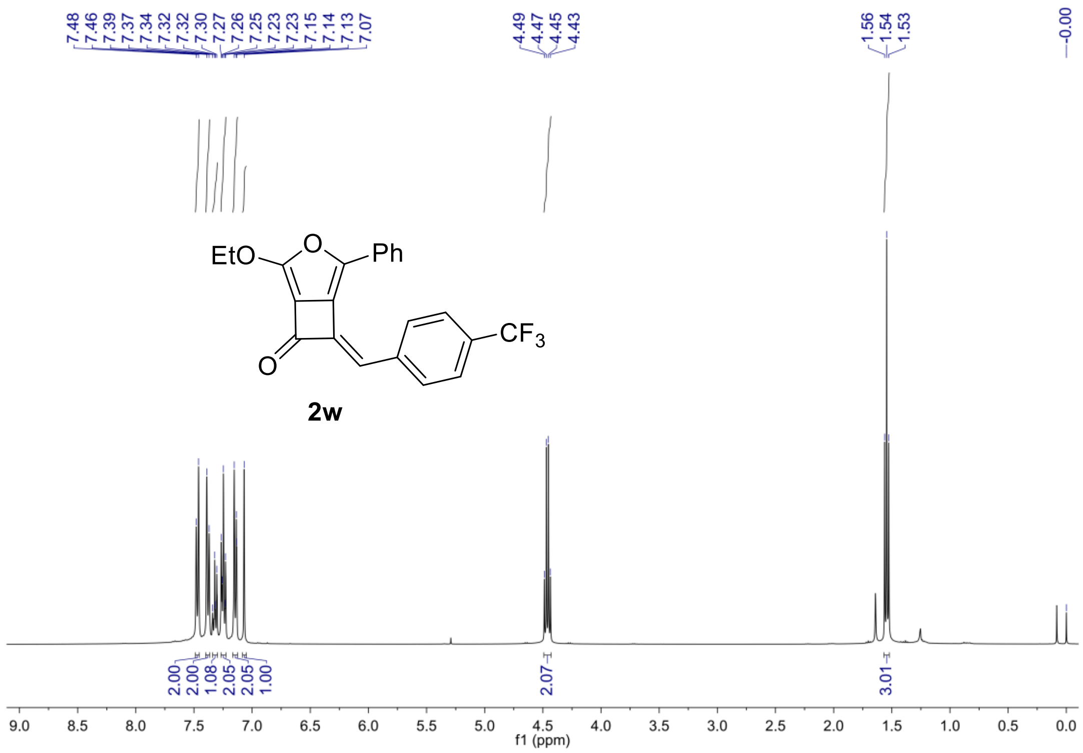

Supplementary Fig. 50 | ^{1}H NMR of compound 2w (400 MHz, at 25 ^{o}C, in $CDCl_3$).

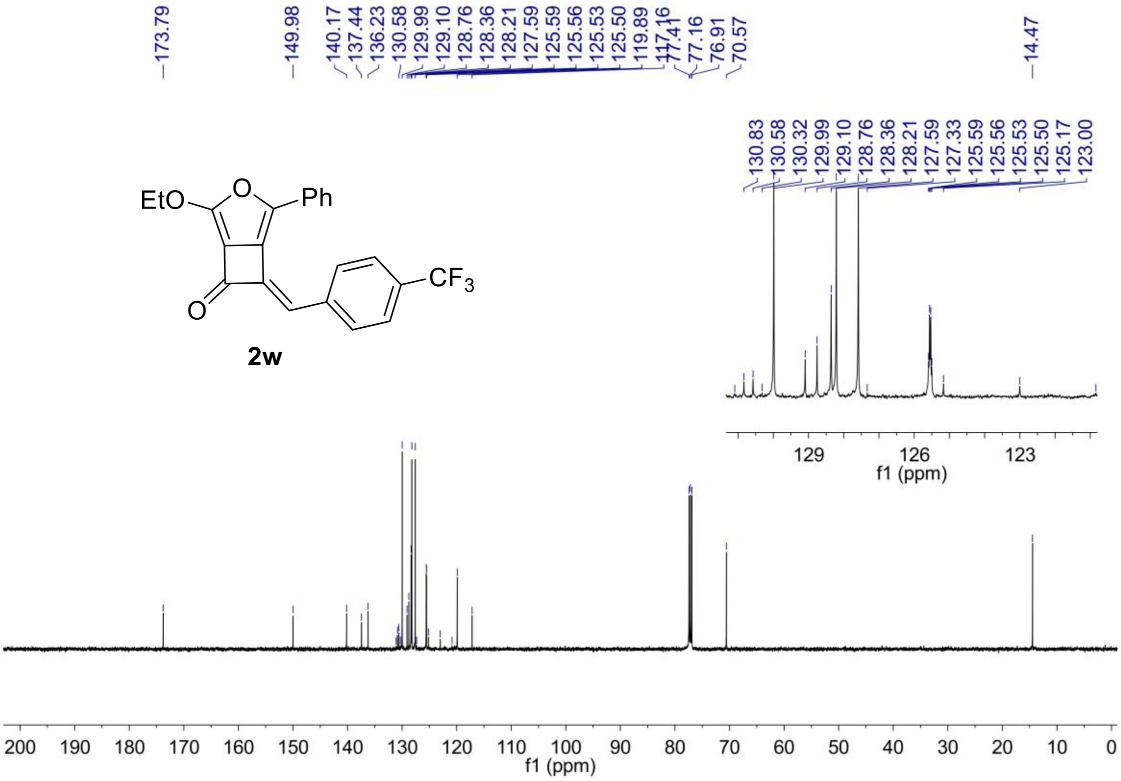

Supplementary Fig. 51 | ^{13}C NMR of compound 2w (125 MHz, at 25 ^{o}C, in $CDCl_3$).

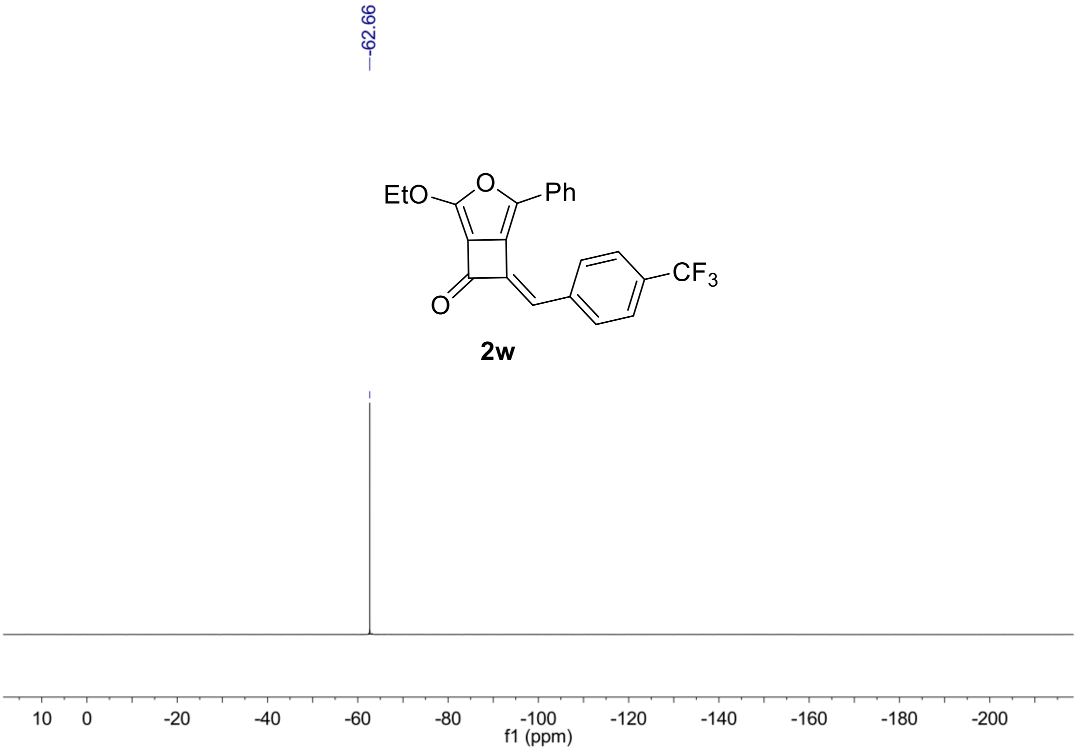

Supplementary Fig. 52 | ^{19}F NMR of compound 2w (376 MHz, at 25 ^{o}C, in $CDCl_3$).

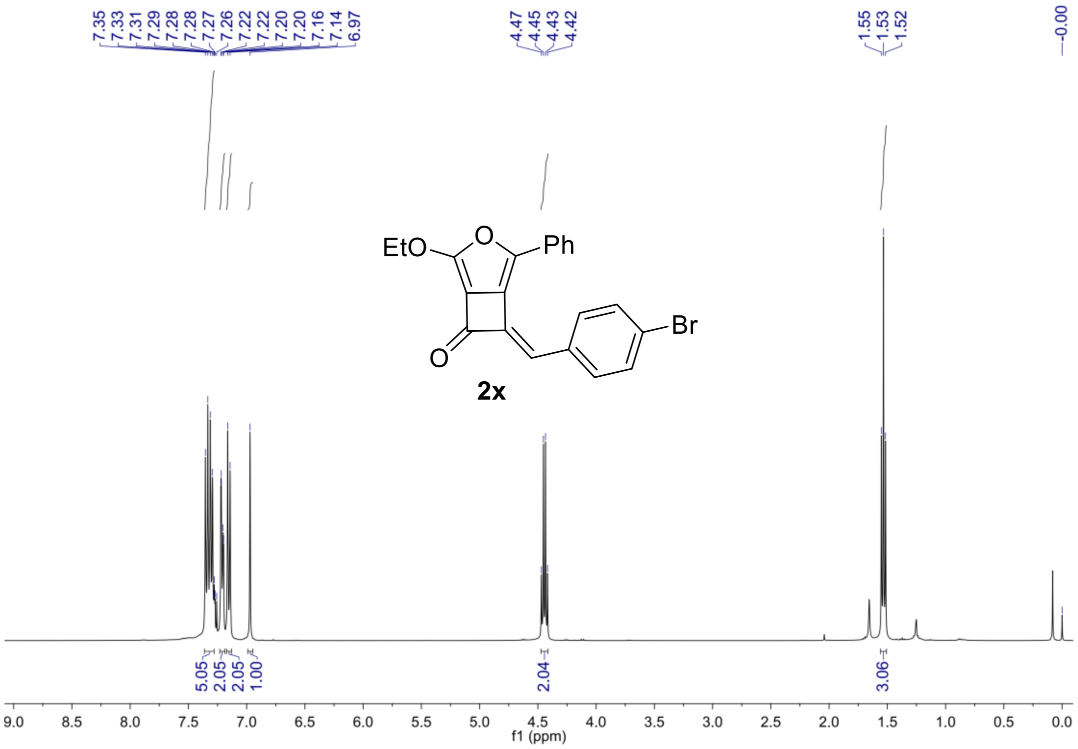

Supplementary Fig. 53 | ^{1}H NMR of compound 2x (400 MHz, at 25 °C, in $CDCl_3$).

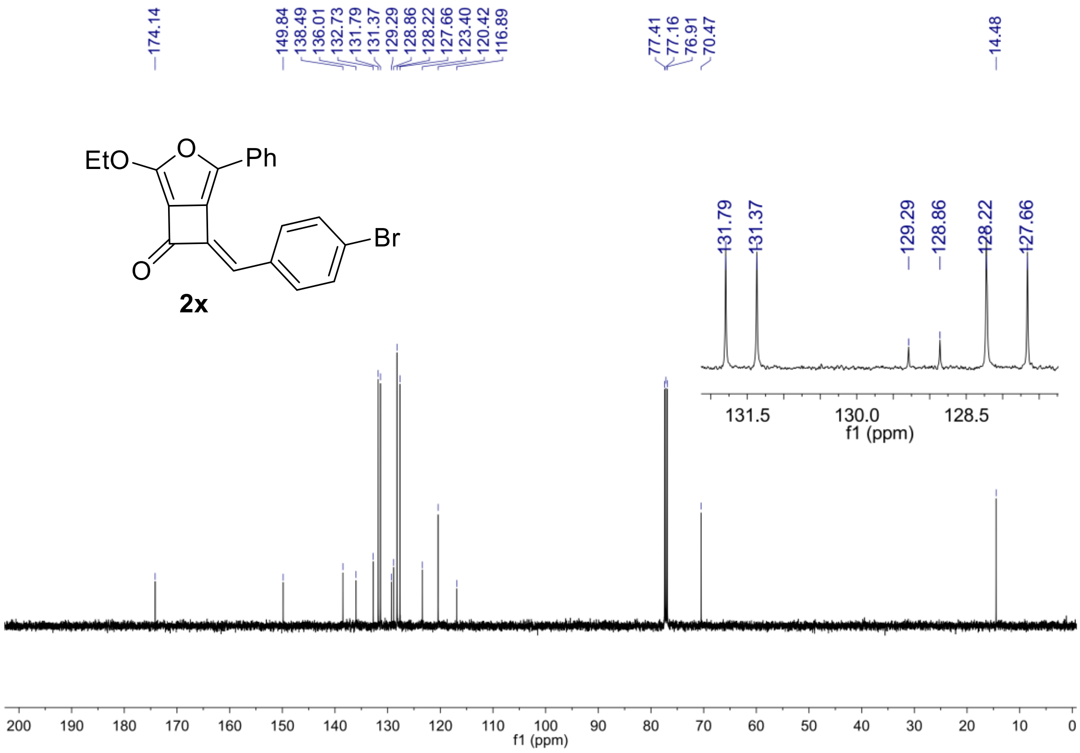

Supplementary Fig. 54 | ^{13}C NMR of compound 2x (125 MHz, at 25 °C, in $CDCl_3$).

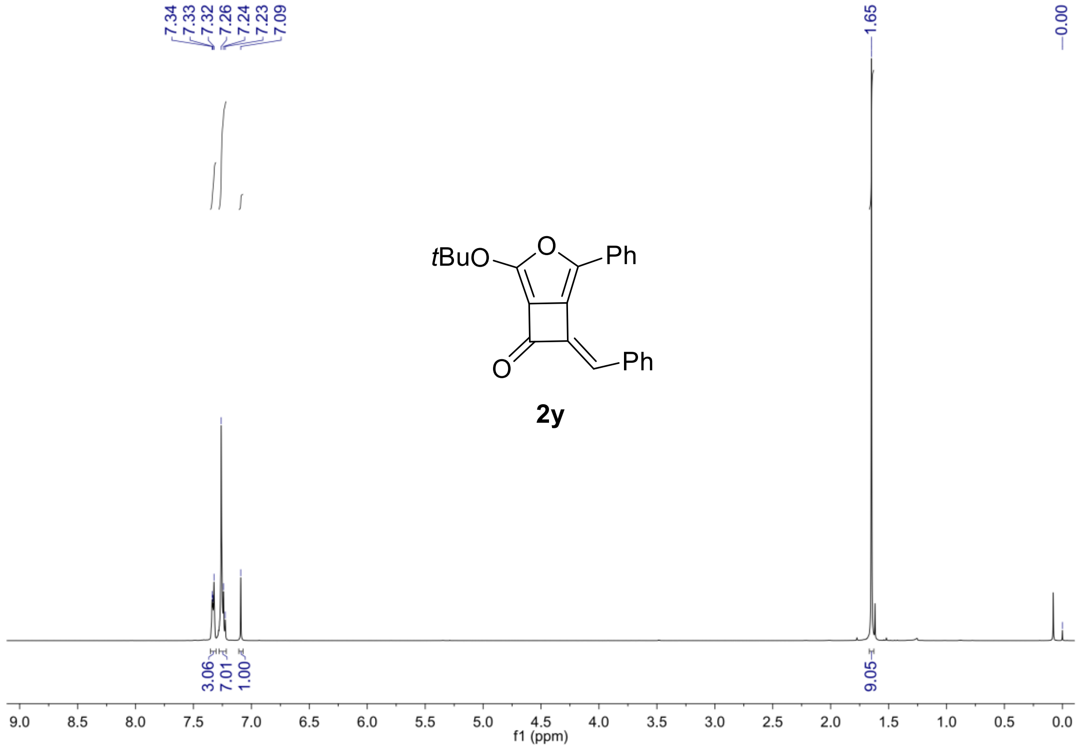

Supplementary Fig. 55 | ^{1}H NMR of compound 2y (500 MHz, at 25 °C, in $CDCl_3$).

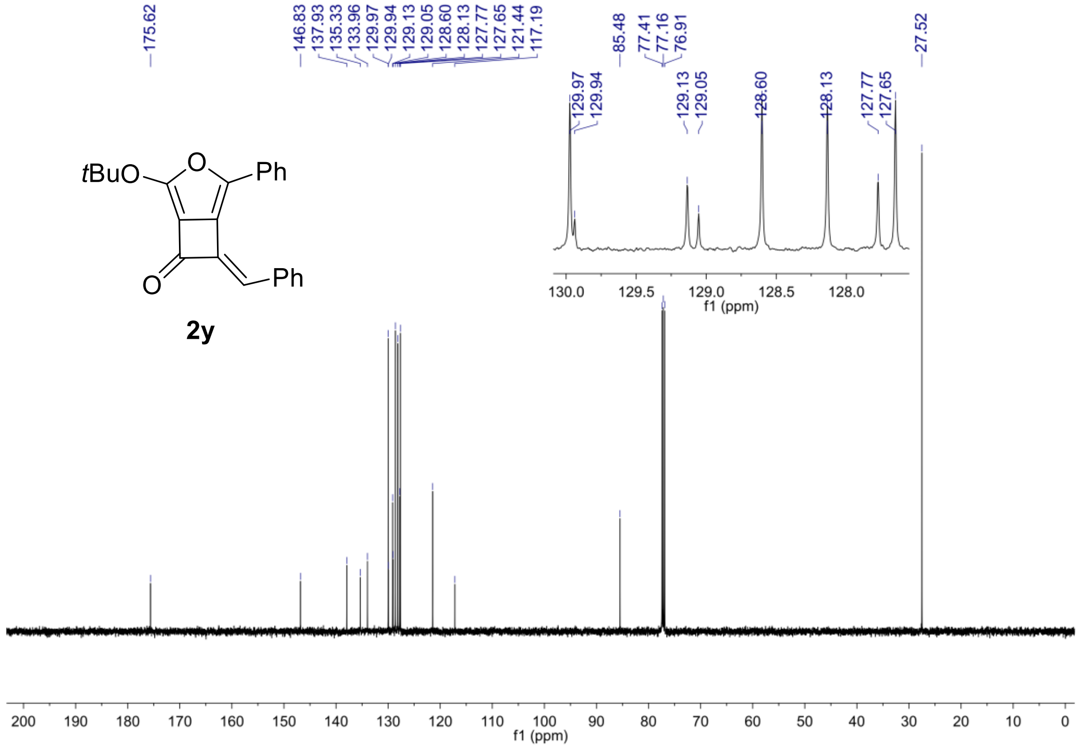

Supplementary Fig. 56 | ^{13}C NMR of compound 2y (125 MHz, at 25 °C, in $CDCl_3$).

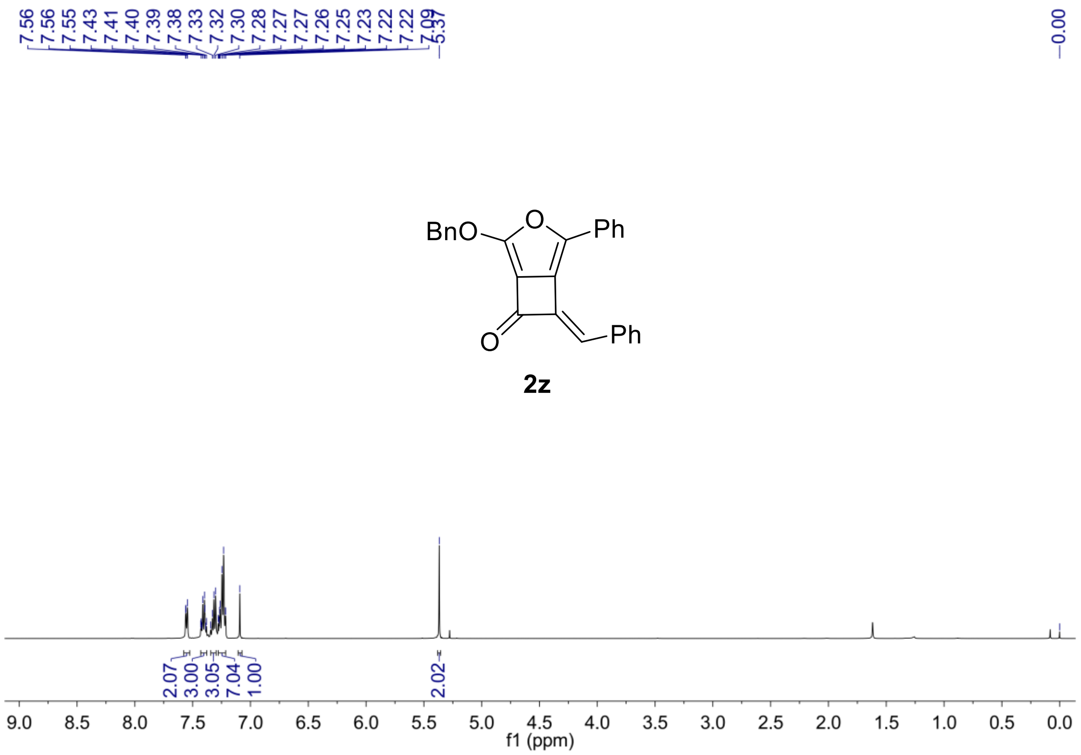

Supplementary Fig. 57 | ^{1}H NMR of compound 2z (500 MHz, at 25 °C, in $CDCl_3$).

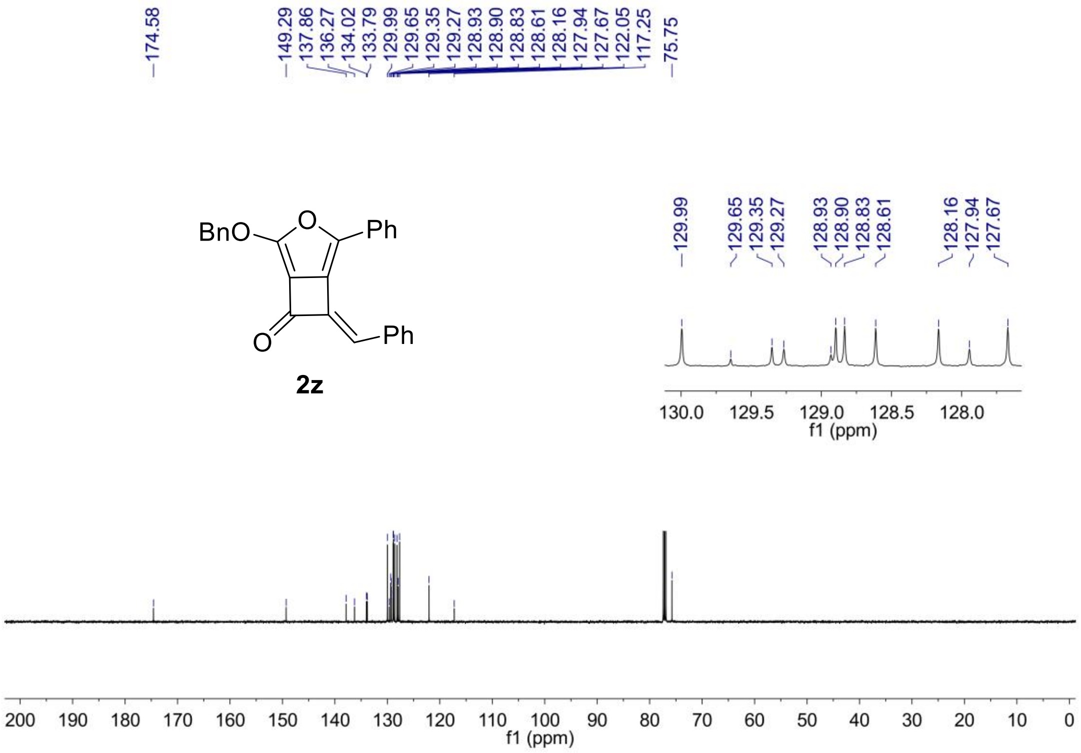

Supplementary Fig. 58 | ^{13}C NMR of compound 2z (125 MHz, at 25 °C, in $CDCl_3$).

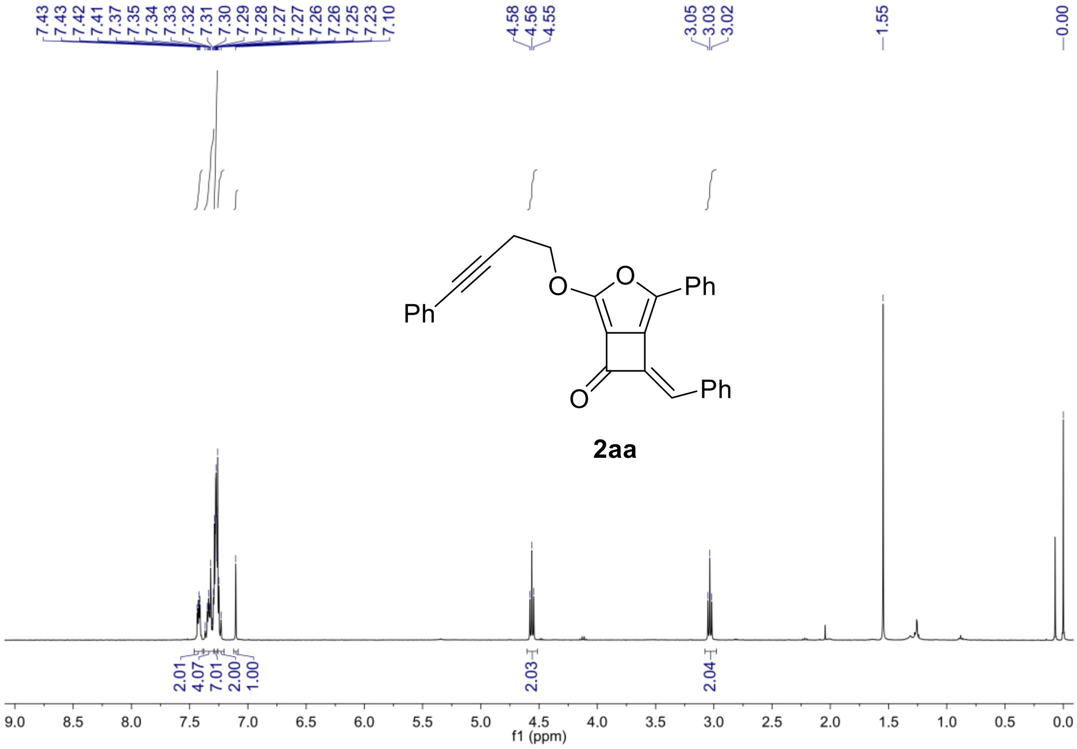

Supplementary Fig. 59 | ^{1}H NMR of compound 2aa (400 MHz, at 25 °C, in $CDCl_3$).

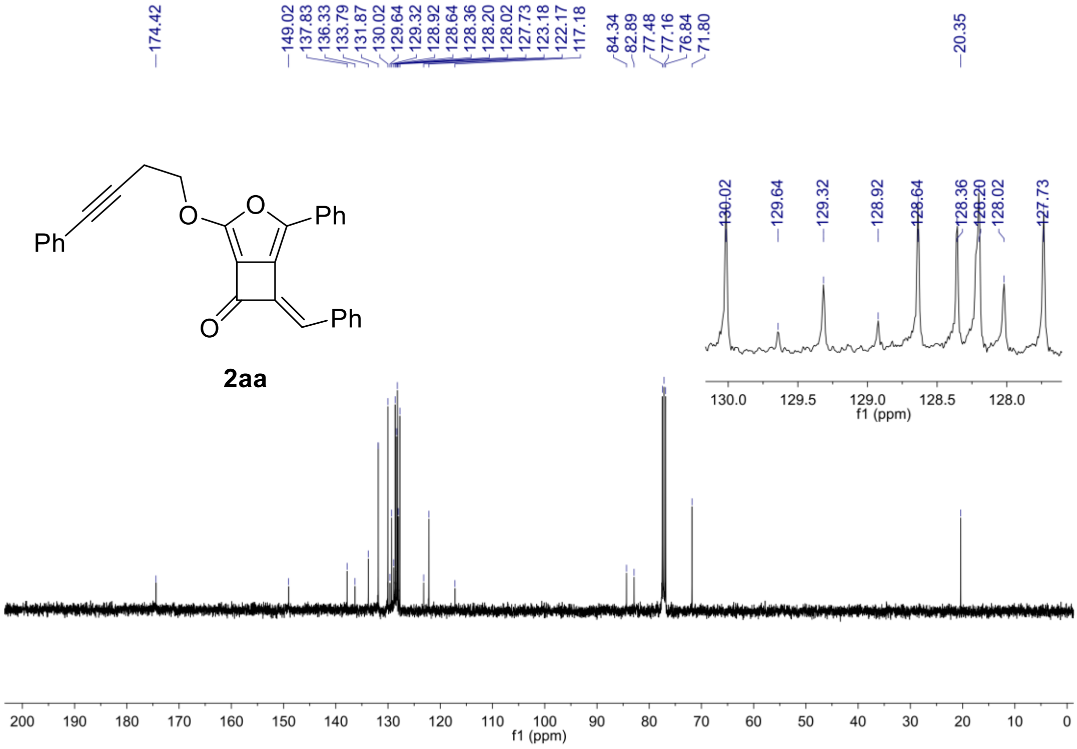

Supplementary Fig. 60 | ^{13}C NMR of compound 2aa (100 MHz, at 25 °C, in $CDCl_3$).

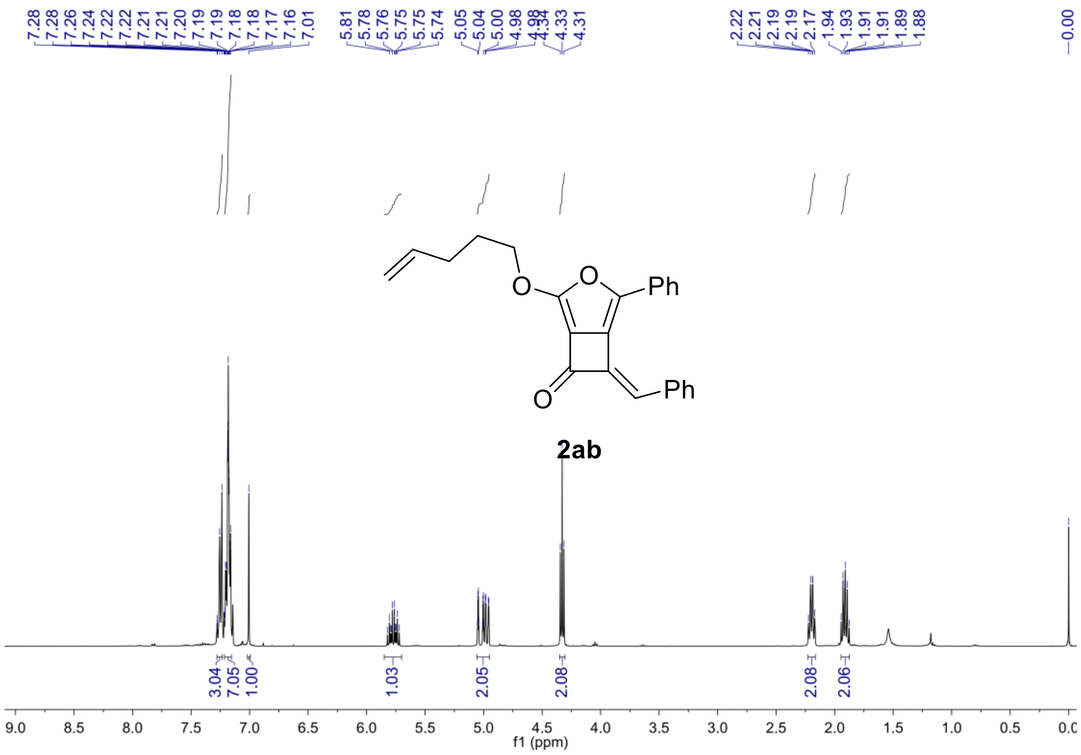

Supplementary Fig. 61 | ^{1}H NMR of compound 2ab (400 MHz, at 25 °C, in $CDCl_3$).

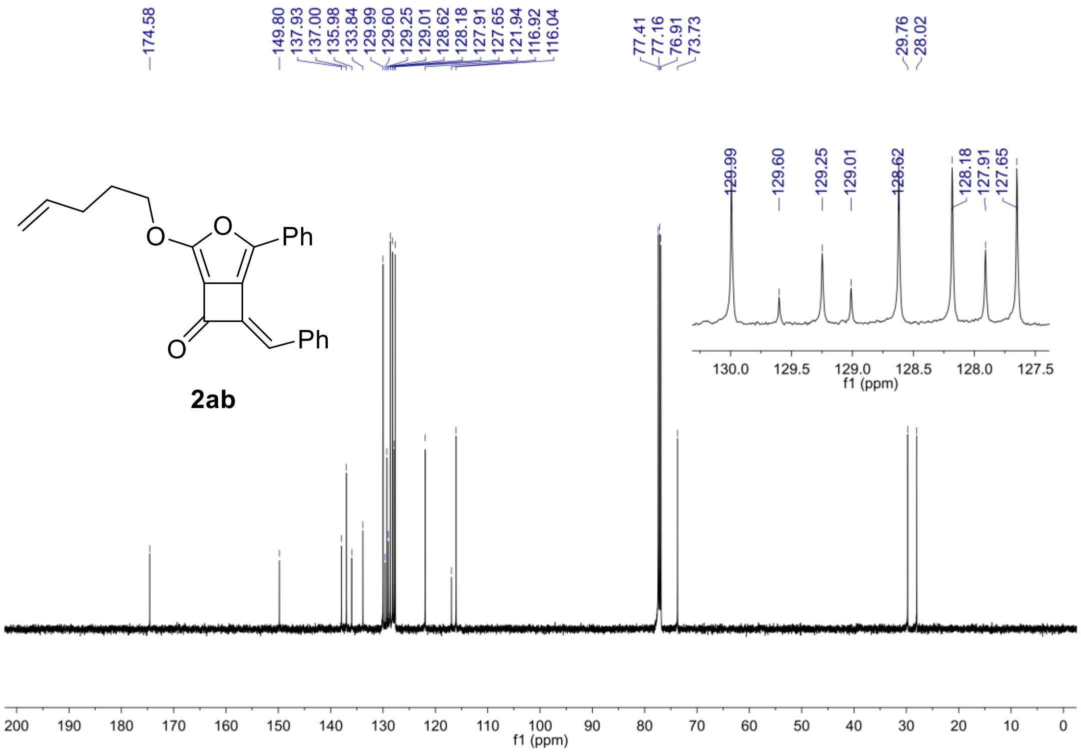

Supplementary Fig. 62 | ^{13}C NMR of compound 2ab (125 MHz, at 25 °C, in $CDCl_3$).

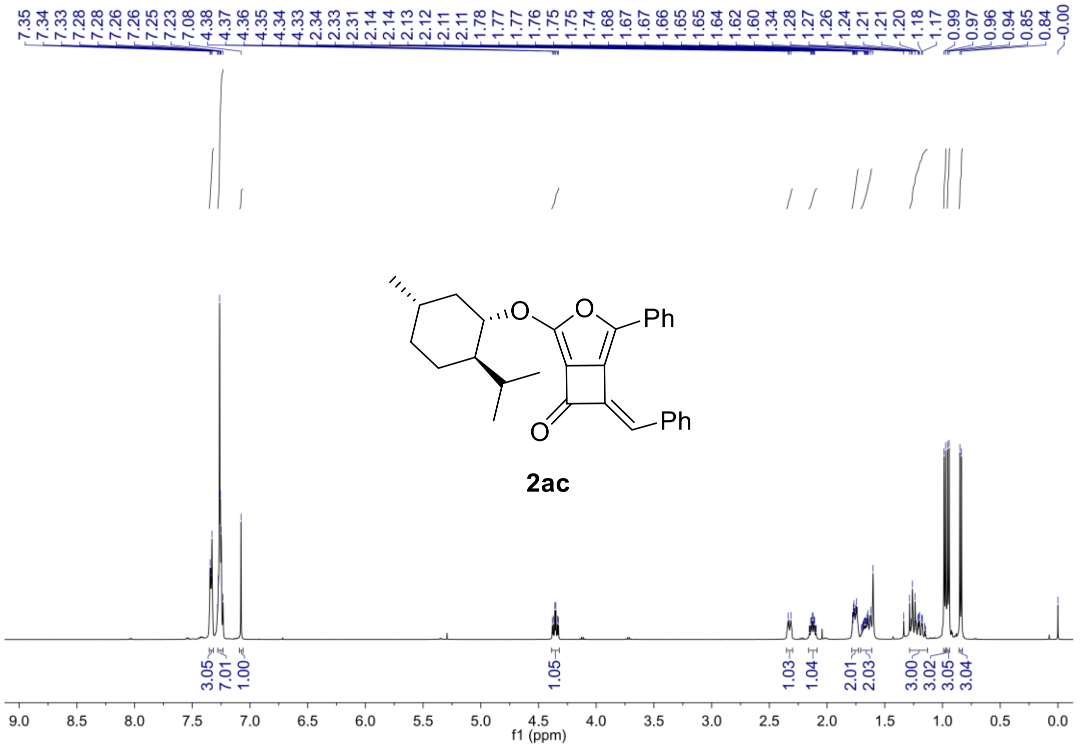

Supplementary Fig. 63 | ^{1}H NMR of compound 2ac (500 MHz, at 25 °C, in $CDCl_3$).

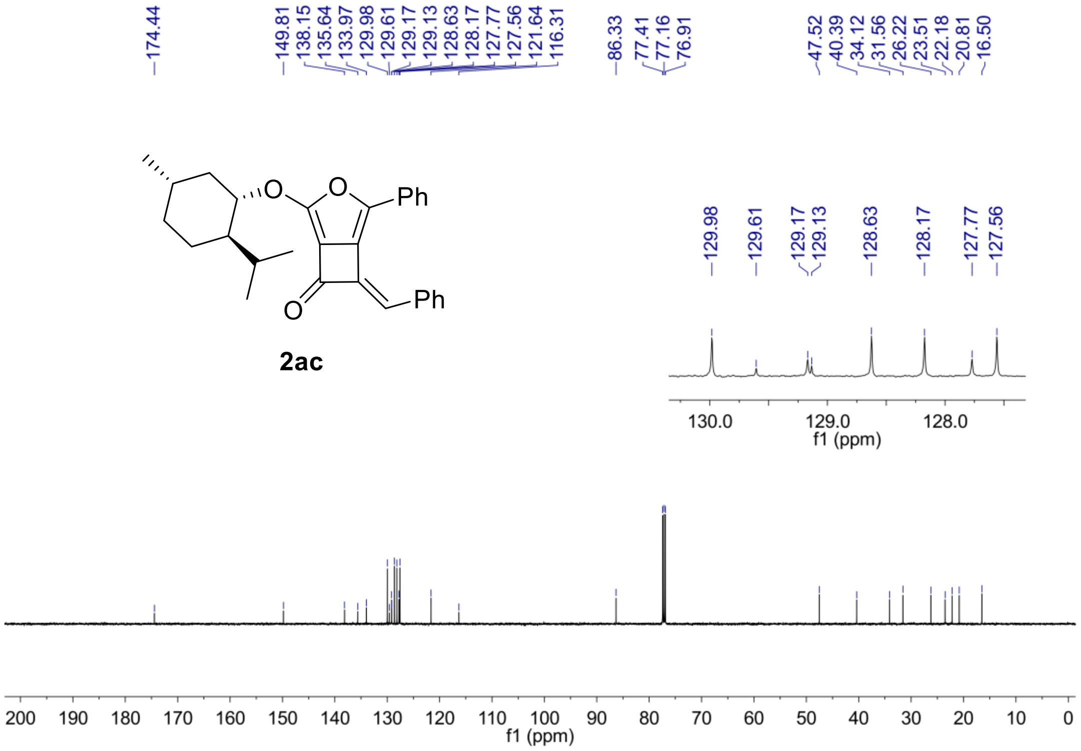

Supplementary Fig. 64 | ^{13}C NMR of compound 2ac (125 MHz, at 25 °C, in $CDCl_3$).

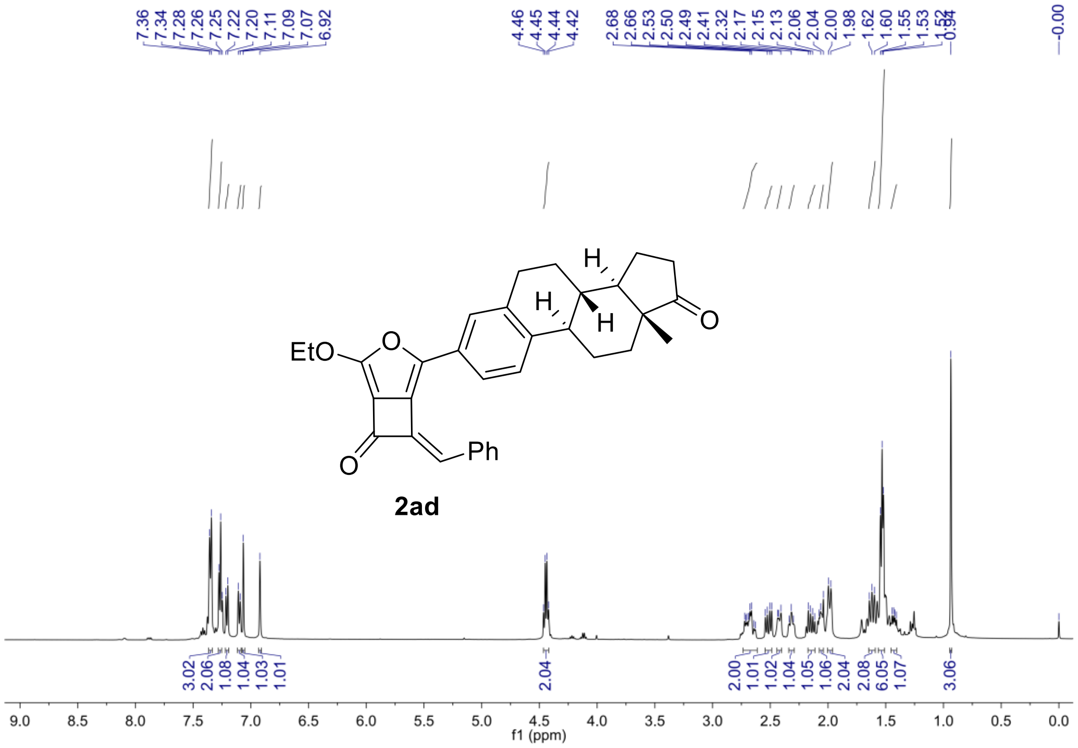

Supplementary Fig. 65 | ^{1}H NMR of compound 2ad (500 MHz, at 25 ºC, in $CDCl_3$).

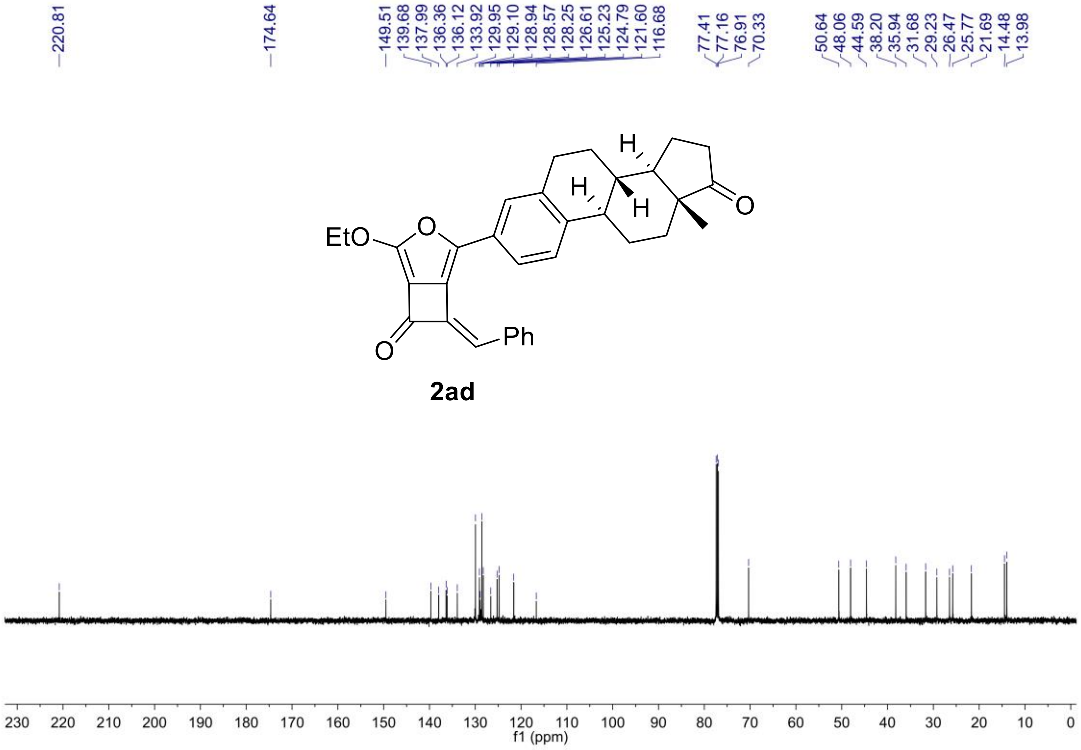

Supplementary Fig. 66 | ^{13}C NMR of compound 2ad (125 MHz, at 25 ºC, in $CDCl_3$).

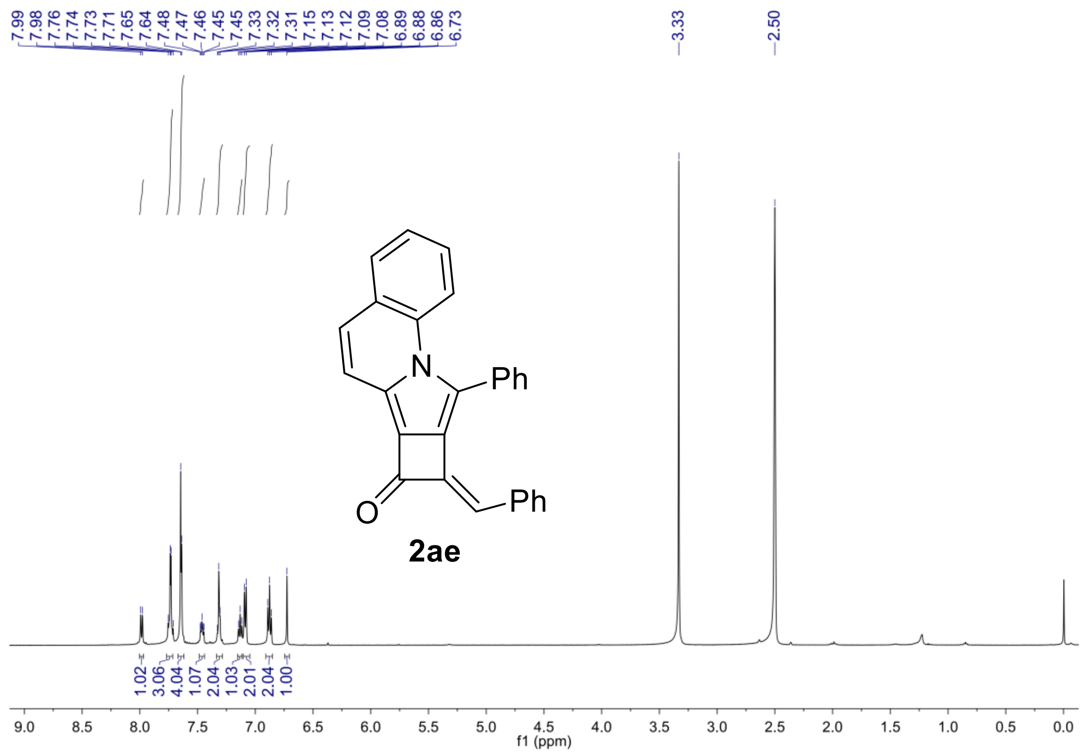

Supplementary Fig. 67 | ^{1}H NMR of compound 2ae (500 MHz, at 25 °C, in DMSO).

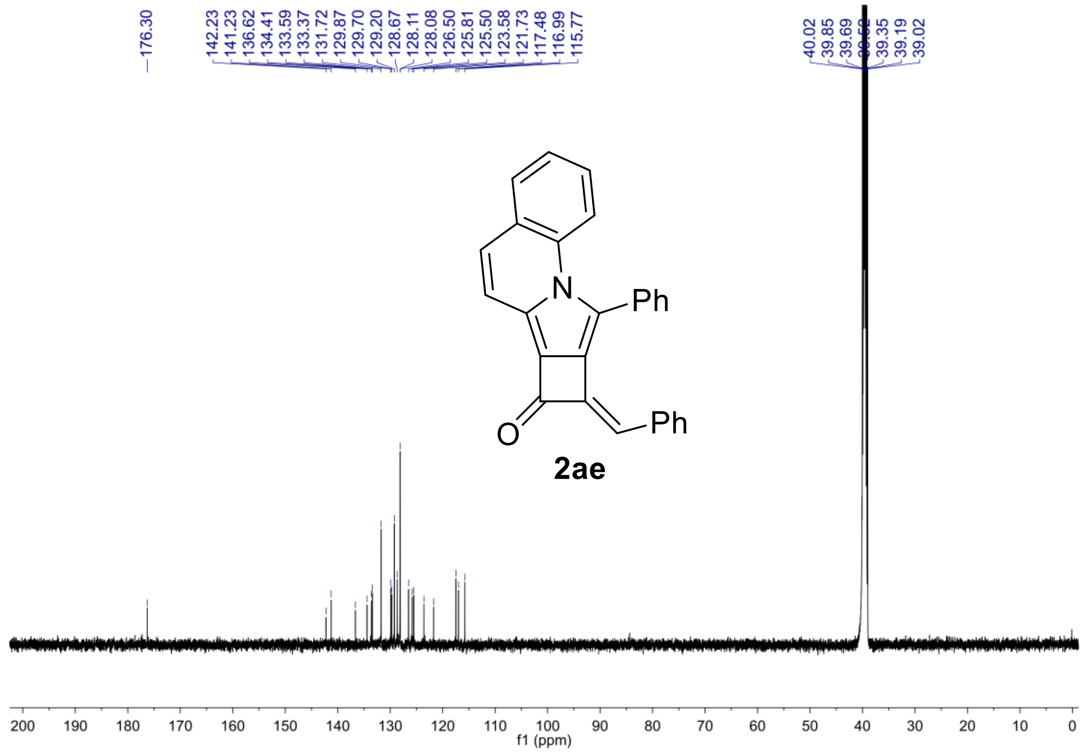

Supplementary Fig. 68 | ^{13}C NMR of compound 2ae (125 MHz, at 25 °C, in DMSO).

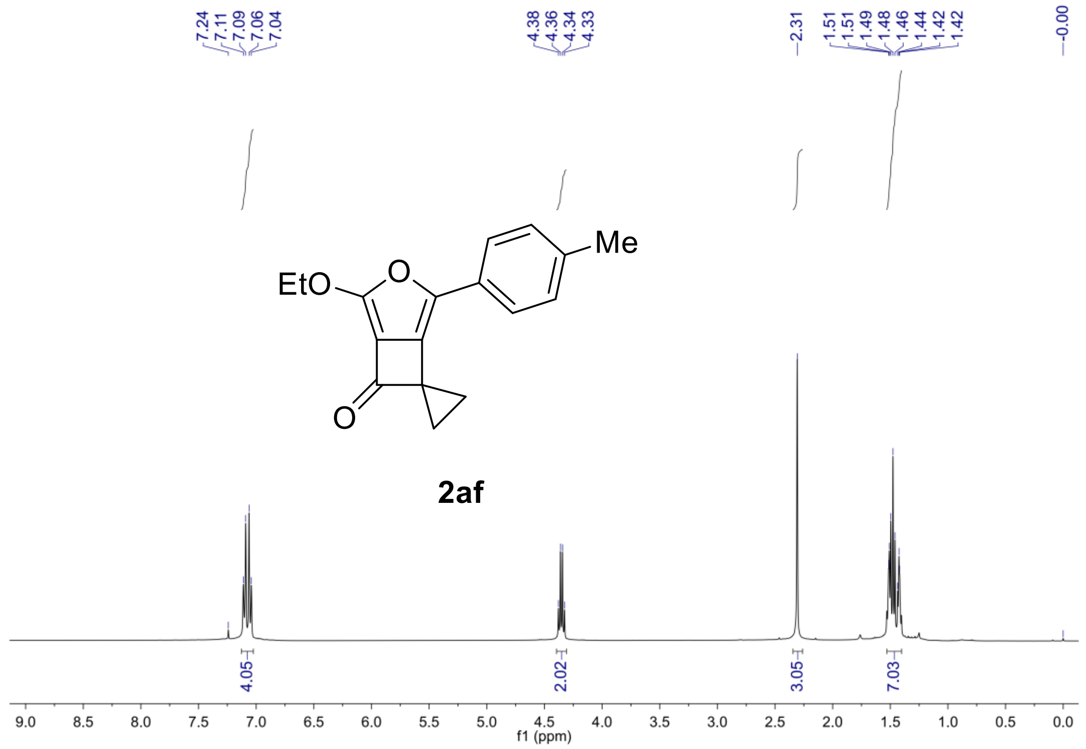

Supplementary Fig. 69 | ^{1}H NMR of compound 2af (400 MHz, at 25 °C, in $CDCl_3$).

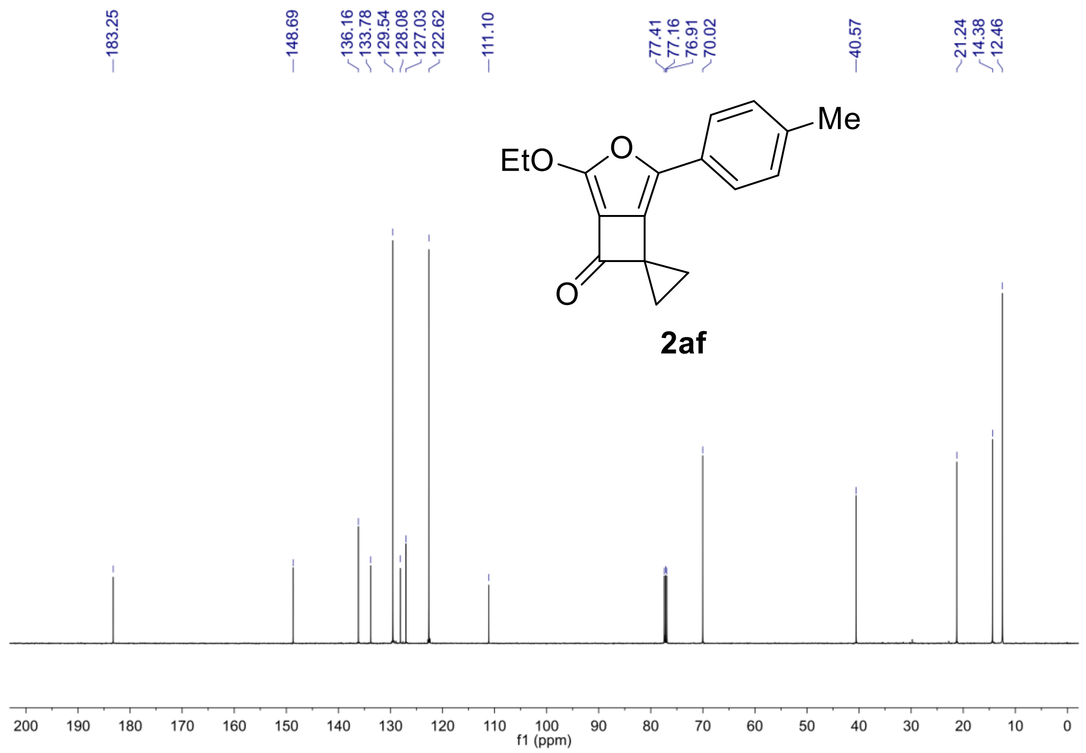

Supplementary Fig. 70 | ^{13}C NMR of compound 2af (125 MHz, at 25 °C, in $CDCl_3$).

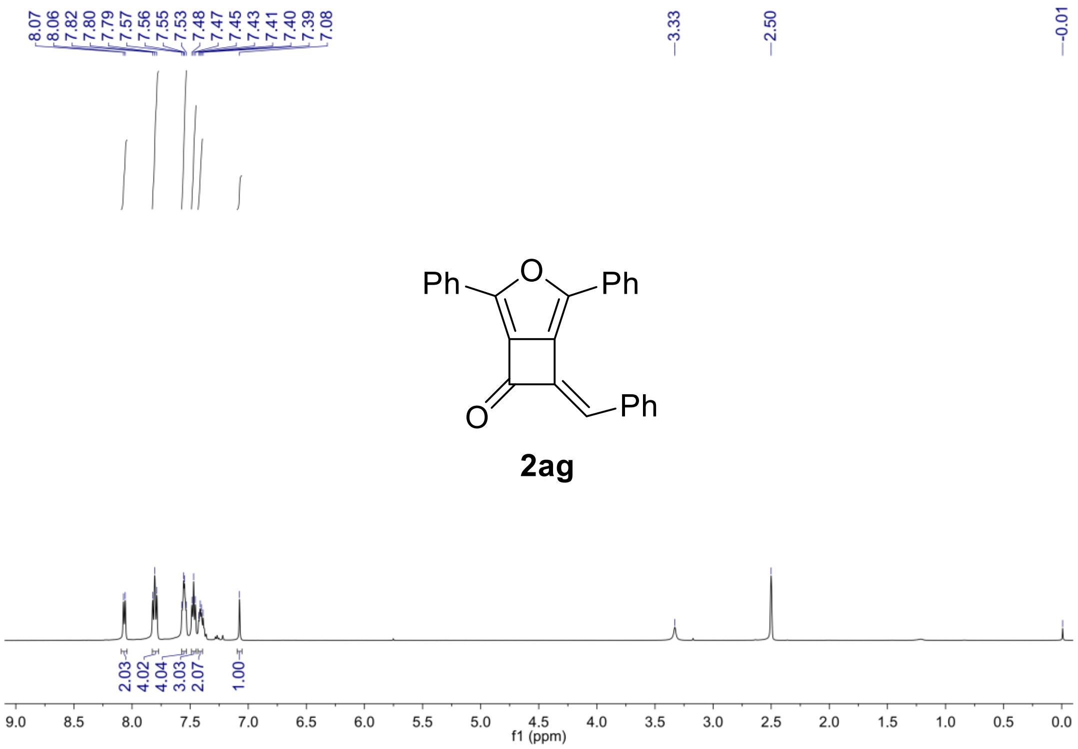

Supplementary Fig. 71 | ^{1}H NMR of compound 2ag (500 MHz, at 25 ^{o}C, in DMSO).

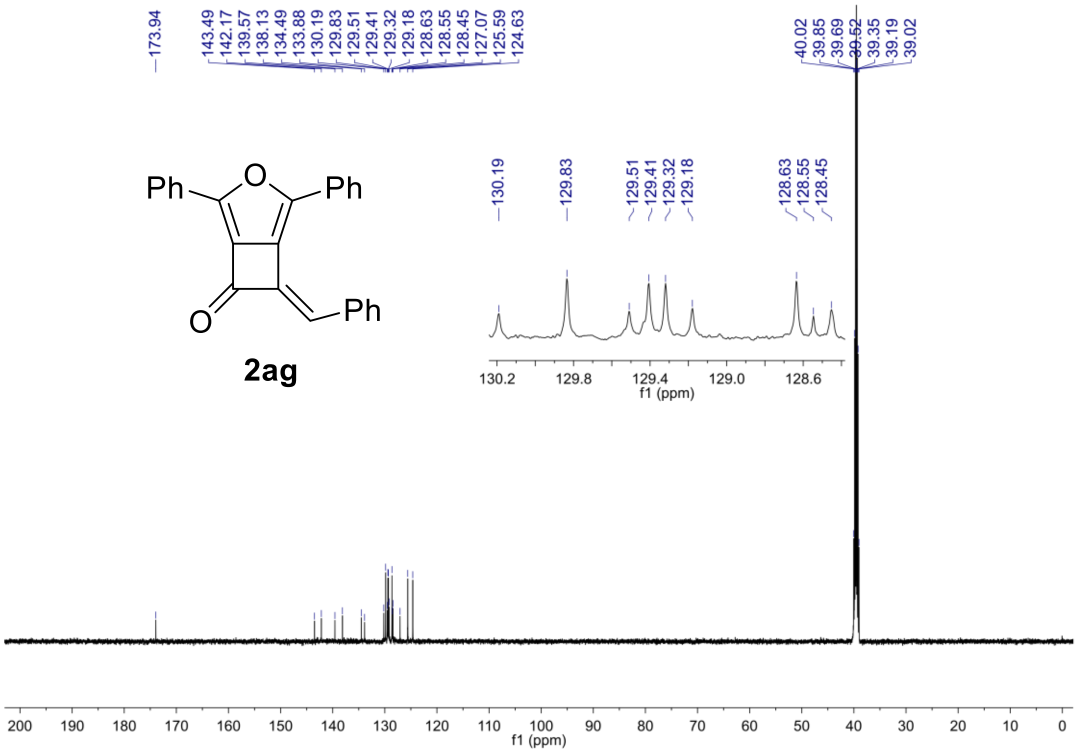

Supplementary Fig. 72 | ^{13}C NMR of compound 2ag (125 MHz, at 25 ^{o}C, in DMSO).

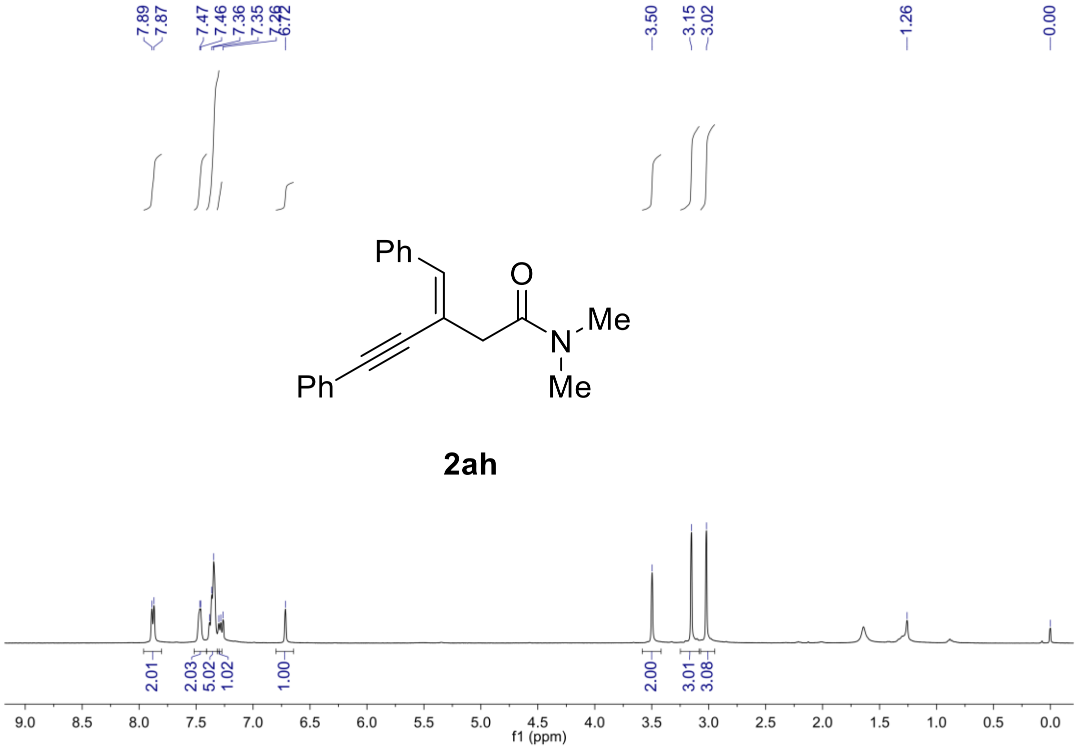

Supplementary Fig. 73 | ^{1}H NMR of compound 2ah (400 MHz, at 25 °C, in $CDCl_3$).

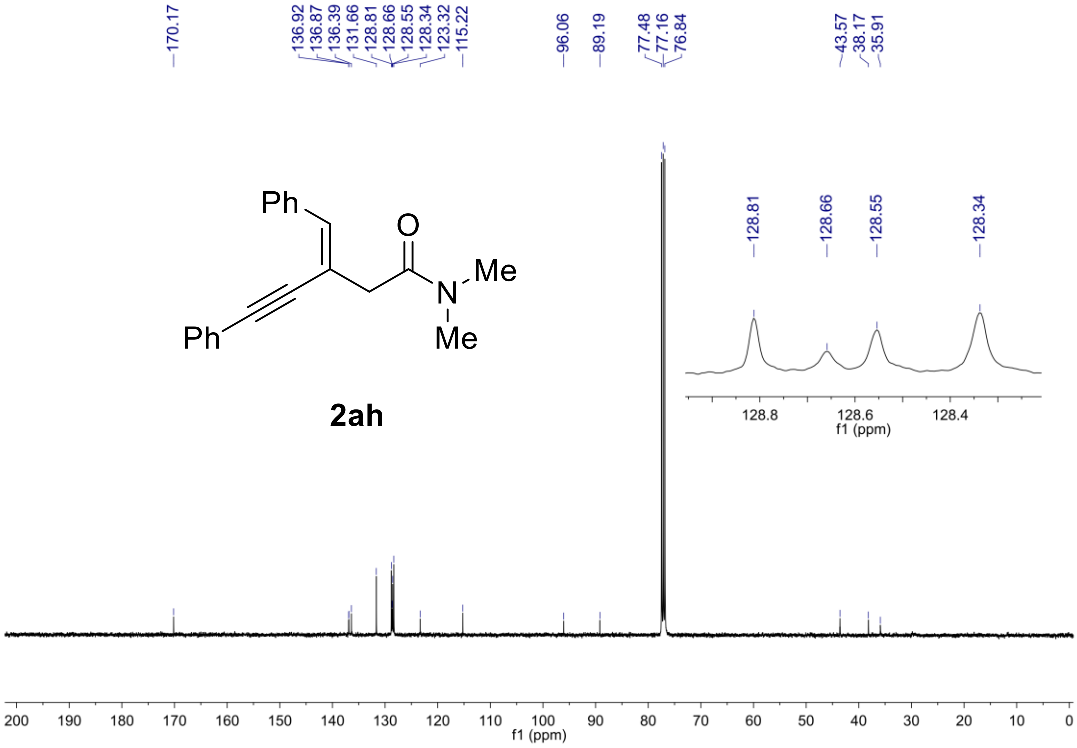

Supplementary Fig. 74 | ^{13}C NMR of compound 2ah (100 MHz, at 25 °C, in $CDCl_3$).

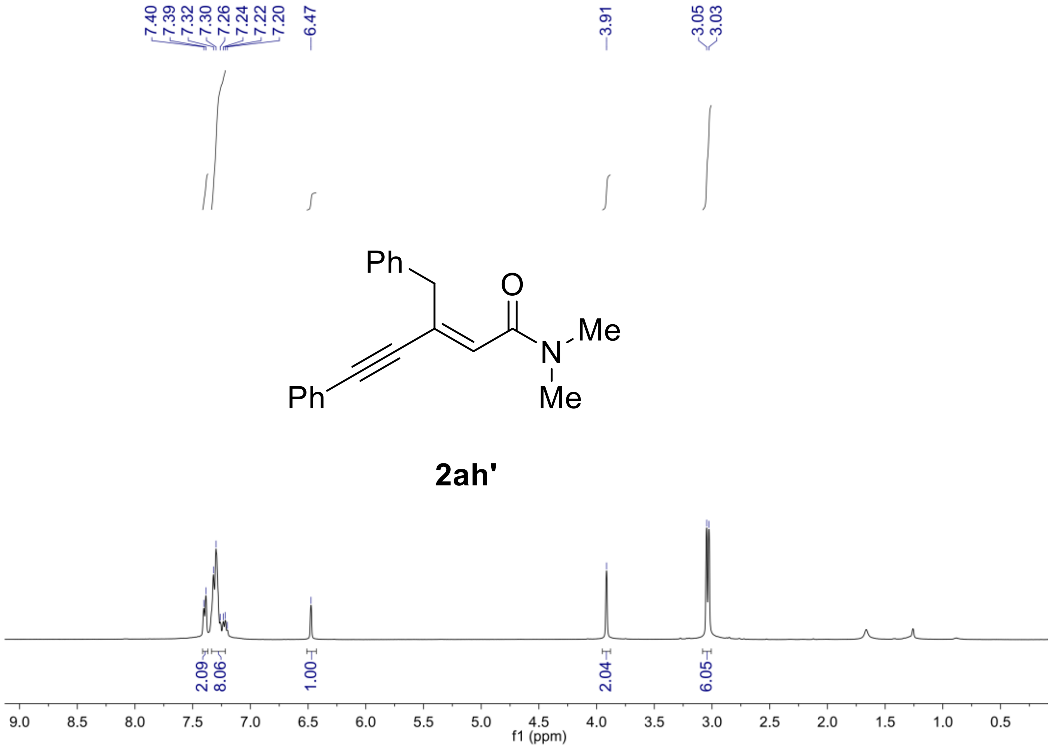

Supplementary Fig. 75 | ^{1}H NMR of compound 2ah' (400 MHz, at 25 ^{o}C, in $CDCl_3$).

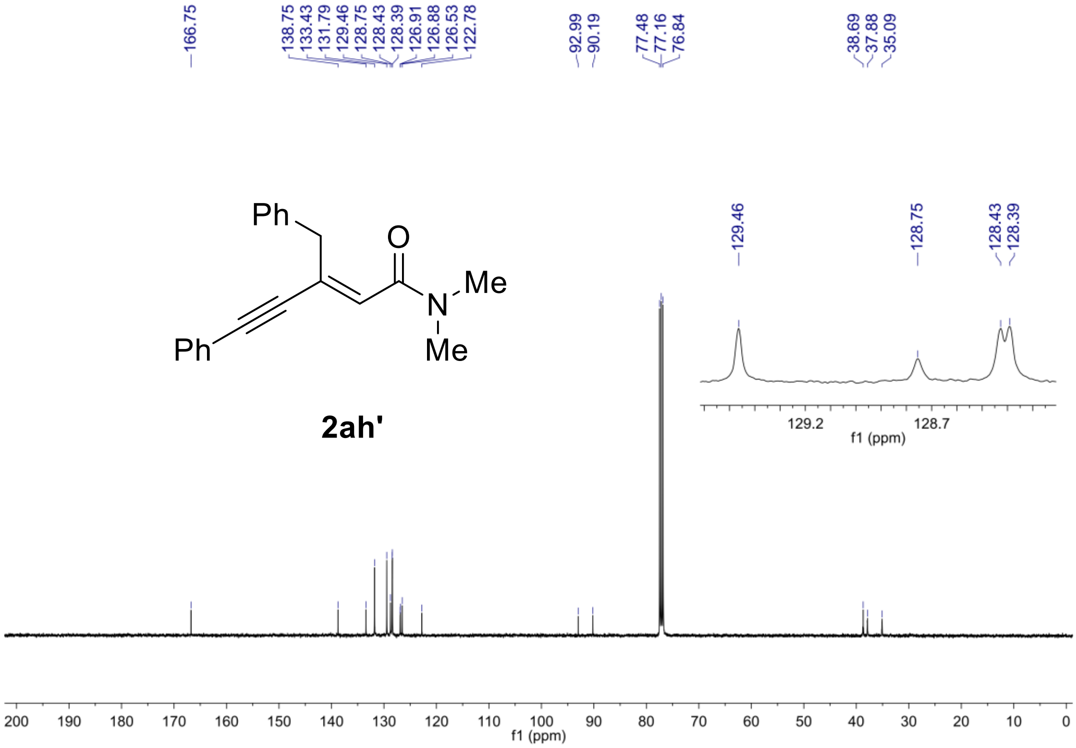

Supplementary Fig. 76 | ^{13}C NMR of compound 2ah' (100 MHz, at 25 ^{o}C, in $CDCl_3$).

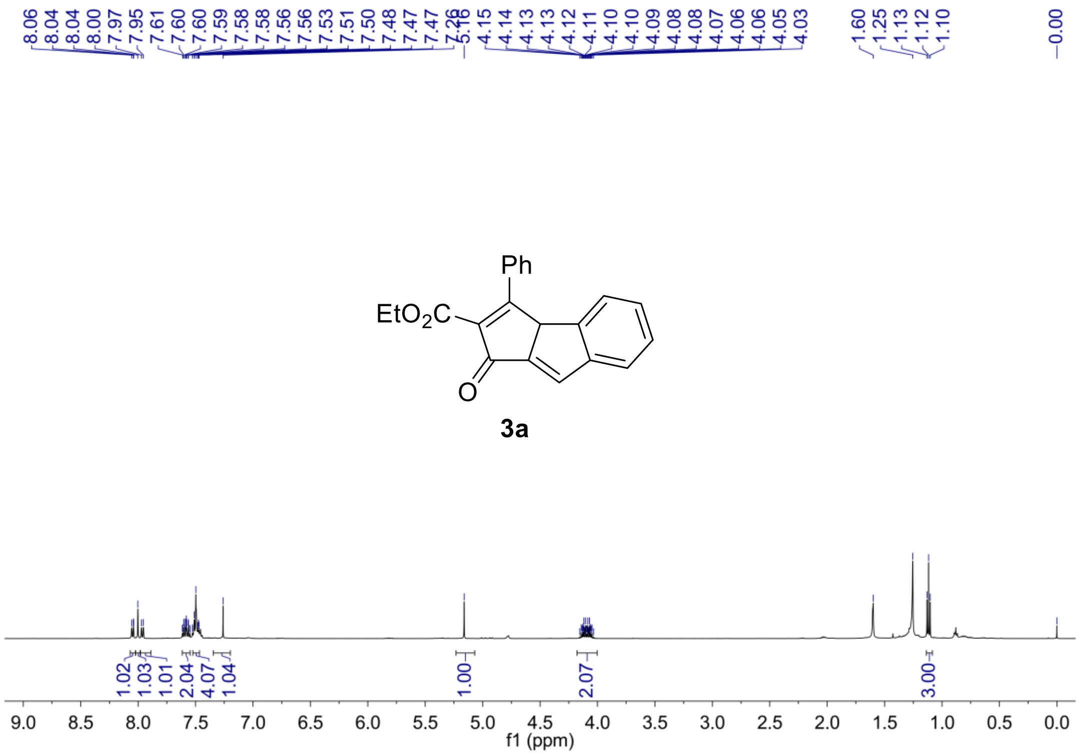

Supplementary Fig. 77 | ^{1}H NMR of compound 3a (500 MHz, at 25 °C, in $CDCl_3$).

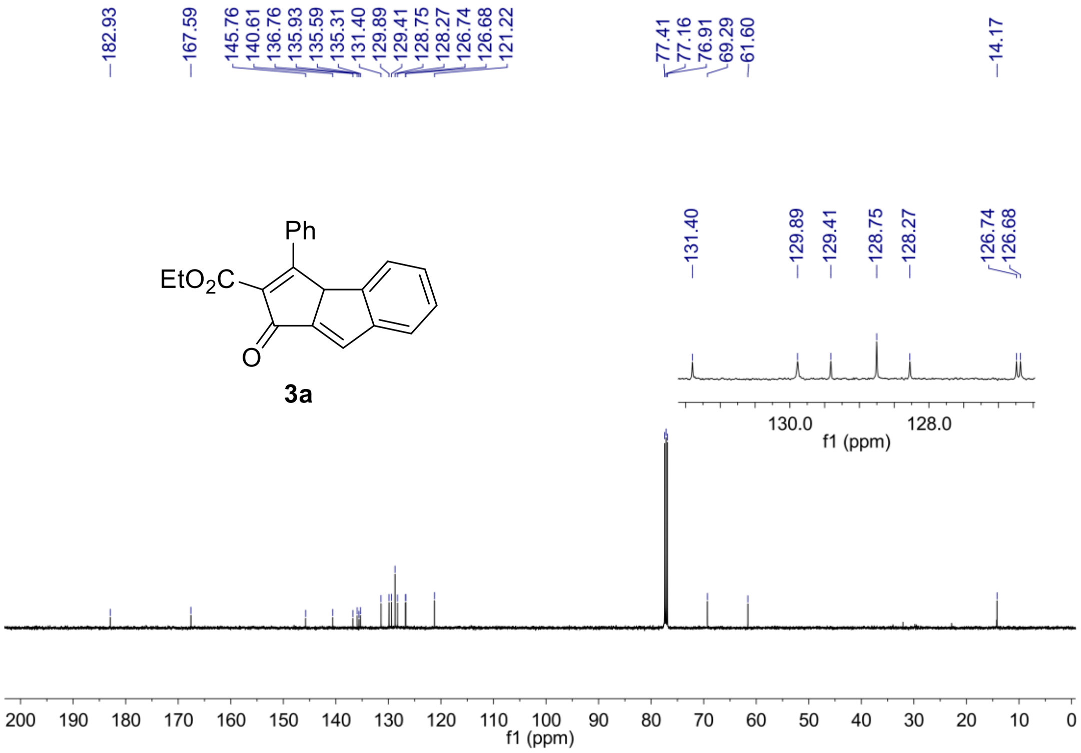

Supplementary Fig. 78 | ^{13}C NMR of compound 3a (125 MHz, at 25 °C, in $CDCl_3$).

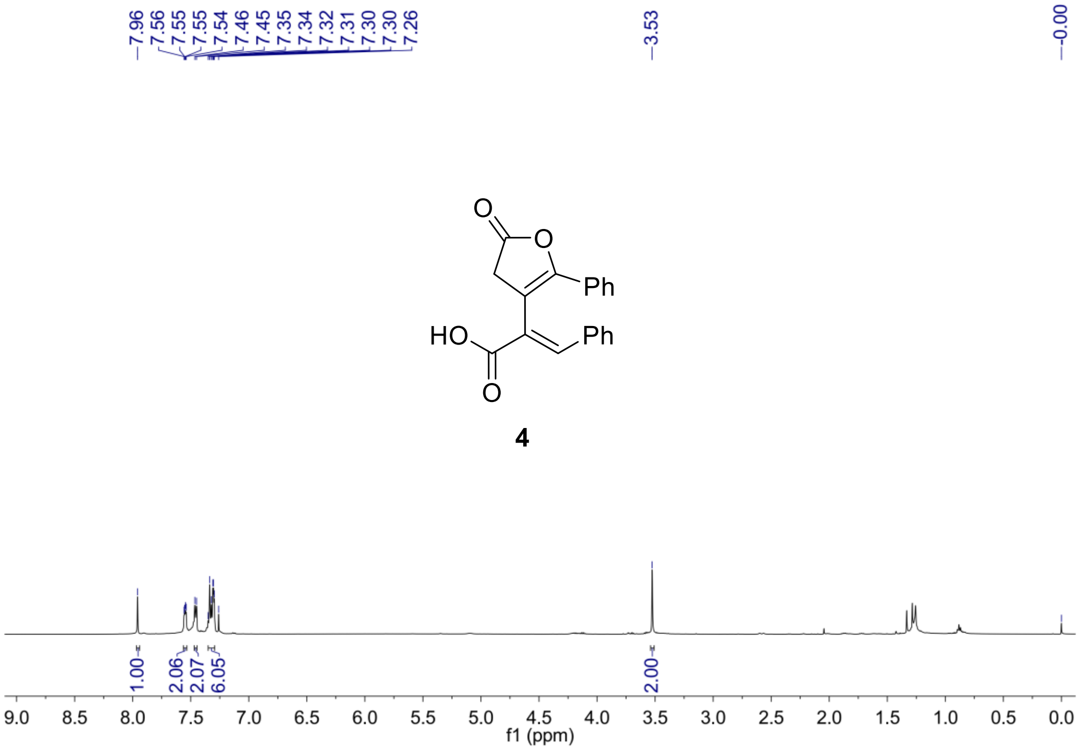

Supplementary Fig. 79 | ^{1}H NMR of compound 4 (400 MHz, at 25 °C, in $CDCl_3$).

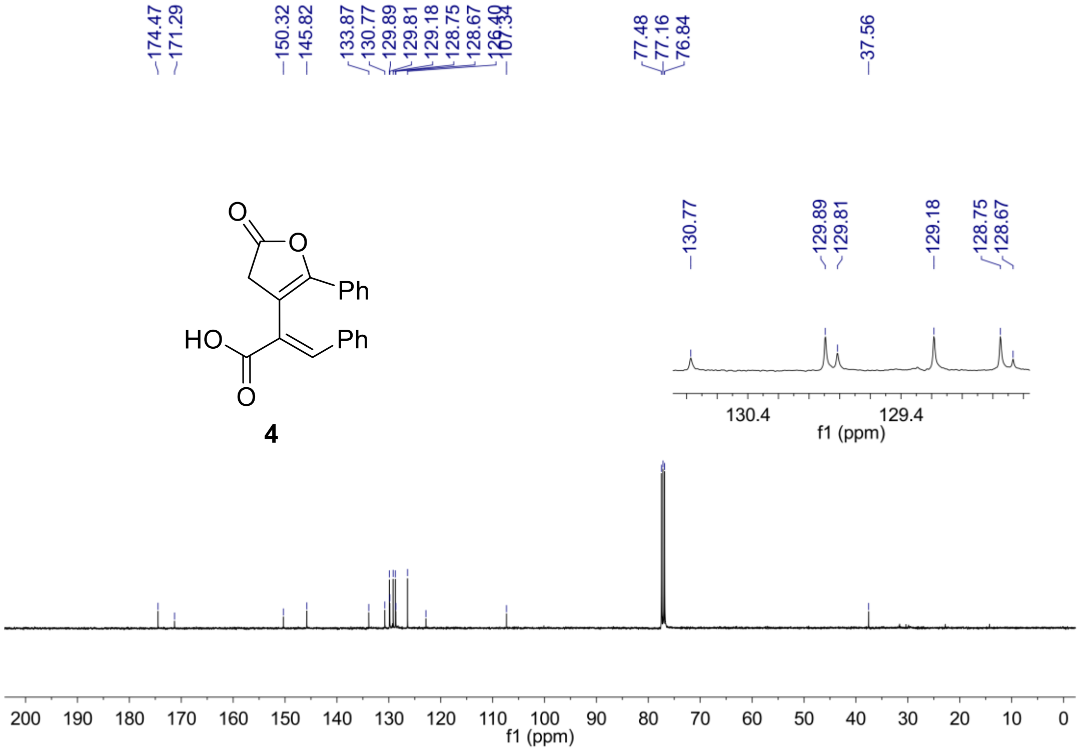

Supplementary Fig. 80 | ^{13}C NMR of compound 4 (100 MHz, at 25 °C, in $CDCl_3$).

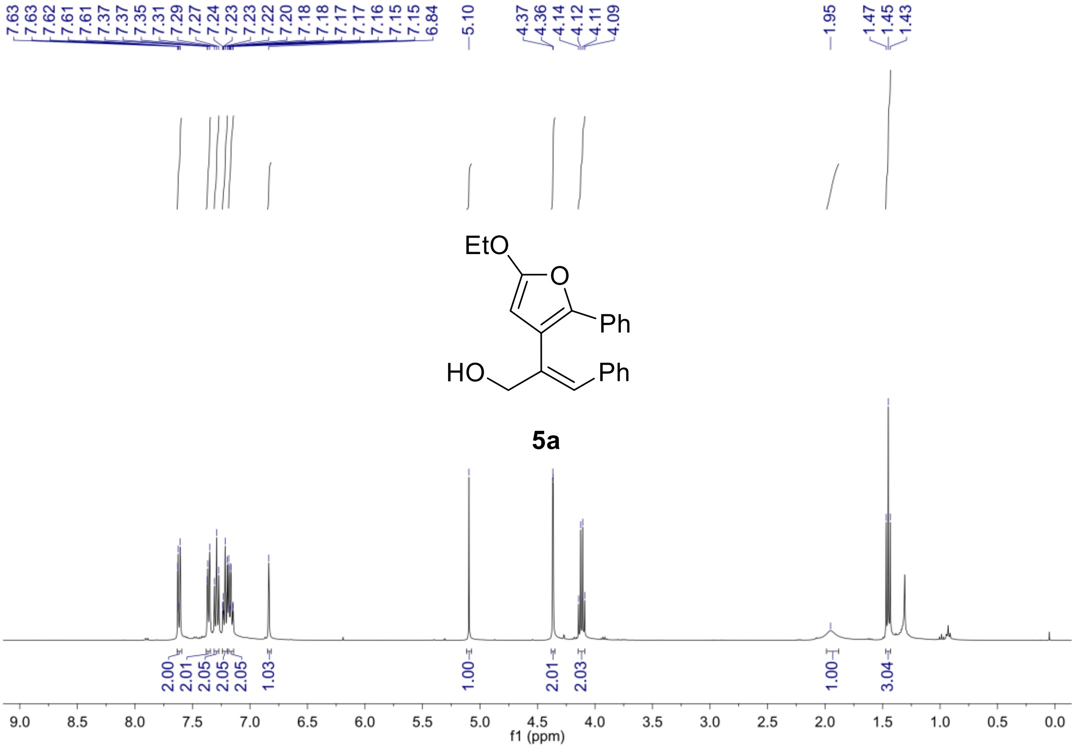

Supplementary Fig. 81 | ^{1}H NMR of compound 5a (400 MHz, at 25 °C, in $CDCl_3$).

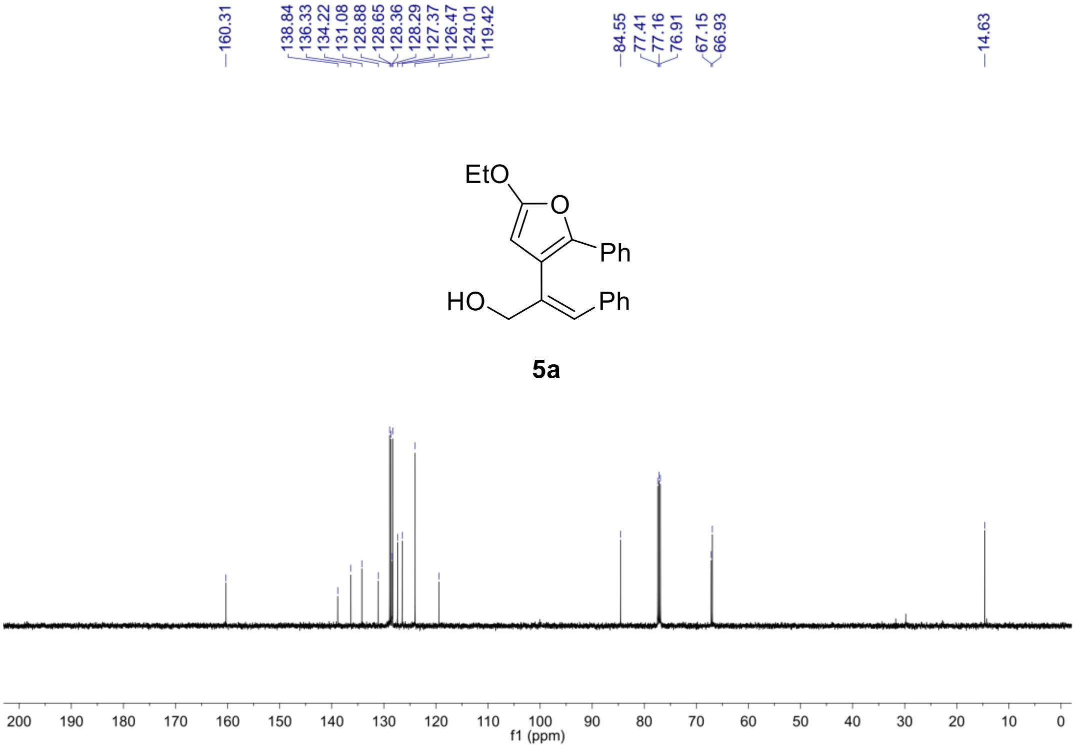

Supplementary Fig. 82 | ^{13}C NMR of compound 5a (125 MHz, at 25 °C, in $CDCl_3$).

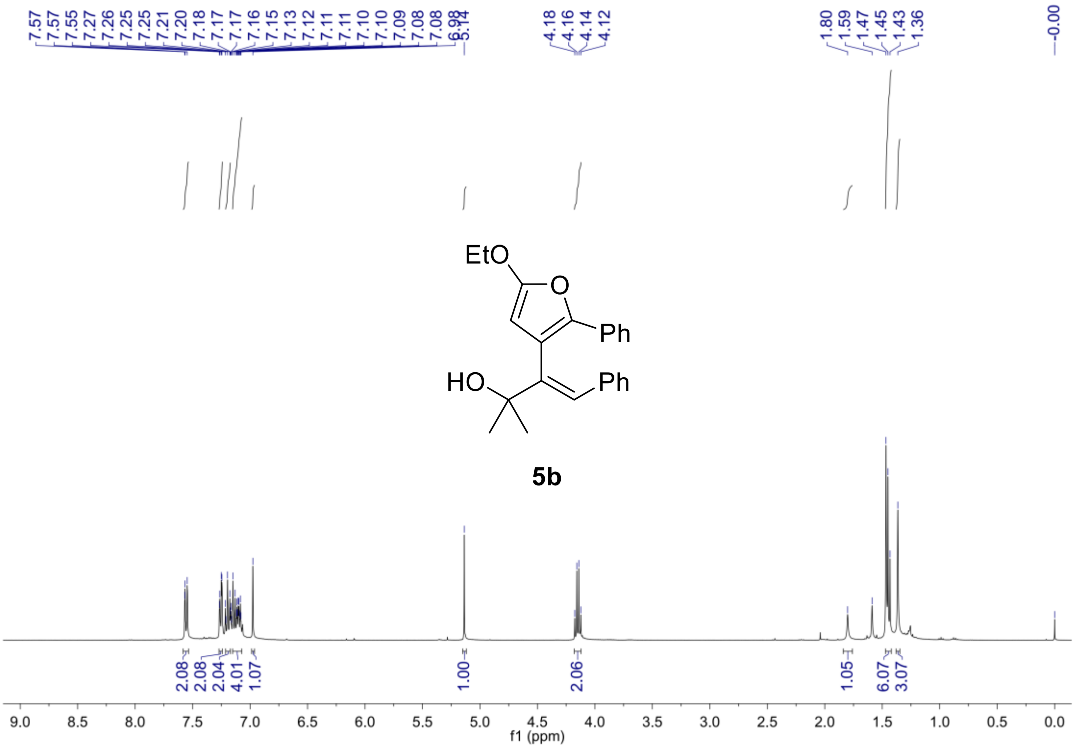

Supplementary Fig. 83 | ^{1}H NMR of compound 5b (400 MHz, at 25 °C, in $CDCl_3$).

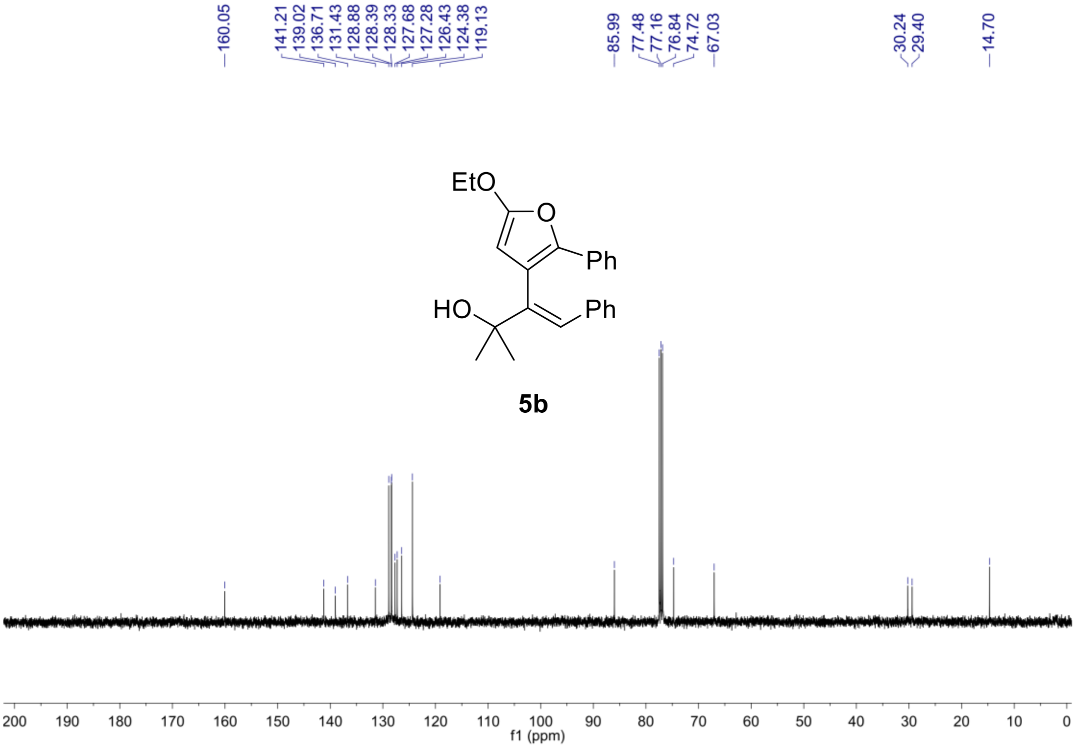

Supplementary Fig. 84 | ^{13}C NMR of compound 5b (100 MHz, at 25 °C, in $CDCl_3$).

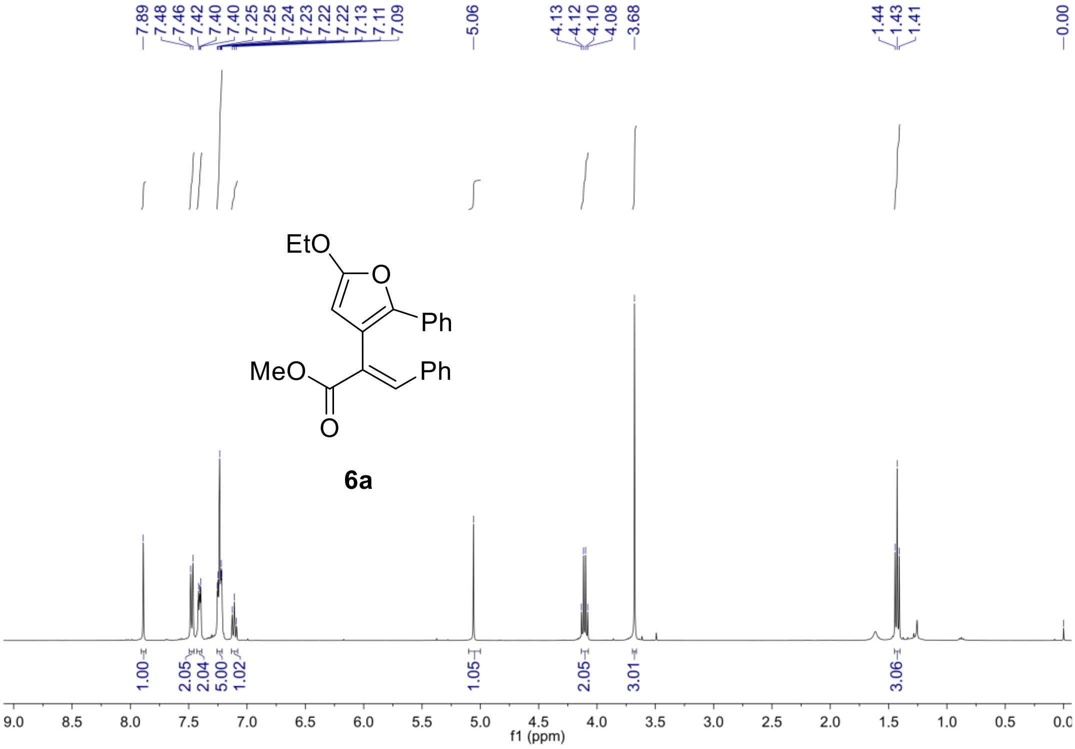

Supplementary Fig. 85 | ^{1}H NMR of compound 6a (400 MHz, at 25 °C, in $CDCl_3$).

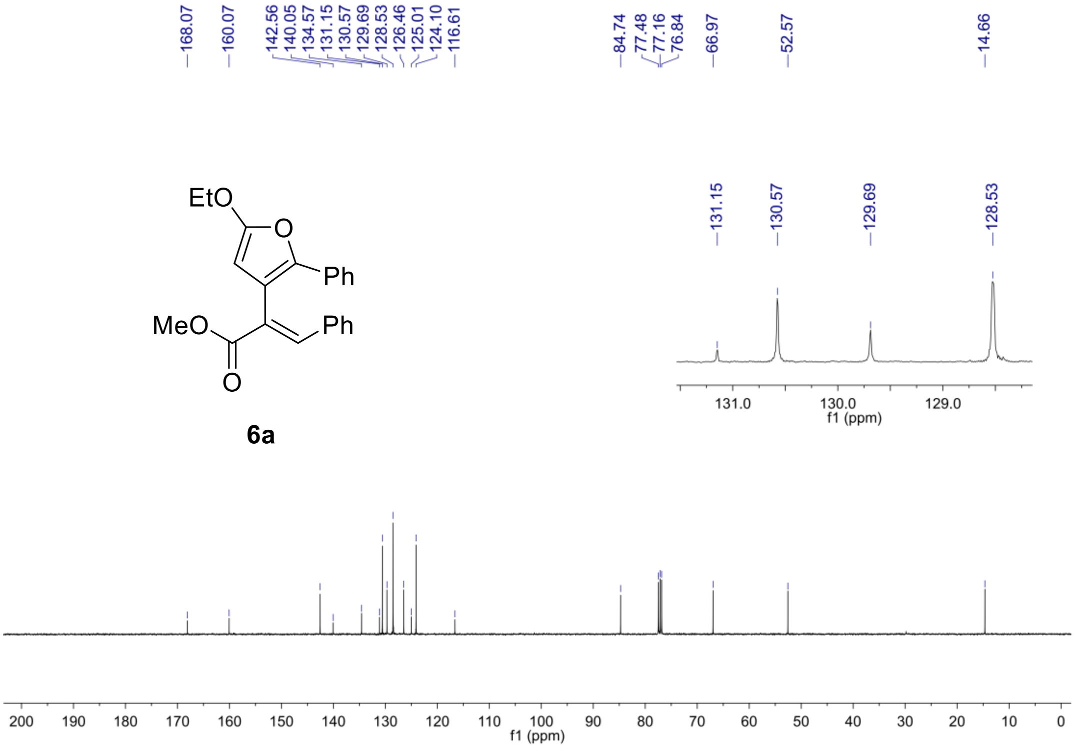

Supplementary Fig. 86 | ^{13}C NMR of compound 6a (100 MHz, at 25 °C, in $CDCl_3$).

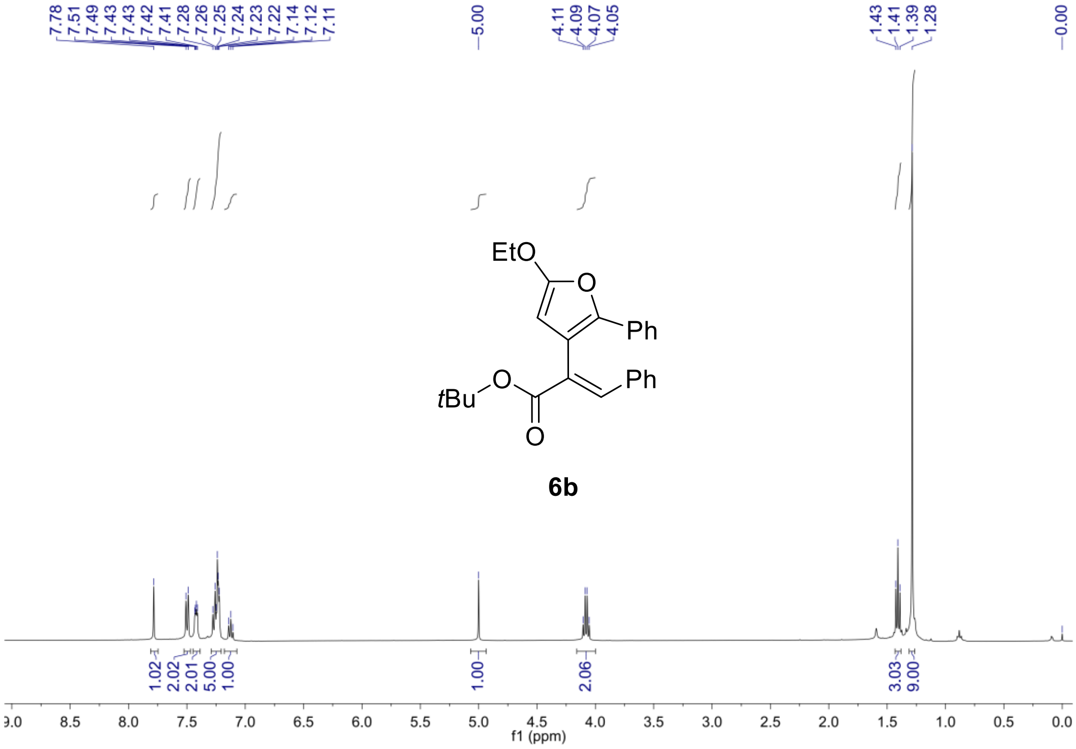

Supplementary Fig. 87 | ^{1}H NMR of compound 6b (400 MHz, at 25 °C, in $CDCl_3$).

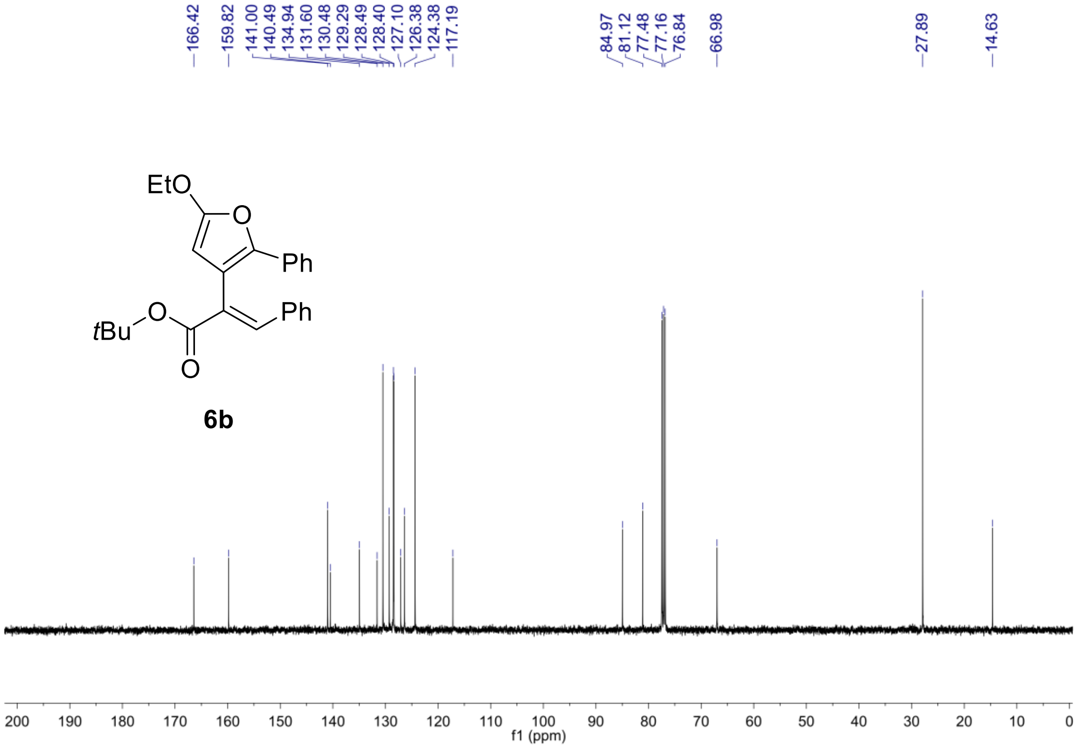

Supplementary Fig. 88 | ^{13}C NMR of compound 6b (100 MHz, at 25 °C, in $CDCl_3$).

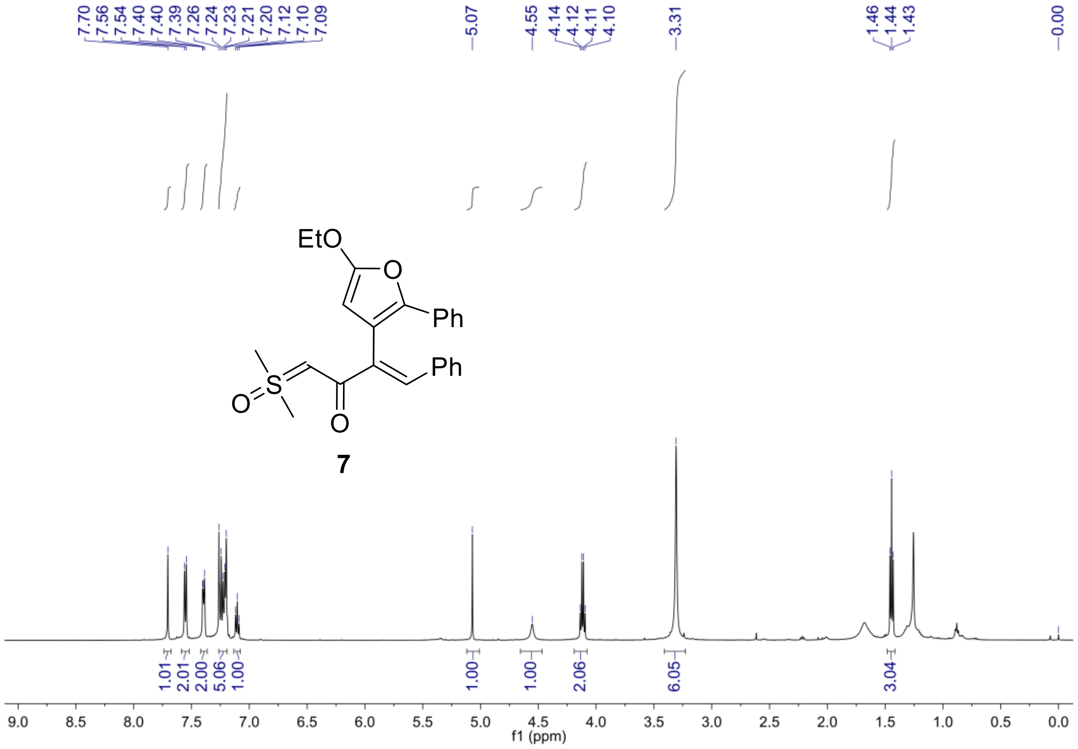

Supplementary Fig. 89 | ^{1}H NMR of compound 7 (500 MHz, at 25 ^{o}C, in $CDCl_3$).

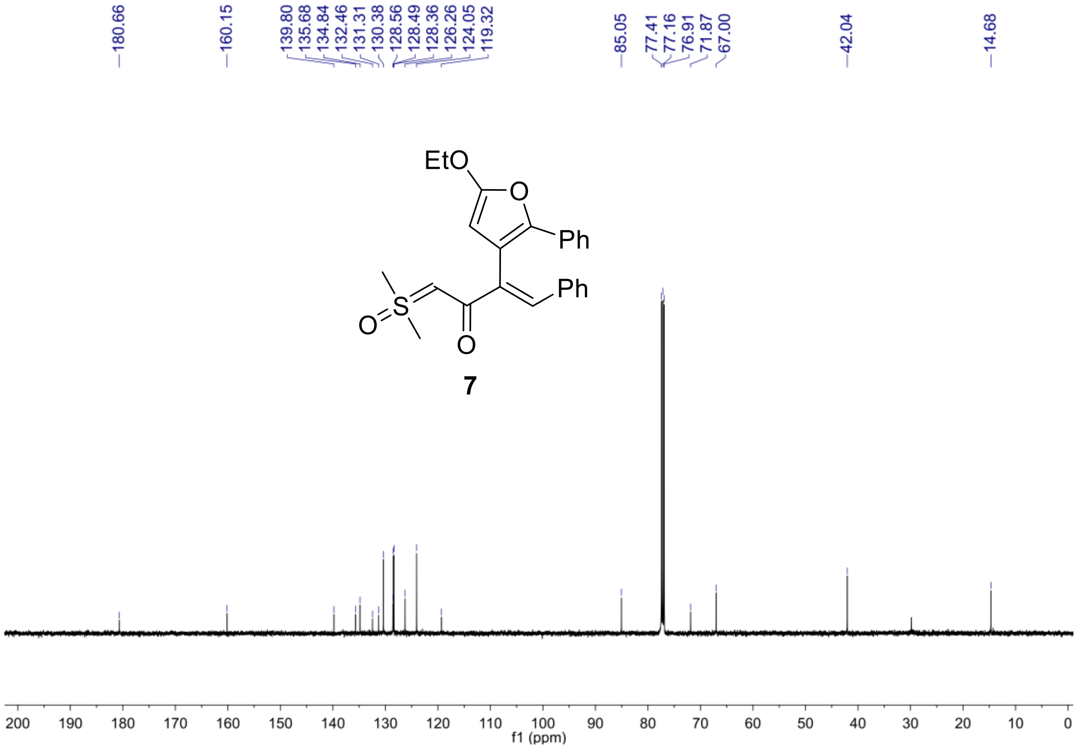

Supplementary Fig. 90 | ^{13}C NMR of compound 7 (125 MHz, at 25 ^{o}C, in $CDCl_3$).

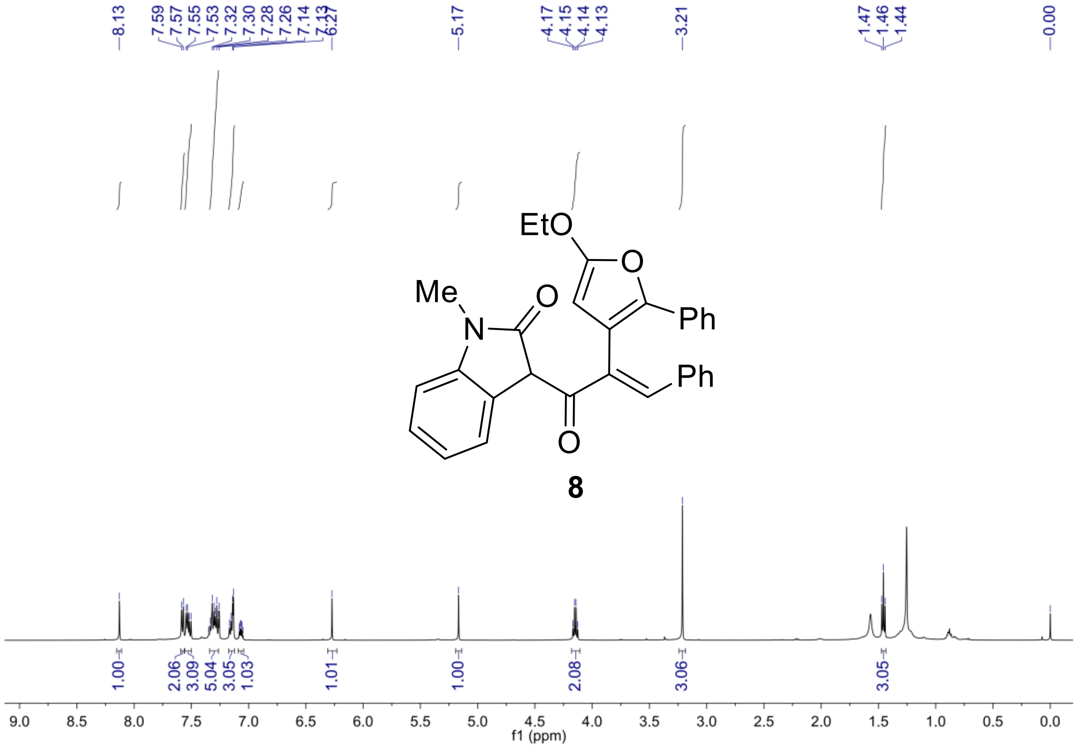

Supplementary Fig. 91 | ^{1}H NMR of compound 8 (500 MHz, at 25 ºC, in $CDCl_3$).

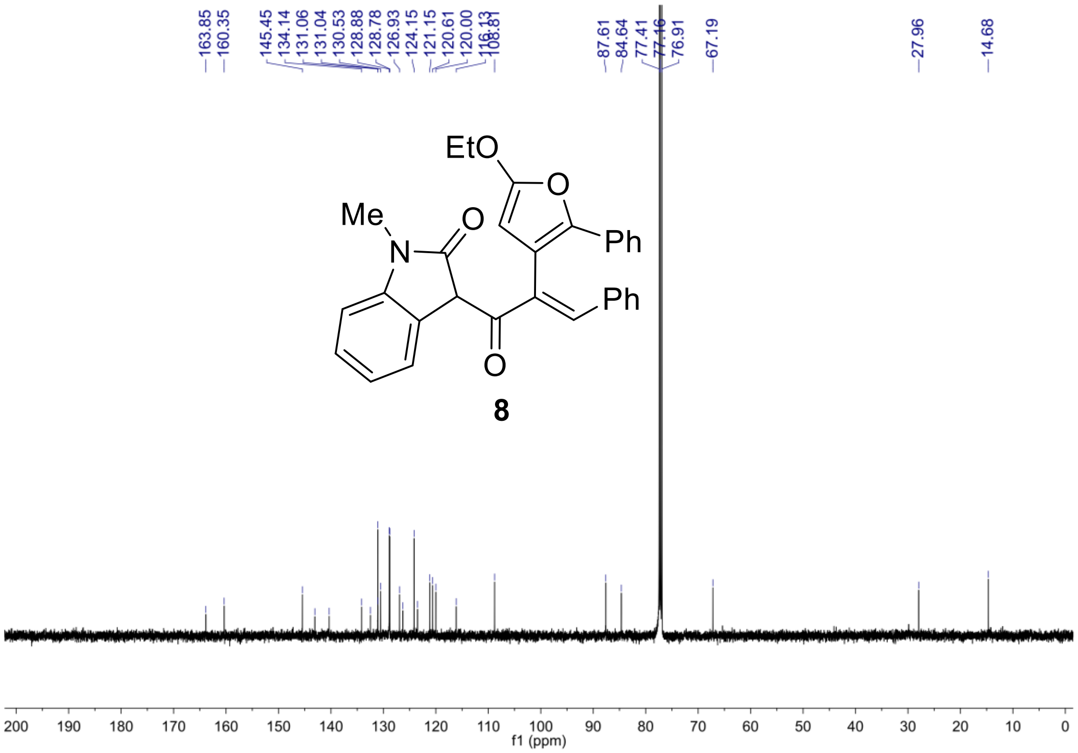

Supplementary Fig. 92 | ^{13}C NMR of compound 8 (125 MHz, at 25 ºC, in $CDCl_3$).

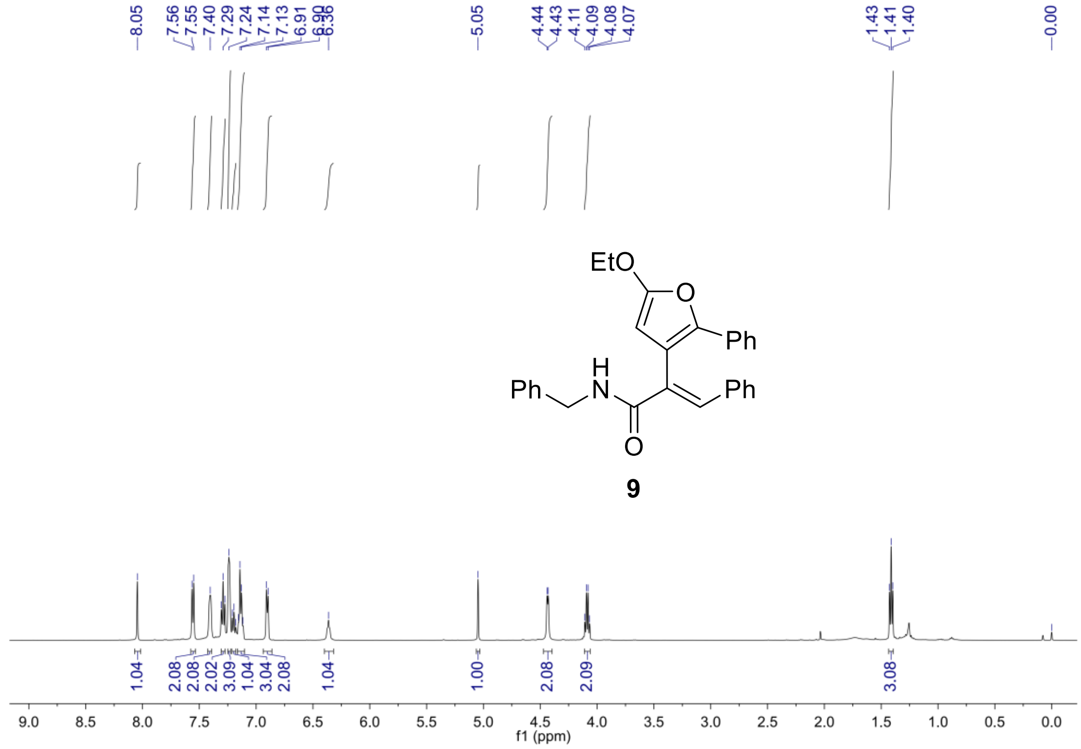

Supplementary Fig. 93 | ^{1}H NMR of compound 9 (500 MHz, at 25 ^{o}C, in $CDCl_3$).

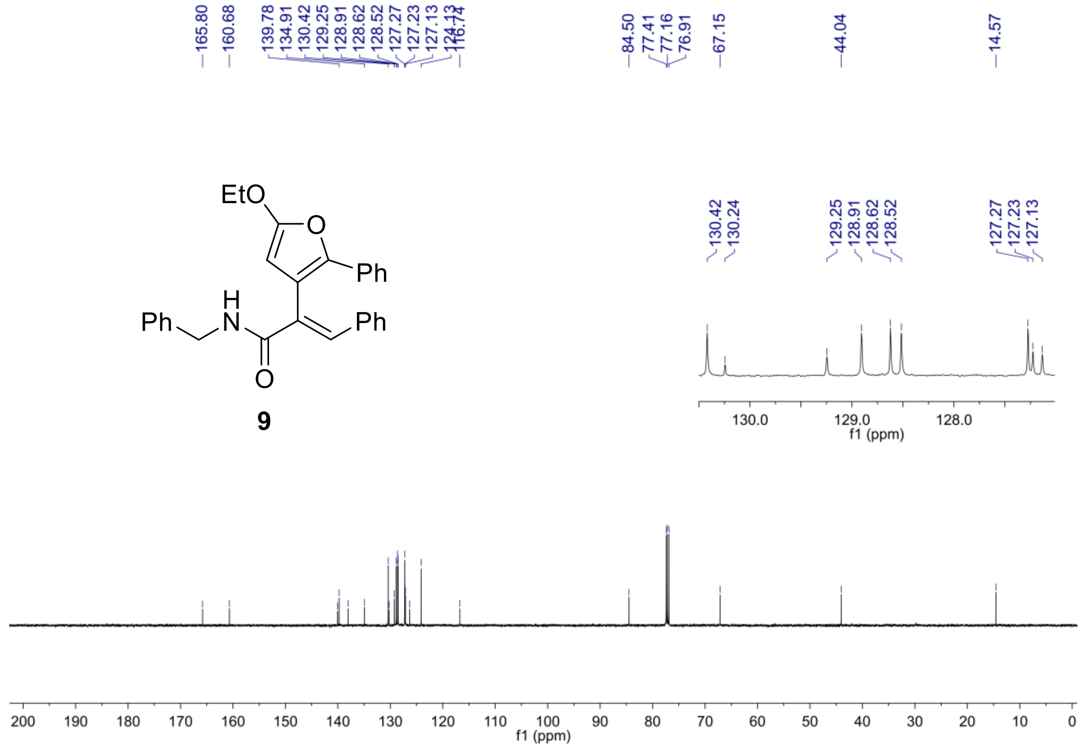

Supplementary Fig. 94 | ^{13}C NMR of compound 9 (125 MHz, at 25 ^{o}C, in $CDCl_3$).

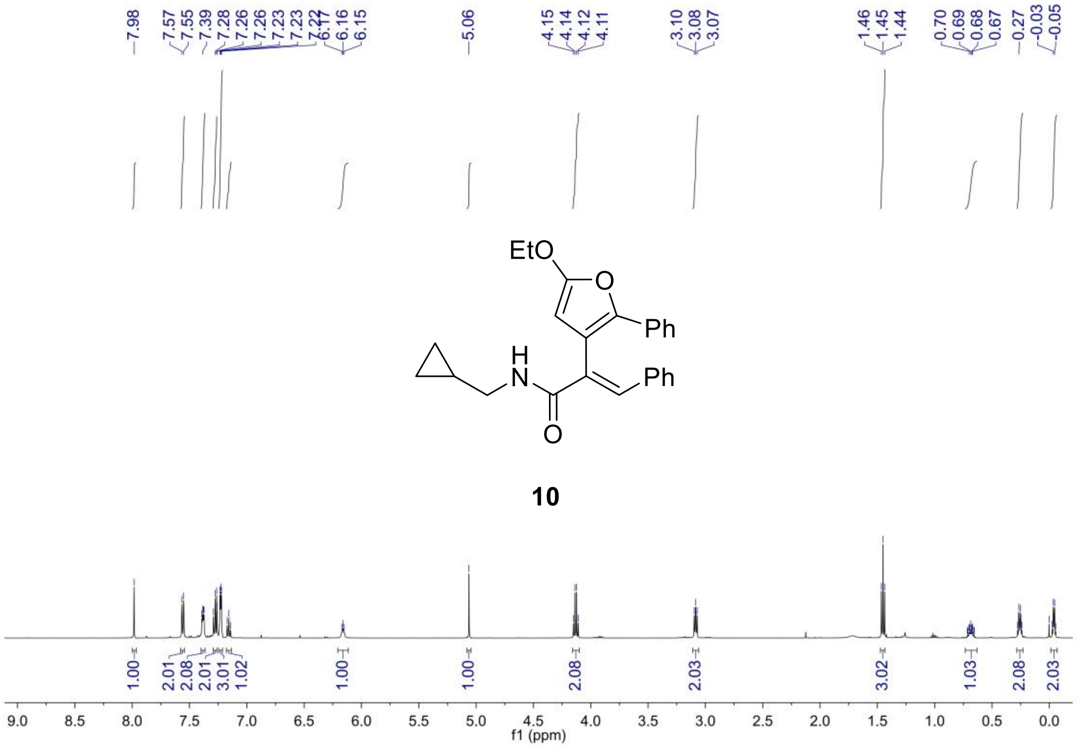

Supplementary Fig. 95 | ^{1}H NMR of compound 10 (500 MHz, at 25 °C, in $CDCl_3$).

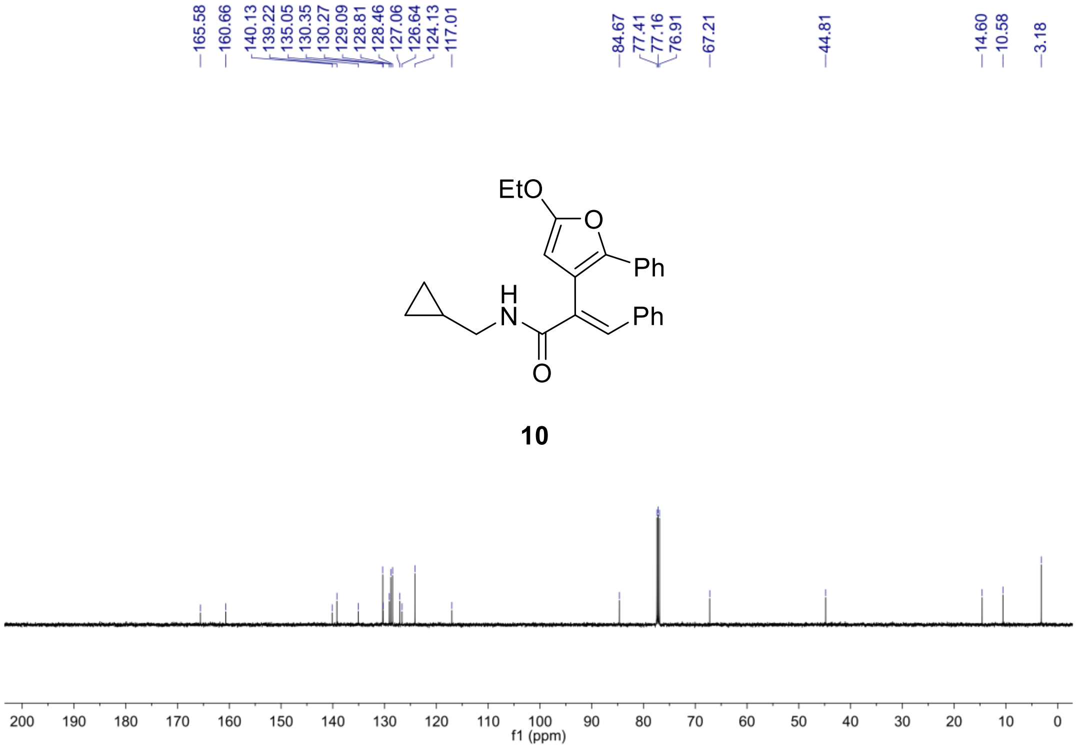

Supplementary Fig. 96 | ^{13}C NMR of compound 10 (125 MHz, at 25 °C, in $CDCl_3$).

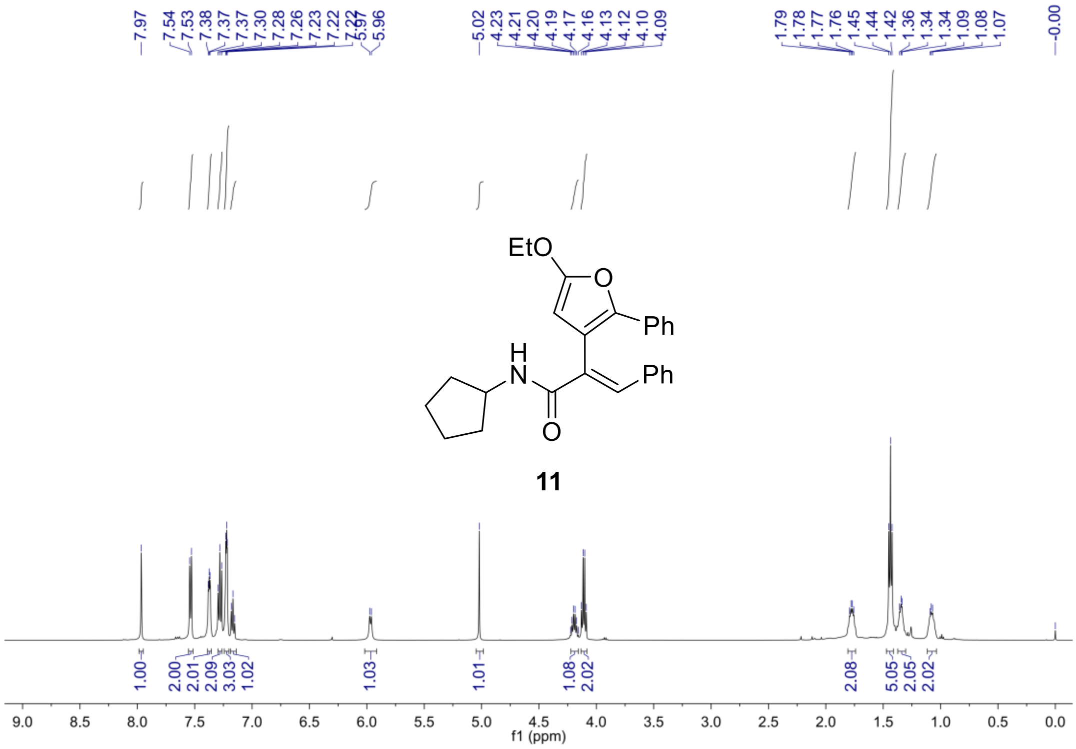

Supplementary Fig. 97 | ^{1}H NMR of compound 11 (500 MHz, at 25 °C, in $CDCl_3$).

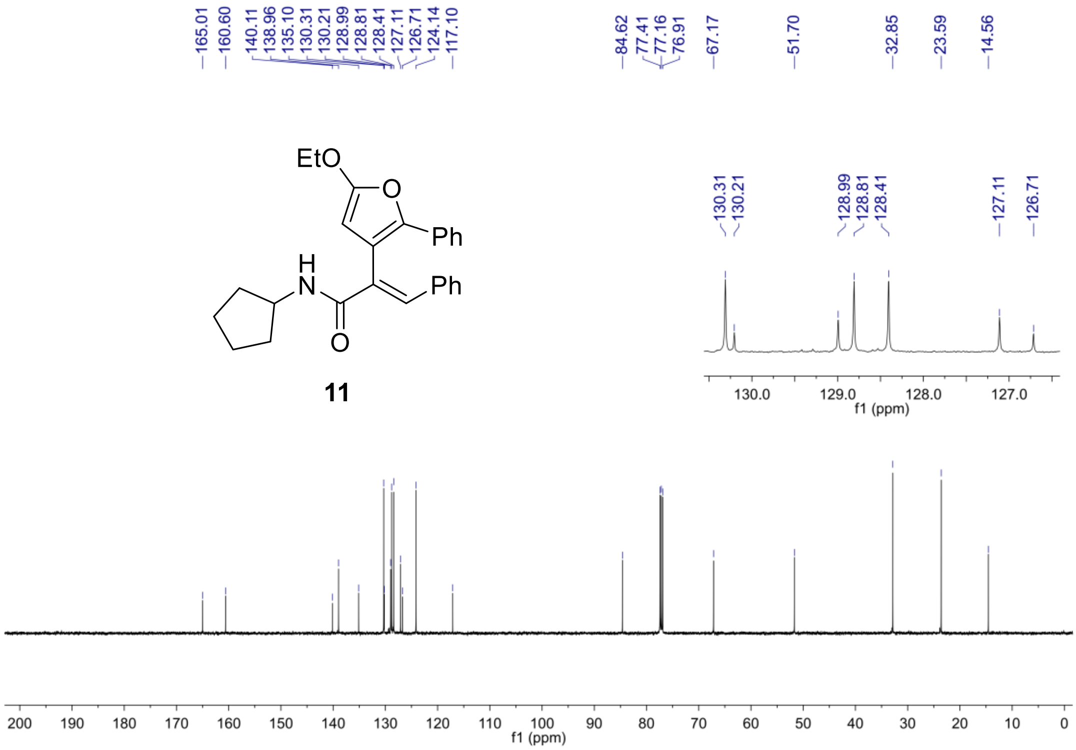

Supplementary Fig. 98 | ^{13}C NMR of compound 11 (125 MHz, at 25 °C, in $CDCl_3$).

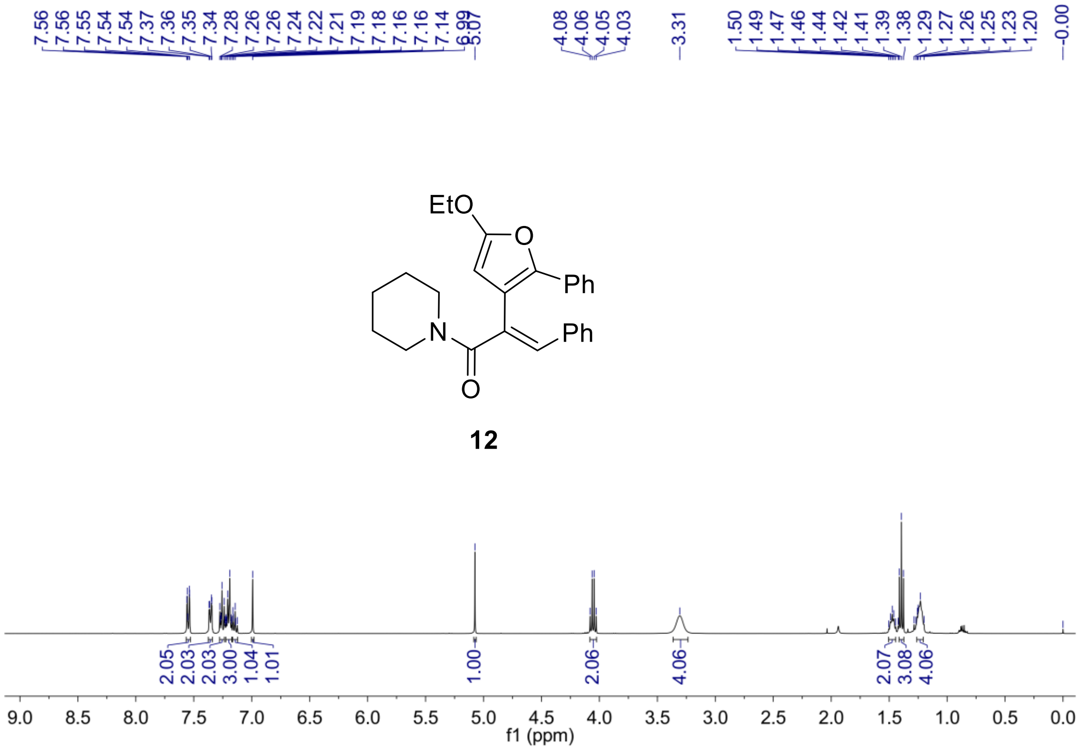

Supplementary Fig. 99 | ^{1}H NMR of compound 12 (400 MHz, at 25 °C, in $CDCl_3$).

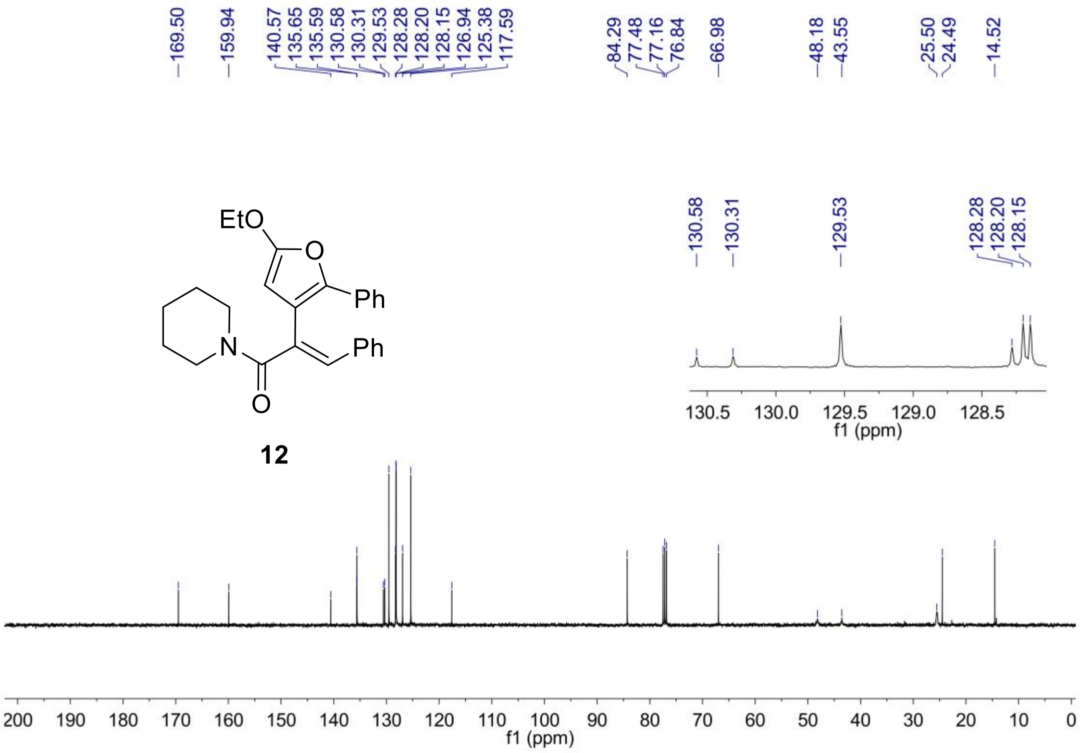

Supplementary Fig. 100 | ^{13}C NMR of compound 12 (100 MHz, at 25 °C, in $CDCl_3$).

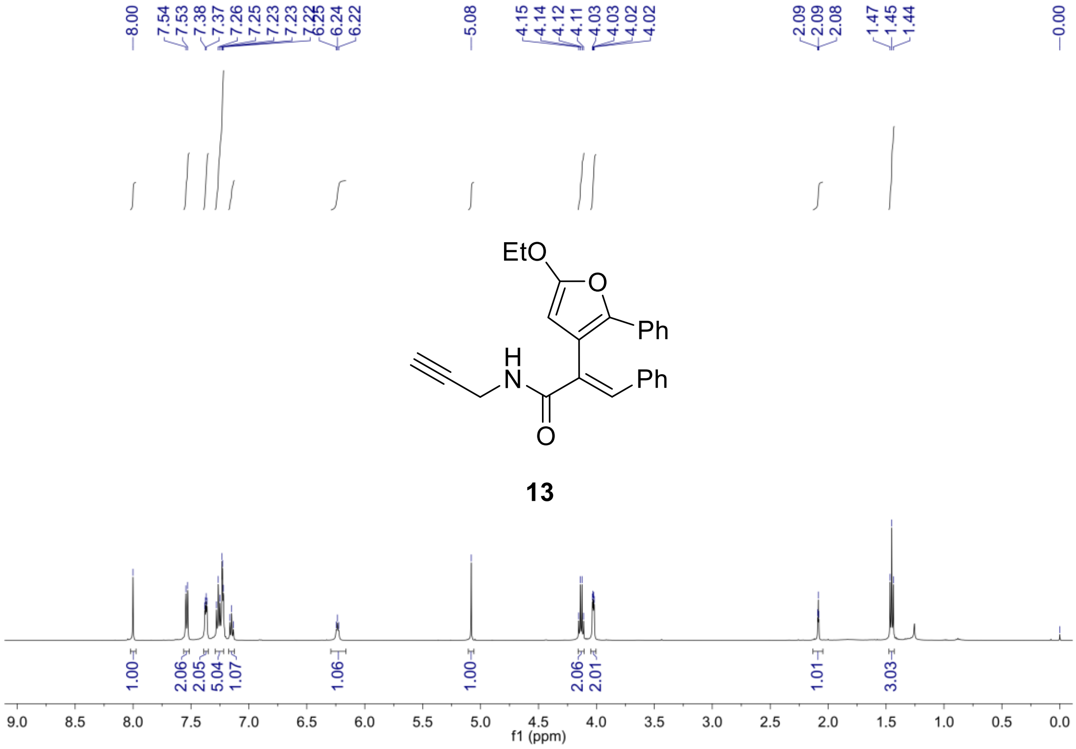

Supplementary Fig. 101 | ¹H NMR of compound 13 (500 MHz, at 25 °C, in $CDCl_3$).

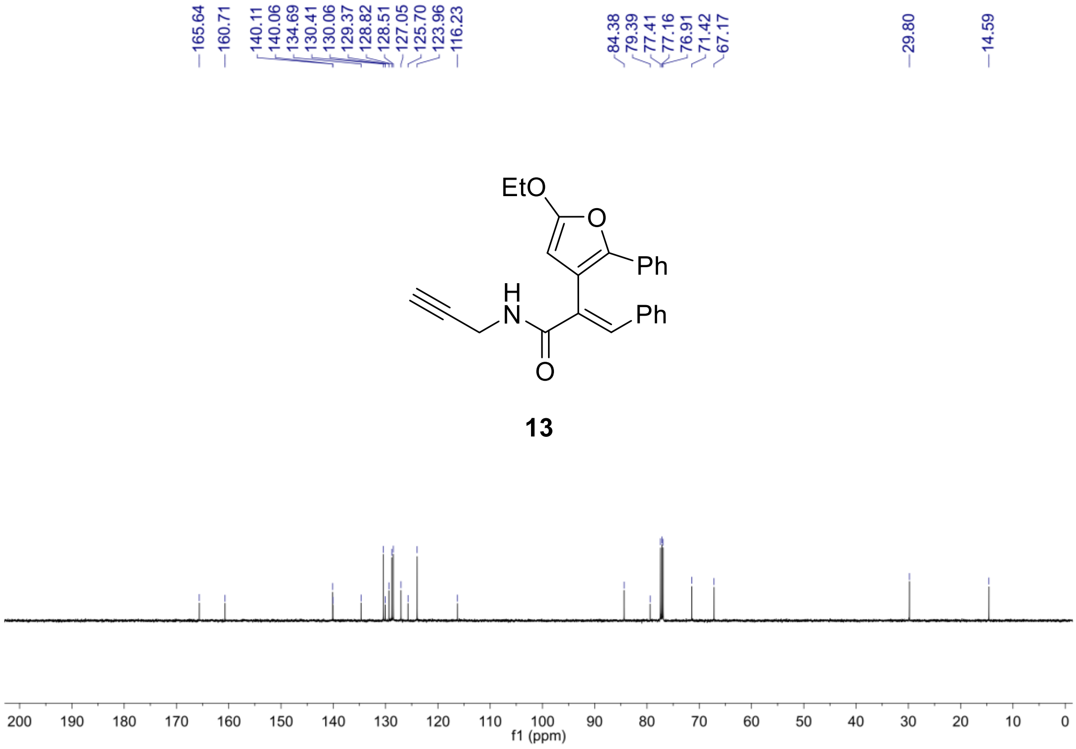

Supplementary Fig. 102 | ¹³C NMR of compound 13 (125 MHz, at 25 °C, in $CDCl_3$).

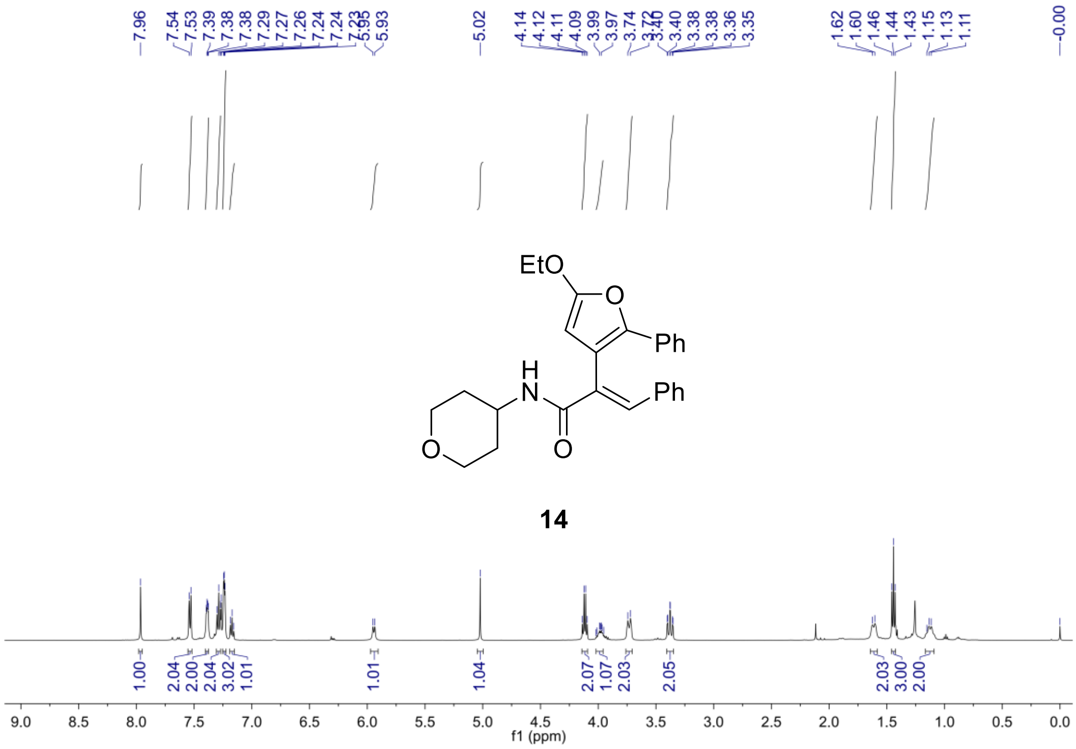

Supplementary Fig. 103 | ^{1}H NMR of compound 14 (500 MHz, at 25 °C, in $CDCl_3$).

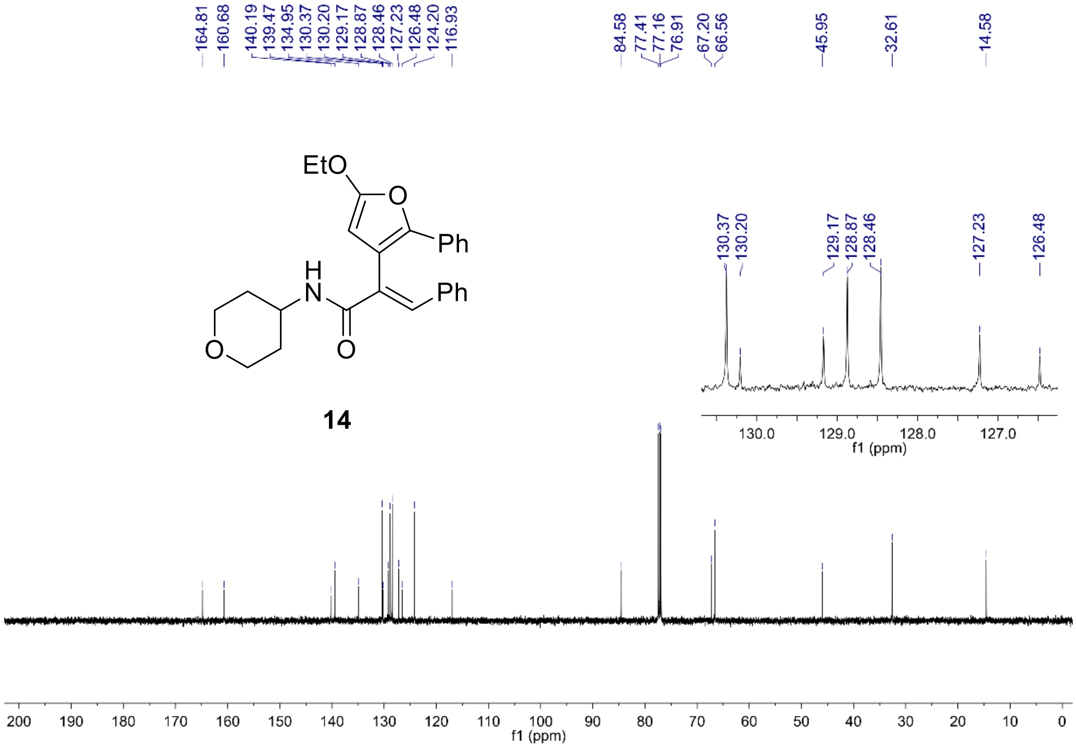

Supplementary Fig. 104 | ^{13}C NMR of compound 14 (125 MHz, at 25 °C, in $CDCl_3$).

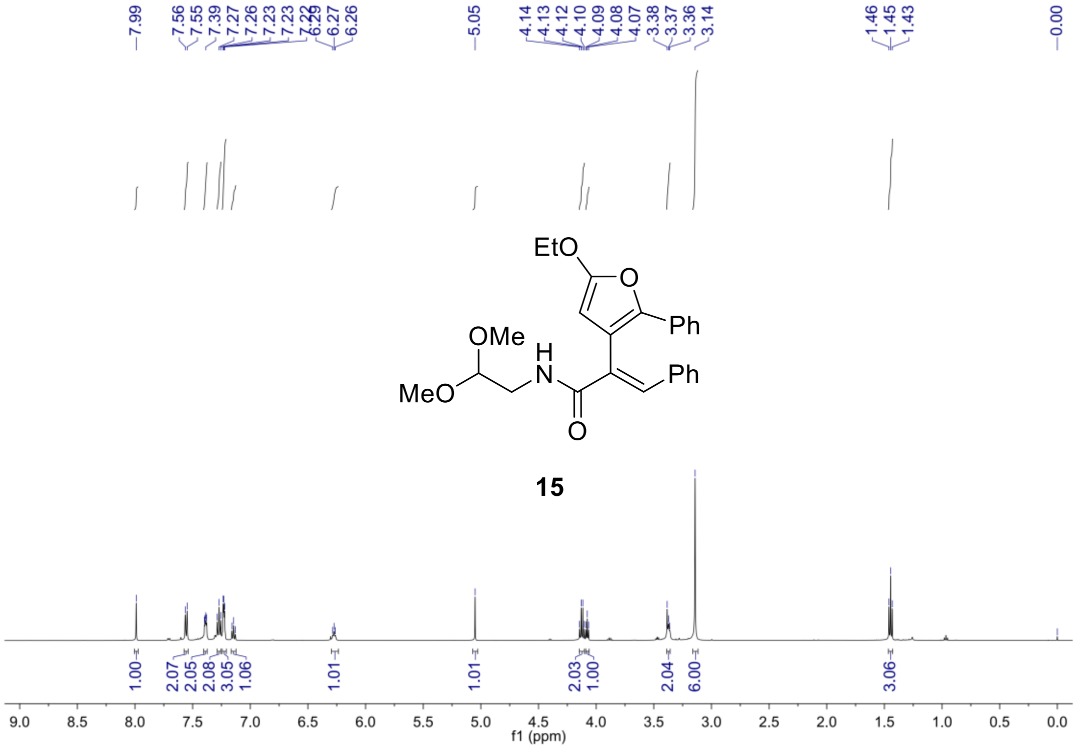

Supplementary Fig. 105 | ^{1}H NMR of compound 15 (500 MHz, at 25 °C, in $CDCl_3$).

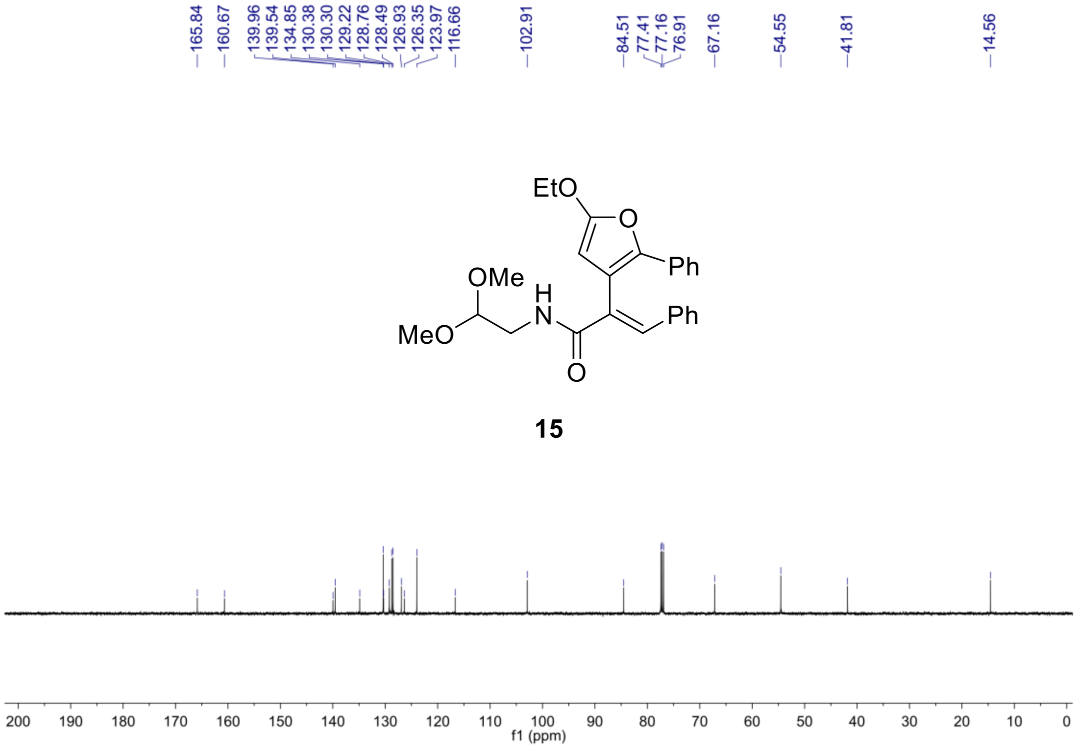

Supplementary Fig. 106 | ^{13}C NMR of compound 15 (125 MHz, at 25 °C, in $CDCl_3$).

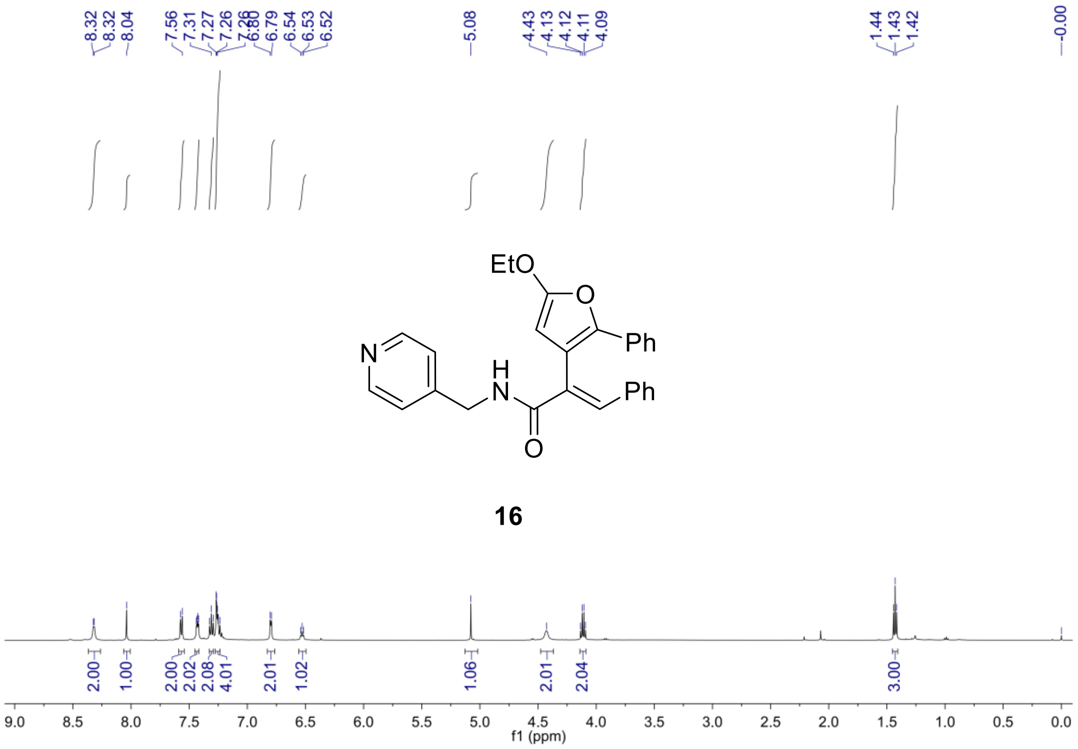

Supplementary Fig. 107 | ^{1}H NMR of compound 16 (500 MHz, at 25 °C, in $CDCl_3$).

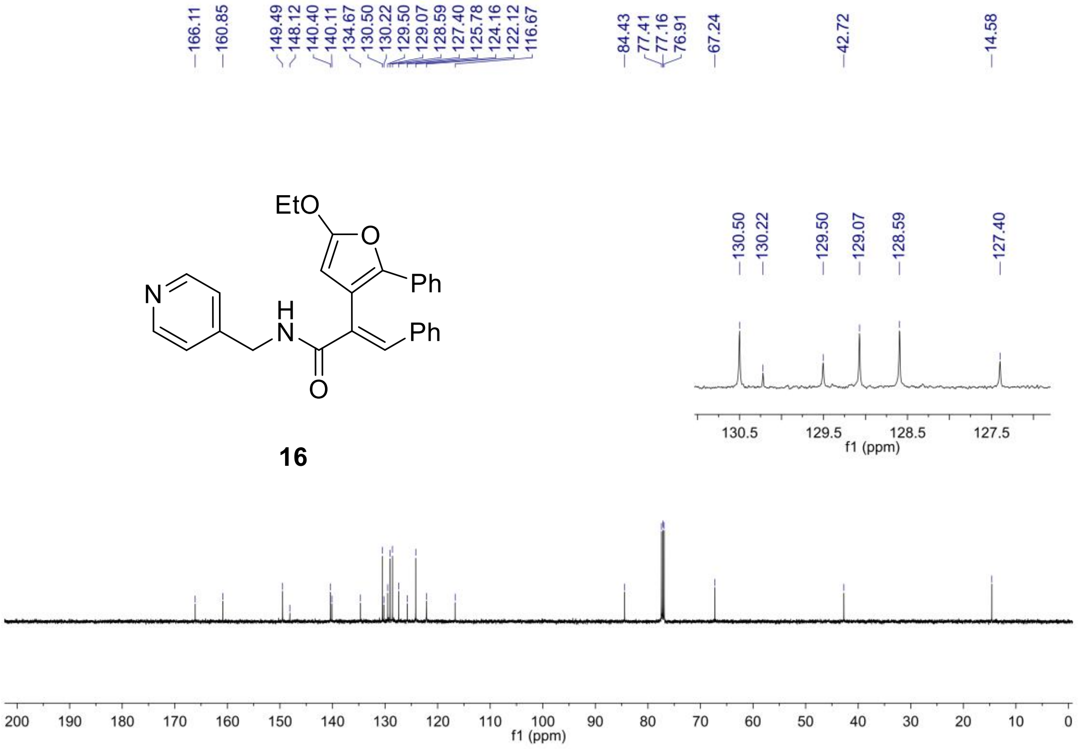

Supplementary Fig. 108 | ^{13}C NMR of compound 16 (125 MHz, at 25 °C, in $CDCl_3$).

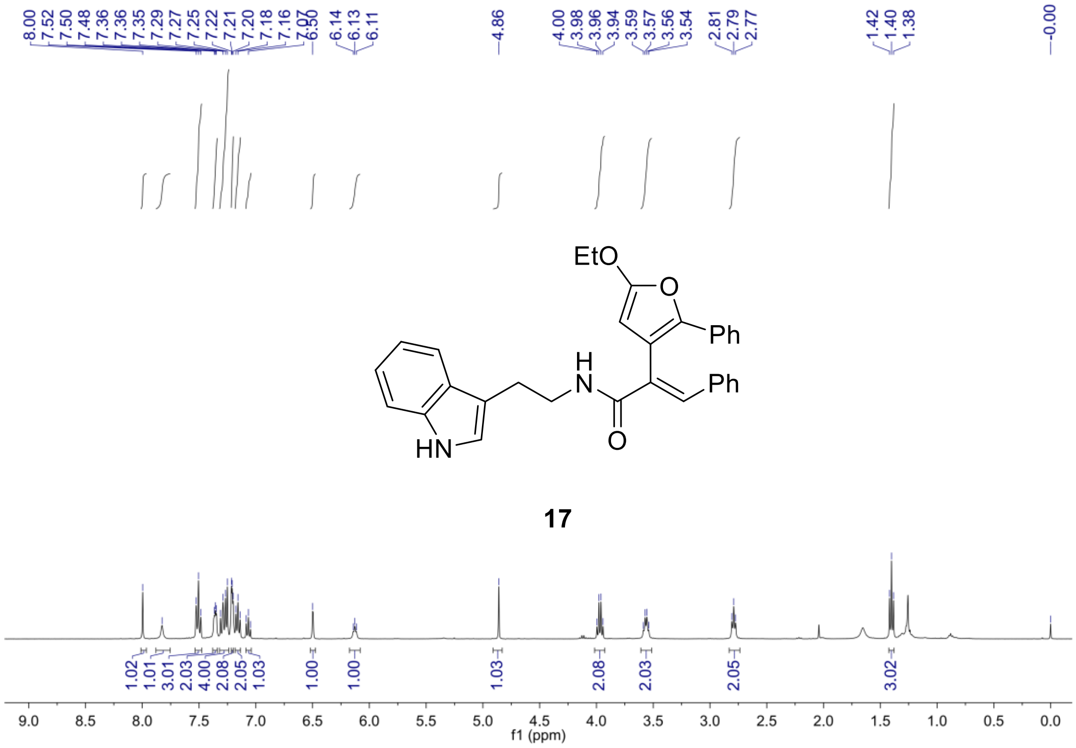

Supplementary Fig. 109 | ^{1}H NMR of compound 17 (400 MHz, at 25 ^{o}C, in $CDCl_3$).

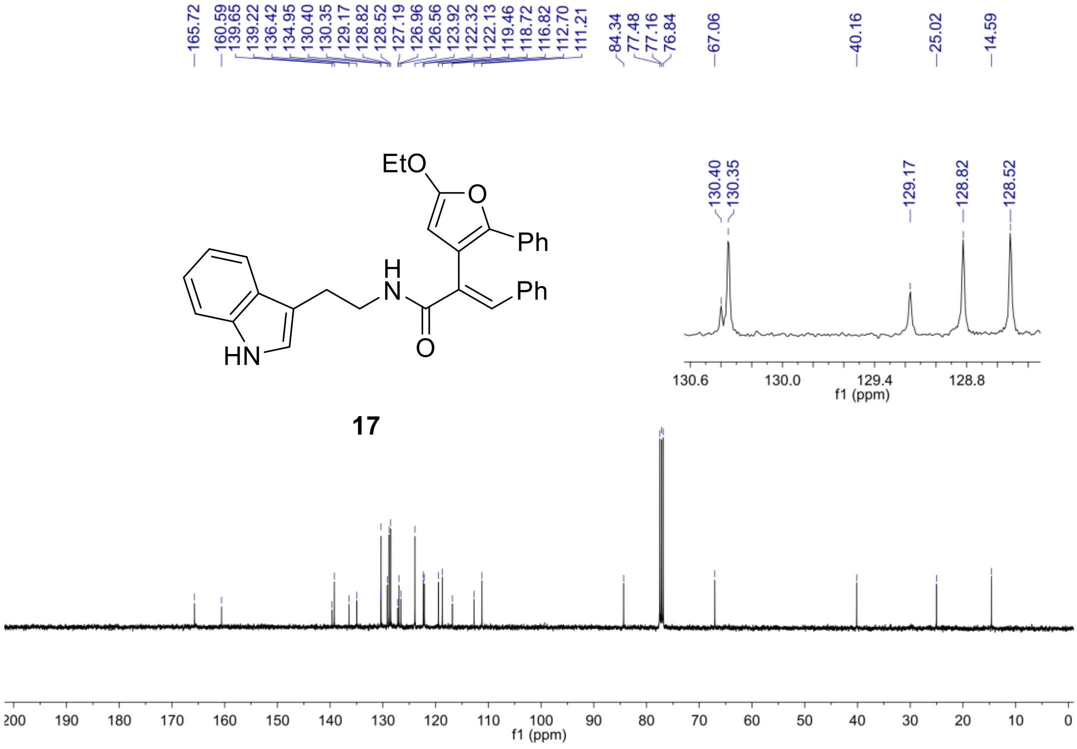

Supplementary Fig. 110 | ^{13}C NMR of compound 17 (100 MHz, at 25 ^{o}C, in $CDCl_3$).

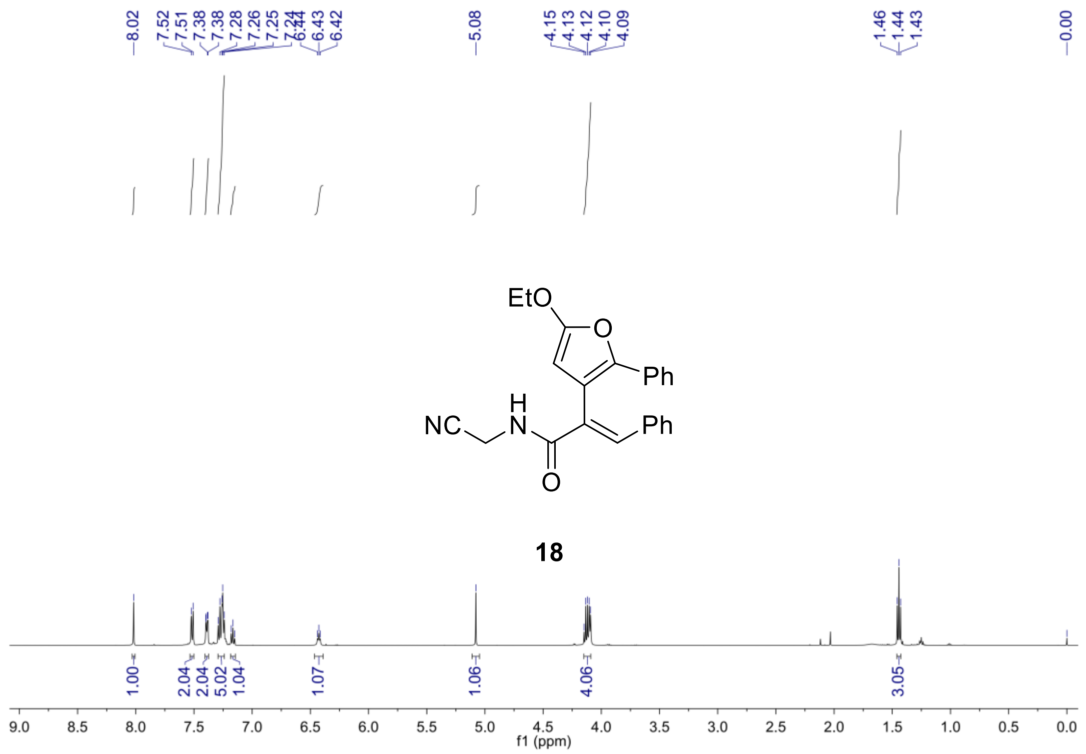

Supplementary Fig. 111 | ^{1}H NMR of compound 18 (500 MHz, at 25 °C, in $CDCl_3$).

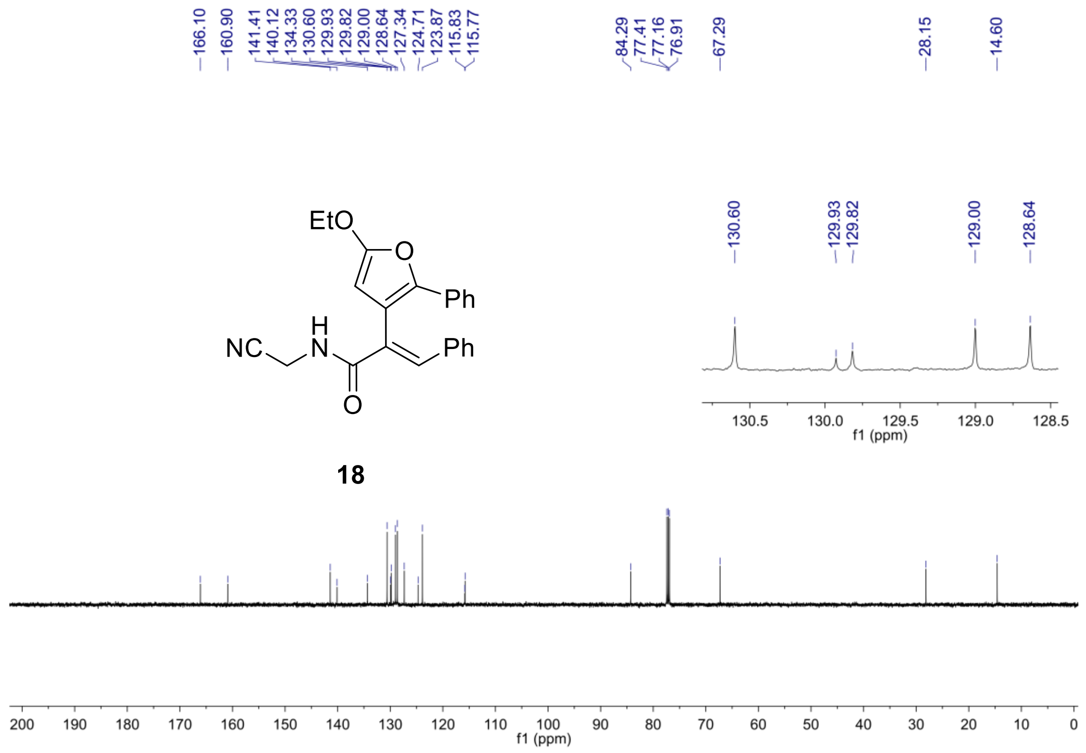

Supplementary Fig. 112 | ^{13}C NMR of compound 18 (125 MHz, at 25 °C, in $CDCl_3$).

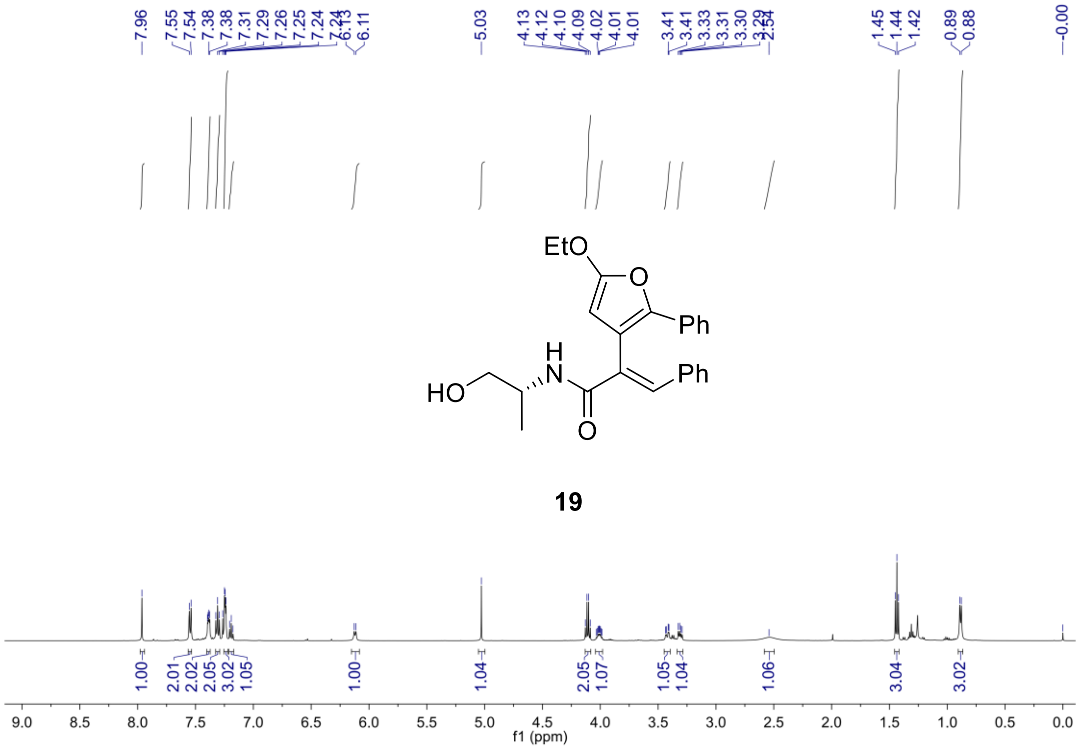

Supplementary Fig. 113 | ^{1}H NMR of compound 19 (500 MHz, at 25 °C, in $CDCl_3$).

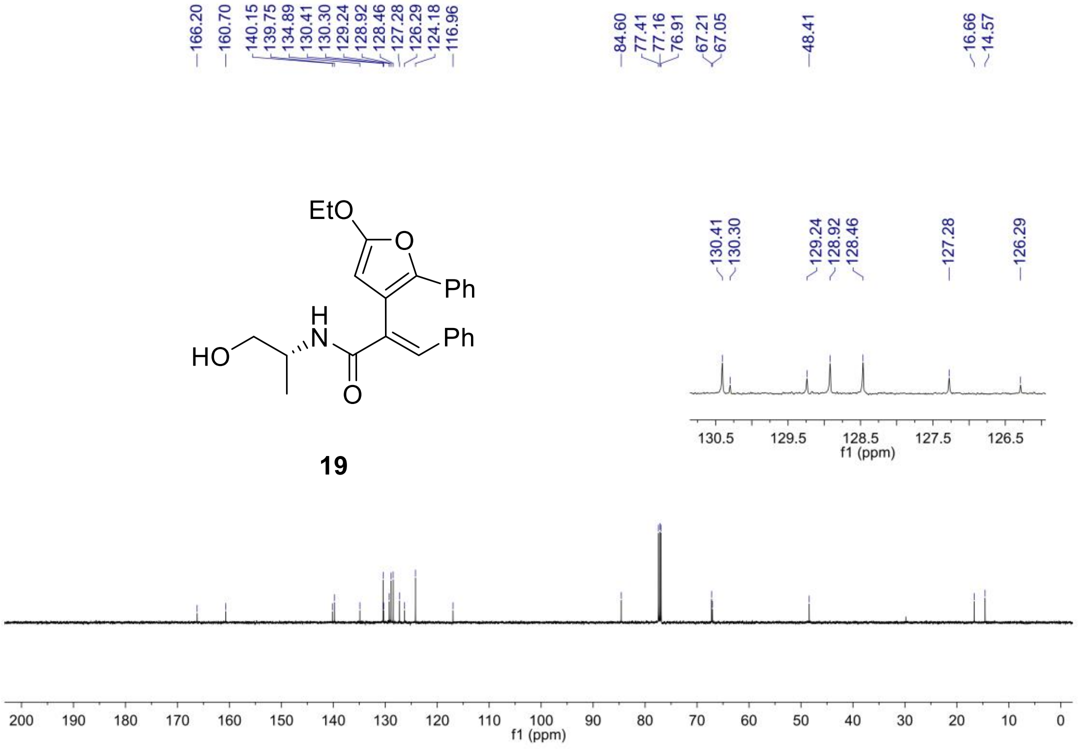

Supplementary Fig. 114 | ^{13}C NMR of compound 19 (125 MHz, at 25 °C, in $CDCl_3$).

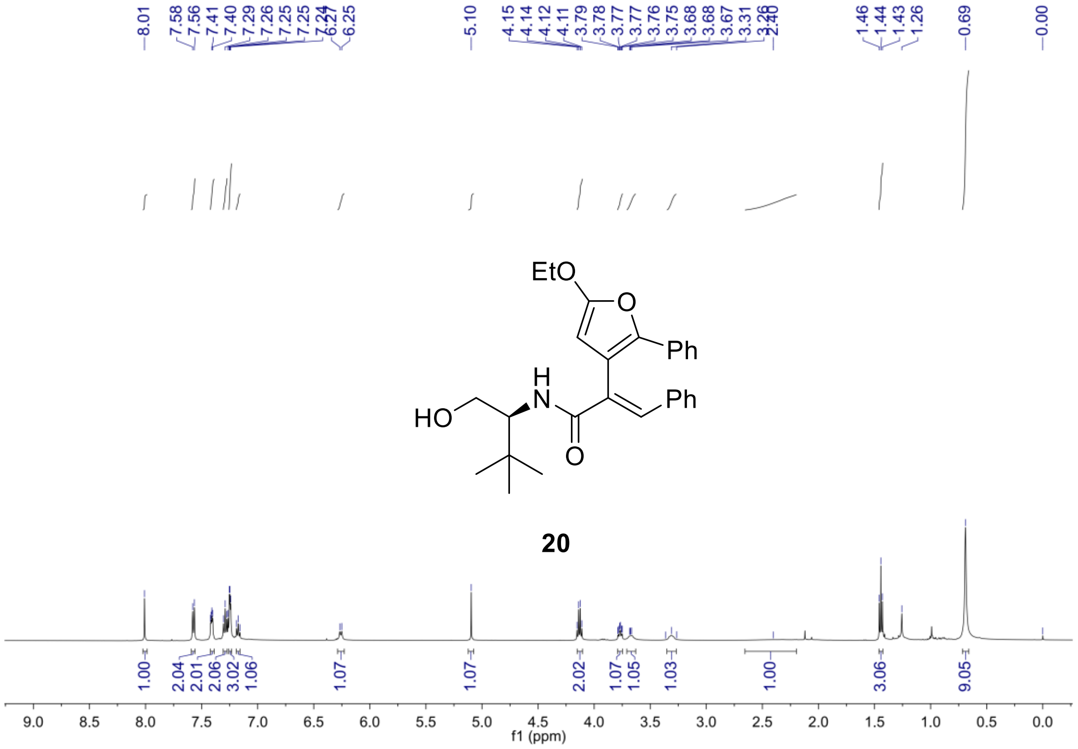

Supplementary Fig. 115 | 1H NMR of compound 20 (500 MHz, at 25 °C, in CDCl3).

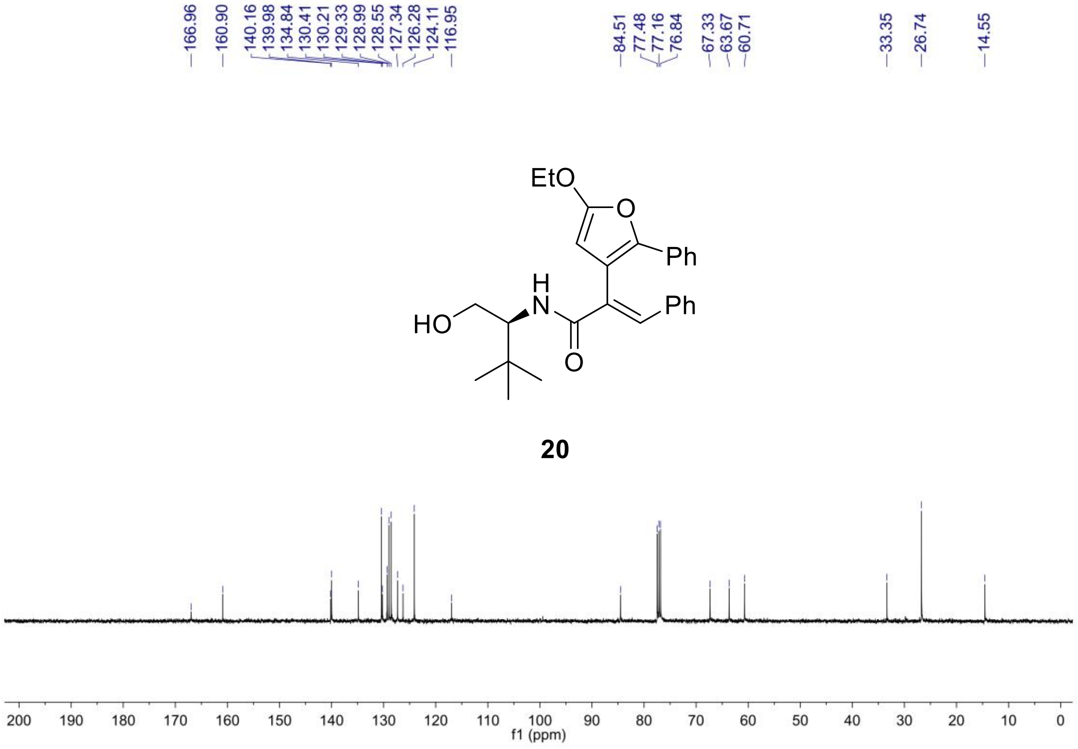

Supplementary Fig. 116 | 13C NMR of compound 20 (100 MHz, at 25 °C, in CDCl3).

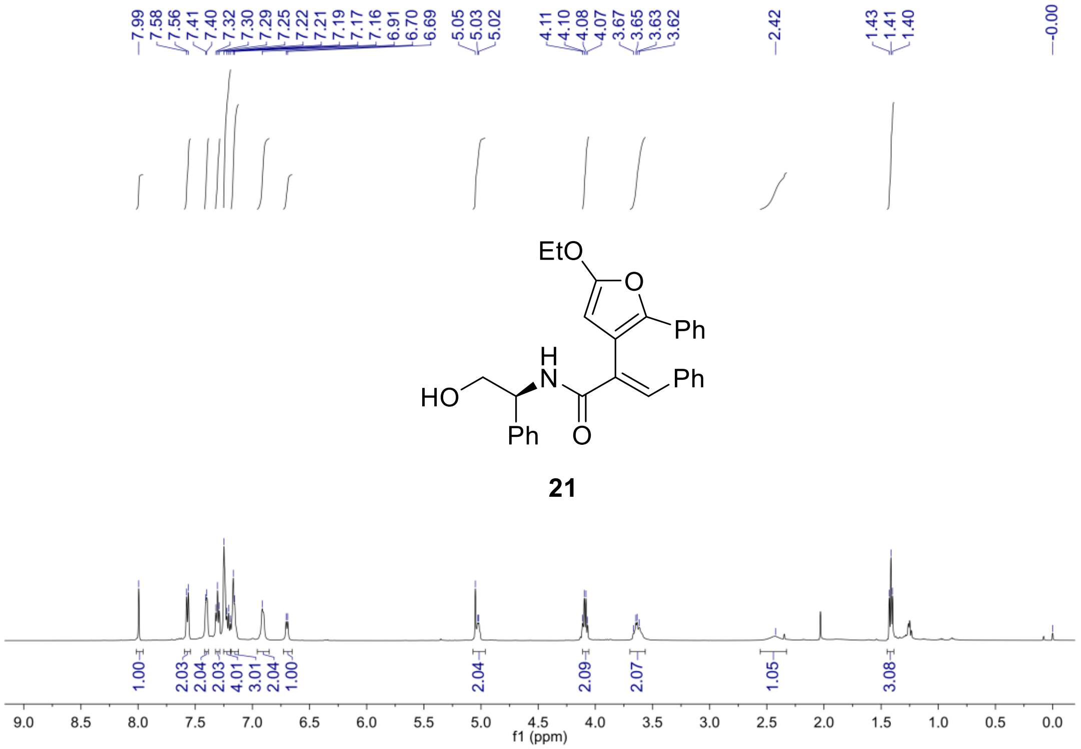

Supplementary Fig. 117 | ^{1}H NMR of compound 21 (500 MHz, at 25 ^{o}C, in $CDCl_3$).

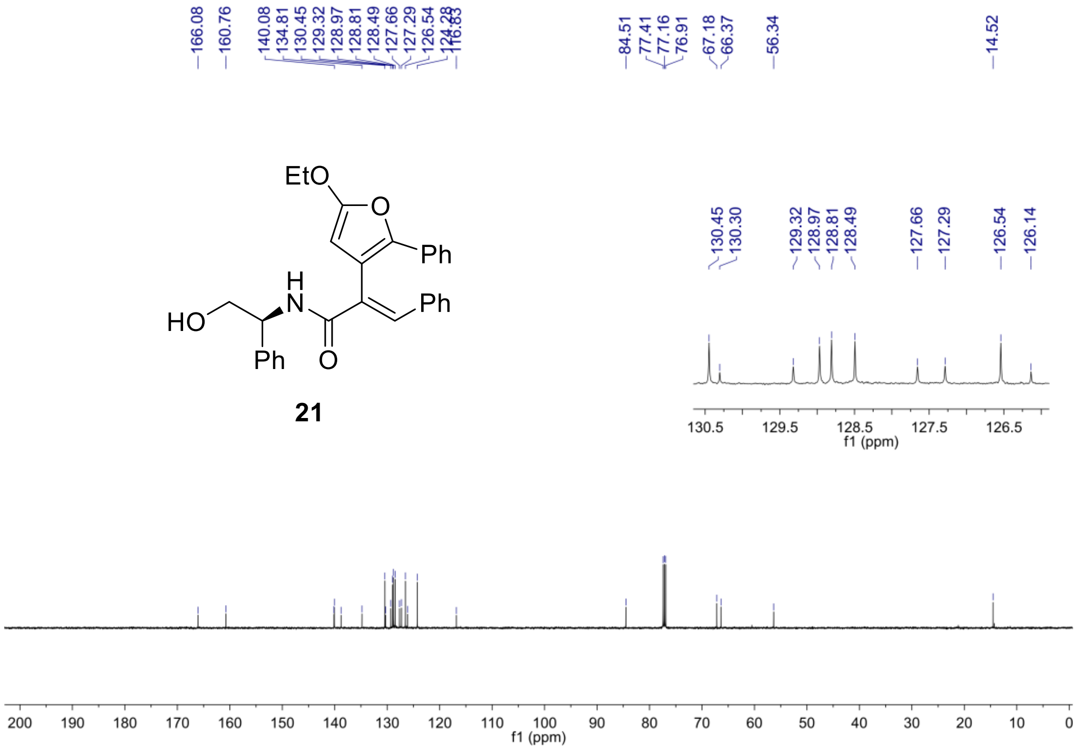

Supplementary Fig. 118 | ^{13}C NMR of compound 21 (125 MHz, at 25 ^{o}C, in $CDCl_3$).

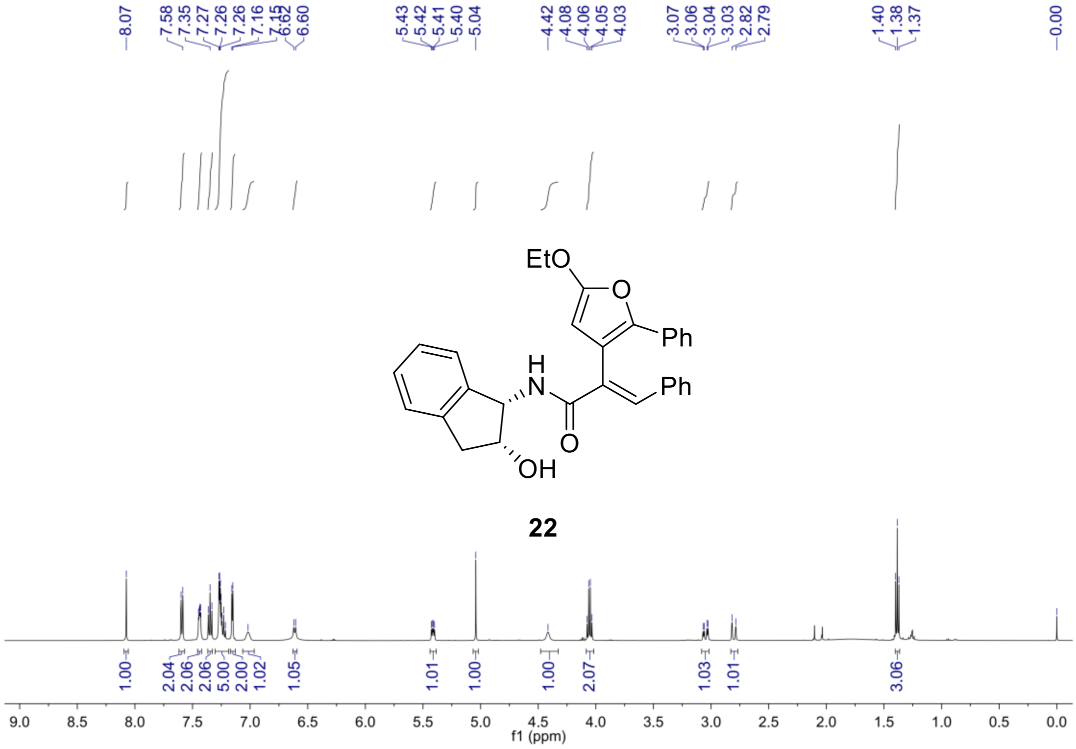

Supplementary Fig. 119 | ^{1}H NMR of compound 22 (500 MHz, at 25 °C, in $CDCl_3$).

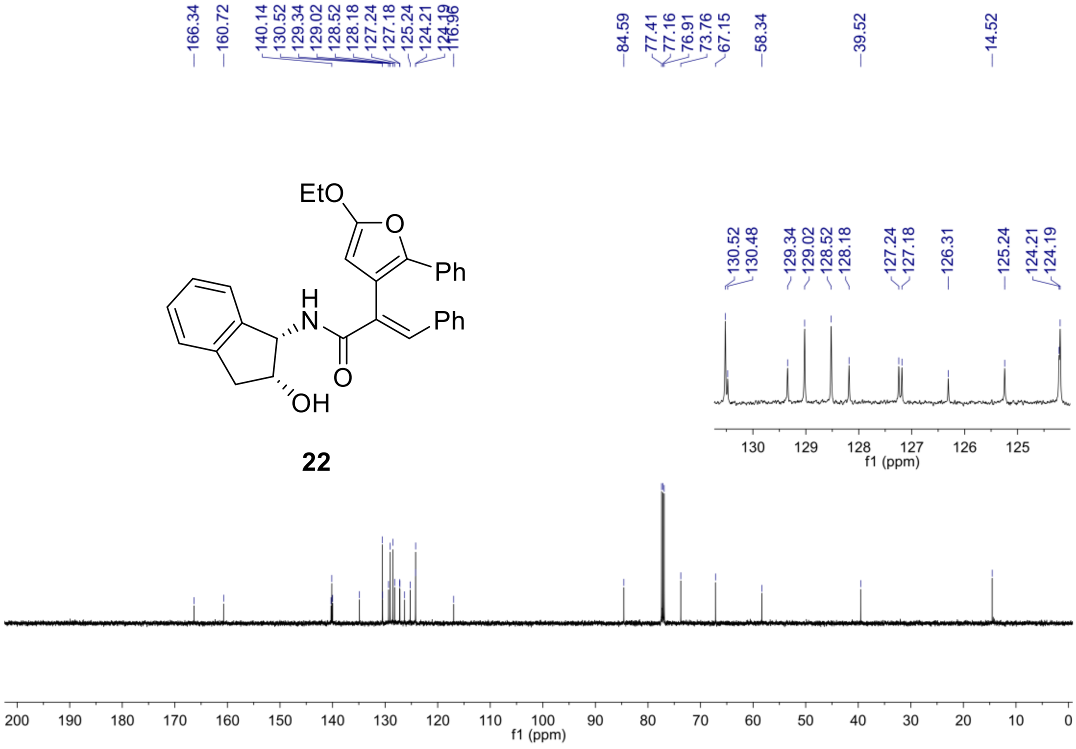

Supplementary Fig. 120 | ^{13}C NMR of compound 22 (125 MHz, at 25 °C, in $CDCl_3$).

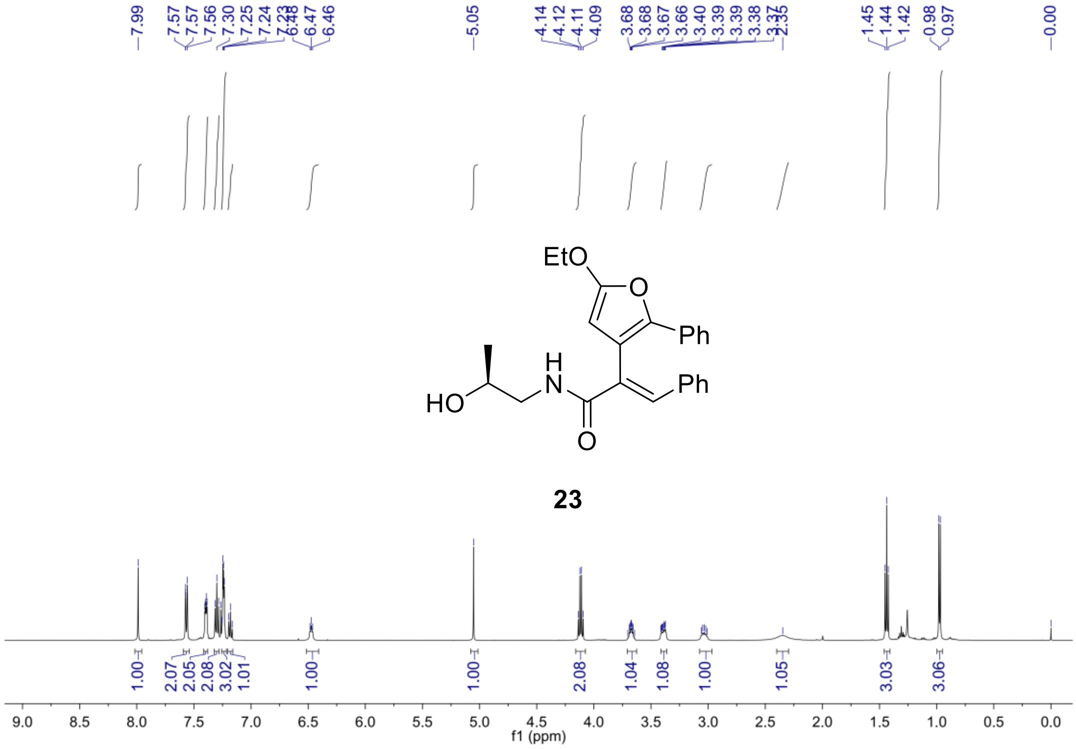

Supplementary Fig. 121 | ^{1}H NMR of compound 23 (500 MHz, at 25 °C, in $CDCl_3$).

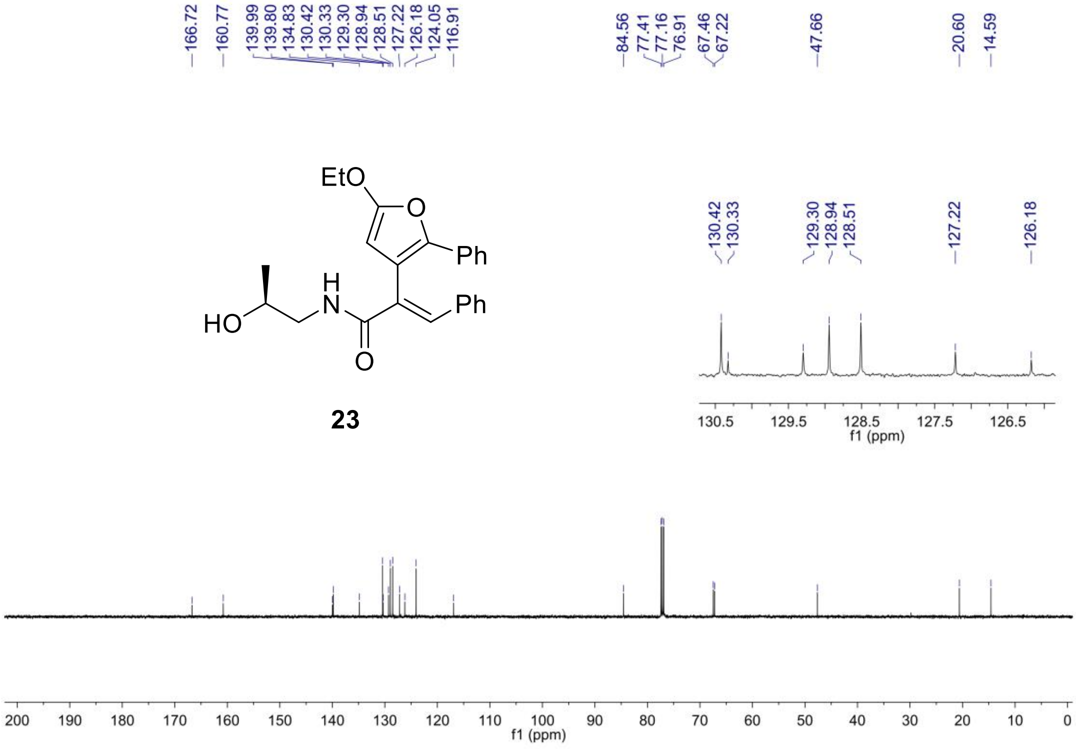

Supplementary Fig. 122 | ^{13}C NMR of compound 23 (125 MHz, at 25 °C, in $CDCl_3$).

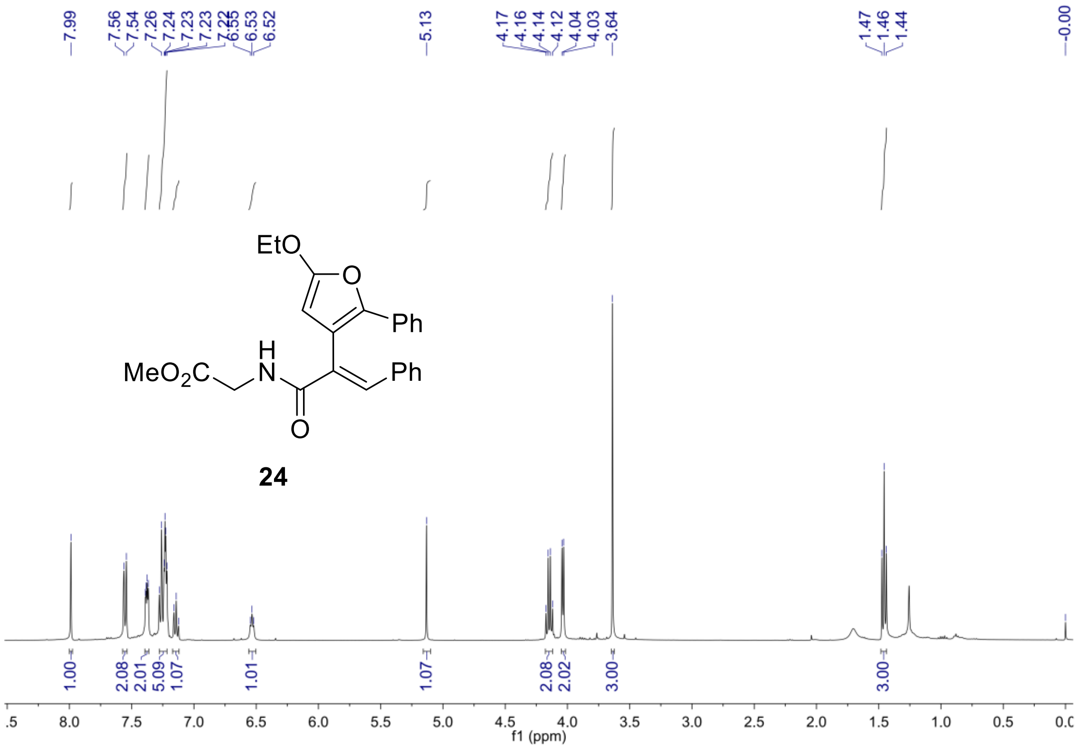

Supplementary Fig. 123 | ^{1}H NMR of compound 24 (400 MHz, at 25 °C, in $CDCl_3$).

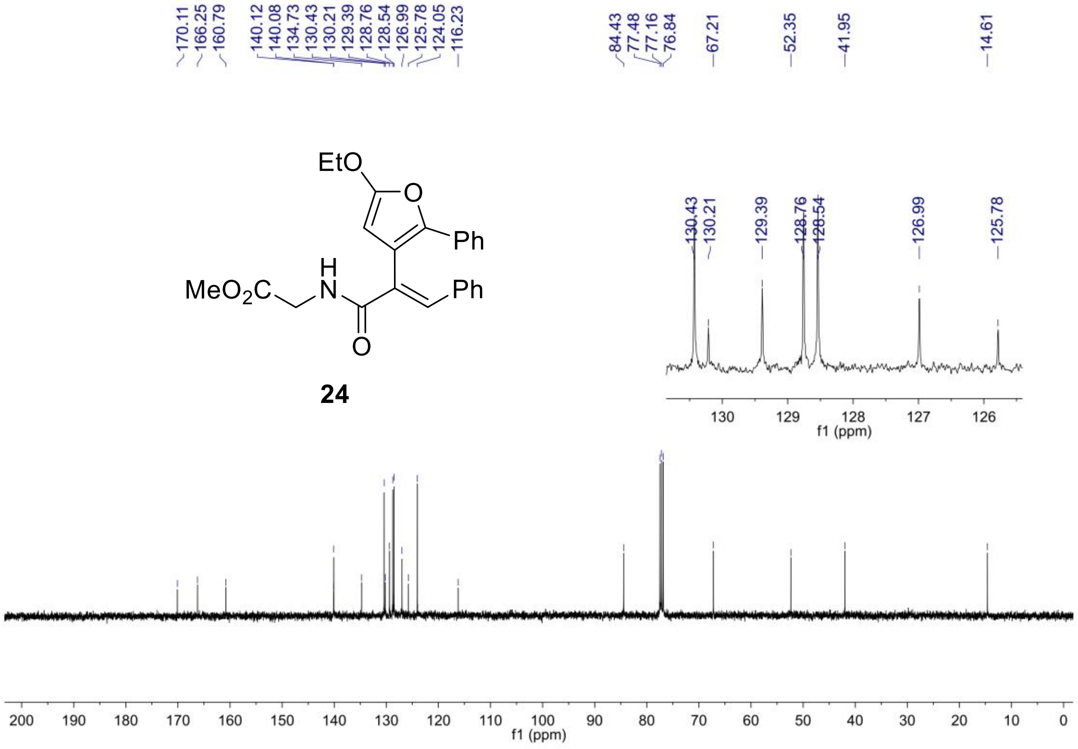

Supplementary Fig. 124 | ^{13}C NMR of compound 24 (100 MHz, at 25 °C, in $CDCl_3$).

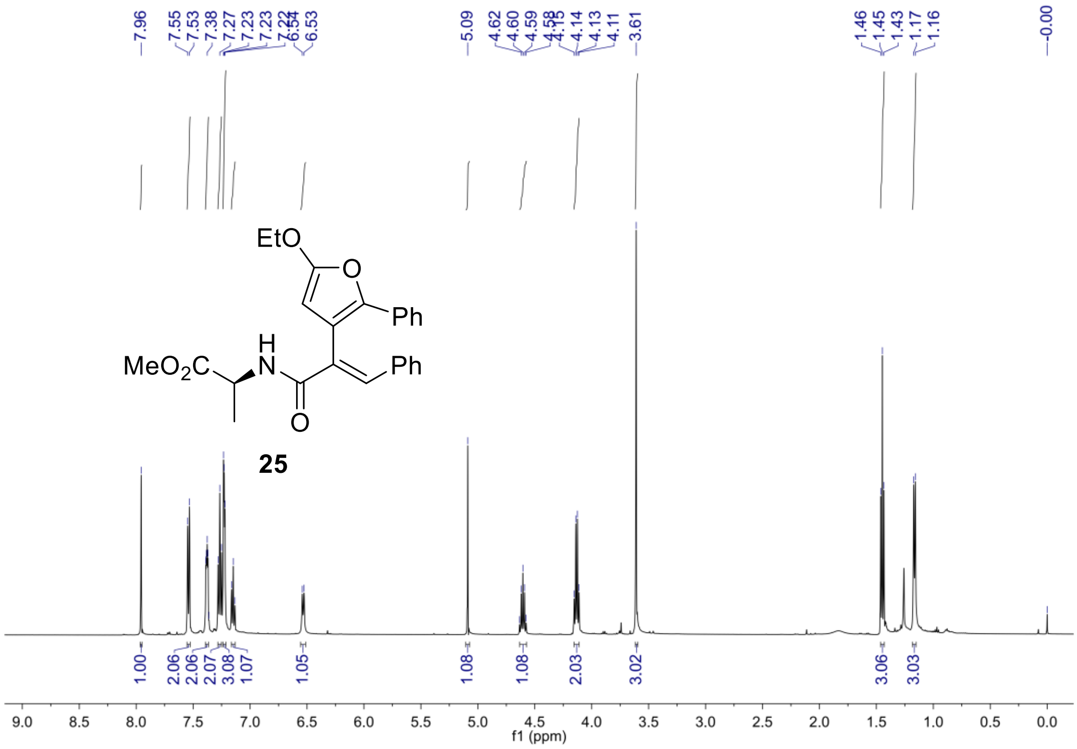

Supplementary Fig. 125 | ^{1}H NMR of compound 25 (500 MHz, at 25 °C, in $CDCl_3$).

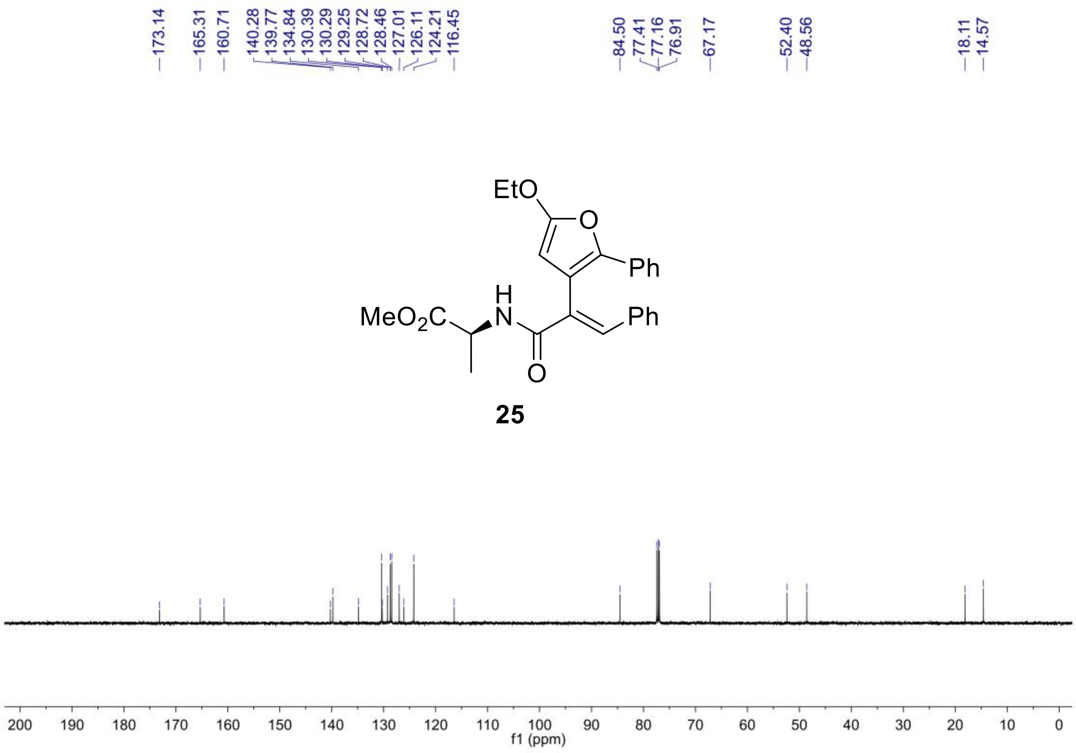

Supplementary Fig. 126 | ^{13}C NMR of compound 25 (125 MHz, at 25 °C, in $CDCl_3$).

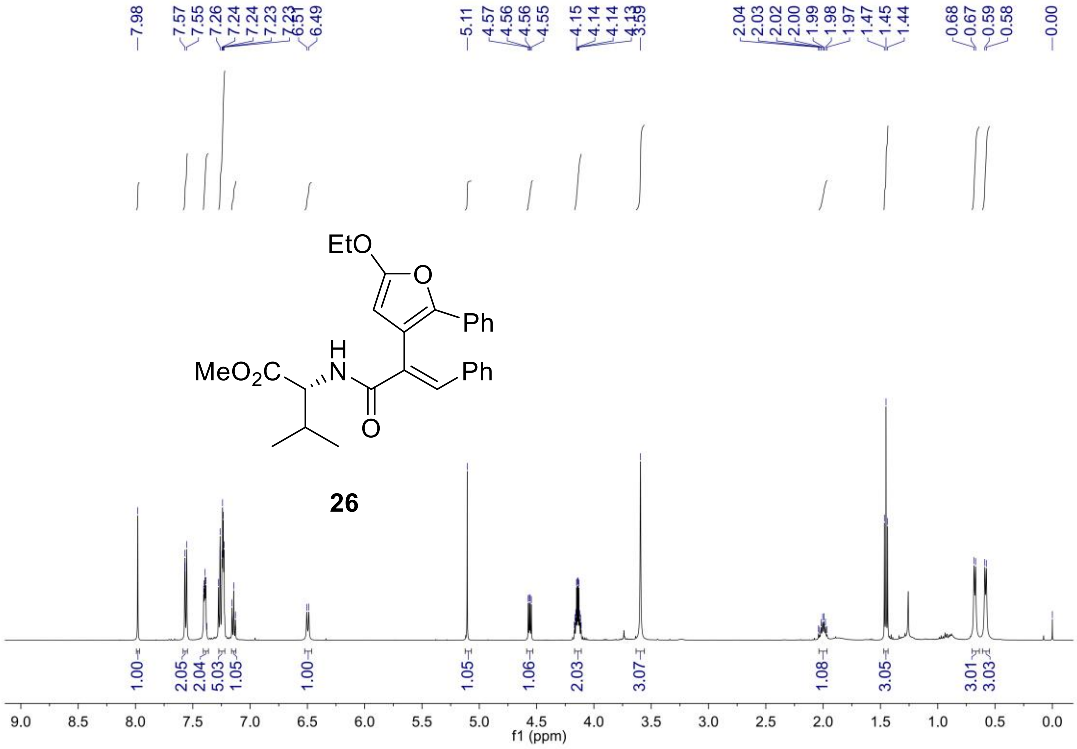

Supplementary Fig. 127 | ^{1}H NMR of compound 26 (500 MHz, at 25 °C, in CDCl3).

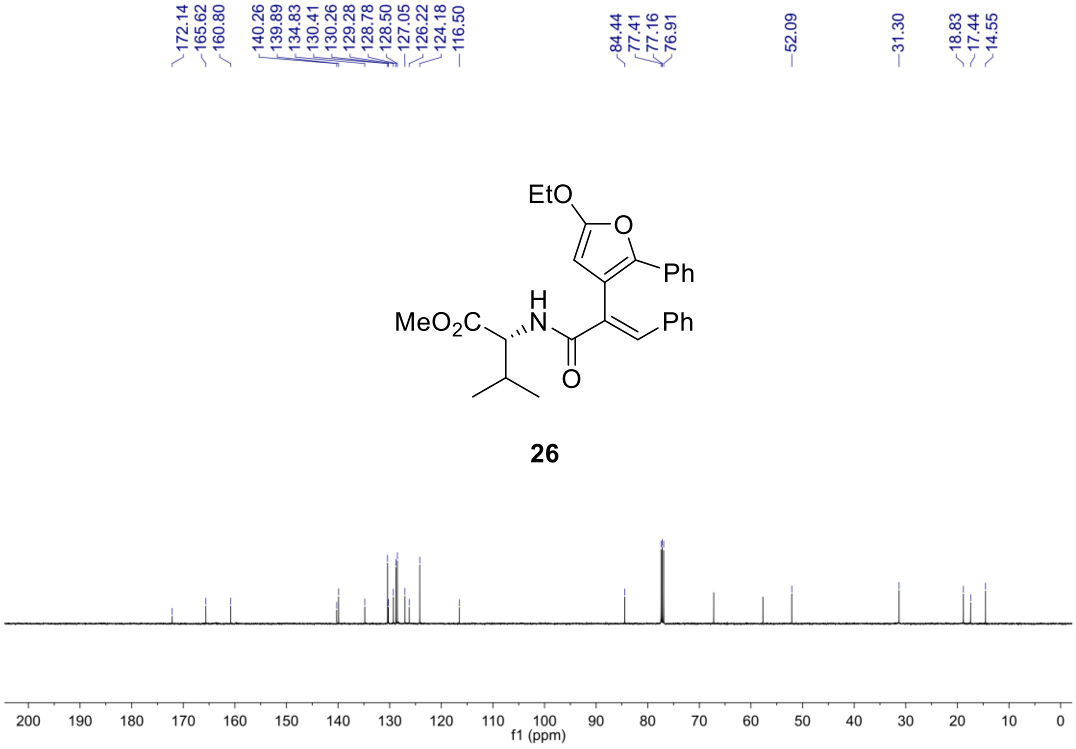

Supplementary Fig. 128 | ^{13}C NMR of compound 26 (125 MHz, at 25 °C, in CDCl3).

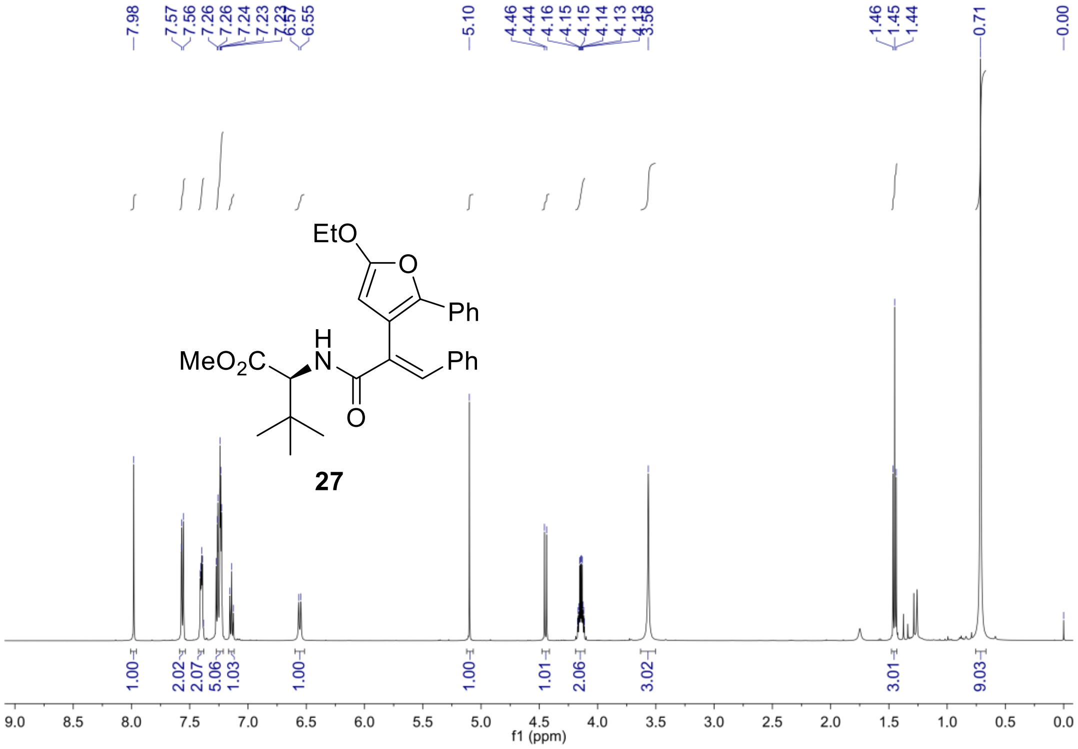

Supplementary Fig. 129 | ^{1}H NMR of compound 27 (500 MHz, at 25 °C, in $CDCl_3$).

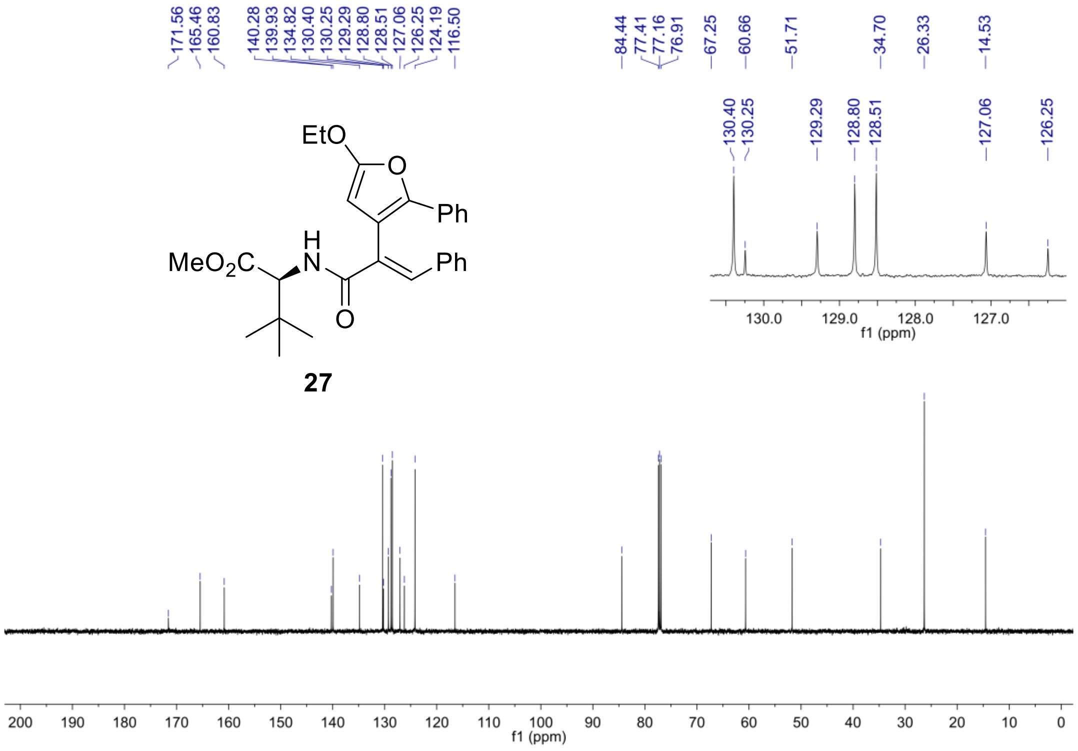

Supplementary Fig. 130 | ^{13}C NMR of compound 27 (125 MHz, at 25 °C, in $CDCl_3$).

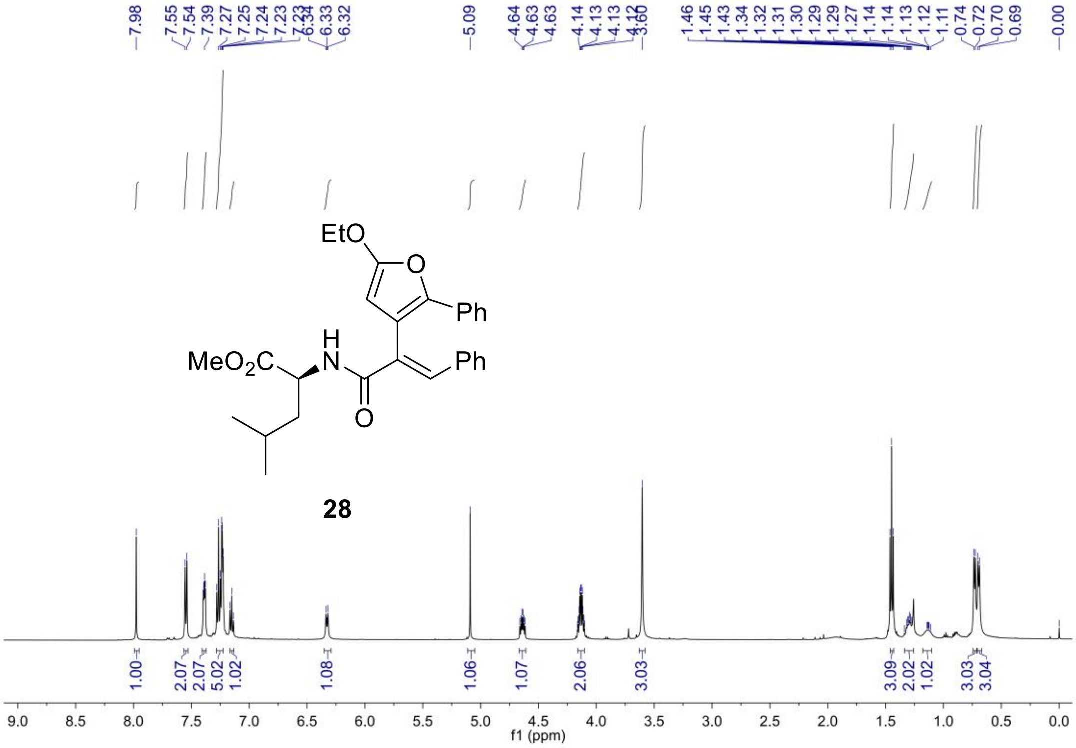

Supplementary Fig. 131 | ^{1}H NMR of compound 28 (500 MHz, at 25 °C, in $CDCl_3$).

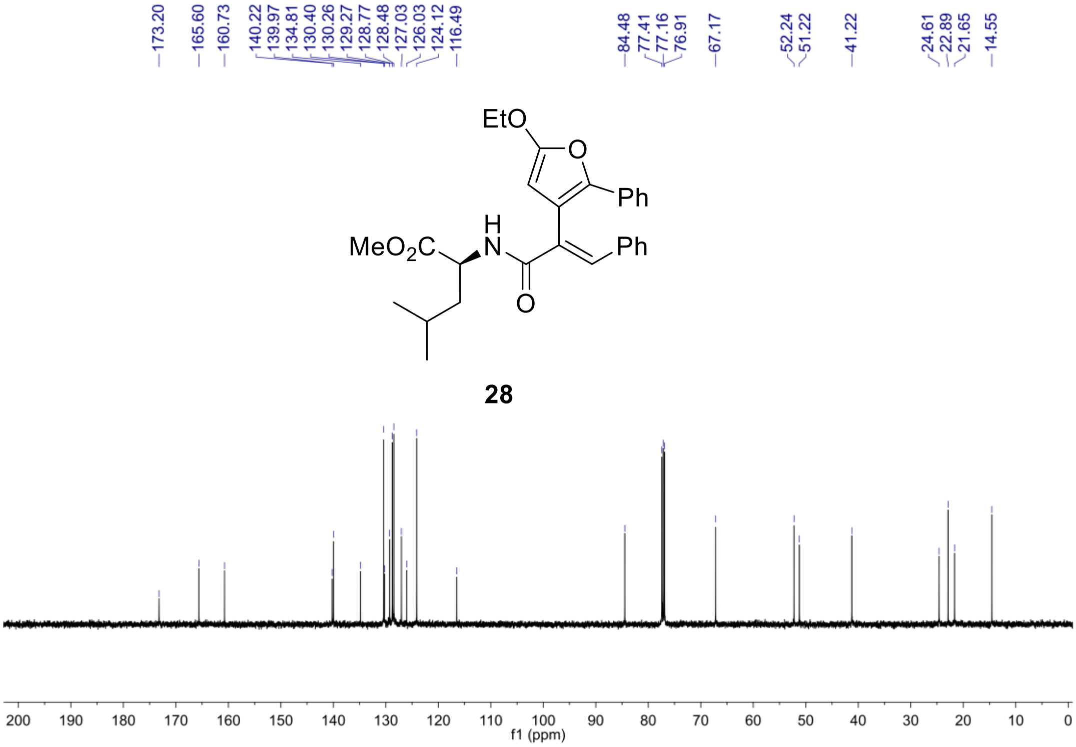

Supplementary Fig. 132 | ^{13}C NMR of compound 28 (125 MHz, at 25 °C, in $CDCl_3$).

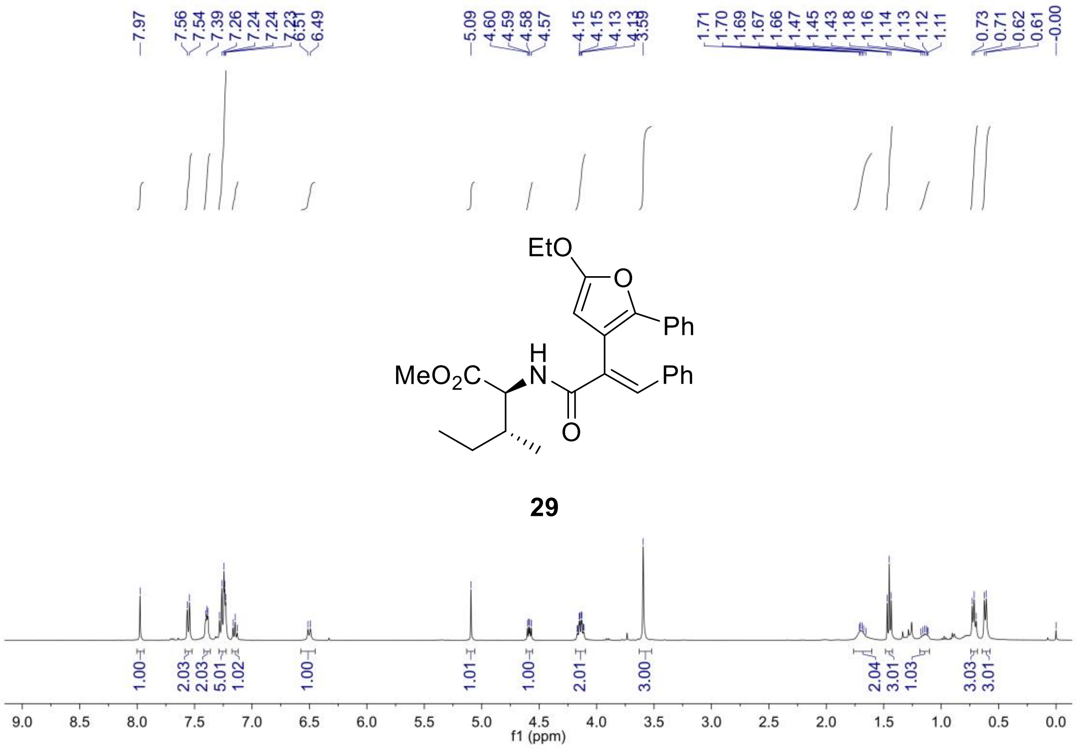

Supplementary Fig. 133 | ^{1}H NMR of compound 29 (400 MHz, at 25 ^{o}C, in $CDCl_3$).

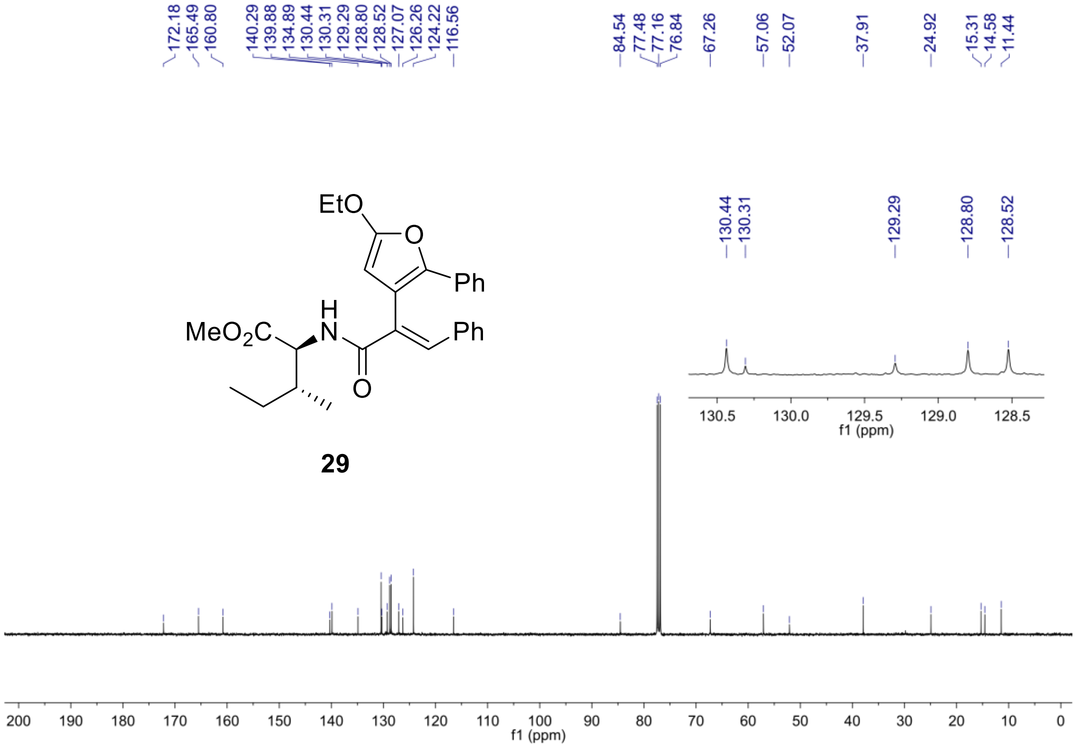

Supplementary Fig. 134 | ^{13}C NMR of compound 29 (100 MHz, at 25 ^{o}C, in $CDCl_3$).

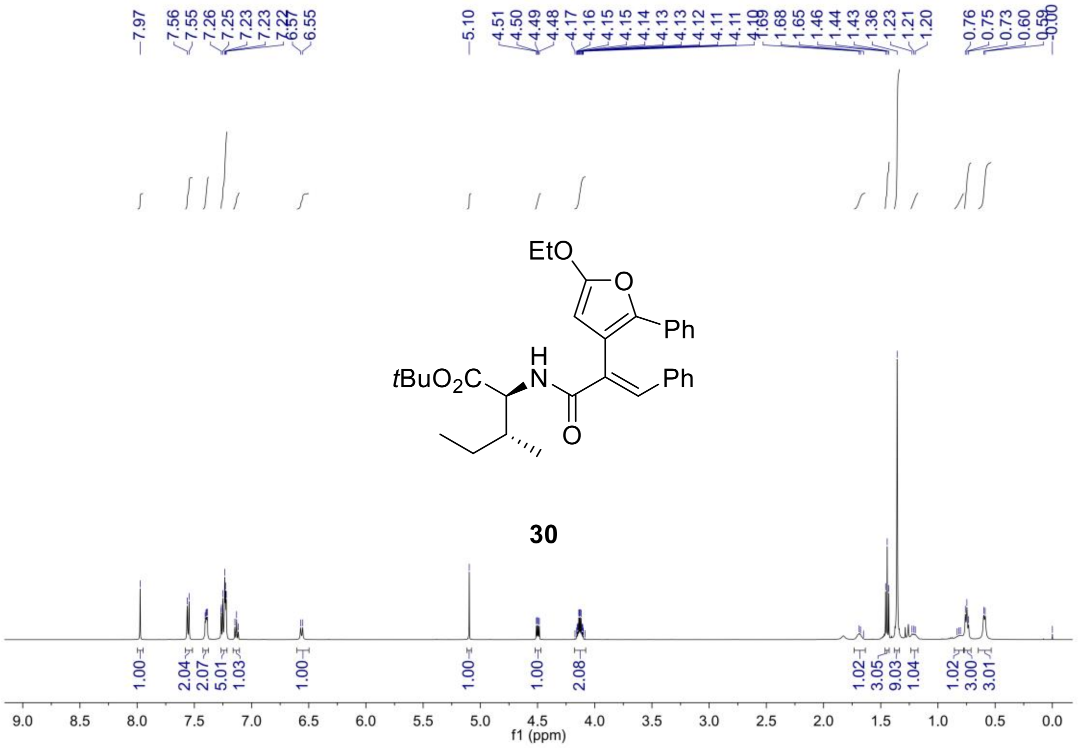

Supplementary Fig. 135 | ^{1}H NMR of compound 30 (500 MHz, at 25 ºC, in $CDCl_3$).

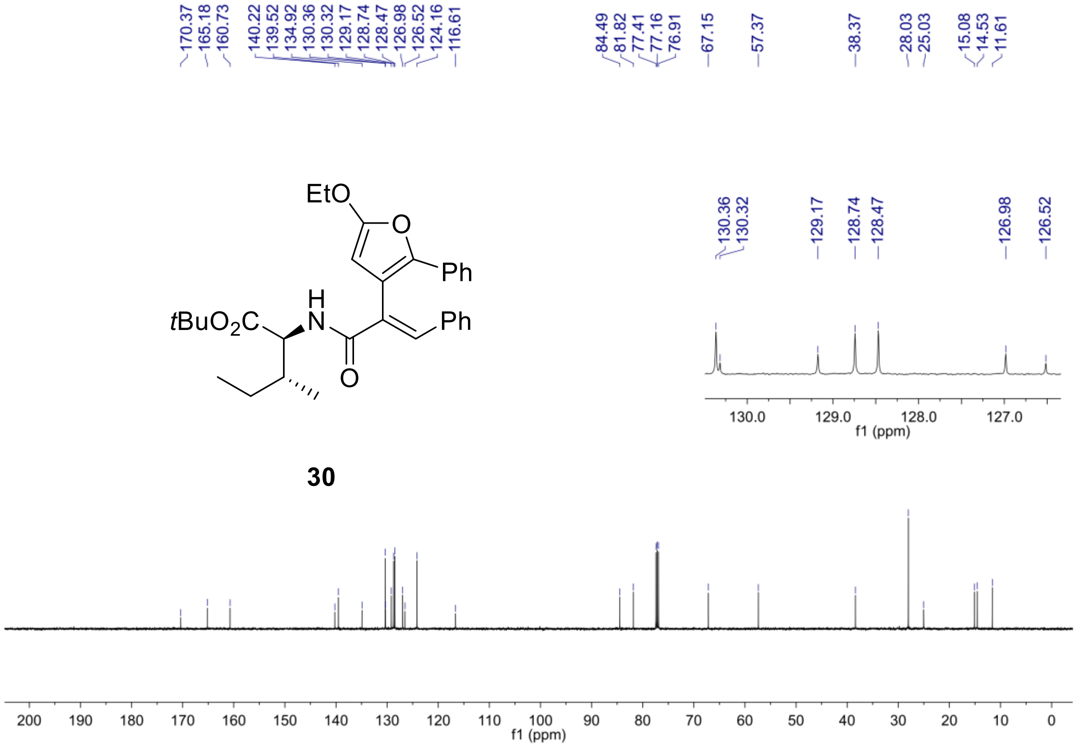

Supplementary Fig. 136 | ^{13}C NMR of compound 30 (125 MHz, at 25 ºC, in $CDCl_3$).

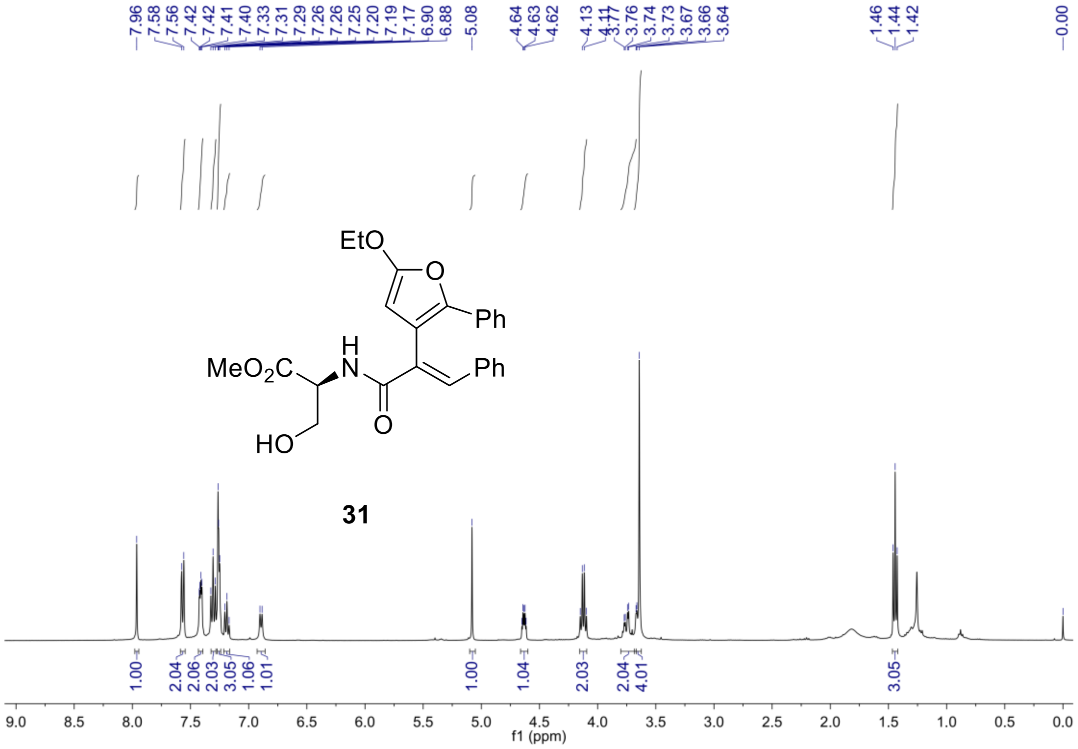

Supplementary Fig. 137 | ^{1}H NMR of compound 31 (400 MHz, at 25 °C, in $CDCl_3$).

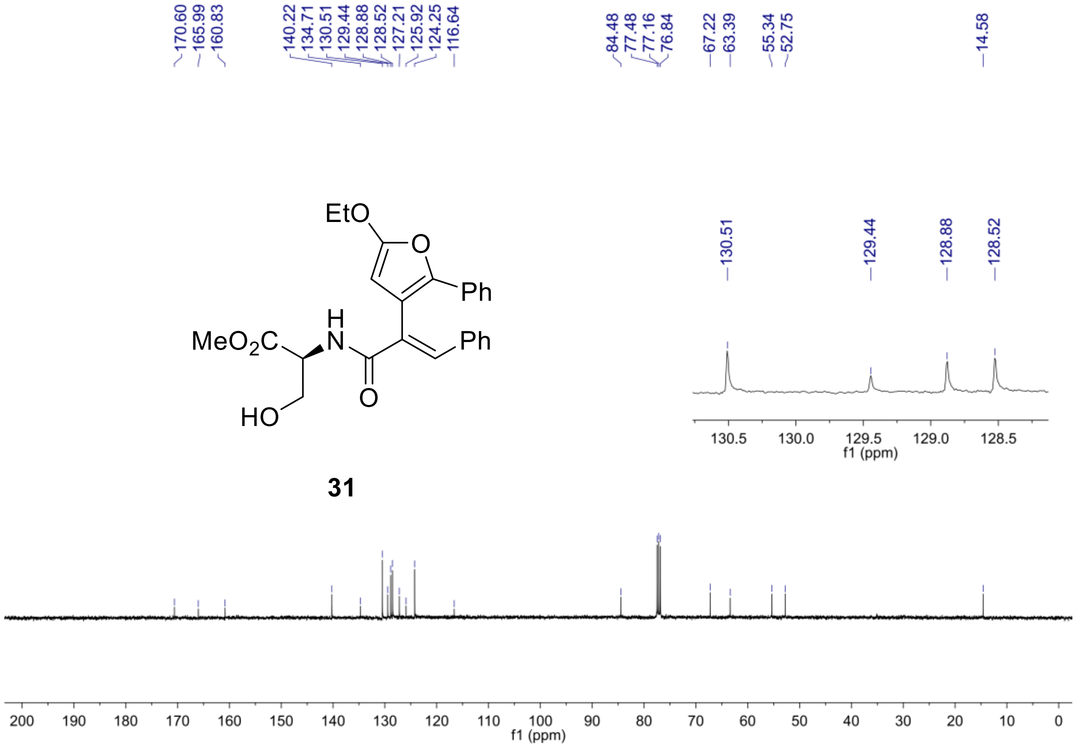

Supplementary Fig. 138 | ^{13}C NMR of compound 31 (100 MHz, at 25 °C, in $CDCl_3$).

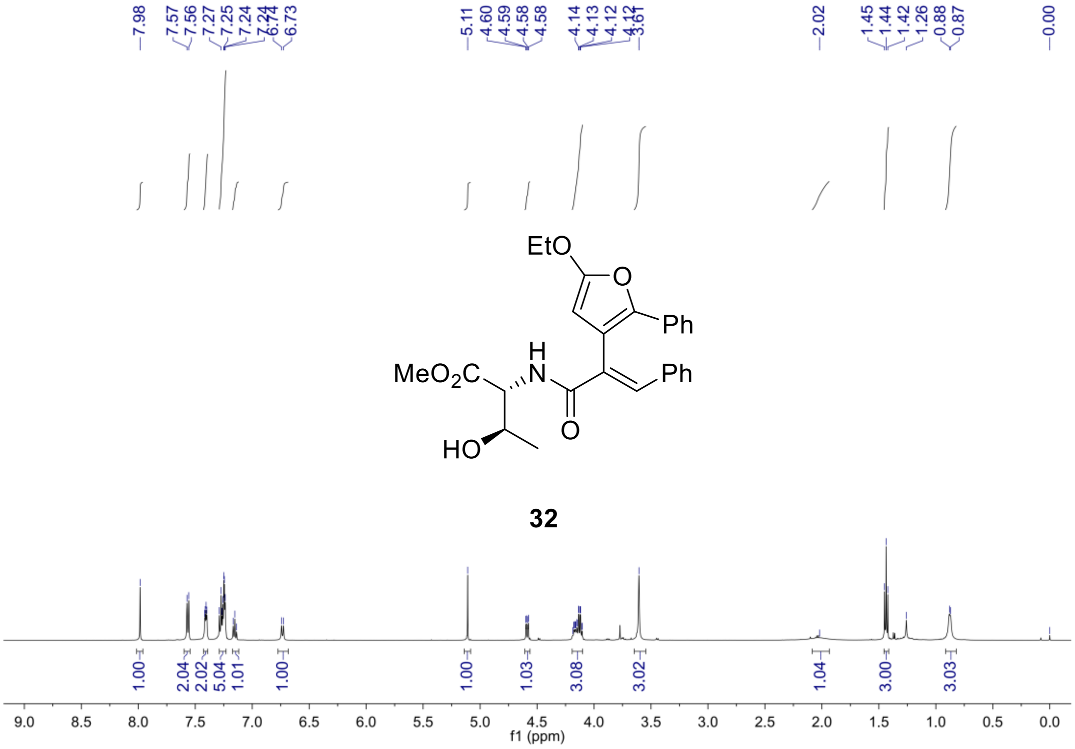

Supplementary Fig. 139 | ^{1}H NMR of compound 32 (500 MHz, at 25 ºC, in $CDCl_3$).

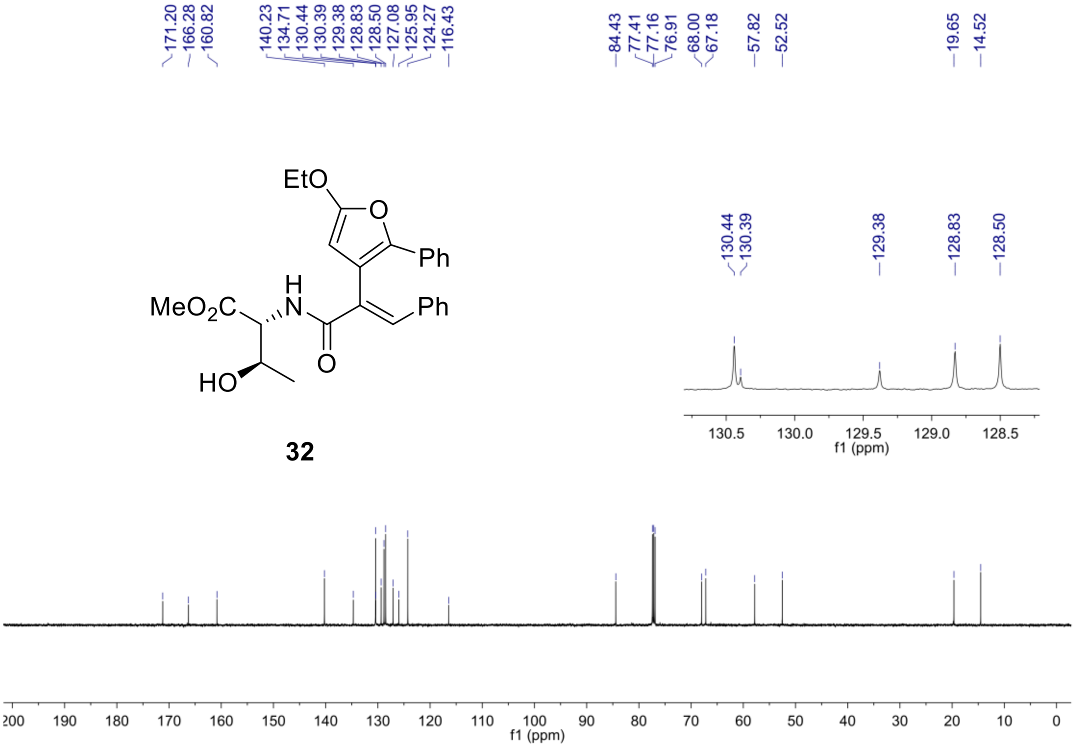

Supplementary Fig. 140 | ^{13}C NMR of compound 32 (125 MHz, at 25 ºC, in $CDCl_3$).

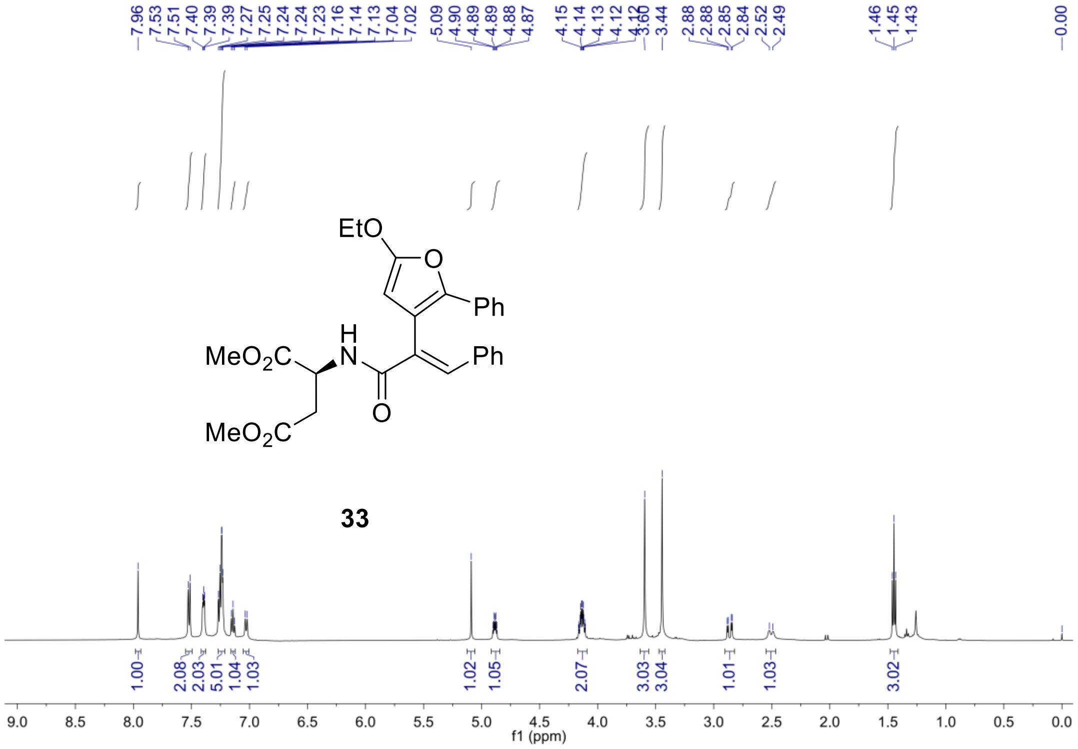

Supplementary Fig. 141 | ^{1}H NMR of compound 33 (500 MHz, at 25 °C, in $CDCl_3$).

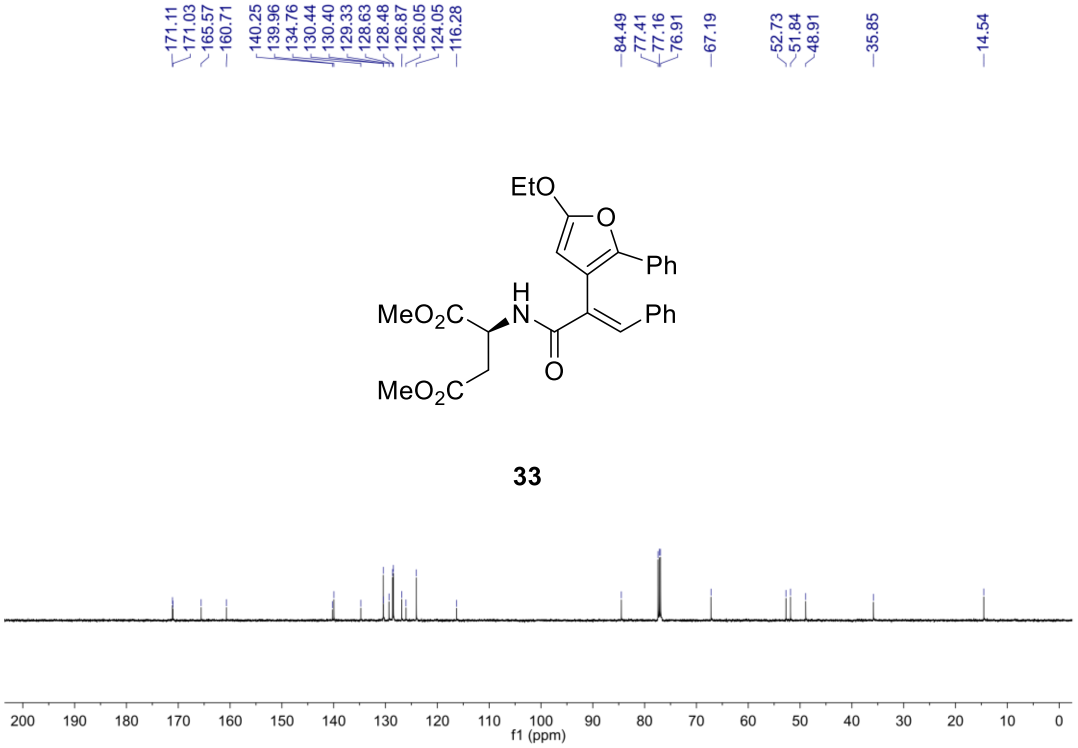

Supplementary Fig. 142 | ^{13}C NMR of compound 33 (125 MHz, at 25 °C, in $CDCl_3$).

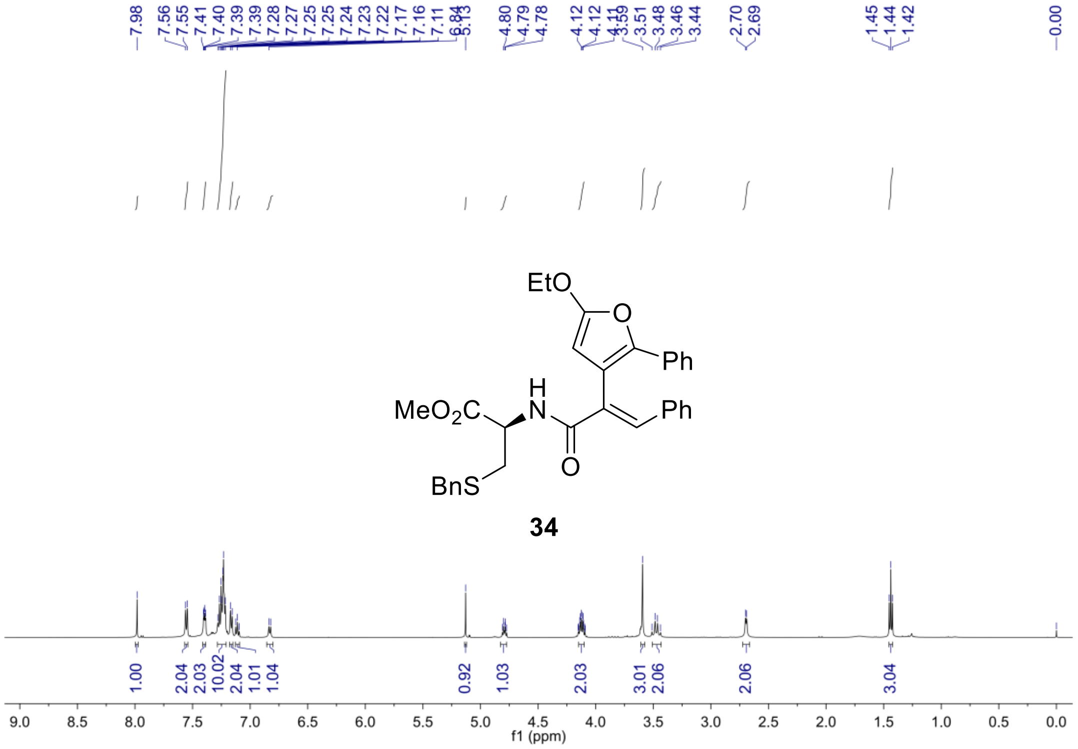

Supplementary Fig. 143 | ^{1}H NMR of compound 34 (500 MHz, at 25 °C, in $CDCl_3$).

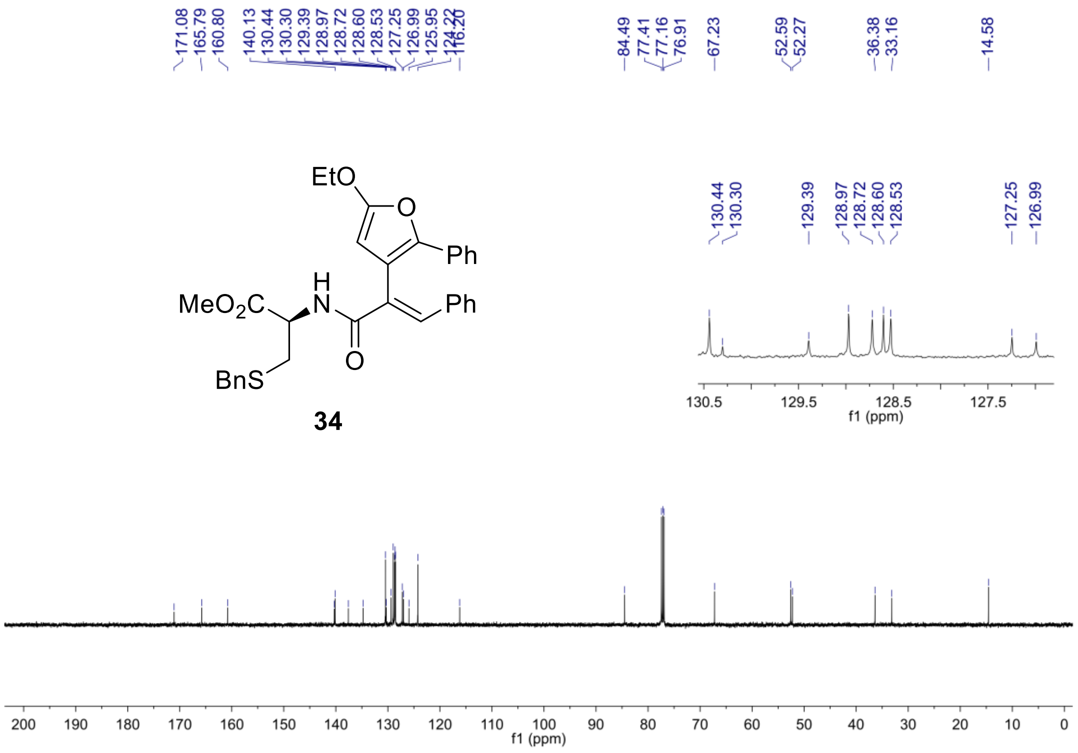

Supplementary Fig. 144 | ^{13}C NMR of compound 34 (125 MHz, at 25 °C, in $CDCl_3$).

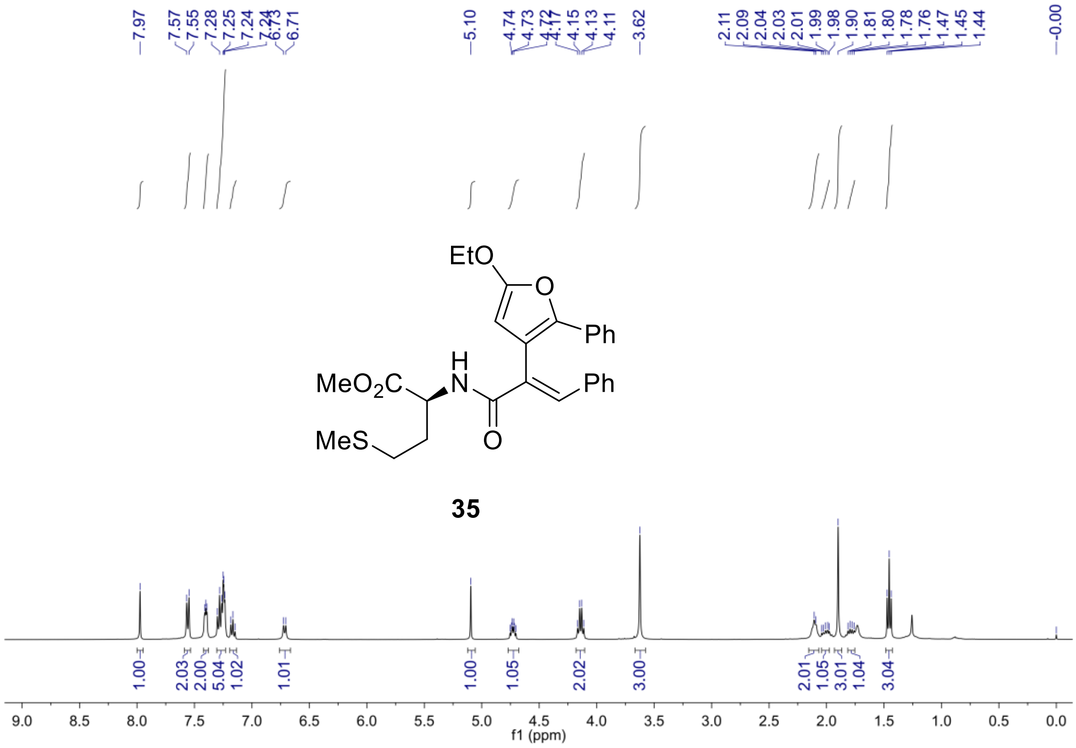

35

Supplementary Fig. 145 | ^{1}H NMR of compound 35 (400 MHz, at 25 ^{o}C, in $CDCl_3$).

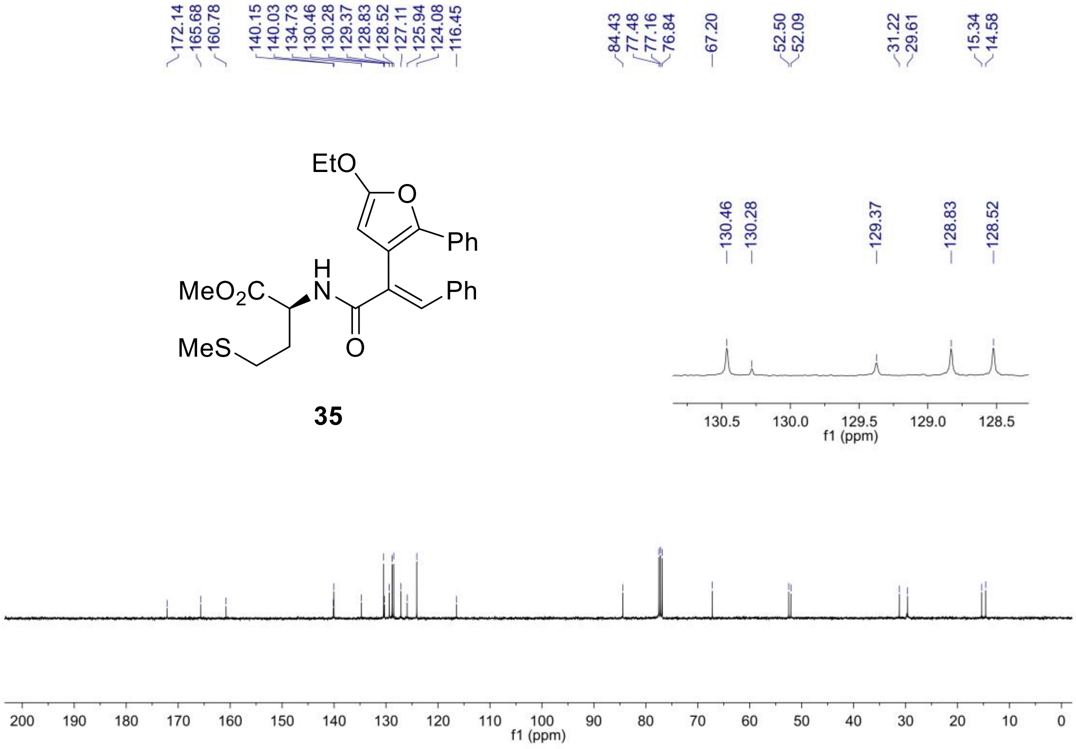

35

Supplementary Fig. 146 | ^{13}C NMR of compound 35 (100 MHz, at 25 ^{o}C, in $CDCl_3$).

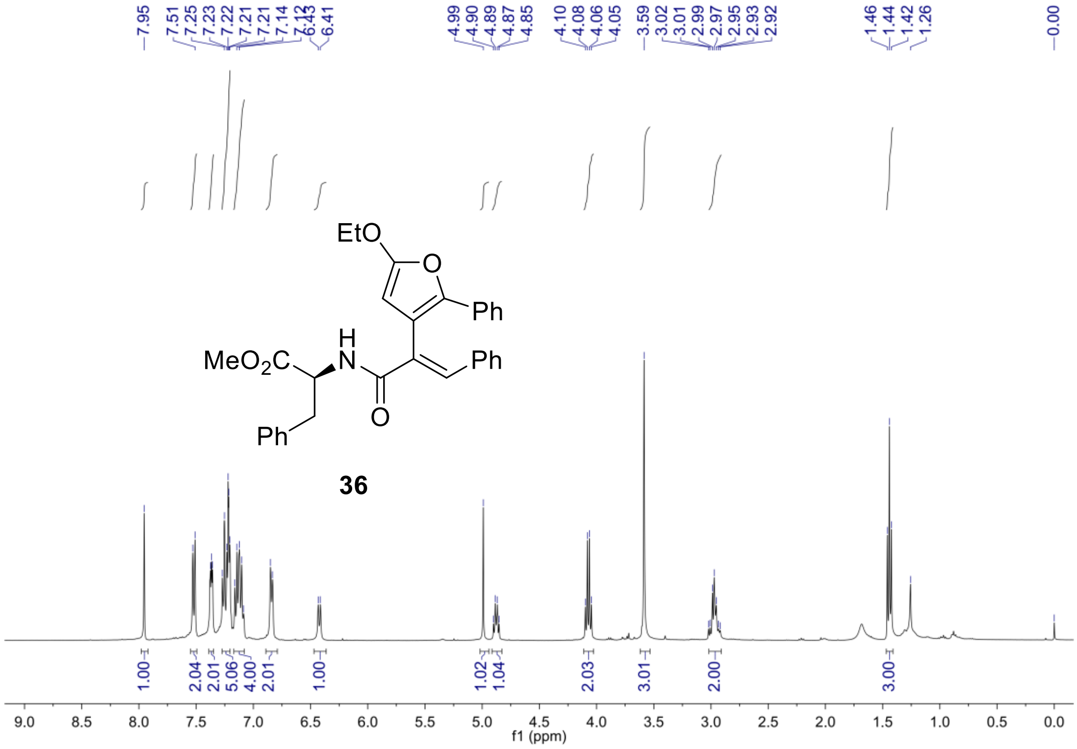

Supplementary Fig. 147 | ^{1}H NMR of compound 36 (400 MHz, at 25 ^oC, in $CDCl_3$).

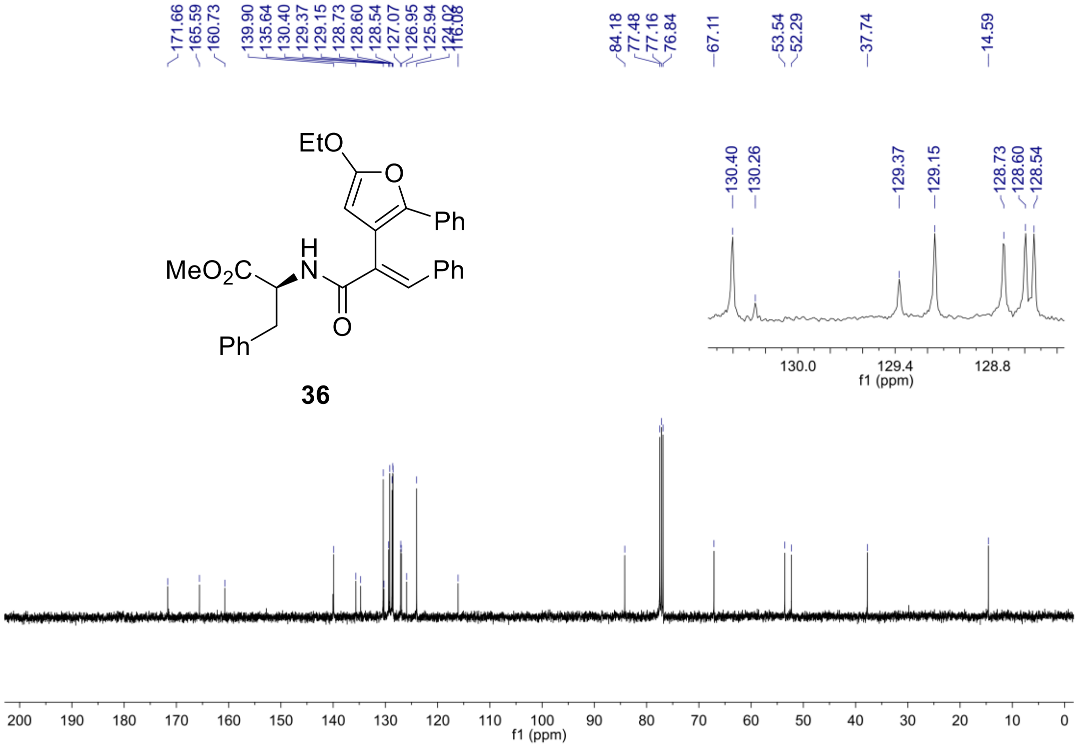

Supplementary Fig. 148 | ^{13}C NMR of compound 36 (100 MHz, at 25 ^oC, in $CDCl_3$).

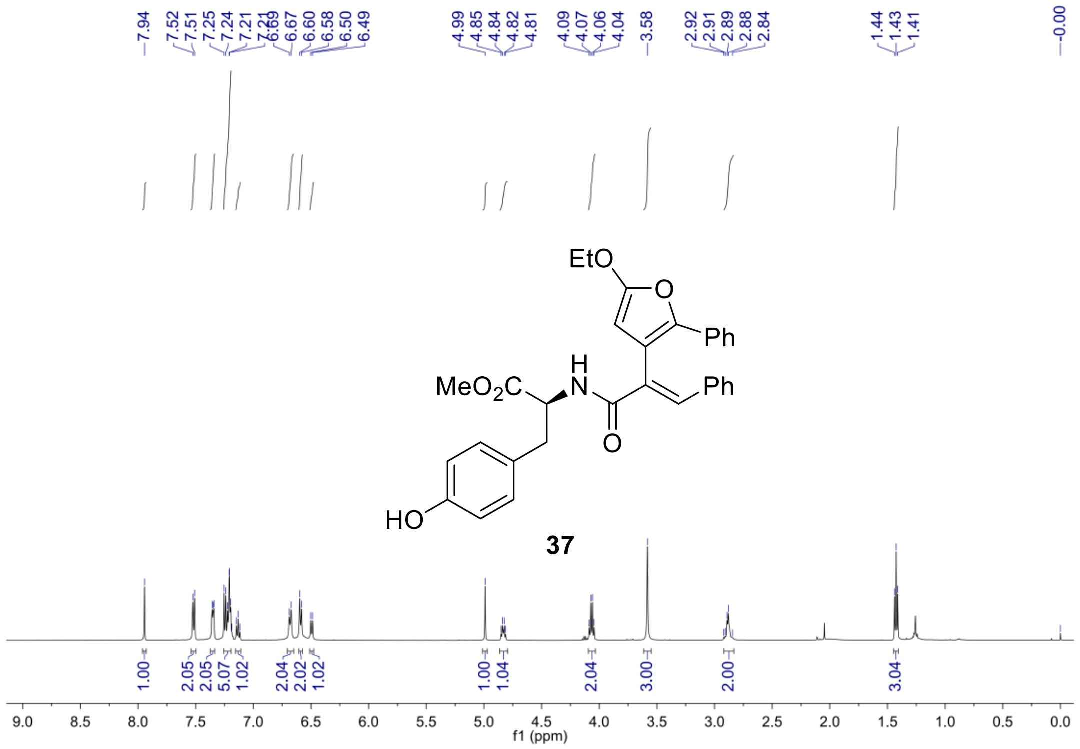

Supplementary Fig. 149 | ^{1}H NMR of compound 37 (500 MHz, at 25 °C, in $CDCl_3$).

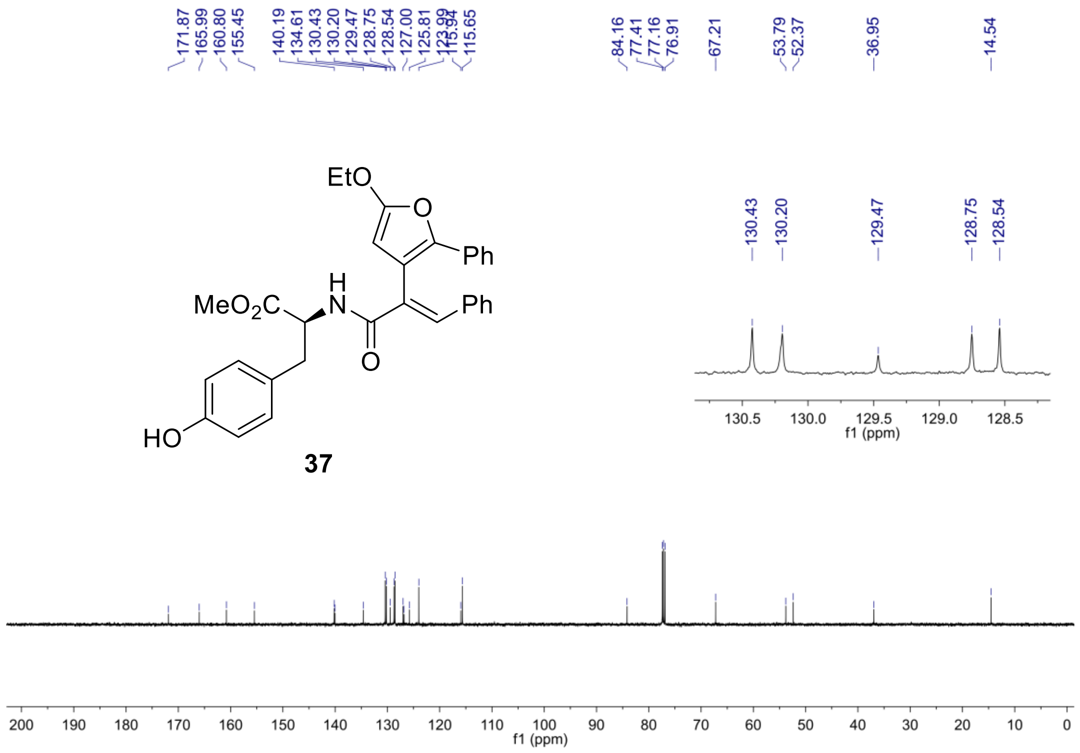

Supplementary Fig. 150 | ^{13}C NMR of compound 37 (125 MHz, at 25 °C, in $CDCl_3$).

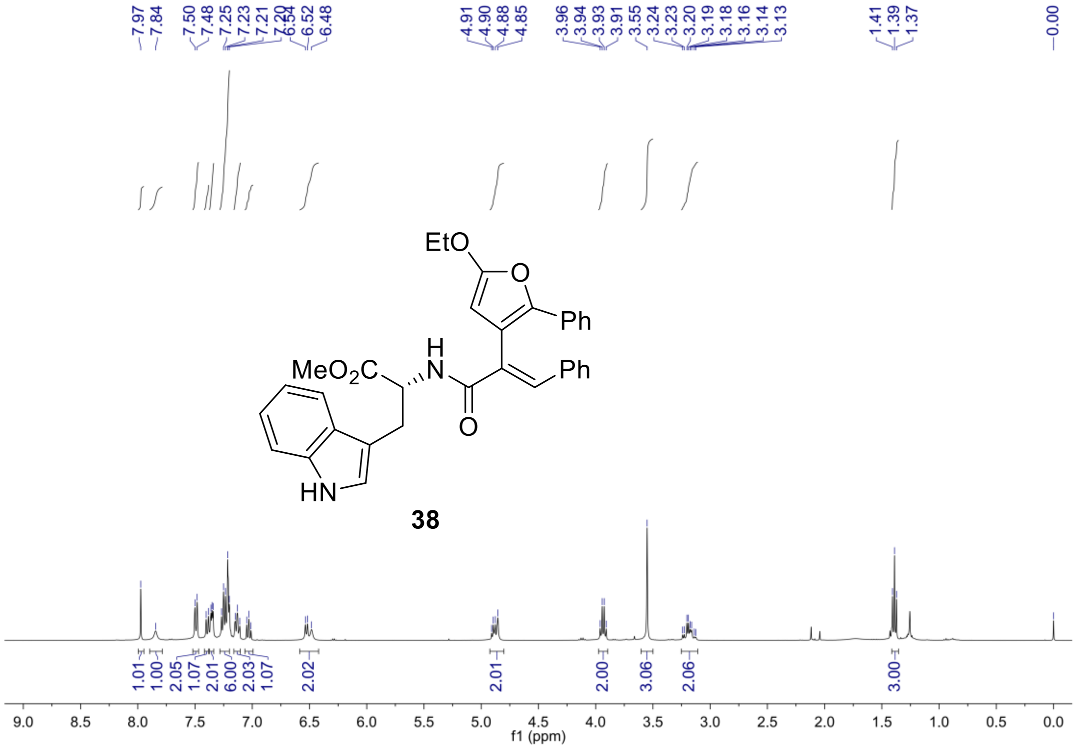

Supplementary Fig. 151 | ^{1}H NMR of compound 38 (400 MHz, at 25 °C, in $CDCl_3$).

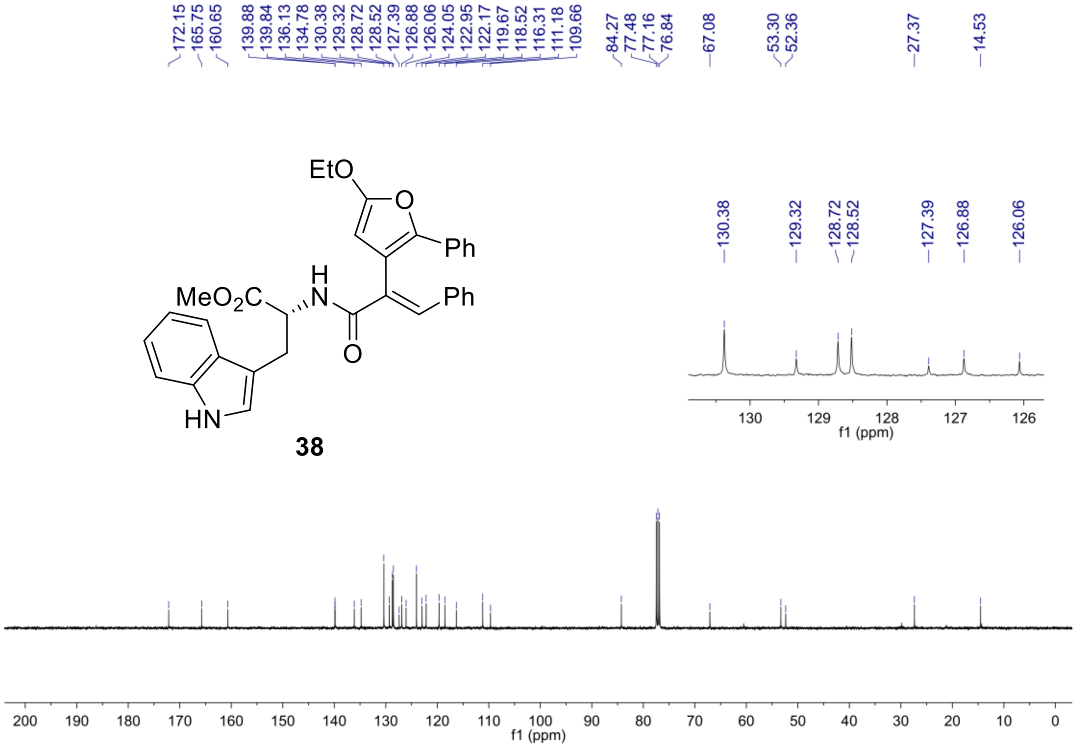

Supplementary Fig. 152 | ^{13}C NMR of compound 38 (100 MHz, at 25 °C, in $CDCl_3$).

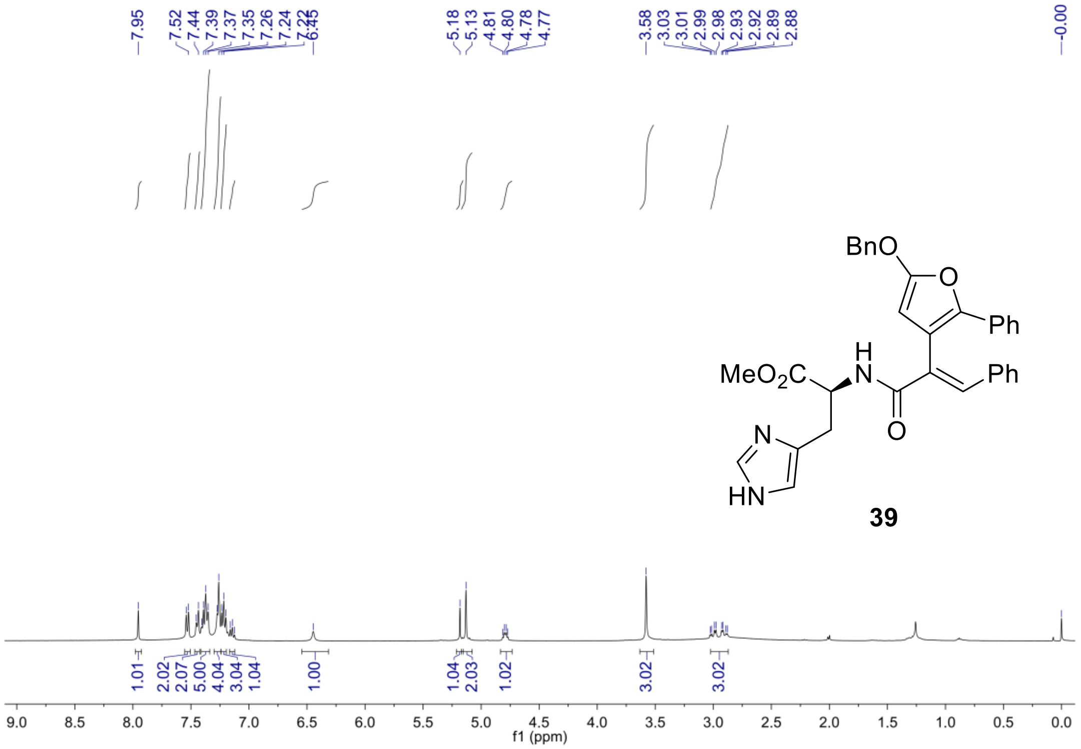

Supplementary Fig. 153 | ^{1}H NMR of compound 39 (400 MHz, at 25 °C, in $CDCl_3$).

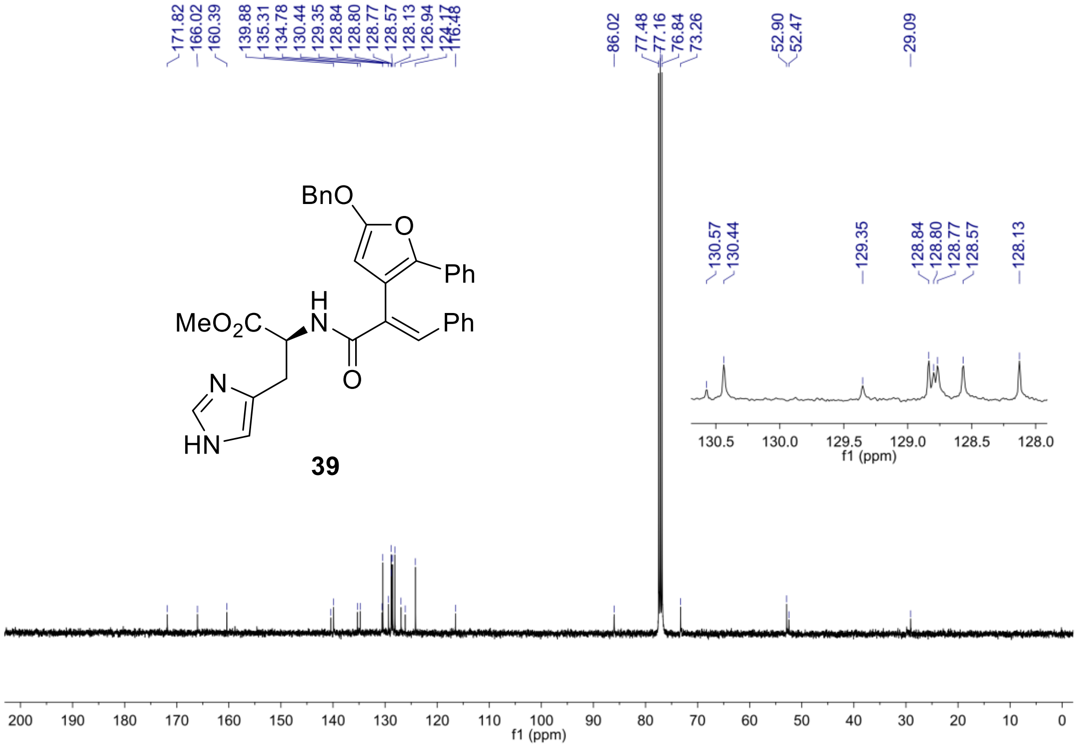

Supplementary Fig. 154 | ^{13}C NMR of compound 39 (100 MHz, at 25 °C, in $CDCl_3$).

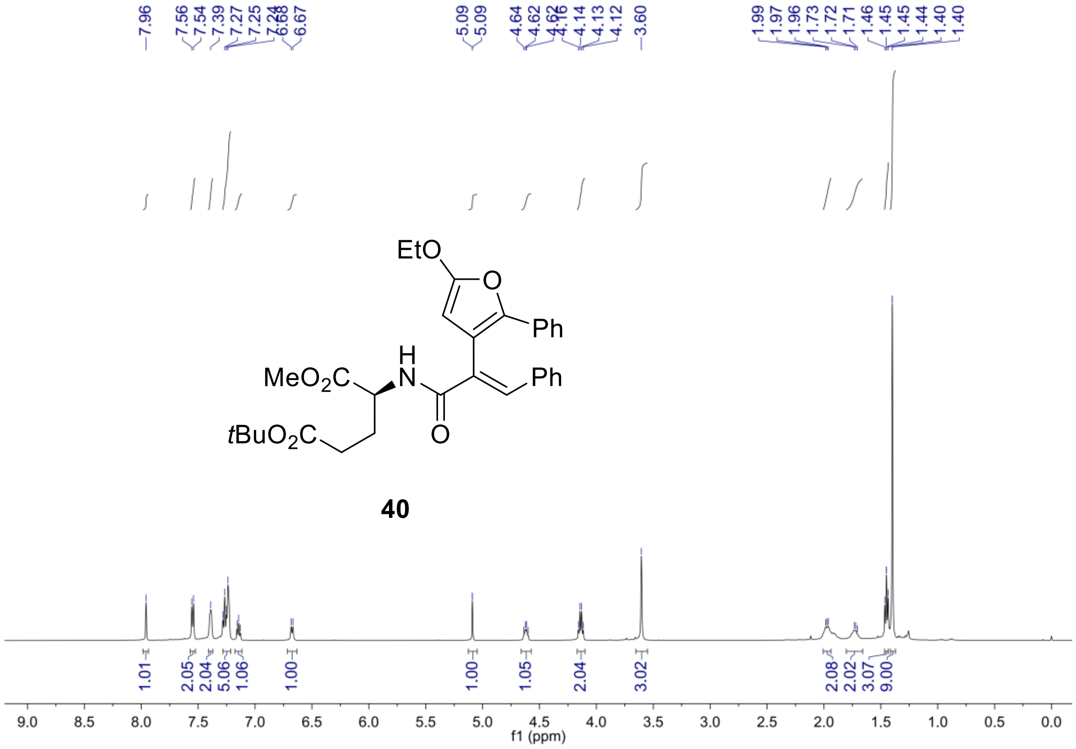

Supplementary Fig. 155 | ^{1}H NMR of compound 40 (500 MHz, at 25 °C, in $CDCl_3$).

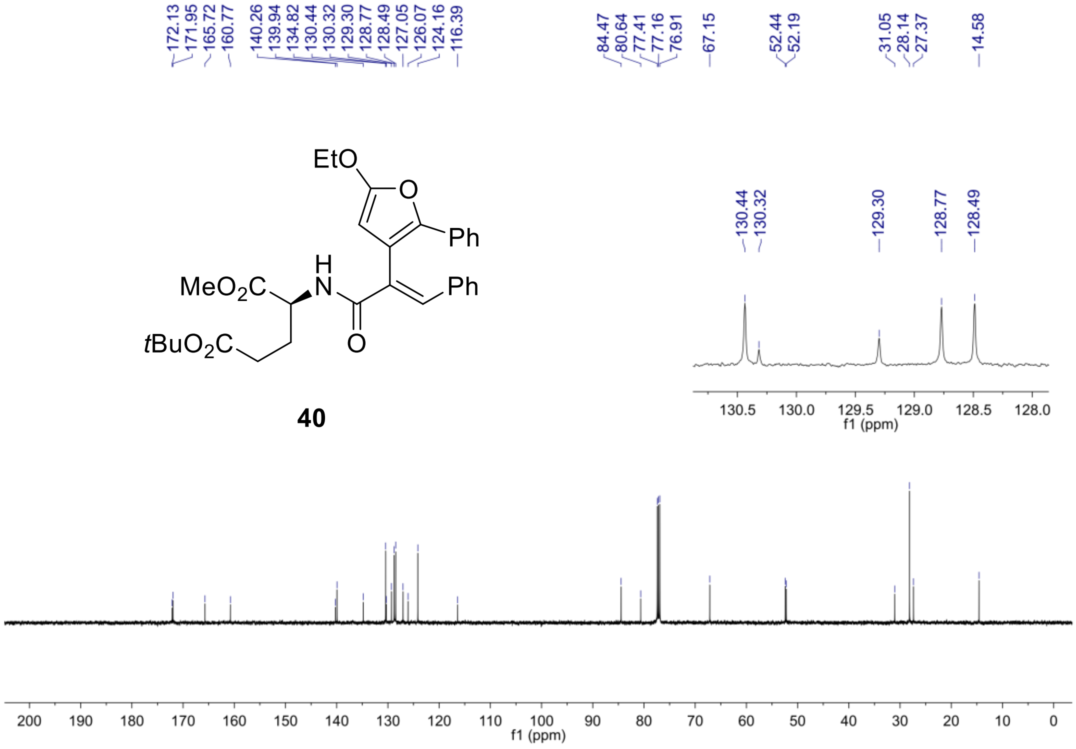

Supplementary Fig. 156 | ^{13}C NMR of compound 40 (125 MHz, at 25 °C, in $CDCl_3$).

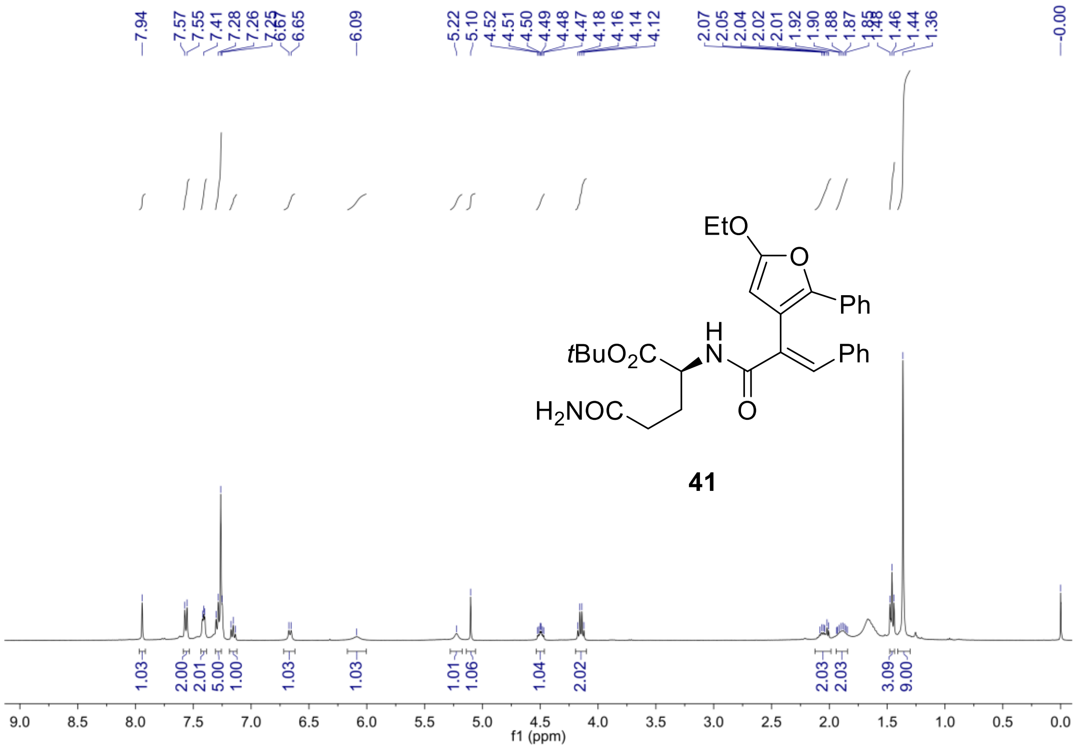

Supplementary Fig. 157 | ^{1}H NMR of compound 41 (400 MHz, at 25 °C, in $CDCl_3$).

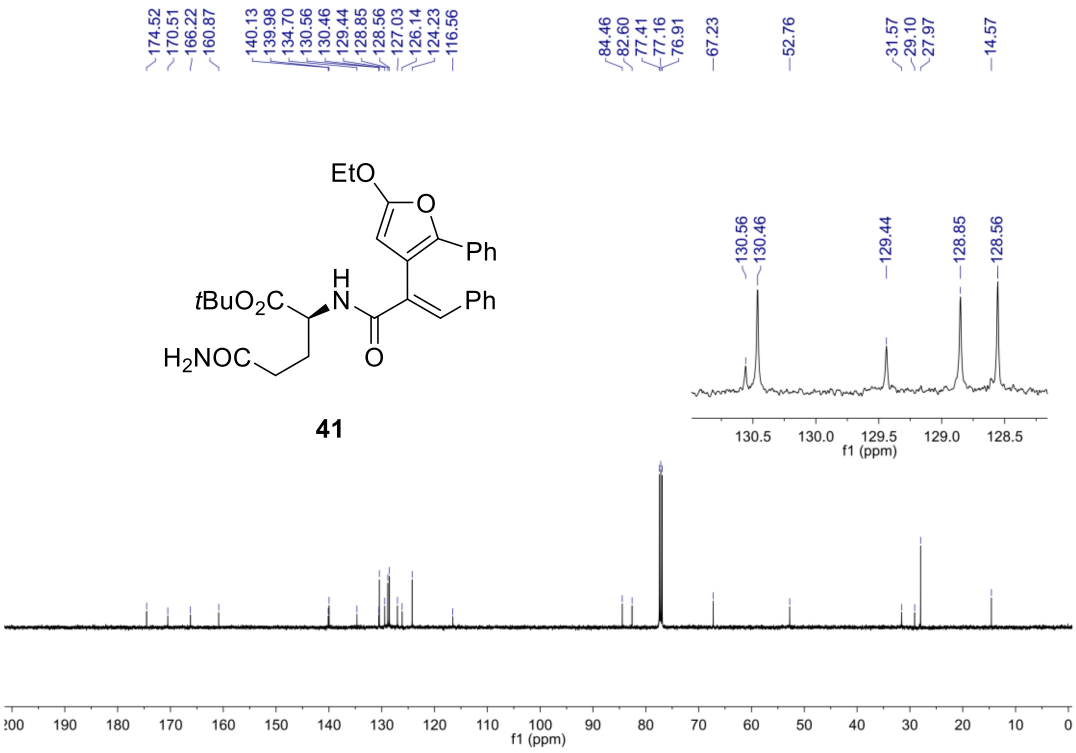

Supplementary Fig. 158 | ^{13}C NMR of compound 41 (125 MHz, at 25 °C, in $CDCl_3$).

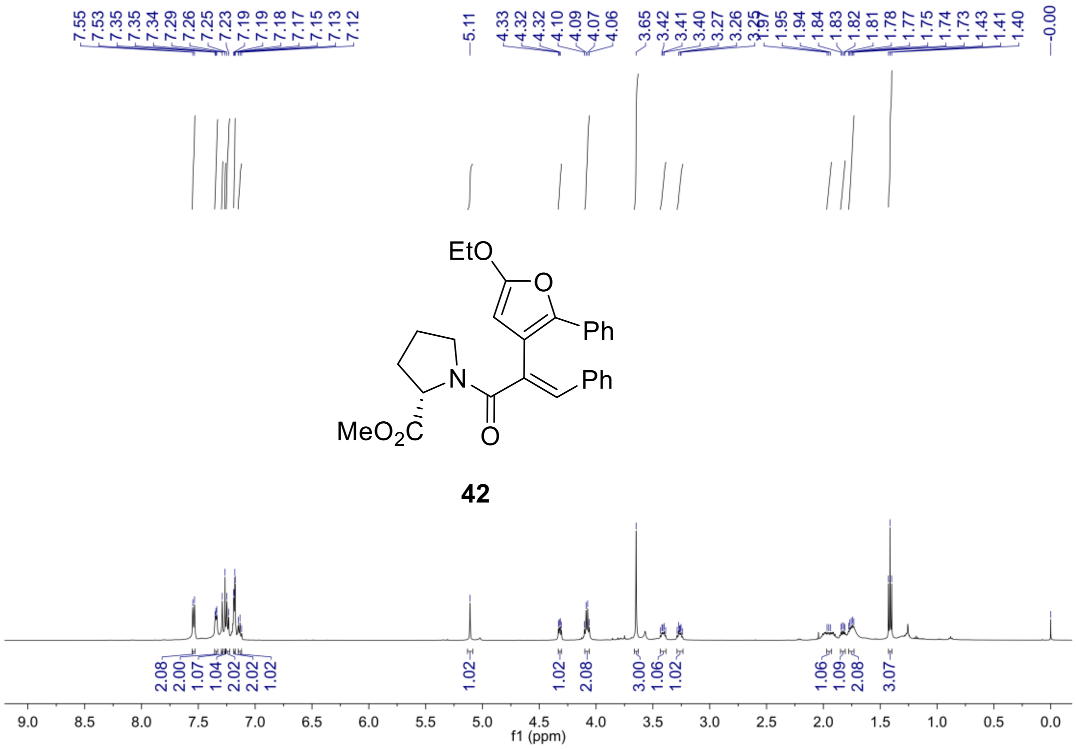

Supplementary Fig. 159 | ^{1}H NMR of compound 42 (500 MHz, at 25 °C, in $CDCl_3$).

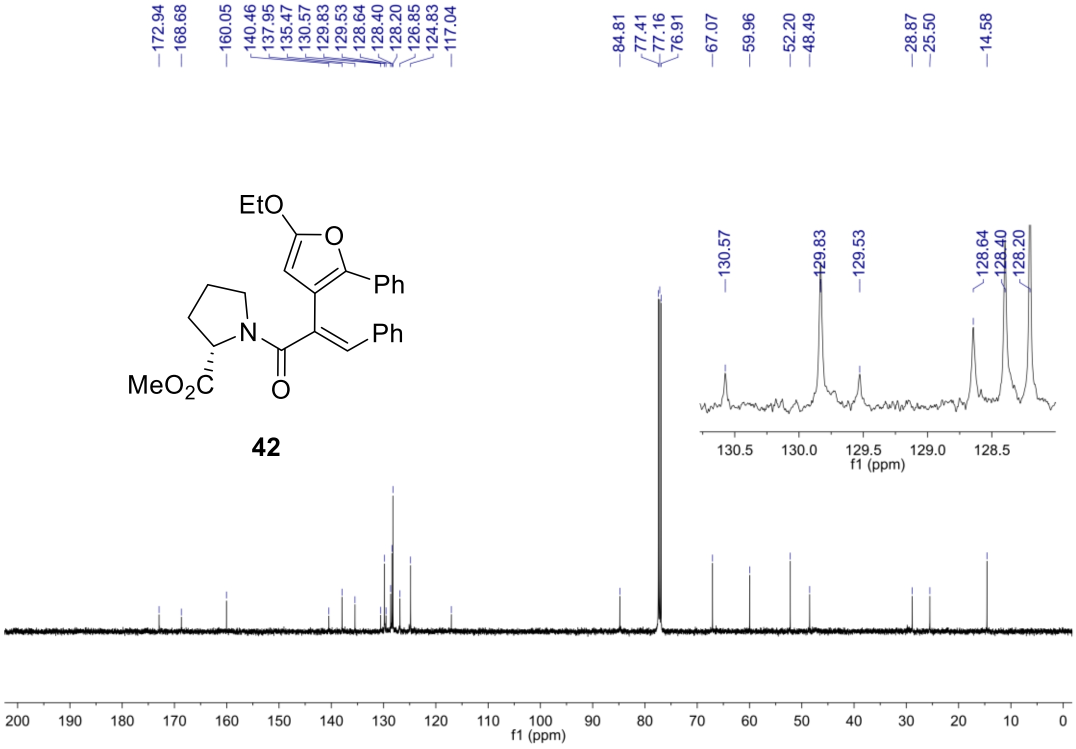

Supplementary Fig. 160 | ^{13}C NMR of compound 42 (125 MHz, at 25 °C, in $CDCl_3$).

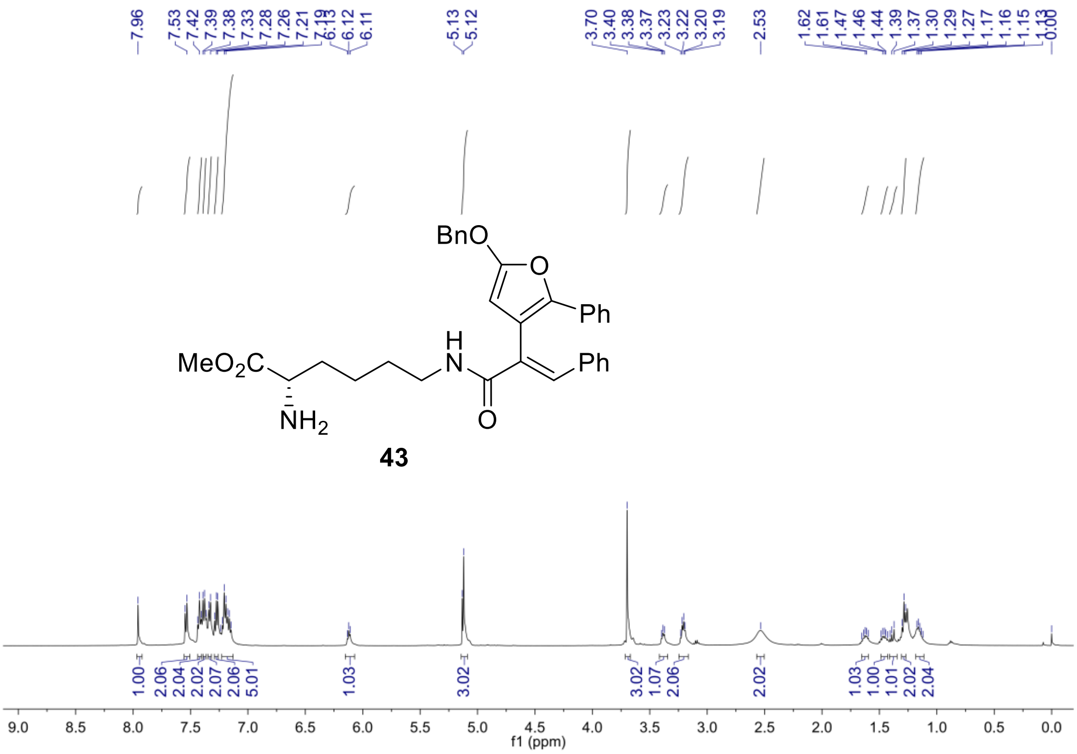

Supplementary Fig. 161 | ^{1}H NMR of compound 43 (500 MHz, at 25 °C, in $CDCl_3$).

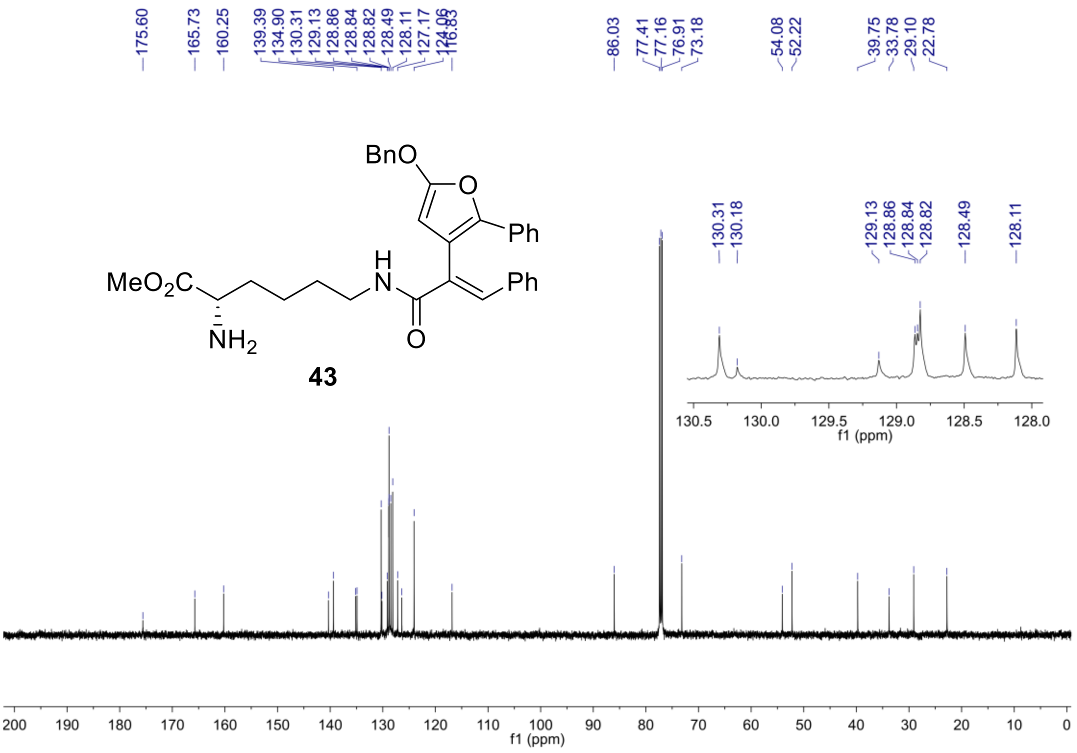

Supplementary Fig. 162 | ^{13}C NMR of compound 43 (125 MHz, at 25 °C, in $CDCl_3$).

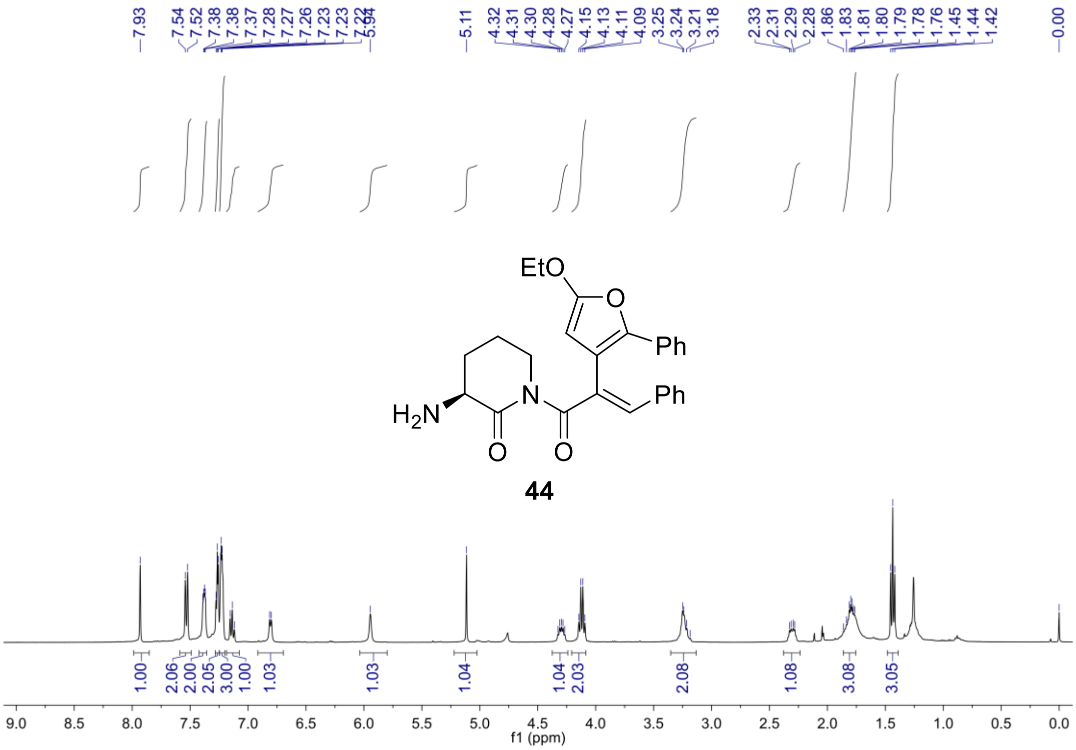

Supplementary Fig. 163 | ^{1}H NMR of compound 44 (400 MHz, at 25 °C, in $CDCl_3$).

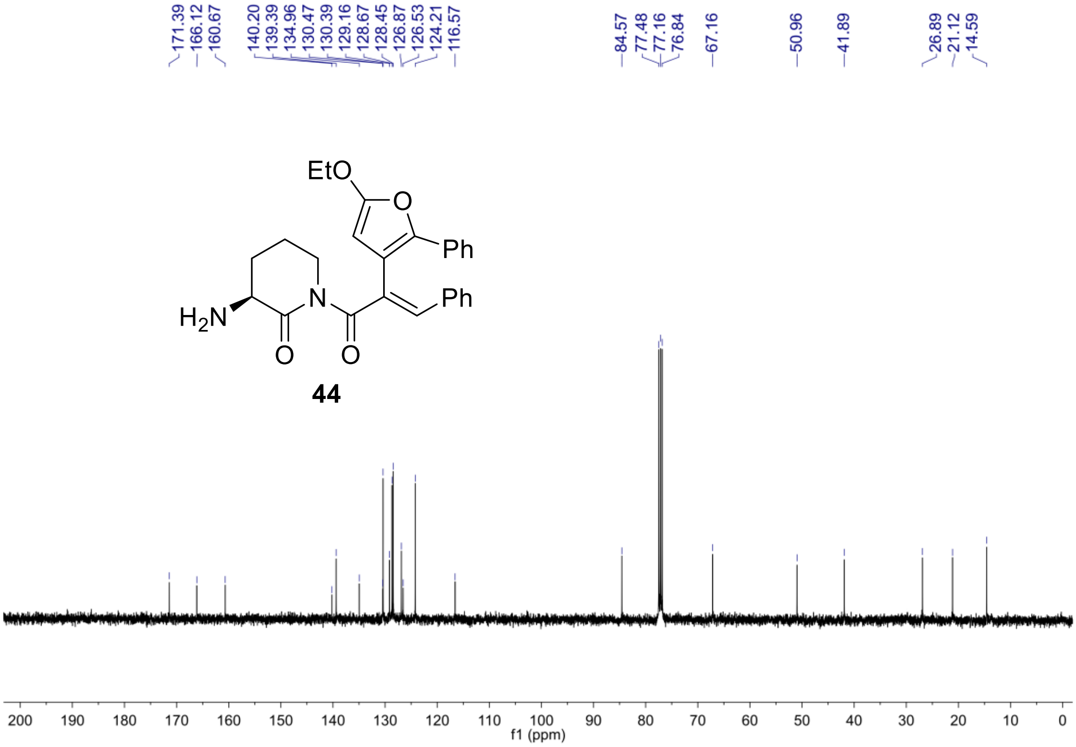

Supplementary Fig. 164 | ^{13}C NMR of compound 44 (100 MHz, at 25 °C, in $CDCl_3$).

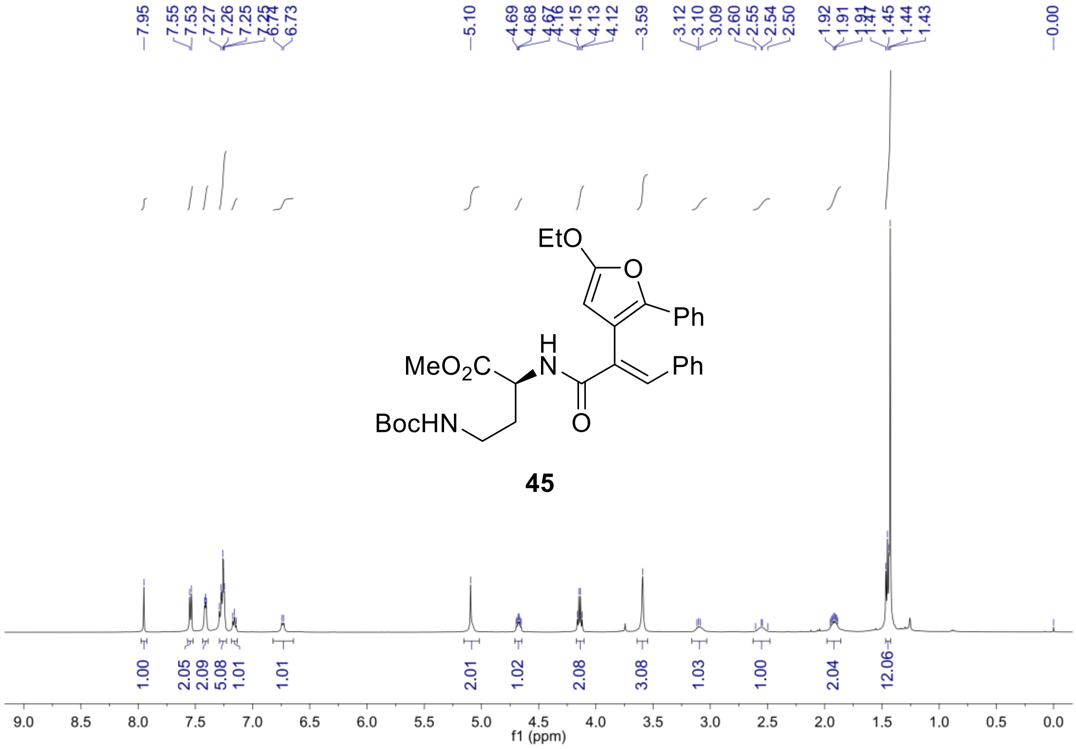

Supplementary Fig. 165 | ^{1}H NMR of compound 45 (500 MHz, at 25 °C, in $CDCl_3$).

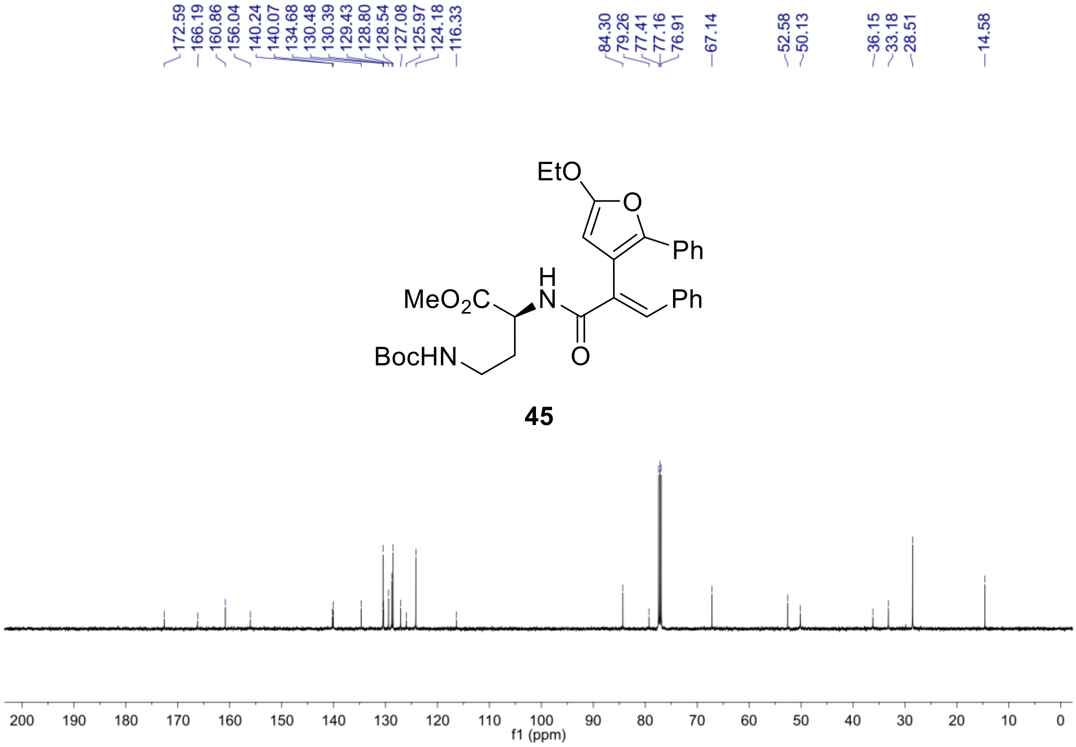

Supplementary Fig. 166 | ^{13}C NMR of compound 45 (125 MHz, at 25 °C, in $CDCl_3$).

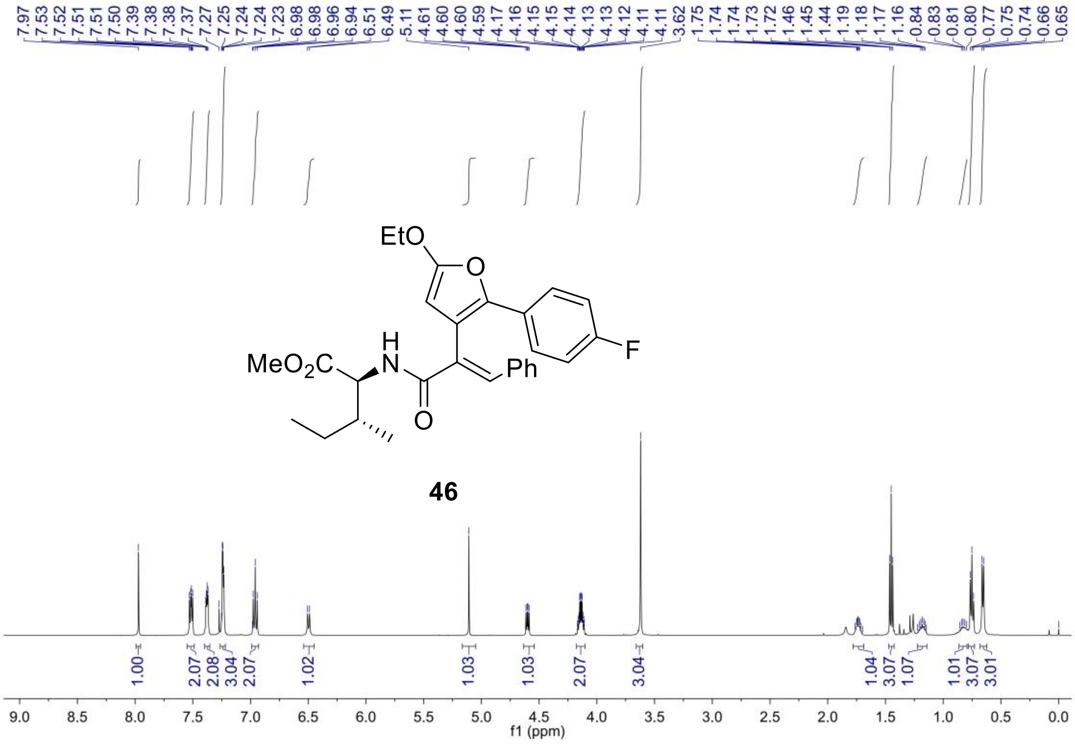

Supplementary Fig. 167 | ^{1}H NMR of compound 46 (500 MHz, at 25 ^{o}C, in $CDCl_3$).

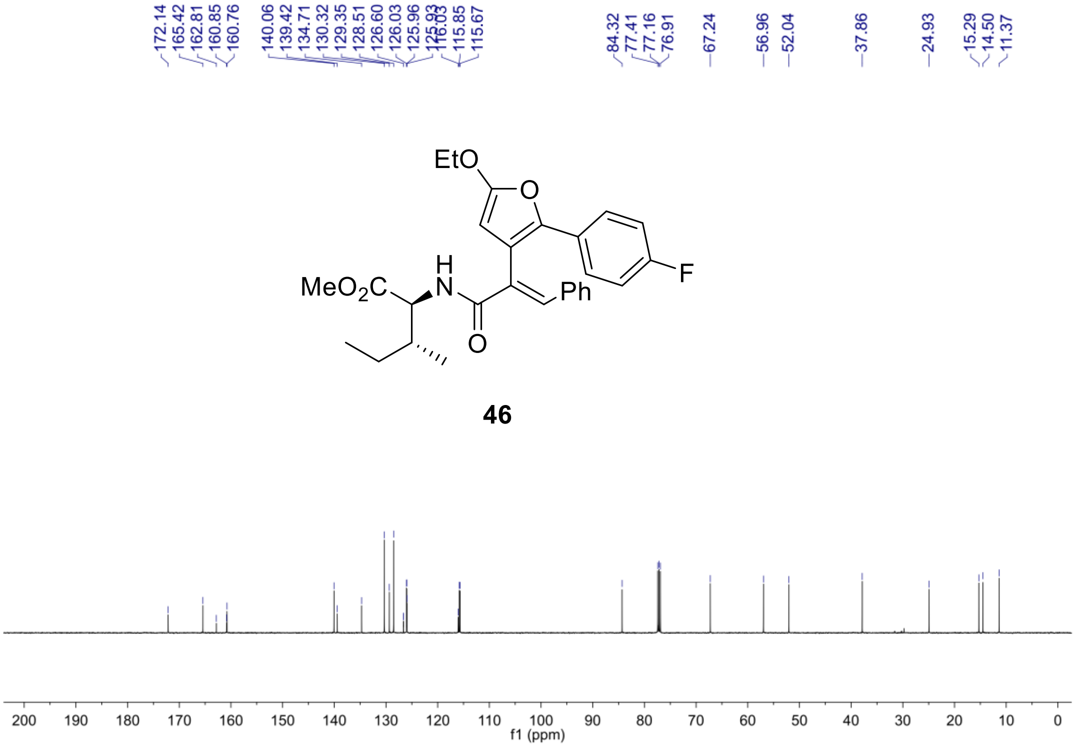

Supplementary Fig. 168 | ^{13}C NMR of compound 46 (125 MHz, at 25 ^{o}C, in $CDCl_3$).

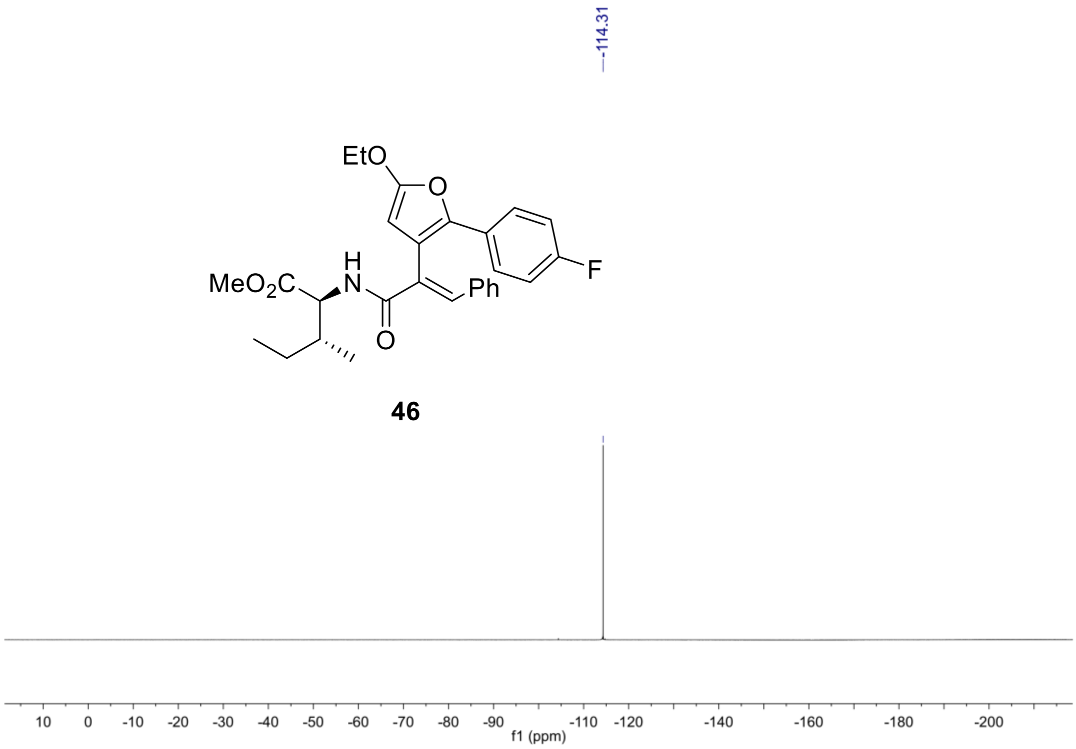

Supplementary Fig. 169 | ^{19}F NMR of compound 46 (376 MHz, at 25 °C, in $CDCl_3$).

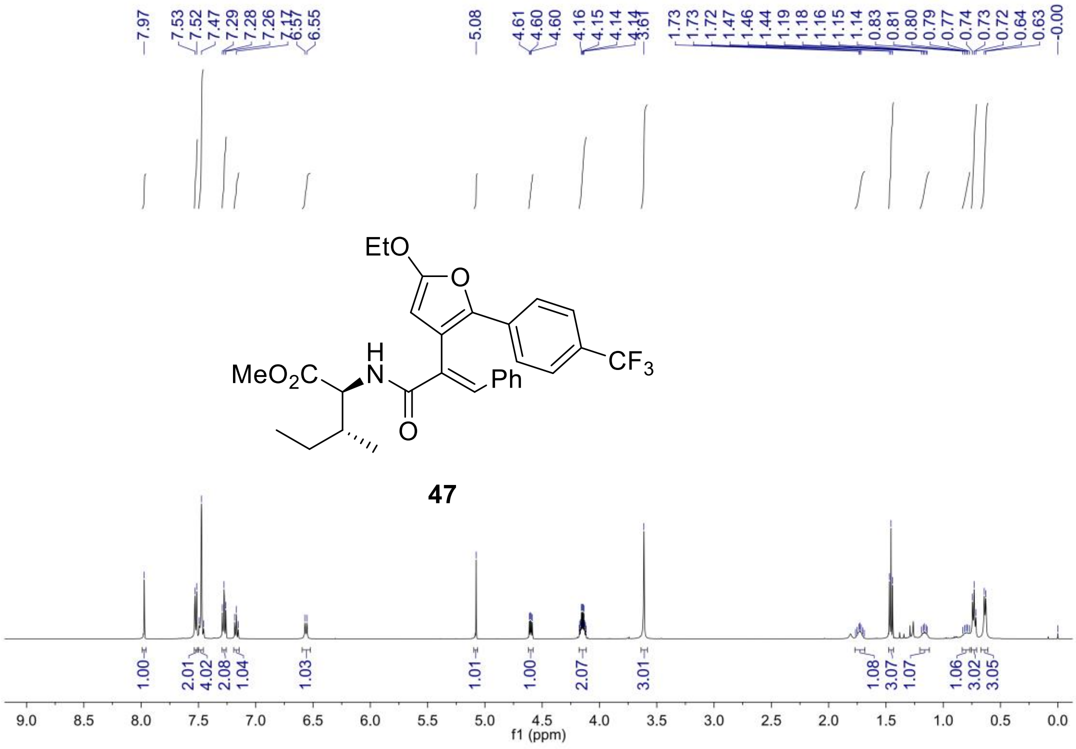

Supplementary Fig. 170 | ^{1}H NMR of compound 47 (500 MHz, at 25 °C, in $CDCl_3$).

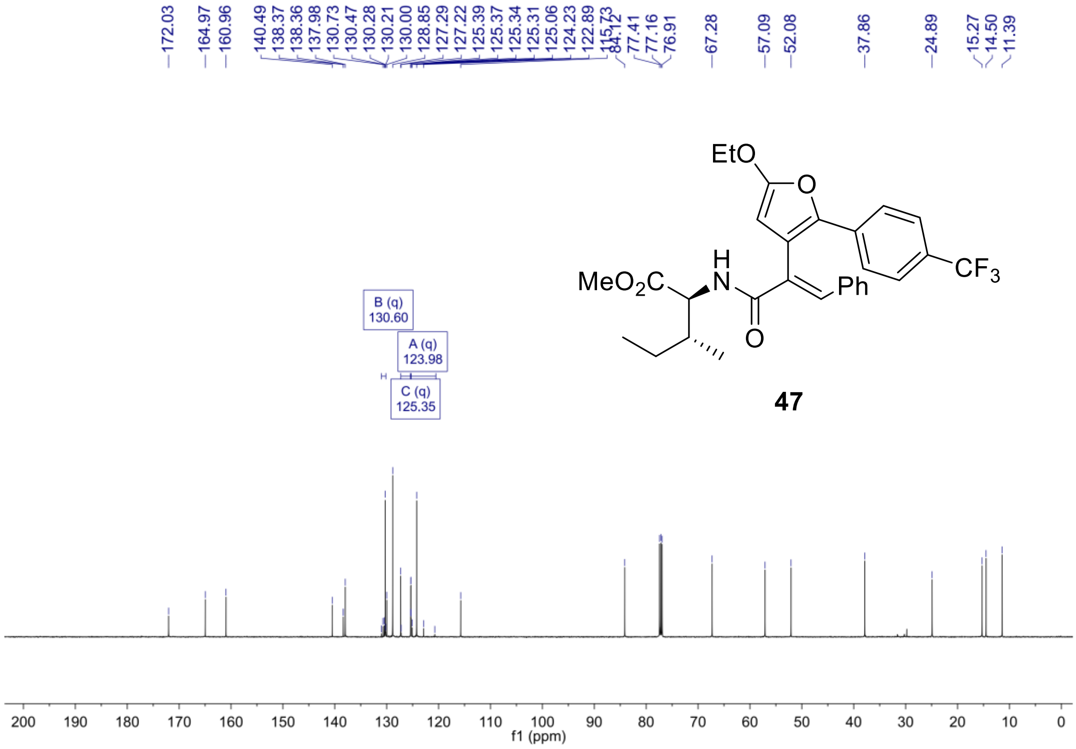

47

Supplementary Fig. 171 | ^{13}C NMR of compound 47 (125 MHz, at 25 °C, in $CDCl_3$).

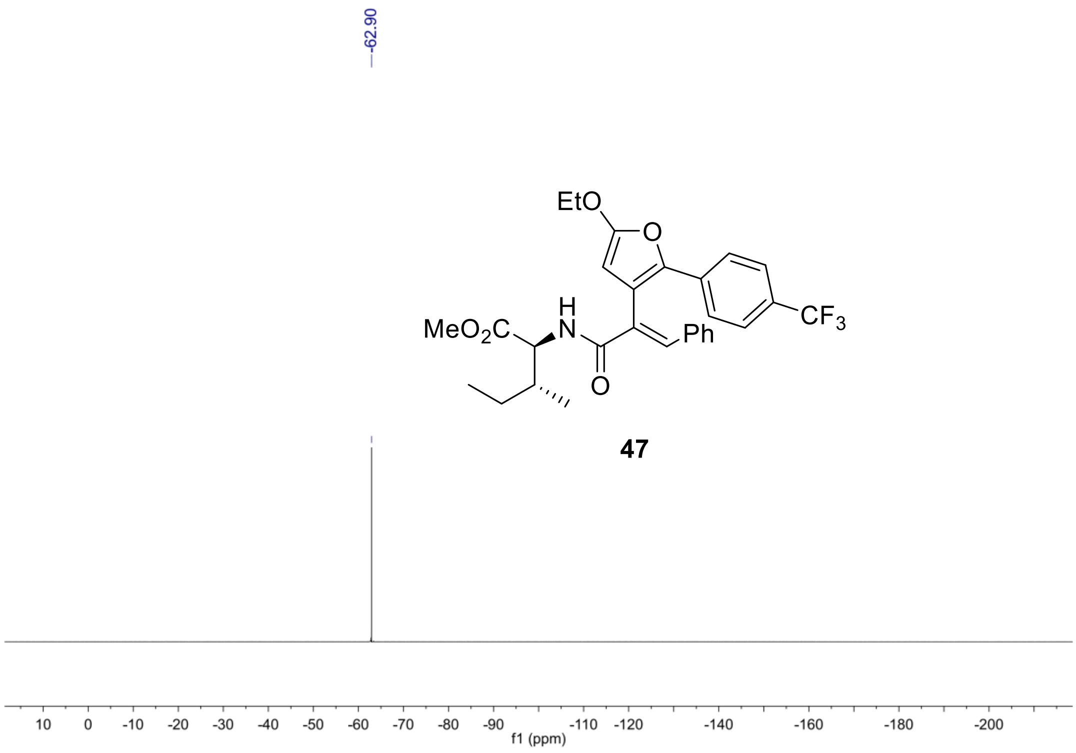

47

Supplementary Fig. 172 | ^{19}F NMR of compound 47 (376 MHz, at 25 °C, in $CDCl_3$).

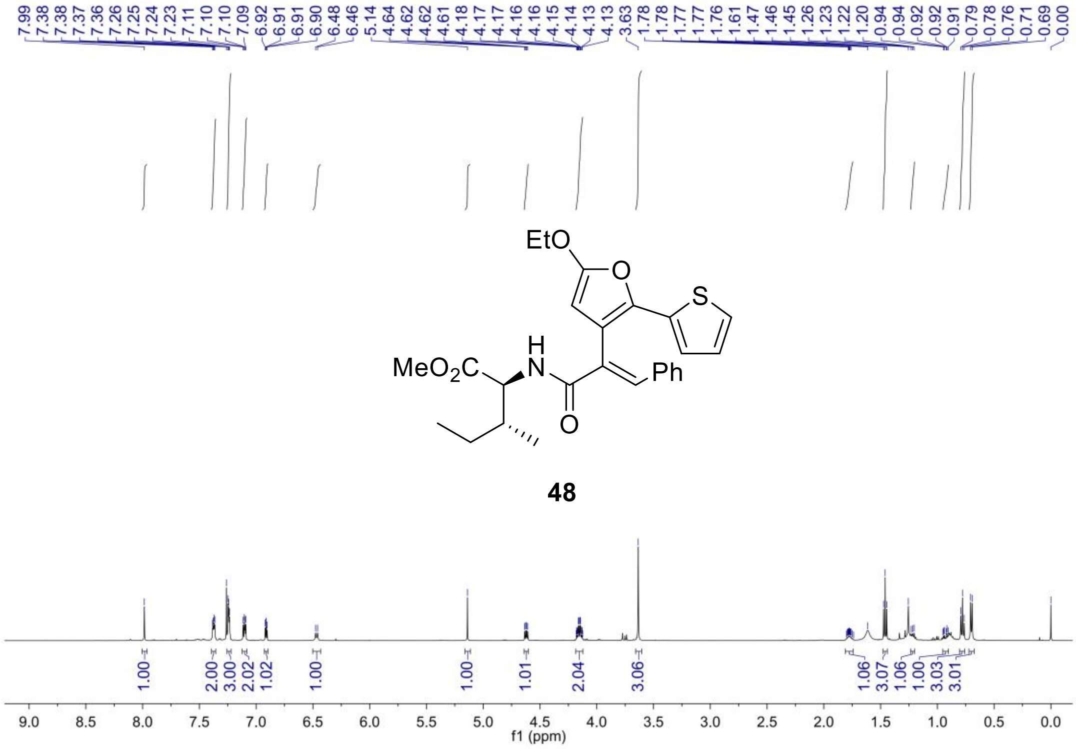

Supplementary Fig. 173 | ^{1}H NMR of compound 48 (500 MHz, at 25 °C, in $CDCl_3$).

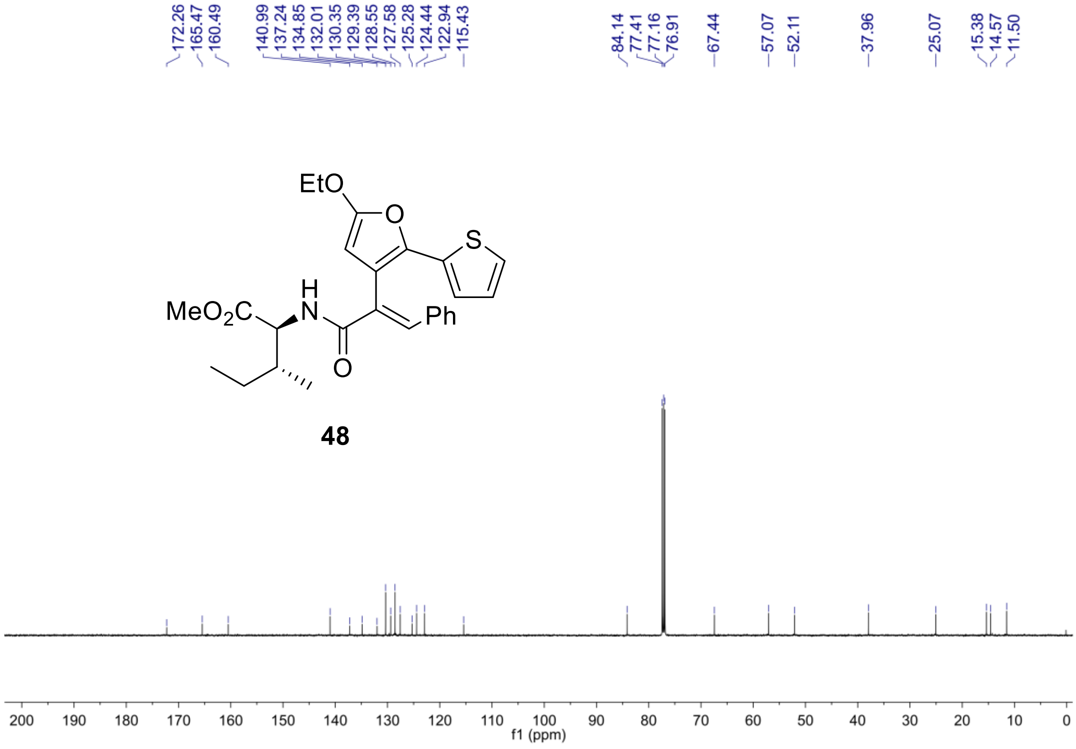

Supplementary Fig. 174 | ^{13}C NMR of compound 48 (125 MHz, at 25 °C, in $CDCl_3$).

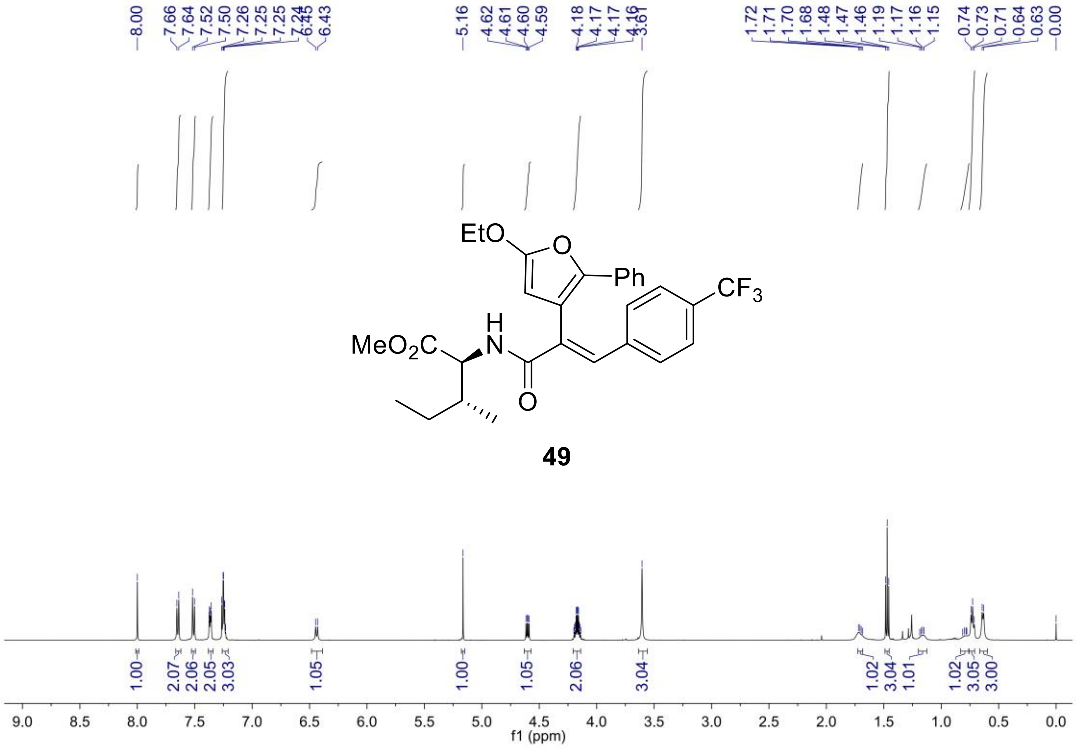

Supplementary Fig. 175 | ^{1}H NMR of compound 49 (500 MHz, at 25 °C, in $CDCl_3$).

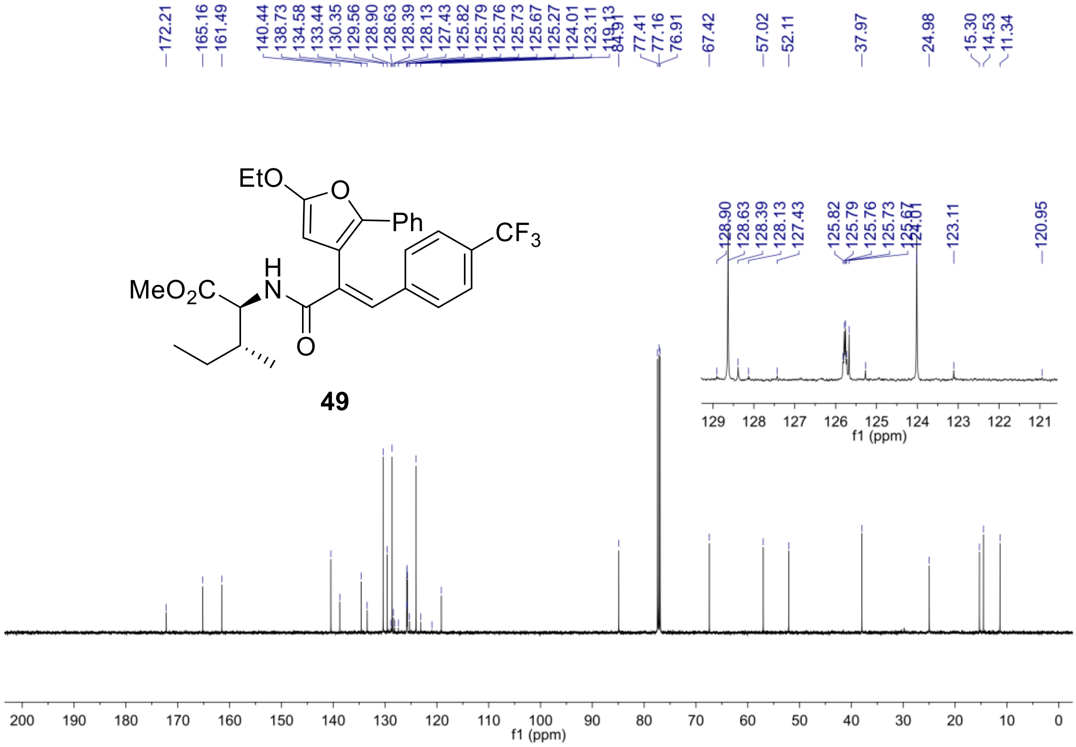

Supplementary Fig. 176 | ^{13}C NMR of compound 49 (125 MHz, at 25 °C, in $CDCl_3$).

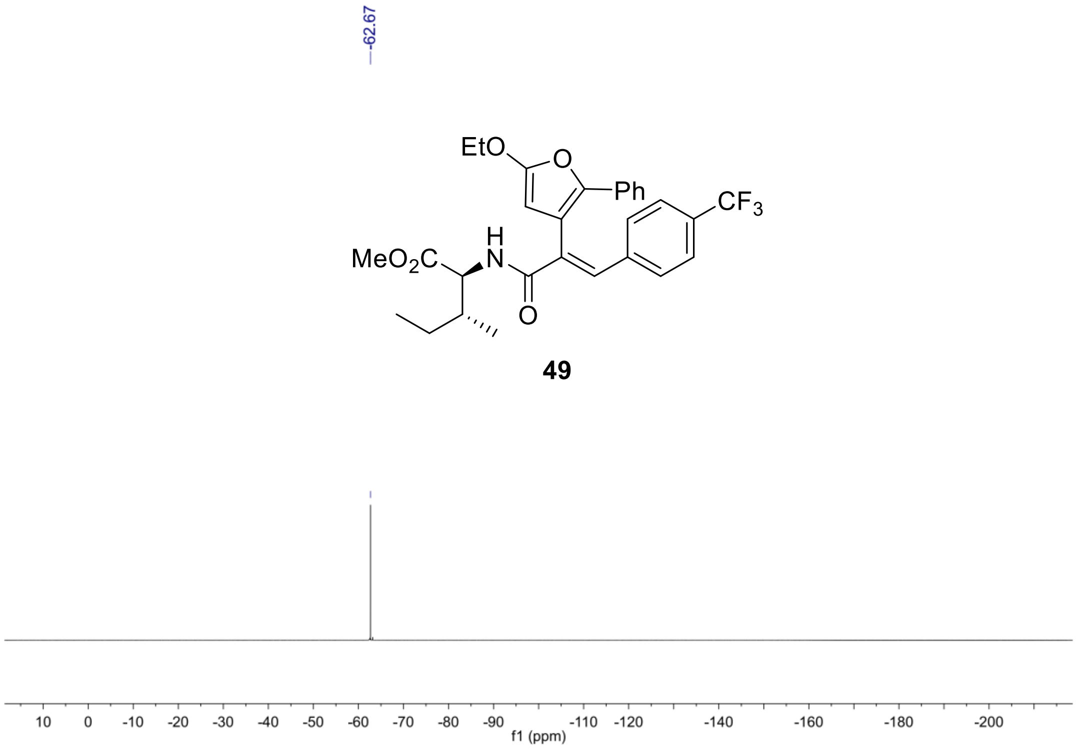

Supplementary Fig. 177 | ^{19}F NMR of compound 49 (376 MHz, at 25 ºC, in $CDCl_3$).

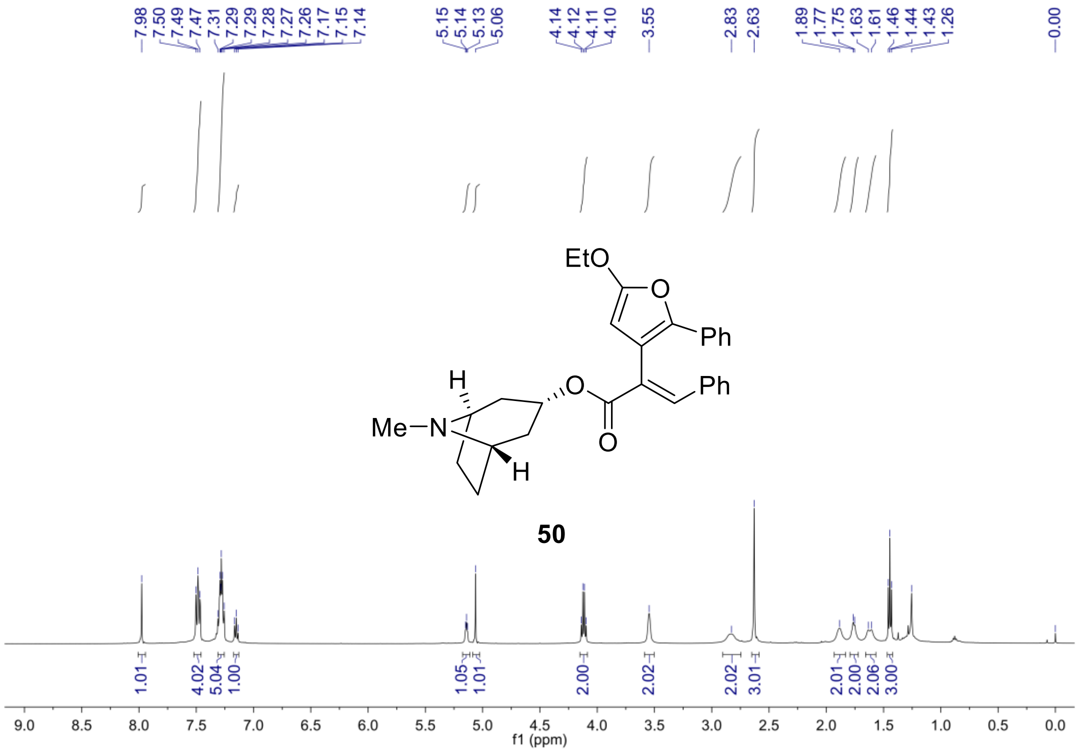

Supplementary Fig. 178 | ^{1}H NMR of compound 50 (500 MHz, at 25 ºC, in $CDCl_3$).

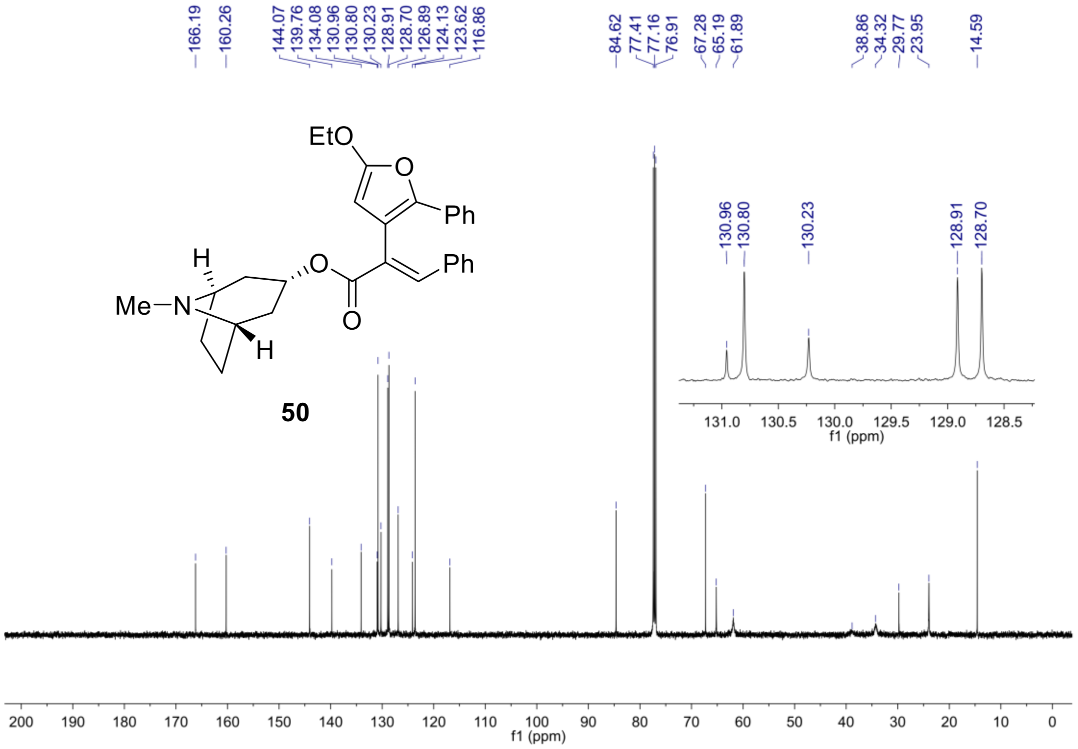

Supplementary Fig. 179 | ^{13}C NMR of compound 50 (125 MHz, at 25 °C, in $CDCl_3$).

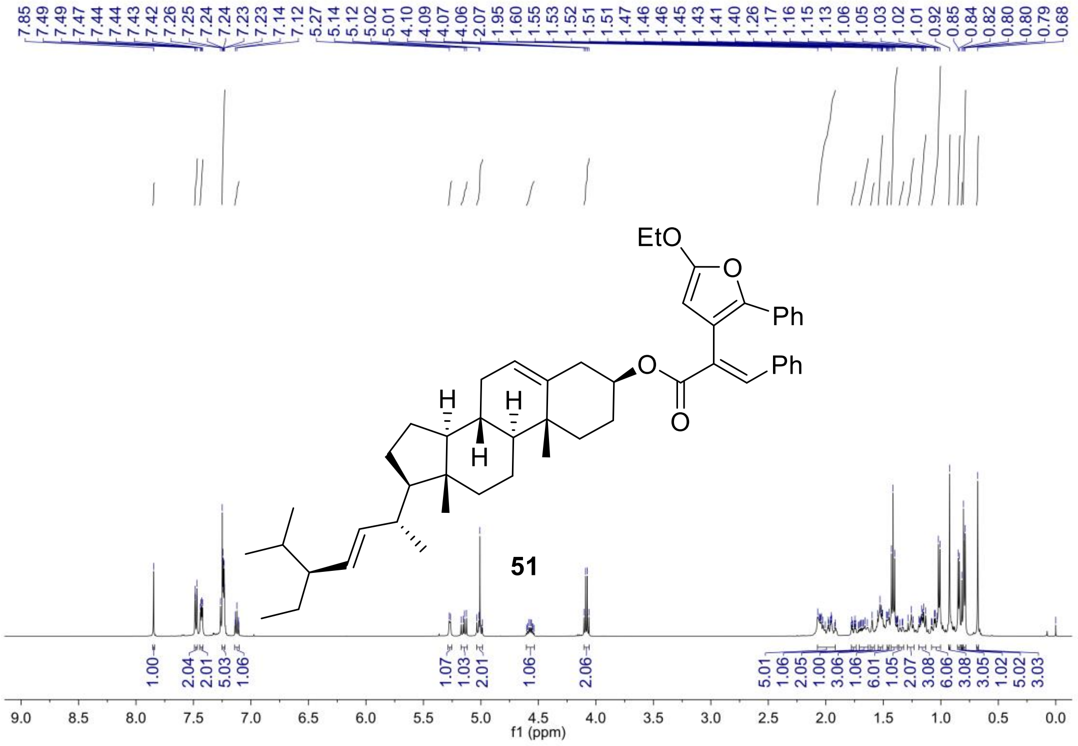

Supplementary Fig. 180 | ^{1}H NMR of compound 51 (500 MHz, at 25 °C, in $CDCl_3$).

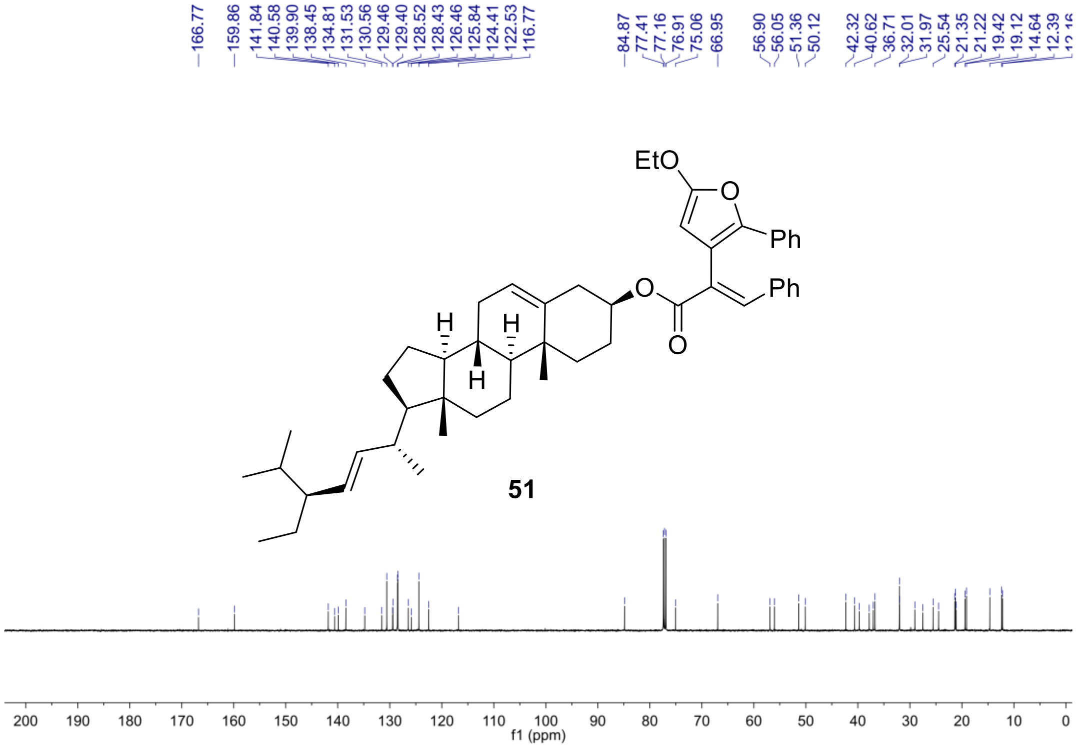

Supplementary Fig. 181 | ^{13}C NMR of compound 51 (125 MHz, at 25 °C, in $CDCl_3$).

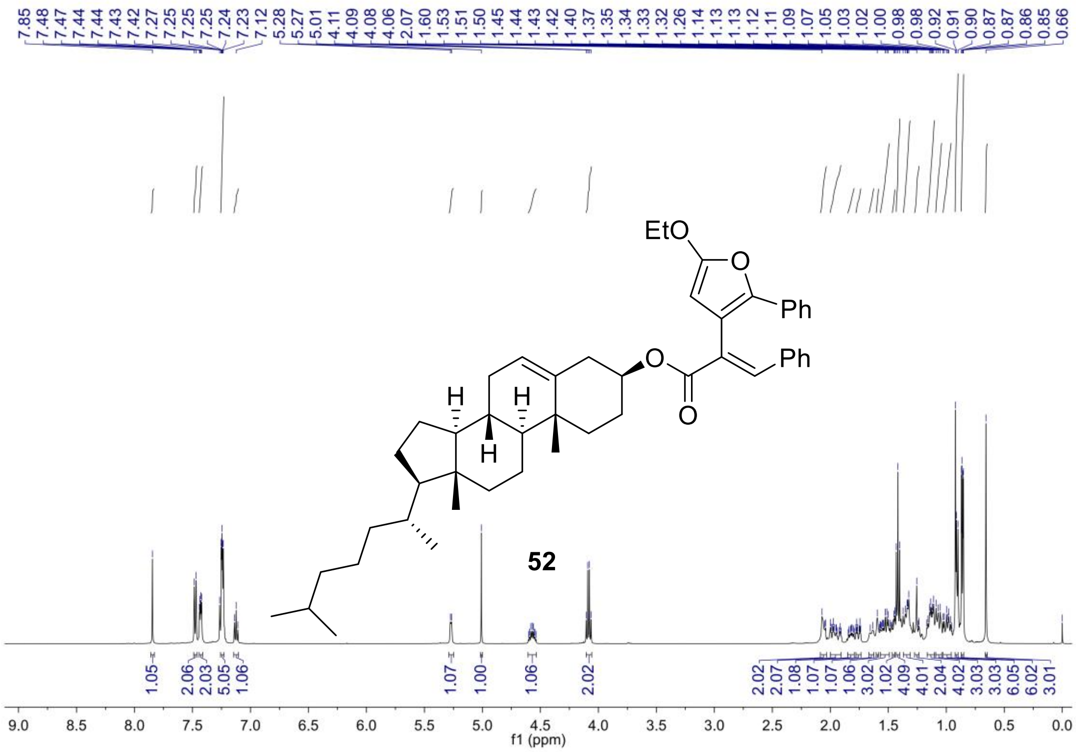

Supplementary Fig. 182 | ^{1}H NMR of compound 52 (500 MHz, at 25 °C, in $CDCl_3$).

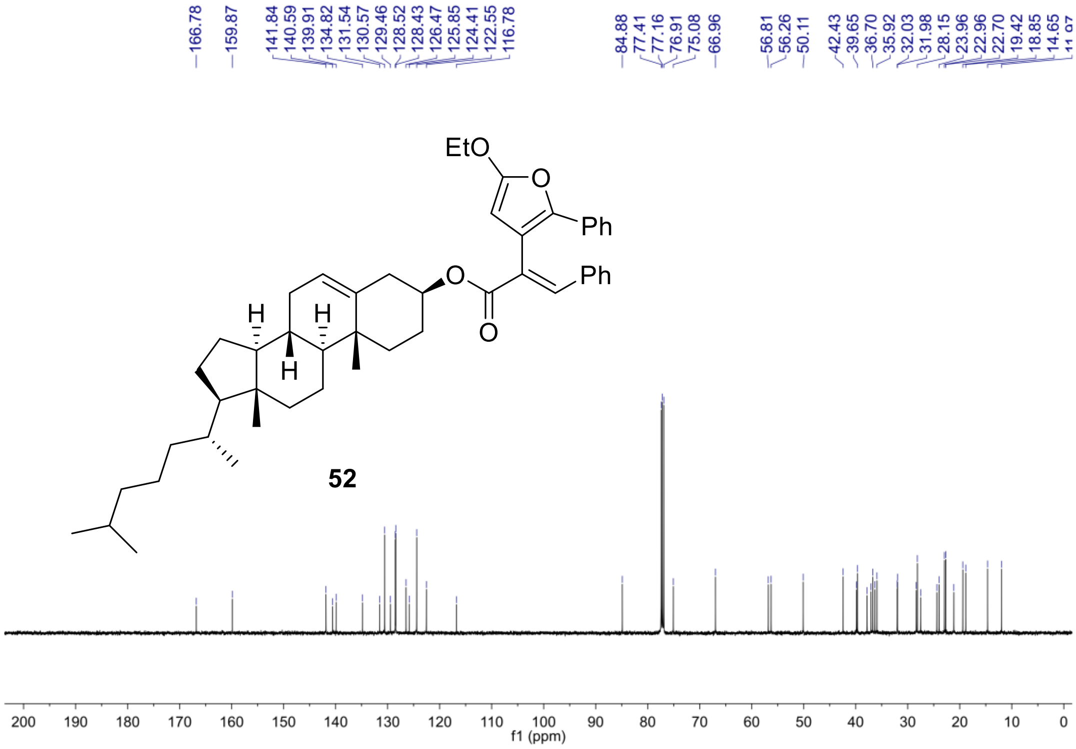

Supplementary Fig. 183 | ^{13}C NMR of compound 52 (125 MHz, at 25 °C, in $CDCl_3$).

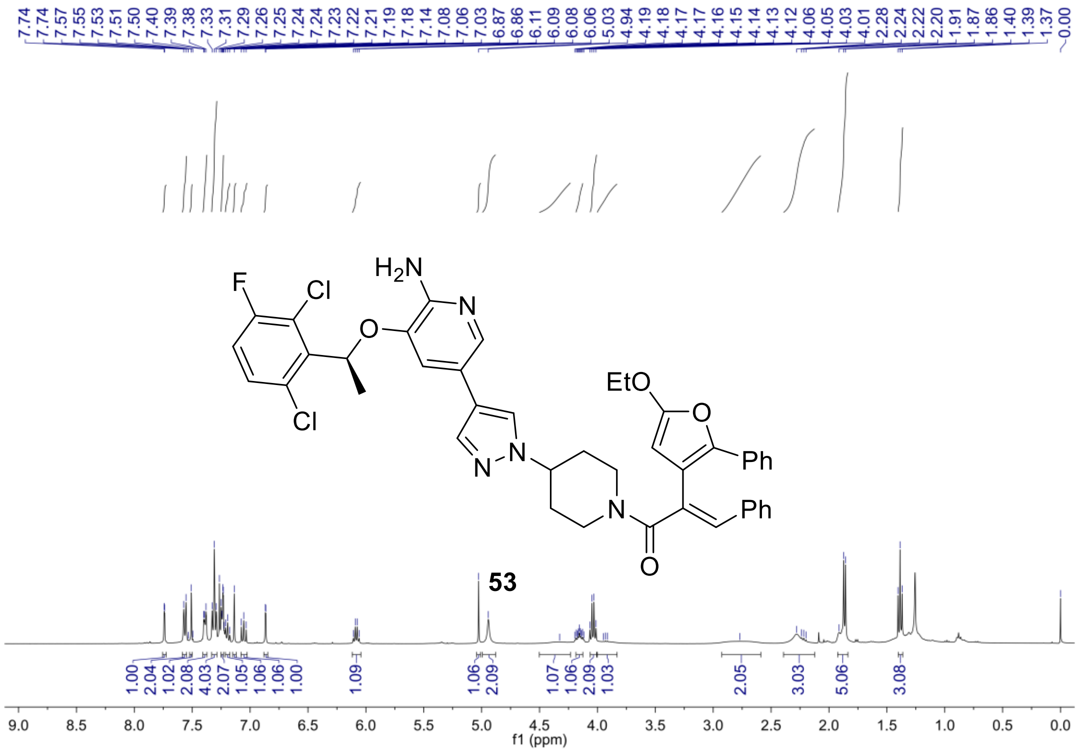

Supplementary Fig. 184 | ^{1}H NMR of compound 53 (400 MHz, at 25 °C, in $CDCl_3$).

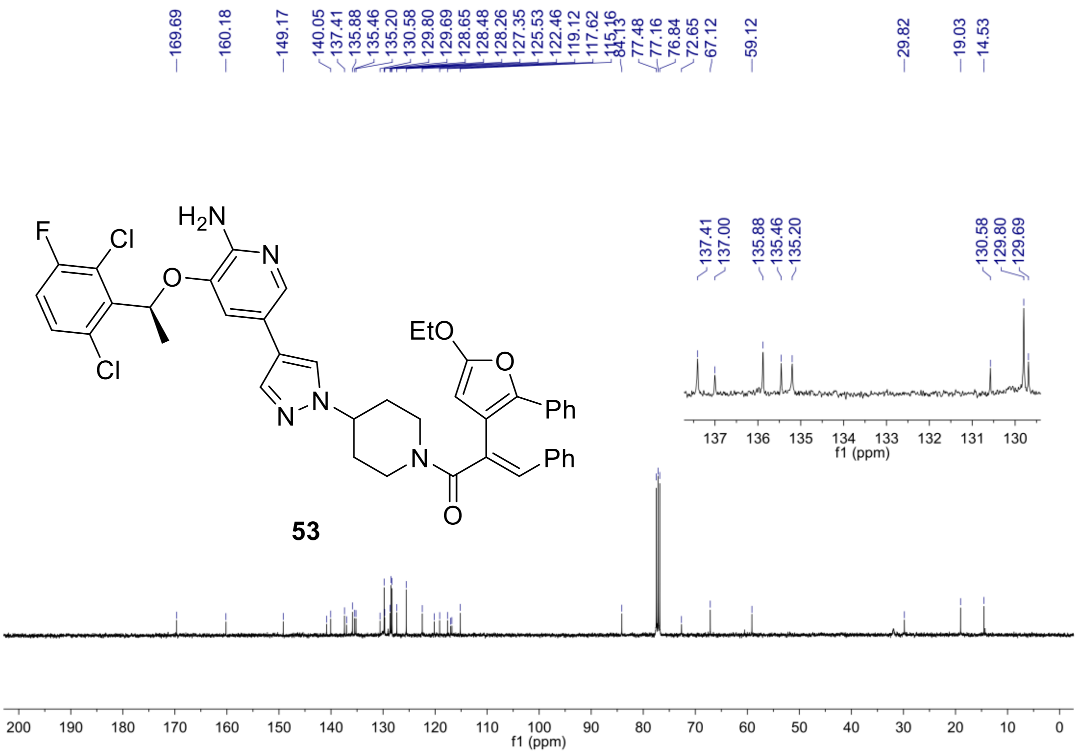

Supplementary Fig. 185 | ^{13}C NMR of compound 53 (100 MHz, at 25 ºC, in $CDCl_3$).

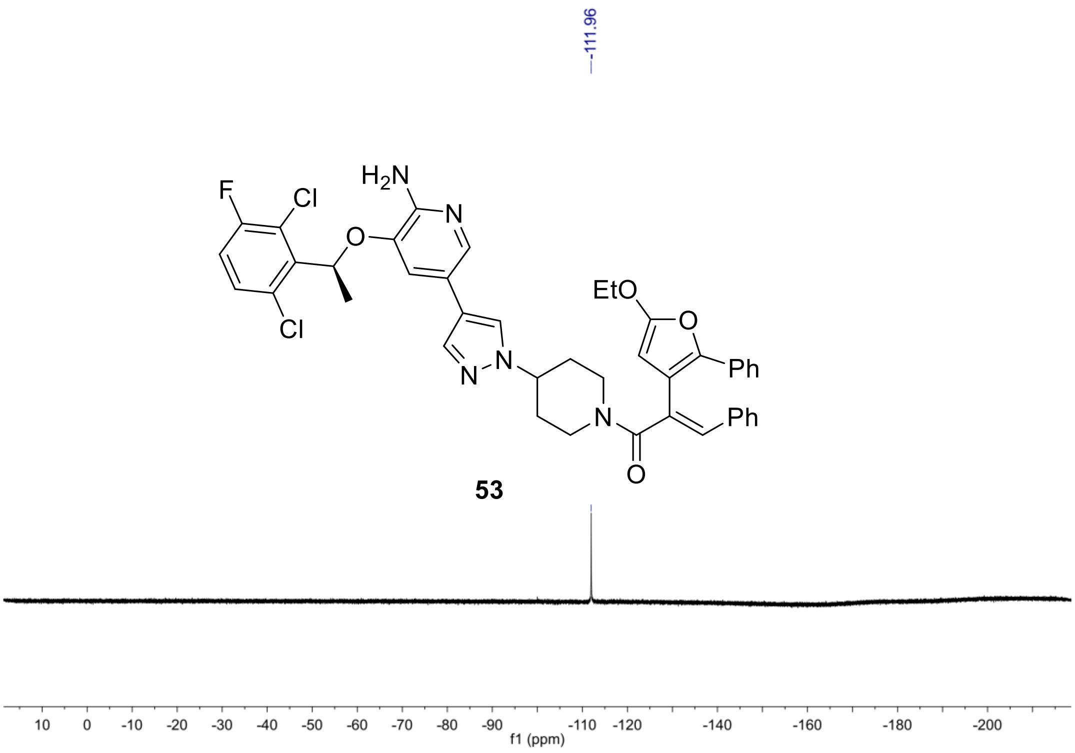

Supplementary Fig. 186 | ^{19}F NMR of compound 53 (376 MHz, at 25 ºC, in $CDCl_3$).

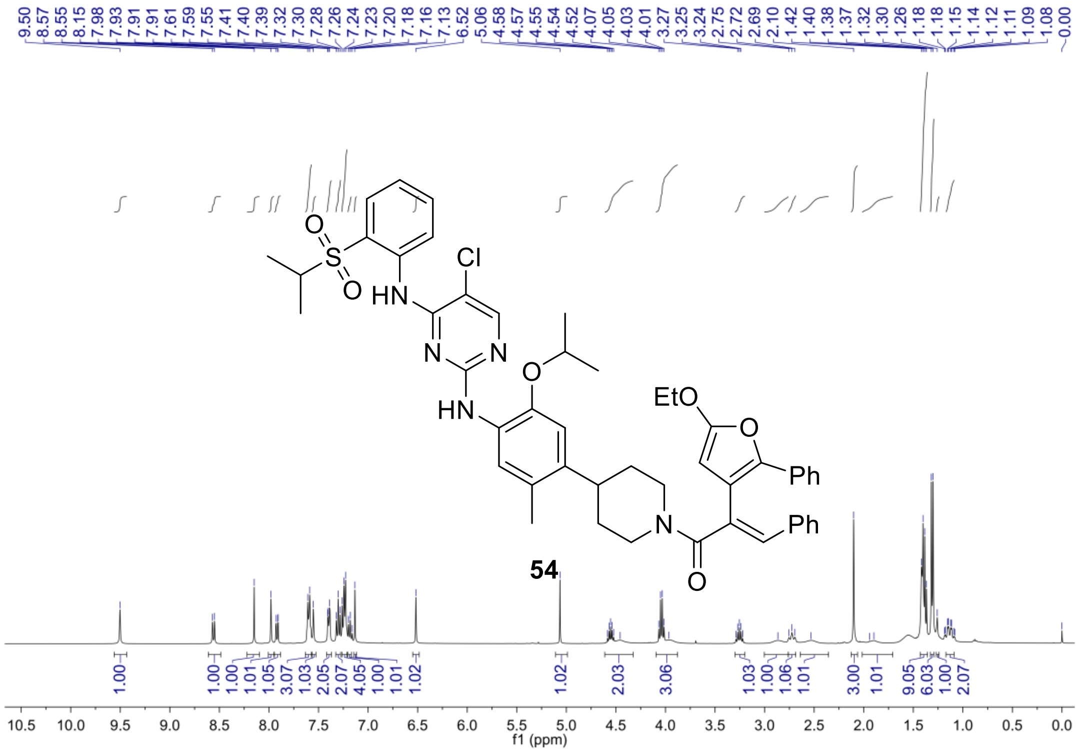

Supplementary Fig. 187 | ^{1}H NMR of compound 54 (400 MHz, at 25 ºC, in $CDCl_3$).

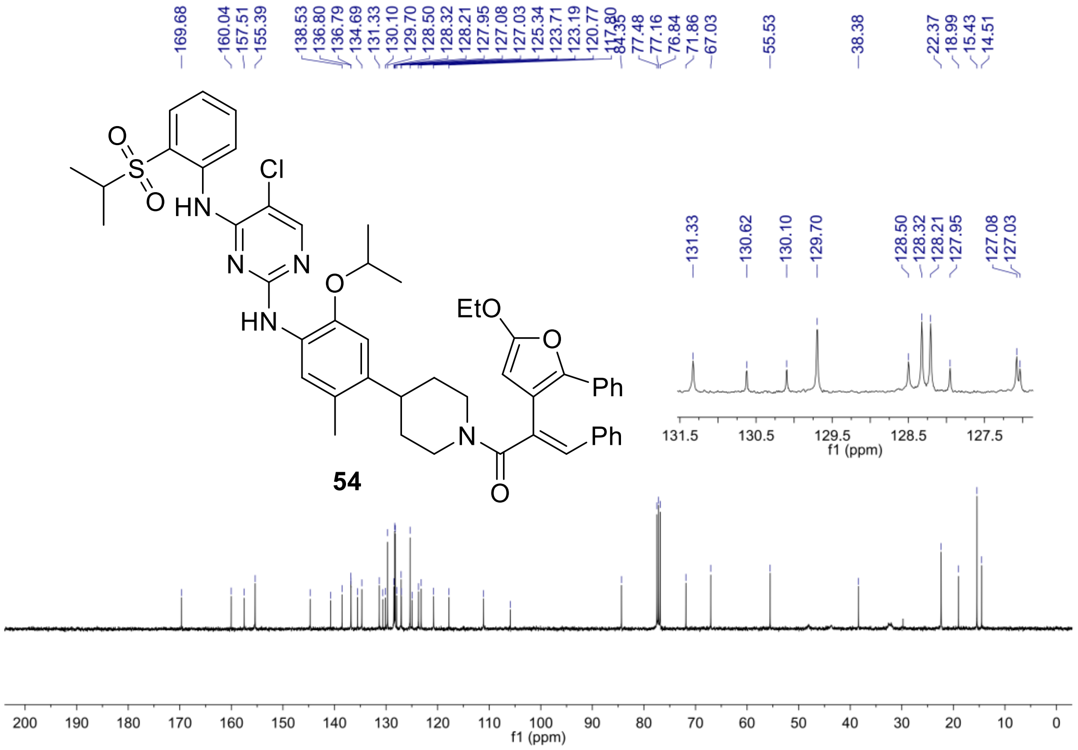

Supplementary Fig. 188 | ^{13}C NMR of compound 54 (100 MHz, at 25 ºC, in $CDCl_3$).

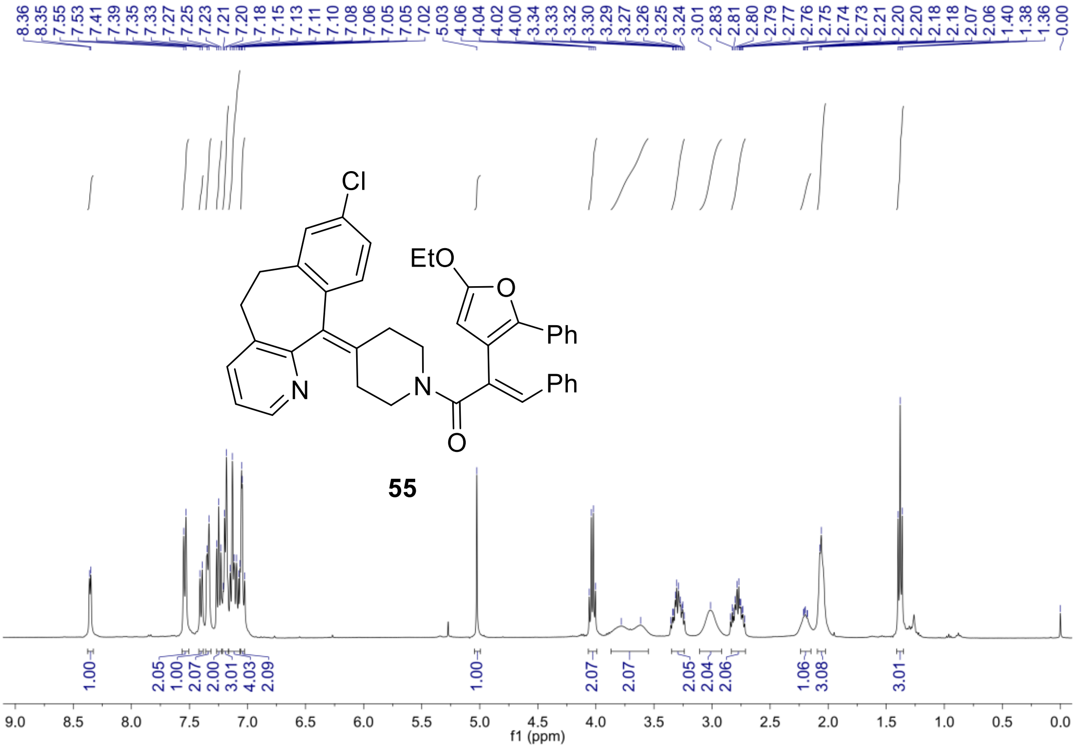

Supplementary Fig. 189 | ^{1}H NMR of compound 55 (400 MHz, at 25 °C, in $CDCl_3$).

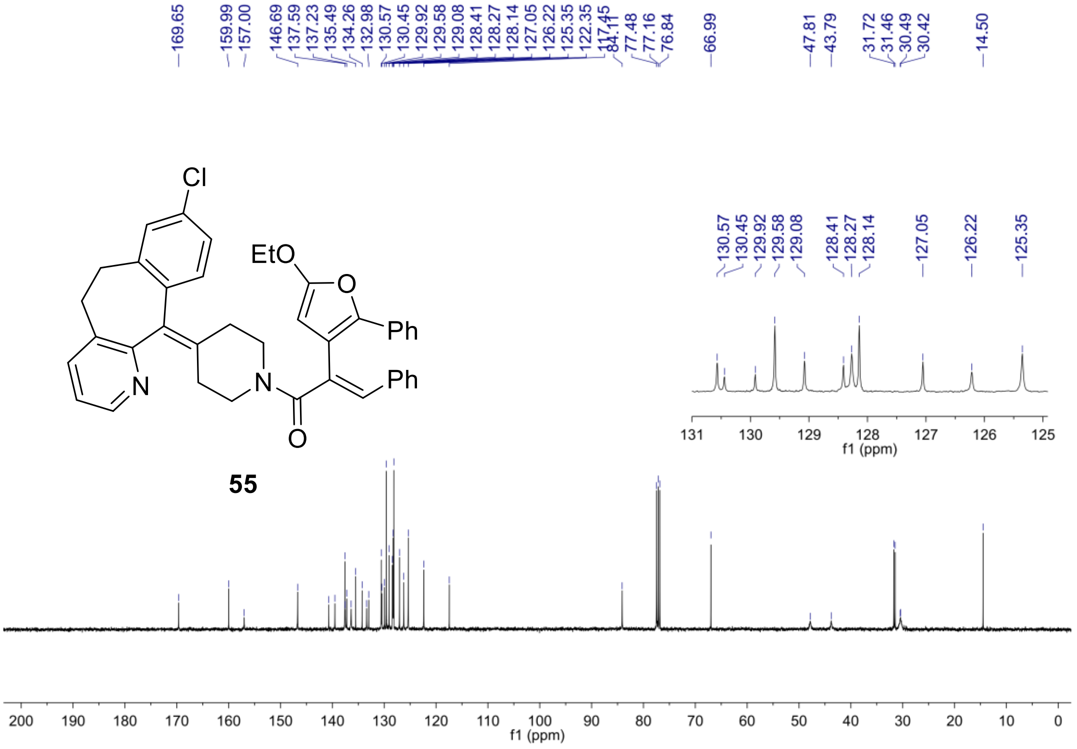

Supplementary Fig. 190 | ^{13}C NMR of compound 55 (100 MHz, at 25 °C, in $CDCl_3$).

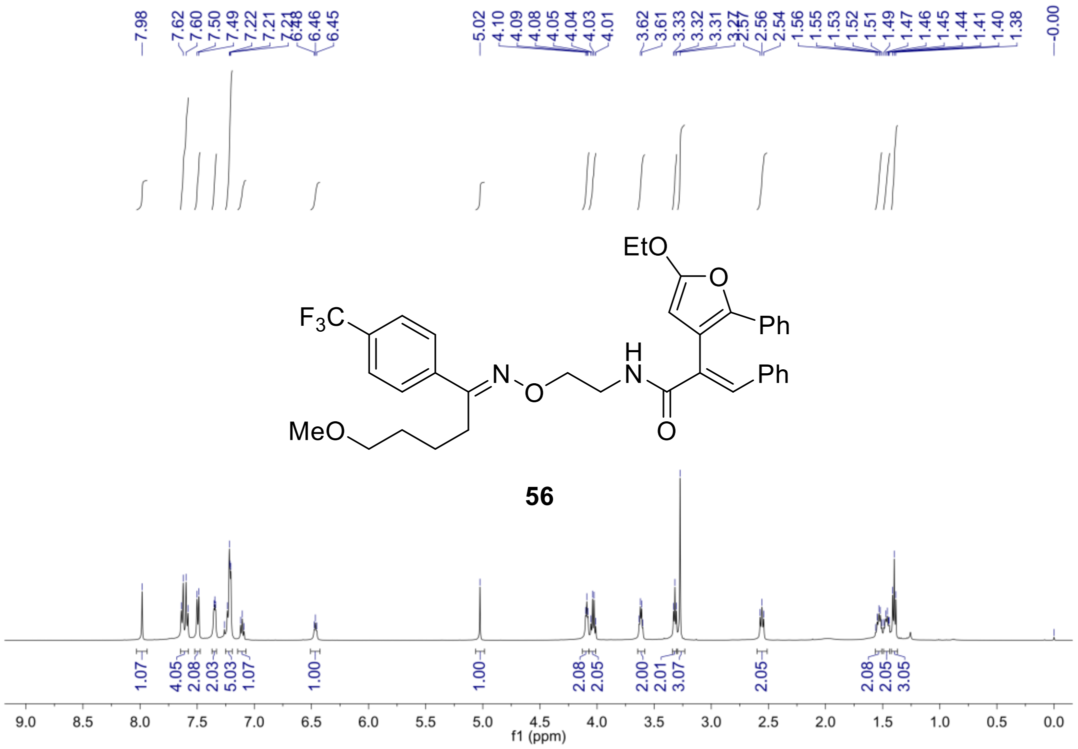

Supplementary Fig. 191 | ^{1}H NMR of compound 56 (500 MHz, at 25 ^{o}C, in $CDCl_3$).

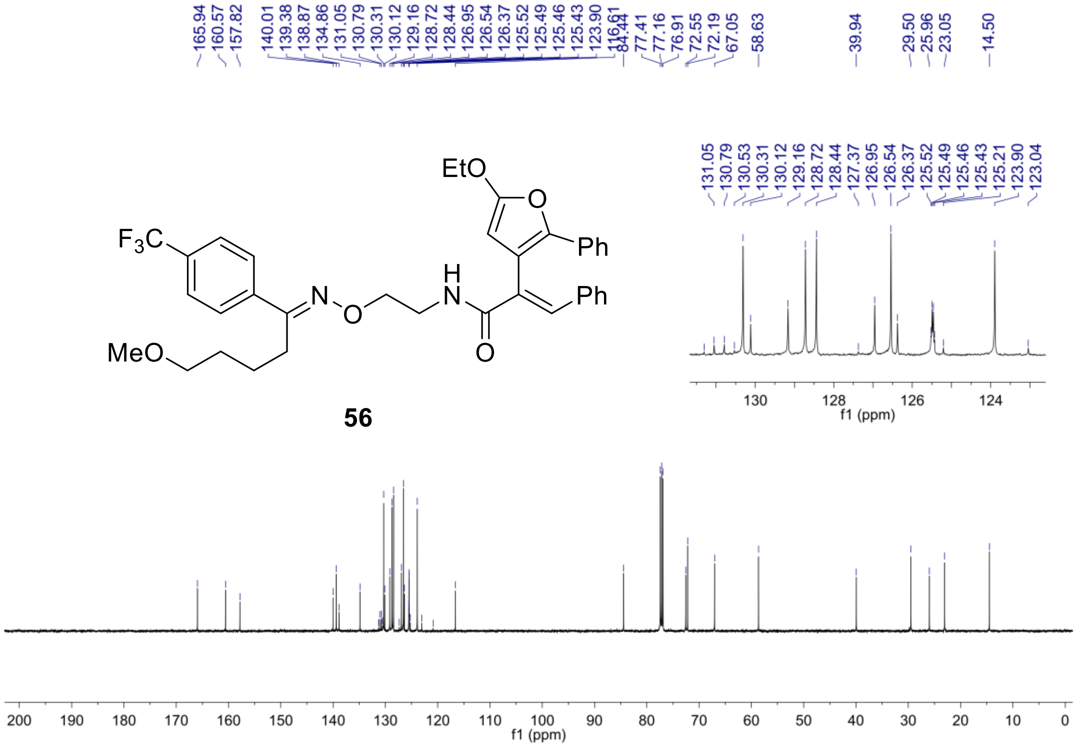

Supplementary Fig. 192 | ^{13}C NMR of compound 56 (125 MHz, at 25 ^{o}C, in $CDCl_3$).

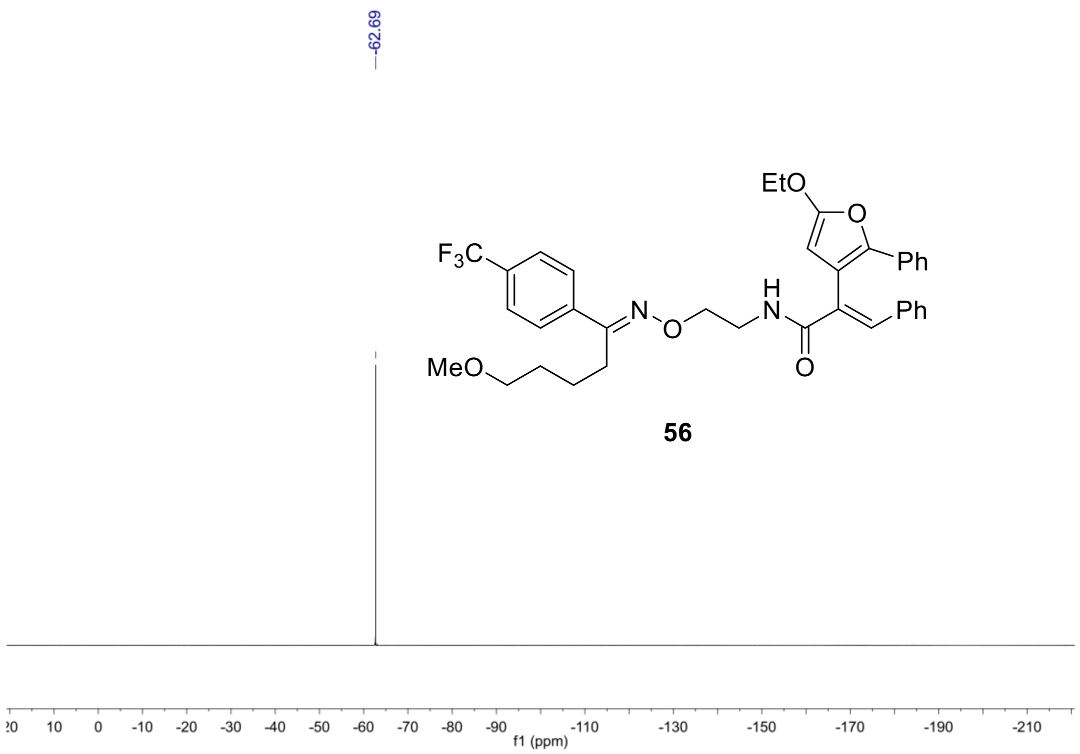

Supplementary Fig. 193 | ^{19}F NMR of compound 56 (470 MHz, at 25 ºC, in $CDCl_3$).

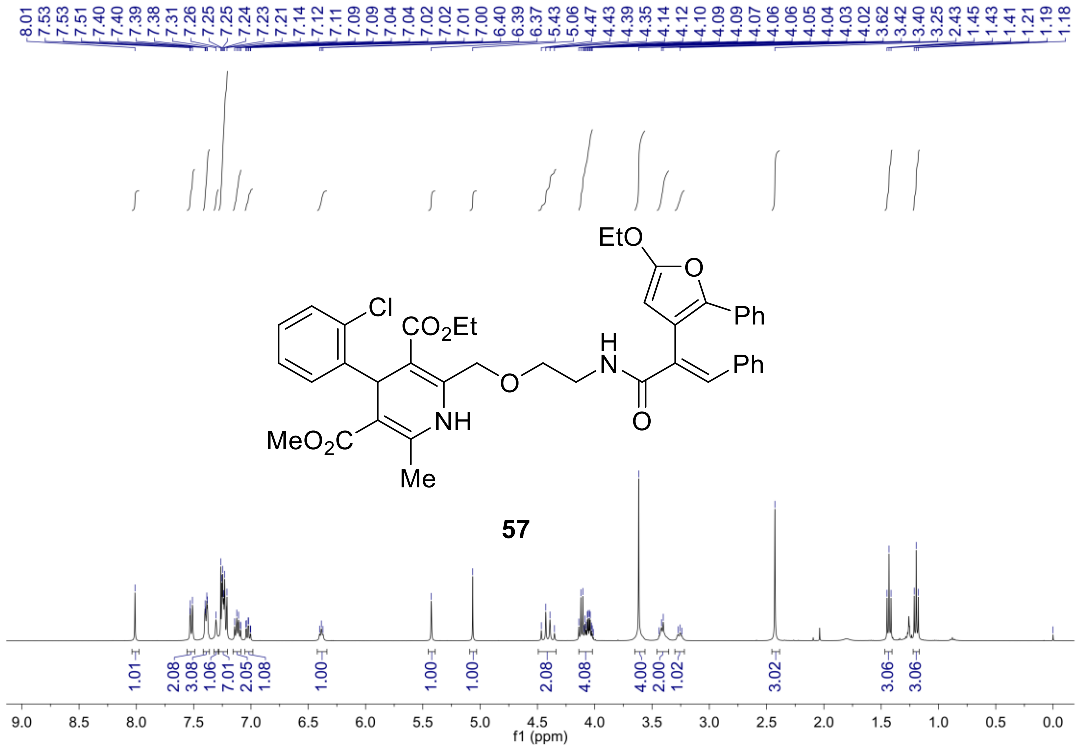

Supplementary Fig. 194 | ^{1}H NMR of compound 57 (400 MHz, at 25 ºC, in $CDCl_3$).

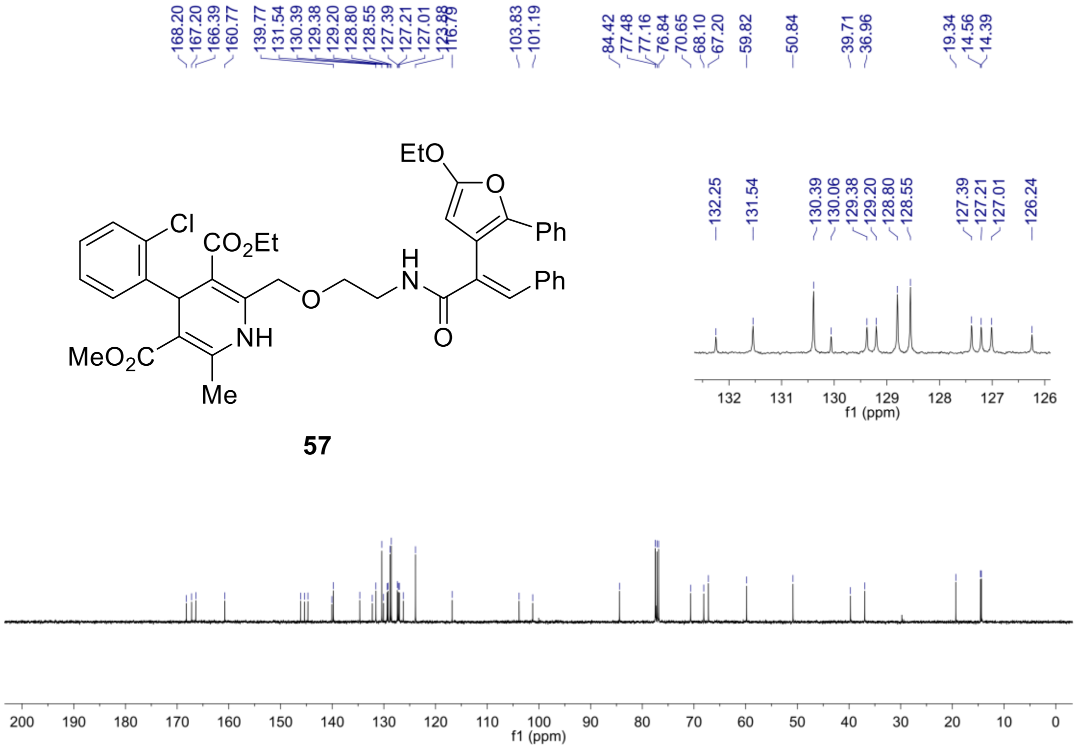

Supplementary Fig. 195 | ^{13}C NMR of compound 57 (100 MHz, at 25 ºC, in $CDCl_3$).

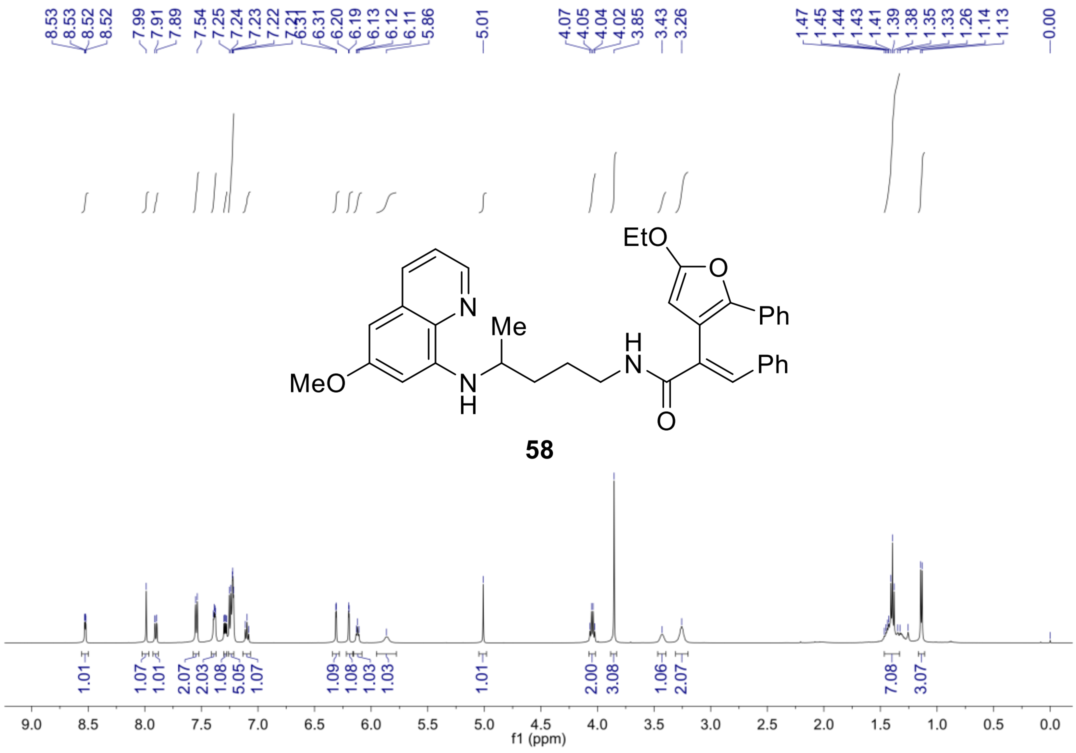

Supplementary Fig. 196 | ^{1}H NMR of compound 58 (500 MHz, at 25 ºC, in $CDCl_3$).

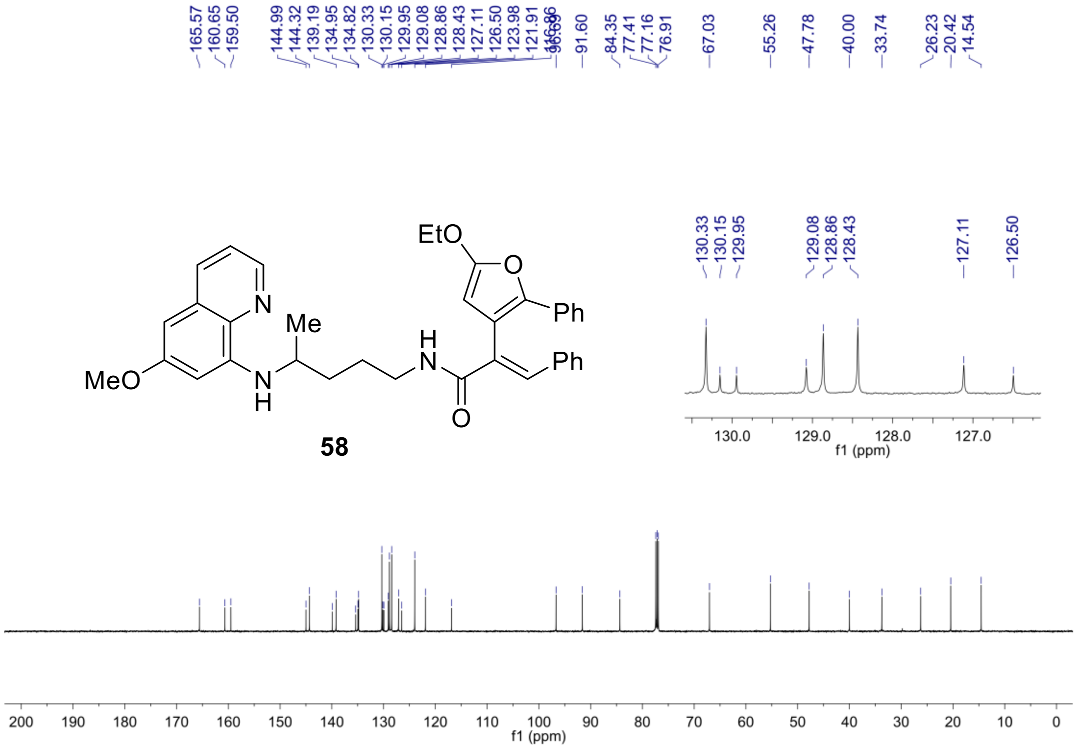

Supplementary Fig. 197 | ^{13}C NMR of compound 58 (125 MHz, at 25 °C, in $CDCl_3$).

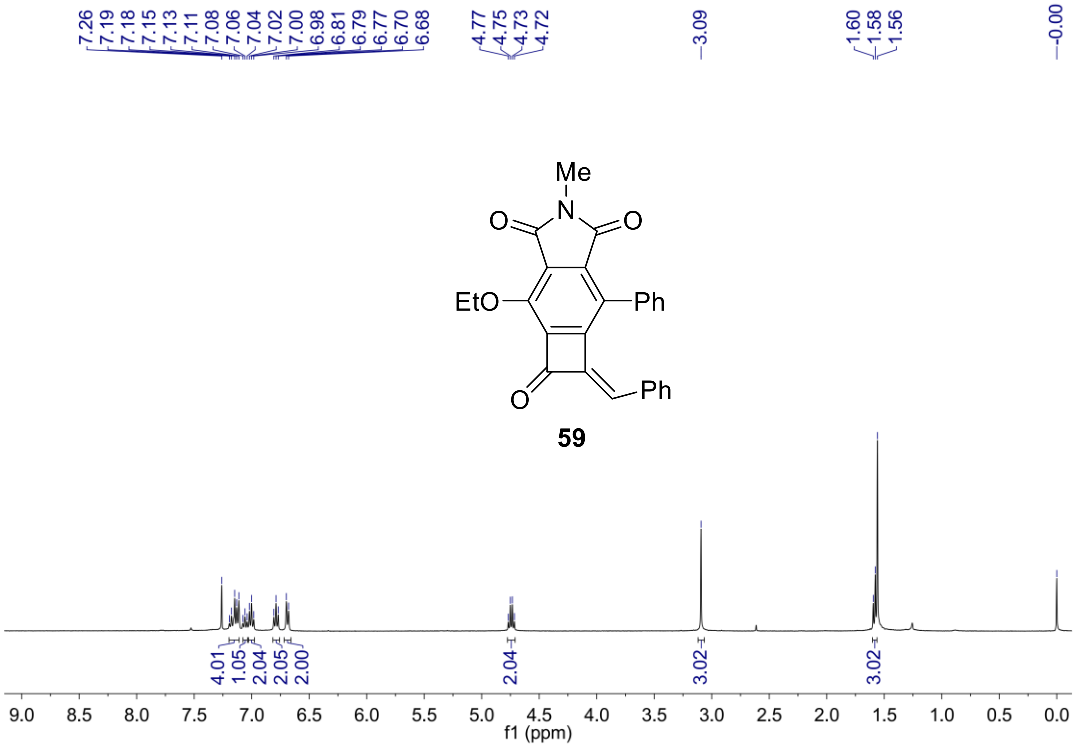

Supplementary Fig. 198 | ^{1}H NMR of compound 59 (400 MHz, at 25 °C, in $CDCl_3$).

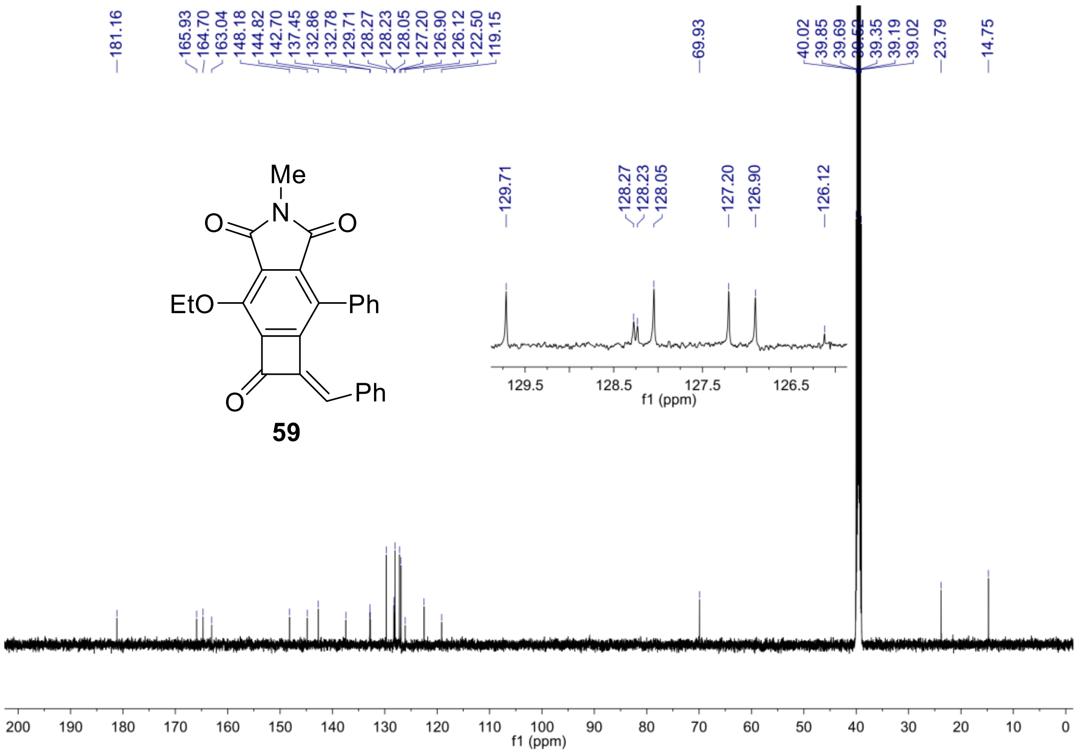

Supplementary Fig. 199 | ^{13}C NMR of compound 59 (125 MHz, at 25 °C, in DMSO).

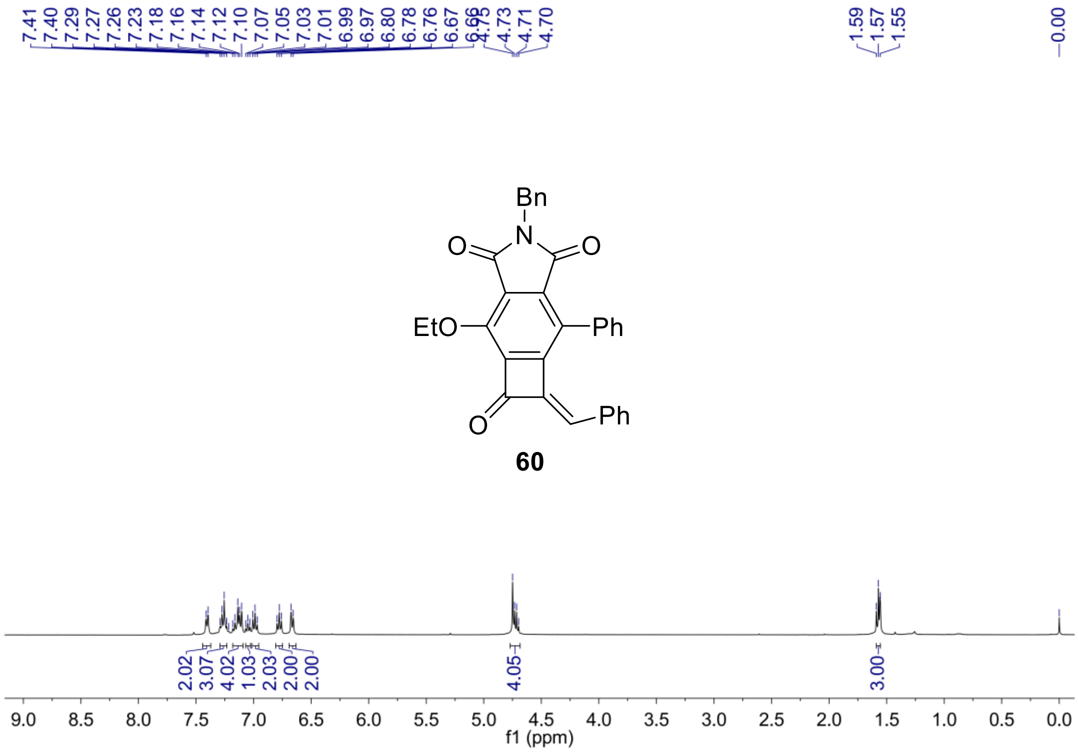

Supplementary Fig. 200 | ^{1}H NMR of compound 60 (400 MHz, at 25 °C, in $CDCl_3$).

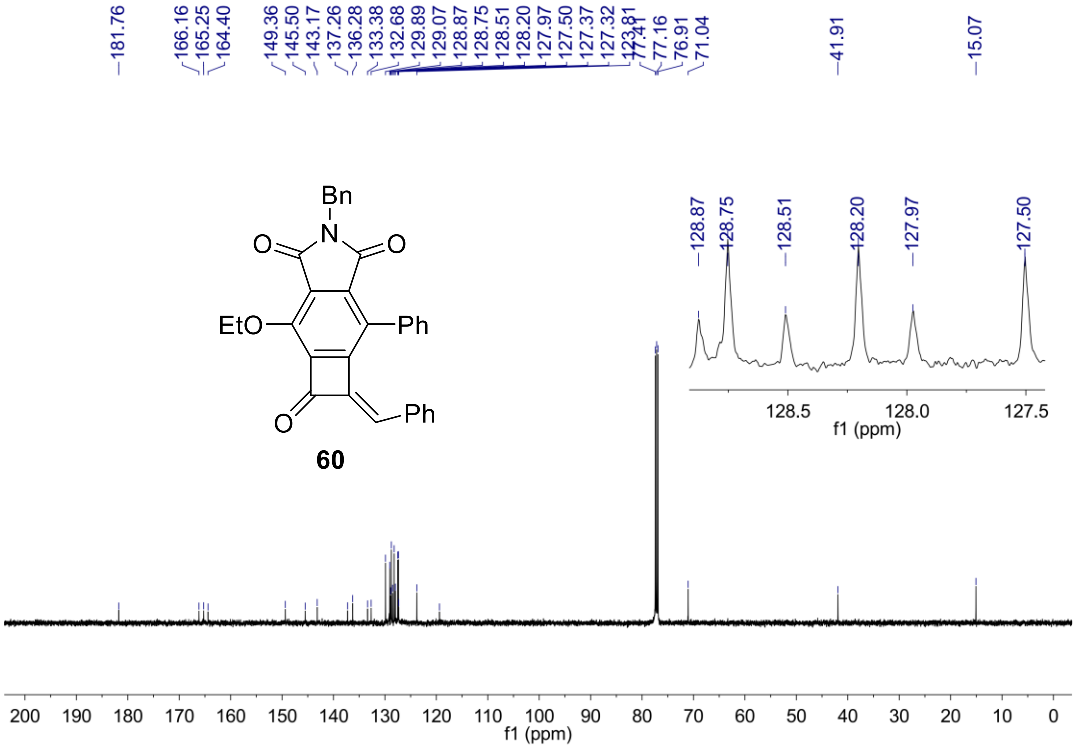

Supplementary Fig. 201 | ^{13}C NMR of compound 60 (100 MHz, at 25 °C, in $CDCl_3$).

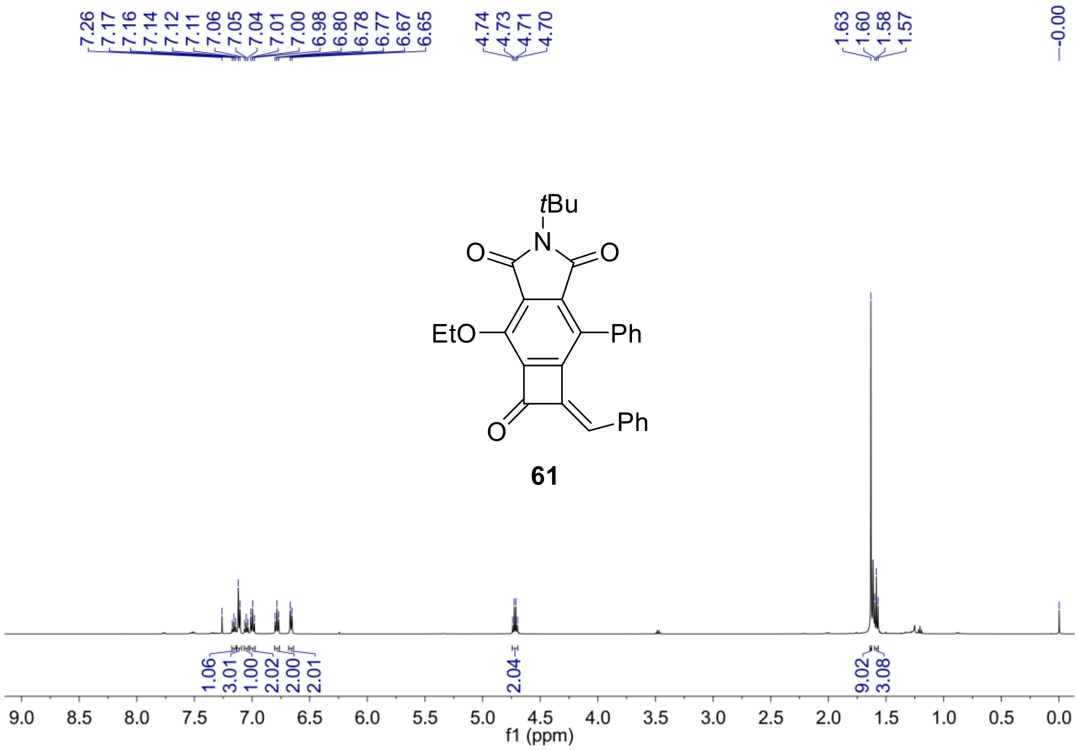

Supplementary Fig. 202 | ^{1}H NMR of compound 61 (500 MHz, at 25 °C, in $CDCl_3$).

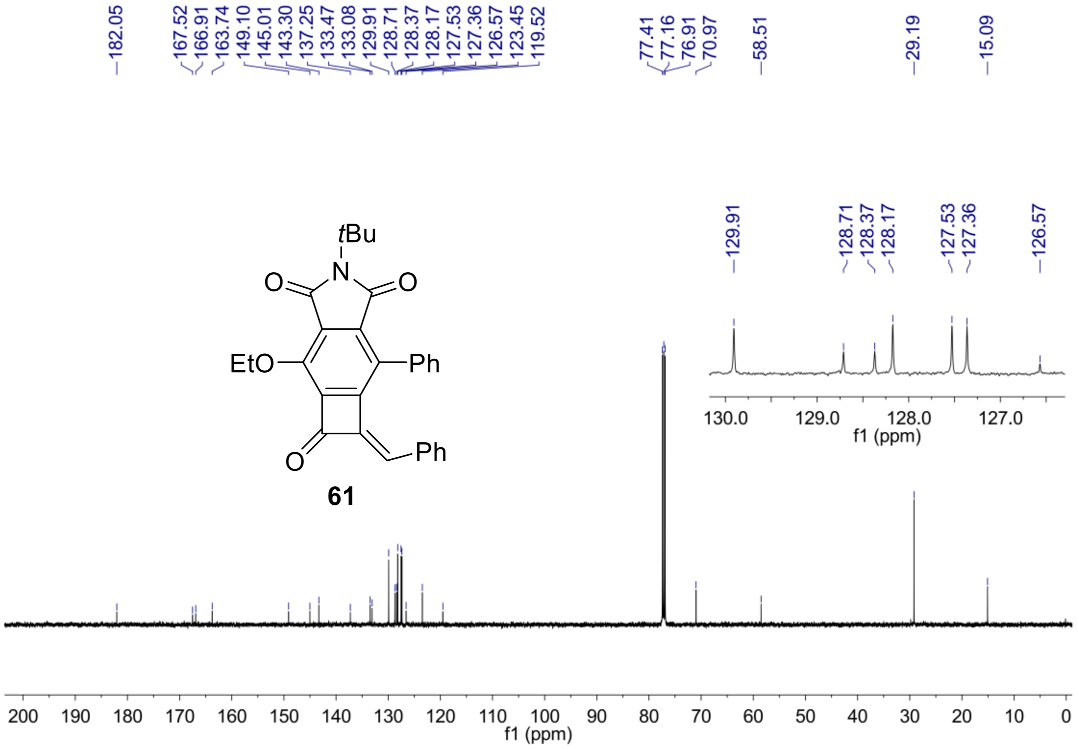

Supplementary Fig. 203 | ^{13}C NMR of compound 61 (125 MHz, at 25 °C, in $CDCl_3$).

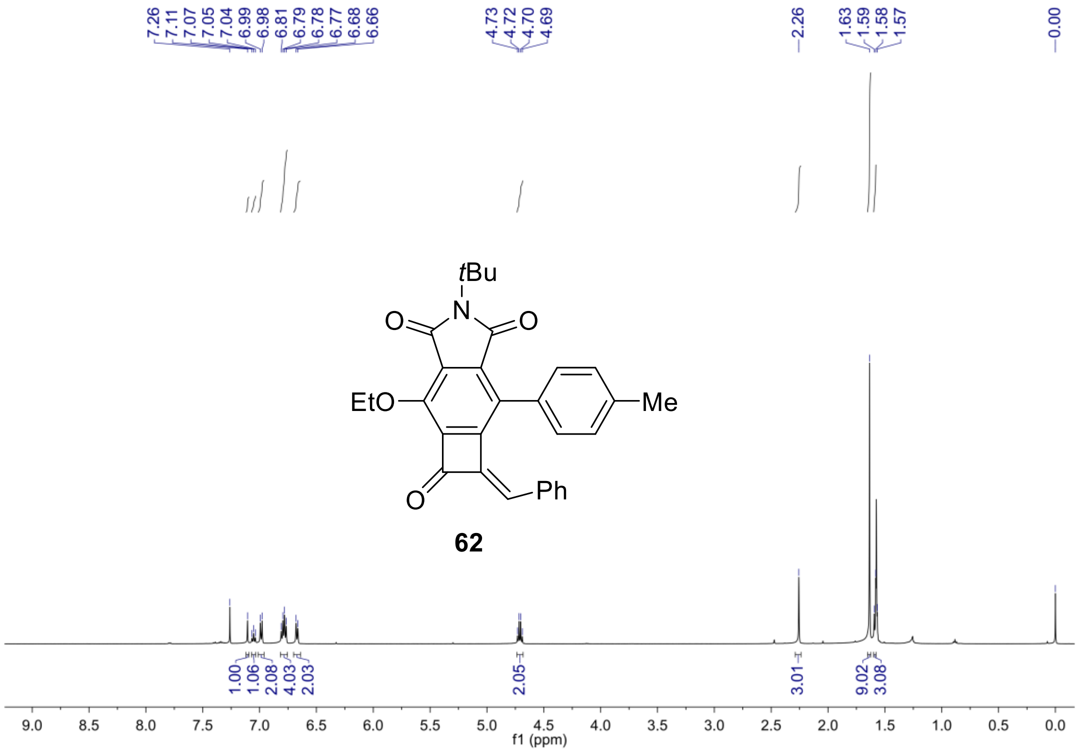

Supplementary Fig. 204 | ^{1}H NMR of compound 62 (500 MHz, at 25 °C, in $CDCl_3$).

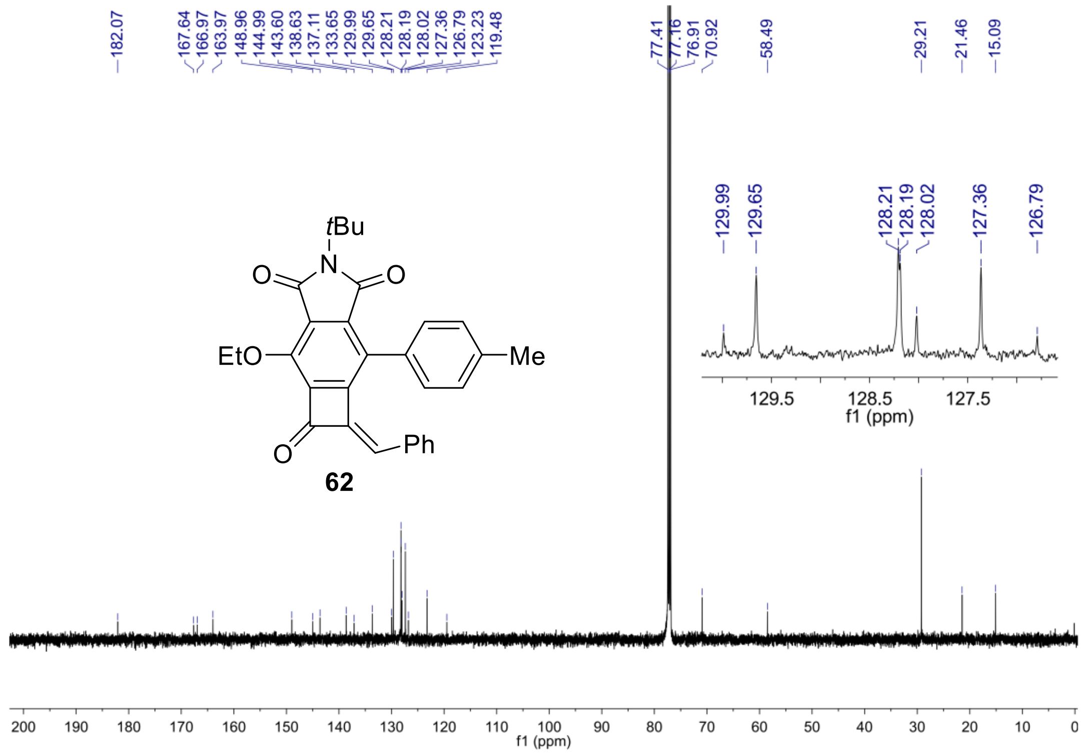

Supplementary Fig. 205 | ^{13}C NMR of compound 62 (125 MHz, at 25 °C, in $CDCl_3$).

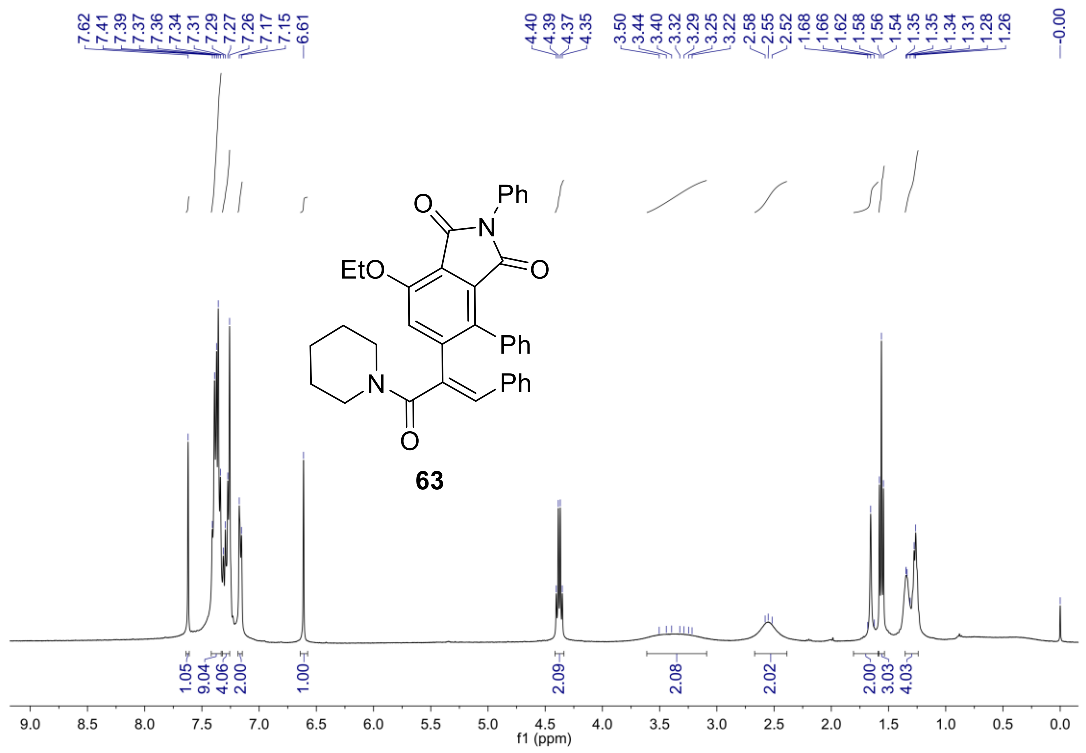

Supplementary Fig. 206 | ^{1}H NMR of compound 63 (400 MHz, at 25 °C, in $CDCl_3$).

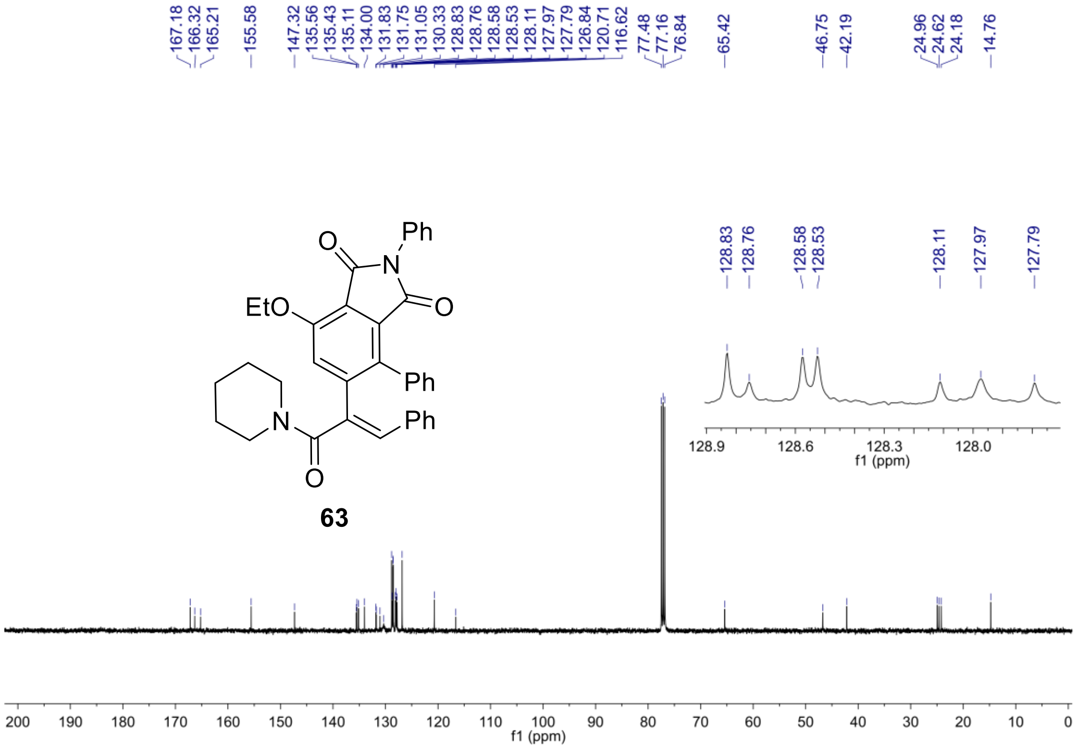

Supplementary Fig. 207 | ^{13}C NMR of compound 63 (100 MHz, at 25 °C, in $CDCl_3$).

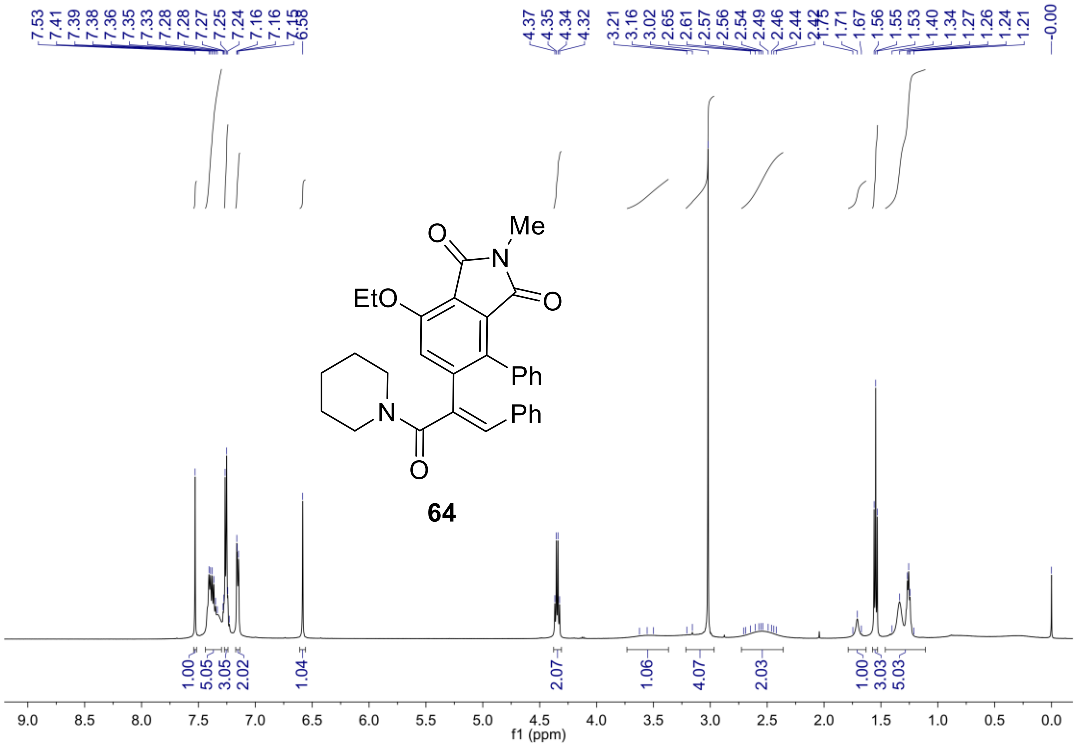

Supplementary Fig. 208 | ^{1}H NMR of compound 64 (500 MHz, at 25 °C, in $CDCl_3$).

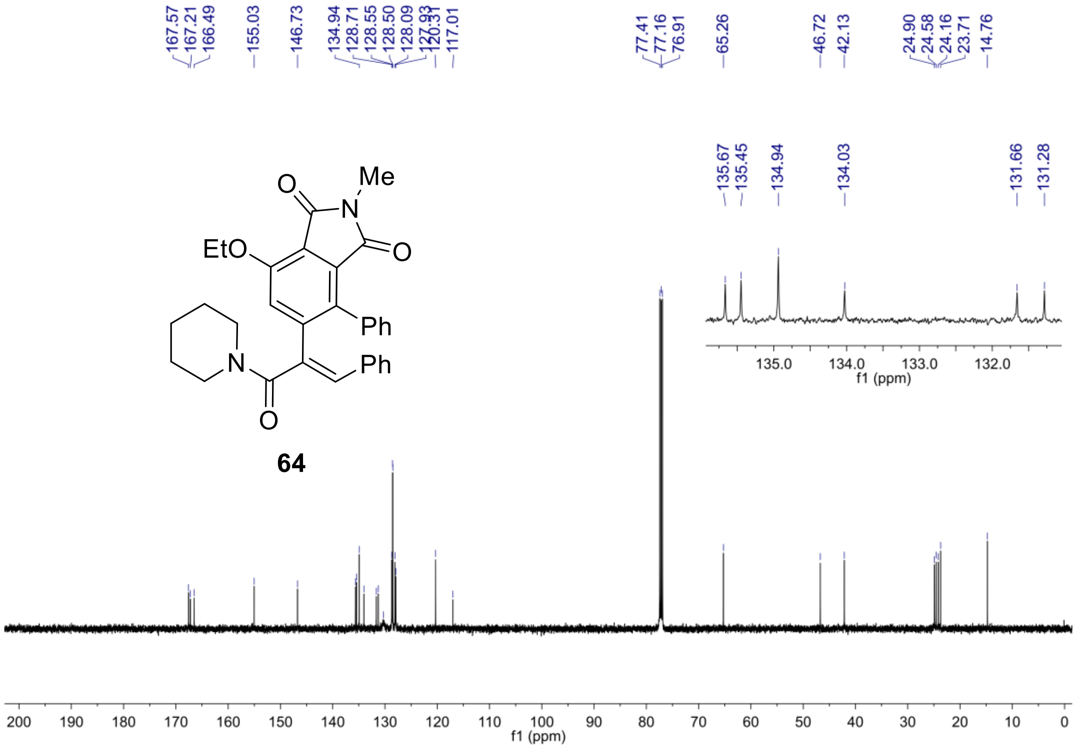

64

Supplementary Fig. 209 | ^{13}C NMR of compound 64 (125 MHz, at 25 °C, in $CDCl_3$).

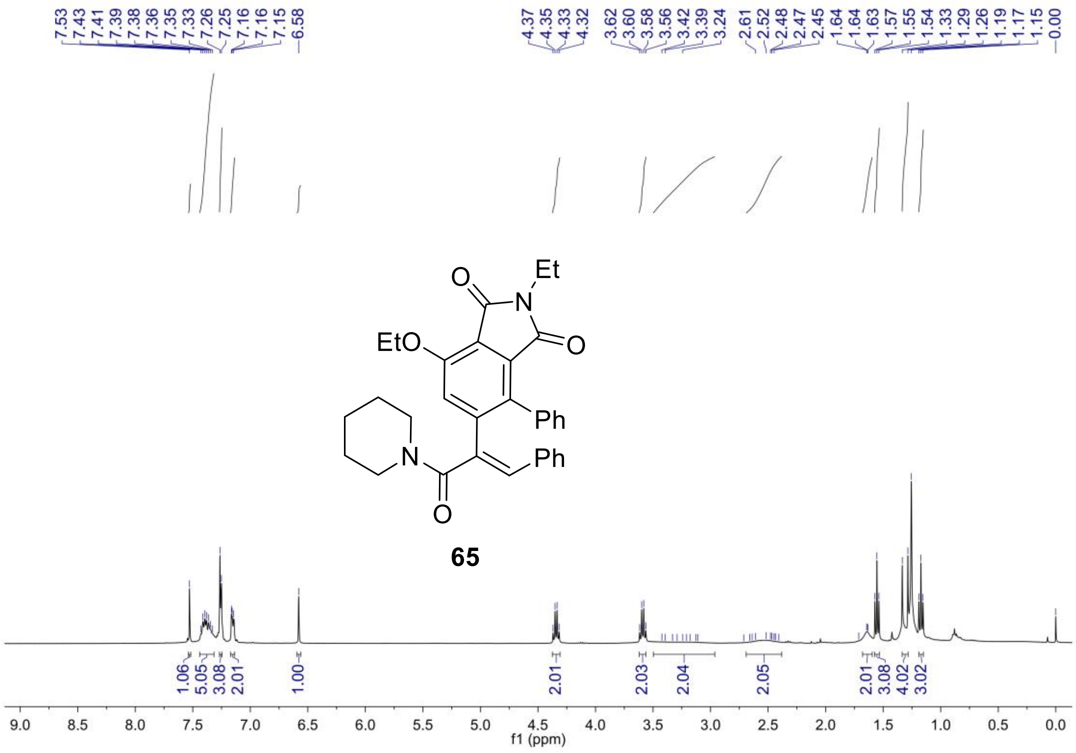

65

Supplementary Fig. 210 | ^{1}H NMR of compound 65 (400 MHz, at 25 °C, in $CDCl_3$).

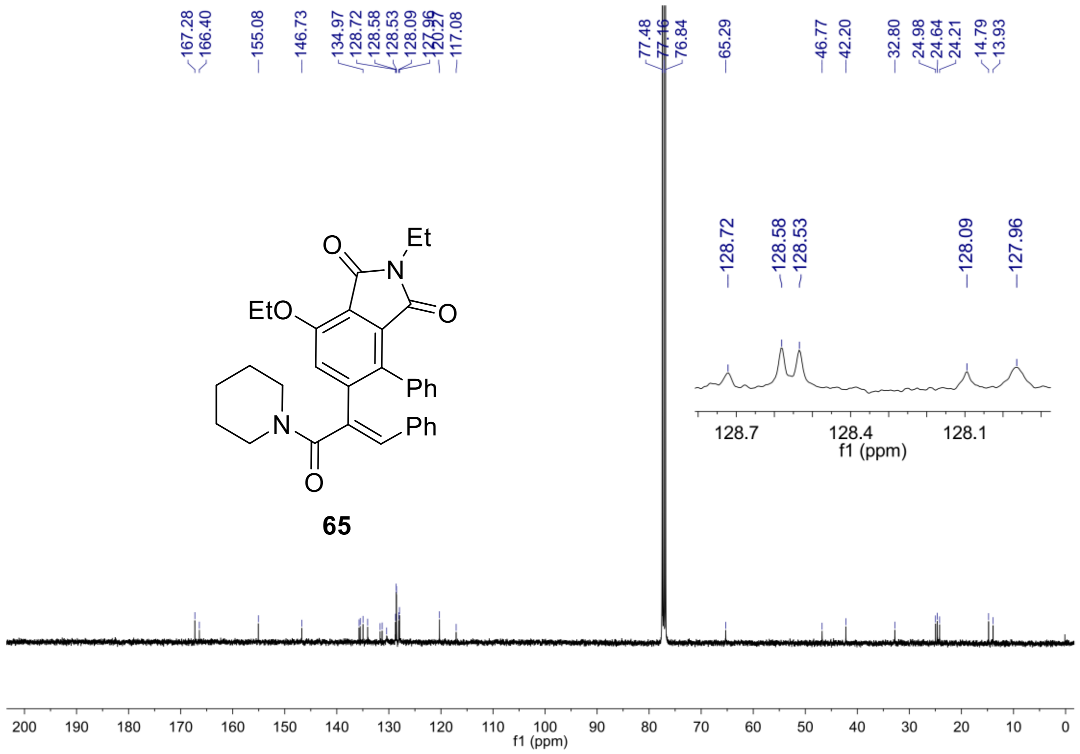

Supplementary Fig. 211 | ^{13}C NMR of compound 65 (100 MHz, at 25 °C, in $CDCl_3$).

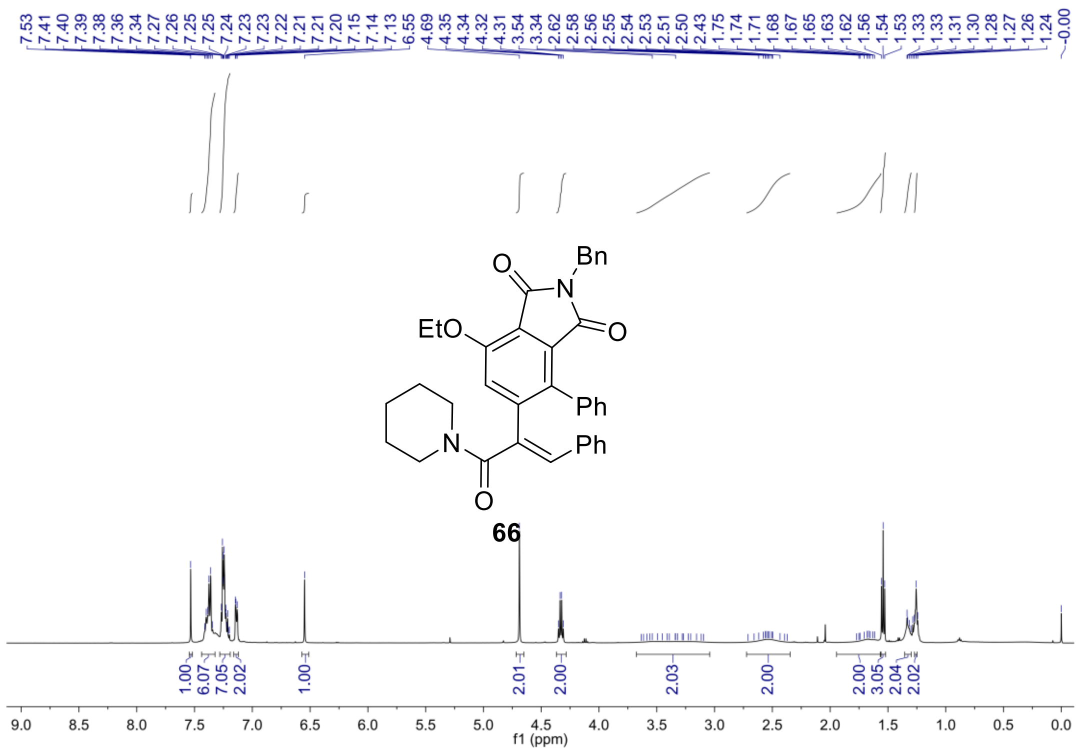

Supplementary Fig. 212 | ^{1}H NMR of compound 66 (500 MHz, at 25 °C, in $CDCl_3$).

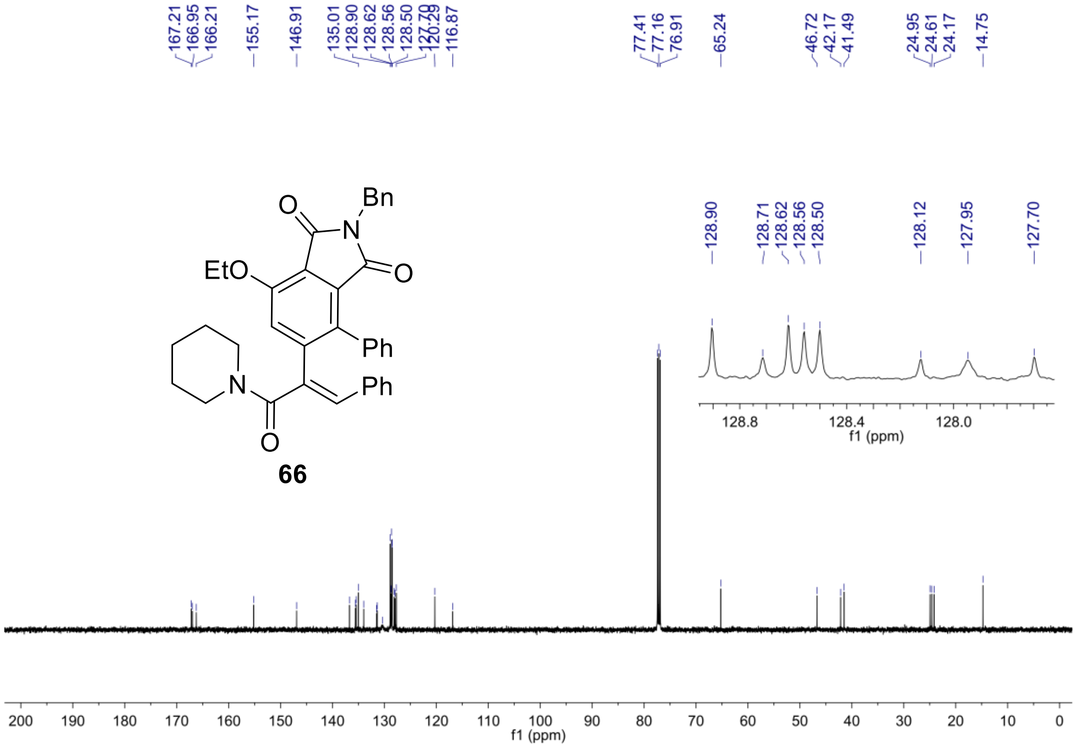

Supplementary Fig. 213 | ^{13}C NMR of compound 66 (125 MHz, at 25 °C, in $CDCl_3$).

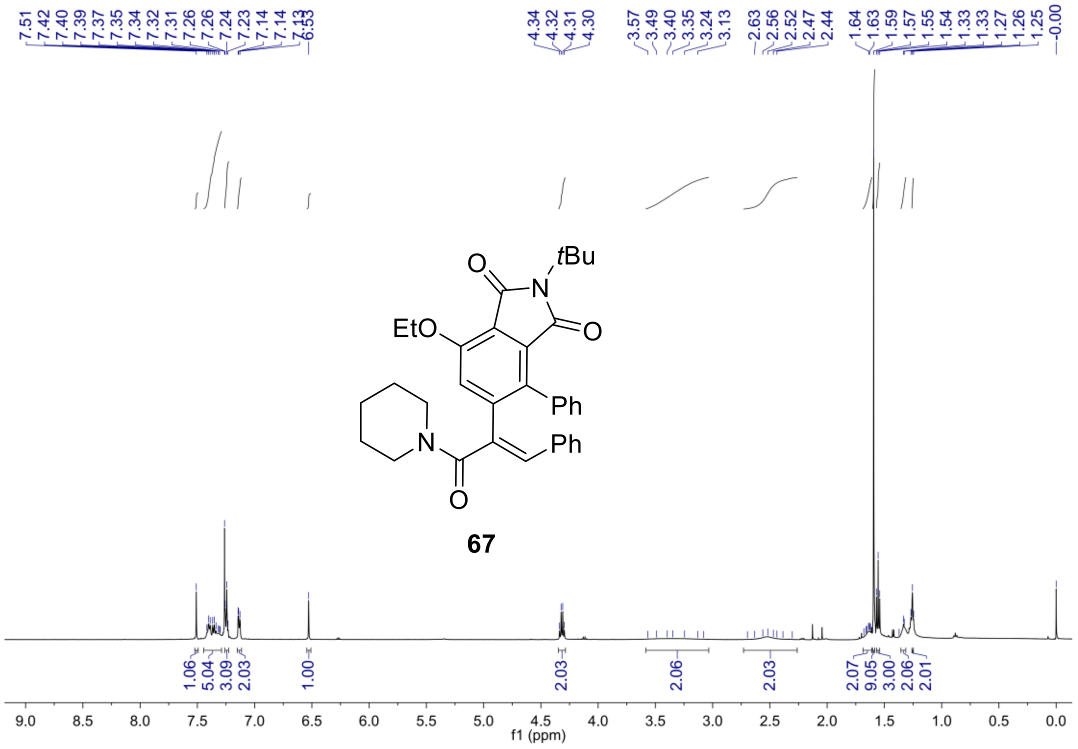

Supplementary Fig. 214 | ^{1}H NMR of compound 67 (500 MHz, at 25 °C, in $CDCl_3$).

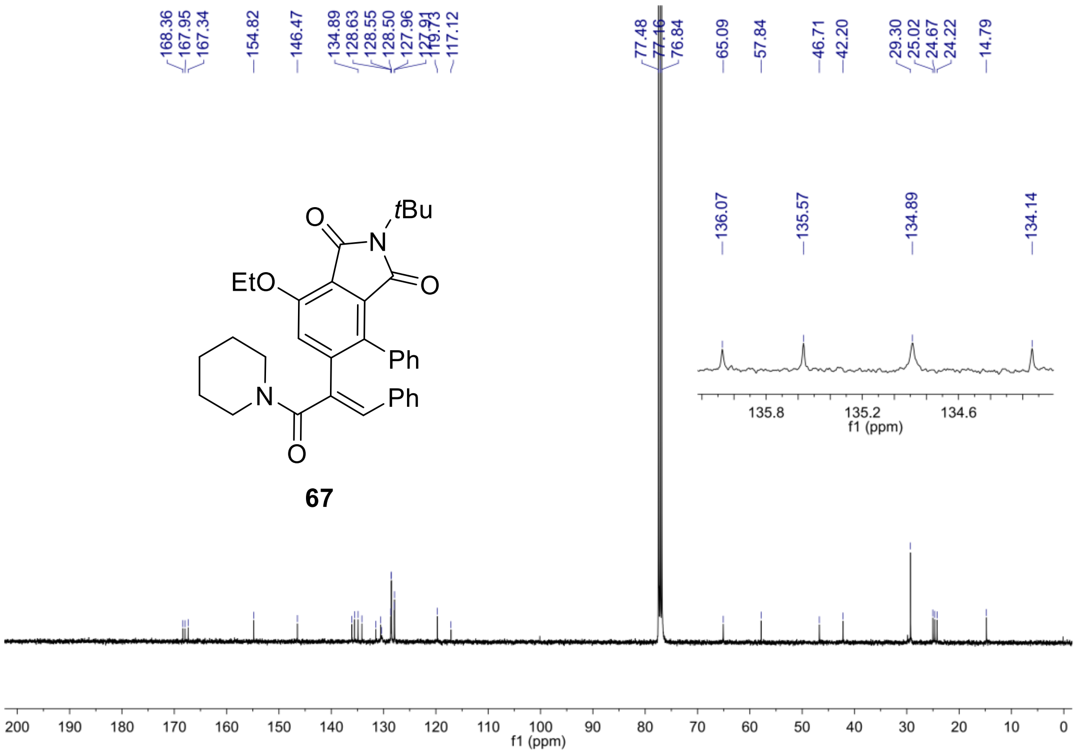

Supplementary Fig. 215 | ^{13}C NMR of compound 67 (125 MHz, at 25 °C, in $CDCl_3$).

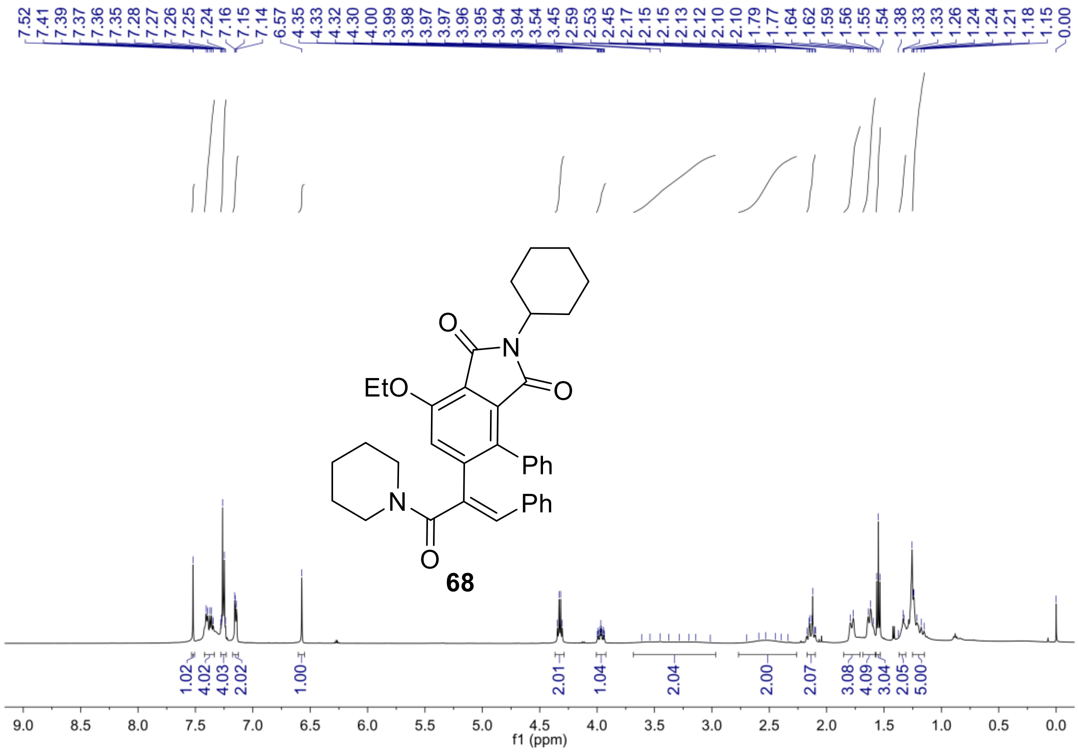

Supplementary Fig. 216 | ^{1}H NMR of compound 68 (500 MHz, at 25 °C, in $CDCl_3$).

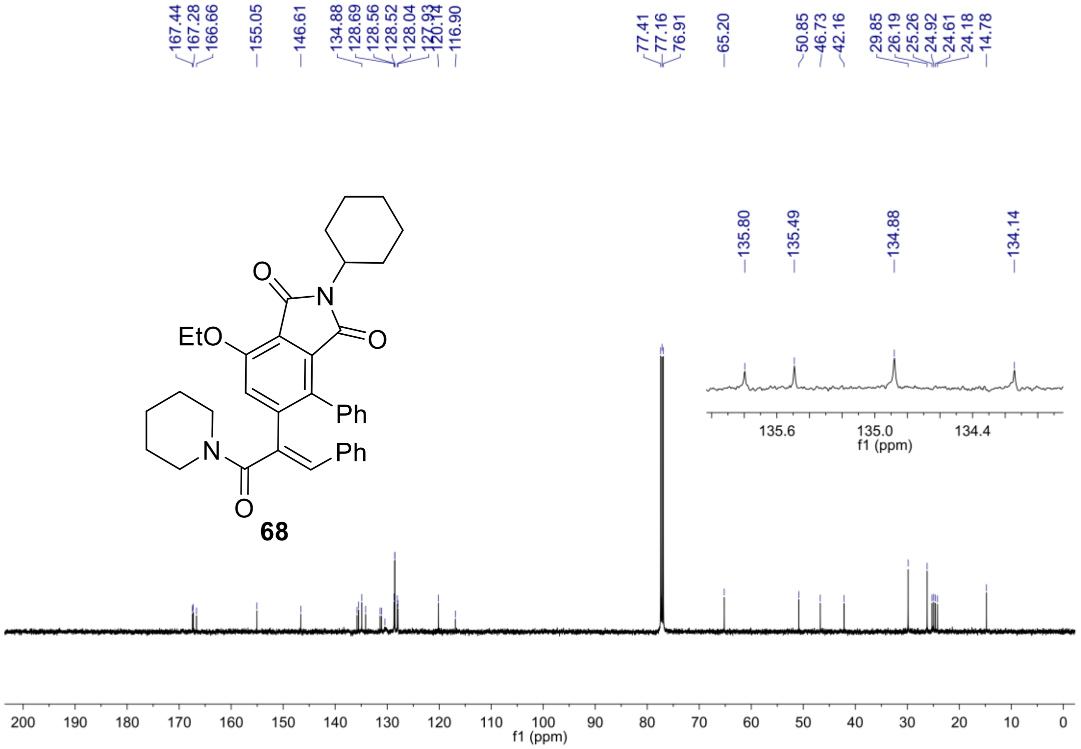

Supplementary Fig. 217 | ^{13}C NMR of compound 68 (125 MHz, at 25 ^{o}C, in $CDCl_3$).

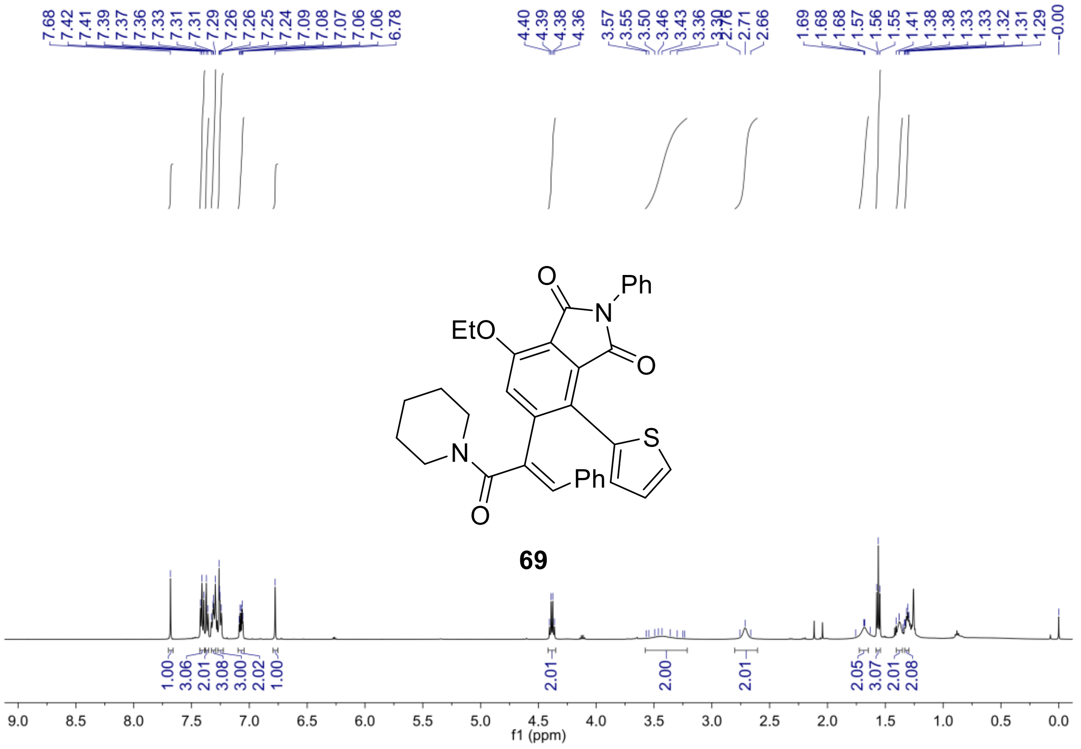

Supplementary Fig. 218 | ^{1}H NMR of compound 69 (500 MHz, at 25 ^{o}C, in $CDCl_3$).

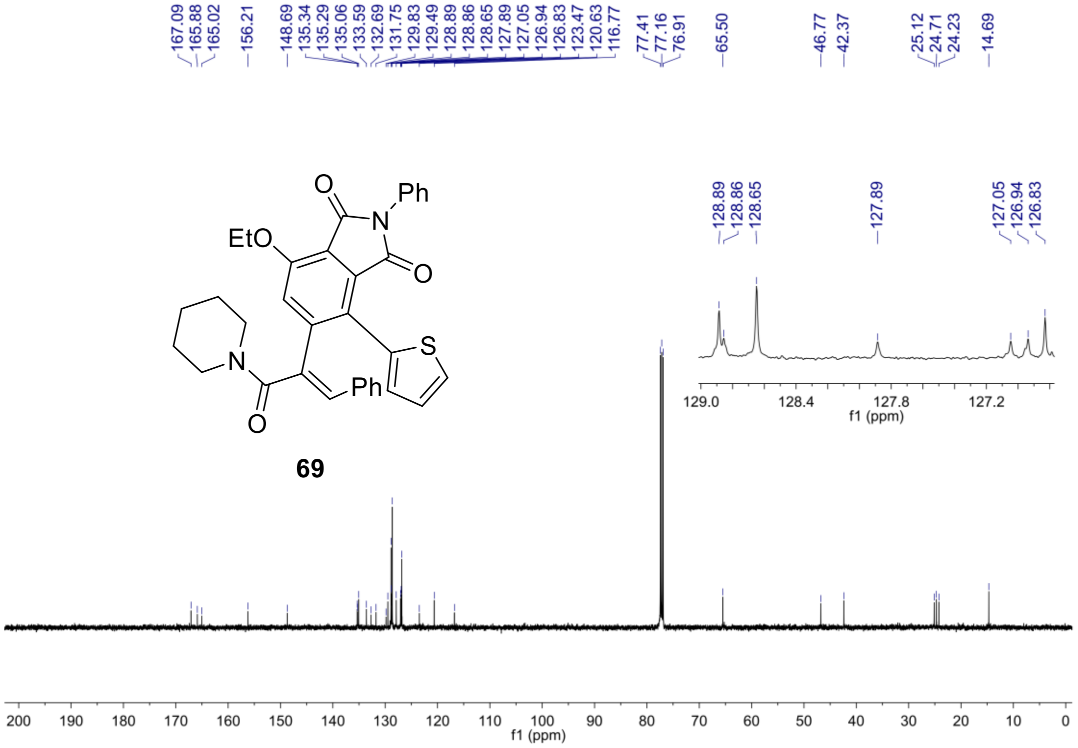

Supplementary Fig. 219 | ^{13}C NMR of compound 69 (125 MHz, at 25 °C, in $CDCl_3$).

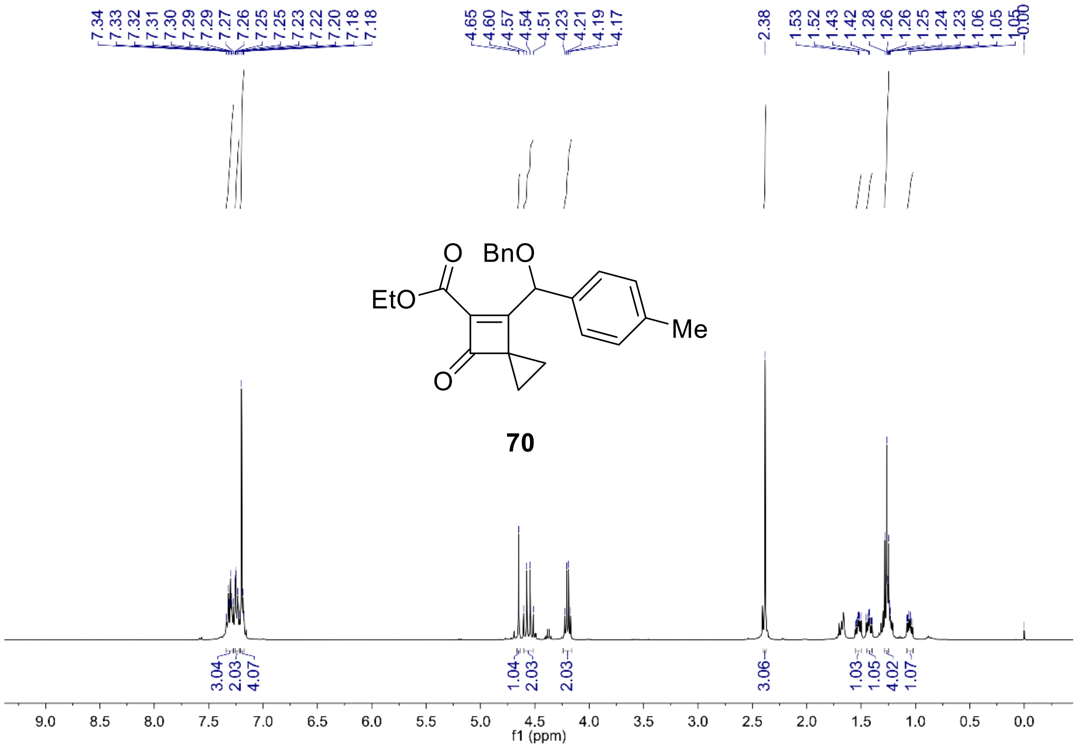

Supplementary Fig. 220 | ^{1}H NMR of compound 70 (400 MHz, at 25 °C, in $CDCl_3$).

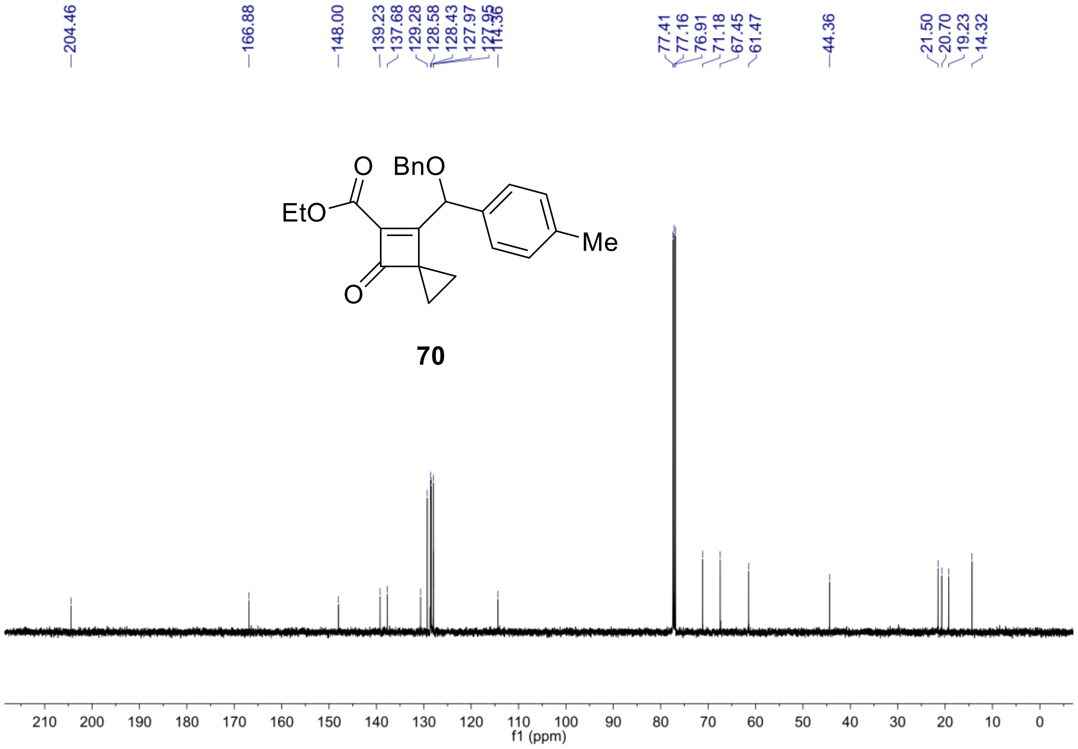

Supplementary Fig. 221 | ^{13}C NMR of compound 70 (100 MHz, at 25 °C, in $CDCl_3$).

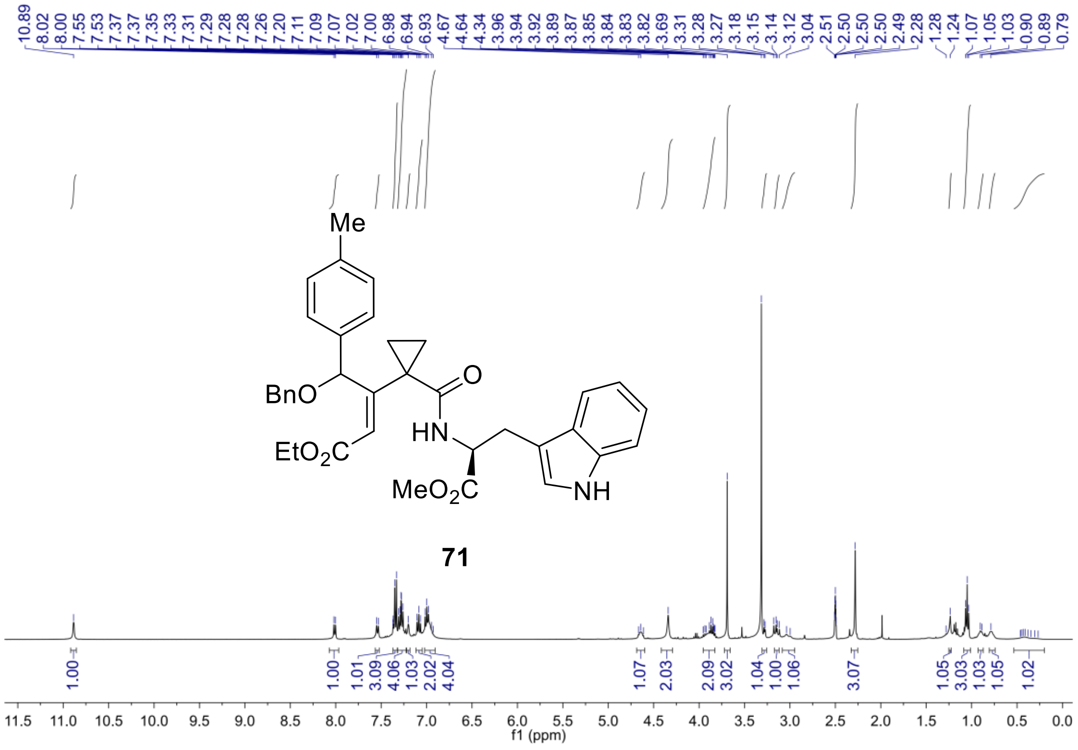

Supplementary Fig. 222 | ^{1}H NMR of compound 71 (400 MHz, at 25 °C, in DMSO).

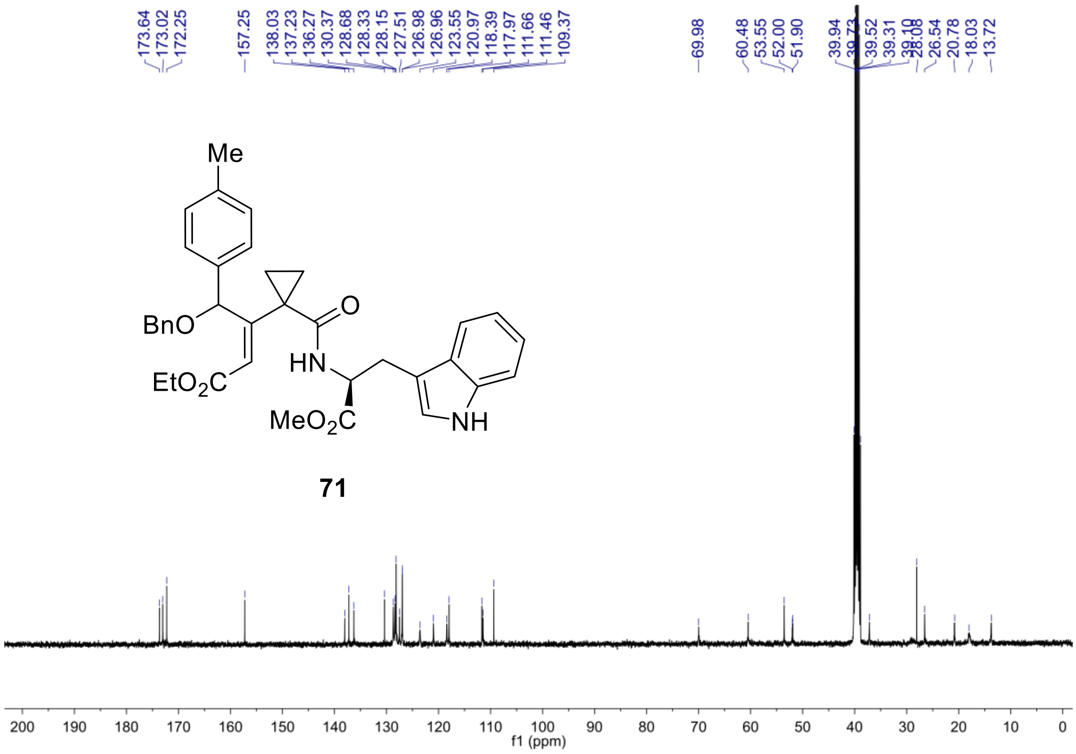

Supplementary Fig. 223 | ^{13}C NMR of compound 71 (100 MHz, at 25 °C, in DMSO).

X-Ray crystal structure and data for 2g, 2af, 38, 59, and 71

Crystallographic data for 2g.

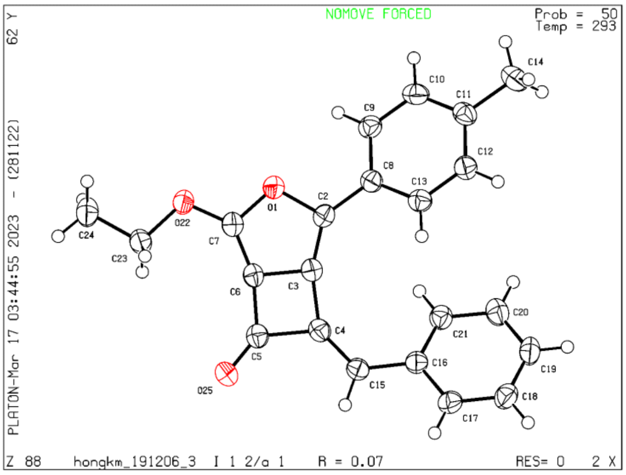

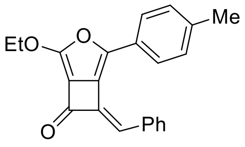

2g

CCDC: 2249373

Supplementary Fig. 224 | Crystallographic data of compound 2g

Datablock: hongkm_191206_3

Bond precision: C-C = 0.0040 A Wavelength=1.54184

Cell:	a=7.8189(2)	b=13.1571(3)	c=32.8959(8)
	alpha=90	beta=93.053(2)	gamma=90
Temperature:	293 K		

	Calculated	Reported
Volume	3379.33(14)	3379.33(14)
Space group	I 2/a	I 1 2/a 1
Hall group	-I 2ya	-I 2ya
Moiety formula	C22 H18 O3	C22 H18 O3
Sum formula	C22 H18 O3	C22 H18 O3
Mr	330.36	330.36
Dx,g cm-3	1.299	1.299
Z	8	8
Mu (mm-1)	0.687	0.687
F000	1392.0	1392.0
F000’	1396.21	
h,k,lmax	9,16,41	9,16,41
Nref	3567	3509
Tmin,Tmax	0.960,0.966	0.855,1.000
Tmin’	0.934	

Correction method= # Reported T Limits: Tmin=0.855 Tmax=1.000
AbsCorr = MULTI-SCAN

Data completeness= 0.984 Theta(max)= 76.970

R(reflections)= 0.0705(3203) wR2(reflections)= 0.1737(3509)

S = 1.199 Npar= 229

Crystallographic data for 2af.

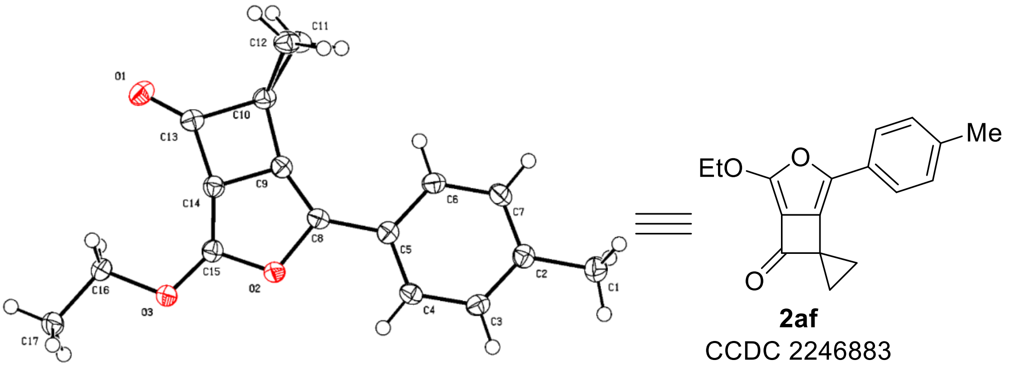

2af
CCDC 2246883

Supplementary Fig. 225 | Crystallographic data of compound 2af

Datablock: 20221010-lmt-2_auto

Bond precision: C-C = 0.0017 A Wavelength=1.54184

Cell:	a=14.1728(3)	b=13.2998(3)	c=14.8890(3)
	alpha=90	beta=104.472(2)	gamma=90
Temperature:	100 K		

	Calculated	Reported
Volume	2717.46(10)	2717.46(10)
Space group	I 2/c	I 1 2/c 1
Hall group	-I 2yc	-I 2yc
Moiety formula	C17 H16 O3	C17 H16 O3
Sum formula	C17 H16 O3	C17 H16 O3
Mr	268.30	268.30
Dx,g cm-3	1.312	1.312
Z	8	8
Mu (mm-1)	0.721	0.721
F000	1136.0	1136.0
F000'	1139.51	
h,k,lmax	18,17,19	18,16,17
Nref	3099	2685
Tmin,Tmax	0.841,0.965	0.677,1.000
Tmin'	0.805	

Correction method= # Reported T Limits: Tmin=0.677 Tmax=1.000
AbsCorr = MULTI-SCAN

Data completeness= 0.866 Theta(max)= 85.723

R(reflections)= 0.0401(2633) wR2(reflections)= 0.1172(2685)

S = 1.179 Npar= 183

Crystallographic data for 38.

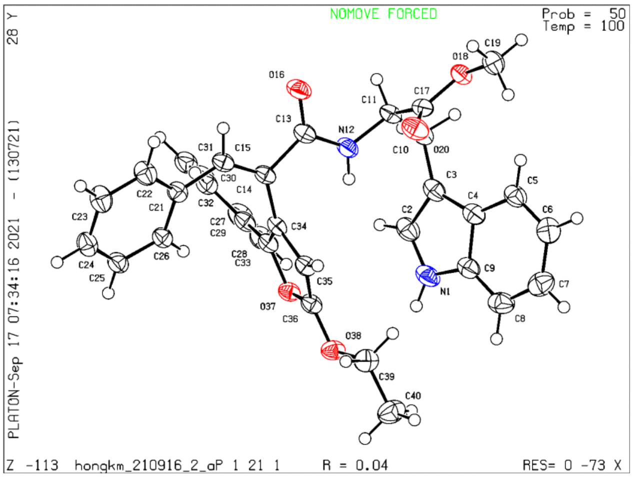

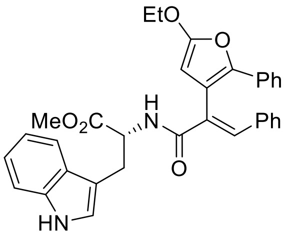

38
CCDC: 2110463

Supplementary Fig. 226 | Crystallographic data of compound 38

Datablock: hongkm_210916_2_auto

Bond precision: C-C = 0.0039 A Wavelength=1.54184

Cell:	a=8.9033(1)	b=13.9107(2)	c=11.3945(2)
	alpha=90	beta=92.947(1)	gamma=90
Temperature:	100 K		

	Calculated	Reported
Volume	1409.36(4)	1409.36(4)
Space group	P 21	P 1 21 1
Hall group	P 2yb	P 2yb
Moiety formula	C33 H30 N2 O5	C33 H30 N2 O5
Sum formula	C33 H30 N2 O5	C33 H30 N2 O5
Mr	534.59	534.59
Dx,g cm-3	1.260	1.260
Z	2	2
Mu (mm-1)	0.689	0.689
F000	564.0	564.0
F000’	565.73	
h,k,lmax	11,17,14	11,17,14
Nref	6088[3173]	5853
Tmin,Tmax	0.883,0.933	0.670,1.000
Tmin’	0.871	

Correction method= # Reported T Limits: Tmin=0.670 Tmax=1.000
AbsCorr = MULTI-SCAN

Data completeness= 1.84/0.96 Theta(max)= 78.852

R(reflections)= 0.0434(5405) wR2(reflections)= 0.1170(5853)

S = 1.082 Npar= 363

Crystallographic data for 59.

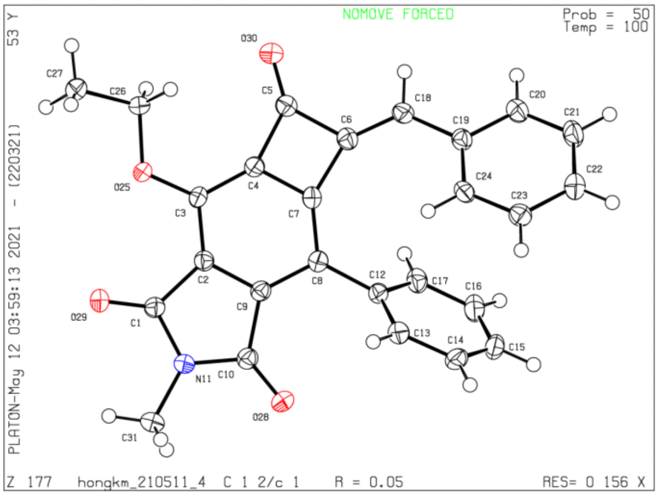

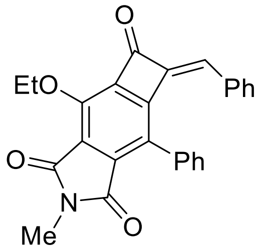

59

CCDC: 2083438

Supplementary Fig. 227 | Crystallographic data of compound 59

Datablock: hongkm_210511_4

Bond precision: C-C = 0.0030 A Wavelength=1.54184

Cell:	a=10.8177(4)	b=19.7267(8)	c=19.0701(8)
	alpha=90	beta=102.808(4)	gamma=90
Temperature:	100 K		

	Calculated	Reported
Volume	3968.3(3)	3968.3(3)
Space group	C 2/c	C 1 2/c 1
Hall group	-C 2yc	-C 2yc
Moiety formula	C26 H19 N O4	C26 H19 N O4
Sum formula	C26 H19 N O4	C26 H19 N O4
Mr	409.42	409.42
Dx,g cm-3	1.371	1.371
Z	8	8
Mu (mm-1)	0.754	0.754
F000	1712.0	1712.0
F000’	1717.35	
h,k,lmax	13,24,24	13,24,24
Nref	4167	3983
Tmin,Tmax	0.956,0.963	0.940,1.000
Tmin’	0.941	

Correction method= # Reported T Limits: Tmin=0.940 Tmax=1.000
AbsCorr = MULTI-SCAN

Data completeness= 0.956 Theta(max)= 76.289

R(reflections)= 0.0512(3187) wR2(reflections)= 0.1256(3983)

S = 1.026 Npar= 282

Crystallographic data for 71.

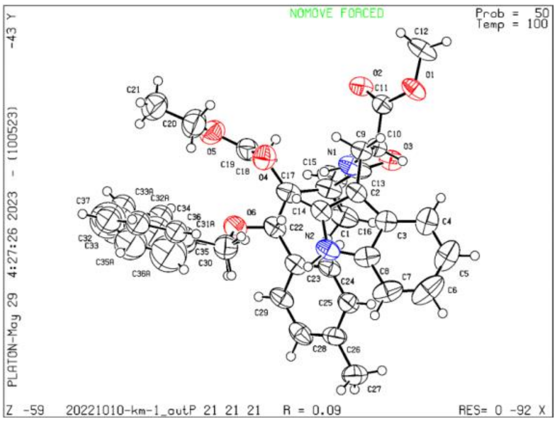

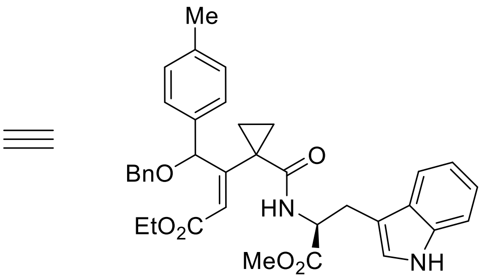

71
CCDC: 2265899

Supplementary Fig. 228 | Crystallographic data of compound 71

Datablock: 20221010-km-1_auto

Bond precision: C-C = 0.0088 A Wavelength=1.54184

Cell:	a=8.7479(3)	b=10.2932(3)	c=36.2659(13)
	alpha=90	beta=90	gamma=90
Temperature:	100 K		

	Calculated	Reported
Volume	3265.52(19)	3265.52(19)
Space group	P 21 21 21	P 21 21 21
Hall group	P 2ac 2ab	P 2ac 2ab
Moiety formula	C36 H38 N2 O6	C36 H38 N2 O6
Sum formula	C36 H38 N2 O6	C36 H38 N2 O6
Mr	594.68	594.68
Dx,g cm-3	1.210	1.210
Z	4	4
Mu (mm-1)	0.665	0.665
F000	1264.0	1264.0
F000’	1267.86	
h,k,lmax	10,12,45	10,12,44
Nref	6760[3842]	6016
Tmin,Tmax	0.923,0.967	0.868,1.000
Tmin’	0.875	

Correction method= # Reported T Limits: Tmin=0.868 Tmax=1.000
AbsCorr = MULTI-SCAN

Data completeness= 1.57/0.89 Theta(max)= 75.562

R(reflections)= 0.0882(5224) wR2(reflections)= 0.2517(6016)

S = 1.041 Npar= 381

General procedure for the *in vitro* anti-tumor activity atudy

Cell viability was measured by CCK-8 assay

Human cancer cell lines HCT116, A549, MB-231 and SJSA-1 were obtained from Cell Cook. Cells were cultured in RPMI1640 medium containing 10% fetal bovine serum and 1% penicillin/streptomycin (Gibco) in a humidified incubator containing 5% CO2 at 37 °C. Human cancer cell lines MCF-7 was obtained from Procell and cells were cultured in MEM medium containing 10% fetal bovine serum, 1% penicillin/streptomycin (Gibco) and 0.01 mg/mL insulin (Procell) in a humidified incubator containing 5% CO_2 at 37 °C. For cell viability, cells were seeded in 96-well plates at 5000 cells per well. After 24 hours, serially diluted compounds were added and cells were cultured for another 48 hours. Cell viability was measured using a Cell Counting Kit-8 (CCK-8) assay according to the manufacturer's instructions (Yeasen Biotechnology, China).

These representative products 6a, **9 - 10**, **14 - 21**, **23 - 40**, **42 - 43**, **46 - 47**, **49 - 55**, **63 – 66**, and **68** on cell viability was evaluated *via* CCK8 assay in HCT116 (colon cancer), MCF-7 (breast cancer), A549 (lung adenocarcinoma), SJSA-1 (osteosarcoma cancer) and MDA-MB-231 (breast cancer) human cancer cell lines, and the *in vitro* anti-tumor activity results are listed in **Supplementary Table 1** and **Supplementary Table 2**.

Most of these furan derivatives showed significant anti-cancer activity, compounds **21, 29,** and **50** were more potent than other compounds (**Supplementary Fig. 229-231**). The results show that the substituted furan derivatives with ring-opening of methyl *L*-isoleucinate (compound **29**) show the highest anticancer potency against human colon cancer cells (HCT116 cells, IC_{50}=1.19 μM, R^2=0.9838). The results were presented as percentages and vehicle-treated cells set at 5000 (**Supplementary Fig. 230**).

Supplementary Table 1 | Anti-tumor activities of compounds **6a, 9 – 10, 14 – 21, 23 – 40, 42 – 43, 46 – 47, 49 – 55, 63 – 66** and **68** (Inhibition rate at 20 μM)

Compound	HCT-116 (%)	MCF-7 (%)	A549 (%)	SJSA-1 (%)	MB-231 (%)
**6a**	73.34±0.69	12.05±7.93	53.36±2.25	84.26±0.69	< 0
**9**	89.45±1.62	43.30±9.25	1.56	98.16±0.00	94.18±2.88
**10**	99.37±0.31	61.57±1.20	95.48±1.32	37.75±3.34	15.76±4.10
**14**	96.21±3.54	50.38±1.83	95.92±0.04	88.93±1.47	90.29±0.54
**15**	98.13±0.62	86.52±2.76	97.60±0.41	86.29±4.45	53.29±5.34
**16**	96.63±0.33	21.76±0.58	81.84±2.99	70.66±1.01	23.74±2.95
**17**	49.84±1.31	18.90±0.29	< 0	61.17±8.72	22.39±5.46
**18**	94.89±0.17	26.38±17.78	69.58±5.70	83.77±4.97	45.79±4.96
**19**	94.02±2.27	13.87	77.39±6.73	< 0	16.09±6.06
**20**	97.88±0.54	25.73±1.08	6.32±4.79	38.79±9.83	20.00±5.61
**21**	91.84±0.54	91.28±4.55	49.18±5.91	98.70±0.00	96.80±1.88
**23**	54.90±0.35	8.90	37.80±7.60	< 0	13.72±1.69
**24**	61.37±3.62	27.93±12.48	< 0	80.00±2.45	58.00±0.00
**25**	90.34±0.80	84.12±3.44	86.64±4.73	92.86±0.1	64.04±0.44
**26**	55.54±3.84	19.15±6.57	38.76±0.125	3.66±0.42	12.44±3.04
**27**	23.33±6.50	23.44±1.52	22.37±8.87	96.92±0.29	66.97±2.80
**28**	87.50±1.51	38.72±10.95	92.11±1.84	90.05±0.47	66.23±2.78
**29**	99.34±0.26	75.26±0.71	96.09±0.47	98.04±0.15	23.46±6.05
**30**	26.62±7.26	6.89	8.48	3.03±1.63	11.02±0.80
**31**	18.61±1.00	< 0	< 0	58.55±4.66	< 0
**32**	< 0	0.45	15.25±1.76	< 0	14.26±5.39
**33**	12.63	< 0	15.31±2.14	10.61±2.97	1.69±0.24
**34**	96.47±0.32	35.88±8.33	77.32±4.95	71.59±1.63	32.97±1.74
**35**	87.71±0.41	< 0	34.54±7.26	60.63±4.00	17.19±5.60
**36**	72.47±1.15	34.48±6.76	0.28	97.08±0.46	89.07±0.94
**37**	46.67±1.26	38.38±2.60	49.07±3.94	13.25±1.29	3.54±0.00
**38**	86.61±4.57	< 0	19.80±5.45	66.99±2.09	10.21±4.57
**39**	90.93±1.65	17.57±0.08	36.46±1.74	87.45±2.98	12.25±3.32
**40**	95.44±0.68	< 0	75.74±0.00	73.50±0.93	56.68±4.04
**42**	59.47±0.77	13.29±4.11	< 0	47.82±4.98	34.28±6.29
**43**	98.50±1.19	62.98±2.46	88.70±0.00	97.99±0.00	9.50±2.30
**46**	94.10±2.41	12.11±3.52	54.10±3.59	40.05±5.24	16.78±1.71
**47**	91.48±3.05	20.74±3.95	71.37±2.50	84.4±3.05	25.54±3.77
**49**	98.47±0.62	36.94±3.81	85.97±6.54	91.77±1.26	62.91±8.30
**50**	97.33±4.00	100.73±0.29	99.64±0.036	99.33±0.08	93.35±3.70
**51**	< 0	27.20	4.68	< 0	< 0
**52**	< 0	< 0	< 0	< 0	< 0
**53**	< 0	7.47	2.13	7.19	15.11
**54**	< 0	< 0	< 0	< 0	< 0
**55**	34.87	< 0	1.10	32.94±4.59	20.65±8.28
**63**	30.45±3.23	31.67±7.52	45.04±7.36	30.21±8.71	18.70±4.08
**64**	< 0	68.77±2.78	87.58±0.25	84.75±0.62	45.44±1.13
**65**	50.43	< 0	17.15±9.27	35.77±3.17	28.03±1.92
**66**	< 0	< 0	30.94	45.10±0.31	39.67±0.56
**68**	20.66±3.71	16.82±1.88	< 0	11.88±2.54	36.26±1.67

Supplementary Table 2 | Anti-tumor activities of compounds **6a, 9 - 10, 14 - 16, 18 - 21, 24 - 25, 28 - 29, 34 - 36, 38 - 40, 43, 46 - 47, 49 - 50, 64** (IC_{50}, μM)

Compound	HCT-116 (%)	MCF-7 (%)	A549 (%)	SJSA-1 (%)	MB-231 (%)
**6a**	7.25±0.46	-	-	6.55±0.51	-
**9**	3.67±0.26	-	-	6.39±0.45	18.68±1.67
**10**	4.00±0.16	-	4.70±0.34	-	-
**14**	6.23±0.41	-	6.18±0.31	-	6.59±0.54
**15**	6.11±0.32	7.74±0.35	7.87±0.42	-	-
**16**	11.71±0.71	-	-	-	-
**18**	11.83±0.68	-	19.62±1.70	-	-
**19**	15.02±0.88	-	18.41±0.69	-	-
**20**	11.09±0.43	-	-	-	-
**21**	2.33±0.16	6.85±0.33	-	3.47±0.26	12.62±1.22
**24**	-	-	-	9.43±0.53	-
**25**	3.62±0.15	3.47±0.33	6.06±0.34	-	-
**28**	2.28±0.11	-	4.13±0.24	-	-
**29**	1.19± 0.06	10.40±0.78	2.74±0.09	2.08±0.09	-
**34**	8.08±0.38	-	-	-	-
**35**	13.12±1.51	-	-	-	-
**36**	6.37±0.39	-	-	8.50±0.34	-
**38**	10.24±1.64	-	-	15.03±1.34	-
**39**	17.37±2.64	-	-	-	-
**40**	2.68±0.10	-	7.7±0.35	4.18±0.30	-
**43**	11.53±1.23	-	8.85±0.74	7.83±0.99	-
**46**	10.81±0.57	-	-	-	-
**47**	7.89±0.34	-	13.87±1.19	-	-
**49**	5.85±0.30	-	11.22±1.01	-	-
**50**	4.42±0.30	4.23±0.352	6.43±0.18	4.30±0.28	6.88±0.14
**64**	-	3.99±0.36	8.11±0.39	-	-
**Camptothecin**	0.10±0.01	0.36±0.09	0.25±0.05	0.04±0.01	0.03±0.05

IC_{50} is the half maximal inhibitory concentration.

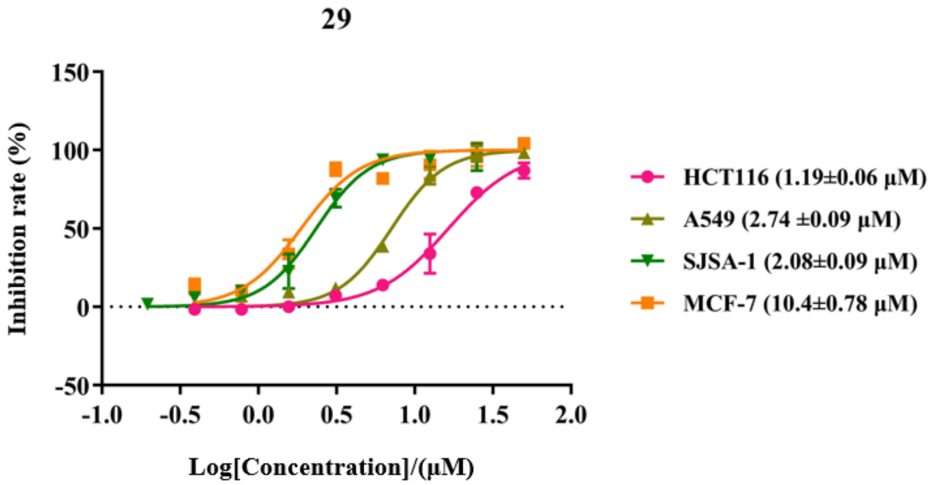

Supplementary Fig. 229 | Compound **21** on the inhibition of HCT116, MCF-7, SJSA-1 and MDA-MB-231 cells.

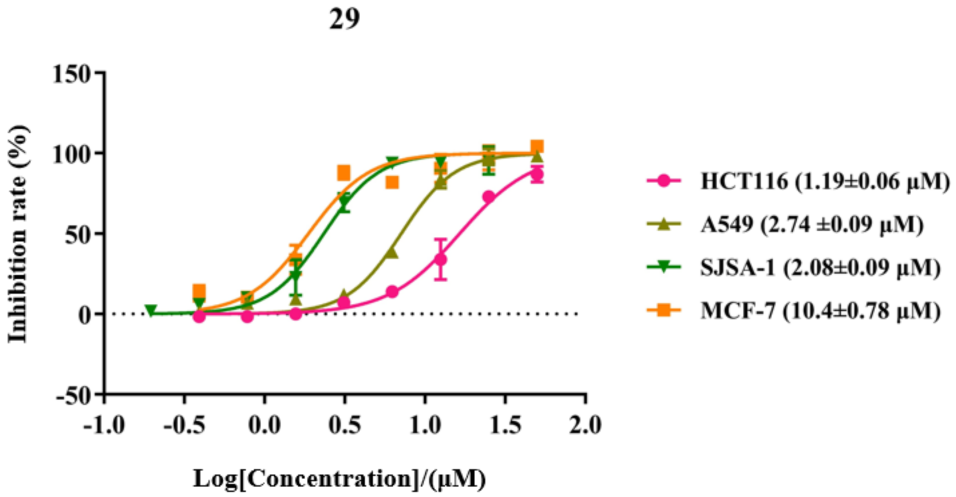

Supplementary Fig. 230 | Compound **29** on the inhibition of HCT116, MCF-7, A549 and SJSA-1 cells.

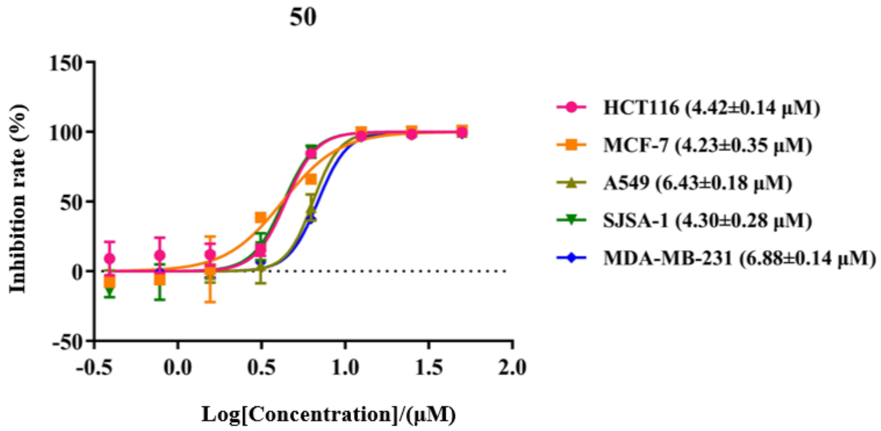

Supplementary Fig. 231 | Compound **50** on the inhibition of HCT116, MCF-7, A549, SJSA-1 and MDA-MB-231 cells.

Computational studies

All DFT calculations were performed with Gaussian 09[2] software package. In the calculation of full potential energy surface using $Rh_2(HCOO)_4$ as catalyst, the functional B3LYP[3,4] and a basis set def2-SVP[5] were employed for optimizing the geometries of minima and transition states in the gas phase, while for chemoselectivity study in the real system, the geometries of minima and transition states were optimized at SMD[6](EtOAc)/B3LYP/def2-SVP level in the solution. Frequency calculations at the same level were performed to validate each structure as either a minimum or a transition state. Quasiharmonic corrections were applied by setting all positive frequencies that are less than 100 cm^{-1} to 100 cm^{-1} using Shermo software package[7]. Pruned integration grids with 99 radial shells and 590 angular points per shell were used. Based on the gas-phase or solution-phase optimized structures, single-point energies were computed at BMK[8]/def2-TZVPP level under SMD model to account for solvation effects of EtOAc. All discussed energy differences were based on the Gibbs energies in EtOAc at 298 K. 3D structure was prepared with CYLview[9]. Noncovalent interactions were analyzed by Multiwfn[10] using fchk file which was generated from Gaussian 09[2] at SMD(EtOAc)/B3LYP/def2-SVP.

In order to investigate the regiochemistry of this reaction, we have carried out a benchmark study on the cyclization steps using the state-of-the-art method DLPNO-CCSD(T)[11-13].

Three elementary steps are computed using several popular functionals in the Rh-catalyzed reactions[14]. All these steps were studied using quantum chemical calculations with the Gaussian 09 software package for DFT calculations and Orca[15] for DLPNO-CCSD(T) calculations. For DFT calculations, pruned integration grids with 99 radial shells and 590 angular points per shell were used. Geometry optimizations of all the stationary points were carried out in the gas phase at the B3LYP/def2-SVP level. Unscaled harmonic frequency calculations at the same level were performed to validate each structure as either a minimum or a transition state.

Based on the optimized structures, single-point energy refinements were performed at DLPNO-CCSD(T) and other popular functionals (BMK[16], M06[17], M06L[18], ωB97X-D[19], PBE0[20], B3LYP-D3(BJ)[21], BP86[22,23]) with the same basis set def2-TZVPP. The results are shown in **Supplementary Table 3**, the BMK functional, which has been used in our previous studies on the Rh-catalyzed cycloaddition[24], performed the best on the cyclization steps compared to other functionals though it overestimated the barrier of nitrogen-release step (M06 performs better). Thus, we choose the BMK functional to study the mechanism and regioselectivity of this reaction.

Supplementary Table 3 | Benchmark Study

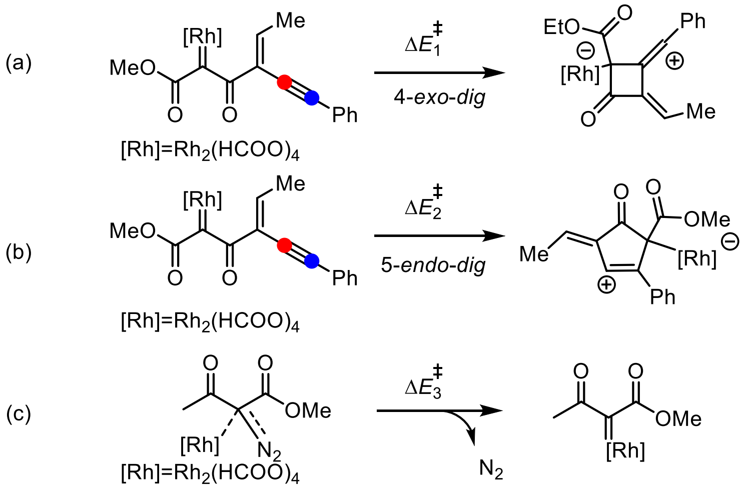

	DLPNO-CCSD(T)	BMK	M06	M06L	ωB97X-D	PBE0	B3LYP-D3	BP86
$\Delta E_1^\ddagger$	4.7	3.5	7.0	7.6	6.4	7.5	5.7	10.2
$\Delta E_2^\ddagger$	6.1	6.0	13.0	14.7	11.3	14.1	11.6	18.9
$\Delta E_3^\ddagger$	15.4	22.8	15.7	14.9	17.4	18.8	15.0	12.4

The formation of the rhodium carbene has been computed (**Supplementary Fig. 232**). The overall activation free energy of this step via **Com1-TS** (computed at SMD(EtOAc)/BMK/def2-TZVPP//B3LYP/def2-SVP) is 28.5 kcal/mol, which is the rate-determined step of this reaction. According to the above benchmark study, BMK functional overestimated the barrier of nitrogen-release step, we also computed the activation free energy at SMD(EtOAc)-M06/def2-TZVPP based on the same

optimized structures (at B3LYP/def2-SVP in the gas phase), the overall activation free energy of carbene formation step via **Com1-TS** is 16.5 kcal/mol, which is also the rate-determined step of this reaction and consistent with the experiments (reaction at 40 °C).

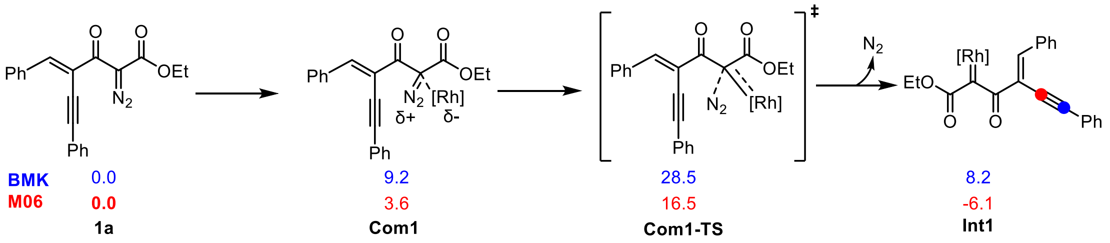

Supplementary Fig. 232 | The formation of the rhodium carbene. Computed at SMD(EtOAc)/BMK/def2-TZVPP//B3LYP/def2-SVP and SMD(EtOAc)/M06/def2-TZVPP//B3LYP/def2-SVP

The Gibbs free energy surface of 4-*exo-dig* pathway using $Rh_2(HCOO)_4$ as catalyst is shown in **Supplementary Fig. 233**. The Rh carbenoid **Int1** undergoes the electrophilic 4-*exo-dig* cyclization *via* **TS1** to afford the vinyl cationic species **Int2**, requiring an activation free energy of 0.6 kcal/mol. Then **Int2** undergoes an intramolecular cyclization *via* **TS2** to afford the product **2a** with an activation free energy of 3.8 kcal/mol. The stepwise carbene/alkyne metathesis (CAM) through 1,3-Rh migration (**TS3**) is disfavored over **TS2** by 4.5 kcal/mol. While CAM via intersystem crossing (ISC) process involving a triplet intermediate ^{3}**Int2** is also disfavored, ^{3}**Int2** has higher free energy of 12.4 kcal/mol than that of **TS2**. Other pathways such as cyclopropenation have also been considered, but we cannot locate the corresponding transition state and 3,4-fused bicyclic intermediate. This is reasonable because this intermediate has large ring strains in the cyclopropene and cyclobutanone (see the dashed box in **Supplementary Fig. 233**).

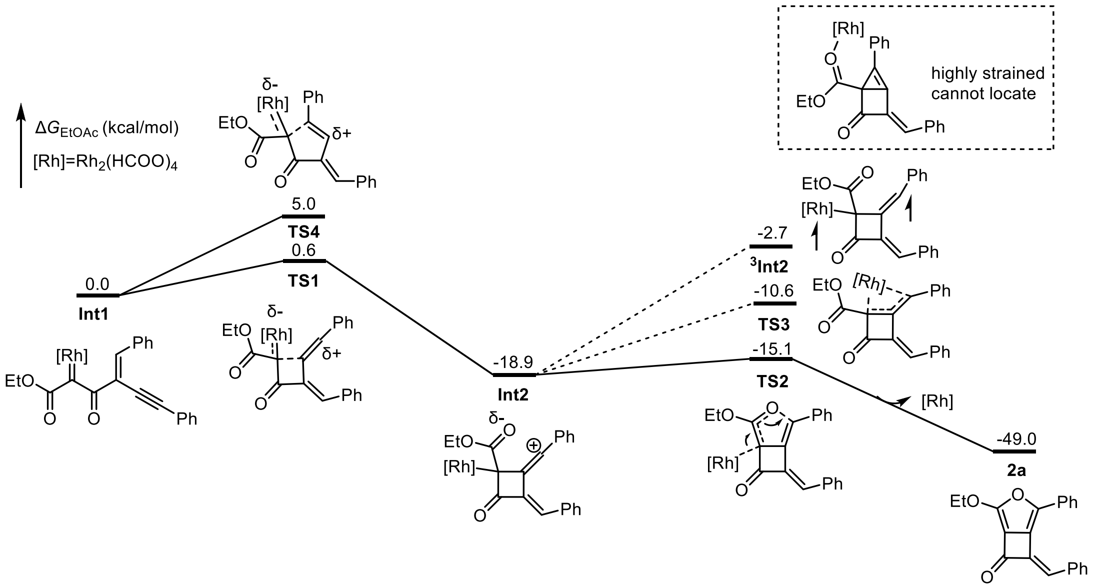

Supplementary Fig. 233 | Gibbs free energy surface of 4-*exo-dig* pathway. Computed at SMD(EtOAc)/BMK/def2-TZVPP//B3LYP/def2-SVP.

The Gibbs free energy surface of 5-*endo-dig* pathway using $Rh_2(HCOO)_4$ as catalyst is shown in **Supplementary Fig. 234**. The carbenoid **Int1** undergo 5-*endo-dig* cyclization to afford **Int3** with an activation free energy of 5.0 kcal/mol. Here we found both singlet and triplet transition states of 1,3-Rh migration. **Int3** could give **Int4** directly through a singlet diradical transition state **^{1}TS5** ($<S^2>$=0.0347), while a comparable ISC process via **MECP1** is also found, giving a more stable species triplet **^{3}Int3,** then 1,3-Rh migration *via* triplet transition state **^{3}TS5** afford the Rh carbenoid **^{3}Int4**. Subsequently, another ISC process occurs via **MECP2**, giving the more stable closed-shell complex **Int4**. Then the aromatic C-H insertion of Rh carbenoid proceeds through a stepwise process: The C-C bond formation *via* **TS6** requires an activation free energy of 6.6 kcal/mol, followed by a hydrogen migration via **TS7**, giving the correspond product **3a**.

Axial coordination

We have also considered about the effect of axial coordination[25] on the cyclization steps (**Supplementary Fig. 235**). Our DFT calculations found that the axial coordination has insignificant effect on the regioselectivity of cyclizations (ΔΔG = 0.8

kcal/mol vs. 1.2 kcal/mol for $Rh_2(OAc)_4$; ΔΔG = -0.5 kcal/mol vs. -0.1 kcal/mol for $Rh_2(tfacam)_4$, the changes in activation free energies of both cyclizations are also small (< 1.0 kcal/mol), thus the axial solvent might not play an important role in this case.

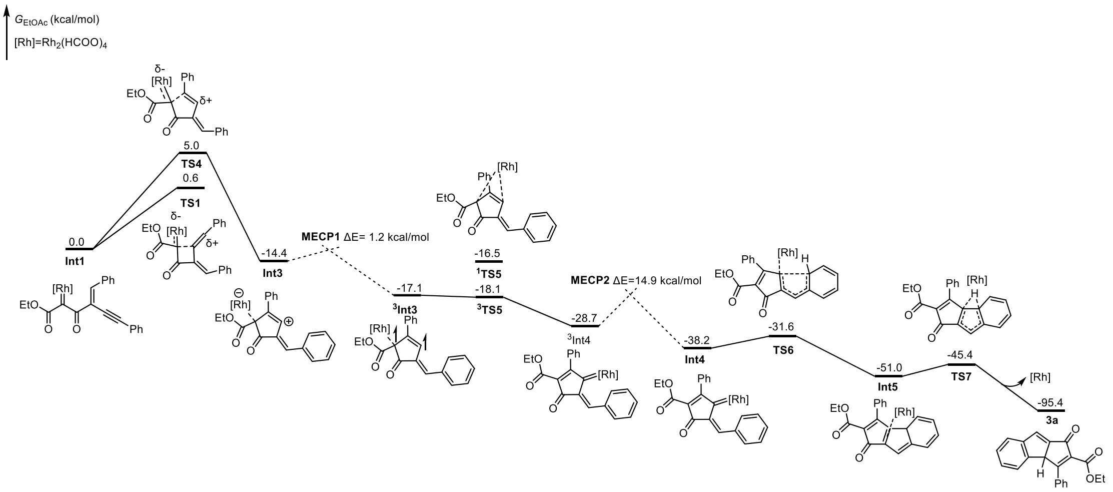

Supplementary Fig. 234 | Gibbs free energy surface of 5-*endo-dig* pathway. Computed at SMD(EtOAc)/BMK/def2-TZVPP//B3LYP/def2-SVP.

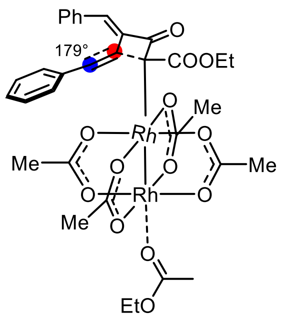

TS1-Me-EA_ligand ΔG = 5.0 (ΔΔG = 0.0)

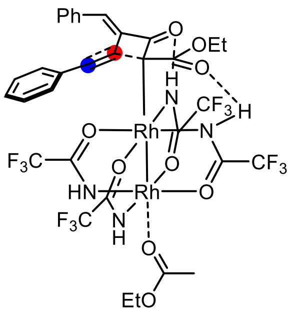

TS1-CF$_3$-EA_ligand ΔG = 2.8 (ΔΔG = 0.0)

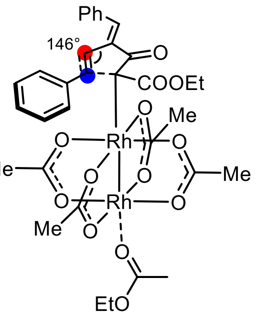

TS4-Me-EA_ligand ΔG = 5.8 (ΔΔG = 0.8)

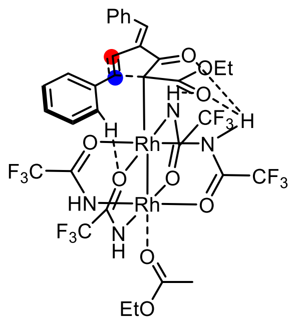

TS4-CF$_3$-EA_ligand ΔG = 2.3 (ΔΔG = -0.5)

Supplementary Fig. 235 | The key transition states in the regioselectivity of the real system with axial effect. Computed at SMD(EtOAc)/BMK/def2-TZVPP//SMD(EtOAc)/B3LYP/def2-SVP.

Competition between intramolecular cyclization and intermolecular O-H insertion of substrate 1af.

We have tried to use BnOH to capture the proposed cation **Int2**. When substrate **1af** was used, both the O-H insertion product **70** and intramolecular cyclization product **2af** were observed (**70**:**2af** = 1:1.3). Here we carried out DFT calculations to investigate the competition between intramolecular cyclization and intermolecular O-H insertion of substrate **1af** (using catalyst $Rh_2(OAc)_4$), which is shown in **Supplementary Fig. 236**. ωB97X-D, which accounts well for dispersion and intermolecular interactions is used[19]. According to our studies above, **Int2-CP** is obtained through 4-*exo-dig* cyclization from the substrate **1af** and $Rh_2(OAc)_4$. Then **Int2-CP** can undergo intramolecular cyclization *via* **TS2-CP**, which will deliver product **2af**, requiring activation free energy of 3.5 kcal/mol (path a). The 1,3 Rh-migration, which forms Rh-carbene *via* **TS3-CP**, is disfavored over **TS2-CP** by 6.7 kcal/mol (path b). When BnOH is added, the vinyl cation **Int2-CP** can undergo O-H insertion to afford **Int3-CP** *via* **TS4-CP**. This process is highly exothermic (-30.6 kcal/mol) and has comparable activation free energies with **TS2** (3.5 kcal/mol vs. 3.5 kcal/mol), which is consistent with our experiments. Then **Int3-CP** can afford the O-H insertion product through sequential proton transfer process. DFT calculations suggested that direct 1,5-proton transfer *via* **TS5-CP** is difficult, requiring activation free energy of 28.2 kcal/mol. However, with the assistant of an additional molecule of BnOH as a proton transporter[26], the proton transfer is facilitated. This process requires activation free energy of 22.4 kcal/mol *via* **TS6-CP**, affording the O-H insertion product **70**.

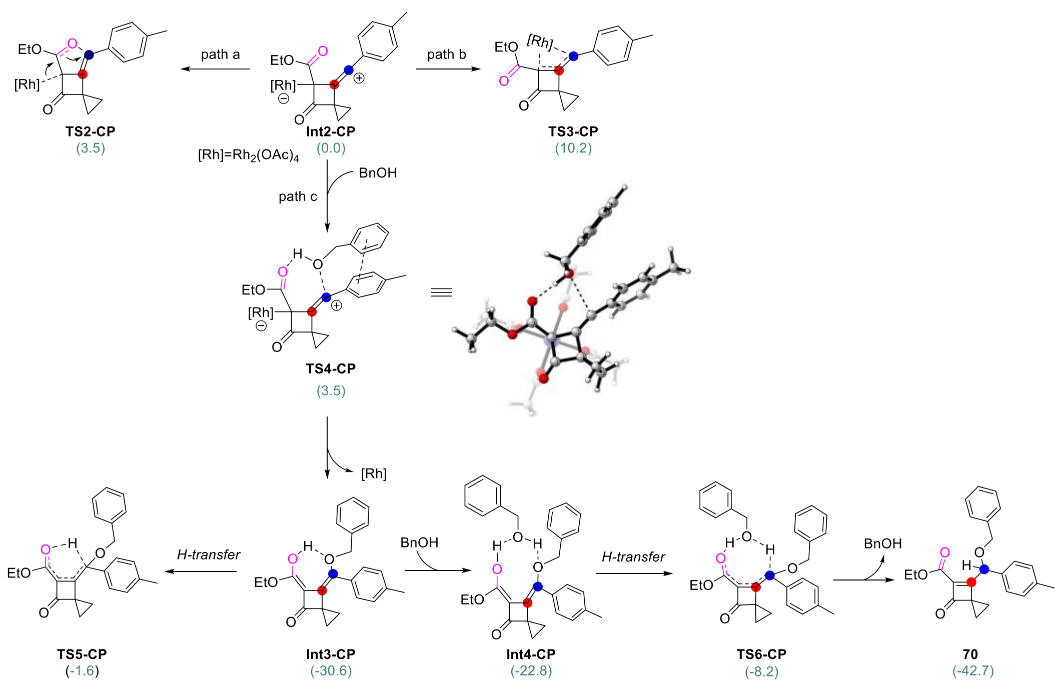

Supplementary Fig. 236 | Competition between intramolecular cyclization and intermolecular O-H insertion of substrate 1af in EtOAc. Computed at SMD(EtOAc)/ωB97X-D/def2-TZVPP//B3LYP/def2-SVP.

Supplementary Table 4 | Computed Energies for the Stationary Points.

Thermal corrections to Gibbs energies (TCGs), single-point energies (SPEs) in gas phase and solvent.

	Imaginary Frequencies (cm^{-1})	SPEs (in gas phase)[a] (hartree)	TCGs (in gas phase)[b] (hartree)	SPEs (under SMD model)[c] (hartree)
**$Rh_2(HCOO)_4$**	None	-977.421178	0.063739	-976.997184
**Int1-gas phase**	None	-2012.102984	0.348576	-2012.128718
**Int2**	None	-2012.106061	0.348893	-2012.159103
**^{3}Int2**	None	-2012.088106	0.346828	-2012.131219
**Int3**	None	-2012.105425	0.349887	-2012.152993
**^{3}Int3**	None	-2012.112157	0.349359	-2012.156829
**Int4**	None	-2012.155012	0.352458	-2012.193545
**^{3}Int4**	None	-2012.127162	0.34968	-2012.175521
**Int5**	None	-2012.158498	0.352998	-2012.21448
**2a**	None	-1034.706976	0.266731	-1035.194602
**3a**	None	-1034.777076	0.268341	-1035.270055
**TS1-gas phase**	-299.82	-2012.087742	0.347893	-2012.127144
**TS2**	-97.00	-2012.105249	0.350047	-2012.154182
**TS3**	-72.45	-2012.086513	0.348692	-2012.145783
**TS4-gas phase**	-223.23	-2012.079257	0.348711	-2012.120845
**^{1}TS5**	-16.20	-2012.105274	0.350174	-2012.157496
**^{3}TS5**	-30.89	-2012.108183	0.349623	-2012.158551
**TS6**	-279.67	-2012.133516	0.352096	-2012.182673
**TS7**	-955.69	-2012.143975	0.35003	-2012.202575
**MECP1**	None	-2012.103481		
**MECP2**	None	-2012.103483		
**1a**	None	-1144.108417	0.269328	-1144.64783
**Com1**	None	-2121.543118	0.356089	-2121.650396
**Com1-TS**	-251.12	-2121.520422	0.353801	-2121.617266
	**Imaginary**	**SPEs[d]**	**TCGs[e]**	**SPEs[f]**

	**Frequencies (cm^{-1})**	**(hartree)**	**(hartree)**	**(under SMD model) (hartree)**
**Int1-Me**	None	-2169.332208	0.448756	-2169.367475
**Int1-CF_3**	None	-3279.861200	0.403315	-3280.851868
**Int1-solution phase**	None	-2012.138636	0.348087	-2012.128452
**TS1-solution phase**	-279.43	-2012.127084	0.348185	-2012.124025
**TS4-solution phase**	-210.76	-2012.115149	0.348307	-2012.12201
**TS1-Me**	-332.68	-2169.316757	0.448727	-2169.360394
**TS4-Me**	-225.85	-2169.306427	0.449016	-2169.358695
**TS1-CF_3**	-283.33	-3279.853619	0.403709	-3280.849644
**TS4-CF_3**	-218.15	-3279.841843	0.404157	-3280.850264
**Int1-Me-EA_ligand**	None	-2476.828137	0.557467	-2477.027769
**TS1-Me-EA_ligand**	-347.81	-2476.811465	0.557175	-2477.01957
**TS4-Me-EA_ligand**	-249.33	-2476.802974	0.557722	-2477.018839
**Int1-CF_3-EA_ligand**	None	-3587.361013	0.512837	-3588.515612
**TS1-CF_3-EA_ligand**	-299.97	-3587.351478	0.512933	-3588.511241
**TS4-CF_3-EA_ligand**	-243.00	-3587.342051	0.513523	-3588.512642
	**Imaginary Frequencies (cm^{-1})**	**SPEs[a] (hartree)**	**TCGs[b] (hartree)**	**SPEs[g] (under SMD model) (hartree)**
**Int2-CP**	None	-2016.961296	0.424817	-2018.4867
**Int3-CP**	None	-1228.938822	0.369434	-1229.914122
**Int4-CP**	None	-1575.468221	0.49353	-1576.722838
**BnOH**	None	-346.523587	0.101281	-346.801364
**$Rh_2(OAc)_4$**	None	-1134.610555	0.162164	-1135.428208
**TS2-CP**	-106.48	-2016.960303	0.426057	-2018.482401
**TS3-CP**	-58.29	-2016.947946	0.424526	-2018.470221
**TS4-CP**	-36.82	-2363.505364	0.552165	-2365.305583
**TS5-CP**	-1622.01	-1228.88823	0.363531	-1229.862033
**TS6-CP**	-1208.35	-1575.445615	0.489236	-1576.695359
**70**	None	-1228.949232	0.366797	-1229.930747

[a]Computed at B3LYP/def2-SVP.

[b]Computed at B3LYP/def2-SVP after quasiharmonic corrections..

[c]Computed at SMD(EtOAc)/BMK/def2-TZVPP//B3LYP/def2-SVP.

[d]Computed at SMD(EtOAc)/B3LYP/def2-SVP.

[e]Computed at SMD(EtOAc)/B3LYP/def2-SVP after quasiharmonic corrections.

[f]Computed at SMD(EtOAc)/BMK/def2-TZVPP//SMD(EtOAc)/B3LYP/def2-SVP.

[g]Computed at SMD(EtOAc)/ωB97X-D/def2-TZVPP//B3LYP/def2-SVP.

Supplementary References

1. Kardile, R. D., Chao, T.-H., Cheng, M.-J. & Liu, R.-S. Gold(I)-catalyzed highly diastereo- and enantioselective cyclization–[4+3] annulation cascades between 2-(1-alkynyl)-2-alken-1-ones and anthranils. *Angew. Chem. Int. Ed.* **59**, 10396 – 10400 (2020).

2. Frisch, M. J. et al. Gaussian 09, Revision E.01; Gaussian, Inc.: Wallingford, CT, 2013.

3. (a) Becke, A. D. Density‐functional thermochemistry. III. The role of exact exchange. *J. Chem. Phys.* **98**, 5648−5652 (1993).

4. Lee, C., Yang, W. & Parr, R. G. Development of the Colle-Salvetti correlation-energy formula into a functional of the electron density. *Phys. Rev. B.* **37**, 785−789 (1988).

5. Weigend, F. & Ahlrichs, R. Balanced basis sets of split valence, triple zeta valence and quadruple zeta valence quality for H to Rn: Design and assessment of accuracy. *Phys. Chem. Chem. Phys.* **7**, 3297−3305 (2005).

6. Marenich, A. V., Cramer, C. J. & Truhlar, D. G. Universal solvation model based on solute electron density and a continuum model of the solvent defined by the bulk dielectric constant and atomic surface tensions. *J. Phys. Chem. B,* **113**, 6378−6396 (2009).

7. Lu, T. & Chen, Q. Shermo: A general code for calculating molecular thermochemistry properties. *Comput. Theor. Chem.* **1200**, 113249 (2021).

8. Boese, A. D. & Martin, J. M. L. Development of density functionals for thermochemical kinetics. *J. Chem. Phys.* **121**, 3405−3416 (2004).

9. Legault, C. Y. CYLview, 1.0b; Universitéde Sherbrooke, (2009). http://www.cylview.org.

10. Lu, T. & Chen, F. Multiwfn: A multifunctional wavefunction analyzer. *J. Comput. Chem.* **33**, 580−592 (2012).

11. Riplinger, C. & Neese, F. An efficient and near linear scaling pair natural orbital based local coupled cluster method. *J. Chem. Phys.* **138**, 034106 (2013).

12. Riplinger, C., Sandhoefer, B., Hansen, A. & Neese, F. Natural triple excitations in local coupled cluster calculations with pair natural orbitals. *J. Chem. Phys.* **139**, 134101 (2013).
13. Neese, F., Atanasov, M., Bistoni, G., Maganas, D. & Ye, S. Chemistry and quantum mechanics in 2019: Give us insight and numbers. *J. Am. Chem. Soc.* **141**, 2814−2824 (2019).
14. Sperger, T., Sanhueza, I. A., Kalvet, I. & Schoenebeck, F. Computational studies of synthetically relevant homogeneous organometallic catalysis involving Ni, Pd, Ir, and Rh: An overview of commonly employed DFT methods and mechanistic insights. *Chem. Rev.* **115**, 9532-9586 (2015).
15. Neese, F. The ORCA program system. *Wiley Interdiscip. Rev.: Comput. Mol. Sci.* **2**, 73−78 (2012).
16. Boese, A. D. & Martin, J. M. L. Development of density functionals for thermochemical kinetics. *J. Chem. Phys.* **121**, 3405−3416 (2004).
17. Zhao, Y. & Truhlar, D. G. The M06 suite of density functionals for main group thermochemistry, thermochemical kinetics, noncovalent interactions, excited states, and transition elements: Two new functionals and systematic testing of four M06-class functionals and 12 other functionals. *Theor. Chem. Acc.* **120**, 215−241 (2008).
18. Zhao, Y. & Truhlar, D. G. A new local density functional for main-group thermochemistry, transition metal bonding, thermochemical kinetics, and noncovalent interactions. *J. Chem. Phys.* **125**, 194101 (2006).
19. Chai, J.-D. & Head-Gordon, M. Long-range corrected hybrid density functionals with damped atom−atom dispersion corrections. *Phys. Chem. Chem. Phys.* **10**, 6615−6620 (2008).
20. Adamo, C. & Barone, V. Toward reliable density functional methods without adjustable parameters: The PBE0 model. *J. Chem. Phys.* **110**, 6158−6170 (1999).

21. Grimme, S., Antony, J., Ehrlich, S. & Krieg, H. A consistent and accurate Ab initio parametrization of density functional dispersion correction (DFT-D) for the 94 elements H−Pu. *J. Chem. Phys.* **132**, 154104 (2010).
22. Becke, A. D. Density-functional exchange-energy approximation with correct asymptotic behavior. *Phys. Rev. A* **38**, 3098−3100 (1988).
23. Perdew, J. P. Density-functional approximation for the correlation energy of the inhomogeneous electron gas. *Phys. Rev. B* **33**, 8822−8824 (1986).
24. Wang, Y., Liao, W., Wang, Y., Jiao, L. & Yu, Z.-X. Mechanism and stereochemistry of rhodium-catalyzed [5 + 2 + 1] cycloaddition of Ene–vinylcyclopropanes and carbon monoxide revealed by visual kinetic analysis and quantum chemical calculations. *J. Am. Chem. Soc.* **144**, 2624-2636 (2022).
25. Laconsay, C. J., Pla-Quintana, A. & Tantillo, D. J. Effects of axial solvent coordination to dirhodium complexes on the reactivity and selectivity in C–H insertion reactions: A computational study. *Organometallics.* **40**, 4120-4132 (2021).
26. Liang, Y., Zhou, H. & Yu, Z.-X. Why is copper(I) complex more competent than dirhodium(II) complex in catalytic asymmetric O−H insertion reactions? A computational study of the metal carbenoid O−H insertion into water. *J. Am. Chem. Soc.* **131**, 17783–17785 (2009).
