# Supplementary material for: Catalytic 4-exo-dig carbocyclization for the construction of furan-fused cyclobutanones and synthetic applications
Source: Nat Commun. 2023 Oct 11;14:6378. doi: 10.1038/s41467-023-42032-9 (PMC10567718; doi:10.1038/s41467-023-42032-9)
Supplement: Supplementary file 3 — Description of Additional Supplementary Files [file 41467_2023_42032_MOESM3_ESM.docx]

**Description of Additional Supplementary Files**

**File Name: Supplementary Data 1**

**Description:** Cartesian coordinates for the stationary points.
