# Supplementary material for: Catalytic 4-exo-dig carbocyclization for the construction of furan-fused cyclobutanones and synthetic applications
Source: Nat Commun. 2023 Oct 11;14:6378. doi: 10.1038/s41467-023-42032-9 (PMC10567718; doi:10.1038/s41467-023-42032-9)
Supplement: Supplementary file 4 — Supplementary Data 1 [file 41467_2023_42032_MOESM4_ESM.docx]

Cartesian coordinates for the stationary points

**2a**

C 4.067640 0.480629 0.945569

C 5.291497 -0.013175 0.481466

C 5.336338 -1.251793 -0.168518

C 4.165817 -1.985822 -0.355497

C 2.919318 -1.490855 0.084060

C 2.893700 -0.248208 0.750649

H 4.026350 1.438522 1.470186

H 6.209415 0.559545 0.636400

H 6.290050 -1.649432 -0.524910

H 4.205983 -2.957093 -0.856099

H 1.950271 0.130967 1.145989

C 1.718225 -2.302099 -0.115301

C 0.417765 -1.945958 -0.146091

H 1.884519 -3.378785 -0.245273

C -0.500400 -0.778512 -0.087125

C -0.919036 0.526427 -0.055542

C -0.327524 1.853855 -0.149262

C 0.932248 2.034738 -0.755852

C -1.007719 2.988858 0.340357

C 1.503024 3.305033 -0.842640

H 1.452875 1.176199 -1.182034

C -0.435481 4.256510 0.241457

H -1.987071 2.866136 0.805894

C 0.824537 4.421825 -0.344430

H 2.480491 3.423061 -1.317221

H -0.975905 5.123856 0.629583

H 1.270650 5.416572 -0.419704

C -0.820618 -2.857860 -0.175785

C -1.667760 -1.626075 -0.056514

C -2.754992 -0.795289 0.033736

O -2.324896 0.488722 0.040772

O -4.050069 -1.029686 0.109351

O -0.981587 -4.044210 -0.258869

C -4.967316 0.079376 0.155433

H -4.750239 0.685893 1.050647

H -4.802815 0.718496 -0.727911

C -6.371083 -0.484909 0.188608

H -6.516354 -1.123088 1.073284

H -7.101883 0.337400 0.228532

H -6.572999 -1.087040 -0.710073

**^3^Int2**

C 5.988371 -1.073765 -0.494665

C 6.738161 -2.220359 -0.205462

C 6.261756 -3.138754 0.735605

C 5.046136 -2.910745 1.378984

C 4.271479 -1.764391 1.094344

C 4.771281 -0.846744 0.145197

H 6.359304 -0.347865 -1.222772

H 7.692466 -2.394078 -0.709071

H 6.841894 -4.034640 0.970524

H 4.678392 -3.629877 2.115881

H 4.200357 0.055520 -0.082376

C 3.005778 -1.597382 1.801098

C 2.021748 -0.676229 1.702725

H 2.800444 -2.358577 2.564033

C 1.624705 0.562849 0.966366

C 2.243669 1.299574 0.008304

C 2.394116 2.395238 -0.857536

C 2.987837 2.197882 -2.138987

C 2.016389 3.716690 -0.478275

C 3.183475 3.270633 -2.999180

H 3.278553 1.188226 -2.436265

C 2.236990 4.779385 -1.348093

H 1.529130 3.863540 0.485436

C 2.816340 4.566780 -2.606491

H 3.631468 3.103611 -3.981930

H 1.943094 5.788066 -1.046938

H 2.980579 5.409248 -3.283019

C 0.759814 -0.556964 2.519835

C 0.360431 0.659550 1.693182

C -0.355534 1.887181 2.119741

O -0.065723 3.012041 1.768590

O -1.329734 1.603751 2.986233

O 0.321379 -1.141153 3.471453

C -2.109893 2.707366 3.471314

H -1.439471 3.429695 3.965413

H -2.561647 3.222226 2.608033

C -3.153054 2.157641 4.421230

H -2.678671 1.635443 5.265974

H -3.768437 2.979132 4.820243

H -3.814984 1.444721 3.906526

Rh -1.029109 -0.290752 -0.044116

Rh -2.707855 -1.236290 -1.533772

O -2.287223 1.344992 0.127037

O -0.176191 0.573651 -1.716614

O 0.077909 -1.998569 -0.352819

O -2.040188 -1.240873 1.477365

O -3.601729 -2.110411 0.090379

O -1.484147 -2.877013 -1.733144

O -3.840374 0.453829 -1.255988

O -1.736041 -0.311811 -3.093735

C -0.713083 0.377111 -2.842248

C -0.390441 -2.894296 -1.110415

C -3.068637 -1.920627 1.217707

C -3.381105 1.351472 -0.502285

H -3.559688 -2.407074 2.084445

H -4.009704 2.256131 -0.374484

H 0.239079 -3.797637 -1.240099

H -0.222641 0.868997 -3.706643

**^3^Int3**

C -6.351955 -0.555122 1.406359

C -7.310699 -1.387596 0.811888

C -7.075387 -1.923709 -0.459046

C -5.890818 -1.628803 -1.130291

C -4.912417 -0.788059 -0.548609

C -5.168387 -0.257057 0.738106

H -6.533007 -0.138476 2.400284

H -8.238832 -1.617902 1.340985

H -7.818965 -2.573999 -0.926183

H -5.706368 -2.048037 -2.122884

H -4.423913 0.388077 1.209825

C -3.700712 -0.519022 -1.293772

C -2.641494 0.287691 -0.961170

H -3.600111 -1.015070 -2.265400

C -2.315098 1.112009 0.112249

C -1.065143 1.772047 0.009050

C -0.626986 2.735617 1.025411

C 0.727527 3.038763 1.272934

C -1.612229 3.362890 1.818655

C 1.074289 3.948297 2.269930

H 1.506243 2.539235 0.700548

C -1.262262 4.283793 2.804279

H -2.665307 3.132510 1.640342

C 0.085140 4.580055 3.033231

H 2.128998 4.164143 2.458506

H -2.041428 4.769089 3.397020

H 0.365135 5.296665 3.809748

C -1.433575 0.441594 -1.910566

C -0.436798 1.320312 -1.203979

C 0.578211 2.039409 -2.036263

O 1.110431 1.576644 -3.013233

O 0.781926 3.306585 -1.617667

O -1.391012 -0.029592 -3.018164

C 1.730595 4.081282 -2.369573

H 2.722440 3.607376 -2.282080

H 1.453939 4.049161 -3.435286

C 1.724787 5.494290 -1.823368

H 2.007195 5.512529 -0.759664

H 2.442291 6.114654 -2.383029

H 0.726815 5.949061 -1.919992

Rh 0.913483 -0.561043 -0.118573

Rh 2.362750 -2.340224 0.666310

O 2.597799 0.634225 -0.221535

O 0.664235 -0.028084 1.867038

O -0.655735 -1.880325 0.060640

O 1.263585 -1.278847 -2.009194

O 2.628329 -2.926415 -1.276486

O 0.696495 -3.536655 0.800306

O 3.946605 -1.040032 0.481246

O 2.035656 -1.675466 2.586148

C 1.269789 -0.690791 2.754310

C -0.417070 -3.051034 0.471036

C 2.021274 -2.270636 -2.169156

C 3.716231 0.131058 0.085384

H 2.170314 -2.604970 -3.215245

H 4.593339 0.803765 -0.003119

H -1.293340 -3.725700 0.550574

H 1.106684 -0.365073 3.801879

**^3^Int4**

C -2.284338 3.643903 2.399081

C -2.765558 4.903806 2.017406

C -2.176123 5.575683 0.940762

C -1.112972 4.990792 0.255253

C -0.629577 3.711020 0.611685

C -1.234689 3.049139 1.704799

H -2.733706 3.123607 3.248826

H -3.591364 5.364475 2.565743

H -2.539806 6.562537 0.643867

H -0.641426 5.522778 -0.575376

H -0.871538 2.067298 2.010430

C 0.516646 3.185123 -0.107725

C 1.118588 1.956238 -0.086740

H 1.066872 3.918824 -0.709961

C 0.852196 0.675257 0.496297

C 2.103060 -0.049430 0.668130

C 2.236682 -1.272329 1.480659

C 3.109900 -2.319019 1.132528

C 1.468239 -1.398862 2.653036

C 3.208181 -3.456883 1.931055

H 3.698818 -2.254062 0.217550

C 1.581419 -2.531030 3.461801

H 0.779679 -0.598212 2.927669

C 2.449331 -3.565866 3.102559

H 3.886185 -4.263590 1.641105

H 0.987177 -2.606049 4.376153

H 2.536392 -4.454970 3.732492

C 2.562384 1.868368 -0.601449

C 3.121221 0.631918 -0.004879

C 4.576811 0.336086 0.005079

O 5.189915 -0.127416 0.940951

O 5.147762 0.672578 -1.161837

O 3.118895 2.729503 -1.247196

C 6.574976 0.539753 -1.252219

H 7.038768 1.109445 -0.430505

H 6.848459 -0.517932 -1.101191

C 7.002082 1.051738 -2.612159

H 6.706528 2.103910 -2.741307

H 8.095952 0.978862 -2.716410

H 6.537042 0.463923 -3.418562

Rh -0.867897 -0.221251 -0.292513

Rh -2.828326 -1.648664 -0.859990

O 0.129045 -2.031707 -0.200553

O -1.580691 -0.397877 1.662228

O -2.100017 1.398344 -0.584616

O -0.393896 -0.174384 -2.282276

O -2.166301 -1.482360 -2.804923

O -3.884040 0.099839 -1.075520

O -1.678465 -3.327978 -0.619309

O -3.349142 -1.712230 1.126484

C -2.614576 -1.074794 1.925506

C -3.306587 1.205378 -0.902053

C -1.137338 -0.811838 -3.082732

C -0.459966 -3.134944 -0.363402

H -0.844400 -0.770036 -4.150423

H 0.176630 -4.037214 -0.269071

H -3.921995 2.115757 -1.043787

H -2.908697 -1.108907 2.994235

**3a**

C 3.438101 0.495526 0.265537

C 4.757610 0.212320 -0.121956

C 5.143746 -1.088491 -0.467819

C 4.216828 -2.135881 -0.443663

C 2.896397 -1.858319 -0.072458

C 2.504040 -0.535635 0.277045

H 3.155948 1.515728 0.531663

H 5.491865 1.021080 -0.158168

H 6.177596 -1.285395 -0.762564

H 4.516592 -3.151341 -0.715096

C 1.720306 -2.731075 -0.026809

C 0.657953 -2.007708 0.401451

H 1.683506 -3.753178 -0.408546

C 1.051212 -0.593456 0.725795

C -0.116014 0.219448 0.157564

C -0.068793 1.691833 0.104034

C -0.659874 2.419539 -0.949275

C 0.596464 2.407876 1.122901

C -0.587105 3.813619 -0.973942

H -1.176102 1.882211 -1.744126

C 0.650935 3.801284 1.101850

H 1.055722 1.870340 1.955243

C 0.062470 4.510031 0.049061

H -1.044131 4.359560 -1.803091

H 1.155837 4.336372 1.910091

H 0.112410 5.601644 0.026836

C -0.788256 -2.069130 0.093176

C -1.160028 -0.620597 -0.120046

C -2.541393 -0.271624 -0.566653

O -2.824850 0.376165 -1.550233

O -3.449232 -0.805511 0.256138

O -1.498549 -3.038778 -0.067608

C -4.833649 -0.663812 -0.109787

H -5.090969 0.408161 -0.122747

H -4.967218 -1.040858 -1.136323

C -5.659793 -1.438864 0.894836

H -5.515605 -1.044687 1.912640

H -6.728791 -1.361576 0.642091

H -5.375793 -2.502074 0.893401

H 1.043240 -0.452284 1.827265

**Int1-CF_3_**

C -5.453462 3.942606 0.670030

C -4.767591 5.158307 0.548846

C -3.368921 5.178887 0.597631

C -2.662537 3.990564 0.766888

C -3.338447 2.751304 0.882397

C -4.752246 2.750120 0.832827

H -6.545854 3.927005 0.638004

H -5.324781 6.089841 0.419009

H -2.829597 6.124850 0.505686

H -1.569914 4.005155 0.805292

H -5.298053 1.812751 0.931558

C -2.519551 1.566261 1.050108

C -2.835301 0.228065 1.061885

H -1.455231 1.791348 1.164805

C -4.109120 -0.373843 0.872257

C -5.152007 -0.987971 0.708032

C -6.367620 -1.712600 0.516799

C -7.556038 -1.050620 0.135889

C -6.393438 -3.112908 0.705639

C -8.734711 -1.773183 -0.047373

H -7.543683 0.030483 -0.018386

C -7.577592 -3.825146 0.519283

H -5.476231 -3.629174 0.997402

C -8.750527 -3.159789 0.143486

H -9.648108 -1.250514 -0.342910

H -7.585603 -4.908022 0.668050

H -9.676588 -3.721950 -0.002142

C -1.721327 -0.719248 1.249734

C -0.336774 -0.292392 1.552259

C 0.005621 -0.101611 2.984293

O -0.245846 0.960383 3.509579

O 0.617986 -1.134369 3.547914

O -1.796847 -1.945493 1.167645

C 0.972977 -1.019564 4.962173

H 1.654436 -0.162470 5.073027

H 0.053240 -0.802882 5.525919

C 1.612332 -2.318805 5.389057

H 2.535018 -2.518002 4.822278

H 1.874316 -2.255376 6.456887

H 0.922394 -3.166201 5.254057

Rh 1.001637 -0.116607 0.122172

Rh 2.686546 0.036076 -1.732921

O -0.415297 0.649360 -1.214644

O 1.531290 1.839708 0.637784

O 4.146479 -0.651936 -0.392967

O 2.243835 -1.946494 -2.241168

C -0.047690 0.828607 -2.420363

C 2.396265 2.434315 -0.082268

C 3.754631 -0.939242 0.770948

C 1.310956 -2.496447 -1.592797

C 4.865743 -1.472033 1.696792

C 0.978619 -3.929168 -2.051215

C -1.187007 1.292968 -3.342932

C 2.666908 3.880743 0.362339

N 2.542124 -0.825508 1.241947

N 0.635740 -1.976946 -0.605252

N 1.147719 0.655939 -2.897291

N 3.022144 1.936169 -1.104159

H 3.687884 2.559178 -1.560931

H 1.253558 0.835551 -3.895551

H -0.107181 -2.539258 -0.188861

H 2.392356 -1.141477 2.198050

F 5.400160 -2.589331 1.192916

F 4.410603 -1.754417 2.930710

F 5.841052 -0.564452 1.820727

F 3.113747 3.912400 1.623292

F 3.580472 4.482891 -0.414361

F 1.540091 4.605977 0.305784

F 2.051476 -4.718392 -1.917170

F -0.022727 -4.471391 -1.341813

F 0.619617 -3.934687 -3.340630

F -2.164340 0.378884 -3.377719

F -0.765462 1.491996 -4.602266

F -1.711393 2.442427 -2.899830

**Int1-Me**

C -4.848690 4.018691 0.372655

C -4.207773 5.175428 0.834264

C -2.888686 5.102048 1.295850

C -2.219076 3.880159 1.295000

C -2.852430 2.698417 0.840183

C -4.184838 2.793457 0.374891

H -5.877105 4.074213 0.006627

H -4.736093 6.132434 0.831729

H -2.381681 6.000605 1.656388

H -1.188828 3.823918 1.655166

H -4.696105 1.904796 0.008007

C -2.077729 1.468526 0.885406

C -2.422158 0.168210 0.612004

H -1.039866 1.618962 1.200505

C -3.687258 -0.333192 0.200124

C -4.731425 -0.867285 -0.141176

C -5.947611 -1.499910 -0.541203

C -5.953403 -2.873617 -0.873180

C -7.157549 -0.773795 -0.609606

C -7.138042 -3.497251 -1.262976

H -5.020387 -3.438887 -0.820195

C -8.336541 -1.407905 -1.000599

H -7.163395 0.287271 -0.350918

C -8.331707 -2.768766 -1.328356

H -7.130415 -4.560291 -1.517320

H -9.266930 -0.836195 -1.048917

H -9.258213 -3.261889 -1.633770

C -1.381164 -0.871754 0.795597

C 0.001370 -0.572607 1.236395

C 0.263692 -0.622547 2.695730

O 0.181969 0.407787 3.332620

O 0.579963 -1.814989 3.162387

O -1.541702 -2.071049 0.607043

C 0.888917 -1.921903 4.579356

H 1.754042 -1.274729 4.791231

H 0.029953 -1.541051 5.153230

C 1.176849 -3.372984 4.884136

H 2.037477 -3.738634 4.302514

H 1.413105 -3.480315 5.954555

H 0.305429 -4.007201 4.658089

Rh 1.482299 -0.203473 -0.022314

Rh 3.319459 0.264273 -1.573080

O 1.899694 -2.191581 -0.361113

O 0.225852 -0.190409 -1.652514

O 1.285888 1.848150 0.126415

O 2.871984 -0.180475 1.497679

O 4.567642 0.260569 0.077679

O 2.971127 2.272942 -1.303873

O 3.590363 -1.764306 -1.784602

O 1.916101 0.229172 -3.086227

C 0.699367 0.020295 -2.817906

C 2.046635 2.636585 -0.526674

C 4.099493 0.037757 1.228976

C 2.831597 -2.550264 -1.151606

C 5.060557 -0.010798 2.389376

H 4.599186 0.417758 3.290030

H 5.300705 -1.066312 2.601226

H 5.991101 0.517763 2.144802

C 1.798567 4.111240 -0.342689

H 0.781548 4.355056 -0.688065

H 1.854425 4.364178 0.727328

H 2.531377 4.702646 -0.905636

C -0.295741 0.024779 -3.949905

H -0.879741 -0.908054 -3.932998

H -1.003285 0.856894 -3.805431

H 0.210790 0.135528 -4.916764

C 3.006732 -4.033111 -1.352367

H 2.924037 -4.560384 -0.391209

H 2.195258 -4.398648 -2.003317

H 3.971738 -4.249370 -1.828309

**Int1-gas phase**

C -4.401468 4.080345 0.926172

C -3.677894 5.115576 1.530719

C -2.361769 4.891389 1.948980

C -1.777287 3.640989 1.762142

C -2.494137 2.581050 1.157995

C -3.822479 2.826687 0.740928

H -5.428310 4.254872 0.595144

H -4.139463 6.095999 1.673522

H -1.790975 5.695093 2.420503

H -0.748326 3.463314 2.085010

H -4.393763 2.030255 0.266227

C -1.799966 1.313463 1.002779

C -2.218521 0.105989 0.503553

H -0.760169 1.343189 1.343257

C -3.502744 -0.244394 0.008366

C -4.566926 -0.664796 -0.417228

C -5.801109 -1.177525 -0.917152

C -5.845758 -2.465483 -1.496015

C -6.991351 -0.421847 -0.840923

C -7.047916 -2.975830 -1.983236

H -4.926449 -3.051748 -1.553629

C -8.188584 -0.941490 -1.331099

H -6.966190 0.572659 -0.390534

C -8.221768 -2.217936 -1.903375

H -7.070146 -3.973605 -2.428604

H -9.103408 -0.347173 -1.265399

H -9.162257 -2.622303 -2.285894

C -1.232615 -0.997576 0.492986

C 0.152365 -0.874073 0.999152

C 0.398004 -1.257853 2.409504

O 0.325365 -0.384533 3.247429

O 0.706128 -2.524948 2.610777

O -1.418367 -2.129472 0.068826

C 1.036779 -2.916254 3.965161

H 1.913947 -2.332010 4.284363

H 0.197150 -2.644954 4.624246

C 1.307437 -4.404512 3.963184

H 2.146441 -4.650779 3.295104

H 1.564214 -4.736442 4.981162

H 0.421908 -4.965439 3.628046

Rh 1.670475 -0.253095 -0.108334

Rh 3.565833 0.554138 -1.462479

O 2.012205 -2.084961 -0.980423

O 0.443838 0.264962 -1.678417

O 1.540524 1.688503 0.601358

O 3.037129 -0.708092 1.369852

O 4.782069 0.028023 0.125017

O 3.292960 2.417547 -0.629272

O 3.748633 -1.343616 -2.228891

O 2.182837 1.006796 -2.928558

C 0.969695 0.766722 -2.717210

C 2.360762 2.565311 0.192998

C 4.263151 -0.467088 1.153238

C 2.946430 -2.219158 -1.824237

H 4.951093 -0.731201 1.982570

H 3.056883 -3.238139 -2.247554

H 2.223991 3.578433 0.623268

H 0.259402 1.021147 -3.530126

**Int2**

C 5.517958 -1.965799 -0.557394

C 6.512266 -2.619914 0.179515

C 6.295538 -2.907741 1.530926

C 5.097040 -2.537764 2.140711

C 4.094548 -1.855859 1.419320

C 4.322735 -1.588390 0.053218

H 5.672909 -1.757922 -1.619195

H 7.448240 -2.914248 -0.301794

H 7.062388 -3.427568 2.110571

H 4.928832 -2.769272 3.195853

H 3.540402 -1.114839 -0.541316

C 2.863875 -1.477607 2.112147

C 1.914915 -0.578763 1.790492

H 2.674390 -1.986146 3.065401

C 1.677449 0.479624 0.753650

C 2.305402 1.144311 -0.156071

C 2.931263 1.806577 -1.205584

C 2.768130 1.346598 -2.543916

C 3.734434 2.955285 -0.953602

C 3.410539 2.002391 -3.585415

H 2.119825 0.486869 -2.716347

C 4.357437 3.610209 -2.006922

H 3.831047 3.310916 0.073247

C 4.200077 3.130933 -3.316512

H 3.291680 1.651629 -4.612905

H 4.967485 4.496343 -1.819321

H 4.695230 3.649662 -4.141806

C 0.658420 -0.123946 2.501547

C 0.330326 0.814756 1.366169

C -0.009580 2.251884 1.410216

O 0.509807 3.074534 0.674362

O -0.919867 2.546023 2.339718

O 0.190627 -0.390123 3.574461

C -1.397280 3.899286 2.378163

H -0.545237 4.577070 2.552372

H -1.818492 4.148438 1.391290

C -2.432352 3.994488 3.479342

H -1.996352 3.726134 4.453837

H -2.820868 5.023190 3.543018

H -3.274974 3.314479 3.282644

Rh -1.269303 -0.193941 0.110372

Rh -3.039595 -1.263167 -1.209924

O -2.341690 1.567796 -0.097876

O -0.298465 0.189060 -1.695016

O -0.346394 -2.043228 0.188591

O -2.378629 -0.668628 1.771433

O -4.004583 -1.675209 0.557517

O -1.981100 -3.026110 -1.032919

O -3.980896 0.566762 -1.298271

O -1.960019 -0.770310 -2.902044

C -0.869281 -0.166380 -2.766869

C -0.904518 -3.021716 -0.385022

C -3.466994 -1.290306 1.629453

C -3.430425 1.548027 -0.737478

H -4.011848 -1.523315 2.567352

H -3.962956 2.519251 -0.811529

H -0.371803 -3.992347 -0.305253

H -0.331380 0.085947 -3.706429

**Int3**

C 6.162258 -0.513764 -1.298811

C 7.064725 -1.426059 -0.737052

C 6.765612 -2.045763 0.482561

C 5.574246 -1.748660 1.138865

C 4.658370 -0.816519 0.595297

C 4.964895 -0.221180 -0.651031

H 6.387959 -0.044211 -2.259355

H 7.994836 -1.666101 -1.258512

H 7.465350 -2.762315 0.919431

H 5.344391 -2.221904 2.097136

H 4.246107 0.465267 -1.103430

C 3.474490 -0.485242 1.358054

C 2.553434 0.492508 1.106010

H 3.258692 -1.105332 2.235300

C 2.483582 1.590128 0.232909

C 1.131401 2.029914 0.097311

C 0.684942 2.804493 -1.060779

C -0.675183 2.932802 -1.412826

C 1.657212 3.427164 -1.874536

C -1.043397 3.661800 -2.540414

H -1.438268 2.436435 -0.815609

C 1.281708 4.155533 -3.003005

H 2.706230 3.351445 -1.583664

C -0.069622 4.276960 -3.338931

H -2.100081 3.748283 -2.806256

H 2.045950 4.639323 -3.616352

H -0.366888 4.851974 -4.219906

C 1.320444 0.683310 2.011449

C 0.445268 1.649885 1.287631

C -0.671558 2.331901 1.990123

O -1.329352 1.840824 2.874563

O -0.835515 3.598638 1.554078

O 1.235171 0.237701 3.128754

C -1.880882 4.361055 2.178972

H -2.846367 3.863955 1.987319

H -1.727848 4.351266 3.270081

C -1.841093 5.764993 1.611517

H -1.999136 5.757546 0.522157

H -2.630989 6.378408 2.072603

H -0.870249 6.243133 1.813571

Rh -0.877886 -0.630327 0.172582

Rh -2.218120 -2.443323 -0.677905

O -2.553980 0.562336 0.013927

O -0.368749 -0.202256 -1.780996

O 0.696243 -1.955560 0.260117

O -1.456325 -1.218522 2.049374

O -2.713896 -2.919034 1.249695

O -0.562844 -3.648833 -0.552345

O -3.811893 -1.150241 -0.756125

O -1.659390 -1.879096 -2.576391

C -0.867257 -0.907401 -2.701390

C 0.506063 -3.145664 -0.115096

C -2.226168 -2.206035 2.172752

C -3.629330 0.042173 -0.396695

H -2.507203 -2.481132 3.208632

H -4.509920 0.713551 -0.448082

H 1.380976 -3.823539 -0.053533

H -0.573990 -0.639454 -3.736449

**Int4**

C -3.196115 3.494735 1.732981

C -4.084970 4.410180 1.153018

C -3.825218 4.923201 -0.122296

C -2.683207 4.520677 -0.812297

C -1.789014 3.580722 -0.252268

C -2.058983 3.085767 1.043255

H -3.389958 3.105070 2.735187

H -4.972724 4.733465 1.702666

H -4.510675 5.643861 -0.574739

H -2.476047 4.924259 -1.806986

H -1.367410 2.382424 1.509879

C -0.623255 3.199527 -1.027507

C 0.328849 2.254234 -0.789709

H -0.405242 3.829303 -1.899199

C 0.500462 1.151012 0.107832

C 1.951535 1.040009 0.367010

C 2.550887 0.598422 1.630622

C 3.929541 0.784216 1.876814

C 1.752095 0.082917 2.673978

C 4.494353 0.423938 3.097868

H 4.565261 1.254073 1.126747

C 2.321592 -0.271241 3.896732

H 0.681484 -0.048610 2.527222

C 3.693812 -0.114229 4.111669

H 5.562816 0.579276 3.265672

H 1.685533 -0.672763 4.689411

H 4.136690 -0.393299 5.071178

C 1.625587 2.268799 -1.592749

C 2.589098 1.474231 -0.786072

C 4.000200 1.302926 -1.225884

O 4.721649 2.217130 -1.542682

O 4.352967 0.009163 -1.256982

O 1.791750 2.814988 -2.655340

C 5.697643 -0.288187 -1.681068

H 5.866532 0.168178 -2.669320

H 6.403095 0.189862 -0.981026

C 5.857082 -1.793375 -1.709336

H 5.147974 -2.250705 -2.416107

H 6.878547 -2.055452 -2.026026

H 5.680494 -2.227730 -0.713469

Rh -0.720084 -0.490946 0.012381

Rh -2.065387 -2.523428 -0.398002

O 0.812273 -1.771260 0.549478

O -1.462644 -0.526425 1.942686

O -2.375319 0.574404 -0.607305

O -0.123119 -0.658567 -1.958558

O -1.361409 -2.516818 -2.332851

O -3.614525 -1.287898 -0.960810

O -0.427347 -3.630926 0.186472

O -2.682458 -2.397180 1.569343

C -2.258795 -1.456714 2.278371

C -3.424064 -0.047687 -0.946245

C -0.567569 -1.607162 -2.671519

C 0.619582 -3.021947 0.512408

H -0.202896 -1.622961 -3.718876

H 1.488592 -3.645231 0.808068

H -4.272229 0.591458 -1.267305

H -2.614938 -1.420398 3.328698

**Int5**

C -0.255831 3.373466 1.105641

C -1.147149 4.399103 1.131091

C -1.704128 4.950326 -0.080255

C -1.410584 4.433013 -1.318166

C -0.520352 3.329472 -1.416045

C 0.260073 2.881163 -0.200550

H 0.177697 2.978530 2.023682

H -1.459931 4.822512 2.088717

H -2.412605 5.778874 0.003306

H -1.905097 4.811775 -2.215902

H 1.222520 3.447651 -0.288840

C -0.247094 2.475928 -2.484974

C 0.546179 1.425617 -1.993630

H -0.576944 2.600821 -3.516111

C 0.772456 1.493260 -0.609528

C 2.072820 0.797627 -0.355015

C 2.821699 0.924088 0.905911

C 4.217589 1.118483 0.883605

C 2.164643 0.895374 2.151599

C 4.935606 1.269506 2.069567

H 4.736524 1.177537 -0.075435

C 2.889981 1.030998 3.337112

H 1.087637 0.729835 2.186757

C 4.275028 1.220607 3.301978

H 6.015895 1.430953 2.032052

H 2.368964 0.987047 4.297083

H 4.838113 1.335675 4.231716

C 1.527167 0.455548 -2.615525

C 2.463919 0.126141 -1.494374

C 3.591639 -0.814577 -1.689950

O 4.233507 -0.905035 -2.710056

O 3.817860 -1.585778 -0.604315

O 1.544726 0.092937 -3.767024

C 4.916459 -2.504913 -0.690905

H 4.766547 -3.163016 -1.562099

H 5.845327 -1.939750 -0.877210

C 4.980212 -3.284572 0.606453

H 4.049826 -3.849815 0.771451

H 5.816797 -4.000129 0.575223

H 5.130328 -2.611778 1.464732

Rh -0.997148 -0.162669 -0.105336

Rh -2.719778 -1.750569 0.558769

O 0.378301 -1.506253 0.637386

O -1.191089 0.662026 1.789581

O -2.514881 1.060660 -0.778535

O -0.947179 -1.139959 -1.918937

O -2.542233 -2.615682 -1.294479

O -4.112715 -0.428945 -0.187273

O -1.225156 -2.975471 1.262454

O -2.807390 -0.803533 2.385775

C -2.038284 0.167258 2.589303

C -3.708703 0.657869 -0.673893

C -1.709316 -2.129802 -2.103626

C -0.033256 -2.588204 1.141369

H -1.632045 -2.622244 -3.093750

H 0.750915 -3.273698 1.521858

H -4.488169 1.347746 -1.057242

H -2.105278 0.650800 3.585780

**Rh_2_(HCOO)_4_**

Rh -0.000521 1.194958 0.000000

Rh 0.000327 -1.194945 -0.000000

O 1.450320 1.130471 -1.451266

O 1.450320 1.130471 1.451266

O -1.450404 1.129848 1.451205

O -1.450404 1.129848 -1.451205

O -1.450471 -1.130503 -1.451421

O -1.450471 -1.130503 1.451421

O 1.450495 -1.129881 -1.451050

O 1.450495 -1.129881 1.451050

C 1.847324 0.000405 1.847666

C -1.846708 -0.000390 1.848462

C -1.846708 -0.000390 -1.848462

C 1.847324 0.000405 -1.847666

H -2.629326 -0.000027 -2.632710

H 2.630495 0.000162 -2.631370

H -2.629326 -0.000027 2.632710

H 2.630495 0.000162 2.631370

**TS1-CF_3_**

C -7.027252 -1.311522 -0.227683

C -7.754399 -2.466749 -0.544108

C -7.135776 -3.512808 -1.237994

C -5.798513 -3.401978 -1.612505

C -5.048377 -2.242608 -1.303472

C -5.690352 -1.195964 -0.600865

H -7.508975 -0.495134 0.316137

H -8.803316 -2.550556 -0.247950

H -7.698338 -4.416280 -1.485963

H -5.312895 -4.218691 -2.153106

H -5.140831 -0.291487 -0.342940

C -3.661600 -2.209255 -1.729917

C -2.694409 -1.255378 -1.608827

H -3.315145 -3.108450 -2.252210

C -2.670465 0.062235 -1.030903

C -2.930500 1.141134 -0.480244

C -3.195925 2.370634 0.159319

C -3.863534 3.408569 -0.537679

C -2.830191 2.560680 1.514431

C -4.157311 4.603333 0.112119

H -4.148895 3.255503 -1.580064

C -3.126085 3.763069 2.149178

H -2.319027 1.756092 2.042747

C -3.787850 4.782250 1.452162

H -4.675158 5.401966 -0.424247

H -2.839314 3.910910 3.192895

H -4.018482 5.723863 1.957278

C -1.330773 -1.389114 -2.140981

C -0.661561 -0.107562 -1.726208

C -0.358317 0.904951 -2.767526

O 0.440766 0.608765 -3.643159

O -0.923699 2.091703 -2.635882

O -0.798165 -2.307197 -2.735859

C -0.489345 3.130645 -3.554838

H 0.609957 3.119765 -3.595771

H -0.866453 2.882629 -4.559862

C -1.018648 4.456246 -3.060549

H -0.634069 4.683578 -2.054567

H -0.687759 5.253268 -3.745253

H -2.118891 4.466932 -3.030373

Rh 0.757088 -0.239939 -0.221169

Rh 2.550664 -0.390435 1.535552

O 0.626589 1.813747 0.200181

O 3.984101 -0.029270 0.055032

C 1.935095 -2.901131 0.245267

C 3.541849 0.153741 -1.114932

C 1.441669 2.283418 1.053407

C -0.182834 -0.834537 2.479176

C 1.322499 3.805240 1.244659

C -1.270763 -1.184617 3.507899

C 2.108225 -4.409809 -0.011385

C 4.634677 0.447000 -2.159124

N 2.313689 1.614283 1.748876

H 2.896424 2.169837 2.374164

N 1.060696 -2.236455 -0.457069

H 0.574576 -2.752273 -1.191542

O 2.687636 -2.446785 1.153731

O -0.636066 -0.623042 1.311216

N 2.295950 0.128050 -1.495309

N 1.060918 -0.777450 2.852116

H 2.106447 0.277676 -2.488150

H 1.238025 -0.996091 3.832052

F -1.958111 -2.266300 3.122360

F -0.760576 -1.433770 4.724352

F -2.144743 -0.171351 3.637935

F 2.263343 4.289504 2.071881

F 0.126456 4.125372 1.760633

F 1.445165 4.443261 0.071718

F 3.364223 -4.682406 -0.387352

F 1.854200 -5.104652 1.106309

F 1.285622 -4.864945 -0.970250

F 5.312819 1.553911 -1.823411

F 5.508508 -0.565090 -2.224982

F 4.130848 0.635432 -3.389208

**TS1-Me**

C 6.260054 -1.654278 -0.912515

C 6.976621 -2.832659 -0.666948

C 6.453257 -3.796427 0.202116

C 5.222576 -3.580867 0.819714

C 4.485431 -2.396535 0.586221

C 5.030142 -1.434624 -0.295286

H 6.665331 -0.900383 -1.592254

H 7.941256 -2.998517 -1.153782

H 7.006519 -4.718496 0.397540

H 4.813064 -4.334643 1.497549

H 4.487027 -0.513396 -0.501571

C 3.209568 -2.252447 1.270379

C 2.283324 -1.256620 1.277766

H 2.927059 -3.102639 1.902140

C 2.211490 0.042754 0.652116

C 2.545824 1.090438 0.074869

C 2.942182 2.290870 -0.552315

C 2.812452 2.444637 -1.954911

C 3.530179 3.331619 0.210010

C 3.260333 3.609112 -2.571530

H 2.362238 1.640580 -2.538490

C 3.966722 4.493963 -0.419042

H 3.639884 3.206551 1.288615

C 3.832979 4.634317 -1.806777

H 3.162171 3.723825 -3.653658

H 4.418597 5.294791 0.171029

H 4.180290 5.547784 -2.296428

C 1.028594 -1.274466 2.058158

C 0.341119 -0.020673 1.583744

C 0.136422 1.089980 2.549965

O -0.340789 0.864416 3.642972

O 0.449253 2.302934 2.096055

O 0.624505 -2.069933 2.874909

C 0.118439 3.416046 2.956766

H -0.920154 3.297682 3.299924

H 0.768138 3.380038 3.847090

C 0.306367 4.697873 2.177607

H -0.351311 4.723852 1.294545

H 0.054030 5.556246 2.820441

H 1.347033 4.817220 1.839058

Rh -1.194696 -0.278620 0.211565

Rh -3.077628 -0.632915 -1.318634

O -2.369957 1.144788 1.139249

O -0.540989 1.164501 -1.119149

O -0.182150 -1.728279 -0.853637

O -2.055186 -1.746852 1.372846

O -3.759857 -2.107481 -0.056715

O -1.894654 -2.019964 -2.292075

O -4.121939 0.775101 -0.231590

O -2.305650 0.871999 -2.491083

C -1.230724 1.443998 -2.150358

C -0.737932 -2.282705 -1.855397

C -3.121092 -2.336036 1.011256

C -3.558103 1.358603 0.737772

C -4.339482 2.409997 1.487092

H -4.299765 2.202993 2.567254

H -3.871308 3.393951 1.322082

H -5.382261 2.441562 1.146966

C 0.047839 -3.367138 -2.550829

H 1.122748 -3.140245 -2.532937

H -0.106991 -4.314436 -2.007090

H -0.301911 -3.498993 -3.583326

C -0.708582 2.527686 -3.062087

H -0.083008 3.241073 -2.510724

H -0.096446 2.061706 -3.852353

H -1.545800 3.046948 -3.548671

C -3.678013 -3.367179 1.960951

H -2.863899 -3.893886 2.477410

H -4.282928 -2.849435 2.724406

H -4.323631 -4.077911 1.428087

**TS1-gas phase**

C 6.009190 -1.528396 -1.277720

C 6.810734 -2.652014 -1.041321

C 6.403574 -3.611124 -0.108350

C 5.204524 -3.444488 0.581891

C 4.384321 -2.314980 0.361117

C 4.811293 -1.358170 -0.587108

H 6.322279 -0.779033 -2.008946

H 7.749869 -2.779599 -1.585531

H 7.022447 -4.491744 0.079836

H 4.886429 -4.196109 1.309144

H 4.197249 -0.481357 -0.788236

C 3.148068 -2.214203 1.122787

C 2.187133 -1.256953 1.174286

H 2.933691 -3.060199 1.786539

C 2.015249 0.032872 0.542563

C 2.250934 1.086447 -0.073589

C 2.508205 2.304066 -0.739194

C 2.192353 2.456611 -2.111201

C 3.130060 3.372527 -0.048088

C 2.497092 3.644418 -2.768548

H 1.705016 1.631771 -2.631644

C 3.423804 4.558572 -0.715292

H 3.371682 3.249960 1.008926

C 3.109309 4.695805 -2.073182

H 2.254568 3.758241 -3.827685

H 3.902367 5.381103 -0.178701

H 3.343118 5.628025 -2.593581

C 0.973558 -1.295197 2.023216

C 0.260111 -0.048225 1.551049

C 0.050489 1.081594 2.493716

O -0.402418 0.877932 3.596300

O 0.319237 2.292956 1.991134

O 0.618467 -2.084031 2.860491

C -0.107257 3.412560 2.792621

H -1.128093 3.213840 3.150935

H 0.543601 3.483375 3.680295

C -0.041888 4.662389 1.940589

H -0.702162 4.570792 1.064932

H -0.367540 5.532480 2.532130

H 0.980646 4.854154 1.581421

Rh -1.322038 -0.358300 0.249329

Rh -3.264836 -0.783071 -1.209870

O -2.401528 1.222459 1.036913

O -0.621431 0.890307 -1.250846

O -0.400649 -1.962882 -0.668044

O -2.218222 -1.638473 1.582887

O -3.983244 -2.062618 0.229314

O -2.172309 -2.333594 -2.031805

O -4.204310 0.795444 -0.268164

O -2.433468 0.536812 -2.564127

C -1.332339 1.065432 -2.283813

C -1.025645 -2.575356 -1.583342

C -3.312547 -2.194653 1.284329

C -3.578428 1.426090 0.618075

H -3.721252 -2.880449 2.054230

H -4.115000 2.270921 1.097995

H -0.482168 -3.428095 -2.040973

H -0.919787 1.775406 -3.032088

**TS2**

C -5.432062 -1.974874 0.678573

C -6.381732 -2.766245 0.021447

C -6.139246 -3.194756 -1.287595

C -4.959507 -2.828536 -1.935080

C -4.002071 -2.013207 -1.295575

C -4.255396 -1.602808 0.030324

H -5.607442 -1.653212 1.708323

H -7.303004 -3.056449 0.532765

H -6.871057 -3.820902 -1.804034

H -4.770976 -3.168638 -2.956876

H -3.508131 -1.015665 0.565108

C -2.790344 -1.646327 -2.025726

C -1.880532 -0.684487 -1.782211

H -2.580185 -2.227796 -2.931724

C -1.676907 0.466974 -0.846105

C -2.299317 1.314500 -0.077890

C -2.996786 1.946529 0.961345

C -2.816786 1.506550 2.300265

C -3.887474 3.018865 0.692079

C -3.532617 2.106868 3.330638

H -2.096132 0.708097 2.484551

C -4.588224 3.617348 1.731524

H -4.002123 3.360703 -0.337841

C -4.414291 3.158622 3.046100

H -3.399090 1.769166 4.360861

H -5.273665 4.442901 1.527627

H -4.969484 3.632625 3.859956

C -0.629171 -0.256455 -2.528725

C -0.340797 0.755919 -1.447042

C -0.057235 2.190257 -1.415866

O -0.721329 2.913855 -0.665663

O 0.904877 2.636493 -2.205539

O -0.149493 -0.583405 -3.579282

C 1.276672 4.022041 -2.080222

H 0.392664 4.648029 -2.283805

H 1.580529 4.203101 -1.037593

C 2.400948 4.287902 -3.057922

H 2.081253 4.084819 -4.091408

H 2.713658 5.341726 -2.991276

H 3.270205 3.650918 -2.835441

Rh 1.277819 -0.200646 -0.122150

Rh 3.071218 -1.213274 1.203923

O 2.079458 1.640894 0.400775

O 0.132060 -0.193307 1.626225

O 0.627732 -2.121406 -0.515604

O 2.556995 -0.292721 -1.725102

O 4.218046 -1.228843 -0.502832

O 2.300965 -3.053847 0.691696

O 3.728160 0.694034 1.631373

O 1.804036 -1.135881 2.832061

C 0.654543 -0.657084 2.681293

C 1.277475 -3.093569 -0.037562

C 3.710528 -0.777296 -1.563210

C 3.100706 1.667646 1.144438

H 4.350852 -0.805113 -2.468728

H 3.486900 2.677589 1.396399

H 0.891911 -4.102201 -0.293388

H 0.012319 -0.636515 3.588130

**TS3**

C -4.573385 -2.433399 -0.307819

C -5.705183 -2.756413 -1.064073

C -5.867082 -2.196875 -2.336231

C -4.911701 -1.313952 -2.839791

C -3.780453 -0.955561 -2.077386

C -3.616248 -1.548435 -0.808195

H -4.426470 -2.887792 0.675778

H -6.450163 -3.452788 -0.670695

H -6.741439 -2.452996 -2.940223

H -5.042762 -0.879849 -3.834783

H -2.706203 -1.353536 -0.237471

C -2.815120 -0.002692 -2.636136

C -1.992278 0.854960 -2.010513

H -2.768777 0.059490 -3.730575

C -1.726762 1.330451 -0.595283

C -2.106146 1.009950 0.620626

C -2.801857 1.008105 1.827611

C -2.279506 0.301379 2.949990

C -4.036188 1.710616 1.964240

C -2.964802 0.310545 4.158533

H -1.323076 -0.212426 2.836078

C -4.727313 1.681506 3.167396

H -4.425086 2.259423 1.104579

C -4.188160 0.988413 4.262719

H -2.557577 -0.214709 5.025339

H -5.679014 2.207974 3.268522

H -4.729198 0.980844 5.212900

C -0.962852 1.883822 -2.482268

C -0.740789 2.272733 -1.088315

C -0.004529 3.193492 -0.251995

O -0.128495 3.224609 0.965459

O 0.824814 4.002132 -0.936388

O -0.575730 2.199812 -3.580844

C 1.623694 4.903788 -0.161841

H 0.965030 5.531967 0.460660

H 2.253998 4.318436 0.527924

C 2.452037 5.733002 -1.122255

H 1.805774 6.307560 -1.803624

H 3.084179 6.441134 -0.563372

H 3.104703 5.091064 -1.733781

Rh 1.130717 -0.259103 0.117395

Rh 2.919482 -1.852312 0.207895

O 2.552468 1.219822 0.227848

O 1.037443 -0.358471 2.181861

O -0.180464 -1.847775 0.016856

O 1.320406 -0.285181 -1.918423

O 3.008926 -1.783210 -1.837907

O 1.511232 -3.345082 0.091478

O 4.236345 -0.286093 0.310858

O 2.747367 -1.836296 2.257142

C 1.862547 -1.106300 2.777576

C 0.296900 -3.016724 0.017275

C 2.200372 -1.021923 -2.439446

C 3.765982 0.884986 0.293957

H 2.269011 -0.997429 -3.544607

H 4.502376 1.711411 0.344624

H -0.435230 -3.845963 -0.053587

H 1.799418 -1.125135 3.884492

**TS4-CF_3_**

C -7.477305 0.070377 -0.014858

C -8.375263 -0.962217 -0.320119

C -7.929167 -2.096930 -1.006517

C -6.592166 -2.199474 -1.386529

C -5.672820 -1.167715 -1.087418

C -6.141422 -0.028695 -0.392939

H -7.825235 0.956517 0.521587

H -9.423410 -0.879599 -0.021247

H -8.626632 -2.903419 -1.245587

H -6.241544 -3.084778 -1.923490

H -5.456052 0.786214 -0.146455

C -4.296164 -1.333338 -1.513074

C -3.236158 -0.495128 -1.354999

H -4.062265 -2.261845 -2.043998

C -3.033753 0.772812 -0.772370

C -2.213312 1.655852 -0.419032

C -1.896569 2.900756 0.229061

C -0.598004 3.435870 0.313785

C -2.976153 3.606149 0.810967

C -0.389378 4.650588 0.966310

H 0.242417 2.897794 -0.118051

C -2.753830 4.819022 1.458920

H -3.986237 3.195857 0.747539

C -1.459531 5.346029 1.539228

H 0.624685 5.052838 1.029871

H -3.595906 5.354797 1.904046

H -1.286249 6.297341 2.048985

C -1.859632 -0.733655 -1.886829

C -0.961271 0.405034 -1.451427

C -0.415863 1.184863 -2.609658

O 0.253624 0.614679 -3.448404

O -0.708463 2.477581 -2.630259

O -1.492894 -1.663128 -2.570234

C -0.106188 3.275628 -3.683921

H 0.983608 3.279351 -3.524549

H -0.306527 2.788982 -4.650217

C -0.693144 4.666322 -3.618368

H -0.479799 5.148073 -2.651816

H -0.249466 5.283718 -4.415398

H -1.784248 4.645395 -3.767590

Rh 0.575652 -0.277491 -0.095399

Rh 2.513142 -1.087082 1.273779

O 1.726039 1.410552 -0.576499

O -0.145921 0.659251 1.643845

O 1.379192 -2.769023 1.787114

O 3.224367 -2.117557 -0.401366

C 2.566971 -1.952555 -1.471823

C 2.914650 1.458257 -0.125570

C 0.547690 0.549724 2.701570

C 0.214715 -2.842265 1.301547

C -0.561733 -4.099633 1.735051

C -0.068357 1.255256 3.921319

C 3.684087 2.710569 -0.575245

C 3.174161 -2.659446 -2.695842

F -1.785542 -4.162649 1.181475

F 0.100871 -5.208168 1.385046

F -0.717838 -4.116111 3.066101

F -1.307381 0.801886 4.154198

F 0.654114 1.060647 5.037675

F -0.147986 2.577573 3.715634

F 4.939854 2.736093 -0.104907

F 3.745430 2.776397 -1.912454

F 3.064224 3.821198 -0.141816

F 4.422905 -2.224367 -2.917663

F 3.232480 -3.982955 -2.493817

F 2.463435 -2.447098 -3.814387

N 3.483743 0.582033 0.645907

N -0.361759 -1.980904 0.509377

N 1.680759 -0.076766 2.824511

N 1.471795 -1.267070 -1.618051

H 1.082674 -1.184968 -2.557977

H 2.088909 -0.068526 3.758573

H -1.307922 -2.221612 0.224187

H 4.452533 0.779382 0.895641

**TS4-Me**

C 6.815773 -0.692062 -1.176577

C 7.679258 -1.764298 -0.912178

C 7.285565 -2.775448 -0.028986

C 6.036112 -2.715706 0.586917

C 5.153052 -1.642226 0.332022

C 5.567522 -0.630076 -0.562899

H 7.121529 0.098857 -1.865962

H 8.658382 -1.809222 -1.395998

H 7.955744 -3.613019 0.179982

H 5.727697 -3.505469 1.277018

H 4.906553 0.213208 -0.779583

C 3.865468 -1.631853 1.004928

C 2.863277 -0.719360 0.922614

H 3.660163 -2.470409 1.677942

C 2.659260 0.488105 0.230675

C 1.834721 1.388306 -0.072282

C 1.505736 2.581345 -0.810129

C 0.237684 3.191998 -0.794604

C 2.536170 3.150376 -1.593667

C 0.012285 4.343659 -1.549020

H -0.565440 2.758429 -0.202238

C 2.298458 4.301461 -2.342695

H 3.523384 2.683728 -1.606313

C 1.035340 4.903189 -2.323531

H -0.976217 4.809967 -1.532397

H 3.104862 4.730783 -2.942536

H 0.848796 5.806704 -2.909792

C 1.583045 -0.763850 1.711833

C 0.694709 0.386548 1.282079

C 0.386391 1.346923 2.388977

O -0.147736 0.968261 3.406124

O 0.741323 2.613968 2.155046

O 1.315766 -1.551044 2.585370

C 0.426288 3.579717 3.185114

H -0.669553 3.639229 3.284240

H 0.826939 3.216650 4.144242

C 1.029438 4.909360 2.792482

H 0.612170 5.276719 1.842231

H 0.810663 5.653627 3.574582

H 2.123306 4.833767 2.687391

Rh -1.021389 -0.364587 0.172417

Rh -3.001760 -1.257235 -0.949582

O -2.115407 1.357003 0.519074

O -0.425646 0.309664 -1.689672

O -0.094869 -2.157568 -0.261353

O -1.812457 -1.173720 1.894428

O -3.620597 -2.023648 0.851195

O -1.918207 -2.974361 -1.308150

O -3.946360 0.525424 -0.497954

O -2.283248 -0.453954 -2.706638

C -1.163847 0.133552 -2.709598

C -0.726791 -3.063720 -0.890504

C -2.901208 -1.823105 1.875238

C -3.323811 1.432183 0.122806

C -3.351719 -2.433466 3.179400

H -4.435503 -2.610506 3.172836

H -2.839939 -3.402103 3.308397

H -3.069250 -1.789143 4.023222

C -0.000887 -4.366173 -1.121785

H -0.378163 -4.864686 -2.024931

H 1.082423 -4.200802 -1.195649

H -0.188518 -5.028205 -0.259447

C -4.048225 2.728419 0.390317

H -3.787197 3.448457 -0.403784

H -5.135402 2.575758 0.375255

H -3.733561 3.155949 1.352444

C -0.665934 0.701295 -4.015561

H 0.413882 0.524192 -4.122044

H -1.210456 0.266367 -4.863606

H -0.824890 1.792393 -4.011154

**TS4-gas phase**

C 6.494661 -1.065113 -1.438003

C 7.343954 -2.126981 -1.099579

C 6.972358 -3.019812 -0.089413

C 5.760138 -2.851877 0.578240

C 4.892206 -1.786718 0.252105

C 5.284018 -0.895978 -0.771778

H 6.781299 -0.367460 -2.228646

H 8.293147 -2.257057 -1.625229

H 7.630170 -3.850284 0.177889

H 5.470181 -3.550598 1.367284

H 4.629793 -0.065471 -1.049420

C 3.641299 -1.659797 0.982152

C 2.672035 -0.719810 0.858490

H 3.431246 -2.419860 1.741738

C 2.474111 0.429287 0.074015

C 1.643120 1.319424 -0.248276

C 1.293622 2.466259 -1.046779

C 0.028611 3.082888 -1.025794

C 2.297098 2.983835 -1.896838

C -0.217953 4.188622 -1.838633

H -0.757032 2.689436 -0.384207

C 2.039317 4.089859 -2.703658

H 3.281812 2.512088 -1.913842

C 0.779637 4.697411 -2.677517

H -1.204951 4.657352 -1.818871

H 2.826023 4.479269 -3.354350

H 0.575994 5.565149 -3.309904

C 1.422242 -0.663624 1.705464

C 0.525562 0.453756 1.206179

C 0.229745 1.497321 2.240630

O -0.334671 1.203743 3.264645

O 0.635744 2.735332 1.927395

O 1.183035 -1.363506 2.652678

C 0.320502 3.773505 2.878570

H -0.775760 3.862213 2.946996

H 0.684244 3.466228 3.871488

C 0.968371 5.057446 2.405180

H 0.593478 5.350025 1.412721

H 0.744468 5.869794 3.114100

H 2.061887 4.947156 2.341690

Rh -1.177104 -0.389303 0.198485

Rh -3.176134 -1.433532 -0.780926

O -2.323501 1.313114 0.478643

O -0.698613 0.204184 -1.728221

O -0.189894 -2.164326 -0.171030

O -1.849140 -1.126442 1.996552

O -3.679013 -2.104850 1.091403

O -2.031396 -3.122859 -1.077694

O -4.169329 0.338429 -0.400905

O -2.568239 -0.710200 -2.615485

C -1.485085 -0.079687 -2.677574

C -0.829709 -3.111473 -0.713848

C -2.912457 -1.802897 2.043409

C -3.542649 1.284353 0.131933

H -3.199378 -2.171655 3.049246

H -4.116566 2.214655 0.324388

H -0.250103 -4.041578 -0.887811

H -1.172591 0.275999 -3.681340

**^1^TS5**

C -3.24043500 4.65441800 1.54359400

C -4.33694600 5.22181200 0.88039900

C -4.46181000 5.08332500 -0.50672500

C -3.49294500 4.38597600 -1.22493400

C -2.37217100 3.81898000 -0.57434800

C -2.27212100 3.95250200 0.83052900

H -3.14948700 4.75193400 2.62831900

H -5.09920400 5.76257000 1.44724300

H -5.31794500 5.51984800 -1.02688400

H -3.58598700 4.28231200 -2.30933300

H -1.42875800 3.49181900 1.34790500

C -1.37391300 3.14432900 -1.37910700

C -0.15442700 2.64908700 -1.00480400

H -1.62577000 2.98307000 -2.43402100

C 0.60782100 2.71615200 0.18269500

C 1.66740400 1.73967700 0.13855400

C 2.43734500 1.34736800 1.32803900

C 3.12284900 0.11856000 1.40546500

C 2.47718300 2.21573000 2.43802900

C 3.83973700 -0.21916400 2.55176500

H 3.06022500 -0.59421500 0.58196700

C 3.20105500 1.87604500 3.58087500

H 1.94768200 3.16813900 2.37711900

C 3.88767000 0.65943700 3.64068600

H 4.35801300 -1.18014900 2.60129300

H 3.23195000 2.56559000 4.42827900

H 4.45444800 0.39213900 4.53640800

C 0.73549900 1.91509000 -2.01150000

C 1.85980200 1.36074600 -1.20202100

C 3.00375100 0.67732900 -1.85193300

O 2.88758700 -0.13423100 -2.73915300

O 4.18683800 1.08926600 -1.35979800

O 0.58727100 1.91439400 -3.21022600

C 5.36553400 0.48348300 -1.91795400

H 5.32320600 -0.60458300 -1.74338500

H 5.36035800 0.63241400 -3.00974700

C 6.57330000 1.11837400 -1.26080400

H 6.56093900 0.95671500 -0.17206600

H 7.49694500 0.67761400 -1.66737600

H 6.59688300 2.20310200 -1.44699700

Rh -0.28595100 -0.95918200 0.09342100

Rh -1.74223000 -2.85497500 0.21960500

O 1.24187300 -2.25299900 -0.36704000

O 0.10952300 -1.22037200 2.09543200

O -1.89600300 0.22247600 0.58302500

O -0.78738300 -0.80819600 -1.88681500

O -2.14979100 -2.60617700 -1.77463700

O -3.27021700 -1.56879500 0.69781100

O -0.13911800 -4.03938400 -0.27198900

O -1.26074800 -3.01499400 2.20947700

C -0.45665900 -2.17351100 2.69664400

C -3.01153700 -0.33677400 0.77305000

C -1.58591800 -1.65106200 -2.37792800

C 0.97809900 -3.48398300 -0.45586200

H -1.81758500 -1.53822900 -3.45513800

H 1.82250400 -4.14976300 -0.72285900

H -3.85636700 0.33070600 1.03486500

H -0.22102100 -2.28436800 3.77393500

**^3^TS5**

C -5.670716 1.180223 1.641011

C -6.893709 0.979894 0.986202

C -6.934339 0.934334 -0.411377

C -5.761029 1.089967 -1.147556

C -4.519689 1.300490 -0.504987

C -4.497479 1.338507 0.908437

H -5.635489 1.210198 2.732978

H -7.811880 0.856994 1.566221

H -7.884369 0.775943 -0.927708

H -5.792989 1.053012 -2.239748

H -3.546630 1.482331 1.426031

C -3.330528 1.456159 -1.322738

C -2.051856 1.741693 -0.935460

H -3.458736 1.332554 -2.403921

C -1.410319 2.004529 0.286140

C -0.017627 2.249785 0.182887

C 0.815138 2.614046 1.344696

C 2.172939 2.259580 1.451152

C 0.218136 3.322954 2.406151

C 2.910611 2.621733 2.578042

H 2.641059 1.665836 0.666881

C 0.961400 3.693620 3.526620

H -0.836971 3.598259 2.336522

C 2.312720 3.345882 3.615229

H 3.961228 2.329184 2.651369

H 0.483821 4.254923 4.333575

H 2.896979 3.631490 4.493799

C -0.908171 1.833255 -1.964812

C 0.331070 2.114218 -1.188822

C 1.617721 2.360025 -1.886045

O 1.988411 1.746095 -2.857666

O 2.311421 3.379896 -1.341309

O -1.053657 1.745929 -3.161626

C 3.571374 3.701329 -1.953512

H 4.240755 2.828524 -1.871098

H 3.410832 3.878691 -3.029003

C 4.136421 4.919655 -1.252914

H 4.292363 4.724296 -0.180935

H 5.104246 5.194789 -1.700568

H 3.454740 5.778720 -1.349015

Rh 0.398482 -0.921601 0.028570

Rh 0.878093 -3.266925 0.125238

O 2.384562 -0.611326 -0.415213

O 0.844140 -0.822058 2.036000

O -1.551544 -1.376978 0.493842

O -0.037188 -1.176775 -1.956007

O 0.433503 -3.384372 -1.872731

O -1.095103 -3.589257 0.588016

O 2.826094 -2.826484 -0.351775

O 1.305145 -3.033959 2.121237

C 1.191854 -1.882415 2.623813

C -1.858761 -2.588433 0.666401

C 0.080745 -2.324850 -2.462461

C 3.140434 -1.617243 -0.517324

H -0.152525 -2.411318 -3.541711

H 4.197711 -1.413268 -0.778727

H -2.918733 -2.795104 0.915177

H 1.424388 -1.791694 3.703765

**TS6**

C -0.985676 3.454005 1.265365

C -2.194453 4.111281 1.125499

C -2.671121 4.478733 -0.155850

C -1.970736 4.138514 -1.303380

C -0.771890 3.403864 -1.196633

C -0.226243 3.129592 0.108923

H -0.577120 3.242416 2.254283

H -2.768105 4.391783 2.012229

H -3.618589 5.017339 -0.239596

H -2.373709 4.371743 -2.291798

H 0.863212 3.170980 0.231869

C -0.090026 2.750669 -2.276503

C 0.676360 1.709551 -1.841871

H -0.145952 3.066890 -3.320315

C 0.620740 1.273844 -0.474120

C 2.023447 0.894489 -0.136509

C 2.599507 0.942354 1.212446

C 3.991415 1.110527 1.380231

C 1.784968 0.902532 2.363473

C 4.552298 1.201556 2.652683

H 4.636479 1.212050 0.505748

C 2.352994 0.990609 3.635126

H 0.707827 0.776799 2.262674

C 3.735539 1.134638 3.786810

H 5.631186 1.338159 2.759890

H 1.708506 0.943650 4.516613

H 4.175136 1.206394 4.784964

C 1.847900 1.003684 -2.479939

C 2.654247 0.536092 -1.313457

C 3.947021 -0.173577 -1.504619

O 4.822171 0.206852 -2.244817

O 4.015536 -1.299485 -0.773800

O 2.064766 0.880899 -3.661197

C 5.231342 -2.062079 -0.875080

H 5.414030 -2.301890 -1.934769

H 6.073524 -1.434375 -0.538777

C 5.076590 -3.306014 -0.025643

H 4.235622 -3.921746 -0.379803

H 5.994361 -3.912285 -0.077594

H 4.893209 -3.043869 1.027644

Rh -0.907960 -0.195515 -0.031277

Rh -2.545334 -2.010821 0.228783

O 0.450465 -1.455870 0.885180

O -1.522730 0.487625 1.824261

O -2.417533 0.889206 -0.929304

O -0.434094 -1.047327 -1.849983

O -1.947145 -2.713456 -1.612305

O -3.925144 -0.783319 -0.687766

O -1.061799 -3.123806 1.124449

O -3.031583 -1.184555 2.058108

C -2.428756 -0.153073 2.437317

C -3.560182 0.360751 -1.054202

C -1.044901 -2.090455 -2.224784

C 0.078600 -2.610085 1.242750

H -0.740841 -2.493863 -3.212142

H 0.858908 -3.238174 1.719756

H -4.328571 0.991653 -1.547516

H -2.718044 0.255355 3.428080

**TS7**

C -1.319822 3.774811 -0.219110

C -0.481633 4.865050 -0.225518

C 0.681394 4.932134 0.596252

C 1.029923 3.883511 1.427572

C 0.214212 2.732991 1.476867

C -1.026760 2.689746 0.686250

H -2.210176 3.743738 -0.847595

H -0.709704 5.705366 -0.886465

H 1.311341 5.823502 0.551210

H 1.943899 3.919314 2.023608

H -1.914935 2.601900 1.524236

C 0.373478 1.495502 2.165649

C -0.723185 0.681468 1.834493

H 1.133165 1.281971 2.916629

C -1.572279 1.318703 0.909050

C -2.733970 0.458826 0.605803

C -3.796851 0.830355 -0.346013

C -5.157267 0.679355 -0.020973

C -3.453432 1.351139 -1.608118

C -6.147727 1.039000 -0.935806

H -5.435814 0.296485 0.963326

C -4.446730 1.694129 -2.527584

H -2.398812 1.455642 -1.873877

C -5.796235 1.543210 -2.192689

H -7.200800 0.927972 -0.665410

H -4.166078 2.080386 -3.510712

H -6.573483 1.821120 -2.908970

C -1.369734 -0.620428 2.218704

C -2.621555 -0.683781 1.360104

C -3.497421 -1.877921 1.376564

O -3.788159 -2.495286 2.373025

O -3.922927 -2.218098 0.140417

O -0.983119 -1.436798 3.016876

C -4.812820 -3.342774 0.056057

H -4.321914 -4.222194 0.503508

H -5.710015 -3.137388 0.663686

C -5.154723 -3.562341 -1.403218

H -4.248395 -3.768525 -1.993355

H -5.835296 -4.422122 -1.504825

H -5.648433 -2.676320 -1.831050

Rh 1.597052 -0.008141 0.358687

Rh 3.144638 -1.145091 -1.102106

O 0.272642 -1.543293 -0.023720

O 0.874448 0.980621 -1.307081

O 3.055435 1.437611 0.613919

O 2.419575 -1.064668 1.916152

O 3.843790 -2.158477 0.541121

O 4.496101 0.360703 -0.755896

O 1.728802 -2.603137 -1.387274

O 2.351576 -0.065563 -2.664335

C 1.412224 0.737261 -2.423747

C 4.155373 1.299530 0.010901

C 3.335041 -1.896062 1.664392

C 0.626041 -2.480155 -0.791293

H 3.736600 -2.456713 2.532045

H -0.121424 -3.281084 -0.959367

H 4.907382 2.097639 0.176304

H 1.008268 1.296288 -3.292241

**MECP1**

C 6.1910196 -0.5423325 -1.2907403

C 7.0789481 -1.4720034 -0.7317364

C 6.7846769 -2.0655170 0.5017067

C 5.6110324 -1.7293775 1.1722432

C 4.7047841 -0.7890833 0.6268545

C 5.0130983 -0.2109415 -0.6281818

H 6.4162783 -0.0868958 -2.2588128

H 7.9950098 -1.7413295 -1.2633804

H 7.4728098 -2.7936066 0.9382577

H 5.3824034 -2.1858915 2.1388108

H 4.3090449 0.4963307 -1.0727070

C 3.5178266 -0.4528864 1.3826857

C 2.5546555 0.4775829 1.0906440

H 3.3345603 -1.0283551 2.2972930

C 2.4173118 1.4776278 0.1231771

C 1.0810639 1.9717605 0.0505568

C 0.6385571 2.7848365 -1.0830362

C -0.7190716 2.9531666 -1.4279779

C 1.6215077 3.4027759 -1.8885180

C -1.0724937 3.7172603 -2.5372981

H -1.4905315 2.4593082 -0.8401138

C 1.2615440 4.1715622 -2.9937350

H 2.6711179 3.2889998 -1.6119867

C -0.0875728 4.3337356 -3.3212522

H -2.1268816 3.8305589 -2.8008140

H 2.0361831 4.6503121 -3.5977733

H -0.3732423 4.9393385 -4.1850409

C 1.3205025 0.6609913 1.9936691

C 0.4009975 1.5734850 1.2448647

C -0.6940236 2.2764129 1.9682796

O -1.3234378 1.8102435 2.8853731

O -0.8629523 3.5377885 1.5169993

O 1.2400099 0.2312735 3.1178771

C -1.8812360 4.3179886 2.1645456

H -2.8592633 3.8393939 1.9895161

H -1.7079910 4.3002710 3.2523973

H -1.8255844 5.7235410 1.6016205

H -1.9945170 5.7233917 0.5138467

H -2.6014203 6.3470603 2.0731683

H -0.8451535 6.1856093 1.7951841

Rh -0.8834414 -0.5922368 0.1764969

Rh -2.2139704 -2.4213349 -0.6730569

O -2.5712224 0.5865309 0.0144021

O -0.3780125 -0.1700018 -1.7810714

O 0.6982486 -1.9095671 0.2674616

O -1.4601074 -1.1897951 2.0535871

O -2.7101848 -2.8967782 1.2542182

O -0.5494806 -3.6145702 -0.5391692

O -3.8152081 -1.1361747 -0.7572181

O -1.6536543 -1.8599594 -2.5728872

C -0.8682111 -0.8838194 -2.6994575

C 0.5150389 -3.1028276 -0.1022272

C -2.2240561 -2.1819313 2.1764785

C -3.6419050 0.0570139 -0.3982593

H -2.5011061 -2.4602718 3.2126716

H -4.5271936 0.7222365 -0.4517129

H 1.3938412 -3.7751580 -0.0350316

H -0.5717694 -0.6185051 -3.7343962

**MECP2**

C 6.1915620 -0.5429482 -1.2903240

C 7.0794196 -1.4723029 -0.7309231

C 6.7847630 -2.0653987 0.5022814

C 5.6111032 -1.7293810 1.1724745

C 4.7051594 -0.7892706 0.6267984

C 5.0132352 -0.2111226 -0.6282413

H 6.4165763 -0.0878944 -2.2580744

H 7.9952623 -1.7420105 -1.2629311

H 7.4731174 -2.7929936 0.9390054

H 5.3826410 -2.1861497 2.1391012

H 4.3097873 0.4960380 -1.0734600

C 3.5181515 -0.4532767 1.3824688

C 2.5546477 0.4767216 1.0901842

H 3.3351032 -1.0276053 2.2975451

C 2.4178605 1.4769787 0.1230878

C 1.0817597 1.9713384 0.0503808

C 0.6388404 2.7846383 -1.0831907

C -0.7188919 2.9534980 -1.4274482

C 1.6215914 3.4025168 -1.8885086

C -1.0729234 3.7178940 -2.5365776

H -1.4906081 2.4595192 -0.8397968

C 1.2611918 4.1720951 -2.9937526

H 2.6713231 3.2879928 -1.6131383

C -0.0882901 4.3338340 -3.3207454

H -2.1277531 3.8316988 -2.7994972

H 2.0356099 4.6513136 -3.5974946

H -0.3740313 4.9394538 -4.1847904

C 1.3204061 0.6608568 1.9931626

C 0.4011509 1.5732082 1.2443514

C -0.6944736 2.2754523 1.9679507

O -1.3233942 1.8090694 2.8852801

O -0.8639216 3.5367727 1.5166161

O 1.2391058 0.2314602 3.1174280

C -1.8816761 4.3173332 2.1644244

H -2.8601350 3.8397423 1.9894461

H -1.7081960 4.3001474 3.2521898

C -1.8254612 5.7226603 1.6014745

H -1.9949346 5.7226358 0.5137886

H -2.6006472 6.3467685 2.0732000

H -0.8449617 6.1846512 1.7948101

Rh -0.8832880 -0.5916137 0.1760404

Rh -2.2138737 -2.4209607 -0.6728982

O -2.5713590 0.5868145 0.0142155

O -0.3783938 -0.1695289 -1.7818080

O 0.6985428 -1.9088970 0.2670163

O -1.4599566 -1.1889445 2.0533071

O -2.7097998 -2.8961736 1.2545028

O -0.5492135 -3.6140220 -0.5388626

O -3.8153018 -1.1360481 -0.7572505

O -1.6536235 -1.8600020 -2.5728933

C -0.8686072 -0.8837699 -2.6996914

C 0.5151058 -3.1021567 -0.1023045

C -2.2237231 -2.1809804 2.1763930

C -3.6419088 0.0574437 -0.3982016

H -2.5009511 -2.4592415 3.2127362

H -4.5272899 0.7223891 -0.4516880

H 1.3941402 -3.7744874 -0.0350067

H -0.5723929 -0.6185196 -3.7348674

**Int1-solution phase**

C -4.533665 4.057765 0.693738

C -3.858577 5.150954 1.251916

C -2.543855 4.999043 1.707138

C -1.911697 3.762113 1.603676

C -2.579224 2.643934 1.047588

C -3.907172 2.817543 0.591672

H -5.559028 4.175530 0.334070

H -4.357398 6.120511 1.330138

H -2.011618 5.848293 2.142795

H -0.884585 3.643622 1.958126

H -4.444151 1.978504 0.151835

C -1.840870 1.394664 0.983795

C -2.209616 0.139921 0.563036

H -0.809079 1.482332 1.339010

C -3.471206 -0.283116 0.062329

C -4.511219 -0.754079 -0.371861

C -5.719398 -1.316303 -0.884904

C -5.734736 -2.651403 -1.348212

C -6.910410 -0.558590 -0.938381

C -6.910768 -3.206794 -1.850788

H -4.816052 -3.240646 -1.307915

C -8.080893 -1.124521 -1.443043

H -6.908439 0.472940 -0.579685

C -8.085854 -2.447504 -1.900312

H -6.910878 -4.240244 -2.206771

H -8.996833 -0.529117 -1.479247

H -9.005547 -2.886970 -2.295075

C -1.195817 -0.931418 0.653214

C 0.191441 -0.732945 1.127924

C 0.453254 -0.961261 2.570324

O 0.392814 0.000622 3.308668

O 0.749304 -2.199472 2.902646

O -1.360455 -2.104722 0.338911

C 1.062438 -2.464804 4.300327

H 1.948671 -1.869361 4.568552

H 0.218258 -2.119352 4.916500

C 1.305939 -3.947604 4.448472

H 2.150920 -4.276930 3.823932

H 1.545717 -4.172358 5.499679

H 0.413111 -4.527375 4.166899

Rh 1.684073 -0.236774 -0.083274

Rh 3.535813 0.417360 -1.577057

O 2.081938 -2.171334 -0.675512

O 0.421878 -0.002416 -1.694659

O 1.503397 1.781324 0.332451

O 3.081740 -0.426308 1.421725

O 4.786076 0.185226 0.059625

O 3.200868 2.381354 -1.036353

O 3.780200 -1.573815 -2.046073

O 2.116775 0.598135 -3.073278

C 0.913291 0.356068 -2.806787

C 2.277313 2.618625 -0.221561

C 4.296024 -0.174441 1.158427

C 3.013126 -2.405407 -1.500644

H 5.000231 -0.288816 2.007983

H 3.157434 -3.473225 -1.763647

H 2.107083 3.679006 0.055231

H 0.183934 0.465858 -3.635245

**TS1-solution phase**

C 6.104112 -1.427986 -1.256256

C 6.902394 -2.559205 -1.042392

C 6.480150 -3.550564 -0.149676

C 5.268265 -3.409151 0.523613

C 4.449914 -2.272631 0.323424

C 4.892664 -1.281975 -0.583630

H 6.430681 -0.653487 -1.954830

H 7.852062 -2.667272 -1.572879

H 7.097533 -4.436162 0.020362

H 4.937408 -4.184645 1.219599

H 4.284841 -0.397040 -0.767715

C 3.201378 -2.204986 1.065364

C 2.222614 -1.260638 1.120648

H 2.995530 -3.071299 1.704543

C 2.056340 0.028361 0.497717

C 2.246591 1.087436 -0.119253

C 2.474860 2.297811 -0.810560

C 2.142184 2.412984 -2.183238

C 3.087445 3.390602 -0.148954

C 2.415598 3.592864 -2.868119

H 1.673916 1.567882 -2.689812

C 3.350340 4.567312 -0.845276

H 3.352954 3.295865 0.905402

C 3.015112 4.670326 -2.201382

H 2.161379 3.678334 -3.927328

H 3.822010 5.408791 -0.332355

H 3.225300 5.595613 -2.744083

C 1.010658 -1.359091 1.957896

C 0.229318 -0.128659 1.567222

C 0.050921 0.946779 2.578041

O -0.380683 0.662985 3.676173

O 0.326818 2.178995 2.162454

O 0.679542 -2.193642 2.767946

C 0.013994 3.252107 3.082856

H -1.020751 3.120501 3.432947

H 0.676148 3.167367 3.960064

C 0.198262 4.569208 2.365059

H -0.466964 4.640404 1.490287

H -0.046894 5.394777 3.052009

H 1.236632 4.703678 2.025285

Rh -1.358830 -0.380834 0.266549

Rh -3.285482 -0.702331 -1.244005

O -2.512985 1.025862 1.250084

O -0.736444 1.086274 -1.045878

O -0.372022 -1.811712 -0.852377

O -2.192065 -1.874584 1.421792

O -3.946565 -2.187830 0.023699

O -2.127207 -2.088679 -2.258960

O -4.292904 0.704970 -0.115321

O -2.514517 0.812985 -2.420823

C -1.440739 1.358139 -2.060466

C -0.966603 -2.331113 -1.841618

C -3.265000 -2.430904 1.053044

C -3.693683 1.242469 0.850744

H -3.644227 -3.229026 1.724428

H -4.262199 2.000658 1.428690

H -0.384036 -3.090853 -2.403562

H -1.057530 2.176380 -2.704694

**TS4-solution phase**

C 6.525669 -1.009236 -1.462773

C 7.408938 -2.047461 -1.135117

C 7.063523 -2.970761 -0.142267

C 5.842282 -2.857219 0.520583

C 4.941534 -1.814556 0.205856

C 5.306263 -0.892532 -0.801325

H 6.792484 -0.289607 -2.240668

H 8.365407 -2.135595 -1.656824

H 7.748868 -3.782207 0.114726

H 5.571005 -3.578240 1.296161

H 4.626985 -0.080293 -1.072907

C 3.687102 -1.743470 0.933822

C 2.693483 -0.821914 0.841184

H 3.502888 -2.535266 1.666879

C 2.475378 0.349693 0.094194

C 1.672718 1.262044 -0.227355

C 1.348303 2.426304 -1.011405

C 0.096673 3.069713 -0.988244

C 2.366403 2.927076 -1.855360

C -0.127143 4.183487 -1.797260

H -0.695996 2.692312 -0.346085

C 2.130699 4.041625 -2.658067

H 3.341906 2.436887 -1.872767

C 0.882699 4.673847 -2.633131

H -1.103860 4.673590 -1.775704

H 2.926822 4.417937 -3.305318

H 0.697518 5.547600 -3.263213

C 1.445207 -0.799864 1.676595

C 0.567877 0.350105 1.214001

C 0.312893 1.374907 2.278607

O -0.209893 1.057295 3.321717

O 0.697534 2.616159 1.975092

O 1.185957 -1.535682 2.595403

C 0.426413 3.644044 2.958433

H -0.665348 3.729965 3.079178

H 0.844739 3.325229 3.925639

C 1.044568 4.936833 2.476965

H 0.610888 5.257721 1.517312

H 0.858345 5.728184 3.220236

H 2.133969 4.833889 2.351127

Rh -1.204682 -0.415011 0.212894

Rh -3.261446 -1.297990 -0.815500

O -2.243413 1.345794 0.537689

O -0.661150 0.178853 -1.692720

O -0.337918 -2.247316 -0.191685

O -1.950358 -1.146789 1.992872

O -3.823109 -1.991894 1.038574

O -2.224258 -3.051427 -1.156652

O -4.139884 0.527878 -0.389426

O -2.586880 -0.557028 -2.622808

C -1.455145 -0.010472 -2.657522

C -1.027497 -3.131598 -0.775862

C -3.052373 -1.759635 2.008557

C -3.455767 1.415471 0.177796

H -3.378249 -2.137599 3.000000

H -3.966074 2.377590 0.391084

H -0.508330 -4.092285 -0.974201

H -1.105450 0.349917 -3.647238

**1a**

C -2.591287 3.956105 0.016302

C -1.921017 5.183575 0.070904

C -0.532919 5.205125 0.240473

C 0.172237 4.008698 0.352936

C -0.485579 2.758338 0.295485

C -1.888358 2.757391 0.126326

H -3.676573 3.933485 -0.111409

H -2.479161 6.119200 -0.016042

H 0.000666 6.157698 0.286728

H 1.257279 4.032253 0.487355

H -2.425015 1.810999 0.087206

C 0.337866 1.558803 0.417800

C 0.017255 0.229871 0.382108

H 1.395176 1.779564 0.594590

C -1.273534 -0.335563 0.208507

C -2.328431 -0.930818 0.052332

C -3.552591 -1.645434 -0.110335

C -3.621591 -3.013242 0.236949

C -4.708767 -1.010742 -0.614753

C -4.814158 -3.718497 0.083987

H -2.729362 -3.505530 0.629045

C -5.896358 -1.725402 -0.765253

H -4.662432 0.045098 -0.889808

C -5.954072 -3.079371 -0.416789

H -4.855456 -4.775697 0.357929

H -6.784101 -1.223037 -1.157703

H -6.886900 -3.636205 -0.535714

C 1.101425 -0.800264 0.657743

C 2.443311 -0.589605 0.033606

C 3.676059 -1.271313 0.506881

O 3.763510 -1.927693 1.510331

O 4.703272 -1.034881 -0.339912

O 0.884499 -1.766414 1.351302

C 5.972597 -1.614339 0.012126

H 6.268718 -1.246818 1.008108

H 5.854325 -2.706807 0.094507

C 6.973694 -1.229874 -1.057025

H 7.080770 -0.136135 -1.124007

H 7.959312 -1.658913 -0.818796

H 6.662611 -1.607082 -2.043433

N 2.552594 0.141892 -1.057219

N 2.655292 0.756563 -2.000522

**Com1-TS**

C -4.227573 3.943747 1.117572

C -3.537430 4.857998 1.921981

C -2.275149 4.523569 2.424044

C -1.712486 3.285773 2.120830

C -2.400319 2.342159 1.321310

C -3.672773 2.699653 0.820974

H -5.209399 4.205550 0.714783

H -3.980675 5.830179 2.152581

H -1.728187 5.232316 3.050889

H -0.726389 3.021776 2.510918

H -4.217762 2.002256 0.187304

C -1.718613 1.078267 1.068475

C -2.167189 -0.085274 0.503331

H -0.682407 1.085301 1.401036

C -3.489776 -0.329240 0.035282

C -4.602491 -0.625599 -0.368407

C -5.892825 -0.989547 -0.857811

C -6.047016 -2.153779 -1.643076

C -7.031693 -0.205343 -0.571878

C -7.303274 -2.515204 -2.127338

H -5.167585 -2.762373 -1.862994

C -8.283941 -0.575269 -1.061207

H -6.922185 0.691633 0.041408

C -8.424989 -1.729085 -1.840024

H -7.409109 -3.417971 -2.734357

H -9.157579 0.040282 -0.832053

H -9.408124 -2.016063 -2.221486

C -1.263636 -1.270177 0.302182

C 0.160121 -1.250011 0.842679

C 0.410449 -1.295942 2.318586

O 0.076146 -0.359464 3.008372

O 1.060638 -2.369949 2.755356

O -1.646071 -2.241675 -0.323072

C 1.467719 -2.363177 4.142220

H 2.137729 -1.503572 4.299335

H 0.576296 -2.209167 4.770102

C 2.152161 -3.681552 4.430446

H 3.036748 -3.814277 3.789312

H 2.478868 -3.708019 5.481476

H 1.469113 -4.527986 4.260907

N 0.531425 -3.128430 0.388471

N 0.985847 -4.038587 -0.031750

Rh 1.645328 -0.235984 -0.244640

Rh 3.364135 0.991867 -1.506883

O 2.002355 -1.796131 -1.545880

O 0.273908 0.530000 -1.576683

O 1.493610 1.467473 0.922588

O 3.163335 -0.912300 0.986572

O 4.746188 0.206928 -0.187302

O 3.075857 2.587698 -0.242888

O 3.568413 -0.658591 -2.715214

O 1.851341 1.679827 -2.725102

C 0.676169 1.299544 -2.498034

C 2.222605 2.471637 0.667621

C 4.349526 -0.541602 0.737611

C 2.856183 -1.661111 -2.471863

H 5.125729 -0.933147 1.427292

H 2.980902 -2.539517 -3.137227

H 2.080940 3.345890 1.335344

H -0.111981 1.678169 -3.180103

**Com1**

C 4.870107 4.098547 -1.191410

C 4.518876 5.440840 -1.006169

C 3.362030 5.759067 -0.287449

C 2.569086 4.741795 0.240683

C 2.916319 3.381323 0.079299

C 4.083840 3.079188 -0.656505

H 5.765501 3.843787 -1.764333

H 5.141256 6.235018 -1.426368

H 3.075673 6.803718 -0.141795

H 1.663679 4.994572 0.799172

H 4.362579 2.039179 -0.821725

C 2.031344 2.383353 0.670619

C 2.188240 1.037662 0.834550

H 1.080772 2.776622 1.040199

C 3.344794 0.260929 0.565376

C 4.318211 -0.466384 0.435668

C 5.458635 -1.304903 0.258986

C 5.332296 -2.711130 0.313350

C 6.735925 -0.747907 0.026492

C 6.447740 -3.528566 0.137255

H 4.348933 -3.150006 0.495044

C 7.845386 -1.573672 -0.149077

H 6.844526 0.337994 -0.010037

C 7.707315 -2.965045 -0.095061

H 6.334505 -4.614781 0.182111

H 8.827266 -1.128114 -0.327826

H 8.579666 -3.608814 -0.232068

C 1.061314 0.322621 1.547896

C 0.502146 -0.942498 0.932710

C 0.925109 -1.554429 -0.365240

O 1.427290 -0.937263 -1.265739

O 0.646891 -2.868638 -0.391764

O 0.625272 0.722210 2.604521

C 0.857023 -3.545040 -1.648665

H 0.302093 -3.002012 -2.428899

H 1.927282 -3.489468 -1.904373

C 0.379733 -4.973102 -1.493360

H -0.690535 -5.003345 -1.238354

H 0.527558 -5.520663 -2.437118

H 0.938828 -5.495299 -0.701517

N 0.013778 -1.787008 1.857484

N -0.408256 -2.444367 2.659839

Rh -1.861588 -0.205986 0.186783

Rh -4.087585 0.485328 -0.438920

O -2.585246 -2.128668 0.347896

O -2.400638 0.121590 2.142551

O -1.292268 1.764477 -0.009826

O -1.506206 -0.531876 -1.819893

O -3.588237 0.133862 -2.399410

O -3.376900 2.402974 -0.611293

O -4.672350 -1.477423 -0.231851

O -4.473141 0.806923 1.553099

C -3.561957 0.552262 2.385303

C -2.159883 2.610897 -0.363130

C -2.427816 -0.289418 -2.646950

C -3.810772 -2.327982 0.109578

H -2.186457 -0.471678 -3.713658

H -4.162816 -3.374238 0.214076

H -1.807744 3.657067 -0.465634

H -3.804397 0.723932 3.453183

**Int1-CF3-EA_ligand**

C -6.089604 4.000581 0.379471

C -5.401068 5.169183 0.730169

C -4.063247 5.095363 1.134685

C -3.420503 3.860624 1.187446

C -4.098497 2.669570 0.830823

C -5.450511 2.763514 0.426123

H -7.135599 4.056685 0.067336

H -5.908976 6.136380 0.689521

H -3.521475 6.003448 1.410794

H -2.375241 3.802283 1.503235

H -5.999390 1.862773 0.154496

C -3.345800 1.431172 0.914147

C -3.655111 0.142525 0.551407

H -2.339492 1.565242 1.321145

C -4.847624 -0.332609 -0.059161

C -5.827006 -0.838462 -0.585503

C -6.966427 -1.441560 -1.199672

C -8.092065 -0.669212 -1.563061

C -6.979407 -2.832020 -1.452553

C -9.197274 -1.275332 -2.159889

H -8.089589 0.406525 -1.374572

C -8.089605 -3.427308 -2.050285

H -6.110275 -3.432492 -1.175128

C -9.200890 -2.653441 -2.405164

H -10.062713 -0.667657 -2.436673

H -8.087991 -4.503584 -2.241354

H -10.069145 -3.124146 -2.873582

C -2.619773 -0.889613 0.784502

C -1.389576 -0.607582 1.547795

C -1.464344 -0.775432 3.014639

O -1.890562 0.134491 3.693412

O -0.990261 -1.933477 3.467588

O -2.664288 -2.047565 0.364993

C -1.054418 -2.169451 4.907420

H -0.419432 -1.420342 5.405139

H -2.092701 -2.009320 5.234337

C -0.585765 -3.580731 5.170306

H 0.455729 -3.725842 4.844426

H -0.636389 -3.780961 6.252142

H -1.224018 -4.314875 4.654368

Rh 0.322870 -0.214491 0.598518

Rh 2.471737 0.146198 -0.654021

O -0.634013 0.926437 -0.878927

O 0.745970 1.550446 1.645581

O 3.477393 -0.922987 0.850137

O 2.138252 -1.642594 -1.694732

C 0.070274 1.312768 -1.864412

C 1.799259 2.189388 1.331428

C 2.758041 -1.401319 1.770718

C 1.041651 -2.222248 -1.454250

C 3.540430 -2.206381 2.825460

C 0.796455 -3.482465 -2.304236

C -0.733668 2.072547 -2.932893

C 1.992458 3.485367 2.135211

N 1.468702 -1.265444 1.907014

N 0.137806 -1.851986 -0.592727

N 1.345808 1.125051 -2.021506

N 2.669568 1.841740 0.433463

H 3.461233 2.470885 0.306140

H 1.750922 1.497451 -2.879424

H -0.711360 -2.415205 -0.522424

H 1.038602 -1.753984 2.690266

F 4.238558 -3.188771 2.244018

F 2.733626 -2.764797 3.745068

F 4.404502 -1.412398 3.470986

F 2.095811 3.219496 3.443533

F 3.097727 4.150179 1.762662

F 0.945208 4.304802 1.963793

F 1.806872 -4.348781 -2.155003

F -0.339146 -4.112314 -1.969183

F 0.720024 -3.160710 -3.602851

F -1.708151 1.299688 -3.428066

F 0.037478 2.468239 -3.959340

F -1.301547 3.164583 -2.404599

O 4.522139 0.626175 -1.846925

C 5.442048 -0.136921 -2.116400

C 5.476034 -1.594490 -1.765007

O 6.524613 0.272519 -2.766889

H 6.411028 -2.065501 -2.091377

H 5.348672 -1.702506 -0.677619

H 4.613323 -2.092420 -2.232563

C 6.605625 1.662898 -3.161975

H 6.542226 2.289212 -2.257974

H 5.736692 1.900160 -3.795856

C 7.911508 1.863458 -3.896539

H 7.999512 2.916159 -4.208126

H 8.771665 1.622066 -3.252362

H 7.961480 1.232464 -4.797917

**Int1-Me-EA_ligand**

C 5.210002 -3.051565 3.129599

C 4.593421 -3.237616 4.373326

C 3.447582 -2.502322 4.697015

C 2.925329 -1.589981 3.782235

C 3.537745 -1.383046 2.522820

C 4.694030 -2.136379 2.213705

H 6.102650 -3.627384 2.871755

H 5.004917 -3.955896 5.087350

H 2.960186 -2.642184 5.665241

H 2.030165 -1.016571 4.035618

H 5.183646 -2.007202 1.249677

C 2.923379 -0.405523 1.636883

C 3.309432 0.080535 0.413709

H 1.991009 0.012275 2.030206

C 4.467192 -0.264511 -0.336138

C 5.432582 -0.484177 -1.051769

C 6.554764 -0.734875 -1.898546

C 6.623467 -0.134898 -3.176372

C 7.608667 -1.577863 -1.480906

C 7.716216 -0.376073 -4.008273

H 5.811178 0.518249 -3.502627

C 8.696823 -1.812138 -2.321252

H 7.566018 -2.042935 -0.493624

C 8.754979 -1.213962 -3.585464

H 7.758082 0.093343 -4.994560

H 9.506569 -2.465986 -1.987062

H 9.609768 -1.400435 -4.240766

C 2.455118 1.120794 -0.218210

C 1.197478 1.605272 0.383570

C 1.287788 2.794333 1.257557

O 1.416077 2.633229 2.455367

O 1.198670 3.954586 0.629466

O 2.694221 1.662768 -1.291530

C 1.240714 5.158683 1.439830

H 0.393807 5.131076 2.143005

H 2.170874 5.152824 2.029234

C 1.167157 6.347962 0.510942

H 0.232969 6.340316 -0.072167

H 1.196703 7.276283 1.103063

H 2.017135 6.358405 -0.189251

Rh -0.585845 0.749879 0.021599

Rh -2.761064 -0.309600 -0.386967

O -0.912391 1.748526 -1.751449

O 0.267712 -0.811016 -1.014354

O -0.533185 -0.389969 1.748672

O -1.597625 2.237484 1.031641

O -3.600697 1.275231 0.655893

O -2.534991 -1.345518 1.379654

O -2.899099 0.765199 -2.134971

O -1.726420 -1.795108 -1.386553

C -0.469390 -1.733681 -1.491853

C -1.486928 -1.173389 2.060439

C -2.865473 2.189360 1.126952

C -1.960063 1.549844 -2.445135

C -3.535861 3.343476 1.830162

H -2.897344 3.729292 2.636721

H -3.690051 4.155888 1.100010

H -4.515273 3.043077 2.225384

C -1.326522 -1.950898 3.342412

H -0.454622 -2.618408 3.255290

H -1.130498 -1.257026 4.174230

H -2.223786 -2.545641 3.554624

C 0.232960 -2.818163 -2.270074

H 0.488983 -2.425778 -3.268421

H 1.172380 -3.098057 -1.772451

H -0.415872 -3.695388 -2.389228

C -2.055239 2.300157 -3.749385

H -1.729274 3.341518 -3.614139

H -1.373309 1.830497 -4.477542

H -3.078589 2.266291 -4.144612

O -4.925091 -1.407705 -0.839566

C -5.907355 -1.554179 -0.130365

C -6.031185 -1.051604 1.278972

O -6.989140 -2.212060 -0.544117

H -7.072377 -1.073054 1.624526

H -5.420011 -1.696416 1.930842

H -5.614447 -0.037142 1.339128

C -6.973728 -2.773607 -1.877034

H -6.125871 -3.472440 -1.956154

H -6.803521 -1.961053 -2.600968

C -8.298240 -3.465402 -2.107783

H -8.313555 -3.902854 -3.118486

H -8.456020 -4.276626 -1.379452

H -9.137131 -2.755991 -2.027505

**TS1-CF3-EA_ligand**

C 7.236203 -2.145230 -1.307103

C 7.913474 -3.336458 -1.015033

C 7.439321 -4.177370 -0.001986

C 6.295835 -3.826957 0.713024

C 5.600114 -2.627014 0.434470

C 6.093547 -1.790350 -0.593573

H 7.603218 -1.488738 -2.100066

H 8.809642 -3.608520 -1.578844

H 7.962732 -5.108520 0.229071

H 5.923074 -4.483821 1.503500

H 5.578762 -0.861997 -0.838076

C 4.416656 -2.338599 1.226185

C 3.556910 -1.281557 1.227309

H 4.157637 -3.103478 1.967099

C 3.487620 -0.058817 0.469917

C 3.677824 0.892244 -0.301076

C 3.843449 1.977449 -1.188370

C 4.778162 3.002823 -0.900347

C 3.098004 2.028542 -2.391617

C 4.959414 4.049281 -1.799482

H 5.354225 2.956904 0.025701

C 3.286824 3.083929 -3.278793

H 2.381783 1.234926 -2.604268

C 4.214565 4.091785 -2.985908

H 5.682415 4.838074 -1.578569

H 2.707412 3.125304 -4.203981

H 4.358931 4.917348 -3.687746

C 2.407497 -1.143701 2.137420

C 1.778209 0.140961 1.703952

C 1.847973 1.321615 2.588313

O 1.281325 1.272273 3.671502

O 2.464776 2.393530 2.115243

O 1.993261 -1.891238 3.006185

C 2.380644 3.602935 2.913224

H 1.337759 3.735128 3.237574

H 2.998278 3.466725 3.815605

C 2.855988 4.764079 2.071423

H 2.228104 4.883010 1.175095

H 2.791466 5.690150 2.664617

H 3.902307 4.633333 1.755501

Rh -0.057555 -0.042991 0.665809

Rh -2.288050 -0.232196 -0.478625

O 0.100167 1.901071 -0.125798

O -3.202642 0.501296 1.260859

C -1.517760 -2.580006 1.015340

C -2.424889 0.806788 2.211661

C -0.884776 2.330115 -0.799028

C 0.015098 -1.124722 -2.046017

C -0.707084 3.770904 -1.309111

C 0.725770 -1.726764 -3.269389

C -1.715872 -4.003927 1.566422

C -3.146544 1.354738 3.455268

N -1.969027 1.674994 -1.096237

H -2.657658 2.188808 -1.643651

N -0.430732 -1.929428 1.320198

H 0.206294 -2.383263 1.976554

O -2.464534 -2.180027 0.278916

O 0.801991 -0.807386 -1.101877

N -1.128980 0.695189 2.225616

N -1.275698 -0.975705 -2.061581

H -0.642640 0.973396 3.080907

H -1.757029 -1.303098 -2.897961

F 1.452402 -2.795690 -2.922425

F -0.137064 -2.109923 -4.224668

F 1.561602 -0.828540 -3.820813

F -1.832459 4.256935 -1.860905

F 0.256095 3.826585 -2.242502

F -0.356602 4.594697 -0.312048

F -2.866174 -4.093965 2.246379

F -1.764856 -4.890801 0.560729

F -0.723707 -4.378553 2.390628

F -3.910400 2.407648 3.126903

F -3.947430 0.419826 3.985030

F -2.293079 1.752458 4.411629

C -5.788221 1.244256 -0.680675

H -5.530217 2.137829 -1.274533

H -6.848956 1.306741 -0.406170

H -5.150356 1.233054 0.213222

C -5.510831 0.026886 -1.514698

O -4.403417 -0.457763 -1.698836

O -6.604266 -0.474667 -2.080044

C -6.463018 -1.638197 -2.929427

H -5.978868 -2.440124 -2.350623

H -5.796661 -1.382036 -3.768630

C -7.840854 -2.037881 -3.406130

H -7.761683 -2.921424 -4.058900

H -8.495428 -2.294567 -2.558290

H -8.314991 -1.226619 -3.980994

**TS1-Me-EA_ligand**

C 6.348646 -2.548289 -2.066398

C 6.991473 -3.769293 -1.825669

C 6.659763 -4.516662 -0.690195

C 5.692907 -4.044303 0.195808

C 5.035006 -2.812646 -0.028525

C 5.382972 -2.072041 -1.181376

H 6.603755 -1.962400 -2.953208

H 7.748739 -4.136955 -2.523095

H 7.156356 -5.470830 -0.496216

H 5.432872 -4.630014 1.081698

H 4.893100 -1.121357 -1.387810

C 4.037191 -2.394891 0.945970

C 3.255080 -1.287193 1.037761

H 3.873491 -3.100706 1.768685

C 3.127134 -0.087779 0.242613

C 3.377571 0.806582 -0.581748

C 3.680666 1.833719 -1.501753

C 3.144068 1.804424 -2.812823

C 4.574000 2.873218 -1.140272

C 3.495202 2.789670 -3.731178

H 2.458664 1.001915 -3.088523

C 4.909866 3.856872 -2.066225

H 4.995848 2.887873 -0.133911

C 4.372871 3.816972 -3.359711

H 3.083025 2.761676 -4.742718

H 5.597123 4.657792 -1.783443

H 4.642460 4.589930 -4.084025

C 2.292878 -1.015990 2.128224

C 1.624526 0.236551 1.649292

C 1.786871 1.483310 2.430451

O 1.556369 1.488856 3.623314

O 2.130581 2.564593 1.725780

O 2.078793 -1.630004 3.150396

C 2.148640 3.815493 2.447123

H 1.189687 3.930982 2.974774

H 2.942772 3.775288 3.210968

C 2.378911 4.937761 1.460382

H 1.581190 4.968756 0.701580

H 2.381622 5.900243 1.996510

H 3.345222 4.831148 0.943912

Rh -0.295921 -0.022809 0.778894

Rh -2.561413 -0.349825 -0.104654

O -1.035890 1.657202 1.732160

O 0.063540 1.128430 -0.906086

O 0.248612 -1.722047 -0.259447

O -0.902498 -1.193014 2.362175

O -2.962085 -1.547872 1.522588

O -1.816819 -1.989184 -1.122320

O -3.134311 1.314469 0.987224

O -2.039139 0.881878 -1.675572

C -0.867281 1.353779 -1.739342

C -0.609611 -2.331650 -0.972099

C -2.066708 -1.696067 2.403686

C -2.265151 1.959185 1.643974

C -2.711487 3.212182 2.358695

H -2.151380 3.340750 3.295338

H -2.498784 4.081466 1.713600

H -3.790866 3.183293 2.558525

C -0.143645 -3.589128 -1.666155

H 0.899965 -3.485861 -1.994672

H -0.189108 -4.424239 -0.946853

H -0.793981 -3.828210 -2.517990

C -0.545229 2.233876 -2.923924

H 0.295885 2.902975 -2.702754

H -0.270649 1.593486 -3.778893

H -1.431156 2.816004 -3.213979

C -2.408019 -2.510444 3.627968

H -1.532114 -3.083006 3.963544

H -2.689752 -1.821084 4.441645

H -3.254653 -3.180314 3.427493

O -4.845848 -0.699081 -1.013705

C -5.660549 0.115400 -1.414395

C -5.425259 1.596292 -1.493160

O -6.868673 -0.243209 -1.849584

H -6.354071 2.143439 -1.697370

H -4.963472 1.934889 -0.555422

H -4.699383 1.789068 -2.298899

C -7.201860 -1.650050 -1.840255

H -7.104236 -2.030013 -0.810992

H -6.472479 -2.190717 -2.464226

C -8.612304 -1.799686 -2.364428

H -8.892528 -2.864864 -2.368557

H -9.331832 -1.257371 -1.730631

H -8.697174 -1.417657 -3.394185

**TS4-CF3-EA_ligand**

C -7.648398 -2.103484 1.384413

C -8.272955 -3.315166 1.057046

C -7.720441 -4.142413 0.073138

C -6.549691 -3.760730 -0.580129

C -5.906433 -2.542796 -0.262415

C -6.479173 -1.719216 0.733617

H -8.079405 -1.456607 2.152519

H -9.190831 -3.612223 1.570952

H -8.204826 -5.087511 -0.184583

H -6.115864 -4.405317 -1.349028

H -6.006224 -0.770757 1.000636

C -4.690413 -2.201952 -0.977989

C -3.896153 -1.105013 -0.855754

H -4.353579 -2.922728 -1.730107

C -3.913836 0.058571 -0.056103

C -3.293777 1.075645 0.337062

C -3.130477 2.226533 1.182640

C -2.019508 3.088705 1.125642

C -4.157270 2.480628 2.122297

C -1.942824 4.179434 1.991576

H -1.217658 2.898193 0.416017

C -4.068476 3.574529 2.980242

H -5.021956 1.815373 2.170318

C -2.960819 4.428263 2.918361

H -1.072761 4.838626 1.941001

H -4.868422 3.760639 3.701250

H -2.891992 5.285640 3.592966

C -2.690181 -0.819983 -1.692331

C -2.050376 0.455778 -1.217643

C -2.050813 1.541334 -2.239458

O -1.497641 1.357888 -3.307606

O -2.660079 2.669530 -1.893022

O -2.277841 -1.480599 -2.622661

C -2.576203 3.778433 -2.824411

H -1.521452 4.089358 -2.889879

H -2.887961 3.427494 -3.819741

C -3.462631 4.891531 -2.315219

H -3.135910 5.244669 -1.325019

H -3.416993 5.740764 -3.015289

H -4.511407 4.562841 -2.242084

Rh -0.040404 0.063945 -0.395643

Rh 2.342827 -0.315528 0.279639

O 0.455573 2.087399 -0.688267

O -0.464490 0.429640 1.635188

O 1.873529 -2.334396 0.600313

O 2.807992 -0.751303 -1.717963

C 1.851913 -0.637962 -2.543596

C 1.663316 2.428934 -0.491600

C 0.500190 0.358397 2.455057

C 0.677869 -2.683733 0.380444

C 0.406662 -4.171223 0.667646

C 0.093907 0.619618 3.915200

C 1.927231 3.919952 -0.755400

C 2.259649 -0.887978 -4.004835

F -0.847833 -4.535866 0.352397

F 1.244882 -4.948411 -0.030352

F 0.590762 -4.437504 1.969479

F -0.846595 -0.251180 4.307519

F 1.132065 0.505726 4.761851

F -0.408396 1.854667 4.055281

F 3.212667 4.254326 -0.564446

F 1.601989 4.246800 -2.014026

F 1.183464 4.680383 0.066102

F 3.161952 0.022607 -4.402782

F 2.819847 -2.099206 -4.137449

F 1.218273 -0.824263 -4.848300

N 2.630118 1.655550 -0.098100

N -0.296646 -1.923151 -0.031000

N 1.741275 0.095705 2.168468

N 0.620241 -0.330939 -2.270288

H -0.028542 -0.232086 -3.052250

H 2.387724 0.069486 2.955545

H -1.190417 -2.394033 -0.153058

H 3.543805 2.096639 -0.001229

C 5.059094 -2.668452 -0.234315

H 5.931049 -3.312803 -0.399019

H 4.227380 -3.253622 0.186966

H 4.694745 -2.250918 -1.184062

C 5.377370 -1.543598 0.704274

O 4.609718 -0.633960 0.995514

O 6.599641 -1.618000 1.219141

C 7.016109 -0.592320 2.151344

H 6.314254 -0.582013 3.000291

H 6.949897 0.386398 1.650355

C 8.428149 -0.906506 2.590512

H 8.771461 -0.138262 3.301123

H 8.480526 -1.886256 3.091053

H 9.118791 -0.914770 1.732532

**TS4-Me-EA_ligand**

C -6.781044 -2.816734 1.830825

C -7.359921 -4.026104 1.422041

C -6.918669 -4.648040 0.249082

C -5.905480 -4.064589 -0.510845

C -5.311463 -2.845254 -0.114403

C -5.769253 -2.231681 1.073520

H -7.123111 -2.329372 2.747316

H -8.153816 -4.481837 2.019452

H -7.366305 -5.591436 -0.073590

H -5.559579 -4.550711 -1.426990

H -5.328203 -1.289537 1.408731

C -4.259341 -2.287317 -0.948277

C -3.549784 -1.141623 -0.786802

H -3.988470 -2.863961 -1.838502

C -3.539167 -0.095963 0.157010

C -2.941589 0.927100 0.571566

C -2.766105 2.000422 1.513802

C -1.696885 2.915121 1.464589

C -3.731590 2.120974 2.539557

C -1.601863 3.923794 2.423566

H -0.941907 2.825004 0.685757

C -3.625596 3.133823 3.491441

H -4.565828 1.417206 2.579832

C -2.560577 4.039810 3.437274

H -0.767091 4.628211 2.379294

H -4.380113 3.216345 4.277876

H -2.478169 4.835066 4.182763

C -2.511534 -0.628264 -1.747442

C -1.872756 0.607576 -1.172524

C -2.034405 1.828547 -2.010110

O -1.584147 1.872448 -3.133486

O -2.696284 2.837146 -1.429621

O -2.257708 -1.095273 -2.833031

C -2.838922 4.051842 -2.199813

H -1.838306 4.485422 -2.359740

H -3.251869 3.798986 -3.188655

C -3.744494 4.993997 -1.438833

H -3.319959 5.255478 -0.457197

H -3.873448 5.923200 -2.016349

H -4.738942 4.547236 -1.281054

Rh 0.197516 0.120419 -0.501288

Rh 2.513141 -0.411104 0.085760

O 0.775693 2.108770 -0.562004

O -0.158187 0.236973 1.533261

O -0.190973 -1.904865 -0.388288

O 0.794811 -0.098750 -2.463827

O 2.920326 -0.611198 -1.925532

O 1.938989 -2.393437 0.165037

O 2.912050 1.617985 -0.042008

O 1.987007 -0.185839 2.067460

C 0.784725 0.064268 2.366510

C 0.742123 -2.713461 -0.091693

C 1.983050 -0.416210 -2.758371

C 1.983723 2.427526 -0.326157

C 2.301384 -0.614678 -4.220761

H 3.336084 -0.313051 -4.435887

H 2.203870 -1.687473 -4.458651

H 1.597517 -0.055885 -4.851320

C 0.395169 -4.183051 -0.081707

H 1.068041 -4.736877 0.586749

H -0.652033 -4.332801 0.214692

H 0.519987 -4.579289 -1.103709

C 2.319494 3.899408 -0.352132

H 2.099645 4.328218 0.640407

H 3.386124 4.052472 -0.563819

H 1.702227 4.424685 -1.093906

C 0.422825 0.156289 3.829440

H -0.271927 -0.660998 4.081780

H 1.317093 0.082328 4.461184

H -0.100441 1.104288 4.026143

O 4.800128 -0.951457 0.726229

C 5.857947 -0.584233 0.239374

C 5.986554 0.340370 -0.935653

O 7.034153 -0.969634 0.731229

H 7.016630 0.375029 -1.311462

H 5.287549 0.019951 -1.720455

H 5.675111 1.348306 -0.618926

C 7.035789 -1.843132 1.883921

H 6.481415 -2.761125 1.632626

H 6.498195 -1.344311 2.705868

C 8.474456 -2.134927 2.246457

H 8.505127 -2.799286 3.124438

H 9.000091 -2.635613 1.417899

H 9.017610 -1.209641 2.496070

**BnOH**

C -0.438015 -0.277002 0.000043

C -0.007310 1.054798 0.000071

C 1.359455 1.355704 0.000034

C 2.308907 0.331369 -0.000032

C 1.884053 -1.002296 -0.000050

C 0.520869 -1.302281 -0.000020

H -0.754585 1.848994 0.000111

H 1.682551 2.400341 0.000051

H 3.376047 0.568141 -0.000061

H 2.618944 -1.811777 -0.000103

H 0.197047 -2.348295 -0.000054

C -1.910626 -0.626116 0.000124

H -2.124159 -1.257189 0.888715

H -2.124145 -1.257698 -0.888108

O -2.693976 0.545225 -0.000208

H -3.623892 0.290626 0.000103

**Int2-CP**

C 1.291615 1.126941 2.818007

C 1.858411 0.901466 1.422729

C 2.976091 0.554541 0.885045

C 4.129192 0.040339 0.310605

C 4.186708 -1.325897 -0.085159

C 5.275104 0.862214 0.103123

C 5.348459 -1.844796 -0.636490

H 3.291509 -1.934825 0.044369

C 6.421131 0.330302 -0.461006

H 5.222339 1.914552 0.387297

C 6.481413 -1.030332 -0.837279

H 5.386493 -2.896267 -0.932226

H 7.294622 0.968024 -0.619929

C 0.204074 1.984098 2.168878

C 0.673881 1.586548 0.802265

C 0.972200 2.404934 -0.388247

O 1.988011 2.260972 -1.048553

O 0.036498 3.322130 -0.653517

O -0.619664 2.719282 2.641602

C 0.208755 4.095018 -1.847761

H 1.182366 4.611344 -1.809130

H 0.238359 3.409000 -2.709612

C -0.946026 5.071167 -1.938832

H -0.965214 5.739836 -1.064539

H -0.849875 5.687473 -2.846754

H -1.907182 4.536385 -1.979660

Rh -0.829495 -0.028292 0.106050

Rh -2.524352 -1.593762 -0.675397

O -0.613592 0.545832 -1.868255

O 0.568775 -1.555687 -0.189483

O -1.189154 -0.751162 2.011441

O -2.352196 1.323887 0.332019

O -3.922069 -0.114511 -0.409948

O -2.786506 -2.166819 1.282072

O -2.164911 -0.924990 -2.586551

O -1.028703 -2.988487 -0.891901

C 0.163444 -2.682525 -0.615715

C -2.088394 -1.631005 2.187975

C -3.543368 1.009105 0.034064

C -1.309777 -0.012586 -2.770625

C 2.024016 1.420338 4.103273

C 1.184007 0.182720 3.981658

H 3.112805 1.317295 4.106771

H 1.634326 2.264872 4.680212

H 1.685700 -0.784349 3.892008

H 0.205606 0.153109 4.467572

C 7.728990 -1.583315 -1.467441

H 7.725258 -2.682047 -1.491810

H 7.822376 -1.224149 -2.507486

H 8.630319 -1.245275 -0.932210

C -2.349549 -2.055101 3.616647

H -1.399061 -2.282116 4.121309

H -2.818504 -1.217363 4.156943

H -3.015624 -2.926082 3.646921

C -4.588358 2.088419 0.200668

H -4.359286 2.699499 1.084153

H -4.557967 2.748846 -0.681333

H -5.590231 1.646496 0.274645

C -1.113157 0.484575 -4.185948

H -1.716036 1.396540 -4.327549

H -0.059497 0.746450 -4.353875

H -1.448956 -0.270047 -4.908671

C 1.212076 -3.759797 -0.810751

H 1.963162 -3.414351 -1.537845

H 1.727528 -3.951466 0.143235

H 0.750203 -4.686736 -1.171677

**Rh_2_(OAc)_4_**

Rh 0.000005 -0.007662 1.189094

Rh 0.000021 -0.007646 -1.189095

O -1.451559 1.439442 1.125895

O 1.448910 1.442101 1.125942

O 1.449164 -1.455515 1.125915

O -1.446506 -1.458164 1.125883

O -1.446706 -1.457935 -1.125955

O 1.448955 -1.455730 -1.125922

O -1.451309 1.439686 -1.125903

O 1.449142 1.441898 -1.125857

C 1.859316 1.858886 0.000049

C 1.866227 -1.865655 -0.000004

C -1.863105 -1.868761 -0.000043

C -1.862429 1.855756 -0.000004

C 2.967408 -2.896861 0.000014

H 2.911987 -3.515669 -0.905016

H 3.937811 -2.373813 0.000634

H 2.911285 -3.516334 0.904555

C -2.962628 -2.901734 -0.000018

H -3.933869 -2.380244 0.001121

H -2.906517 -3.520151 -0.905273

H -2.905208 -3.521417 0.904298

C -2.896520 2.954466 0.000014

H -2.376222 3.926350 0.000672

H -3.515090 2.897655 -0.905107

H -3.515822 2.896896 0.904593

C 2.891557 2.959333 -0.000008

H 3.511821 2.901908 -0.903942

H 2.369611 3.930333 -0.002255

H 3.509341 2.904490 0.905763

**Int3-CP**

C -0.288800 -3.034492 -0.096613

C -0.007632 -1.541356 -0.104973

C -0.743307 -0.409663 -0.045215

C -2.216069 -0.282263 0.013412

C -2.811507 0.523063 1.007908

C -3.067633 -0.913150 -0.909001

C -4.195245 0.657516 1.089296

H -2.170026 1.037226 1.727607

C -4.456275 -0.780588 -0.816445

H -2.633601 -1.489714 -1.727440

C -5.049390 0.001196 0.184372

H -4.626747 1.281064 1.878412

H -5.090929 -1.284211 -1.551454

C 1.221265 -3.270516 -0.090228

C 1.442740 -1.812771 -0.069748

C 2.559055 -1.025456 0.117122

O 2.523248 0.278945 0.319457

O 3.761320 -1.574511 0.116995

O 1.889339 -4.277143 -0.082857

C 4.914918 -0.777730 0.439118

H 5.005773 0.040046 -0.294114

H 4.771104 -0.317464 1.430254

C 6.120659 -1.693156 0.412390

H 6.247206 -2.145432 -0.582829

H 7.029533 -1.120684 0.654376

H 6.011905 -2.505168 1.146912

C -1.273222 -3.946597 -0.788264

C -1.271493 -3.853285 0.707830

H -2.110410 -3.507263 -1.333606

H -0.832500 -4.836420 -1.249576

H -2.093198 -3.313992 1.187687

H -0.853361 -4.683280 1.286272

C -6.547867 0.136727 0.298322

H -7.060679 -0.282044 -0.580209

H -6.929159 -0.392519 1.188870

H -6.851681 1.191524 0.398681

C 2.289376 4.608041 -0.629480

C 1.601139 5.499180 0.198278

C 0.312784 5.182453 0.642846

C -0.283020 3.979770 0.258338

C 0.398723 3.077933 -0.574385

C 1.691293 3.403323 -1.010731

H 3.295653 4.851012 -0.980074

H 2.066983 6.442106 0.496001

H -0.229982 5.877725 1.288347

H -1.291630 3.734959 0.602909

H 2.235129 2.705361 -1.653037

C -0.239938 1.769717 -0.958133

H -1.322153 1.895784 -1.116538

H 0.199089 1.367889 -1.887682

O -0.040951 0.808911 0.106020

H 1.566693 0.576510 0.337224

**Int4-CP**

C 0.843072 -0.345978 2.546384

C 0.601947 -0.540903 1.058047

C -0.466313 -0.298699 0.270747

C -1.760398 0.293197 0.710181

C -2.149060 1.563374 0.240814

C -2.648478 -0.391329 1.554387

C -3.366249 2.127063 0.618596

H -1.478373 2.118089 -0.420125

C -3.867437 0.179869 1.933454

H -2.392714 -1.396102 1.896846

C -4.249191 1.448688 1.477416

H -3.636427 3.119286 0.244160

H -4.540511 -0.381040 2.588450

C 2.214785 -0.976298 2.364919

C 1.968906 -1.099816 0.909187

C 2.834989 -1.554211 -0.067983

O 2.746962 -1.425015 -1.376219

O 3.899134 -2.231762 0.346986

O 3.109886 -1.220108 3.139592

C 4.997169 -2.477417 -0.546939

H 4.662457 -3.139250 -1.361852

H 5.313915 -1.525615 -1.004878

C 6.108507 -3.105922 0.268058

H 5.776848 -4.055881 0.713888

H 6.978599 -3.307570 -0.376026

H 6.419408 -2.436262 1.083773

C 0.002206 -0.491328 3.790038

C 0.575220 0.847818 3.436023

H -1.074225 -0.634020 3.679094

H 0.470810 -1.053500 4.604401

H -0.108548 1.610114 3.051594

H 1.426127 1.224298 4.013039

C -5.556028 2.077793 1.895099

H -6.200414 1.360022 2.424102

H -5.388659 2.934749 2.570451

H -6.115677 2.460209 1.025798

C -4.342571 -1.858643 -2.333355

C -4.743184 -3.064096 -1.744632

C -3.799760 -3.865379 -1.097987

C -2.461597 -3.460844 -1.038319

C -2.050022 -2.259157 -1.629688

C -3.006226 -1.461622 -2.278461

H -5.077556 -1.225992 -2.837836

H -5.790126 -3.375153 -1.789250

H -4.104899 -4.806050 -0.632225

H -1.727130 -4.087647 -0.524074

H -2.698503 -0.516109 -2.732077

C -0.595597 -1.846088 -1.597997

H -0.006522 -2.556824 -0.996600

H -0.175111 -1.842039 -2.616858

O -0.370135 -0.510147 -1.116548

H 2.261665 -0.608447 -1.704991

C 1.680419 3.003106 -1.885225

C 1.903262 2.769412 -0.519971

C 1.768667 3.806275 0.407959

C 1.413788 5.090403 -0.016109

C 1.186655 5.331388 -1.374641

C 1.314143 4.292307 -2.300531

H 2.174115 1.768060 -0.179046

H 1.941930 3.606005 1.468312

H 1.309063 5.900086 0.710568

H 0.900791 6.330288 -1.714513

H 1.124748 4.485364 -3.361085

C 1.867638 1.900809 -2.909570

H 2.923050 1.863802 -3.231069

H 1.273220 2.131159 -3.813321

O 1.561544 0.602076 -2.440262

H 0.663943 0.512992 -2.046466

**TS2-CP**

C 1.250468 0.540414 3.002778

C 1.831104 0.641148 1.605435

C 2.946348 0.548803 0.942311

C 4.088012 0.047429 0.303984

C 4.072820 -1.262296 -0.241632

C 5.276181 0.818806 0.198385

C 5.214624 -1.785377 -0.837140

H 3.142855 -1.830593 -0.188386

C 6.399988 0.288906 -0.413800

H 5.280404 1.833750 0.599757

C 6.392999 -1.022939 -0.936908

H 5.195965 -2.798428 -1.247225

H 7.309082 0.891199 -0.493141

C 0.183588 1.553134 2.559609

C 0.671254 1.438319 1.146268

C 1.073314 2.354322 0.083036

O 2.157178 2.169111 -0.484067

O 0.253680 3.349856 -0.219055

O -0.627471 2.191752 3.172314

C 0.602465 4.173830 -1.344483

H 1.603297 4.604556 -1.177875

H 0.662571 3.532180 -2.237534

C -0.461014 5.242596 -1.481275

H -0.513737 5.864056 -0.574238

H -0.229552 5.895748 -2.337280

H -1.450659 4.789956 -1.645185

Rh -0.862406 -0.002416 0.123710

Rh -2.544754 -1.380362 -0.963463

O -0.352369 0.679811 -1.762401

O 0.451303 -1.611907 -0.099864

O -1.513921 -0.810159 1.910752

O -2.297430 1.456882 0.256519

O -3.839331 0.205393 -0.812064

O -3.131414 -2.013591 0.901358

O -1.861817 -0.660869 -2.766884

O -1.151834 -2.892726 -1.040492

C 0.018030 -2.691336 -0.612629

C -2.503409 -1.605908 1.919479

C -3.454552 1.257602 -0.222187

C -0.945378 0.208661 -2.781679

C -4.465419 2.366227 -0.039465

H -4.925612 2.259090 0.956353

H -3.968704 3.345023 -0.074498

H -5.255061 2.295777 -0.798669

C -0.534464 0.764117 -4.127661

H -1.108999 1.685176 -4.319984

H 0.533365 1.021544 -4.125975

H -0.762228 0.044420 -4.924586

C 1.005041 -3.833564 -0.743323

H 1.697751 -3.615721 -1.572479

H 1.597850 -3.931184 0.177568

H 0.480614 -4.772524 -0.959880

C -2.953680 -2.109135 3.272884

H -2.150769 -2.715464 3.720953

H -3.132025 -1.253464 3.941067

H -3.862873 -2.715360 3.179042

C 1.960112 0.497125 4.331857

C 1.090237 -0.649663 3.907353

H 1.579358 1.190488 5.088337

H 3.045610 0.363833 4.326785

H 0.101640 -0.762730 4.358938

H 1.567515 -1.582532 3.595992

C 7.627006 -1.578102 -1.595060

H 7.482442 -2.614350 -1.931184

H 7.910228 -0.969935 -2.470607

H 8.485243 -1.556652 -0.902919

**TS3-CP**

C 2.208474 2.556801 1.946943

C 2.324740 1.748842 0.655695

C 2.619706 0.520300 0.262307

C 3.544972 -0.441223 -0.163642

C 4.923564 -0.109299 -0.315359

C 3.139403 -1.776819 -0.435884

C 5.844948 -1.072172 -0.693123

H 5.239201 0.918138 -0.122158

C 4.071791 -2.723114 -0.839239

H 2.084003 -2.037845 -0.340413

C 5.435052 -2.394285 -0.969919

H 6.900777 -0.805757 -0.792072

H 3.746005 -3.744073 -1.054292

C 1.433987 3.598916 1.104926

C 1.645515 2.773458 -0.077101

C 1.160007 2.440969 -1.385710

O 1.380776 1.316296 -1.855197

O 0.455785 3.376458 -2.031563

O 0.862442 4.618589 1.418565

C -0.060496 3.023138 -3.325684

H 0.783631 2.784152 -3.994256

H -0.668228 2.110582 -3.228650

C -0.863730 4.201505 -3.837487

H -0.237763 5.104068 -3.913701

H -1.269356 3.976480 -4.836678

H -1.704884 4.427287 -3.163680

Rh -0.616846 -0.386021 -0.153778

Rh -2.731047 -1.185688 0.627289

O -1.559172 0.093031 -1.922976

O -0.230566 -2.287942 -0.860052

O 0.160460 -0.949188 1.678496

O -1.121702 1.441610 0.601617

O -3.107362 0.694998 1.349942

O -1.841030 -1.687509 2.400298

O -3.544607 -0.659945 -1.173961

O -2.234757 -3.026609 -0.142121

C -1.117069 -3.186148 -0.715122

C -0.603287 -1.463070 2.550887

C -2.238798 1.606134 1.181330

C -2.799314 -0.136633 -2.055487

C -3.443159 0.272139 -3.358930

H -3.626738 1.358676 -3.337399

H -2.764578 0.062952 -4.197329

H -4.401578 -0.244448 -3.494694

C -0.833974 -4.548510 -1.302884

H 0.244979 -4.701490 -1.432332

H -1.267940 -5.333242 -0.668665

H -1.316299 -4.609850 -2.292109

C 0.002677 -1.805827 3.891426

H 1.065923 -2.056162 3.780291

H -0.077551 -0.924193 4.548340

H -0.547084 -2.632207 4.360512

C -2.564444 2.989777 1.684499

H -3.006650 2.925276 2.689034

H -1.670815 3.627462 1.686353

H -3.323588 3.433934 1.020388

C 3.204762 2.764531 3.054189

C 1.885047 2.091837 3.336172

H 3.348321 3.797336 3.387127

H 4.109664 2.149419 3.058780

H 1.113019 2.664039 3.860423

H 1.876167 1.015409 3.525614

C 6.437528 -3.419512 -1.424439

H 6.057773 -4.444312 -1.304867

H 6.673404 -3.275148 -2.493752

H 7.385803 -3.328311 -0.872398

**TS4-CP**

C 0.113379 0.149237 3.253361

C 0.762695 0.413656 1.877862

C 1.883797 -0.100814 1.480689

C 2.829450 -1.063669 1.143585

C 2.393296 -2.288562 0.563494

C 4.219873 -0.878680 1.387195

C 3.311546 -3.282768 0.256902

H 1.326888 -2.408375 0.368254

C 5.121587 -1.877951 1.070868

H 4.560986 0.064466 1.815839

C 4.686656 -3.095410 0.499523

H 2.968290 -4.221068 -0.186050

H 6.187905 -1.722321 1.252412

C -0.937386 1.194186 2.870226

C -0.338802 1.344869 1.515017

C -0.192453 2.572079 0.728479

O 0.684008 2.798725 -0.098731

O -1.136000 3.465872 1.027861

O -1.827144 1.683098 3.512010

C -1.170568 4.673191 0.255285

H -0.212958 5.206240 0.375438

H -1.265990 4.406560 -0.808736

C -2.342853 5.499545 0.742275

H -2.239349 5.736994 1.812116

H -2.397820 6.444619 0.179154

H -3.288929 4.954074 0.605112

Rh -1.651852 -0.020749 0.062448

Rh -2.906736 -1.337471 -1.544514

O -0.009929 -0.243453 -1.154867

O -1.173258 -1.816543 0.999732

O -3.379627 0.095173 1.169392

O -2.240158 1.635591 -1.024767

O -3.373851 0.397230 -2.527706

O -4.554472 -1.098084 -0.342445

O -1.166316 -1.468939 -2.651957

O -2.394765 -3.014600 -0.469349

C -1.676173 -2.899529 0.560964

C -4.444009 -0.439793 0.734974

C -2.948817 1.495331 -2.068694

C -0.119144 -0.889115 -2.244224

C 0.757177 0.003752 4.607628

C -0.045532 -1.125305 4.026569

H 1.845512 -0.097623 4.646986

H 0.315767 0.609544 5.405261

H 0.487159 -2.005859 3.658729

H -1.049120 -1.321028 4.415443

C 5.689645 -4.158061 0.147345

H 6.310315 -4.418758 1.020451

H 5.208180 -5.073723 -0.222984

H 6.379004 -3.792124 -0.632645

C 6.661995 0.527261 -2.201059

C 7.782154 1.069369 -1.562928

C 7.603845 2.002924 -0.536764

C 6.315147 2.386530 -0.154906

C 5.184900 1.852289 -0.793429

C 5.373894 0.915393 -1.819672

H 6.792023 -0.201604 -3.006091

H 8.788914 0.767399 -1.863594

H 8.471852 2.432159 -0.028941

H 6.184480 3.114178 0.652509

H 4.497570 0.491499 -2.312292

C 3.796462 2.313311 -0.402790

H 3.797858 2.589467 0.672260

H 3.568445 3.251781 -0.949818

O 2.829978 1.332979 -0.685880

H 1.945463 1.761118 -0.589589

C -1.411983 -4.156830 1.361166

H -1.428396 -5.037773 0.705947

H -0.458361 -4.088124 1.901521

H -2.216858 -4.271104 2.105743

C -5.694623 -0.249137 1.561679

H -6.323618 -1.148473 1.512043

H -5.436006 -0.003505 2.599364

H -6.270860 0.588998 1.136877

C -3.295484 2.754953 -2.829840

H -2.380817 3.156773 -3.294100

H -4.036991 2.543782 -3.610020

H -3.676928 3.517456 -2.135258

C 1.113563 -0.969219 -3.116011

H 1.424170 -2.021143 -3.214847

H 0.866862 -0.608521 -4.125941

H 1.924658 -0.370685 -2.682525

**TS5-CP**

C 0.332864 -2.231102 1.271659

C 0.683337 -0.906976 0.649624

C 0.025804 0.139066 -0.050565

C 0.313512 1.597005 0.188270

C -0.223607 2.551361 -0.697295

C 1.113448 2.058331 1.244287

C 0.021694 3.910997 -0.520375

H -0.838490 2.212623 -1.534713

C 1.366774 3.422644 1.406971

H 1.544037 1.345284 1.950765

C 0.825193 4.377473 0.534659

H -0.412095 4.628042 -1.224028

H 1.993660 3.751442 2.241035

C 1.845096 -2.523220 1.296396

C 2.072089 -1.197220 0.647580

C 2.853608 -0.728565 -0.441224

O 2.340392 0.096206 -1.263080

O 4.043855 -1.254196 -0.664012

O 2.498735 -3.465797 1.653724

C 4.747963 -0.878641 -1.866868

H 4.156249 -1.202023 -2.738334

H 4.819286 0.219870 -1.907341

C 6.108410 -1.540536 -1.827990

H 6.011823 -2.635639 -1.778239

H 6.674193 -1.281625 -2.736287

H 6.684280 -1.204815 -0.952270

C -0.742484 -3.228146 0.903355

C -0.712687 -2.643594 2.282165

H -1.512337 -2.885391 0.207727

H -0.411701 -4.263113 0.771370

H -1.475837 -1.911525 2.560075

H -0.353182 -3.270106 3.104422

C 1.078493 5.853373 0.720293

H 1.836425 6.040117 1.495405

H 1.426902 6.322491 -0.214558

H 0.157755 6.383145 1.020124

C -6.074070 -0.463021 0.026776

C -6.162508 -0.988035 -1.267398

C -5.004673 -1.117793 -2.037714

C -3.763945 -0.724558 -1.523558

C -3.668626 -0.198037 -0.228747

C -4.836306 -0.073167 0.541159

H -6.973580 -0.360247 0.639490

H -7.130562 -1.296260 -1.670558

H -5.064122 -1.529073 -3.049026

H -2.857292 -0.826457 -2.121285

H -4.775897 0.333409 1.555754

C -2.348598 0.261959 0.347406

H -2.351031 1.363531 0.447848

H -2.223858 -0.142991 1.370517

O -1.275519 -0.158558 -0.480611

H 1.110985 0.090496 -0.999255

**TS6-CP**

C 0.882551 -1.008187 2.415406

C 0.392775 -0.962608 0.986432

C 0.939821 -0.362872 -0.163921

C 0.704785 -0.846573 -1.563091

C 1.043763 -0.012637 -2.647746

C 0.163872 -2.108443 -1.855524

C 0.851289 -0.428370 -3.962882

H 1.467608 0.974368 -2.446741

C -0.034119 -2.516101 -3.177060

H -0.087286 -2.791346 -1.041804

C 0.301298 -1.688279 -4.257376

H 1.130074 0.241602 -4.781982

H -0.451808 -3.508567 -3.370345

C -0.417388 -1.748784 2.749013

C -0.840297 -1.587767 1.341290

C -2.113829 -1.714073 0.693421

O -2.494363 -1.035633 -0.287788

O -2.935803 -2.595045 1.258564

O -0.858419 -2.214761 3.770931

C -4.289822 -2.666589 0.777887

H -4.744454 -1.664138 0.836573

H -4.276694 -2.952397 -0.286235

C -5.027286 -3.677671 1.630655

H -5.020976 -3.376551 2.689154

H -6.073093 -3.759119 1.295695

H -4.559820 -4.671250 1.554832

C 1.523756 0.063451 3.266679

C 2.233254 -1.239951 3.043234

H 1.913629 0.942069 2.747233

H 1.063113 0.247979 4.242557

H 3.120692 -1.239017 2.406712

H 2.249356 -1.967386 3.861155

C 0.066910 -2.118358 -5.684604

H -0.112803 -3.201476 -5.756955

H -0.812840 -1.608616 -6.115406

H 0.927036 -1.871989 -6.327806

C 6.909157 0.658010 0.626946

C 6.804606 2.045011 0.772298

C 5.565708 2.665062 0.587853

C 4.438418 1.907337 0.255689

C 4.535266 0.516471 0.106918

C 5.781705 -0.099339 0.300181

H 7.871887 0.161902 0.775585

H 7.684663 2.638590 1.032787

H 5.474313 3.748422 0.704062

H 3.469207 2.388687 0.116411

H 5.871088 -1.185119 0.195739

C 3.341942 -0.317351 -0.297606

H 3.377251 -0.510891 -1.386795

H 3.382754 -1.306653 0.195821

O 2.135372 0.353966 0.040582

H -0.183326 0.595447 -0.200991

C -2.899708 2.781214 0.207524

C -3.925432 3.017410 1.133088

C -5.053189 3.760545 0.770571

C -5.170851 4.265209 -0.526849

C -4.155362 4.024904 -1.459187

C -3.025347 3.290645 -1.094479

H -3.843652 2.615599 2.147325

H -5.845164 3.937414 1.502719

H -6.054314 4.841105 -0.813859

H -4.244587 4.412826 -2.477199

H -2.237016 3.095978 -1.825354

C -1.663147 2.020394 0.621288

H -1.833252 1.481167 1.567754

H -0.815739 2.708164 0.777182

O -1.240397 1.101498 -0.401854

H -1.847571 0.232735 -0.437592
